# Photoinduced remote C–N/C bond formation *via* ring-opening 1,4-difunctionalization of methylenecyclobutanes

**DOI:** 10.1039/d6sc02950g

**Published:** 2026-07-31

**Authors:** Yan Zhang, Yahong Chen, Yecai Liu, Na Wu, Wei Wei, Chen Zhu

**Affiliations:** a Key Laboratory of the Ministry of Education for Advanced Catalysis Materials, Zhejiang Key Laboratory of Advanced Catalysis and Adsorption Materials, Zhejiang Normal University Jinhua 321004 China zhangyan001@zjnu.edu.cn; b Guangxi Key Laboratory of Bioactive Molecules Research and Evaluation, Guangxi Key Laboratory of Pharmaceutical Precision Detection and Screening, School of Pharmacy, Guangxi Medical University Nanning 530021 China weiw66@sr.gxmu.edu.cn; c Frontiers Science Center for Transformative Molecules, School of Chemistry and Chemical Engineering, Shanghai Key Laboratory for Molecular Engineering of Chiral Drugs, Shanghai Jiao Tong University 800 Dongchuan Road Shanghai 200240 China chzhu@sjtu.edu.cn

## Abstract

The construction of C–N bonds is a fundamental transformation in organic synthesis. While significant progress has been made in C–N bond formation at the alkene π-bond or at the allylic position, achieving remote C–N bond formation remains a considerable challenge. Herein, we report a photochemical strategy for remote 1,4-difunctionalization to forge C–N bonds, enabled by strain-induced ring-opening of methylenecyclobutanes. Using pyrazole or acetonitrile as nitrogen nucleophiles, this method delivers a range of bishomoallylic amine derivatives. Notably, the protocol is also extendable to C–C bond formation with carbon nucleophiles, including pyrroles, indoles, naphthol, and electron-rich arenes.

## Abstract 2

We report a photochemical strategy for remote 1,4-difunctionalization to forge C–N and C–C bonds, enabled by strain-induced ring-opening of methylenecyclobutanes.

## Graphical abstract

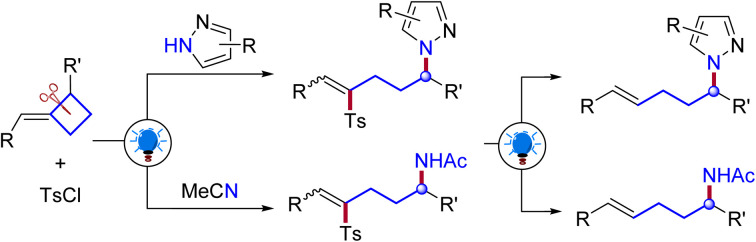

## Introduction

**Fig. 1:**
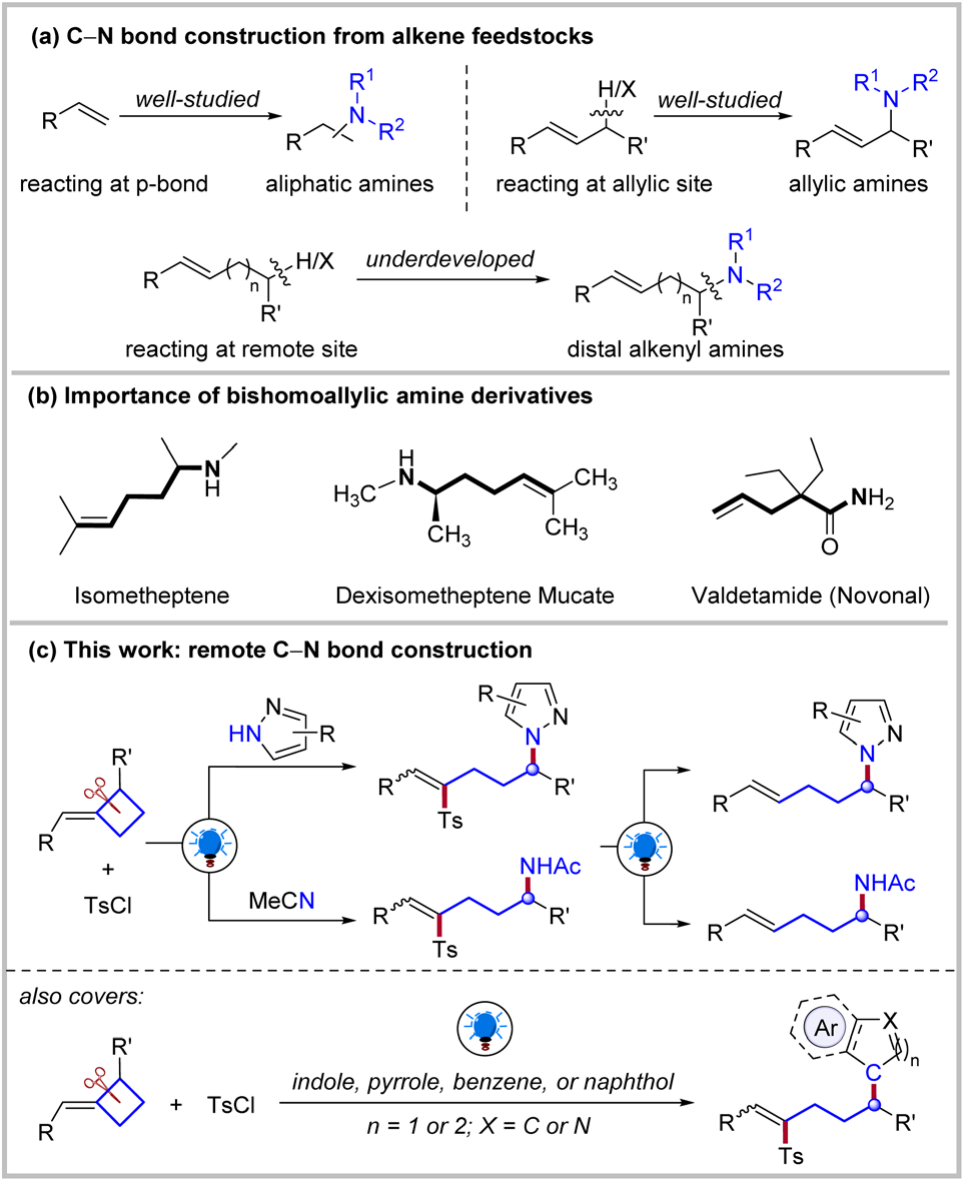
(a) C–N bond construction from alkene feedstocks. (b) Importance of bishomoallylic amine derivatives. (c) Remote C–N bond construction.

The formation of carbon–nitrogen (C–N) bonds represents a pivotal strategy in organic synthesis, serving as a cornerstone for the construction of diverse nitrogen-containing compounds, including pharmaceuticals,^1–3^ bioactive natural products,^4^ and advanced functional materials.^5^ The use of widely available alkenes as feedstocks to incorporate C–N bonds offers a practical and attractive approach. Over the past few years, direct alkene functionalization reactions have witnessed great progress in the field of C–N bond formation (Fig. 1a).^6^ One common strategy involves the addition of C–N bonds across the unsaturated C=C π-system, delivering aliphatic amines. Alternatively, C–N bonds can be installed at the allylic position *via* functionalization of adjacent σ-bonds (*e.g.*, C–H and C–X bonds), leading to allylic amines.^7^ In contrast, the installation of a C–N bond at a more remote site of an alkene remains underexplored.^8^

Ring-opening transformations provide an efficient strategy for remote functionalization,^9,10^ enabling access to products that are otherwise difficult to obtain by conventional methods. We envisioned that methylenecyclobutanes could serve as substrates to achieve remote C–N bond construction through cleavage of the cyclic C–C σ-bond while retaining the alkenyl C=C π-bond, thereby affording valuable bishomoallylic amine derivatives. Such motifs are widely present in numerous bioactive molecules (Fig. 1b).^11^ However, this hypothesis faces several uncertainties: (1) in the presence of an alkene, C–N bond formation at the C=C π-bond may preferentially occur; (2) the ring-opening of cyclobutyl rings is typically driven by high-energy heteroatom-centered radicals (*e.g.*, oxy and imino radicals),^12^ whereas alkyl radical-triggered ring-opening of cyclobutanes remains less developed.^13^

Motivated by our sustained interest in ring-opening reactions for versatile bond formation,^14^ we report herein a novel photochemical strategy for remote bond formation *via* the ring-opening 1,4-difunctionalization of methylenecyclobutanes (Fig. 1c). This transformation proceeds through a cascade process involving sulfonyl radical addition to the alkene, benzylic radical-triggered ring opening driven by ring strain, alkene regeneration, and subsequent C–N/C bond formation. Both pyrazole and acetonitrile can serve as nucleophilic nitrogen sources, delivering bishomoallylic amine derivatives. The incorporated sulfonyl group can be photocatalytically removed to afford *E*-configured alkenes, while the remaining alkene moiety provides versatile handles for further elaboration into valuable molecules. Notably, this method is also extendable to C–C bond formation by employing various carbon nucleophiles, including pyrroles, indoles, naphthol, and electron-rich arenes.

## Results and discussion

We commenced our studies by examining the reactivity of the photocatalyst fac-Ir(ppy)_3_ (ref. 15) in the presence of methylenecyclobutane 1a, pyrazole 2a, TsCl, and MeCN as solvents under blue-light irradiation (Table 1). Under these conditions, the desired remote 1,4-difunctionalized product 3a was obtained in 39% yield, with successful formation of the remote C–N bond (entry 1). Other photocatalysts, such as Ir(dtbbpy)(ppy)_2_PF_6_ and Ru(bpy)_3_(PF_6_)_2_, proved ineffective under otherwise identical conditions (entries 2 and 3). Replacement of MeCN with DMF or 1,4-dioxane led to lower efficiency. Interestingly, the use of a MeCN/DCM solvent mixture (v/v = 1 : 2) significantly improved the yield of 3a (entries 4–7). The addition of organic or inorganic bases generally increased the reaction yields; notably, Ag_2_CO_3_ afforded the desired product in a very good yield of 74% (entries 8–10). Reducing the catalyst loading resulted in a slight decrease in the yield (entry 11). Finally, by moderately increasing the equivalents of both 2a and TsCl, the yield of 3a was increased to 81% (entry 12).

**Table 1:** Reaction condition optimization^a^

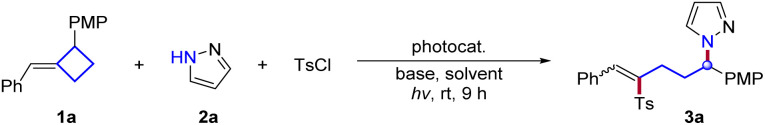				
Entry	Photocatalyst	Solvent	Base	Yield/%
1	fac-Ir(ppy)_3_	MeCN	—	39
2	Ir(dtbbpy)(ppy)_2_PF_6_	MeCN	—	0
3	Ru(bpy)_3_(PF_6_)_2_	MeCN	—	0
4	fac-Ir(ppy)_3_	DMF	—	17
5	fac-Ir(ppy)_3_	DCM	—	45
6	fac-Ir(ppy)_3_	1,4-Dioxane	—	25
7	fac-Ir(ppy)_3_	MeCN : DCM (1 : 2)	—	59
8	fac-Ir(ppy)_3_	MeCN : DCM (1 : 2)	Et_3_N	62
9	fac-Ir(ppy)_3_	MeCN : DCM (1 : 2)	K_3_PO_4_	60
10	fac-Ir(ppy)_3_	MeCN : DCM (1 : 2)	Ag_2_CO_3_	74
11^b^	fac-Ir(ppy)_3_	MeCN : DCM (1 : 2)	Ag_2_CO_3_	63
12^c^	fac-Ir(ppy)_3_	MeCN : DCM (1 : 2)	Ag_2_CO_3_	81

a Reaction conditions: 1a (0.2 mmol), 2a (0.6 mmol), TsCl (0.3 mmol), base (0.2 mmol), solvent (3 mL), photocatalyst (2 mol%), 9 h, rt. Yield of isolated products.

b 1 mol% fac-Ir(ppy)_3_.

c 2a (0.8 mmol), TsCl (0.6 mmol). PMP = *para*-methoxyphenyl. The *Z*/*E* ratio of compound 3a is about 3 : 2, and isomers *Z*- and *E*-(3a) cannot be separated by flash column chromatography.

**Scheme 1:**
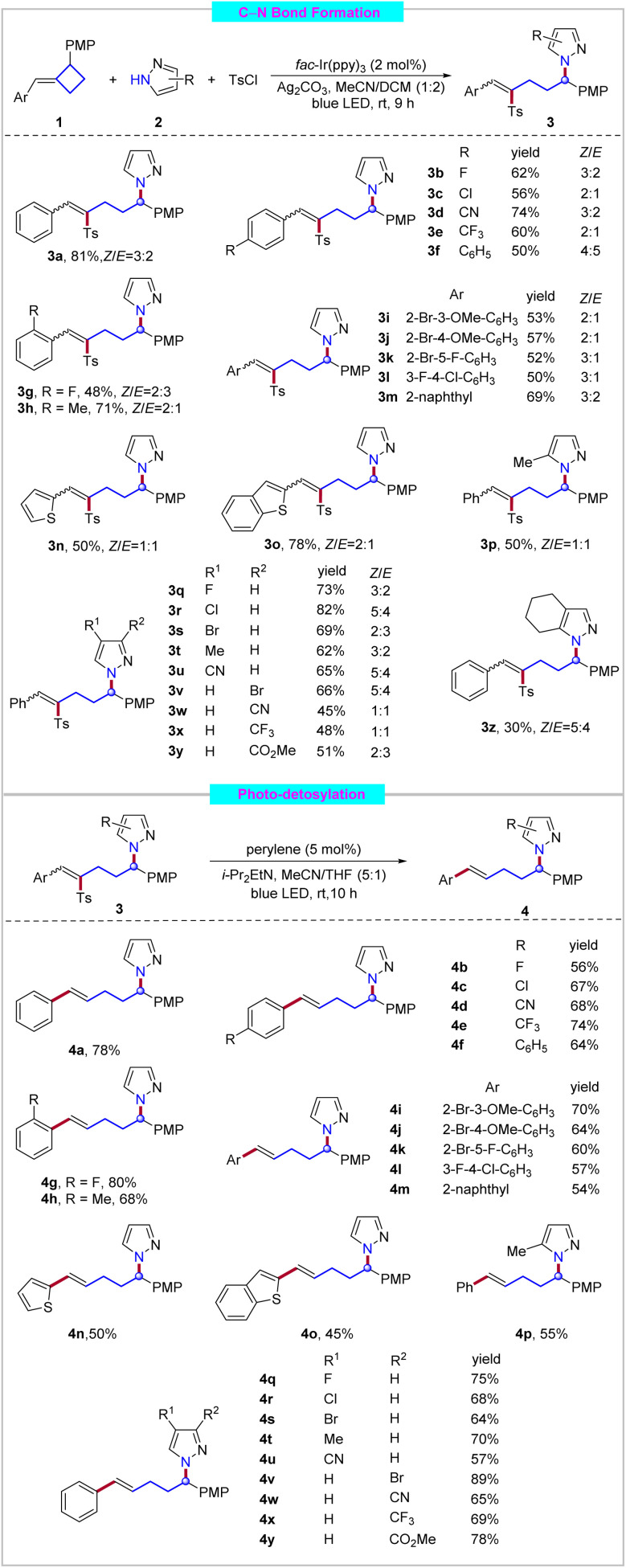
Scope investigation using pyrazoles as nucleophiles. Standard reaction conditions for C–N bond formation: 1 (0.2 mmol), 2 (0.8 mmol), fac-Ir(ppy)_3_ (2 mol%), TsCl (0.6 mmol), and Ag_2_CO_3_ (0.2 mmol) in a mixed solvent of MeCN/DCM (v/v = 1 : 2, 3 mL) at room temperature under N_2_ for 9 h and 30 W blue LEDs. Yields of isolated products are given.

With the optimal reaction conditions in hand, we evaluated the substrate scope using pyrazole as the nucleophile (Scheme 1, top). Both electron-withdrawing groups (EWGs) and electron-donating groups (EDGs) at the *para* position of the phenyl moiety of methylenecyclobutane 1 were tolerated, affording the desired remote 1,4-difunctionalized products in moderate to good yields (3b–3f). Notably, analogous outcomes were achieved when substituents were introduced at the most sterically hindered *ortho* position (3g and 3h). Furthermore, a range of substrates bearing multi-substituted arenes were found to be compatible with the transformation, delivering the corresponding products in moderate yields (3i–3l). Polycyclic moieties such as naphthalene were also tolerated, affording product 3m in 69% yield. Heterocycles including thiophene and benzothiophene were suitable as well, providing the desired products in 50% and 78% yields, respectively (3n, 3o). We further assessed the compatibility of the pyrazole ring 2 within the reaction. It was revealed that the 3-, 4-, and 5-positions of the pyrazole ring tolerated a variety of functional groups, including the electron-donating methyl group (3p and 3t), electron-withdrawing halogens (3q–3s and 3v), trifluoromethyl group (–CF_3_; 3x), cyano group (–CN; 3u and 3w) and ester group (–CO_2_Me; 3y). These target products were obtained in yields ranging from 55% to 82%. Furthermore, aliphatic rings on the pyrazole were tolerated (3z). Notably, the Ts group, strategically incorporated along with the pyrazolyl group, served a dual role as a versatile protecting group and an efficient leaving group in organic synthesis.^16^ Under photochemical conditions, the tosyl (Ts) group was readily removed from *Z*/*E* mixtures of 3, affording exclusively the *E*-configured alkenes 4 in overall yields of 45–89% (Scheme 1, bottom). This stereoselectivity resulted from single-electron reduction of the C=C bond to a radical anion intermediate, wherein the resultant C–C σ-bond underwent free rotation followed by radical desulfonylation, ultimately delivering the thermodynamically favored *E*-alkene products.^14*f*^

**Scheme 2:**
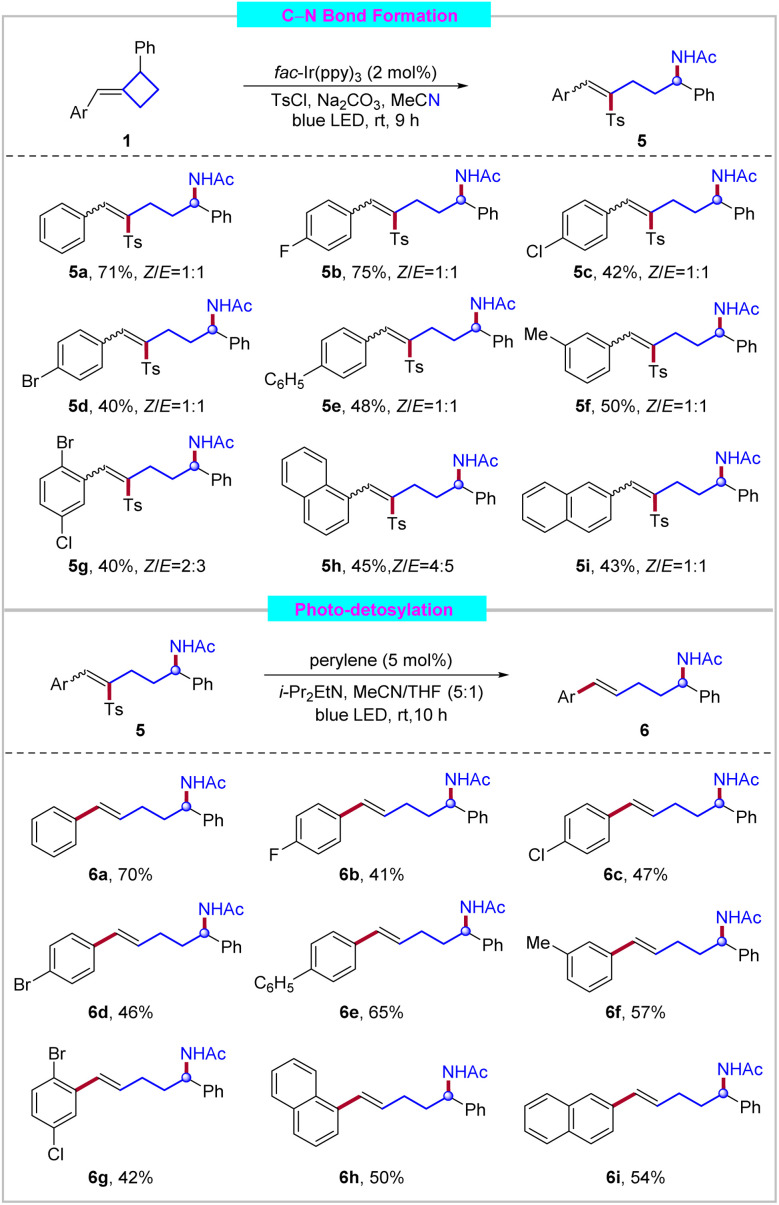
Scope investigation using acetonitrile as a nucleophile. Standard reaction conditions for C–N bond formation: 1 (0.2 mmol), fac-Ir(ppy)_3_ (2 mol%), TsCl (0.3 mmol), and Na_2_CO_3_ (0.3 mmol) in MeCN (3 mL) at room temperature under N_2_ for 9 h and 30 W blue LEDs. Yields of isolated products are given.

Subsequently, we employed acetonitrile as the nitrogen source to further investigate the construction of C–N bonds *via* a Ritter-type reaction. A variety of bishomoallylic amides were synthesized under similar conditions (Scheme 2, top). The phenyl ring of methylenecyclobutane 1 was found to accommodate a broad range of substituents. Both mono-substituted (5b–5f) and di-substituted (5g) phenyl rings readily afforded the corresponding amide products in good yields. The aromatic ring bearing a fluorine atom gave a yield of 75% (5b). Additionally, naphthyl moieties, whether 2-naphthyl or 1-naphthyl, delivered the desired products in moderate yields (5h, 5i). Similarly, subsequent photochemical removal of the Ts group from the *Z*/*E* mixtures of 5*via* C–S bond cleavage provided the corresponding amides 6 with exclusive *E*-olefinic configuration in moderate to good yields (Scheme 2, bottom).

**Scheme 3:**
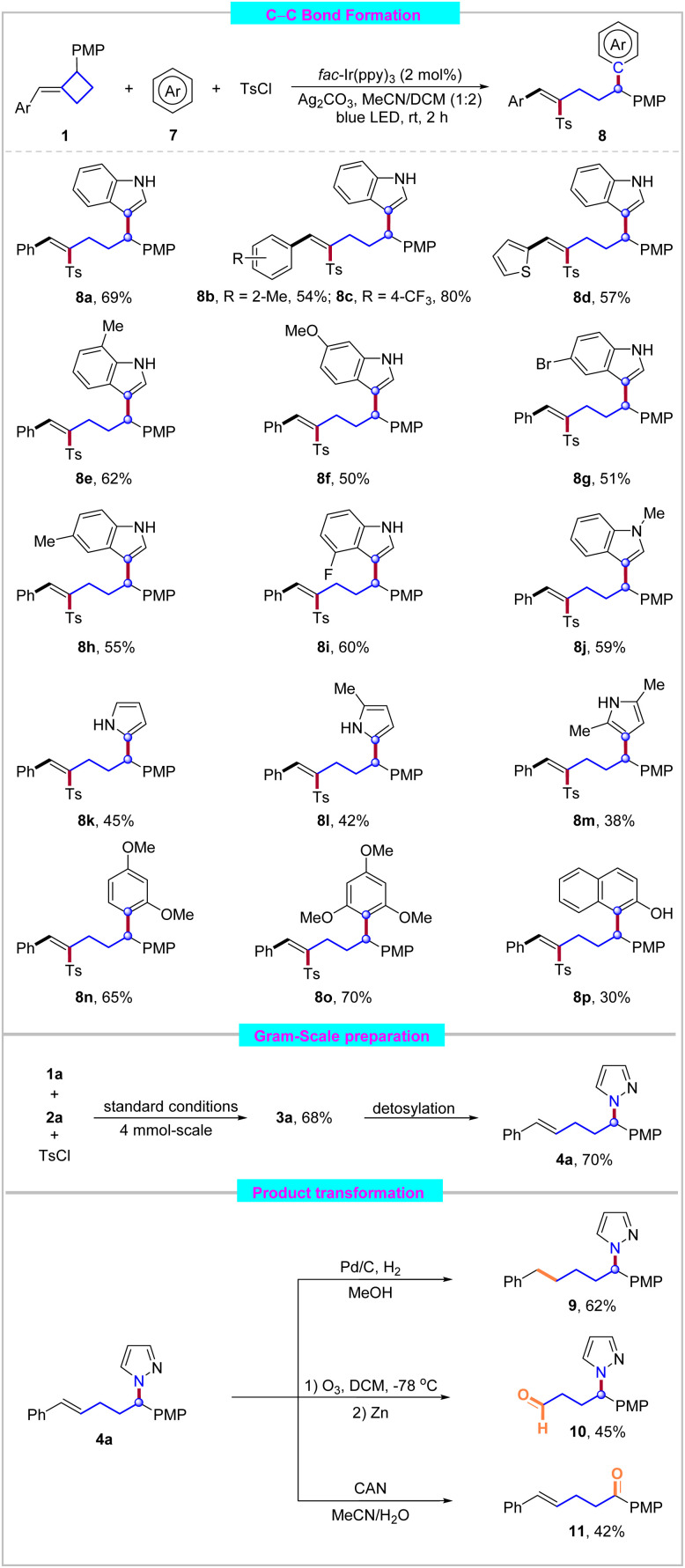
Extension to C–C bond formation, gram-scale preparation, and product transformations. Standard reaction conditions for C–C bond formation: 1 (0.2 mmol), 7 (0.8 mmol), fac-Ir(ppy)_3_ (2 mol%), TsCl (0.6 mmol), and Ag_2_CO_3_ (0.2 mmol) in a mixed solvent of MeCN/DCM (v/v = 1 : 2, 3 mL) at room temperature under N_2_ for 2 h and 30 W blue LEDs. Yields of isolated products are given.

This protocol could be further extended to remote C–C bond formation (Scheme 3, top). Indole, pyrrole, benzene, and naphthol proved to be viable nucleophiles in the reaction with methylenecyclobutane 1, and all products exhibited exclusive *E*-olefin geometry. Both unprotected indoles (8a–8i) and *N*-methyl indole (8j) underwent C–C bond formation at the C3 position with substrate 1 bearing various aryl/heteroaryl substituents, affording the products in yields ranging from 50% to 80%. Pyrrole, which is structurally like pyrazole, also enabled C–C bond formation, occurring at the C2 position while exclusively retaining the olefin geometry (8k–8m). Electron-rich phenyl rings were tolerated, successfully delivering C–C bond formation in good yields (8n and 8o). In the presence of naphthol, the C–C bond was selectively formed at the C1 position (8p).

Gram-scale preparation from 4.0 mmol of 1a afforded 3a in a high yield, followed by detosylation with comparable efficiency (Scheme 3, middle).

Product transformation was then conducted (Scheme 3, bottom). The olefinic moiety in 4a underwent Pd/C-catalyzed hydrogenation to furnish the corresponding reduced product 9.

Moreover, 4a could be converted to the corresponding aldehyde 10*via* O_3_/Zn-mediated ozonolysis.^17^ When 4a was treated with ceric ammonium nitrate (CAN) in a mixed MeCN/H_2_O solvent, oxidation-induced C–N bond cleavage occurred, providing a synthetic route to γ,δ-unsaturated ketone 11.

**Fig. 2:**
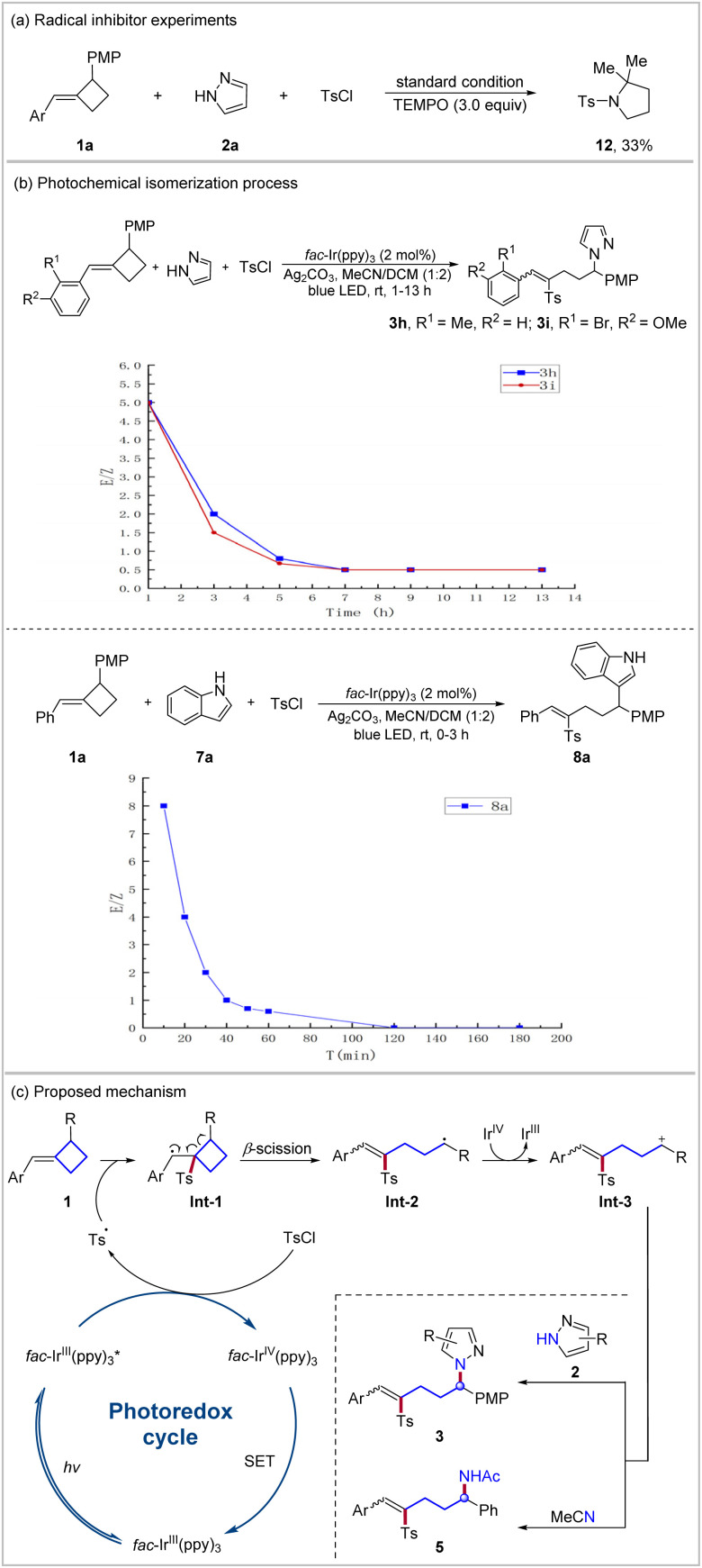
(a) Radical inhibitor experiments. (b) Photochemical isomerization process. (c) Proposed mechanism.

Subsequently, the reaction mechanism was investigated through a series of control experiments. We commenced by performing a radical trapping experiment using 2,2,6,6-tetramethyl-1-piperidinyloxy (TEMPO) under standard conditions, which led to the successful identification of the TEMPO adduct 12 (Fig. 2a).^18^ This observation indicates that the reaction proceeded *via* a radical pathway involving the generation of a Ts radical. To track time-dependent stereoselectivity shifts, we performed time-course control experiments on compounds 3h, 3i and 8a. As shown in Fig. 2b, the *Z*/*E* ratio evolved dynamically throughout the reaction and stabilized after 7 h for compound 3 and after 2 h for compound 8.

A plausible reaction mechanism is proposed (Fig. 2c). First, the photocatalyst fac-Ir^III^(ppy)_3_ is photoexcited under blue light to form the excited state 
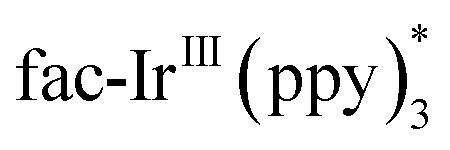
. Upon reaction with TsCl, fac-Ir^III^(ppy)_3_ induces homolytic cleavage of the S–Cl bond in TsCl *via* a single electron transfer (SET) process, generating the Ts radical while being simultaneously oxidized to fac-Ir^IV^(ppy)_3_. The Ts radical then undergoes regioselective addition to the alkenyl moiety of methylenecyclobutane 1, affording the benzyl radical intermediate Int-1. Driven by the strain of the four-membered ring, Int-1 undergoes β-scission of the C–C bond to give intermediate Int-2, which is then oxidized by fac-Ir^IV^(ppy)_3_*via* a second SET to afford the cationic intermediate Int-3 and regenerate the photocatalyst. Finally, Int-3 participates in a nucleophilic reaction with pyrazole 2 or MeCN, enabling selective formation of C–N bonds.

## Conclusion

We have developed a novel photoinduced ring-opening 1,4-difunctionalization of methylenecyclobutanes, enabling efficient construction of C–N bonds. This transformation proceeds through a cascade sequence involving sulfonyl radical addition, benzylic radical-triggered cyclobutyl C–C σ-bond cleavage, alkene regeneration, and nucleophilic capture, affording valuable bishomoallylic amine derivatives. The method features a broad substrate scope, good functional group tolerance, and exclusive *E*-olefin geometry. The incorporated sulfonyl group can be photocatalytically removed, and the remaining alkene moiety serves as a versatile handle for further derivatization. Moreover, the protocol is readily extendable to C–C bond formation using carbon nucleophiles such as indoles, pyrroles, and electron-rich arenes.

## Author contributions

YZ and CZ conceived the idea and designed the experiments. YC, YL and NW performed the laboratory experiments. YZ helped with the analysis of the data. YZ, WW and CZ prepared the manuscript. YZ and CZ supervised the project. All authors have given approval to the final version of the manuscript.

## Conflicts of interest

There are no conflicts to declare.

## Supplementary Material

SC-OLF-D6SC02950G-s001

## Data Availability

Data supporting the manuscript are provided in the supplementary information (SI). Supplementary information: experimental methods, compound characterization, and NMR spectra for this study. See DOI: https://doi.org/10.1039/d6sc02950g.
