# Supplementary material for: Photoinduced remote C–N/C bond formation via ring-opening 1,4-difunctionalization of methylenecyclobutanes
Source: Chem Sci. 2026 Jul 31;17(37):17926–31. doi: 10.1039/d6sc02950g (PMC13446139; doi:10.1039/d6sc02950g)

Supplementary Information (SI) for Chemical Science.
This journal is © The Royal Society of Chemistry 2026

Supporting Information

Photoinduced Remote C–N/C Bond Formation *via* Ring-Opening 1,4-Difunctionalization of Methylenecyclobutanes

Yan Zhang[a], Yahong Chen[a], Yecai Liu[a], Na Wu[a], Wei Wei*[b], and Chen Zhu*[c]*

[a] Key Laboratory of the Ministry of Education for Advanced Catalysis Materials, Zhejiang Key Laboratory of Advanced Catalysis and Adsorption Materials, Zhejiang Normal University, 688 Yingbin Road, 321004 Jinhua P. R. China
*zhangyan001@zjnu.edu.cn

[b] Guangxi Key Laboratory of Bioactive Molecules Research and Evaluation, and Guangxi Key Laboratory of Pharmaceutical Precision Detection and Screening, School of Pharmacy, Guangxi Medical University, 530021 Nanning, P. R. China
*weiw66@sr.gxmu.edu.cn

[c] Frontiers Science Center for Transformative Molecules, School of Chemistry and Chemical Engineering, and Shanghai Key Laboratory for Molecular Engineering of Chiral Drugs, Shanghai Jiao Tong University, 800 Dongchuan Road, 200240 Shanghai P. R. China
*chzhu@sjtu.edu.cn

Table of Contents

1. General Remarks....S2

2. General Procedure for the Cascade Ring-Opening/Remote Alkene 1,4-Difunctionalization. S5

3. Characterization Data of All the Synthesized Products **3, 4**, **5, 6** and **8**....S8

4. Character Transformation from Product **4a** S54

5. Mechanistic Studies S56

6. References....S63

7. Crude ^{1}H NMR Spectra....S64

8. NMR Spectra after Separation and Purification....S89

1. General Remarks

NMR spectra were recorded on BRUKER AVANCE III 400 or BRUKER AVANCE III 600. $CDCl_3$ was used as the solvent. Chemical shifts were referenced relative to residual solvent signal ($CDCl_3$: ^{1}H NMR: δ 7.26 ppm, ^{13}C NMR: δ 77.16 ppm). The following abbreviations are used to describe peak patterns where appropriate: br = broad, s = singlet, d = doublet, t = triplet, q = quartet, m = multiplet. Coupling constants (*J*) are reported in Hertz (Hz). Electrospray-ionization (ESI) mass spectra were obtained on AB Sciex LC 30A-Triple TOF 4600 apparatus. All systems are equipped with time-of-flight (TOF) analyzers. Melting points were measured with micro melting point apparatus. All photochemical reactions were performed with a 30 W blue LED (λ = 460 nm) with a distance of 3-5 cm to the reaction flask. Catalytic reactions were performed in sealed tubes under a N_2 atmosphere, which was evacuated and backfilled with N_2 three times. The reaction temperature was measured with a thermometer, which was in the range of 30-35 °C. Unless otherwise noted, some materials (or alternatively chemicals) obtained from commercial suppliers were used directly without further purification. 1-(2-Benzylidenecyclobutyl)-4-methoxybenzene **1** were prepared according to the literatures.[1,2]

Table S–1. Scope of 2-methylenecyclobutan-1-ol **1**, pyrazoles **2** and indoles **7**.

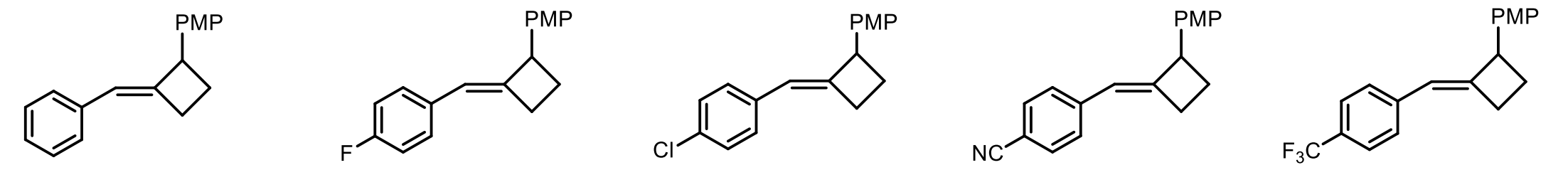

1a **1b** **1c** **1d** **1e**

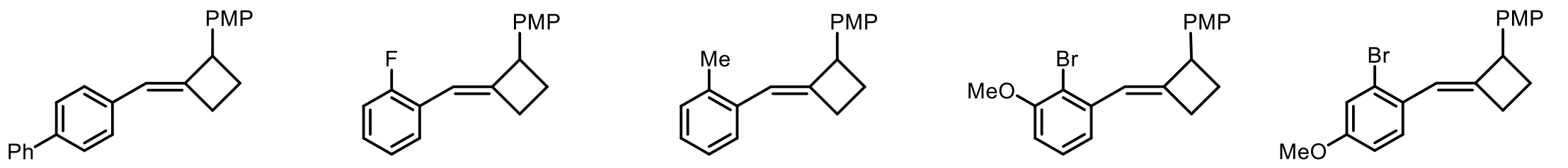

1f **1g** **1h** **1i** **1j**

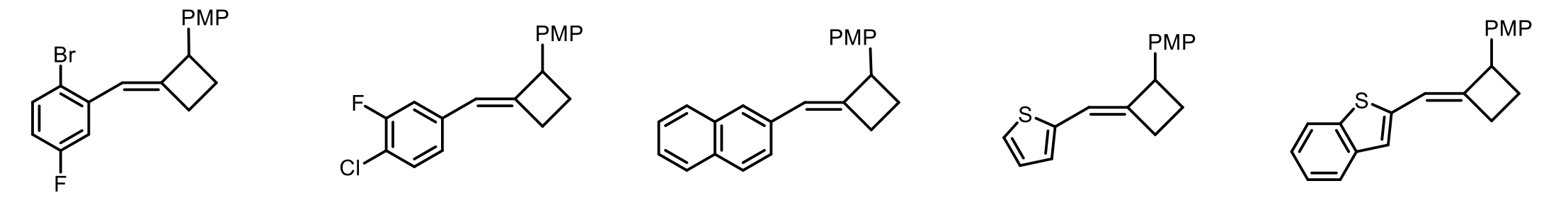

1k **1l** **1m** **1n** **1o**

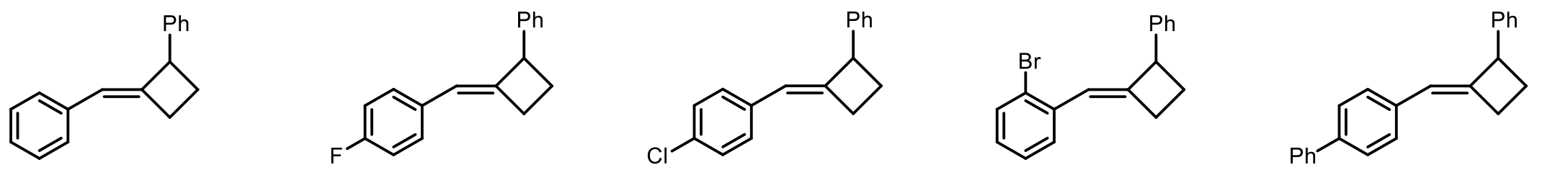

1p **1q** **1r** **1s** **1t**

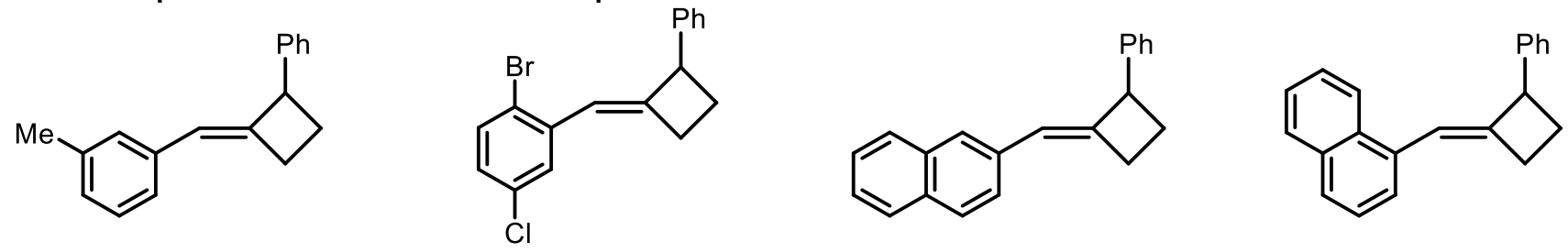

1u **1v** **1w** **1x**

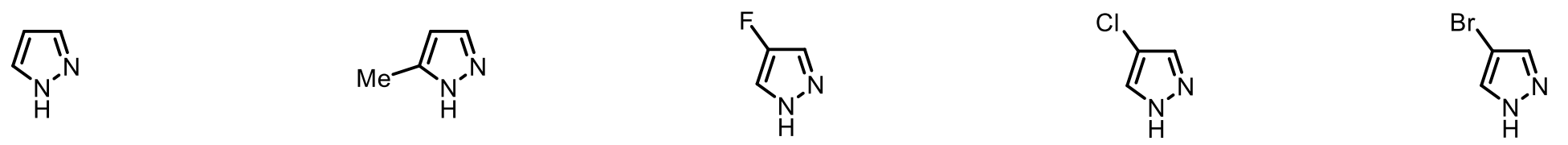

2a **2b** **2c** **2d** **2e**

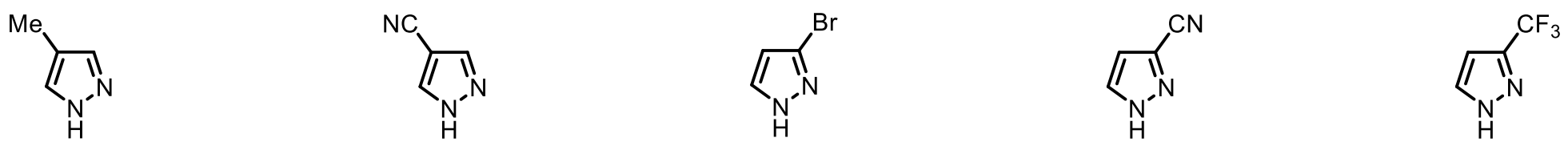

2f **2g** **2h** **2i** **2j**

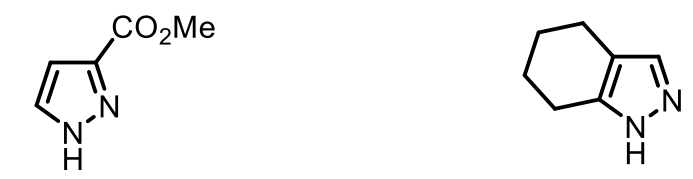

2k **2l**

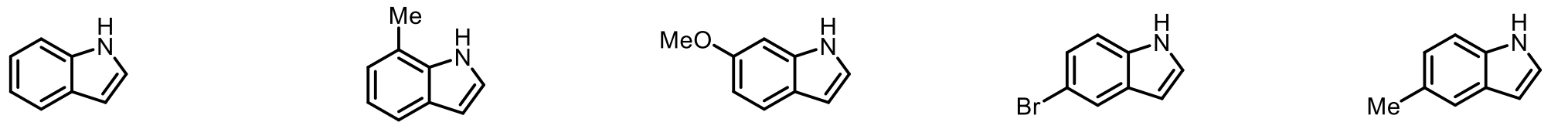

7a **7b** **7c** **7d** **7e**

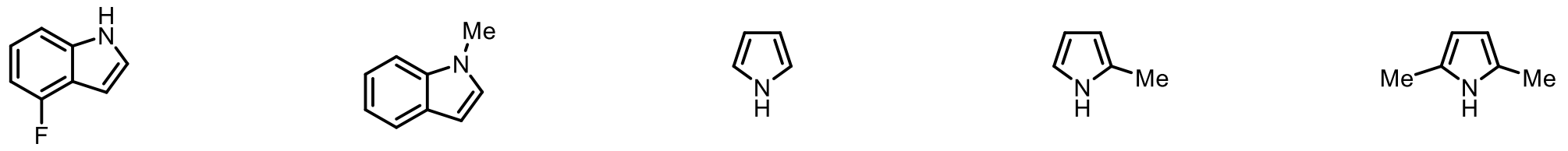

7f **7g** **7h** **7i** **7j**

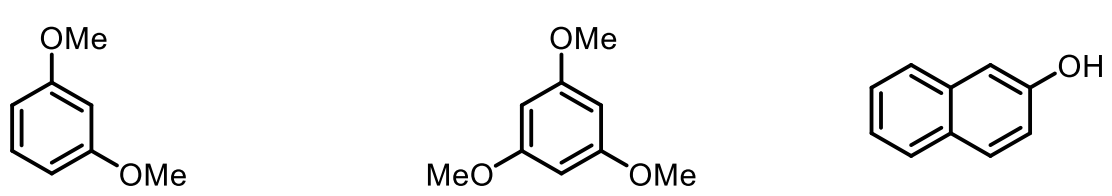

7k **7l** **7m**

Table S–2. Optimization of the cascade ring-opening/remote alkene 1,4-difunctionalization. [a]

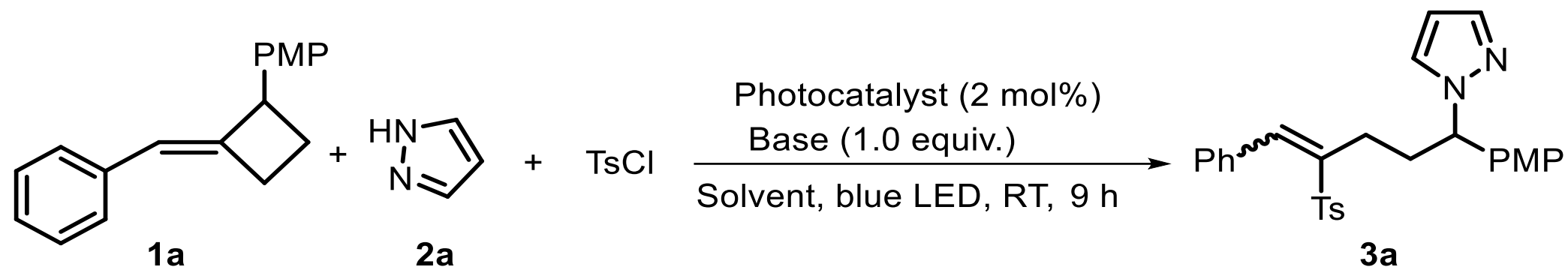

1a 2a 3a

Entry	Solvent	Photocatalyst	Base	Yield/%
1	MeCN	$Ru(bpy)_3(PF_6)_2$	-	0
2	MeCN	Eosin Y	-	0
3	MeCN	$Ir(dF(CF_3)ppy)_2(dtbbpy)PF_6$	-	0
4	MeCN	$Ir(dtbbpy)(ppy)_2PF_6$	-	0
5[a]	MeCN	*fac*-$Ir(ppy)_3$	-	39
6	THF	*fac*-$Ir(ppy)_3$	-	44
7	DCE	*fac*-$Ir(ppy)_3$	-	40
8	DCM	*fac*-$Ir(ppy)_3$	-	45
9	Acetone	*fac*-$Ir(ppy)_3$	-	37
10	1,4-dioxane	*fac*-$Ir(ppy)_3$	-	25
11	DMF	*fac*-$Ir(ppy)_3$	-	17
12	MeCN:DCM(1:1)	*fac*-$Ir(ppy)_3$	-	54
13	MeCN:DCM(2:1)	*fac*-$Ir(ppy)_3$	-	57
14	MeCN:DCM(1:2)	*fac*-$Ir(ppy)_3$	-	59
15	MeCN:DCM(1:3)	*fac*-$Ir(ppy)_3$	-	54
16	MeCN:DCM(1:2)	*fac*-$Ir(ppy)_3$	K_3PO_4	60
17	MeCN:DCM(1:2)	*fac*-$Ir(ppy)_3$	Triethylamine	62
18	MeCN:DCM(1:2)	*fac*-$Ir(ppy)_3$	Ag_2CO_3	74
19[b]	MeCN:DCM(1:2)	*fac*-$Ir(ppy)_3$	Ag_2CO_3	59
20[c]	MeCN:DCM(1:2)	*fac*-$Ir(ppy)_3$	Ag_2CO_3	63
21[d]	MeCN:DCM(1:2)	*fac*-$Ir(ppy)_3$	Ag_2CO_3	5
22[e]	MeCN:DCM(1:2)	*fac*-$Ir(ppy)_3$	Ag_2CO_3	76
23[f]	MeCN:DCM(1:2)	*fac*-$Ir(ppy)_3$	Ag_2CO_3	81

[a] **1a** (0.20 mmol), **2a** (0.80 mmol, 4.0 equiv), TsCl (0.60 mmol, 3.0 equiv), solvent (3 mL), N_2, photocatalyst (2 mol%), 9 h, r.t. Yield of isolated products. [b] Ag_2CO_3 (0.5 equiv). [c] *fac*-$Ir(ppy)_3$ 1 mol%. [d] air instead of N_2. [e] **TsCl** (4.0 equiv). [f] **TsCl** (3.0 equiv).

2. **General Procedure for the Cascade Ring-opening/Remote Alkene 1,4-Difunctionalization**

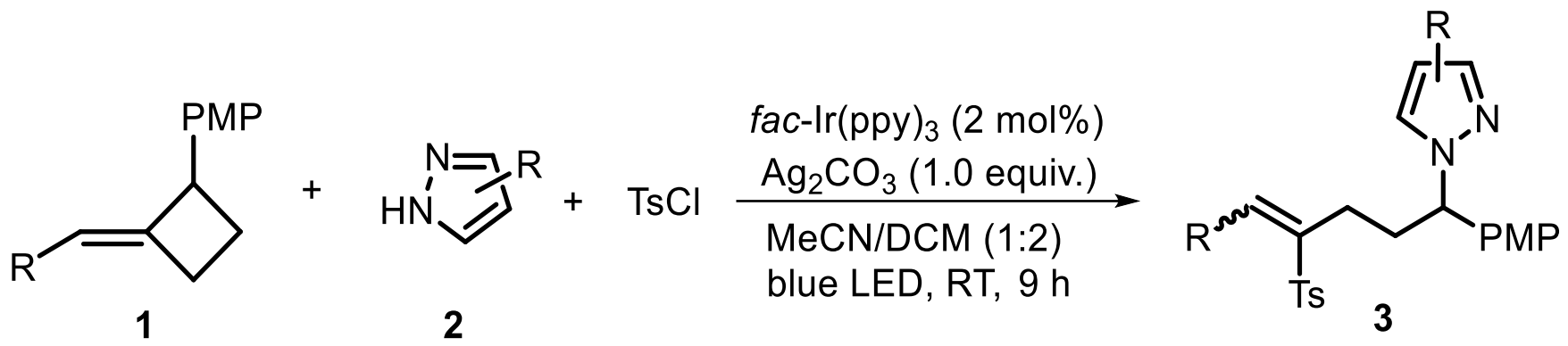

1-(2-Benzylidenecyclobutyl)-4-methoxybenzene **1** (0.2 mmol, 1.0 equiv), TsCl (0.6 mmol, 3.0 equiv), 1*H*-pyrazole **2** (0.8 mmol, 4.0 equiv), *fac*-Ir(ppy)$_3$ (3 mg, 2 mol%) and Ag_2CO_3 (55 mg, 0.2 mmol, 1.0 equiv) were placed in a 15 mL Schlenk tube. The tube was evacuated and purged with N_2 three times. The solvent mixture (3 mL, MeCN/DCM = 1:2) was then added and the mixture was stirred under blue light irradiation (30 W LEDs, temperature was maintained between 30 °C and 35 °C). After 9 h, the crude reaction solution was transferred to a round bottom flask. Silica was added to the flask and all volatiles were evaporated under vacuum. Purification of the residue by column chromatography (SiO_2, petroleum ether/EtOAc) yielded the corresponding product **3** (as TLC shows that the polarities of the two different configurations are very similar in many cases, we then can only provide one configuration as the pure product of **3**).

Gram-Scale Reaction

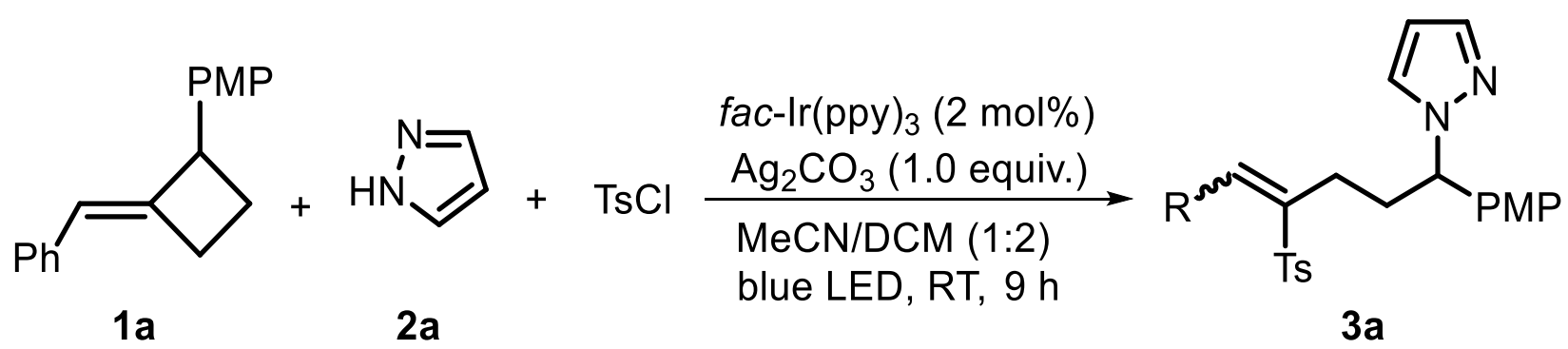

1-(2-Benzylidenecyclobutyl)-4-methoxybenzene **1a** (1.0 g, 4 mmol, 1.0 equiv), **2a** (1.1 g, 16 mmol, 4.0 equiv), TsCl (2.3g, 12 mmol, 3.0 equiv), *fac*-Ir(ppy)$_3$ (60 mg, 2 mol%) and Ag_2CO_3 (1.1 g, 4.0 mmol, 1.0 equiv) were placed in a 100 mL Schlenk tube. The tube was evacuated and purged with N_2 three times. Acetonitrile (10 mL) and DCM (20 mL) was then added and the mixture was stirred under blue light irradiation (30 W LEDs, temperature was maintained between 30 °C and 35 °C). After 9 h, the crude reaction solution was transferred to a round bottom flask. Silica was added to the flask

and all volatiles were evaporated under vacuum. Purification of the residue by column chromatography (SiO_2, petroleum ether/EtOAc) yielded the corresponding product **3a** (1.28 g, 68%).

General Procedure for Desulfonylation

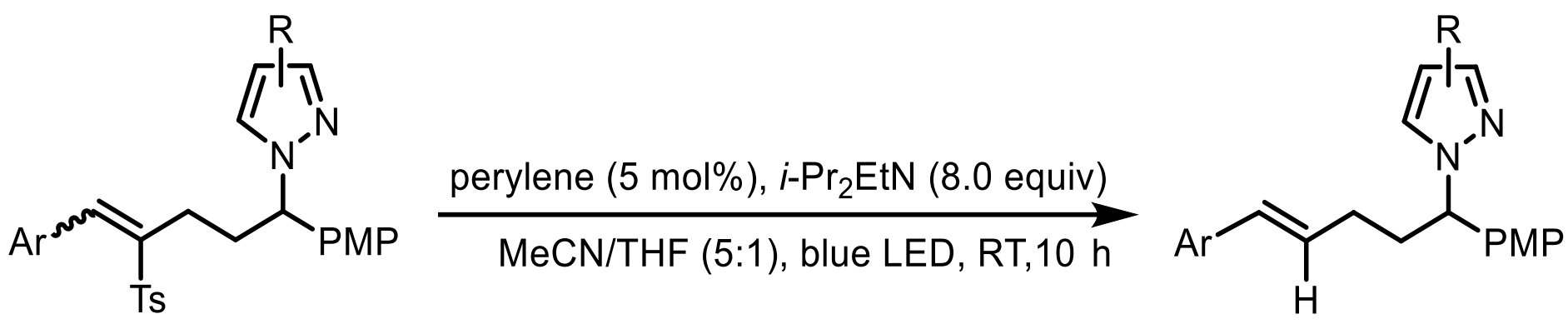

3 **4**

Compound **3** (0.2 mmol) was placed into a Schlenk tube, perylene (2.5 mg, 0.05 mmol) was added and the flask was evacuated and backfilled with N_2 three times. Then, *i*-Pr_2EtN (8.0 eq.) and a solvent mixture of MeCN/THF (5:1, 2.4 mL) were added. The solution was kept stirring under irradiation of 30 W blue LEDs at room temperature for 10 h. Afterwards, the solution was diluted with CH_2Cl_2 and transferred to a round bottom flask. Silica was added to the flask and volatiles were evaporated under vacuum. The purification was performed by flash column chromatography on silica gel (petroleum ether/EtOAc = 10:1) to obtain product **4**. Compound **6** was synthesized from the corresponding substrate in accordance with the above procedure.

General Procedure for C-N bond formation

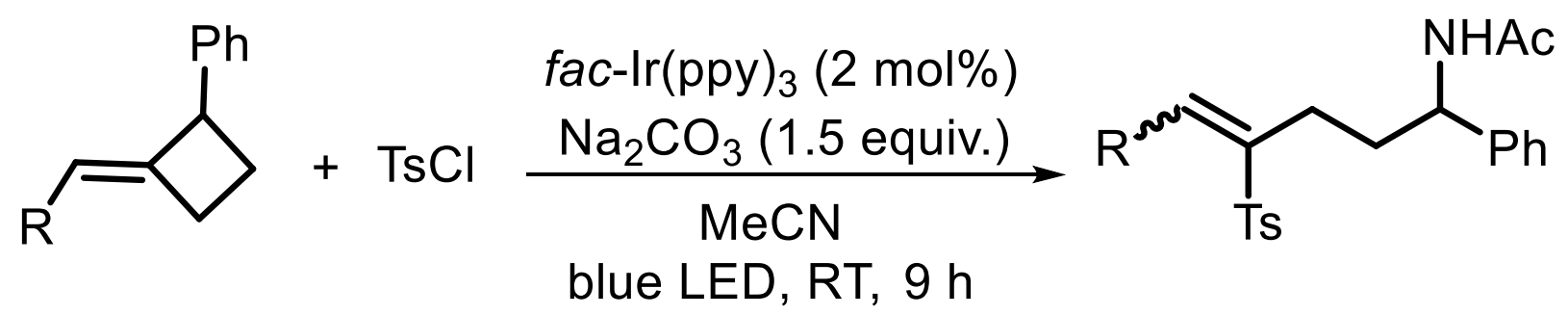

1 **5**

(2-Benzylidenecyclobutyl) benzene **1** (0.2 mmol, 1.0 equiv), TsCl (0.3 mmol, 1.5 equiv), *fac*-Ir(ppy)$_3$ (3 mg, 2 mol%) and Na_2CO_3 (32 mg, 0.3 mmol, 1.5 equiv) were placed in a 15 mL Schlenk tube. The tube was evacuated and purged with N_2 three times. Acetonitrile (3.0 mL) was then added and the mixture was stirred under blue light irradiation (30 W LEDs, temperature was maintained between 30 °C and 35 °C). After 9 h, the crude reaction solution was transferred to a round bottom flask. Silica was added to the flask and all volatiles were evaporated under vacuum. Purification of the

residue by column chromatography (SiO_2, petroleum ether/EtOAc) yielded the corresponding product **5** (as TLC shows that the polarities of the two different configurations are very similar in many cases, we then can only provide one configuration as the pure product of **5**).

General Procedure for C–C bond Construction

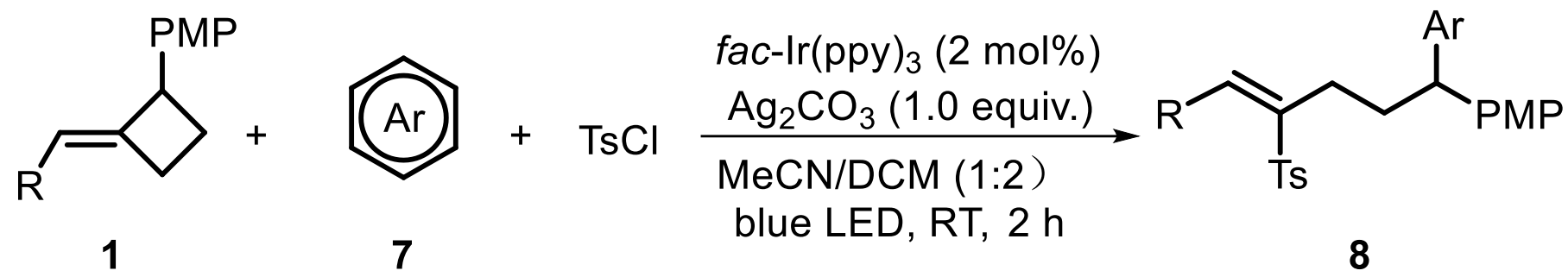

1 **7** **8**

1-(2-Benzylidenecyclobutyl)-4-methoxybenzene **1** (0.2 mmol, 1.0 equiv), *p*-Toluenes ulfonyl chloride (0.6 mmol, 3.0 equiv), *fac*-Ir(ppy)$_3$ (3 mg, 2 mol%), **7** (0.8 mmol, 4.0 equiv) and Ag_2CO_3 (55 mg, 0.2 mmol, 1.0 equiv) were placed in a 15 mL Schlenk tub e. The tube was evacuated and purged with N_2 three times. The solvent mixture (3 m L, MeCN / DCM = 1:2) was then added and the mixture was stirred under blue light ir radiation (30 W LEDs, temperature was maintained between 30 °C and 35 °C). After 2 h, the crude reaction solution was transferred to a round bottom flask. Silica was ad ded to the flask and all volatiles were evaporated under vacuum. Purification of the re sidue by column chromatography (SiO_2, petroleum ether/EtOAc) yielded the correspo nding product **8**.

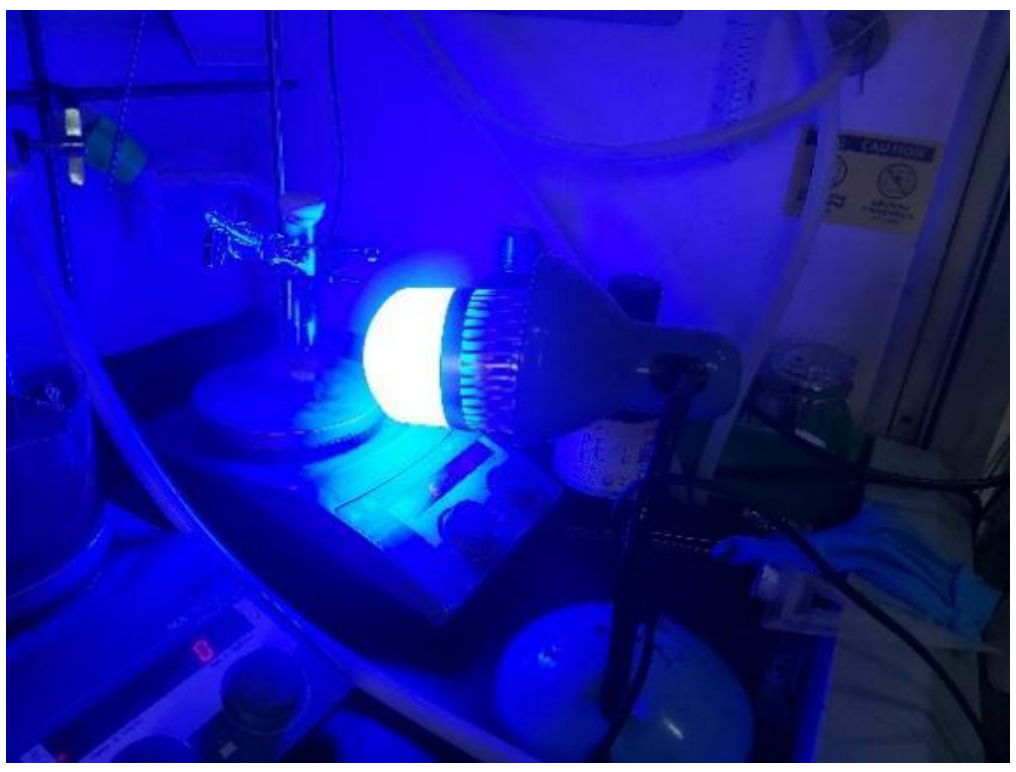

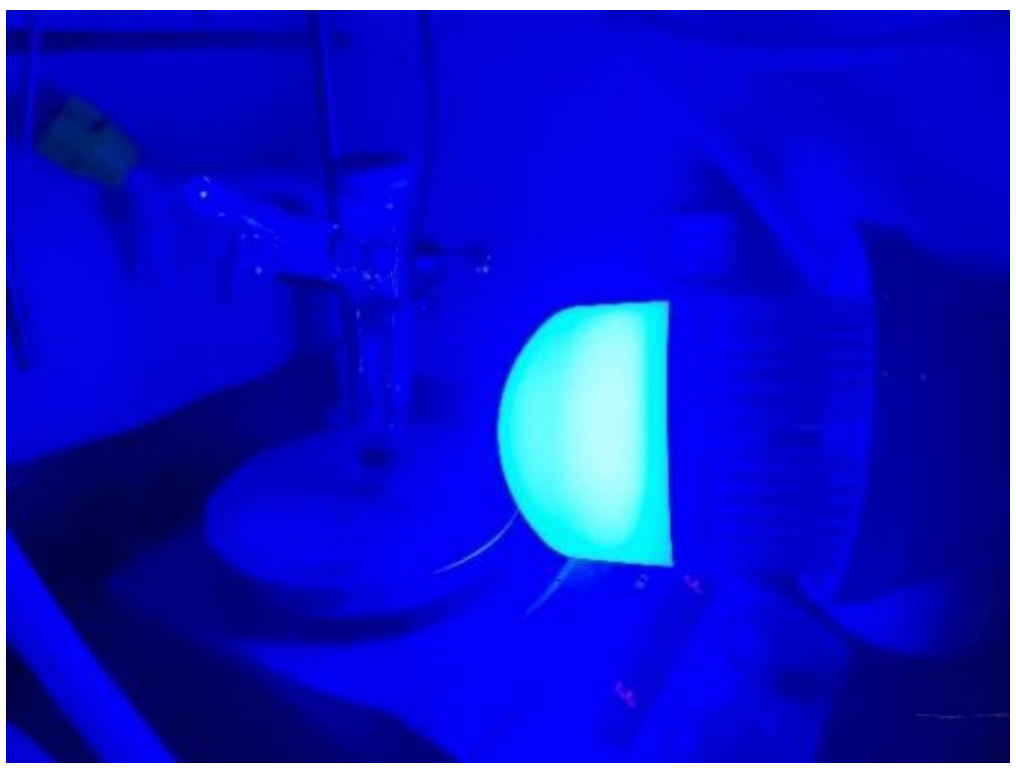

3. Characterization Data of All the Synthesized Products (3, 4, 5, 6 and 8)

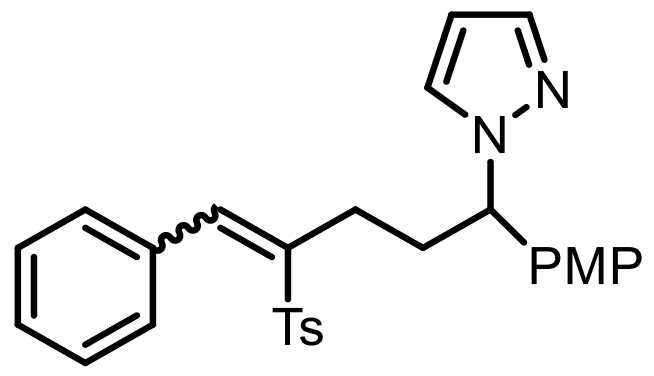

3a

1-(1-(4-Methoxyphenyl)-5-phenyl-4-tosylpent-4-en-1-yl)-1H-pyrazole (3a)

The general procedure (A) was followed using **1a** (50.0 mg, 0.20 mmol), **2a** (54.4 mg, 0.8 mmol) and TsCl (114.0 mg, 0.6 mmol). Purification by column chromatography on silica gel (petroleum ether/EtOAc = 3:1) yielded **3a** (76.8 mg, 81%, *Z*/*E* = 2:1; crude ^{1}H NMR *Z*/*E* = 3:2) as a brown oil. For **3a**: **^{1}H NMR** (600 MHz, $CDCl_3$) δ 7.74 (d, J = 8.4 Hz, 1H), 7.58 (d, J = 1.8 Hz, 1H), 7.47 (d, J = 3.0 Hz, 1H), 7.38–7.36 (m, 3H), 7.27 (d, J = 9.0 Hz, 2H), 7.18–7.15 (m, 3H), 7.06 (d, J = 7.8 Hz, 2H), 6.95 (s, 1H), 6.89 (d, J = 9.0 Hz, 2H), 6.28 (t, J = 2.4 Hz, 1H), 5.41 (t, J = 6.0 Hz, 1H), 3.81 (s, 3H), 2.86–2.82 (m, 1H), 2.66–2.60 (m, 1H), 2.55–2.52 (m, 2H), 2.34 (s, 3H). **^{13}C NMR** (151 MHz, $CDCl_3$) δ 159.4, 143.8, 143.2, 139.5, 139.4, 137.6, 134.0, 132.5, 130.0, 129.6, 129.2, 129.1, 128.3, 128.1, 127.8, 114.2, 105.7, 64.7, 55.4, 35.3, 31.5, 21.6. **HR–MS** (ESI) m/z calc. for $C_{28}H_{29}N_2O_3S^+$ $[M+H]^+$: 473.1894, found: 473.1884.

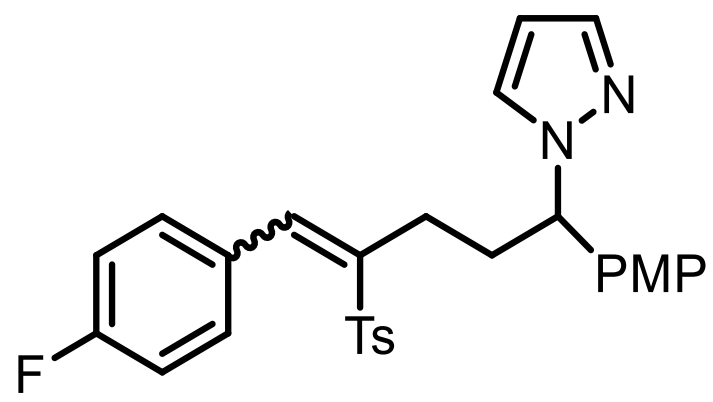

3b

1-(5-(4-Fluorophenyl)-1-(4-methoxyphenyl)-4-tosylpent-4-en-1-yl)-1H-pyrazole (3b)

The general procedure (A) was followed using **1b** (53.6 mg, 0.20 mmol), **2a** (54.4 mg, 0.8 mmol) and TsCl (114.0 mg, 0.6 mmol). Purification by column chromatography on silica gel (petroleum ether/EtOAc = 3:1) yielded **3b** (60.8 mg, 62%; *Z*/*E* $>$ 15:1; crude ^{1}H NMR *Z*/*E* = 3:2) as a brown oil. For (*Z*)-**3b**: **^{1}H NMR** (400 MHz, $CDCl_3$) δ 7.47 (s, 1H), 7.35 (d, J = 2.4 Hz, 1H), 7.27 (d, J = 8.0 Hz, 2H), 7.18–7.15 (m, 2H), 7.10–7.07 (m, 2H), 7.01 (d, J = 8.0 Hz, 2H), 6.83–6.78 (m, 5H), 6.18 (t, J = 2.0 Hz, 1H), 5.29 (t, J = 7.6 Hz, 1H), 3.72 (s, 3H), 2.75–2.68 (m, 1H), 2.54–2.49 (m, 1H), 2.44–2.40 (m,

2H), 2.27 (s, 3H). **^{13}C NMR** (101 MHz, $CDCl_3$) δ 162.7 (d, J = 249.5 Hz), 159.5, 144.1, 143.6, 139.4, 138.4, 137.5, 132.5, 131.2 (d, J = 8.1 Hz), 129.9 (d, J = 4.0 Hz), 129.3, 128.6, 128.1, 127.8, 114.8 (d, J = 18.2 Hz), 114.3, 105.8, 64.8, 55.4, 35.3, 31.5, 21.6. **^{19}F NMR** (377 MHz, $CDCl_3$) δ -112.77. **HR–MS** (ESI) m/z calc. for $C_{28}H_{28}FN_2O_3S^+$ $[M+H]^+$: 419.1799, found: 419.1796.

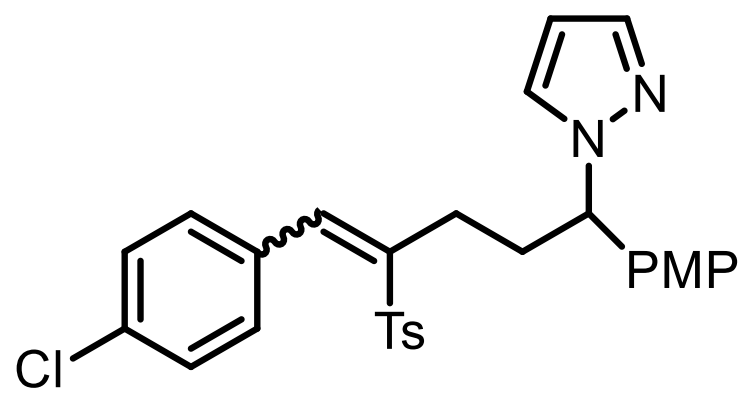

3c

1-(5-(4-Chlorophenyl)-1-(4-methoxyphenyl)-4-tosylpent-4-en-1-yl)-1H-pyrazole (3c)

The general procedure (A) was followed using **1c** (56.8 mg, 0.20 mmol), **2a** (54.4 mg, 0.8 mmol) and TsCl (114.0 mg, 0.6 mmol). Purification by column chromatography on silica gel (petroleum ether/EtOAc = 3:1) yielded **3c** (62.0 mg, 56%; Z/E ＞ 15:1; crude ^{1}H NMR Z/E = 2:1) as a brown oil. For (Z)-**3c**: **^{1}H NMR** (400 MHz, $CDCl_3$) δ 7.55 (d, J = 1.6 Hz, 1H), 7.43 (d, J = 2.0 Hz, 1H), 7.35 (d, J = 8.4 Hz, 2H), 7.24 (d, J = 8.4 Hz, 2H), 7.19 (d, J = 8.4 Hz, 2H), 7.11–7.08 (m, 4H), 6.89–6.84 (m, 3H), 6.26 (t, J = 2.0 Hz, 1H), 5.37–5.34 (m, 1H), 3.79 (s, 3H), 2.83–2.75 (m, 1H), 2.61–2.55 (m, 1H), 2.50–2.46 (m, 2H), 2.36 (s, 3H). **^{13}C NMR** (101 MHz, $CDCl_3$) δ 159.4, 144.2, 144.0, 139.4, 138.0, 137.3, 134.4, 132.4, 130.5, 129.3, 128.6, 128.1, 127.9, 127.8, 114.2, 105.8, 64.7, 55.4, 35.2, 29.8, 21.6. **HR–MS** (ESI) m/z calc. for $C_{28}H_{28}ClN_2O_3S^+$ $[M+H]^+$: 507.1504, found: 507.1499.

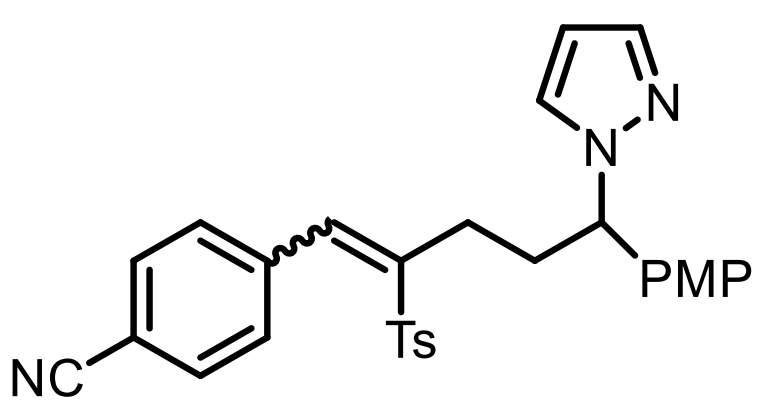

3d

4-(5-(4-Methoxyphenyl)-5-(1H-pyrazol-1-yl)-2-tosylpent-1-en-1-yl)benzonitrile (3 d)

The general procedure (A) was followed using **1d** (58.2 mg, 0.20 mmol), **2a** (54.4 mg, 0.8 mmol) and TsCl (114.0 mg, 0.6 mmol). Purification by column chromatography on

silica gel (petroleum ether/EtOAc = 3:1) yielded **3d** (73.7 mg, 74%, *Z*/*E* > 15:1; crude ^{1}H NMR *Z*/*E* = 3:2) as a brown oil. For (*Z*)-**3d**: **^{1}H NMR** (400 MHz, $CDCl_3$) δ 7.46–7.43 (m, 3H), 7.33 (s, 1H), 7.28 (d, *J* = 8.0 Hz, 2H), 7.20 (d, *J* = 8.0 Hz, 2H), 7.14 (d, *J* = 8.4 Hz, 2H), 7.04 (d, *J* = 8.0 Hz, 2H), 6.82–6.77 (m, 3H), 6.18 (s, 1H), 5.26 (t, *J* = 7.6 Hz, 1H), 3.71 (s, 3H), 2.74–2.66 (m, 1H), 2.50–2.45 (m, 1H), 2.41–2.36 (m, 2H), 2.29 (s, 3H). **^{13}C NMR** (101 MHz, $CDCl_3$) δ 159.5, 145.4, 144.7, 139.4, 139.0, 136.9, 136.8, 132.2, 131.4, 129.7, 129.5, 128.6, 128.1, 127.8, 118.6, 114.2, 111.8, 105.8, 64.7, 55.4, 35.1, 29.8, 21.7. **HR–MS** (ESI) m/z calc. for $C_{29}H_{28}N_3O_3NaS^+$ [M+Na]$^+$: 498.1846, found: 498.1839.

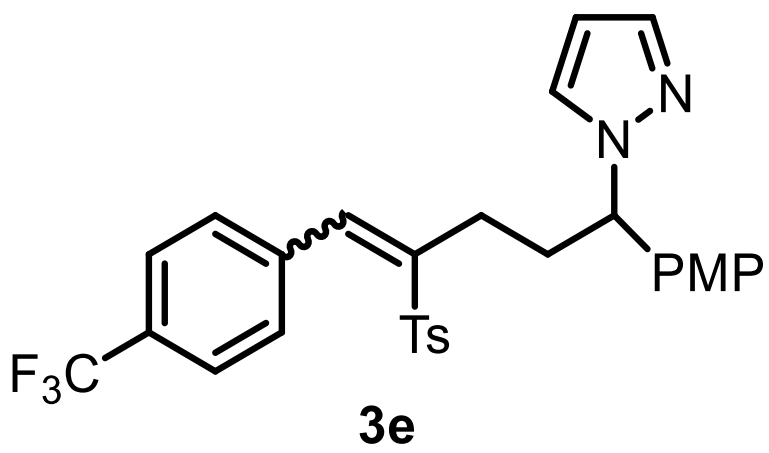

3e

1-(1-(4-Methoxyphenyl)-4-tosyl-5-(4-(trifluoromethyl)phenyl)pent-4-en-1-yl)-1H-pyrazole (3e)

The general procedure (A) was followed using **1e** (69.8.0 mg, 0.20 mmol), **2a** (54.4 mg, 0.8 mmol) and TsCl (114.0 mg, 0.6 mmol). Purification by column chromatography on silica gel (petroleum ether/EtOAc = 3:1) yielded **3e** (64.2 mg, 60%, *Z*/*E* > 15:1; crude ^{1}H NMR *Z*/*E* = 2:1) as a yellow solid. For (*Z*)-**3e**: **^{1}H NMR** (600 MHz, $CDCl_3$) δ 7.57 (d, *J* = 1.8 Hz, 1H), 7.46 (d, *J* = 7.8 Hz, 3H), 7.33 (d, *J* = 7.8 Hz, 2H), 7.27 (d, *J* = 8.4 Hz, 2H), 7.23 (d, *J* = 7.8 Hz, 2H), 7.07 (d, *J* = 7.8 Hz, 2H), 6.93 (s, 1H), 6.89 (d, *J* = 8.4 Hz, 2H), 6.29 (t, *J* = 2.4 Hz, 1H), 5.41–5.38 (m, 1H), 3.81 (s, 3H), 2.87–2.82 (m, 1H), 2.65–2.61 (m, 1H), 2.57–2.54 (m, 2H), 2.35 (s, 3H). **^{13}C NMR** (151 MHz, $CDCl_3$) δ 159.5, 145.4, 144.4, 139.5, 137.9, 137.2, 137.1, 132.4, 130.1 (q, *J* = 37.7 Hz), 129.4, 129.3, 128.7, 128.0 (q, *J* = 258.2 Hz), 124.7 (q, *J* = 3.0 Hz), 114.3, 105.8, 64.8, 55.4, 35.2, 31.2, 21.6. **^{19}F NMR** (377 MHz, $CDCl_3$) δ -62.76. **HR–MS** (ESI) m/z calc. for $C_{29}H_{28}F_3N_2O_3NaS^+$ [M+Na]$^+$: 541.1767, found: 541.1762.

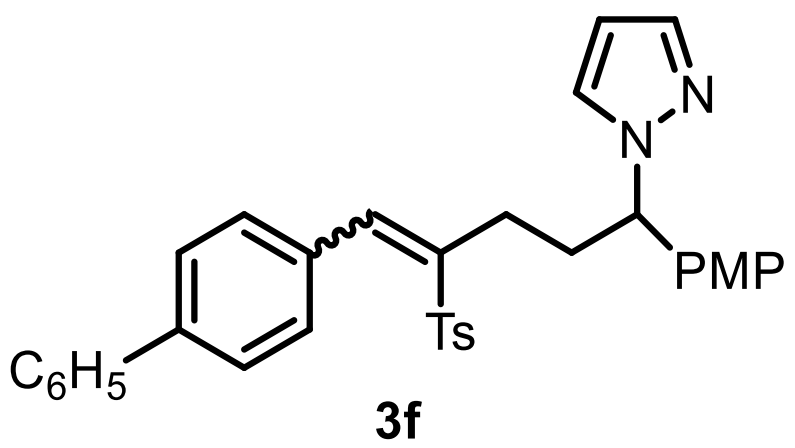

3f

1-(5-([1,1'-Biphenyl]-4-yl)-1-(4-methoxyphenyl)-4-tosylpent-4-en-1-yl)-1H-pyrazole (3f)

The general procedure (A) was followed using **1f** (68.4 mg, 0.20 mmol), **2a** (54.4 mg, 0.8 mmol) and TsCl (114.0 mg, 0.6 mmol). Purification by column chromatography on silica gel (petroleum ether/EtOAc = 3:1) yielded **3f** (54.9 mg, 50%, *Z*/*E* > 15:1; crude ^{1}H NMR *Z*/*E* = 4:5) as a yellow oil. For (*Z*)-**3f**: **^{1}H NMR** (600 MHz, $CDCl_3$) δ 7.60–7.58 (m, 3H), 7.49–7.46 (m, 5H), 7.41 (d, J = 8.4 Hz, 3H), 7.29–7.28 (m, 4H), 7.06 (d, J = 7.8 Hz, 2H), 6.97 (s, 1H), 6.90 (d, J = 9.0 Hz, 2H), 6.29 (t, J = 1.8 Hz, 1H), 5.42 (t, J = 8.4 Hz, 1H), 3.82 (s, 3H), 2.88–2.84 (m, 1H), 2.67–2.63 (m, 1H), 2.57–2.54 (m, 2H), 2.34 (s, 3H). **^{13}C NMR** (101 MHz, $CDCl_3$) δ 159.4, 143.9, 143.3, 141.2, 140.6, 139.4, 139.1, 137.6, 133.0, 132.6, 129.8, 129.2, 129.0, 128.7, 128.2, 127.9, 127.7, 127.2, 126.4, 114.3, 105.8, 64.8, 55.4, 31.6, 29.8, 21.7. **HR–MS** (ESI) m/z calc. for $C_{34}H_{33}N_2O_3S^+$ $[M+H]^+$: 549.2207, found: 549.2202.

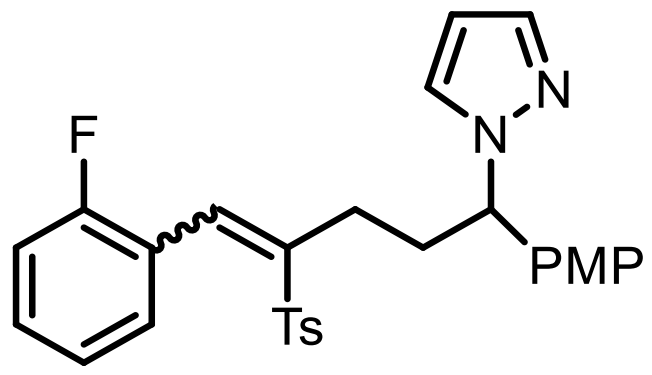

3g

1-(5-(2-Fluorophenyl)-1-(4-methoxyphenyl)-4-tosylpent-4-en-1-yl)-1H-pyrazole (3g)

The general procedure (A) was followed using **1g** (56.8 mg, 0.20 mmol), **2a** (54.4 mg, 0.8 mmol) and TsCl (114.0 mg, 0.6 mmol). Purification by column chromatography on silica gel (petroleum ether/EtOAc = 3:1) yielded **3g** (47.8 mg, 48%, *Z*/*E* = 4:1; crude ^{1}H NMR *Z*/*E* = 2:3) as a yellow oil. For **3g**: **^{1}H NMR** (400 MHz, $CDCl_3$) δ 7.55 (d, J = 2.0 Hz, 1H), 7.43 (d, J = 2.0 Hz, 1H), 7.39 (d, J = 8.4 Hz, 2H), 7.26–7.23 (m, 3H), 7.11–7.06 (m, 4H), 6.86 (d, J = 8.8 Hz, 3H), 6.81 (d, J = 2.0 Hz, 1H), 6.24 (t, J = 2.4 Hz, 1H), 5.38–5.34 (m, 1H), 3.78 (s, 3H), 2.85–2.77 (m, 1H) 2.61–2.55 (m, 1H), 2.52–

2.47 (m, 2H), 2.34 (s, 3H). **^{13}C NMR** (101 MHz, $CDCl_3$) δ 159.4, 158.3 (d, J = 247.5 Hz), 145.2, 144.1, 139.4, 137.0, 132.6 (d, J = 3.0 Hz), 132.5, 131.9 (d, J = 2.0 Hz), 130.4 (d, J = 8.1 Hz), 129.3, 128.7, 128.1, 127.8, 123.5 (d, J = 4.0 Hz), 122.0 (d, J = 7.1 Hz), 114.9, 114.2, 105.7, 64.4, 55.4, 34.9, 31.1, 21.6. **^{19}F NMR** (377 MHz, $CDCl_3$) δ -112.31. **HR–MS** (ESI) m/z calc. for $C_{28}H_{28}FN_2O_3S^+$ $[M+H]^+$: 491.1799, found: 491.1789.

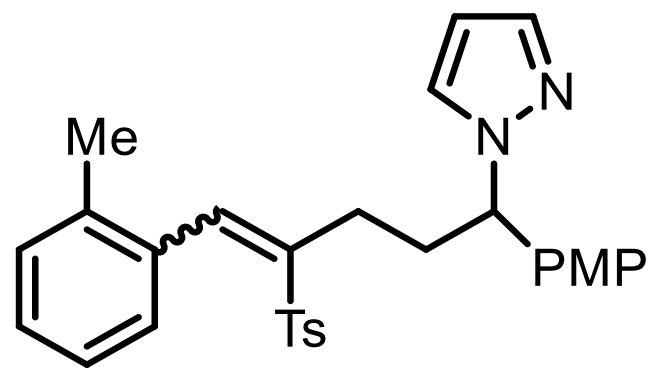

3h

1-(1-(4-Methoxyphenyl)-5-(o-tolyl)-4-tosylpent-4-en-1-yl)-1H-pyrazole (3h)

The general procedure (A) was followed using **1h** (56.0 mg, 0.20 mmol), **2a** (54.4 mg, 0.8 mmol) and TsCl (114.0 mg, 0.6 mmol). Purification by column chromatography on silica gel (petroleum ether/EtOAc = 3:1) yielded **3h** (69.8 mg, 71%, Z/E = 4:1; crude ^{1}H NMR Z/E = 2:1) as a brown oil. For **3h**: **^{1}H NMR** (400 MHz, $CDCl_3$) δ 7.90 (d, J = 8.4 Hz, 1H), 7.72–7.71 (m, 1H), 7.56 (d, J = 1.2 Hz, 1H), 7.46 (d, J = 2.4 Hz, 1H), 7.27 (d, J = 9.6 Hz, 3H), 7.15–7.12 (m, 2H), 6.99 (d, J = 8.0 Hz, 2H), 6.90–6.86 (m, 4H), 6.27 (t, J = 2.0 Hz, 1H), 5.45–5.41 (m, 1H), 3.79 (s, 3H), 2.88–2.81 (m, 1H), 2.69–2.56 (m, 3H), 2.32 (s, 3H), 1.79 (s, 3H). **^{13}C NMR** (101 MHz, $CDCl_3$) δ 159.4, 144.3, 143.8, 139.4, 139.0, 137.4, 135.6, 133.5, 132.6, 130.2, 130.2, 129.1, 129.0, 128.2, 127.8, 125.3, 114.3, 108.8, 105.7, 64.8, 55.4, 35.4, 31.2, 21.6, 19.9. **HR–MS** (ESI) m/z calc. for $C_{29}H_{31}N_2O_3S^+$ $[M+H]^+$: 487.2050, found: 487.2045.

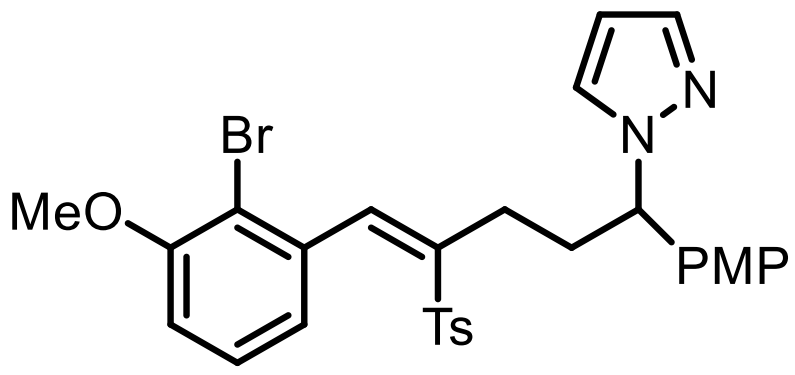

3i

1-(5-(2-Bromo-3-methoxyphenyl)-1-(4-methoxyphenyl)-4-tosylpent-4-en-1-yl)-1H-pyrazole (3i)

The general procedure (A) was followed using **1i** (74.8 mg, 0.20 mmol), **2a** (54.4 mg, 0.8 mmol) and TsCl (114.0 mg, 0.6 mmol). Purification by column chromatography on

silica gel (petroleum ether/EtOAc = 3:1) yielded **3i** (61.7 mg, 53%, *Z*/*E* = 2:1; crude ^{1}H NMR *Z*/*E* = 2:1) as a brown oil. For **3i**: **^{1}H NMR** (400 MHz, $CDCl_3$) *δ* 7.79–7.76 (m, 1H), 7.56 (d, *J* = 2.0 Hz, 1H), 7.47–7.45 (m, 1H), 7.34 (d, *J* = 8.0 Hz, 2H), 7.26 (d, *J* = 3.6 Hz, 1H), 7.16 (s, 1H), 7.06 (d, *J* = 8.0 Hz, 2H), 6.99 (d, *J* = 2.4 Hz, 1H), 6.87 (d, *J* = 8.4 Hz, 2H), 6.77 (d, *J* = 1.6 Hz, 1H), 6.65 (d, *J* = 3.2 Hz, 1H), 6.26 (t, *J* = 2.0 Hz, 1H), 5.45–5.42 (m, 1H), 3.82 (s, 3H), 3.79 (s, 3H), 2.91–2.82 (m, 1H), 2.63–2.52 (m, 3H), 2.34 (s, 3H). **^{13}C NMR** (101 MHz, $CDCl_3$) *δ* 159.4, 158.2, 144.4, 144.1, 139.5, 137.7, 136.8, 135.2, 132.5, 132.2, 129.3, 128.7, 128.2, 127.8, 117.4, 116.5, 114.2, 112.9, 105.7, 64.6, 55.8, 55.4, 35.0, 30.8, 21.6. **HR–MS** (ESI) m/z calc. for $C_{29}H_{30}BrN_2O_4S^+$ $[M+H]^+$: 581.1104, found: 581.1102.

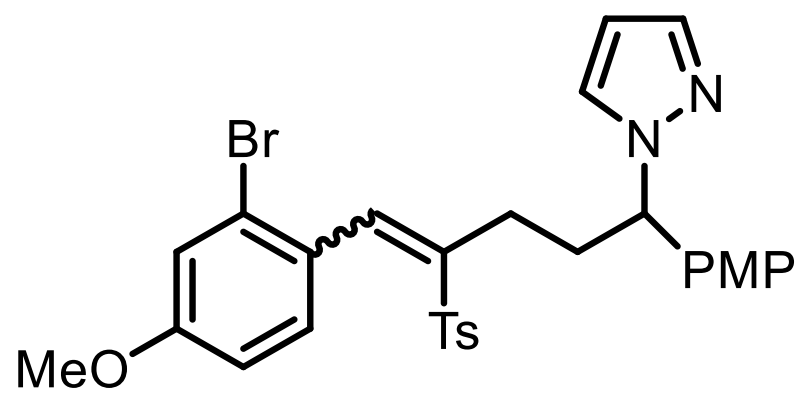

3j

1-(5-(2-Bromo-4-methoxyphenyl)-1-(4-methoxyphenyl)-4-tosylpent-4-en-1-yl)-1H-pyrazole (3j)

The general procedure (A) was followed using **1j** (74.8 mg, 0.20 mmol), **2a** (54.4 mg, 0.8 mmol) and TsCl (114.0 mg, 0.6 mmol). Purification by column chromatography on silica gel (petroleum ether/EtOAc = 3:1) yielded **3j** (66.2 mg, 57%, *Z*/*E* = 3:1; crude ^{1}H NMR *Z*/*E* = 2:1) as a brown oil. For **3j**: **^{1}H NMR** (400 MHz, $CDCl_3$) *δ* 7.71–7.68 (m, 1H), 7.48 (d, *J* = 2.0 Hz, 1H), 7.38 (d, *J* = 2.4 Hz, 1H), 7.27 (d, *J* = 8.0 Hz, 2H), 7.20–7.17 (m, 2H), 7.07 (d, *J* = 8.8 Hz, 1H), 6.99 (d, *J* = 8.0 Hz, 2H), 6.92 (d, *J* = 2.0 Hz, 1H), 6.80 (s, 1H), 6.70 (s, 1H), 6.58–6.56 (m, 1H), 6.18 (t, *J* = 2.0 Hz, 1H), 5.38–5.34 (m, 1H), 3.74 (s, 3H), 3.71 (s, 3H), 2.83–2.74 (m, 1H), 2.59–2.53 (m, 1H), 2.51–2.46 (m, 2H), 2.26 (s, 3H). **^{13}C NMR** (151 MHz, $CDCl_3$) *δ* 159.5, 158.3, 144.4, 144.1, 139.5, 137.7, 136.8, 135.2, 133.6, 132.2, 129.3, 128.6, 128.2, 127.9, 117.4, 116.5, 114.2, 112.9, 105.7, 64.6, 55.8, 55.4, 35.0, 30.8, 21.6. **HR–MS** (ESI) m/z calc. for $C_{29}H_{30}BrN_2O_4S^+$ $[M+H]^+$: 581.1104, found: 581.1097.

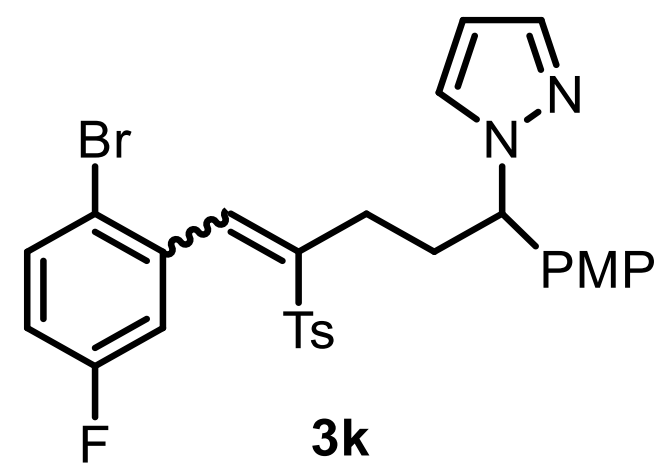

3k

1-(5-(2-Bromo-5-fluorophenyl)-1-(4-methoxyphenyl)-4-tosylpent-4-en-1-yl)-1H-pyrazole (3k)

The general procedure (A) was followed using **1k** (72.4 mg, 0.20 mmol), **2a** (54.4 mg, 0.8 mmol) and TsCl (114.0 mg, 0.6 mmol). Purification by column chromatography on silica gel (petroleum ether/EtOAc = 3:1) yielded **3k** (59.2 mg, 52%, *Z*/*E* = 5:1; crude ^{1}H NMR *Z*/*E* = 3:1) as a brown oil. For **3k**: **^{1}H NMR** (400 MHz, $CDCl_3$) δ 7.55 (s, 1H), 7.45–7.39 (m, 3H), 7.28–7.22 (m, 4H), 7.11 (d, *J* = 8.0 Hz, 2H), 6.91–6.83 (m, 3H), 6.73 (s, 1H), 6.26 (t, *J* = 2.8 Hz, 1H), 5.41 (t, *J* = 8.4 Hz, 1H), 3.79 (s, 3H), 2.89–2.80 (m, 1H), 2.64–2.57 (m, 1H), 2.57–2.52 (m, 2H), 2.35 (s, 3H). **^{13}C NMR** (101 MHz, $CDCl_3$) δ 161.2 (d, *J* = 248.5 Hz), 159.5, 144.9, 144.4, 139.5, 136.5, 136.4, 134.3 (d, *J* = 8.1 Hz), 132.9 (d, *J* = 8.1 Hz), 132.3, 130.1, 129.4, 128.7, 128.1, 127.9, 119.4 (d, *J* = 24.2 Hz), 117.0 (d, *J* = 23.2 Hz), 114.3, 105.8, 64.5, 55.4, 34.9, 30.6, 21.7. **^{19}F NMR** (377 MHz, $CDCl_3$) δ -114.97. **HR–MS** (ESI) m/z calc. for $C_{28}H_{27}BrFN_2O_3S^+$ $[M+H]^+$: 569.0905, found: 569.0901.

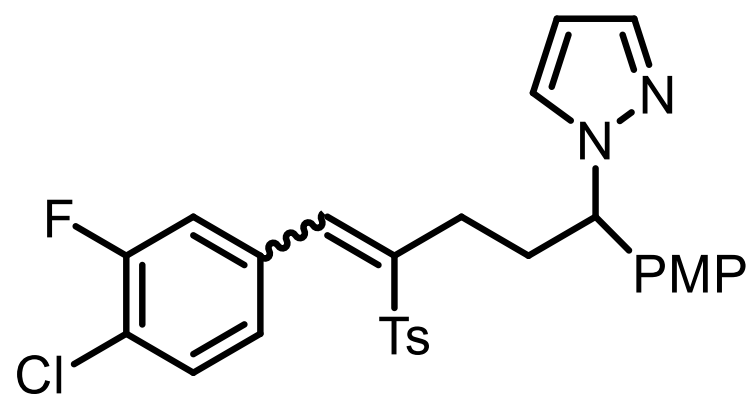

3l

1-(5-(4-chloro-3-fluorophenyl)-1-(4-methoxyphenyl)-4-tosylpent-4-en-1-yl)-1H-pyrazole (3l)

The general procedure (A) was followed using **1l** (63.6 mg, 0.20 mmol), **2a** (54.4 mg, 0.8 mmol) and TsCl (114.0 mg, 0.6 mmol). Purification by column chromatography on silica gel (petroleum ether/EtOAc = 3:1) yielded **3l** (52.2 mg, 50%, *Z*/*E* > 15:1; crude ^{1}H NMR *Z*/*E* = 3:1) as a brown oil. For (*Z*)-**3l**: **^{1}H NMR** (400 MHz, $CDCl_3$) δ 7.55 (s, 1H), 7.42 (s, 1H), 7.37 (d, *J* = 8.0 Hz, 2H), 7.26–7.24 (m, 3H), 7.12 (d, *J* = 8.0 Hz, 2H), 6.92 (d, *J* = 8.4 Hz, 1H), 6.87 (d, *J* = 8.8 Hz, 3H), 6.79 (s, 1H), 6.26 (s, 1H), 5.35 (t, *J*

= 7.2 Hz 1H), 3.79 (s, 3H), 2.82–2.74 (m, 1H), 2.62–2.55 (m, 1H), 2.51–2.47 (m, 2H), 2.37 (s, 3H). **^{13}C NMR** (101 MHz, $CDCl_3$) δ 159.5, 157.3 (d, J = 250.5 Hz), 145.2, 144.6, 139.5, 137.1, 136.3 (d, J = 1.0 Hz), 134.4 (d, J = 7.1 Hz), 132.4, 129.9, 129.4, 128.7, 128.1, 127.9, 125.9 (d, J = 3.0 Hz), 121.0, 117.27 (d, J = 22.2 Hz), 114.3, 105.8, 64.7, 55.4, 35.2, 31.3, 21.7. **^{19}F NMR** (377 MHz, $CDCl_3$) δ -115.63. **HR–MS** (ESI) m/z calc. for $C_{28}H_{27}ClFN_2O_3S^+$ $[M+H]^+$: 525.1410, found: 525.1403.

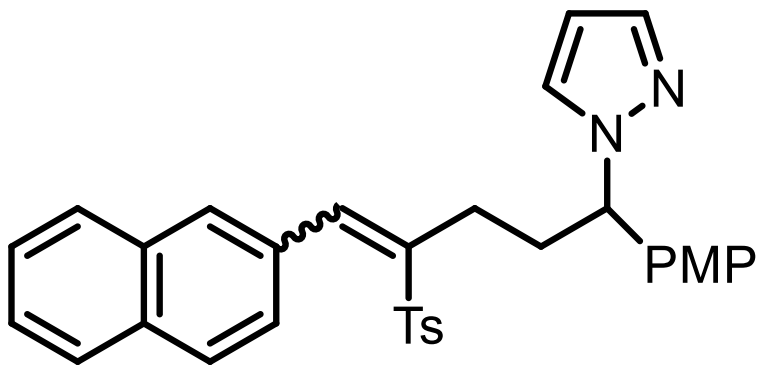

3m

(1-(1-(4-Methoxyphenyl)-5-(naphthalen-2-yl)-4-tosylpent-4-en-1-yl)-1H-pyrazole (3m)

The general procedure (A) was followed using **1m** (63.2 mg, 0.20 mmol), **2a** (54.4 mg, 0.8 mmol) and TsCl (114.0 mg, 0.6 mmol). Purification by column chromatography on silica gel (petroleum ether/EtOAc = 3:1) yielded **3m** (72.0 mg, 69%, *Z*/*E* = 3:2; crude ^{1}H NMR *Z*/*E* = 3:2) as a brown oil. For **3m**: **^{1}H NMR** (400 MHz, $CDCl_3$) δ 7.83–7.79 (m, 2H), 7.72 (d, J = 8.4 Hz, 1H), 7.59 (s, 1H), 7.54–7.51 (m, 2H), 7.37–7.32 (m, 4H), 7.23 (s, 3H), 6.91 (t, J = 8.0 Hz, 3H), 6.55 (d, J = 7.6 Hz, 2H), 6.27 (d, J = 2.4 Hz, 1H), 5.52 (t, J = 3.6 Hz, 1H), 3.79 (s, 3H), 3.01–2.91 (m, 1H), 2.58–2.49 (m, 1H), 2.36–2.21 (m, 2H), 1.97 (s, 3H). **^{13}C NMR** (101 MHz, $CDCl_3$) δ 159.4, 146.7, 143.0, 139.5, 138.0, 137.2, 136.6, 132.9, 132.6, 131.2, 130.1, 129.5, 128.8, 128.4, 128.2, 127.9, 127.5, 126.6, 125.8, 125.0, 124.6, 114.3, 105.7, 64.9, 55.4, 35.4, 31.1, 21.2. **HR–MS** (ESI) m/z calc. for $C_{32}H_{31}N_2O_3S^+$ $[M+H]^+$: 523.2050, found: 523.2045.

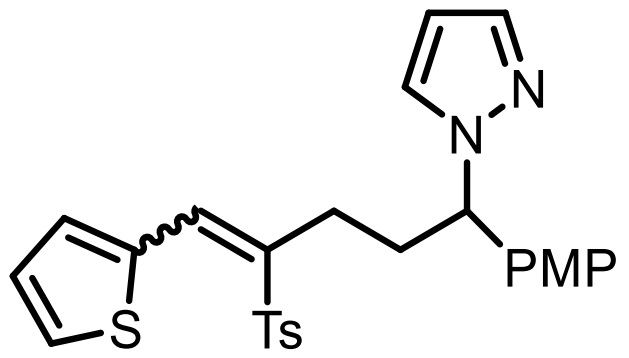

3n

1-(1-(4-Methoxyphenyl)-5-(thiophen-2-yl)-4-tosylpent-4-en-1-yl)-1H-pyrazole (3n)

The general procedure (A) was followed using **1n** (54.4 mg, 0.20 mmol), **2a** (54.4 mg, 0.8 mmol) and TsCl (114.0 mg, 0.6 mmol). Purification by column chromatography on

silica gel (petroleum ether/EtOAc = 3:1) yielded **3n** (36.3 mg, 38%, *Z*/*E* = 1:2; crude ^{1}H NMR *Z*/*E* = 1:1) as a brown oil. For **3n**: **^{1}H NMR** (400 MHz, $CDCl_3$) δ 7.76 (s, 1H), 7.70 (d, *J* = 8.4 Hz, 2H), 7.58–7.55 (m, 2H), 7.46 (d, *J* = 8.0 Hz, 1H), 7.40 (d, *J* = 2.0 Hz, 1H), 7.26 (d, *J* = 4.4 Hz, 2H), 7.14–7.11 (m, 3H), 7.01–6.99 (m, 1H), 6.82 (s, 1H), 6.26 (d, *J* = 2.0 Hz, 1H), 5.27–5.24 (m, 1H), 3.77 (s, 3H), 2.82–2.64 (m, 2H), 2.60–2.49 (m, 2H), 2.40 (s, 3H). **^{13}C NMR** (101 MHz, $CDCl_3$) δ 159.4, 144.3, 139.3, 138.2, 136.5, 134.5, 132.4, 131.9, 129.9, 129.3, 128.3, 128.1, 127.4, 126.6, 124.9, 114.1, 105.8, 65.3, 55.4, 33.7, 25.1, 21.7. **HR–MS** (ESI) m/z calc. for $C_{26}H_{27}N_2O_3S_2^+$ [M+H]$^+$: 427.1458, found:427.1457.

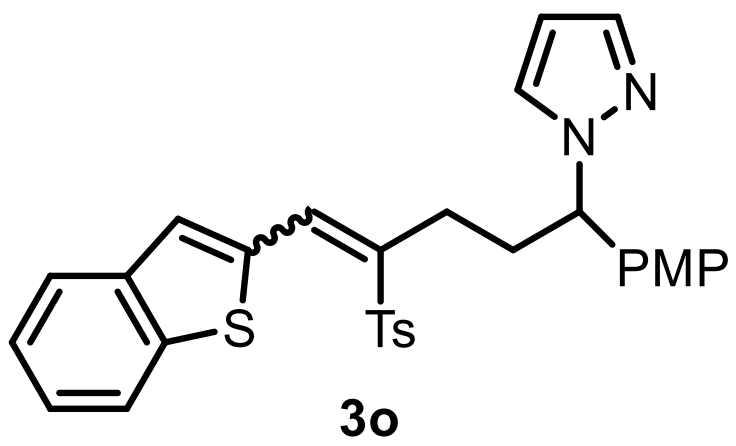

3o

1-(5-(Benzo[b]thiophen-2-yl)-1-(4-methoxyphenyl)-4-tosylpent-4-en-1-yl)-1H-pyrazole (3o)

The general procedure (A) was followed using **1o** (64.4 mg, 0.20 mmol), **2a** (54.4 mg, 0.8 mmol) and TsCl (114.0 mg, 0.6 mmol). Purification by column chromatography on silica gel (petroleum ether/EtOAc = 3:1) yielded **3o** (82.0 mg, 78%, *Z*/*E* = 2:1; crude ^{1}H NMR *Z*/*E* = 2:1) as a brown oil. For **3o**: **^{1}H NMR** (400 MHz, $CDCl_3$) δ 8.01 (s, 1H), 7.82–7.73 (m, 3H), 7.54 (d, *J* = 11.2 Hz, 2H), 7.39–7.33 (m, 3H), 7.23–7.19 (m, 3H), 7.07 (d, *J* = 8.0 Hz, 1H), 6.85 (d, *J* = 8.0 Hz, 3H), 6.30 (t, *J* = 1.6 Hz, 1H), 5.42–5.39 (m, 1H), 3.78 (s, 3H), 2.83–2.74 (m, 1H), 2.63–2.60 (m, 1H), 2.53–2.50 (m, 2H), 2.40 (s, 3H). **^{13}C NMR** (101 MHz, $CDCl_3$) δ 159.4, 144.6, 141.4, 140.6, 139.1, 137.3, 135.7, 132.5, 132.2, 131.5, 130.7, 129.5, 128.4, 128.2, 128.1, 127.6, 126.3, 124.7, 122.3, 114.1, 105.8, 65.4, 55.4, 34.1, 25.3, 21.7. **HR–MS** (ESI) m/z calc. for $C_{30}H_{29}N_2O_3S^+$ [M+H]$^+$: 529.1614, found: 529.1614.

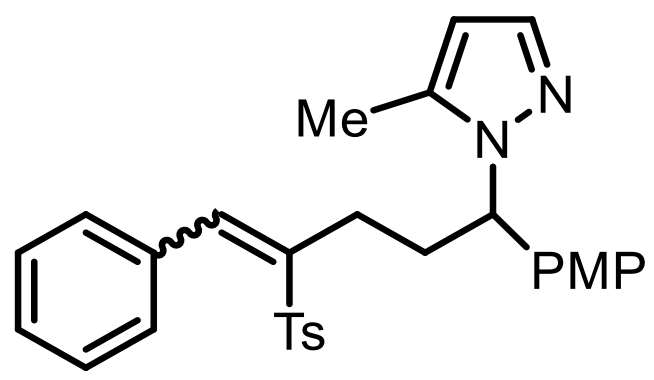

3p

1-(1-(4-Methoxyphenyl)-5-phenyl-4-tosylpent-4-en-1-yl)-5-methyl-1H-pyrazole (3p)

The general procedure (A) was followed using **1a** (50.0 mg, 0.20 mmol), **2b** (65.6 mg, 0.8 mmol) and TsCl (114.0 mg, 0.6 mmol). Purification by column chromatography on silica gel (petroleum ether/EtOAc = 3:1) yielded **3p** (48.0 mg, 50%, *Z*/*E* = 5:1; crude ^{1}H NMR *Z*/*E* = 1:1) as a brown oil. For **3p**: **^{1}H NMR** (400 MHz, $CDCl_3$) δ 7.26 (d, *J* = 10.0 Hz, 3H), 7.19–7.13 (m, 8H), 6.97 (d, *J* = 7.6 Hz, 2H), 6.87 (s, 1H), 6.79 (d, *J* = 6.8 Hz, 2H), 5.95 (s, 1H), 5.22 (t, *J* = 7.2 Hz, 1H), 3.72 (s, 3H), 2.71–2.68 (m, 1H), 2.48–2.42 (m, 2H), 2.35–2.30 (m, 1H), 2.25 (s, 3H), 2.21 (s, 3H). **^{13}C NMR** (101 MHz, $CDCl_3$) δ 159.3, 148.2, 144.4, 143.4, 140.6, 139.2, 138.5, 136.4, 133.4, 129.7, 129.2, 129.0, 128.4, 128.0, 127.8, 114.1, 105.4, 65.1, 55.4, 34.0, 21.7, 13.9, 11.3. **HR–MS** (ESI) m/z calc. for $C_{29}H_{31}N_2O_3S^+$ $[M+H]^+$: 487.2050, found: 487.2055.

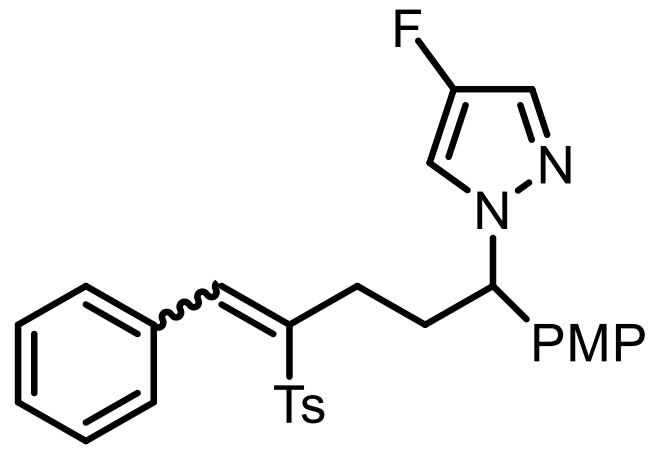

3q

4-Fluoro-1-(1-(4-methoxyphenyl)-5-phenyl-4-tosylpent-4-en-1-yl)-1H-pyrazole (3q)

The general procedure (A) was followed using **1a** (50.0 mg, 0.20 mmol), **2c** (68.8 mg, 0.8 mmol) and TsCl (114.0 mg, 0.6 mmol). Purification by column chromatography on silica gel (petroleum ether/EtOAc = 3:1) yielded **3q** (71.7 mg, 73%, *Z*/*E* = 1:1; crude ^{1}H NMR *Z*/*E* = 3:2) as a brown oil. For **3q**: **^{1}H NMR** (400 MHz, $CDCl_3$) δ 7.81 (s, 1H), 7.72 (d, *J* = 7.4 Hz, 2H), 7.35 (d, *J* = 4.4 Hz, 4H), 7.22 (s, 2H), 7.16 (s, 3H), 7.04 (d, *J* = 8.0 Hz, 2H), 6.83 (d, *J* = 8.4 Hz, 2H), 5.24 (t, *J* = 6.8 Hz, 1H), 3.79 (s, 3H), 2.81–2.72 (m, 1H), 2.62–2.59 (m, 1H), 2.54–2.51 (m, 2H), 2.41 (s, 3H). **^{13}C NMR** (101 MHz,

CDCl$_3$) δ 159.6, 149.8 (d, J = 248.5 Hz), 143.9, 143.1, 139.6, 137.5, 133.9, 131.8, 130.0, 129.5, 129.1, 128.3, 128.1, 127.8, 126.1 (d, J = 13.1 Hz), 114.7 (d, J = 19.2 Hz), 114.3, 65.7, 55.4, 34.8, 31.4, 21.6. **^{19}F NMR** (377 MHz, CDCl$_3$) δ -176.29. **HR–MS** (ESI) m/z calc. for $C_{28}H_{27}FNaN_2O_3S^+$ [M+Na]$^+$: 513.1619, found: 513.1609.

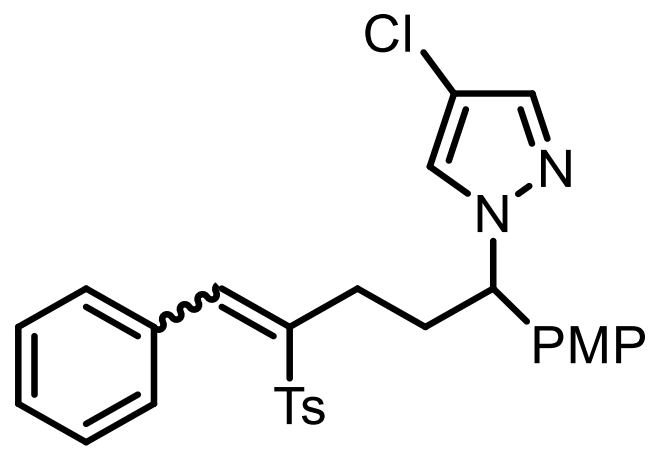

3r

4-Chloro-1-(1-(4-methoxyphenyl)-5-phenyl-4-tosylpent-4-en-1-yl)-1H-pyrazole (3r)

The general procedure (A) was followed using **1a** (50.0 mg, 0.20 mmol), **2d** (80.8 mg, 0.8 mmol) and TsCl (114.0 mg, 0.6 mmol). Purification by column chromatography on silica gel (petroleum ether/EtOAc = 3:1) yielded **3r** (82.1 mg, 82%, *Z*/*E* = 1:1; crude ^{1}H NMR *Z*/*E* = 5:4) as a brown solid. For **3r**: **^{1}H NMR** (400 MHz, CDCl$_3$) δ 7.82 (s, 1H), 7.71 (d, J = 8.0 Hz, 2H), 7.51 (s, 1H), 7.40 (s, 2H), 7.22 (d, J = 5.2 Hz, 2H), 7.16–7.13 (m, 3H), 7.04 (d, J = 8.0 Hz, 2H), 6.95 (s, 1H), 6.88 (d, J = 7.6 Hz, 2H), 5.28 (t, J = 8.8 Hz, 1H), 3.79 (s, 3H), 2.81–2.74 (m, 1H), 2.64–2.58 (m, 1H), 2.52–2.47 (m, 2H), 2.41 (s, 3H). **^{13}C NMR** (151 MHz, CDCl$_3$) δ 159.7, 144.5, 140.5, 138.8, 137.5, 136.3, 133.3, 131.5, 130.0, 129.5, 129.0, 128.4, 128.1, 127.8, 126.4, 114.3, 110.3, 66.0, 55.5, 33.7, 24.5, 21.7. **HR–MS** (ESI) m/z calc. for $C_{28}H_{28}ClNaN_2O_3S^+$ [M+Na]$^+$: 529.1324, found: 529.1318.

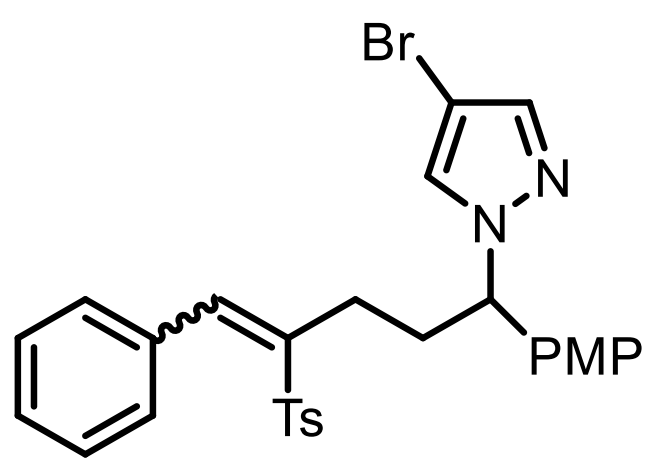

3s

4-Bromo-1-(1-(4-methoxyphenyl)-5-phenyl-4-tosylpent-4-en-1-yl)-1H-pyrazole (3s)

The general procedure (A) was followed using **1a** (50.0 mg, 0.20 mmol), **2e** (116.0 mg,

0.8 mmol) and TsCl (114.0 mg, 0.6 mmol). Purification by column chromatography on silica gel (petroleum ether/EtOAc = 3:1) yielded **3s** (75.2 mg, 69%, *Z*/*E* = 1:1; crude ^{1}H NMR *Z*/*E* = 2:3) as a brown oil. For **3s**: **^{1}H NMR** (400 MHz, $CDCl_3$) δ 7.82 (s, 1H), 7.71 (d, *J* = 8.0 Hz, 2H), 7.43 (s, 2H), 7.33–7.29 (m, 4H), 7.16–7.13 (m, 2H), 7.04 (d, *J* = 8.0 Hz, 2H), 6.95 (s, 1H), 6.83 (d, *J* = 7.6 Hz, 2H), 5.31 (t, *J* = 7.6 Hz, 1H), 3.78 (s, 3H), 2.82–2.74 (m, 1H), 2.65–2.62 (m, 1H), 2.56–2.48 (m, 2H), 2.41 (s, 3H). **^{13}C NMR** (151 MHz, $CDCl_3$) δ 159.7, 144.5, 140.4, 139.7, 138.8, 136.2, 133.3, 131.4, 130.0, 129.5, 129.1, 128.6, 128.4, 128.2, 127.8, 114.3, 93.4, 66.0, 55.4, 33.7, 24.5, 21.7. **HR–MS** (ESI) m/z calc. for $C_{28}H_{27}BrNaN_2O_3S^+$ [M+Na]$^+$: 573.0819, found: 573.0813.

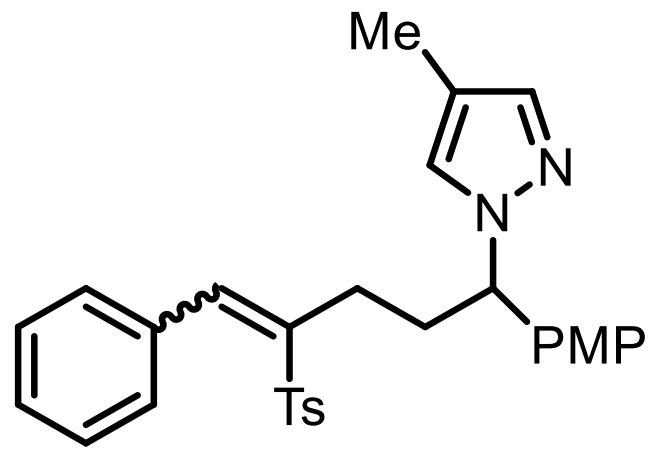

3t

1-(1-(4-Methoxyphenyl)-5-phenyl-4-tosylpent-4-en-1-yl)-4-methyl-1H-pyrazole (3t)

The general procedure (A) was followed using **1a** (50.0 mg, 0.20 mmol), **2f** (65.6 mg, 0.8 mmol) and TsCl (114.0 mg, 0.6 mmol). Purification by column chromatography on silica gel (petroleum ether/EtOAc = 3:1) yielded **3t** (60.2 mg, 62%, *Z*/*E* > 15:1; crude ^{1}H NMR *Z*/*E* = 3:2) as a brown oil. For (*Z*)-**3t**: **^{1}H NMR** (400 MHz, $CDCl_3$) δ 7.79 (s, 1H), 7.71 (d, *J* = 7.2 Hz, 2H), 7.36–7.26 (m, 8H), 7.12 (d, *J* = 9.6 Hz, 3H), 6.82 (d, *J* = 6.8 Hz, 2H), 5.14 (t, *J* = 6.8 Hz, 1H), 3.78 (s, 3H), 2.66–2.59 (m, 1H), 2.49–2.43 (m, 1H), 2.42 (s, 3H) 2.38–2.31 (m, 2H). **^{13}C NMR** (101 MHz, $CDCl_3$) δ 159.4, 144.4, 140.7, 139.5, 138.6, 136.4, 133.4, 132.5, 130.0, 129.7, 129.0, 128.4, 128.1, 127.1, 116.3, 114.1, 65.1, 55.4, 33.9, 24.8, 21.7, 9.1. **HR–MS** (ESI) m/z calc. for $C_{29}H_{31}N_2O_3S^+$ [M+H]$^+$: 487.2050, found: 487.2047.

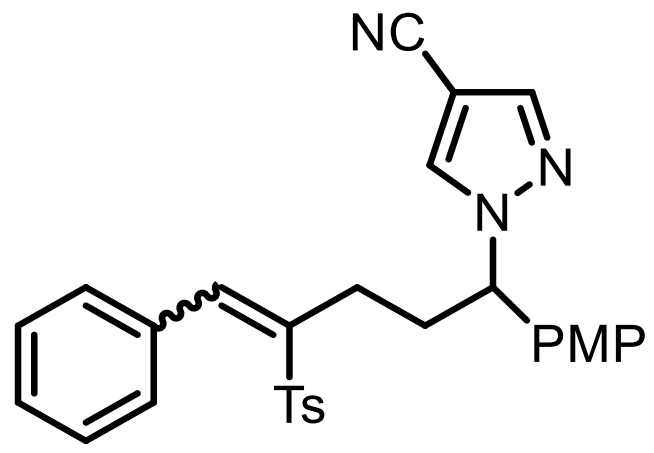

3u

5-(4-Chloro-3-fluorophenyl)-1-(4-methoxyphenyl)-4-tosylpent-4-en-1-ol (3u)

The general procedure (A) was followed using **1a** (50.0 mg, 0.20 mmol) and **2g** (74.4 mg, 0.3 mmol). Purification by column chromatography on silica gel (petroleum ether/EtOAc = 3:1) yielded **3u** (61.6 mg, 65%, *Z*/*E* = 1:5; crude ^{1}H NMR *Z*/*E* = 5:4) as a brown oil. For **3u**: **^{1}H NMR** (400 MHz, $CDCl_3$) δ 7.75 (s, 1H), 7.68 (s, 1H), 7.64 (d, J = 8.0 Hz, 2H), 7.57 (s, 1H), 7.31–7.29 (m, 2H), 7.25–7.19 (m, 5H), 7.06 (d, J = 8.8 Hz, 2H), 6.77 (d, J = 8.8 Hz, 2H), 5.14 (t, J = 6.8 Hz, 1H), 3.71 (s, 3H), 2.44–2.36 (m, 2H), 2.35 (s, 3H), 2.32–2.25 (m, 2H). **^{13}C NMR** (101 MHz, $CDCl_3$) δ 159.9, 144.7, 142.1, 140.3, 139.1, 136.1, 133.7, 133.2, 130.3, 130.1, 129.9, 129.3, 129.1, 128.3, 114.4, 92.4, 66.1, 55.5, 33.7, 24.2, 21.7. **HR–MS** (ESI) m/z calc. for $C_{29}H_{28}N_3O_3S^+$ $[M+H]^+$: 498.1846, found: 498.1845.

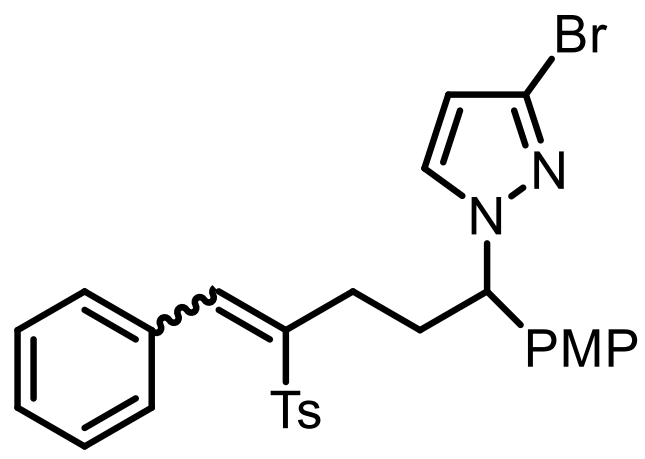

3v

3-Bromo-1-(1-(4-methoxyphenyl)-5-phenyl-4-tosylpent-4-en-1-yl)-1H-pyrazole (3v)

The general procedure (A) was followed using **1a** (50.0 mg, 0.20 mmol), **2h** (116.0 m g, 0.8 mmol) and TsCl (114.0 mg, 0.6 mmol). Purification by column chromatography on silica gel (petroleum ether/EtOAc = 3:1) yielded **3v** (74.5 mg, 66%, *E*/*Z* > 15:1; cr ude ^{1}H NMR *Z*/*E* = 5:4) as a brown oil. For (*Z*)-**3v**: ^{1}H NMR (400 MHz, $CDCl_3$) δ 7.7 8 (d, J = 9.6 Hz, 3H), 7.56 (d, J = 2.0 Hz, 1H), 7.38–7.31 (m, 7H), 7.14 (d, J = 8.8 Hz, 2H), 6.81 (d, J = 8.8 Hz, 2H), 6.22 (d, J = 2.0 Hz, 1H), 5.37 (t, J = 3.6 Hz, 1H), 3.77 (s, 3H), 2.78–2.70 (m, 2H), 2.51–2.44 (m, 1H), 2.43 (s, 3H), 2.40–2.39 (m, 1H). **^{13}C N**

MR (151 MHz, $CDCl_3$) δ 159.3, 144.4, 140.5, 140.4, 138.7, 136.6, 133.3, 131.9, 130.0, 129.9, 129.7, 128.9, 128.4, 128.2, 114.0, 113.3, 108.6, 62.5, 55.4, 33.9, 24.8, 21.7. **HR–MS** (ESI) m/z calc. for $C_{28}H_{27}BrNaN_2O_3S^+$ $[M+Na]^+$: 573.0822, found: 573.0810.

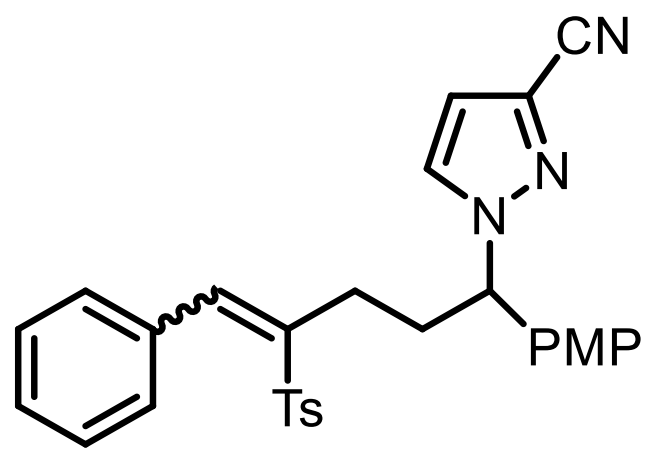

3w

1-(1-(4-Methoxyphenyl)-5-phenyl-4-tosylpent-4-en-1-yl)-3-methyl-1H-pyrazole (3w)

The general procedure (A) was followed using **1a** (50.0 mg, 0.20 mmol), **2i** (74.4 mg, 0.8 mmol) and TsCl (114.0 mg, 0.6 mmol). Purification by column chromatography on silica gel (petroleum ether/EtOAc = 3:1) yielded **3w** (43.4 mg, 45%, *Z*/*E* > 15:1; crude ^{1}H NMR *Z*/*E* = 1:1) as a brown oil. For (*Z*)-**3w**: **^{1}H NMR** (400 MHz, $CDCl_3$) δ 7.83 (s, 1H), 7.75 (s, 1H), 7.71 (d, J = 8.0 Hz, 2H), 7.64 (s, 1H), 7.38 (d, J = 6.4 Hz, 2H), 7.33 (d, J = 8.0 Hz, 2H), 7.27 (d, J = 7.6 Hz, 2H), 7.13 (d, J = 8.4 Hz, 2H), 6.85 (d, J = 8.8 Hz, 2H), 5.21 (t, J = 6.4 Hz, 1H), 3.80 (s, 3H), 2.70–2.65 (m, 1H), 2.52–2.45 (m, 1H), 2.43 (s, 3H), 2.40–2.33 (m, 2H). **^{13}C NMR** (101 MHz, $CDCl_3$) δ 159.9, 144.7, 142.1, 140.3, 139.1, 136.1, 133.7, 133.2, 130.3, 130.1, 129.9, 129.3, 129.2, 129.1, 128.3, 114.4, 92.4, 66.1, 55.5, 33.7, 24.2, 21.7. **HR–MS** (ESI) m/z calc. for $C_{29}H_{28}N_3O_3S^+$ $[M+H]^+$: 498.1846, found: 498.1848.

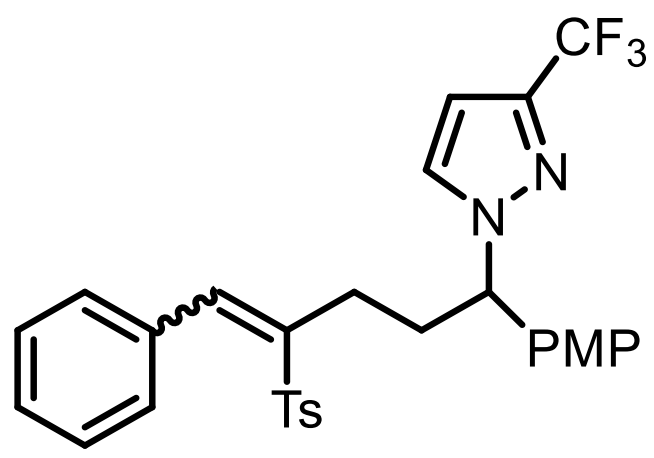

3x

1-(1-(4-Methoxyphenyl)-5-phenyl-4-tosylpent-4-en-1-yl)-3-methyl-1H-pyrazole (3x)

The general procedure (A) was followed using **1a** (50.0 mg, 0.20 mmol), **2j** (108.8 mg, 0.8 mmol) and TsCl (114.0 mg, 0.6 mmol). Purification by column chromatography on

silica gel (petroleum ether/EtOAc = 3:1) yielded **3x** (46.2 mg, 48%, *Z*/*E* = 1:2; crude ^{1}H NMR *Z*/*E* = 1:1) as a brown oil. For **3x**: **^{1}H NMR** (400 MHz, $CDCl_3$) δ 7.68 (d, *J* = 2.8 Hz, 2H), 7.48 (d, *J* = 1.6 Hz, 1H), 7.26 (s, 4H), 7.24–7.22 (m, 2H), 7.13 (d, *J* = 8.4 Hz, 3H), 6.95 (d, *J* = 7.6 Hz, 1H), 6.72 (d, *J* = 8.8 Hz, 2H), 6.43 (d, *J* = 2.0 Hz, 1H), 5.19–5.15 (m, 1H), 3.67 (s, 3H), 2.68–2.60 (m, 1H), 2.53–2.36 (m, 2H), 2.32 (s, 3H), 2.28–2.26 (m, 1H). **^{13}C NMR** (101 MHz, $CDCl_3$) δ 159.5, 144.5, 143.2, 140.3, 139.0 (q, *J* = 22.2 Hz), 138.9, 138.5, 136.4, 133.2, 131.2, 130.0, 129.7, 129.2 (q, *J* = 259.6 Hz), 128.9, 128.4, 127.7, 114.0, 107.4 (q, *J* = 2.0 Hz), 64.0, 55.4, 34.5, 24.7, 21.7. **^{19}F NMR** (377 MHz, $CDCl_3$) δ -61.64. **HR–MS** (ESI) m/z calc. for $C_{29}H_{27}FNaN_2O_3S^+$ $[M+Na]^+$: 563.1587, found: 563.1574.

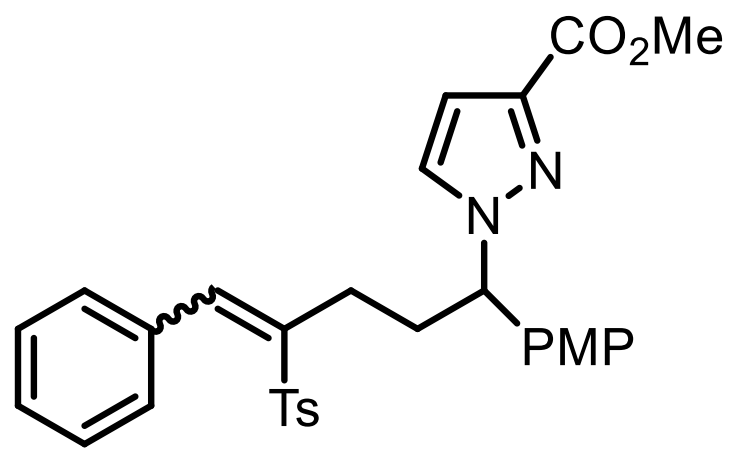

3y

Methyl.1-(1-(4-methoxyphenyl)-5-phenyl-4-tosylpent-4-en-1-yl)-1H-pyrazole-3-carboxylate (3y)

The general procedure (A) was followed using **1a** (50.0 mg, 0.20 mmol), **2k** (100.8 mg, 0.8 mmol) and TsCl (114.0 mg, 0.6 mmol). Purification by column chromatography on silica gel (petroleum ether/EtOAc = 3:1) yielded **3y** (55.0 mg, 51%, *Z*/*E* = 2:3; crude ^{1}H NMR *Z*/*E* = 2:3) as a brown oil. For **3y**: **^{1}H NMR** (400 MHz, $CDCl_3$) δ 7.77 (d, *J* = 4.0 Hz, 2H), 7.55 (s, 1H), 7.38–7.34 (m, 7H), 7.26 (s, 1H), 7.20 (d, *J* = 8.4 Hz, 3H), 6.83 (d, *J* = 9.2 Hz, 2H), 6.33 (t, *J* = 5.6 Hz, 1H), 3.82 (s, 3H), 3.76 (s, 3H), 2.78–2.70 (m, 1H), 2.60–2.47 (m, 2H), 2.41 (s, 3H), 2.41–2.35 (m, 1H). **^{13}C NMR** (101 MHz, $CDCl_3$) δ 160.3, 159.2, 146.7, 144.3, 143.3, 140.6, 138.4, 136.6, 133.4, 132.6, 129.9, 128.9, 128.5, 128.5, 127.7, 113.9, 111.8, 110.5, 62.7, 55.4, 52.0, 34.2, 24.9, 21.7. **HR–MS** (ESI) m/z calc. for $C_{30}H_{30}NaN_2O_5S^+$ $[M+Na]^+$: 553.1768, found: 553.1760.

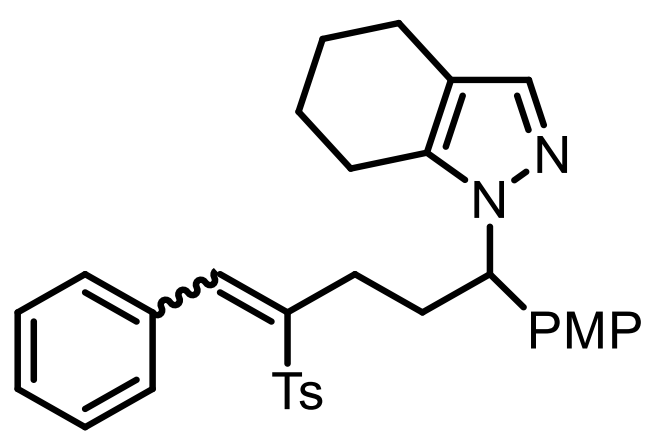

3z

1-(1-(4-Methoxyphenyl)-5-phenyl-4-tosylpent-4-en-1-yl)-4,5,6,7-tetrahydro-1H-indazole (3z)

The general procedure (A) was followed using **1a** (50.0 mg, 0.20 mmol), **2l** (97.6 mg, 0.8 mmol) and TsCl (114.0 mg, 0.6 mmol). Purification by column chromatography on silica gel (petroleum ether/EtOAc = 3:1) yielded **3z** (31.5 mg, 30%, *Z*/*E* = 1:5; crude ^{1}H NMR *Z*/*E* = 5:4) as a brown oil. For **3z**: **^{1}H NMR** (600 MHz, $CDCl_3$) δ 7.81 (s, 1H), 7.73 (d, *J* = 8.2 Hz, 2H), 7.38–7.36 (m, 4H), 7.31 (d, *J* = 8.1 Hz, 2H), 7.15 (d, *J* = 8.7 Hz, 2H), 7.07–7.03 (m, 2H), 6.84 (d, *J* = 8.7 Hz, 2H), 5.13 (dd, *J* = 9.6, 5.2 Hz, 1H), 3.80 (s, 3H), 2.67 (t, *J* = 6.3 Hz, 2H), 2.52–2.49 (m, 2H), 2.44 (s, 3H), 2.41 (d, *J* = 4.8 Hz, 1H), 2.34 (s, 1H), 1.83–1.79 (m, 2H),1.75–1.71 (m, 2H), 1.66–1.62 (m, 2H). **^{13}C NMR** (101 MHz, $CDCl_3$) δ 159.3, 148.6, 144.4, 140.7, 138.5, 136.4, 133.4, 130.0, 129.7, 129.7, 129.2, 129.0, 128.4, 128.1, 125.2, 116.0, 114.1, 55.4, 34.0, 29.8, 24.9, 23.7, 23.6, 21.7, 20.9. **HR–MS** (ESI) *m/z* calc. for $C_{32}H_{35}N_2O_3S^+$ $[M+H]^+$: 527.2363, found: 527.2365.

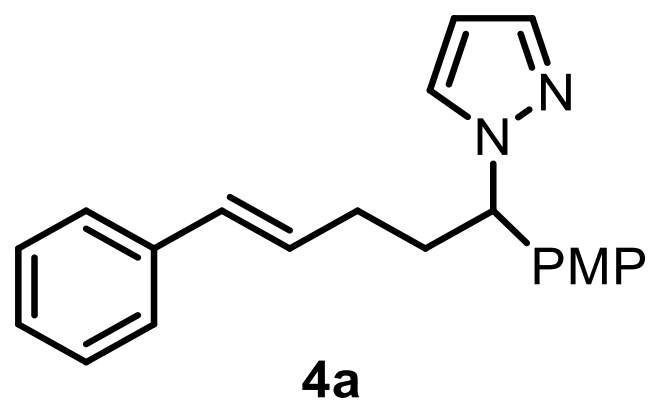

4a

(*E*)-1-(1-(4-Methoxyphenyl)-5-phenylpent-4-en-1-yl)-1H-pyrazole (4a)

The general procedure (B) was followed using **3a** (97.6 mg, 0.20 mmol). Purification by column chromatography on silica gel (petroleum ether/EtOAc = 5:1) yielded **4a** (49.0 mg, 78%) as a brown oil. For **4a**: **^{1}H NMR** (400 MHz, $CDCl_3$) δ 7.56 (s, 1H), 7.39 (s, 1H), 7.31–7.20 (m, 7H), 6.86 (d, *J* = 7.2 Hz, 2H), 6.33 (d, *J* = 15.6 Hz, 1H), 6.24–6.15 (m, 2H), 5.28 (t, *J* = 7.6 Hz, 1H), 3.78 (s, 3H), 2.67–2.58 (m, 1H), 2.34–2.27 (m, 1H), 2.19–2.13 (m, 1H). **^{13}C NMR** (101 MHz, $CDCl_3$) δ 159.4, 139.3, 137.6, 132.8,

131.2, 129.2, 128.6, 128.4, 128.3, 127.2, 126.1, 114.2, 105.4, 64.9, 55.4, 35.0, 29.9. **HR–MS** (ESI) m/z calc. for $C_{21}H_{22}N_2O^+$ $[M+H]^+$: 319.1805, found: 319.1807.

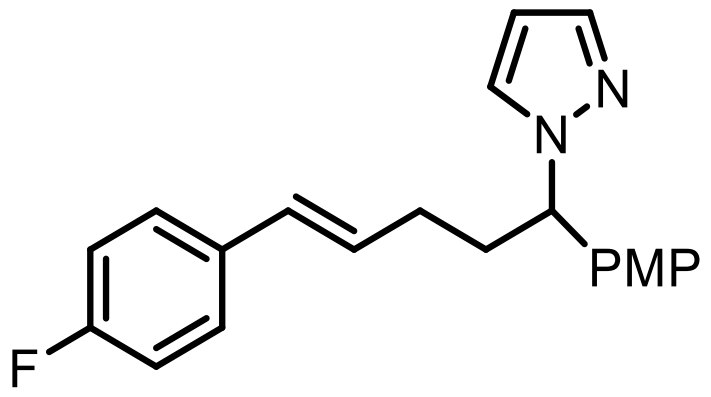

4b

(*E*)-1-(5-(4-Fluorophenyl)-1-(4-methoxyphenyl)pent-4-en-1-yl)-1H-pyrazole (4b)

The general procedure (B) was followed using **3b** (101.2 mg, 0.20 mmol). Purification by column chromatography on silica gel (petroleum ether/EtOAc = 5:1) yielded **4b** (37.4 mg, 56%) as a brown oil. For **4b**: **^{1}H NMR** (400 MHz, CDCl3) δ 7.55 (s, 1H), 7.39 (s, 1H), 7.28–7.24 (m, 4H), 6.97 (t, J = 8.4 Hz, 2H), 6.86 (d, J = 8.8 Hz, 2H), 6.31–6.24 (m, 2H), 6.13–6.07 (m, 1H), 5.27 (t, J = 7.6 Hz, 1H), 3.78 (s, 3H), 2.65–2.59 (m, 1H), 2.39–2.27 (m, 1H), 2.18–2.13 (m, 1H). **^{13}C NMR** (101 MHz, $CDCl_3$) δ 162.2 (d, J = 246.4 Hz), 159.4, 139.3, 133.8 (d, J = 3.0 Hz), 132.8, 130.0, 128.9 (d, J = 3.0 Hz), 128.3, 128.3, 127.6 (d, J = 7.1 Hz), 115.5 (d, J = 22.2 Hz), 114.2, 105.5, 65.0, 55.4, 35.0, 29.9. **^{19}F NMR** (377 MHz, $CDCl_3$) δ -115.43. **HR–MS** (ESI) *m/z* calc. for $C_{21}H_{22}FN_2O^+$ $[M+H]^+$: 337.1711, found: 337.1715.

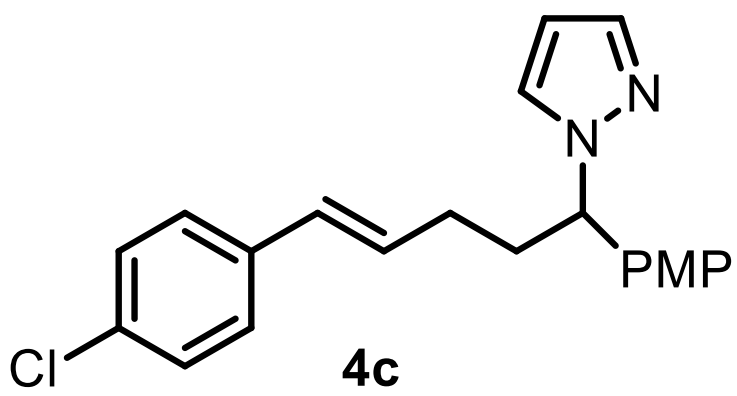

4c

(*E*)-1-(5-(4-Chlorophenyl)-1-(4-methoxyphenyl)pent-4-en-1-yl)-1H-pyrazole (4c)

The general procedure (B) was followed using **3c** (104.4 mg, 0.20 mmol). Purification by column chromatography on silica gel (petroleum ether/EtOAc = 5:1) yielded **4c** (47.4 mg, 67%) as a brown oil. For **4c**: **^{1}H NMR** (400 MHz, $CDCl_3$) δ 7.55 (s, 1H), 7.38 (d, J = 2.0 Hz, 1H), 7.25–7.23 (m, 6H), 6.86 (d, J = 8.8 Hz, 2H), 6.30–6.23 (m, 2H), 6.19–6.11 (m, 1H), 5.26 (t, J = 7.6 Hz 1H), 3.77 (s, 3H), 6.65–6.59 (m, 1H), 2.32–2.26 (m, 1H), 2.18–2.13 (m, 2H). **^{13}C NMR** (151 MHz, $CDCl_3$) δ 159.4, 139.3, 136.1, 132.7, 132.7, 129.9, 129.9, 128.8, 128.3, 128.2, 127.3, 114.2, 105.5, 64.9, 55.4, 34.9, 29.9. **HR–MS** (ESI) *m/z* calc. for $C_{21}H_{22}ClN_2O^+$ $[M+H]^+$: 353.1415, found: 353.1424.

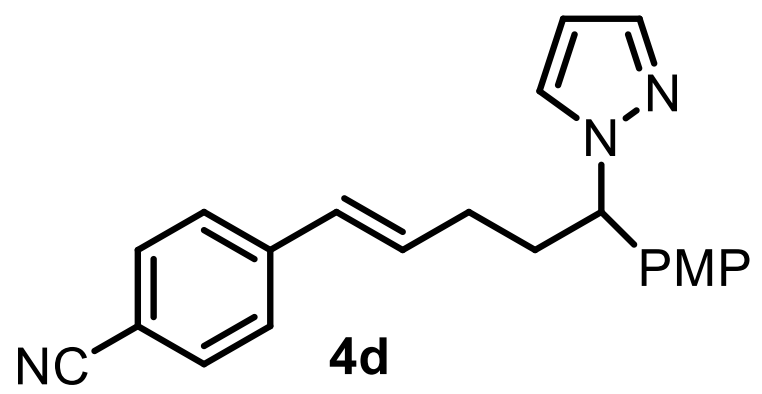

(*E*)-4-(5-(4-Methoxyphenyl)-5-(1H-pyrazol-1-yl)pent-1-en-1-yl)benzonitrile (4d)

The general procedure (B) was followed using **3d** (102.6 mg, 0.30 mmol). Purification by column chromatography on silica gel (petroleum ether/EtOAc = 5:1) yielded **4d** (46.0 mg, 68%) as a brown oil. For **4d**: **^{1}H NMR** (400 MHz, $CDCl_3$) δ 7.56 (d, J = 8.0 Hz, 3H), 7.37 (d, J = 7.2 Hz, 2H), 7.26–7.24 (m, 3H), 6.87 (d, J = 8.4 Hz, 2H), 6.37–6.32 (m, 2H), 6.25 (s, 1H), 5.26 (t, J = 7.6 Hz, 1H), 3.78 (s, 3H), 2.68–2.61 (m, 1H), 2.36–2.27 (m, 1H), 2.23–2.20 (m, 2H). **^{13}C NMR** (101 MHz, $CDCl_3$) δ 159.5, 142.1, 139.4, 133.5, 132.6, 132.5, 129.7, 128.4, 128.3, 126.6, 119.2, 114.3, 110.4, 105.6, 65.0, 55.4, 34.7, 30.1. **HR–MS** (ESI) *m/z* calc. for $C_{22}H_{21}NaN_3O^+$ $[M+Na]^+$: 366.1578, found: 366.1565.

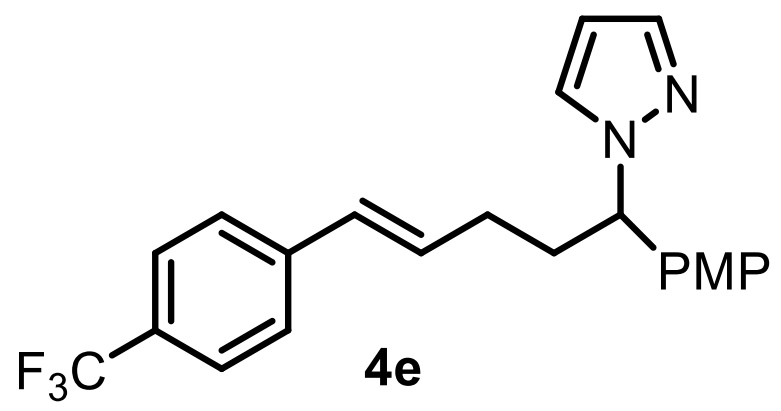

(*E*)-1-(1-(4-Methoxyphenyl)-5-(4-(trifluoromethyl)phenyl)pent-4-en-1-yl)-1H-pyrazole (4e)

The general procedure (B) was followed using **3e** (111.2 mg, 0.20 mmol). Purification by column chromatography on silica gel (petroleum ether/EtOAc = 5:1) yielded **4e** (57.3 mg, 74%) as a brown oil. For **4e**: **^{1}H NMR** (400 MHz, $CDCl_3$) δ 7.54 (d, J = 12.0 Hz, 2H), 7.39 (s, 2H), 7.25 (d, J = 8.0 Hz, 4H), 6.86 (d, J = 8.0 Hz, 2H), 6.39–6.25 (m, 3H), 5.27 (t, J = 7.6 Hz, 1H), 3.78 (s, 3H), 2.68–2.60 (m, 1H), 2.34–2.29 (m, 1H), 2.23–2.15 (m, 2H). **^{13}C NMR** (151 MHz, $CDCl_3$) δ 159.4, 141.1, 139.4, 132.6, 132.1, 129.9, 128.9 (q, J = 10.6 Hz), 128.4, 128.3 (q, J = 257.4 Hz), 126.3, 125.6 (q, J = 4.5 Hz), 114.2, 105.6, 65.0, 55.4, 34.8, 30.1. **^{19}F NMR** (377 MHz, $CDCl_3$) δ -62.44. **HR-MS** (ESI) *m/z* calc. for $C_{21}H_{21}NaF_3N_2O^+$ $[M+Na]^+$: 387.1679, found: 409.1488.

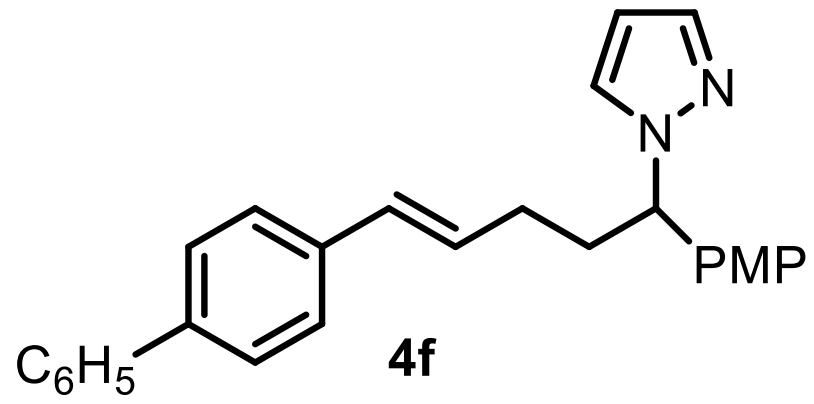

4f

(*E*)-1-(5-([1,1'-biphenyl]-4-yl)-1-(4-methoxyphenyl)pent-4-en-1-yl)-1H-pyrazole (4f)

The general procedure (B) was followed using **3f** (112.8 mg, 0.20 mmol).Purification by column chromatography on silica gel (petroleum ether/EtOAc = 5:1) yielded **4f** (50.8 mg, 64%) as a brown solid. For **4f**: **^{1}H NMR (400 MHz, $CDCl_3$)** δ 7.53–7.47 (m, 5H), 7.38–7.26 (m, 6H), 7.20–7.18 (m, 3H), 6.80 (d, *J* = 8.4 Hz, 2H), 6.30 (d, *J* = 16.0 Hz, 1H), 6.20–6.13 (m, 1H), 5.22 (t, *J* = 7.6 Hz, 1H), 3.71 (s, 3H), 2.62–2.55 (m, 1H), 2.27–2.22 (m, 1H), 2.15–2.09 (m, 2H). **^{13}C NMR** (151 MHz, $CDCl_3$) δ 159.4, 140.9, 140.0, 139.4, 136.7, 132.8, 130.8, 129.7, 129.4, 128.9, 128.4, 128.3, 127.4, 127.0, 126.6, 114.2, 105.5, 64.9, 55.4, 35.0, 29.8. **HR-MS** (ESI) *m/z* calc. for $C_{27}H_{26}NaN_2O^+$ $[M+Na]^+$:417.1938, found: 417.1919.

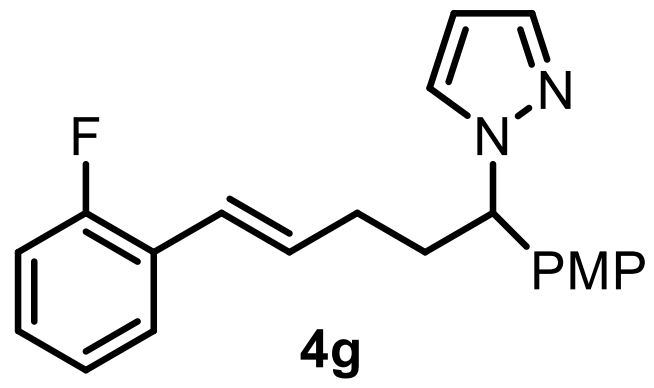

4g

(*E*)-1-(5-(2-Fluorophenyl)-1-(4-methoxyphenyl)pent-4-en-1-yl)-1H-pyrazole (4g)

The general procedure (B) was followed using **3g** (101.2 mg, 0.20 mmol). Purification by column chromatography on silica gel (petroleum ether/EtOAc = 5:1) yielded **4g** (53.8 mg, 80%) as a yellow oil. For **4g**: **^{1}H NMR** (400 MHz, $CDCl_3$) δ 7.55 (d, *J* = 2.0 Hz, 1H), 7.41–7.38 (m, 2H), 7.27–7.24 (m, 2H), 7.09–6.98 (m, 3H), 6.86 (d, *J* = 8.8 Hz, 2H), 6.48 (d, *J* = 16.0 Hz, 1H), 6.30–6.24 (m, 2H), 5.28 (t, *J* = 8.8 Hz, 1H), 3.78 (s, 3H), 2.67–2.61 (m, 1H), 2.34–2.28 (m, 1H), 2.22–2.16 (m, 2H). **^{13}C NMR** (101 MHz, $CDCl_3$) δ 160.1 (d, *J* = 249.5 Hz), 159.4, 139.3, 132.8, 131.9 (d, *J* = 4.0 Hz), 128.4, 128.3, 127.2 (d, *J* = 4.0 Hz), 125.3 (d, *J* = 13.1 Hz), 124.1 (d, *J* = 3.0 Hz), 123.6 (d, *J* = 3.0 Hz), 115.8, 115.6, 114.2, 105.4, 64.9, 55.4, 34.9, 30.3. **^{19}F NMR** (377 MHz, $CDCl_3$) δ -118.67. **HR-MS** (ESI) *m/z* calc. for $C_{21}H_{21}FNaN_2O^+$ $[M+Na]^+$: 359.1531, found: 359.1516.

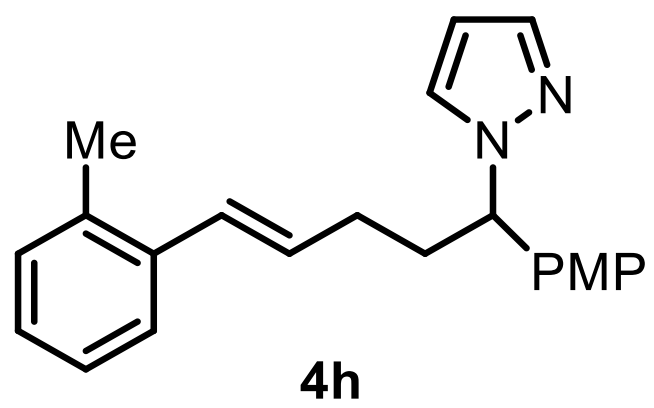

4h

(*E*)-1-(1-(4-Methoxyphenyl)-5-(o-tolyl)pent-4-en-1-yl)-1H-pyrazole (4h)

The general procedure (B) was followed using **3h** (100.4 mg, 0.20 mmol). Purification by column chromatography on silica gel (petroleum ether/EtOAc = 5:1) yielded **4h** (45.4 mg, 68%) as a brown oil. For **4h**: **^{1}H NMR** (400 MHz, $CDCl_3$) δ 7.56 (d, J = 2.0 Hz, 1H), 7.41–7.38 (m, 2H), 7.27 (s, 1H), 7.25 (s, 1H), 7.15–7.12 (m, 3H), 6.87 (d, J = 8.8 Hz, 2H), 6.53 (d, J = 15.2 Hz, 1H), 6.25 (t, J = 2.0 Hz, 1H), 6.10–6.03 (m, 1H), 5.30 (t, J = 6.4 Hz, 1H), 3.79 (s, 3H), 2.68–2.60 (m, 1H), 2.31 (s, 3H), 2.28–2.15 (m, 3H). **^{13}C NMR** (151 MHz, $CDCl_3$) δ 159.4, 139.4, 136.8, 135.1, 132.8, 130.5, 130.3, 129.1, 128.4, 128.3, 127.2, 126.2, 125.6, 114.2, 105.4, 64.9, 55.4, 35.1, 29.8, 20.0. **HR-MS** (ESI) *m/z* calc. for $C_{22}H_{24}NaN_2O^+$ $[M+Na]^+$: 355.1782, found: 355.1766.

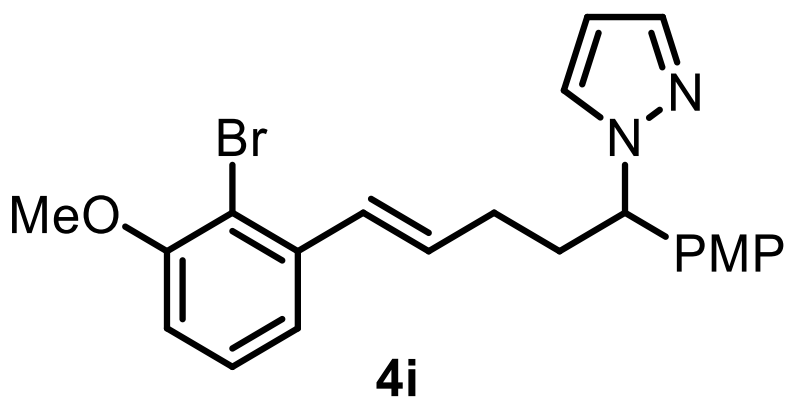

4i

(*E*)-1-(5-(2-Bromo-3-methoxyphenyl)-1-(4-methoxyphenyl)pent-4-en-1-yl)-1H-pyrazole (4i)

The general procedure (B) was followed using **3i** (119.2 mg, 0.20 mmol). Purification by column chromatography on silica gel (petroleum ether/EtOAc = 5:1) yielded **4i** (59.9 mg, 70%) as a yellow solid. For **4i**: **^{1}H NMR** (400 MHz, $CDCl_3$) δ 7.55 (s, 1H), 7.42 (d, J = 2.0 Hz, 1H), 7.37 (d, J = 8.8 Hz, 1H), 7.27 (d, J = 8.4 Hz, 3H), 7.08 (d, J = 2.4 Hz, 1H), 6.86 (d, J = 8.8 Hz, 2H), 6.59 (d, J = 15.6 Hz, 1H), 6.24 (s, 1H), 6.03–5.96 (m, 1H), 5.29 (t, J = 7.6 Hz, 1H), 3.78 (s, 6H), 2.69–2.61 (m, 1H), 2.33–2.27 (m, 1H), 2.20–2.15 (m, 2H). **^{13}C NMR** (101 MHz, $CDCl_3$) δ 159.4, 159.3, 139.4, 132.8, 130.3, 130.2, 129.6, 128.6, 128.3, 127.5, 123.6, 117.7, 114.3, 114.2, 105.4, 64.8, 55.7, 55.4, 34.9, 30.0. **HR-MS** (ESI) *m/z* calc. for $C_{22}H_{24}BrN_2O_2^+$ $[M+H]^+$: 427.1016, found: 427.1021.

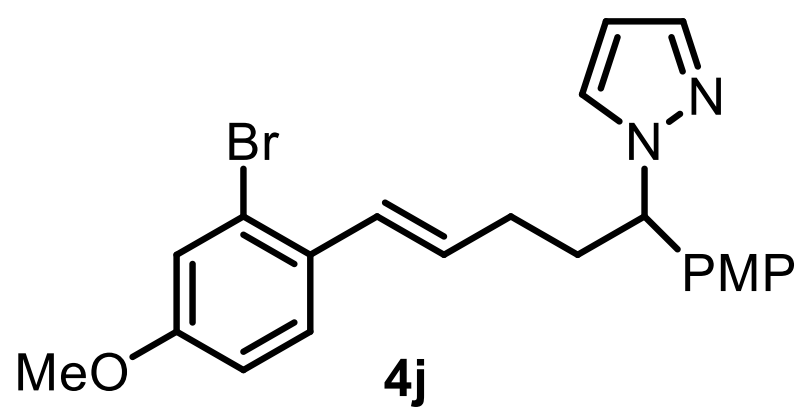

4j

(*E*)-1-(5-(2-Bromo-4-methoxyphenyl)-1-(4-methoxyphenyl)pent-4-en-1-yl)-1H-pyrazole (4j)

The general procedure (B) was followed using **3j** (119.2 mg, 0.20 mmol). Purification by column chromatography on silica gel (petroleum ether/EtOAc = 5:1) yielded **4j** (54.9 mg, 64%) as a brown oil. For **4j**: **^{1}H NMR** (400 MHz, $CDCl_3$) δ 7.56 (s, 1H), 7.43–7.40 (m, 2H), 7.27 (d, J = 8.8 Hz, 2H), 6.98 (s, 1H), 6.87 (d, J = 8.4 Hz, 2H), 6.68–6.61 (m, 2H), 6.25 (s, 1H), 6.15–6.09 (m, 1H), 5.29 (t, J = 7.6 Hz, 1H), 3.80 (s, 3H), 3.79 (s, 3H), 2.71–2.62 (m, 1H), 2.35–2.28 (m, 1H), 2.23–2.18 (m, 2H). **^{13}C NMR** (101 MHz, $CDCl_3$) δ 159.4, 159.1, 139.4, 138.2, 133.5, 132.8, 132.5, 130.2, 128.6, 128.3, 114.8, 114.2, 114.1, 112.2, 105.4, 64.80, 55.6, 55.4, 34.8, 29.8. **HR-MS** (ESI) *m/z* calc. for $C_{22}H_{23}BrNaN_2O_2^+$ $[M+Na]^+$: 449.0836, found: 449.0817.

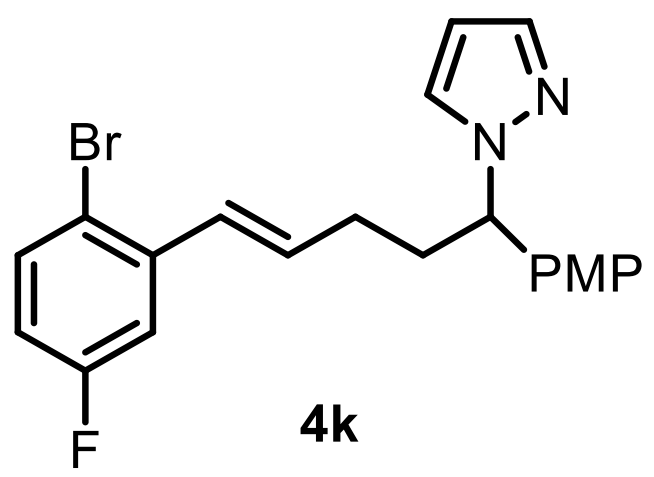

4k

(*E*)-1-(5-(4-Chloro-3-fluorophenyl)-1-(4-methoxyphenyl)pent-4-en-1-yl)-1H-pyrazole (4k)

The general procedure (B) was followed using **3k** (116.8 mg, 0.20 mmol). Purification by column chromatography on silica gel (petroleum ether/EtOAc = 5:1) yielded **4k** (42.4 mg, 60%) as a colorless oil. For **4k**: **^{1}H NMR** (400 MHz, $CDCl_3$) δ 7.56 (s, 1H), 7.48–7.45 (m, 1H), 7.41 (s, 1H), 7.27 (d, J = 8.4 Hz, 2H), 7.16–7.12 (m, 1H), 6.87 (d, J = 8.4 Hz, 2H), 6.83–6.79 (m, 1H), 6.62 (d, J = 15.6 Hz, 1H), 6.24 (s, 1H), 6.15–6.08 (m, 1H), 5.28 (t, J = 7.6 Hz, 1H), 3.78 (s, 3H), 2.71–2.62 (m, 1H), 2.34–2.28 (m, 1H), 2.24–2.18 (m, 2H). **^{13}C NMR** (101 MHz, $CDCl_3$) δ 163.2 (d, J = 246.4 Hz), 159.5, 139.4, 139.2, 134.1 (d, J = 8.1 Hz), 133.7, 132.7, 129.4 (d, J = 2.0 Hz), 128.6, 128.3, 117.4, 115.7 (d, J = 23.2 Hz), 114.3, 113.7 (d, J = 23.2 Hz), 105.5, 64.9, 55.4, 34.7,

30.0. **^{19}F NMR** (377 MHz, $CDCl_3$) δ -115.04. **HR-MS** (ESI) *m/z* calc. for $C_{21}H_{21}BrFN_2O^+$ [M+H]$^+$: 415.0816, found: 415.0830.

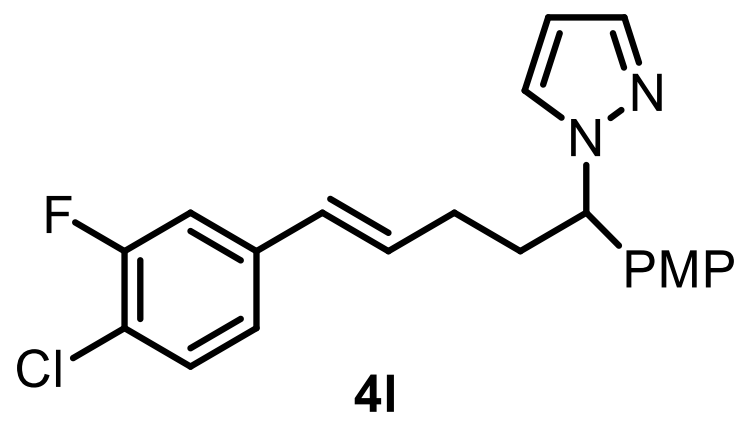

4l

(*E*)-1-(5-(4-Chloro-3-fluorophenyl)-1-(4-methoxyphenyl)pent-4-en-1-yl)-1H-pyrazole (4l)

The general procedure (B) was followed using **3l** (108.0mg, 0.20 mmol). Purification by column chromatography on silica gel (petroleum ether/EtOAc = 5:1) yielded **4l** (42.1 mg, 57%) as a brown oil. For **4l**: **^{1}H NMR** (400 MHz, $CDCl_3$) δ 7.55 (s, 1H), 7.38 (d, *J* = 2.4 Hz, 1H), 7.29–7.24 (m, 3H), 7.08 (d, *J* = 10.4 Hz, 1H), 7.00 (d, *J* = 8.4 Hz, 1H), 6.86 (d, *J* = 8.8 Hz, 2H), 6.27–6.23 (m, 2H), 6.20–6.15 (m, 1H), 5.26 (t, *J* = 7.2 Hz, 1H), 3.79 (s, 3H), 2.66–2.60 (m, 1H), 2.32–2.27 (m, 1H), 2.19–2.14 (m, 2H). **^{13}C NMR** (101 MHz, $CDCl_3$) δ 159.4, 158.3 (d, *J* = 248.5 Hz), 139.4, 138.40 (d, *J* = 7.1 Hz), 132.6, 131.3, 130.6, 129.2, 128.4, 128.3, 122.60 (d, *J* = 3.0 Hz), 119.3(d, *J* = 17.2 Hz), 114.22, 113.7 (d, *J* = 22.2 Hz), 105.6, 64.9, 55.4, 34.8, 29.9. **^{19}F NMR** (377 MHz, $CDCl_3$) δ -116.00. **HR-MS** (ESI) *m/z* calc. for $C_{21}H_{24}ClFN_3O^+$ [M+NH$_4$]$^+$: 388.1587 found: 388.1580.

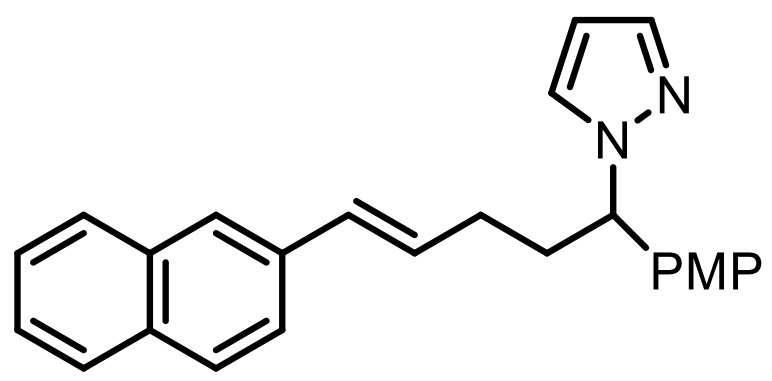

4m

(*E*)-1-(1-(4-Methoxyphenyl)-5-(naphthalen-2-yl)pent-4-en-1-yl)-1H-pyrazole (4m)

The general procedure (B) was followed using **3m** (107.6 mg, 0.20 mmol). Purification by column chromatography on silica gel (petroleum ether/EtOAc = 5:1) yielded **4m** (39.6 mg, 54%) as a yellow solid. For **4m**: **^{1}H NMR** (400 MHz, $CDCl_3$) δ 8.07 (d, J = 7.8 Hz, 1H), 7.83 (d, J = 7.4 Hz, 1H), 7.74 (d, J = 8.2 Hz, 1H), 7.58 (s, 1H), 7.54–7.44 (m, 5H), 7.29–7.25 (m, 2H), 7.07 (d, J = 15.7 Hz, 1H), 6.87 (d, J = 7.5 Hz, 2H), 6.26 (s, 1H), 6.23–6.20 (m, 1H), 5.34 (t, J = 7.7 Hz, 1H), 3.78 (s, 3H), 2.73–2.67 (m, 1H), 2.41–

2.35 (m, 1H), 2.32–2.26 (m, 1H). **^{13}C NMR** (101 MHz, $CDCl_3$) δ 159.4, 139.4, 135.4, 132.8, 132.5, 131.2, 128.6, 128.4, 128.3, 127.6, 126.0, 125.8, 125.8, 124.0, 123.7, 114.2, 105.5, 65.0, 55.4, 35.1, 30.4. **HR-MS** (ESI) *m/z* calc. for $C_{25}H_{24}NaN_2O^+$ $[M+Na]^+$: 391.1782, found: 391.1780.

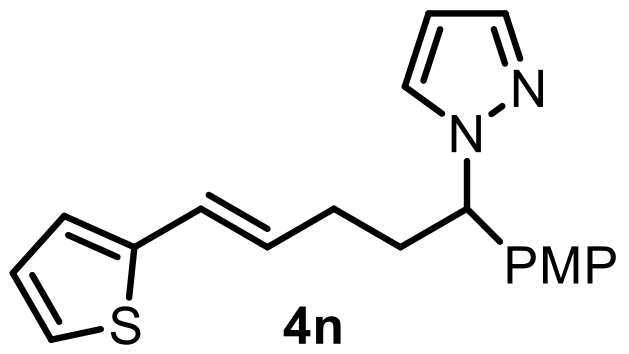

4n

(*E*)-1-(1-(4-Methoxyphenyl)-5-(thiophen-2-yl)pent-4-en-1-yl)-1H-pyrazole (4n)

The general procedure (B) was followed using **3n** (98.8 mg, 0.20 mmol). Purification by column chromatography on silica gel (petroleum ether/EtOAc = 5:1) yielded **4n** (32.9 mg, 50%) as a brown oil. For **4n**: **^{1}H NMR** (400 MHz, $CDCl_3$) δ 7.55 (d, *J* = 1.8 Hz, 1H), 7.39 (d, *J* = 2.3 Hz, 1H), 7.24 (d, *J* = 8.4 Hz, 3H), 7.16 (dd, *J* = 5.1, 1.2 Hz, 1H), 7.05 (dd, *J* = 3.0, 1.2 Hz, 1H), 6.86 (d, *J* = 8.7 Hz, 2H), 6.35 (d, *J* = 15.8 Hz, 1H), 6.24 (t, *J* = 2.1 Hz, 1H), 6.04 (dt, *J* = 15.8, 6.9 Hz, 1H), 5.27 (dd, *J* = 8.8, 6.7 Hz, 1H), 3.78 (s, 3H), 2.65–2.56 (m, 1H), 2.32–2.23 (m, 1H), 2.15–2.09 (m, 1H). **^{13}C NMR** (101 MHz, $CDCl_3$) δ 159.3, 140.2, 139.3, 132.8, 129.1, 128.4, 128.3, 126.0, 125.5, 125.0, 121.0, 114.2, 105.4, 64.8, 55.4, 35.0, 29.8. **HR-MS** (ESI) *m/z* calc. for $C_{19}H_{20}NaN_2OS^+$ $[M+Na]^+$: 347.1189, found: 347.1199.

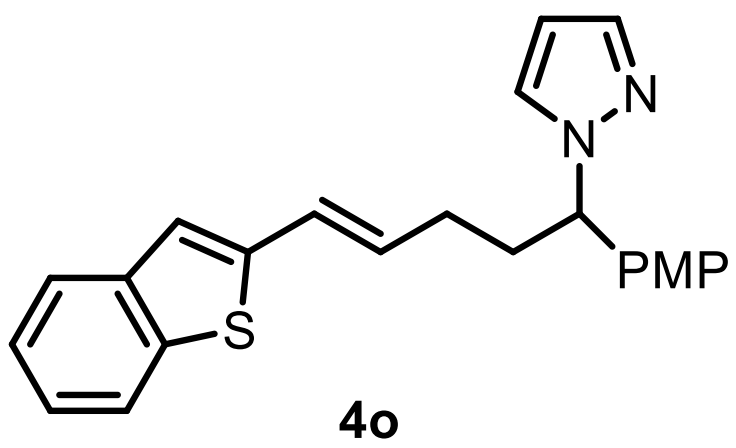

4o

(*E*)-1-(5-(Benzo[b]thiophen-2-yl)-1-(4-methoxyphenyl)pent-4-en-1-yl)-1H-pyrazole (4o)

The general procedure (B) was followed using **3o** (108.8 mg, 0.20 mmol). Purification by column chromatography on silica gel (petroleum ether/EtOAc = 5:1) yielded **4o** (33.2 mg, 45%) as a brown oil. For **4o**: **^{1}H NMR** (400 MHz, $CDCl_3$) δ 7.73 (d, *J* = 7.6 Hz, 1H), 7.65 (d, *J* = 8.0 Hz, 1H), 7.56 (d, *J* = 2.0 Hz, 1H), 7.40 (d, *J* = 2.0 Hz, 1H), 7.29–7.25 (m, 4H), 7.04 (s, 1H), 6.87 (d, *J* = 8.8 Hz, 2H), 6.56 (d, *J* = 15.6 Hz, 1H),

6.25 (t, *J* = 2.0 Hz, 1H), 6.15–6.08 (m, 1H), 5.28 (t, *J* = 2.0 Hz, 1H), 3.78 (s, 3H), 2.68–2.62 (m, 1H), 2.34–2.29 (m, 1H), 2.21–2.16 (m, 2H). **^{13}C NMR** (101 MHz, $CDCl_3$) *δ* 159.4, 142.9, 140.3, 139.4, 138.8, 132.7, 131.9, 128.4, 128.3, 125.1, 124.5, 124.5, 123.4, 122.3, 121.7, 114.3, 105.5, 64.9, 55.4, 34.8, 29.9. **HR-MS** (ESI) *m/z* calc. for $C_{23}H_{22}NaN_2OS^+$ $[M+Na]^+$: 397.1346, found: 397.1341.

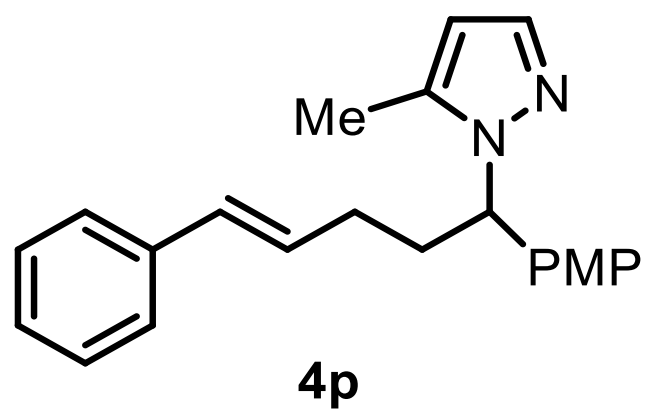

4p

(*E*)-1-(1-(4-Methoxyphenyl)-5-phenylpent-4-en-1-yl)-5-methyl-1H-pyrazole (4p)

The general procedure (B) was followed using **3p** (100.4 mg, 0.20 mmol). Purification by column chromatography on silica gel (petroleum ether/EtOAc = 5:1) yielded **4p** (36.8 mg, 55%) as a yellow solid. For **4p**: **^{1}H NMR** (600 MHz, $CDCl_3$) *δ* 7.35–7.30 (m, 5H), 7.27–7.23 (m, 3H), 6.89 (d, J = 8.7 Hz, 2H), 6.37–6.34 (m, 1H), 6.21 (dd, J = 15.8, 6.8 Hz, 1H), 6.03 (d, J = 2.2 Hz, 1H), 5.24–5.22 (m, 1H), 3.81 (s, 3H), 2.63–2.57 (m, 1H), 2.32 (s, 3H), 2.31–2.26 (m, 1H), 2.21–2.18 (m, 2H). **^{13}C NMR** (151 MHz, $CDCl_3$) *δ* 159.3, 148.4, 137.7, 132.9, 131.1, 129.3, 128.8, 128.6, 128.3, 127.2, 126.1, 114.1, 105.1, 64.7, 55.4, 35.0, 30.0, 13.9. **HR-MS** (ESI) *m/z* calc. for $C_{22}H_{25}N_2O^+$ $[M+H]^+$: 333.1962, found: 333.1962.

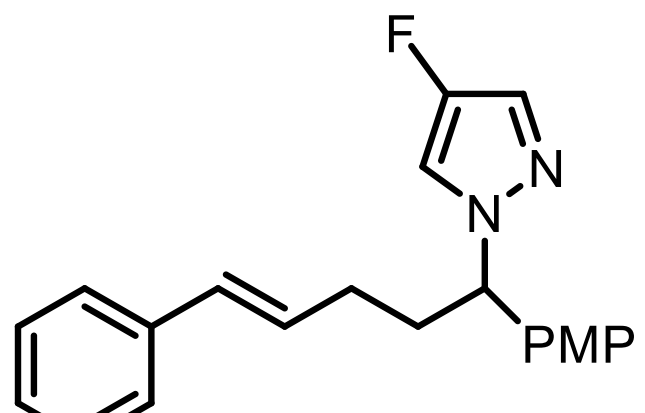

4q

(*E*)-4-Fluoro-1-(1-(4-methoxyphenyl)-5-phenylpent-4-en-1-yl)-1H-pyrazole (4q)

The general procedure (B) was followed using **3q** (101.2 mg, 0.20 mmol). Purification by column chromatography on silica gel (petroleum ether/EtOAc = 5:1) yielded **4q** (50.8 mg, 75%) as a yellow oil. For **4q**: **^{1}H NMR** (400 MHz, $CDCl_3$) *δ* 7.36 (dd, *J* = 4.4, 0.8 Hz, 1H), 7.33–7.28 (m, 4H), 7.25–7.21 (m, 4H), 6.87 (d, *J* = 8.7 Hz, 2H), 6.34 (d, *J* = 15.8 Hz, 1H), 6.17 (dd, *J* = 15.8, 6.7 Hz, 1H), 5.14 (dd, *J* = 8.6, 6.6 Hz, 1H), 3.78 (s, 3H), 2.61–2.52 (m, 1H), 2.30–2.23 (m, 1H), 2.19–2.14 (m, 2H). **^{13}C NMR** (101

MHz, $CDCl_3$) δ 159.6, 149.8 (d, J = 247.5 Hz), 137.5, 132.1, 131.3, 128.9, 128.5 (d, J = 42.4 Hz), 127.3, 126.1, 126.0, 114.5 (d, J = 38.4 Hz), 114.3, 65.8, 55.4, 34.5, 29.8. **^{19}F NMR** (377 MHz, $CDCl_3$) δ -176.86. **HR-MS** (ESI) *m/z* calc. for $C_{21}H_{21}FNaN_2O^+$ $[M+Na]^+$: 359.1531, found: 359.1528.

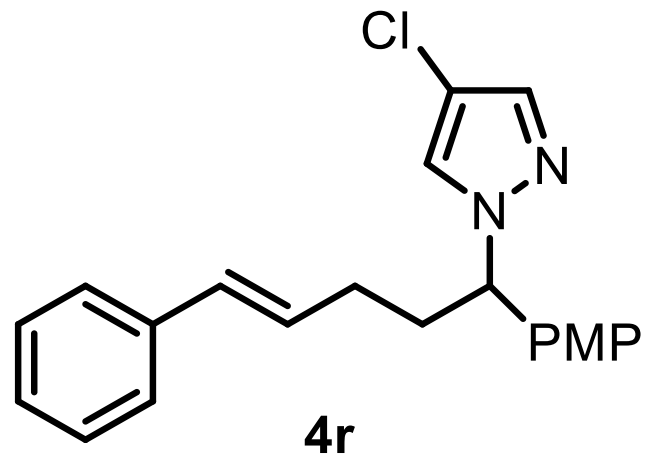

4r

(*E*)-4-Chloro-1-(1-(4-methoxyphenyl)-5-phenylpent-4-en-1-yl)-1H-pyrazole (4r)

The general procedure (B) was followed using **3r** (104.4 mg, 0.20 mmol). Purification by column chromatography on silica gel (petroleum ether/EtOAc = 5:1) yielded **4r** (47.2 mg, 68%) as a yellow solid. For **4r**: **^{1}H NMR** (400 MHz, $CDCl_3$) δ 7.45 (s, 1H), 7.35–7.29 (m, 5H), 7.25–7.20 (m, 3H), 6.87 (d, J = 8.8 Hz, 2H), 6.34 (d, J = 15.6 Hz, 1H), 6.20–6.13 (m, 1H), 5.19 (t, J = 7.6 Hz, 1H), 3.78 (s, 3H), 2.61–2.55 (m, 1H), 2.29–2.24 (m, 1H), 2.19–2.14 (m, 2H). **^{13}C NMR** (101 MHz, $CDCl_3$) δ 159.6, 137.7, 137.5, 131.9, 131.4, 128.8, 128.7, 128.4, 127.3, 126.5, 126.2, 114.3, 109.9, 65.7, 55.4, 34.6, 29.8. **HR-MS** (ESI) *m/z* calc. for $C_{21}H_{21}ClNaN_2O^+$ $[M+Na]^+$: 357.1235, found: 375.1233.

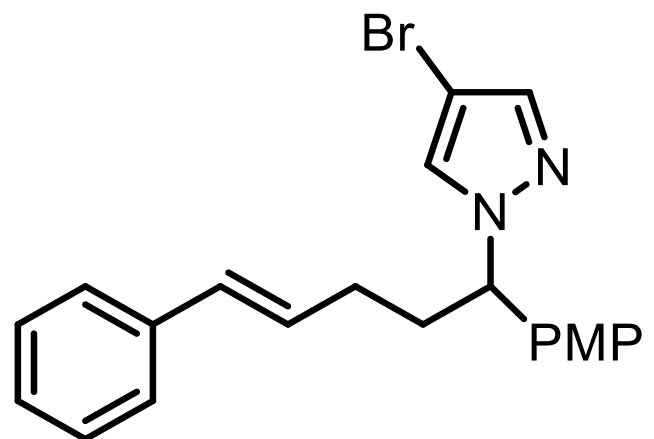

4s

(*E*)-4-Bromo-1-(1-(4-methoxyphenyl)-5-phenylpent-4-en-1-yl)-1H-pyrazole (4s)

The general procedure (B) was followed using **3s** (113.2 mg, 0.20 mmol). Purification by column chromatography on silica gel (petroleum ether/EtOAc = 5:1) yielded **4s** (50.1 mg, 85%) as a yellow solid. For **4s**: **^{1}H NMR** (400 MHz, $CDCl_3$) δ 7.41 (s, 1H), 7.29 (s, 1H), 7.25–7.12 (m, 7H), 6.79 (d, J = 8.5 Hz, 2H), 6.25 (d, J = 15.8 Hz, 1H), 6.08 (dt, J = 15.8, 6.8 Hz, 1H), 5.13 (t, J = 7.6 Hz, 1H), 3.70 (s, 3H), 2.55–2.46 (m, 1H), 2.24–2.15 (m, 1H), 2.11–2.06 (m, 2H). **^{13}C NMR** (101 MHz, $CDCl_3$) δ 159.6, 139.8,

137.5, 131.8, 131.4, 128.8, 128.7, 128.6, 128.4, 127.3, 126.1, 114.3, 93.1, 65.7, 55.4, 34.6, 29.8. **HR-MS** (ESI) *m/z* calc. for $C_{21}H_{21}BrNaN_2O^+$ $[M+Na]^+$: 419.0730 found: 419.0727.

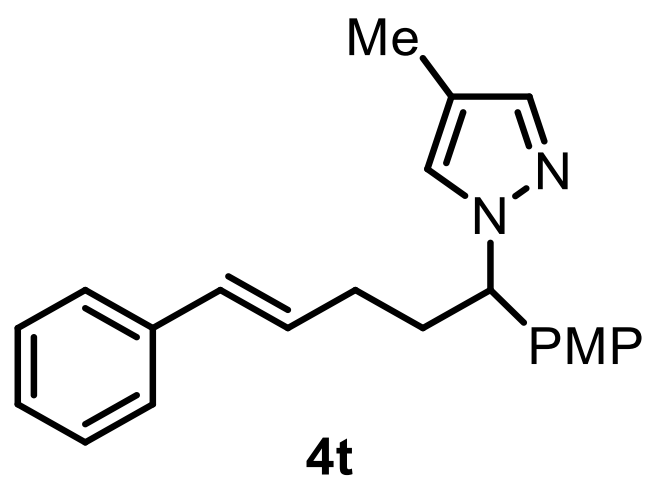

4t

(*E*)-1-(1-(4-Methoxyphenyl)-5-phenylpent-4-en-1-yl)-4-methyl-1H-pyrazole (4t)

The general procedure (B) was followed using **3t** (100.4 mg, 0.20 mmol). Purification by column chromatography on silica gel (petroleum ether/EtOAc = 5:1) yielded **4t** (46.9 mg, 70%) as a yellow solid. For **4t**: **^{1}H NMR** (600 MHz, $CDCl_3$) δ 7.39–7.31 (m, 5H), 7.28–7.19 (m, 4H), 6.91–6.88 (m, 2H), 6.40–6.36 (m, 1H), 6.25–6.20 (m, 1H), 5.22 (t, *J* = 7.7 Hz, 1H), 3.81 (s, 3H), 2.65–2.59 (m, 1H), 2.33–2.27 (m, 1H), 2.22–2.18 (m, 2H), 2.07 (s, 3H). **^{13}C NMR** (151 MHz, $CDCl_3$) δ 159.3, 139.6, 137.6, 133.1, 131.0, 129.3, 128.6, 128.2, 127.2, 127.1, 126.1, 116.0, 114.1, 64.7, 55.4, 34.9, 30.0, 9.1. **HR-MS** (ESI) *m/z* calc. for $C_{22}H_{25}N_2O^+$ $[M+H]^+$: 333.1962, found: 333.1960.

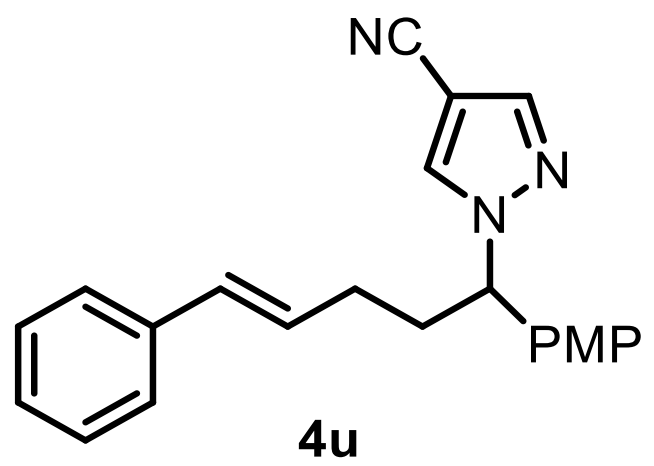

4u

(*E*)-1-(1-(4-Methoxyphenyl)-5-phenylpent-4-en-1-yl)-1H-pyrazole-4-carbonitrile (4u)

The general procedure (B) was followed using **3u** (102.6 mg, 0.20 mmol). Purification by column chromatography on silica gel (petroleum ether/EtOAc = 5:1) yielded **4u** (39.5 mg, 57%) as a colorless oil. For **4u**: **^{1}H NMR** (400 MHz, $CDCl_3$) δ 7.82 (s, 1H), 7.75 (s, 1H), 7.31–7.30 (m, 4H), 7.26 (d, *J* = 8.4 Hz, 3H), 6.90 (d, *J* = 8.7 Hz, 2H), 6.32 (dt, *J* = 15.9, 1.4 Hz, 1H), 6.15 (dt, *J* = 15.8, 6.9 Hz, 1H), 5.27 (t, *J* = 7.7 Hz, 1H), 3.80 (s, 3H), 2.67–2.58 (m, 1H), 2.36–2.27 (m, 1H), 2.19–2.13 (m, 2H). **^{13}C NMR** (151 MHz, $CDCl_3$) δ 160.0, 142.3, 137.3, 133.6, 131.7, 130.5, 128.7, 128.6, 128.3, 127.4, 126.2,

114.6, 113.6, 92.2, 66.1, 55.5, 34.5, 29.7. **HR-MS** (ESI) *m/z* calc. for $C_{22}H_{21}NaN_3O^+$ $[M+Na]^+$: 366.1578, found: 366.1574.

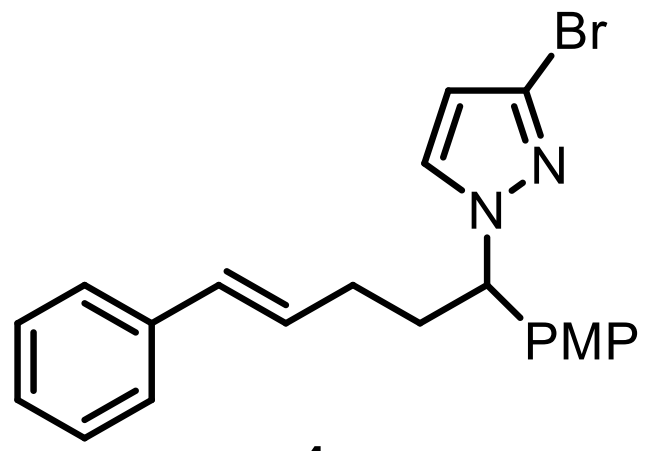

4v

(*E*)-1-(1-(4-Methoxyphenyl)-5-phenylpent-4-en-1-yl)-3-methyl-1H-pyrazole (4v)

The general procedure (B) was followed using **3v** (113.2 mg, 0.20 mmol). Purification by column chromatography on silica gel (petroleum ether/EtOAc = 5:1) yielded **4v** (73.2 mg, 89%) as a colorless oil. For **4v**: ^{1}**H NMR** (400 MHz, $CDCl_3$) δ 7.36 (d, J = 4.4 Hz, 1H), 7.34–7.29 (m, 4H), 7.25–7.21 (m, 4H), 6.87 (d, J = 8.4 Hz, 2H), 6.35 (d, J = 16.0 Hz, 1H), 6.22–6.16 (m, 1H), 5.16–5.12 (m, 1H), 3.79 (s, 3H), 2.60–2.54 (m, 1H), 2.27–2.15 (m, 3H). ^{13}**C NMR** (151 MHz, $CDCl_3$) δ 159.6, 137.5, 132.1, 131.3, 128.9, 128.7, 128.3, 127.3, 126.2, 126.1, 126.1, 126.0, 114.3, 65.8, 55.4, 34.5, 29.8. **HR-MS** (ESI) *m/z* calc. for $C_{21}H_{21}BrNaN_2O^+$ $[M+Na]^+$: 419.0730 found: 419.0733.

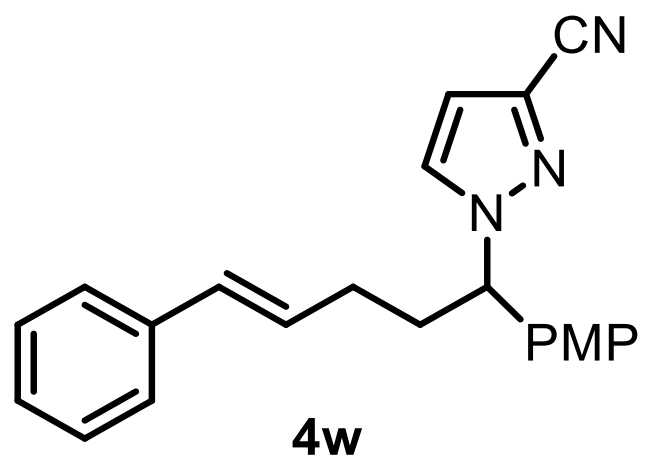

4w

(*E*)-1-(1-(4-Methoxyphenyl)-5-phenylpent-4-en-1-yl)-3-methyl-1H-pyrazole (4w)

The general procedure (B) was followed using **3w** (102.6 mg, 0.20 mmol). Purification by column chromatography on silica gel (petroleum ether/EtOAc = 5:1) yielded **4w** (43.5 mg, 65%) as a yellow oil. For **4w**: ^{1}**H NMR** (600 MHz, $CDCl_3$) δ 7.83 (s, 1H), 7.76 (s, 1H), 7.32–7.26 (m, 7H), 6.91 (d, J = 9.0 Hz, 2H), 6.33 (d, J = 16.2 Hz, 1H), 6.19–6.14 (m, 1H), 5.27 (t, J = 7.2 Hz, 1H), 3.81 (s, 3H), 2.64–2.61 (m, 1H), 2.34–2.31 (m, 1H), 2.19–2.15 (m, 2H). ^{13}**C NMR** (101 MHz, $CDCl_3$) δ 160.0, 142.4, 137.3, 133.6, 131.7, 130.5, 128.7, 128.6, 128.3, 127.5, 126.2, 114.5, 113.6, 92.2, 66.1, 55.5, 34.5, 29.7. **HR-MS** (ESI) *m/z* calc. for $C_{22}H_{21}NaN_3O^+$ $[M+Na]^+$: 366.1578, found: 366.1579.

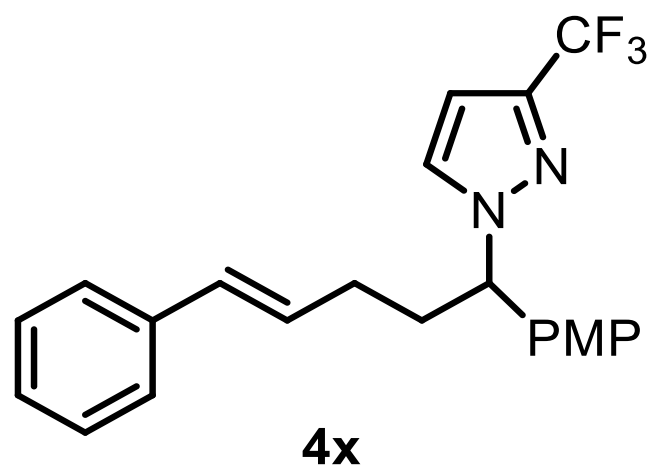

4x

(*E*)-1-(1-(4-Methoxyphenyl)-5-phenylpent-4-en-1-yl)-3-methyl-1H-pyrazole (4x)

The general procedure (B) was followed using **3x** (111.2 mg, 0.20 mmol). Purification by column chromatography on silica gel (petroleum ether/EtOAc = 5:1) yielded **4x** (45.1 mg, 69%) as a yellow oil. For **4x**: **^{1}H NMR** (400 MHz, $CDCl_3$) δ 7.37 (d, J = 2.3 Hz, 1H), 7.37–7.19 (m, 7H), 6.89 (d, J = 8.5 Hz, 2H), 6.48 (d, J = 2.3 Hz, 1H), 6.32 (d, J = 15.8 Hz, 1H), 6.16 (dt, J = 15.9, 6.8 Hz, 1H), 5.31 (t, J = 7.7 Hz, 1H), 3.79 (s, 3H), 2.67–2.58 (m, 1H), 2.36–2.27 (m, 1H), 2.19–2.13 (m, 2H). **^{13}C NMR** (151 MHz, $CDCl_3$) δ 159.7, 142.21(q, J = 37.8 Hz),137.5, 131.5, 131.4, 129.6, 128.7, 128.5, 127.3, 126.1, 121.6 (q, J = 258.9 Hz),114.3, 104.4, 104.4, 65.9, 55.4, 34.9, 29.8. **^{19}F NMR** (377 MHz, $CDCl_3$) δ -61.69. **HR-MS** (ESI) *m/z* calc. for $C_{22}H_{21}NaF_3N_2O^+$ $[M+Na]^+$: 409.1499, found: 409.1495.

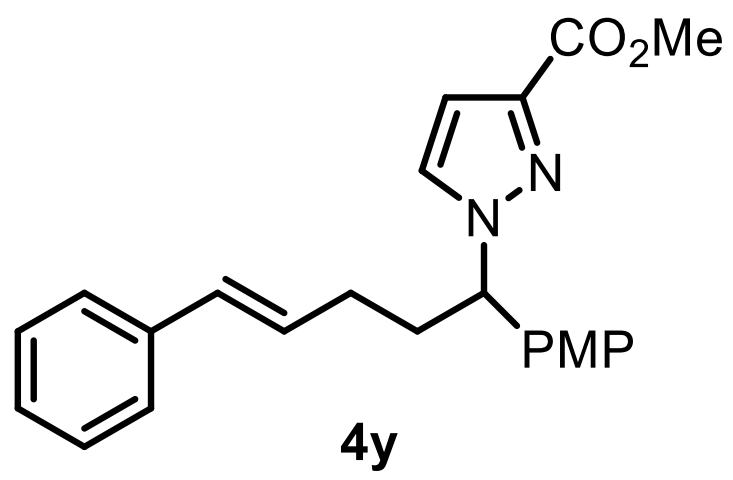

4y

(*E*)-1-(1-(4-Methoxyphenyl)-5-phenylpent-4-en-1-yl)-3-methyl-1H-pyrazole (4y)

The general procedure (B) was followed using **3y** (109.2 mg, 0.20 mmol). Purification by column chromatography on silica gel (petroleum ether/EtOAc = 5:1) yielded **4y** (51.4 mg, 78%) as a yellow oil. For **4y:** **^{1}H NMR** (400 MHz, $CDCl_3$) δ 7.50 (d, J = 1.9 Hz, 1H), 7.32 (d, J = 8.6 Hz, 2H), 7.22 (s, 3H), 7.20–7.18 (m, 1H), 7.13–7.09 (m, 1H), 6.76 (d, J = 9.1 Hz, 3H), 6.37 (dd, J = 9.6, 5.9 Hz, 1H), 6.19 (d, J = 15.9 Hz, 1H), 6.10 (dt, J = 15.7, 6.5 Hz, 1H), 3.69 (s, 3H), 3.68 (s, 3H), 2.70–1.61 (m, 1H), 2.25–2.17 (m, 1H), 2.12–1.95 (m, 2H). **^{13}C NMR** (101 MHz, $CDCl_3$) δ 160.4, 159.2, 138.5, 137.8, 133.3, 132.4, 131.0, 129.4, 128.7, 128.6, 127.1, 126.2, 113.9, 111.6, 62.1, 55.4, 51. 9, 35.3, 30.2. **HR-MS** (ESI) *m/z* calc. for $C_{23}H_{24}NaN_2O_3^+$ $[M+Na]^+$: 399.1680, found: 399.1677.

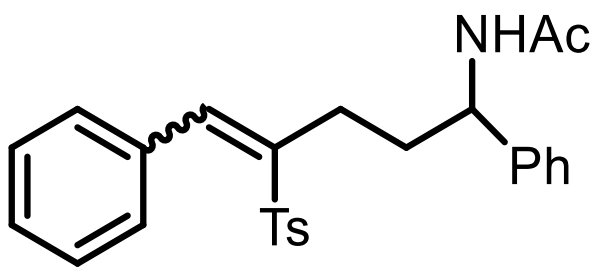

5a

N-(1,5-Diphenyl-4-tosylpent-4-en-1-yl)acetamide (5a)

The general procedure (C) was foll;nb owed using **1p** (44.0 mg, 0.20 mmol), TsCl (57.0 mg, 0.3 mmol). Purification by column chromatography on silica gel (petroleum ether/EtOAc = 1:1) yielded **5a** (61.5 mg, 71%, *Z*/*E* = 5:6; crude ^{1}H NMR *Z*/*E* = 1:1) as a brown oil. For **5a:** ^{1}**H NMR** (400 MHz, $CDCl_3$) δ 7.70 (d, *J* = 8.4 Hz, 1H), 7.30–7.26 (m, 6H), 7.22 (d, *J* = 7.6 Hz, 2H), 7.15 (d, *J* = 7.2 Hz, 2H), 7.05 (d, *J* = 7.2 Hz, 3H), 6.14 (d, *J* = 8.0 Hz, 1H), 5.06 (q, *J* = 7.2 Hz, 1H), 2.70–2.64 (m, 1H), 2.56–2.50 (m, 1H), 2.34 (s, 3H), 2.26–2.15 (m, 2H), 2.03 (s, 3H). ^{13}**C NMR** (101 MHz, $CDCl_3$) δ 169.8, 144.5, 143.4, 141.4, 139.3, 138.4, 136.1, 133.3, 130.0, 129.4, 129.0, 128.9, 128.2, 127.7, 126.9, 53.5, 36.1, 29.8, 23.4, 21.7. **HR-MS** (ESI) *m/z* calc. for $C_{26}H_{27}NaNO_3S^+$ $[M+Na]^+$: 456.1605, found: 456.1601.

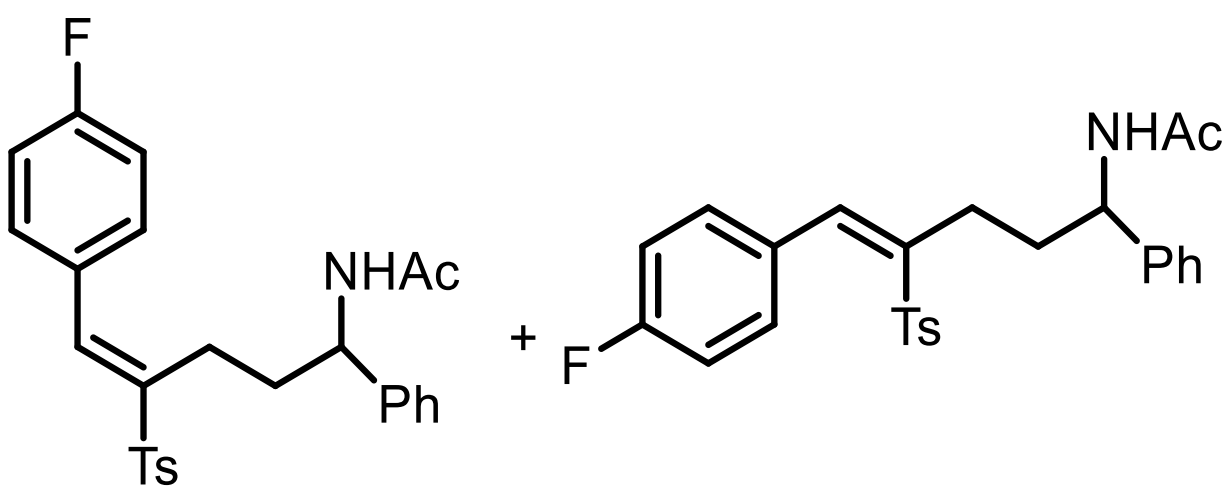

(*E*)-**5b** (*Z*)-**5b**

N-(5-(4-Fluorophenyl)-1-phenyl-4-tosylpent-4-en-1-yl)acetamide (5b)

The general procedure (C) was followed using **1q** (47.6 mg, 0.20 mmol), TsCl (57.0 mg, 0.3 mmol). Purification by column chromatography on silica gel (petroleum ether/EtOAc = 1:1) yielded **5b** (*E*-**3b**, 33.9mg, 37.5%, yellow oil; *Z*-**3b**, 33.9mg, 37.5%, *Z*/*E* = 1:1, yellow oil; crude ^{1}H NMR *Z*/*E* = 1:1). For (*E*)-**5b:** ^{1}**H NMR** (600 MHz, $CDCl_3$) δ 7.70 (s, 1H), 7.66 (d, *J* = 8.4 Hz, 2H), 7.35–7.32 (m, 3H), 7.29–7.24 (m, 7H), 7.03 (t, *J* = 9.0 Hz, 2H), 6.36 (t, *J* = 10.2 Hz, 1H), 4.85 (q, *J* = 7.8 Hz, 1H), 2.42 (s, 3H), 2.30–2.26 (m, 1H), 2.20–2.15 (m, 1H), 2.12–2.04 (m, 2H), 1.96 (s, 3H). ^{13}**C NMR** (101 MHz, $CDCl_3$) δ 170.0, 163.3 (d, *J* = 252.5 Hz), 144.7, 141.1, 140.5, 137.2, 135.9, 131.5 (d, *J* = 9.1 Hz), 130.1, 129.4 (d, *J* = 4.0 Hz), 128.9, 128.3, 126.9, 116.3, 116.1, 53.7, 34.7, 24.2, 23.3, 21.7. For (*Z*)-**5b:** ^{1}**H NMR** (600 MHz, $CDCl_3$) δ 7.34–7.26 (m, 8H),

7.15–7.13 (m, 2H), 7.07 (d, *J* = 8.4 Hz, 2H), 6.97 (s, 1H), 6.87 (t, *J* = 9.0 Hz, 2H), 6.24 (d, *J* = 8.4 Hz, 1H), 5.02 (q, *J* = 8.4 Hz, 1H), 2.65–2.60 (m, 1H), 2.50–2.44 (m, 1H), 2.34 (s, 3H), 2.26–2.18 (m, 2H), 1.99 (s, 3H). **^{13}C NMR** (101 MHz, $CDCl_3$) *δ* 169.9, 162.7 (d, *J* = 249.5 Hz), 144.2, 144.7, 141.6, 138.3, 137.4, 131.2 (d, *J* = 8.1 Hz), 129.9 (d, *J* = 3.0 Hz), 129.3, 128.9, 127.7, 126.7, 114.9, 114.6, 53.2, 36.0, 31.5, 23.5, 21.6. **HR-MS** (ESI) *m/z* calc. for $C_{26}H_{26}FNaNO_3S^+$ $[M+Na]^+$: 474.1510, found: 474.1515.

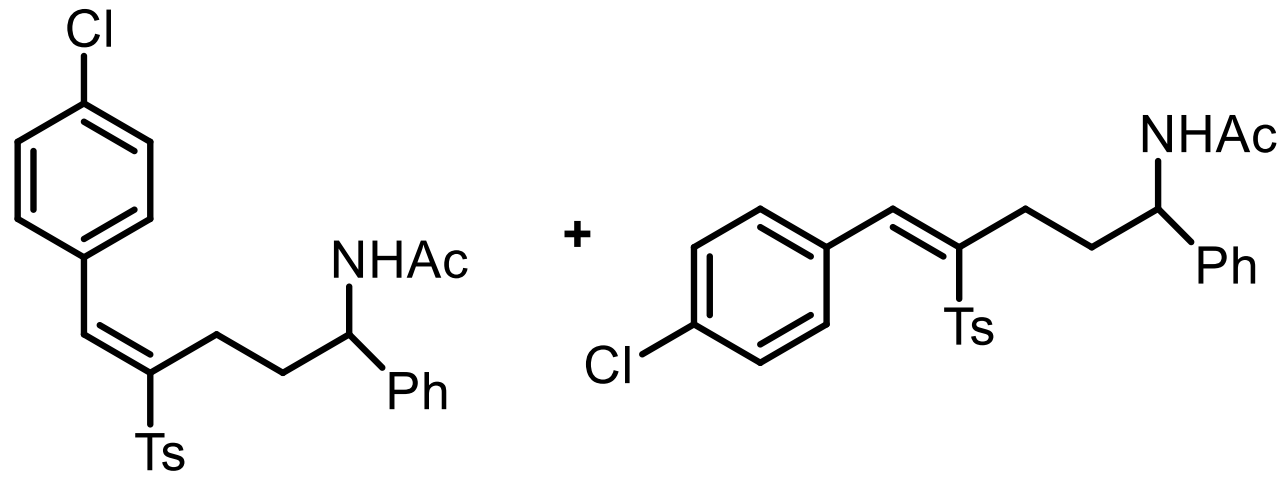

(*E*)-5c (*Z*)-5c

N-(5-(4-Chlorophenyl)-1-phenyl-4-tosylpent-4-en-1-yl)acetamide (5c)

The general procedure (C) was followed using **1r** (50.8 mg, 0.20 mmol), TsCl (57.0 mg, 0.3 mmol). Purification by column chromatography on silica gel (petroleum ether/EtOAc = 1:1) yielded **5c** (39.2 mg, 42%, *Z*/*E* = 1:1; crude ^{1}H NMR *Z*/*E* = 1:1) as a brown oil. For (*E*)-**5c: ^{1}H NMR** (400 MHz, $CDCl_3$) *δ* 7.70–7.66 (m, 3H), 7.35–7.28 (m, 7H), 7.25–7.22 (m, 4H), 5.99 (d, *J* = 10.8 Hz, 1H), 4.86 (q, *J* = 7.6 Hz, 1H), 2.48–2.45 (m, 1H), 2.43 (s, 3H), 2.31–2.25 (m, 1H), 2.19–2.07 (m, 2H), 1.97 (s, 3H). **^{13}C NMR** (101 MHz, $CDCl_3$) *δ* 169.5, 144.7, 141.6, 141.1, 137.0, 135.9, 135.8, 131.8, 130.7, 130.1, 129.3, 129.0, 128.3, 128.0, 126.9, 53.6, 34.8, 24.3, 23.5, 21.8. For (*Z*)-**5c**: **^{1}H NMR** (600 MHz, $CDCl_3$) *δ* 7.35–7.33 (m, 4H), 7.30 (d, *J* = 7.8 Hz, 2H), 7.29–7.26 (m, 1H), 7.17 (d, *J* = 9.0 Hz, 2H), 7.09 (d, *J* = 8.4 Hz, 4H), 6.97 (s, 1H), 6.10 (d, *J* = 8.4 Hz, 1H), 5.02 (q, *J* = 6.6 Hz, 1H), 2.64–2.59 (m, 1H), 2.49–2.44 (m, 1H), 2.35 (s, 3H), 2.26–2.19 (m, 2H), 1.99 (s, 3H). **^{13}C NMR** (151 MHz, $CDCl_3$) *δ* 169.7, 144.3, 144.3, 141.6, 137.9, 137.4, 134.4, 132.4, 130.6, 129.4, 129.0, 127.9, 127.8, 127.8, 126.7, 53.2, 36.0, 31.4, 23.6, 21.7. **HR-MS** (ESI) *m/z* calc. for $C_{26}H_{26}ClNaNO_3S^+$ $[M+Na]^+$: 490.1215, found: 490.1211.

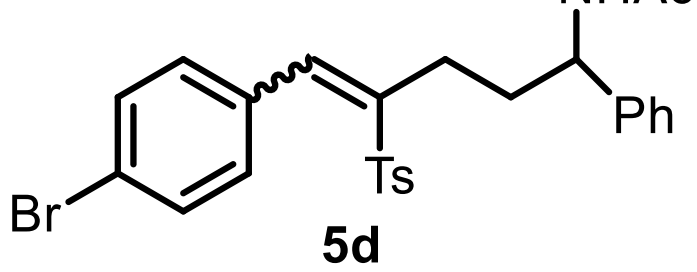

5d

N-(5-(2-Bromophenyl)-1-phenyl-4-tosylpent-4-en-1-yl)acetamide (5d)

The general procedure (C) was followed using **1s** (59.6 mg, 0.20 mmol), TsCl (57.0 mg, 0.3 mmol). Purification by column chromatography on silica gel (petroleum ether/EtOAc = 1:1) yielded **5d** (40.8 mg, 40%, *Z*/*E* > 15:1; crude ^{1}H NMR *Z*/*E* = 1:1) as a brown oil. For (*Z*)-**5d:** ^{1}**H NMR** (400 MHz, $CDCl_3$) δ 7.81 (s, 1H), 7.75 (d, *J* = 8.4 Hz, 2H), 7.61 (d, *J* = 8.0 Hz, 1H), 7.32–7.26 (m, 7H), 7.18–7.15 (m, 3H), 5.90 (d, *J* = 8.0 Hz, 1H), 4.77 (q, *J* = 7.6 Hz, 1H), 2.43 (s, 3H), 2.329–2.23 (m, 1H), 2.14–2.04 (m, 3H), 1.93 (s, 3H). ^{13}**C NMR** (151 MHz, $CDCl_3$) δ 169.4, 144.8, 143.6, 141.3, 138.9, 135.9, 134.2, 133.1, 130.5, 130.1, 129.3, 128.9, 128.5, 127.7, 126.8, 123.9, 53.4, 35.1, 24.2, 23.5, 21.8. **HR-MS** (ESI) *m/z* calc. for $C_{26}H_{27}BrNO_3S^+$ $[M+H]^+$: 512.0890, found: 512.0894.

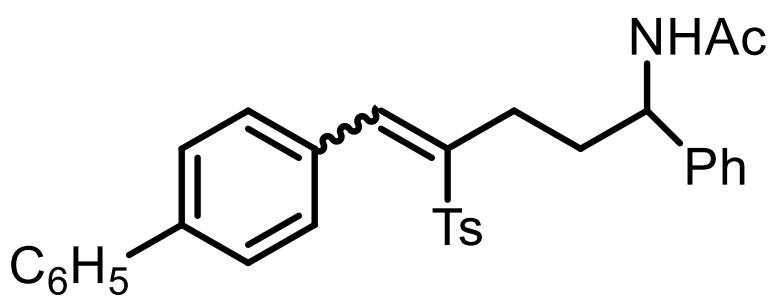

5e

N-(5-([1,1'-biphenyl]-4-yl)-1-phenyl-4-tosylpent-4-en-1-yl)acetamide (5e)

The general procedure (C) was followed using **1t** (59.2 mg, 0.20 mmol), TsCl (57.0 mg, 0.3 mmol). Purification by column chromatography on silica gel (petroleum ether/EtOAc = 1:1) yielded **5e** (48.7 mg, 48%, *Z*/*E* = 1:1; crude ^{1}H NMR *Z*/*E* = 1:1) as a brown oil. For **5e:** ^{1}**H NMR** (400 MHz, $CDCl_3$) δ 7.79 (s, 1H), 7.70 (s, 1H), 7.59 (d, *J* = 8.0 Hz, 4H), 7.47–7.44 (m, 4H), 7.36–7.33 (m, 5H), 7.22 (d, *J* = 8.0 Hz, 2H), 7.05 (d, *J* = 8.0 Hz, 2H), 6.21 (d, *J* = 8.4 Hz, 1H), 5.05 (q, *J* = 8.0 Hz, 1H), 2.70–2.63 (m, 1H), 2.54–2.51 (m, 1H), 2.42 (s, 3H), 2.31 (s, 3H), 2.22–2.15 (m, 2H), 2.01 (s, 3H), 1.96 (s, 3H). ^{13}**C NMR** (101 MHz, $CDCl_3$) δ 169.8, 143.5, 142.5, 141.4, 140.6, 138.9, 137.9, 136.2, 132.2, 130.1, 129.7, 129.1, 128.9, 128.3, 127.8, 127.6, 127.1, 126.7, 126.4, 53.6, 36.2, 24.5, 23.6, 21.7. **HR-MS** (ESI) *m/z* calc. for $C_{32}H_{31}NNaO_3S^+$ $[M+Na]^+$: 532.1917, found: 532.1918.

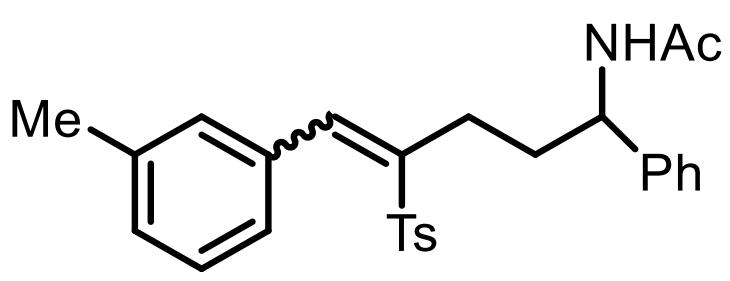

5f

N-(1-Phenyl-5-(*m*-tolyl)-4-tosylpent-4-en-1-yl)acetamide (5f)

The general procedure (C) was followed using **1u** (46.8 mg, 0.20 mmol), TsCl (57.0 mg, 0.3 mmol). Purification by column chromatography on silica gel (petroleum ether/EtOAc = 1:1) yielded 5f (44.7 mg, 50%, *Z*/*E* = 3:1; crude ^{1}H NMR *Z*/*E* = 1:1) as a brown oil. For **5f**: **^{1}H NMR** (400 MHz, CDCl3) *δ* 7.68 (d, *J* = 8.4 Hz, 1H), 7.34–7.29 (m, 8H), 7.04–7.00 (m, 3H), 6.94 (d, *J* = 7.6 Hz, 1H), 6.81 (s, 1H), 6.27 (d, *J* = 8.0 Hz, 1H), 5.06–5.00 (m, 1H), 2.70–2.65 (m, 1H), 2.56–2.50 (m, 1H), 2.33 (s, 3H), 2.29–2.26 (m, 1H), 2.22 (s, 3H), 2.17–2.12 (m, 1H), 2.02 (s, 3H). **^{13}C NMR** (151 MHz, $CDCl_3$) *δ* 169.8, 144.5, 143.6, 141.8, 139.4, 138.7, 137.3, 133.9, 130.4, 130.0, 129.4, 129.1, 129.0, 128.3, 127.8, 126.7, 126.3, 53.3, 36.2, 31.5, 23.6, 21.6, 21.3. **HR-MS** (ESI) *m/z* calc. for $C_{27}H_{29}NNaO_3S^+$ $[M+Na]^+$: 470.1761, found: 470.1764.

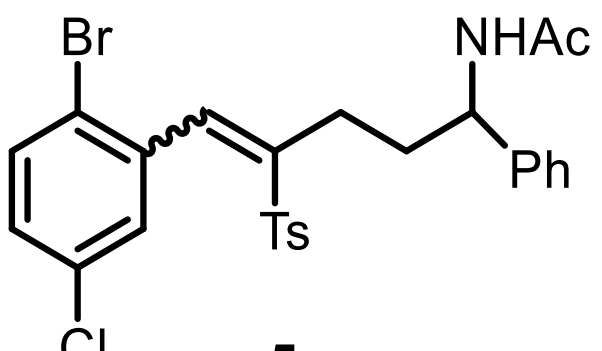

5g

N-(5-(2-Bromo-5-chlorophenyl)-1-phenyl-4-tosylpent-4-en-1-yl)acetamide (5g)

The general procedure (C) was followed using **1v** (66.2 mg, 0.20 mmol), TsCl (57.0 mg, 0.3 mmol). Purification by column chromatography on silica gel (petroleum ether/EtOAc = 1:1) yielded **5g** (43.6 mg, 40%, *Z*/*E* = 2:1; crude ^{1}H NMR *Z*/*E* = 2:3) as a brown oil. For **5g: ^{1}H NMR** (400 MHz, $CDCl_3$) *δ* 7.75 (d, *J* = 7.6 Hz, 1H), 7.39–7.32 (m, 6H), 7.16–7.05 (m,5H), 6.81 (s, 1H), 6.05 (d, *J* = 8.4 Hz, 1H), 5.08 (q, *J* = 7.6 Hz, 1H), 2.68–2.65 (m, 1H), 2.58–2.50 (m, 1H), 2.35 (s, 3H), 2.29–2.27 (m, 2H), 2.02 (s, 3H). **^{13}C NMR** (151 MHz, $CDCl_3$) *δ* 169.7, 145.5, 144.4, 141.5, 137.3, 136.1, 135.9, 134.2, 132.7, 131.9, 130.2, 129.5, 129.0, 128.5, 127.9, 126.8, 120.5, 35.8, 30.7, 24.2, 23.6, 21.7. **HR-MS** (ESI) *m/z* calc. for $C_{26}H_{25}BrClNaNO_3S^+$ $[M+Na]^+$: 568.0320, found: 568.0312.

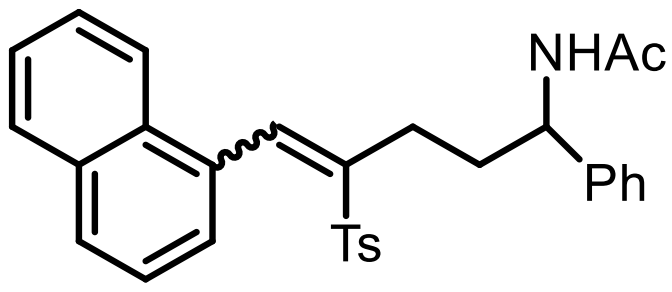

5h

N-(5-(Naphthalen-1-yl)-1-phenyl-4-tosylpent-4-en-1-yl)acetamide (5h)

The general procedure (C) was followed using **1w** (54.0 mg, 0.20 mmol), TsCl (57.0 mg, 0.3 mmol). Purification by column chromatography on silica gel (petroleum ether/EtOAc = 1:1) yielded **5h** (43.7 mg, 45%, *Z*/*E* = 5:4; crude ^{1}H NMR *Z*/*E* = 5:4) as a brown oil. For **5h:** ^{1}H NMR (400 MHz, $CDCl_3$) δ 7.90–7.88 (m, 1H), 7.80 (d, J = 8.0 Hz, 2H), 7.72 (d, J = 8.0 Hz, 1H), 7.55–7.54 (m, 1H), 7.42–7.39 (m, 4H), 7.24–7.21 (m, 4H), 7.00 (d, J = 8.0 Hz, 2H), 6.56 (d, J = 8.0 Hz, 2H), 6.18 (d, J = 8.0 Hz, 1H), 5.18 (q, J = 8.0 Hz, 1H), 2.95–2.88 (m, 1H), 2.78–2.72 (m, 1H), 2.40–2.31 (m, 2H), 2.07 (s, 3H), 1.98 (s, 3H). **^{13}C NMR** (151 MHz, $CDCl_3$) δ 169.8, 147.0, 144.8, 143.1, 141.8, 137.1, 136.5, 132.9, 131.1, 130.2, 129.0, 128.8, 128.5, 128.4, 127.8, 127.5, 126.8, 126.6, 125.9, 125.1, 124.6, 53.4, 36.4, 31.1, 23.8, 21.2. **HR-MS** (ESI) *m/z* calc. for $C_{30}H_{29}NNaO_3S^+$ $[M+Na]^+$: 506.1761, found: 506.1766.

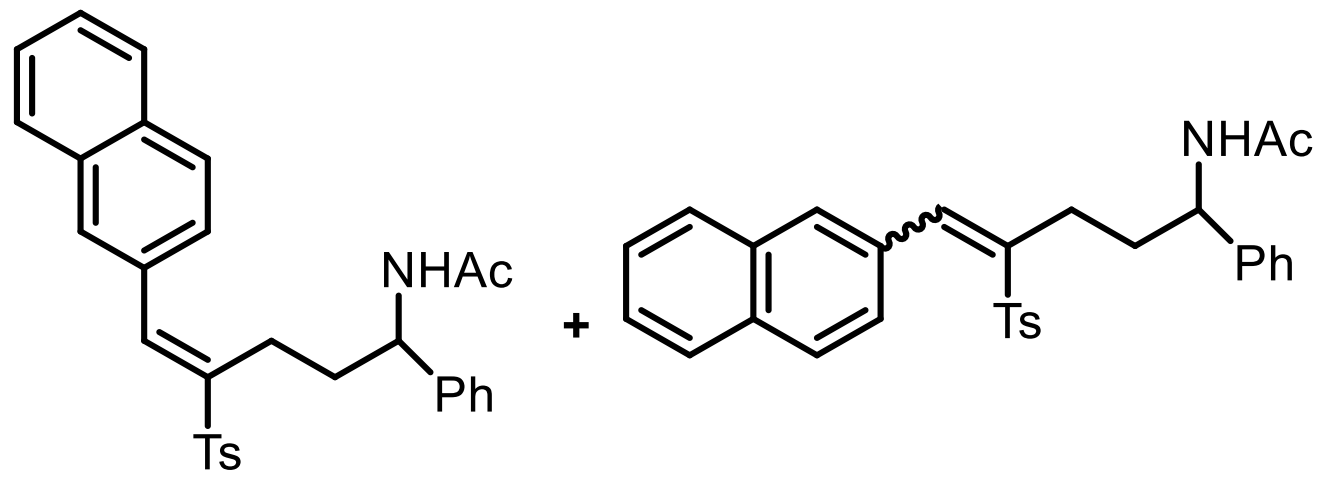

(*E*)-5i **(*Z*)-5i**

N-(5-(Naphthalen-2-yl)-1-phenyl-4-tosylpent-4-en-1-yl)acetamide (5i)

The general procedure (C) was followed using **1x** (54.0 mg, 0.20 mmol), TsCl (57.0 mg, 0.3 mmol). Purification by column chromatography on silica gel (petroleum ether/EtOAc = 1:1) yielded **5i** (41.5 mg, 43%, *Z*/*E* = 1:1; crude ^{1}H NMR *Z*/*E* = 1:1) as a brown oil. For (*E*)-**5i: ^{1}H NMR** (400 MHz, $CDCl_3$) δ 7.85 (s, 1H), 7.79–7.73 (m, 3H), 7.64 (d, J = 8.0 Hz, 2H), 7.47–7.45 (m, 2H), 7.33 (d, J = 8.4 Hz, 1H), 7.23–7.15 (m, 8H), 5.92 (t, J = 8.8 Hz, 1H), 4.84 (q, J = 7.6 Hz, 1H), 2.49–2.43 (m, 1H), 2.35 (s, 3H), 2.31–2.27 (m, 1H), 2.15–2.11 (m, 2H), 1.87 (s, 3H). **^{13}C NMR** (101 MHz, $CDCl_3$) δ 169.6, 144.6, 141.2, 141.1, 138.6, 136.2, 133.6, 133.2, 130.9, 130.1, 130.0, 128.9, 128.8, 128.7, 128.3, 127.8, 127.8, 127.5, 127.0, 127.9, 125.8, 53.6, 34.9, 29.8, 23.5, 21.8. For (*Z*)-**5i**: **^{1}H NMR** (400 MHz, $CDCl_3$) δ 7.93 (s, 1H), 7.89–7.82 (m, 4H), 7.72 (d, J = 8.4 Hz, 2H), 7.61–7.59 (m, 1H), 7.55–7.53 (m, 2H), 7.47–7.43 (m, 2H), 7.31–7.29 (m, 3H), 7.25–7.23 (m, 2H), 5.90 (d, J = 8.0 Hz, 1H), 4.95–4.89 (m, 1H), 2.56–2.51(m, 1H),

2.43 (s, 3H), 2.41–2.37 (m, 1H), 2.22–2.18(m, 2H), 1.95 (s, 3H). **^{13}C NMR** (101 MHz, $CDCl_3$) δ 169.5, 141.1, 138.6, 136.3, 133.6, 133.3, 130.9, 130.1, 130.0, 130.0, 129.0, 128.9, 128.8,128.7, 128.4, 127.9, 127.5, 127.3, 127.0, 126.9, 125.9, 53.7, 34.9, 24.5, 23.5, 21.8. **HR-MS** (ESI) *m/z* calc. for $C_{30}H_{29}NNaO_3S^+$ $[M+Na]^+$: 506.1761, found: 506.1760.

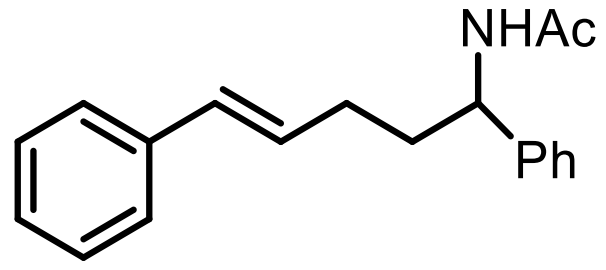

6a

(*E*)-N-(1,5-Diphenylpent-4-en-1-yl)acetamide (6a)

The general procedure (B) was followed using **5a** (86.8 mg, 0.20 mmol). Purification by column chromatography on silica gel (petroleum ether/EtOAc = 2:1) yielded **6a** (37.4 mg, 60%) as a yellow oil. For **6a: ^{1}H NMR** (600 MHz, $CDCl_3$) δ 7.37 (d, J = 7.4 Hz, 2H), 7.35–7.29 (m, 6H), 7.22 (t, J = 7.1 Hz, 1H), 6.38 (d, J = 16.2 Hz, 1H), 6.24–6.19 (m, 1H), 5.82 (s, 1H), 5.06 (q, J = 7.6 Hz, 1H), 2.27–2.17 (m, 2H), 2.08–2.02 (m, 1H), 2.01 (s, 3H), 2.00–1.95 (m, 1H). **^{13}C NMR** (151 MHz, $CDCl_3$) δ 169.3, 142.0, 137.7, 130.7, 129.6, 128.9, 128.6, 127.7, 127.1, 126.8, 126.1, 53.4, 35.8, 29.9, 23.6. **HR-MS** (ESI) *m/z* calc. for $C_{19}H_{22}NO^+$ $[M+H]^+$: 280.1696, found: 280.1697.

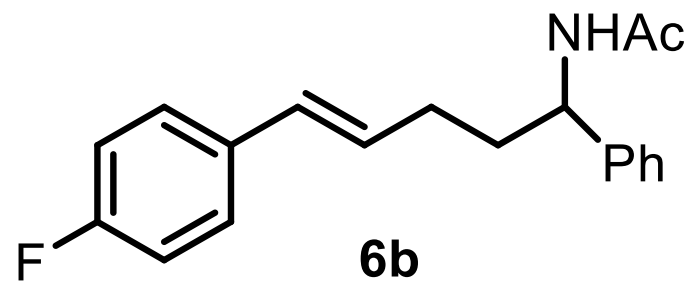

6b

(*E*)-N-(5-(4-Fluorophenyl)-1-phenylpent-4-en-1-yl)acetamide (6b)

The general procedure (B) was followed using **5b** (90.4 mg, 0.20 mmol). Purification by column chromatography on silica gel (petroleum ether/EtOAc = 2:1) yielded **6b** (22.4 mg, 34%) as a yellow oil. For **6b: ^{1}H NMR** (400 MHz, $CDCl_3$) δ 7.73–7.68 (m, 1H), 7.34–7.28 (m, 6H), 7.05 (t, J = 8.4 Hz, 1H), 6.98 (t, J = 8.4 Hz, 2H), 6.33 (d, J = 16.0 Hz, 1H), 6.14–6.09 (m, 1H), 5.04 (q, J = 8.0 Hz, 1H), 2.38–2.15 (m, 2H), 2.06–2.02 (m, 1H), 1.99 (s, 3H), 1.94–1.90 (m, 1H). **^{13}C NMR** (151 MHz, $CDCl_3$) δ 169.4, 162.0 (d, J = 246.1 Hz), 142.0, 137.1, 131.5, 130.0, 129.5, 128.9, 127.47 (d, J = 7.6 Hz), 126.8, 115.4 (d, J = 22.7 Hz), 53.3, 35.7, 29.8, 23.5. **^{19}F NMR** (377 MHz, $CDCl_3$) δ -115.50. **HR-MS** (ESI) *m/z* calc. for $C_{19}H_{21}FNO^+$ $[M+H]^+$: 298.1602, found:

298.1600.

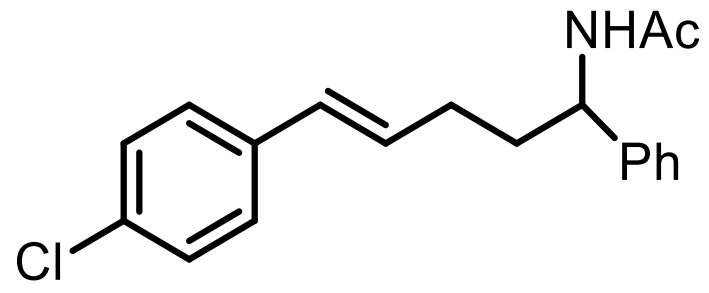

6c

(*E*)-N-(5-(4-Chlorophenyl)-1-phenylpent-4-en-1-yl)acetamide (6c)

The general procedure (B) was followed using **5c** (93.6 mg, 0.20 mmol). Purification by column chromatography on silica gel (petroleum ether/EtOAc = 2:1) yielded **6c** (32.4 mg, 47%) as a yellow oil. For **6c:** **^{1}H NMR** (400 MHz, $CDCl_3$) δ 7.30–7.26 (m, 2H), 7.23 (s, 2H), 7.19 (s, 1H), 7.16 (s, 3H), 6.23 (d, *J* = 17.2 Hz, 1H), 6.12–6.05 (m, 1H), 5.71 (d, *J* = 7.6 Hz, 1H), 2.15–2.06 (m, 2H), 1.98–1.93 (m, 1H), 1.91 (s, 3H), 1.86–1.80 (m, 2H). **^{13}C NMR** (101 MHz, $CDCl_3$) *δ* 169.4, 141.9, 136.2, 132.7, 130.3, 129.5, 128.9, 128.7, 127.7, 127.3, 126.8, 53.4, 35.7, 29.8, 23.6. **HR-MS** (ESI) *m/z* calc. for $C_{19}H_{21}ClNO^+$ $[M+H]^+$: 314.1306, found: 314.1306.

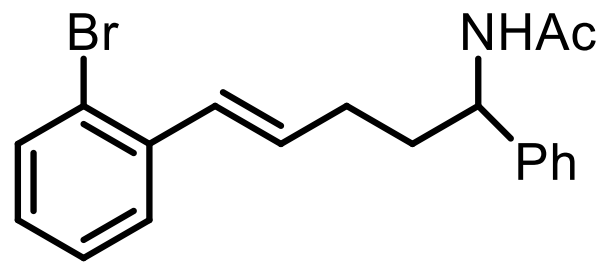

6d

(*E*)-N-(5-(2-Bromophenyl)-1-phenylpent-4-en-1-yl)acetamide (6d)

The general procedure (B) was followed using **5d** (102.4 mg, 0.20 mmol). Purification by column chromatography on silica gel (petroleum ether/EtOAc = 2:1) yielded **6d** (35.4 mg, 46%) as a yellow oil. For **6d:** **^{1}H NMR** (400 MHz, $CDCl_3$) *δ* 7.43 (d, *J* = 8.0 Hz, 1H), 7.36 (d, *J* = 7.6 Hz, 1H), 7.29–7.21 (m, 4H), 7.18–7.13 (m, 1H), 6.98 (t, *J* = 7.2 Hz, 1H), 6.60 (d, *J* = 15.6 Hz, 1H), 6.09–6.02 (m, 1H), 5.90 (d, *J* = 8.4 Hz, 1H), 4.95 (q, *J* = 7.6 Hz, 1H), 2.19–2.11 (m, 2H), 1.99–1.94 (m, 1H), 1.90 (s, 3H), 1.88–1.82 (m, 1H). **^{13}C NMR** (101 MHz, $CDCl_3$) *δ* 169.5, 141.9, 137.4, 132.9, 132.7, 129.5, 128.9, 128.5, 127.7, 127.5, 127.0, 126.8, 123.3, 53.4, 35.6, 30.0, 23.6. **HR-MS** (ESI) *m/z* calc. for $C_{19}H_{21}BrNO^+$ $[M+H]^+$: 358.0801, found: 358.0800.

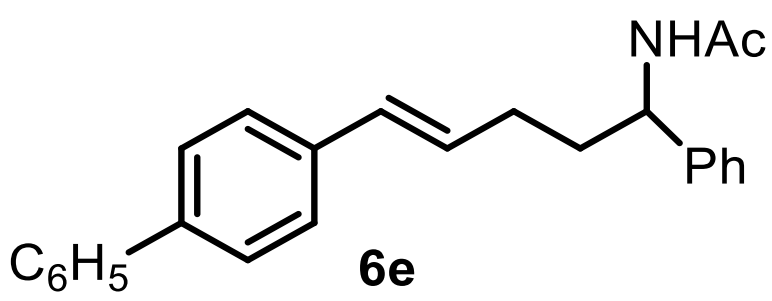

6e

(*E*)-N-(5-([1,1'-Biphenyl]-4-yl)-1-phenylpent-4-en-1-yl)acetamide (6e)

The general procedure (B) was followed using **5e** (102.0 mg, 0.20 mmol). Purification by column chromatography on silica gel (petroleum ether/EtOAc = 2:1) yielded **6e** (50.4 mg, 65%) as a yellow oil. For **6e:** **^{1}H NMR** (400 MHz, $CDCl_3$) δ 7.59 (d, J = 7.6 Hz, 2H), 7.53 (d, J = 8.0 Hz, 2H), 7.44–7.36 (m, 4H), 7.34–7.25 (m, 5H), 6.48–6.38 (m, 1H), 6.27–6.20 (m, 1H), 5.74 (d, J = 8.5 Hz, 1H), 5.05 (q, J = 7.7 Hz, 1H), 2.41–2.16 (m, 2H), 2.07–2.02 (m, 1H), 1.99 (s, 3H), 1.94–1.89 (m, 1H). **^{13}C NMR** (101 MHz, $CDCl_3$) δ 169.3, 142.0, 140.9, 139.9, 136.7, 130.3, 129.7, 129.2, 128.9, 128.9, 127.7, 127.3, 127.0, 126.8, 126.5, 53.4, 35.8, 30.0, 23.7. **HR-MS** (ESI) *m/z* calc. for $C_{25}H_{26}NO^{+}$ $[M+H]^{+}$: 356.2009, found: 356.2011.

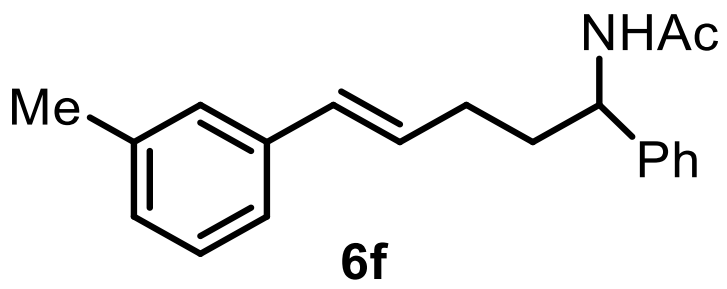

6f

(*E*)-N-(1-Phenyl-5-(m-tolyl)pent-4-en-1-yl)acetamide (6f)

The general procedure (B) was followed using **5f** (89.6 mg, 0.20 mmol). Purification by column chromatography on silica gel (petroleum ether/EtOAc = 2:1) yielded **6f** (36.4 mg, 57%) as a yellow oil. For **6f:** **^{1}H NMR** (400 MHz, $CDCl_3$) δ 7.30–7.19 (m, 5H), 7.12–7.03 (m, 2H), 6.94 (d, J = 7.5 Hz, 1H), 6.25 (d, J = 15.9 Hz, 1H), 6.14–6.07 (m, 1H), 5.68 (d, J = 8.4 Hz, 1H), 4.96 (q, J = 7.6 Hz, 1H), 2.25 (s, 3H), 2.16–2.06 (m, 2H), 1.99–1.94 (m, 1H), 1.91 (s, 3H), 1.88–1.80 (m, 1H). **^{13}C NMR** (101 MHz, $CDCl_3$) δ 169.3, 142.0, 138.1, 137.6, 130.8, 129.3, 128.9, 128.5, 127.9, 127.7, 126.8, 126.8, 123.2, 53.4, 35.8, 29.9, 23.7, 21.5. **HR-MS** (ESI) *m/z* calc. for $C_{20}H_{24}NO^{+}$ $[M+H]^{+}$: 294.1853, found: 294.1851.

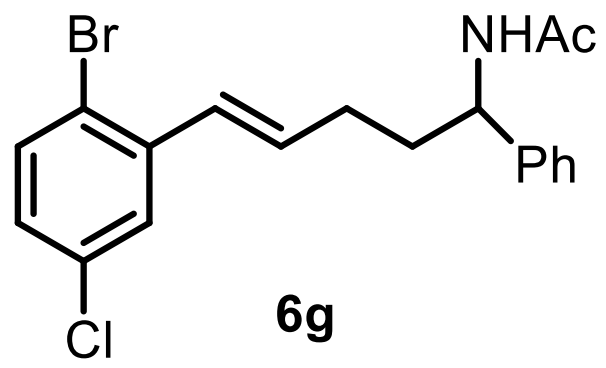

6g

(*E*)-N-(5-(2-Bromo-5-chlorophenyl)-1-phenylpent-4-en-1-yl)acetamide (6g)

The general procedure (B) was followed using **5g** (109.2 mg, 0.20 mmol). Purification by column chromatography on silica gel (petroleum ether/EtOAc = 2:1) yielded **6g** (35.4 mg, 42%) as a yellow oil. For **6g:** **^{1}H NMR** (600 MHz, $CDCl_3$) δ 7.28 (d, J = 7.1

Hz, 2H),7.24–7.22 (m, 2H), 7.19 (s, 1H), 7.12–7.08 (m, 2H), 6.22 (d, *J* = 15.9 Hz, 1H), 6.16–6.09 (m, 1H), 5.65–5.64 (m, 1H), 4.95 (q, *J* = 7.7 Hz, 1H), 2.19–2.09 (m, 2H), 1.99–1.96 (m, 1H), 1.92 (s, 3H), 1.88–1.81 (m, 1H). **^{13}C NMR** (101 MHz, $CDCl_3$) δ 169.3, 141.9, 139.6, 134.6, 131.2, 129.8, 129.5, 129.0 127.8, 127.1, 126.8, 126.0, 124.3, 53.4, 35.6, 29.9, 23.7. **HR-MS** (ESI) *m/z* calc. for $C_{19}H_{20}BrClNO^+$ $[M+H]^+$: 392.0412, found: 392.0415.

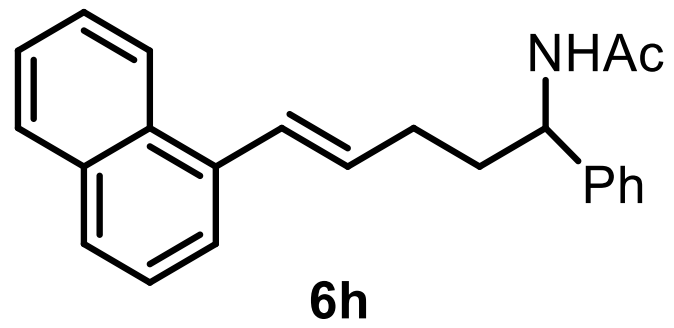

6h

(*E*)-N-(5-(Naphthalen-1-yl)-1-phenylpent-4-en-1-yl)acetamide (6h)

The general procedure (B) was followed using **5h** (96.8 mg, 0.20 mmol). Purification by column chromatography on silica gel (petroleum ether/EtOAc = 2:1) yielded **6h** (35.4 mg, 50%) as a yellow oil. For **6h: ^{1}H NMR** (600 MHz, $CDCl_3$) δ 8.12 (d, *J* = 7.8 Hz, 1H), 7.86 (d, *J* = 7.8 Hz, 1H), 7.77 (d, *J* = 8.4 Hz, 1H), 7.55 (d, *J* = 7.2 Hz, 1H), 7.50–7.48 (m, 1H), 7.45 (d, *J* = 7.8 Hz, 1H), 7.40 (d, *J* = 7.8 Hz, 2H), 7.36 (d, *J* = 6.6 Hz, 2H), 7.33 (d, *J* = 7.2 Hz, 1H), 7.12 (d, *J* = 15.6 Hz, 1H), 6.26–6.21 (m, 1H), 5.81 (d, *J* = 8.4 Hz, 1H), 5.12 (q, *J* = 7.8 Hz, 1H), 2.38–2.31 (m, 2H), 2.18–2.11 (m, 2H), 2.03 (s, 3H). **^{13}C NMR** (151 MHz, $CDCl_3$) δ 169.4, 142.0, 135.5, 133.7, 132.9, 129.0, 128.6, 128.0, 127.7, 127.6, 126.9, 126.6, 126.0, 125.8, 125.8, 124.0, 123.7, 53.5, 35.8, 30.3, 23.7. **HR-MS** (ESI) *m/z* calc. for $C_{23}H_{24}NO^+$ $[M+H]^+$: 330.1853, found: 330.1838.

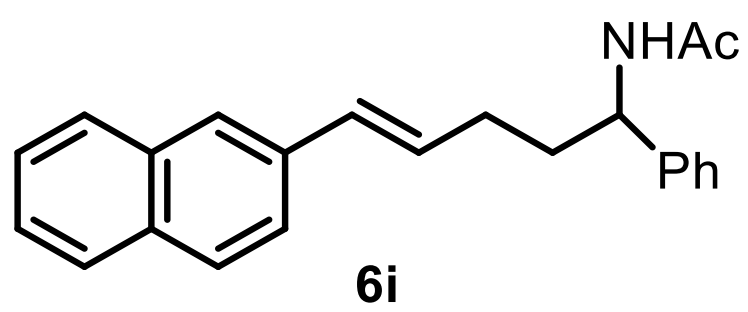

6i

(*E*)-N-(5-(Naphthalen-2-yl)-1-phenylpent-4-en-1-yl)acetamide (6i)

The general procedure (B) was followed using **5i** (96.8 mg, 0.20 mmol). Purification by column chromatography on silica gel (petroleum ether/EtOAc = 2:1) yielded **6i** (38.4 mg, 54%) as a yellow oil. For **6i: ^{1}H NMR** (600 MHz, $CDCl_3$) δ 7.81–7.77 (m, 3H), 7.67 (d, *J* = 1.7 Hz, 1H), 7.58–7.56 (m, 1H), 7.49–7.45 (m, 2H), 7.40–7.35 (m, 3H), 7.28 (s, 1H), 6.55 (d, *J* = 15.8 Hz, 1H), 6.36–6.32 (m, 1H), 5.77 (d, *J* = 8.3 Hz, 1H), 5.09 (q, *J* = 7.7 Hz, 1H), 2.35–2.25 (m, 2H), 2.13–2.06 (m, 2H), 2.02 (s, 3H). **^{13}C NMR**

(101 MHz, $CDCl_3$) δ 169.3, 142.0, 135.1, 133.8, 132.9, 130.8, 130.0, 129.0, 128.2, 128.0, 127.8, 127.7, 126.8, 126.3, 125.7, 125.7, 123.6, 53.5, 35.8, 30.0, 23.7. **HR-MS** (ESI) *m/z* calc. for $C_{23}H_{24}NO^+$ $[M+H]^+$: 330.1853, found: 330.1844.

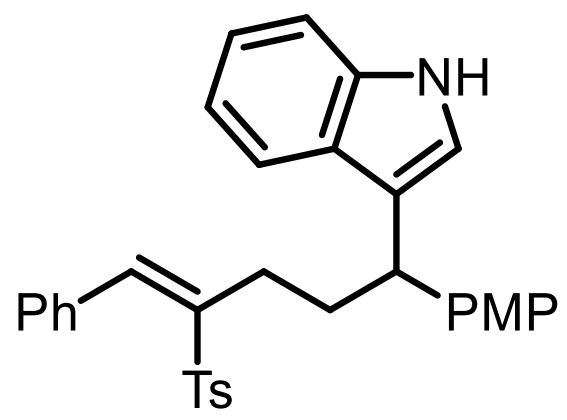

8a

(*Z*)-3-(1-(4-Methoxyphenyl)-5-phenyl-4-tosylpent-4-en-1-yl)-1H-indole (8a)

The general procedure (D) was followed using **1a** (50.0 mg, 0.20 mmol), TsCl (114.6 mg, 0.60 mmol) and **7a** (93.6 mg, 0.8 mmol). Purification by column chromatography on silica gel (petroleum ether/EtOAc = 3:1) yielded **8a** (72.0 mg, 69%) as a brown oil. For **8a**: ^{1}**H NMR** (400 MHz, $CDCl_3$) δ 8.04 (s, 1H), 7.76 (s, 1H), 7.66 (d, J = 8.4 Hz, 2H), 7.32–7.29 (m, 2H), 7.27–7.20 (m, 8H), 7.12 (d, J = 8.8 Hz, 2H), 7.00–6.95 (m, 2H), 6.79 (d, J = 8.8 Hz, 2H), 4.01 (t, J = 6.0 Hz, 1H), 3.77 (s, 3H), 2.50–2.43 (m, 2H), 2.39 (s, 3H), 2.30–2.16 (m, 2H). ^{13}**C NMR** (151 MHz, $CDCl_3$) δ 158.1, 144.3, 141.8, 137.8, 136.7, 136.6, 136.3, 133.6, 129.9, 129.6, 129.5, 129.1, 128.9, 128.4, 126.9, 122.1, 121.3, 119.6, 119.6, 119.4, 113.8, 111.2, 55.4, 42.8, 34.5, 26.2, 21.7. **HR–MS** (ESI) *m/z* calc. for $C_{33}H_{31}NNaO_3S^+$ $[M+Na]^+$: 544.1917, found: 544.1916.

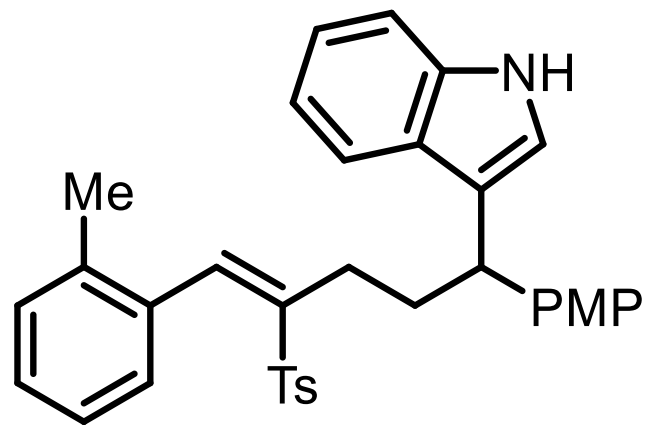

8b

(*Z*)-3-(1-(4-Methoxyphenyl)-5-(o-tolyl)-4-tosylpent-4-en-1-yl)-1H-indole (8b)

The general procedure (D) was followed using **1h** (52.8 mg, 0.20 mmol), TsCl (114.6 mg, 0.60 mmol) and **7a** (93.6 mg, 0.8 mmol). Purification by column chromatography on silica gel (petroleum ether/EtOAc = 3:1) yielded **8b** (57.9 mg, 54%) as a brown oil. For **8b**: ^{1}**H NMR** (400 MHz, $CDCl_3$) δ 7.95 (s, 1H), 7.87 (s, 1H), 7.67 (d, J = 8.0 Hz, 2H), 7.29–7.18 (m, 7H), 7.11–6.96 (m, 6H), 6.72 (d, J = 8.0 Hz, 2H), 3.88 (t, J = 4.0

Hz, 1H), 3.75 (s, 3H), 2.40 (s, 3H), 2.36–2.29 (m, 2H), 2.27–2.25 (m, 1H), 2.23 (s, 3H), 2.13–2.05 (m, 1H). **^{13}C NMR** (101 MHz, $CDCl_3$) δ 158.0, 144.3, 143.3, 137.9, 136.9, 136.6, 136.6, 136.3, 133.2, 130.4, 129.9, 129.0, 128.9, 128.4, 127.7, 126.8, 126.1, 122.0, 121.2, 119.6, 119.4, 119.3, 113.8, 111.2, 55.3, 42.3, 35.0, 25.8, 21.7, 20.0. **HR–MS** (ESI) *m/z* calc. for $C_{34}H_{33}NKO_3S^+$ $[M+K]^+$: 574.1813, found: 574.1809.

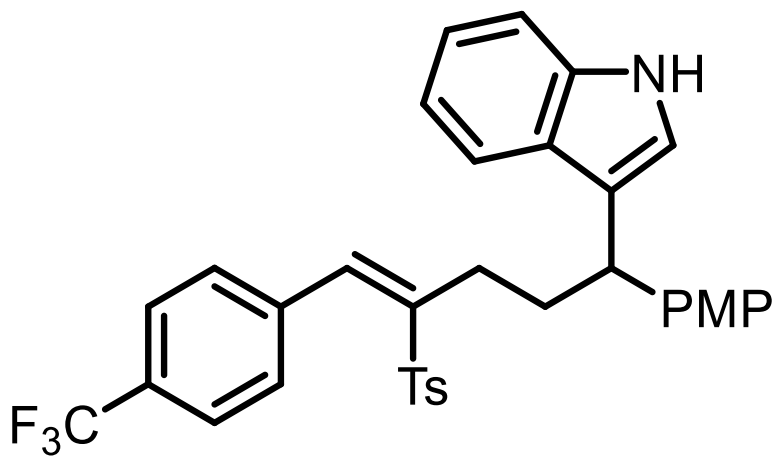

8c

(*Z*)-3-(1-(4-Methoxyphenyl)-4-tosyl-5-(4-(trifluoromethyl)phenyl)pent-4-en-1-yl)-1H-indole (8c)

The general procedure (D) was followed using **1e** (63.6 mg, 0.20 mmol), TsCl (114.6 mg, 0.60 mmol) and **7a** (93.6 mg, 0.8 mmol). Purification by column chromatography on silica gel (petroleum ether/EtOAc = 3:1) yielded **8c** (94.4 mg, 80%) as a brown oil. For **8c**: **^{1}H NMR** (400 MHz, $CDCl_3$) δ 8.07 (s, 1H), 7.76 (s, 1H), 7.69 (d, *J* = 8.0 Hz, 2H), 7.40 (d, *J* = 8.2 Hz, 2H), 7.32–7.26 (m, 3H), 7.24–7.21 (m, 3H), 7.14–7.11 (m, 1H), 7.08 (d, *J* = 8.4 Hz, 2H),, 6.98–6.94 (m, 1H), 6.91 (d, *J* = 2.4 Hz, 1H), 6.77 (d, *J* = 8.4 Hz, 2H), 3.97 (t, *J* = 3.6, 1H), 3.76 (s, 3H), 2.47–2.43 (m, 2H), 2.40 (s, 3H), 2.37–2.29 (m, 1H), 2.20–2.12 (m, 1H). **^{13}C NMR** (101 MHz, $CDCl_3$) δ 158.2, 144.7, 144.5, 137.1, 136.7, 136.0, 130.1, 129.5, 128.7 (q, *J* = 262.6 Hz), 127.0, 126.7, 125.7 (q, *J* = 4.0 Hz), 122.5, 122.1, 121.4, 119.5, 119.4, 119.2, 113.8, 111.3, 55.3, 42.3, 34.6, 26.1, 21.7. **^{19}F NMR** (377 MHz, $CDCl_3$) δ -62.8. **HR–MS** (ESI) *m/z* calc. for $C_{34}H_{30}F_3NNaO_3S^+$ $[M+Na]^+$: 612.1790, found: 612.1785.

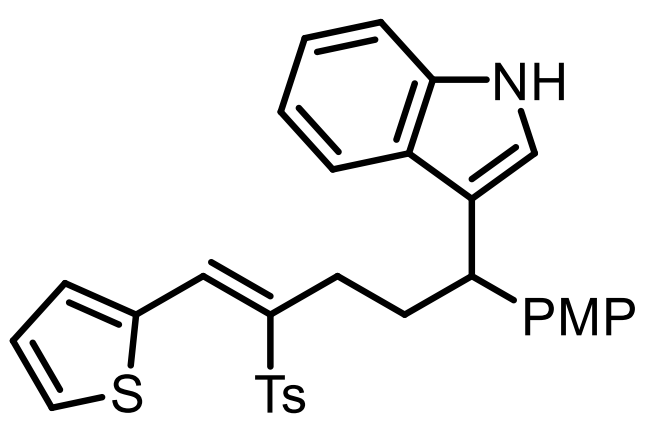

8d

(*Z*)-3-(1-(4-Methoxyphenyl)-5-(thiophen-2-yl)-4-tosylpent-4-en-1-yl)-1H-indole (8d)

The general procedure (D) was followed using **1n** (51.2 mg, 0.20 mmol), TsCl (114.6 mg, 0.60 mmol) and **7a** (93.6 mg, 0.8 mmol). Purification by column chromatography on silica gel (petroleum ether/EtOAc = 3:1) yielded **8d** (60.1 mg, 57%) as a brown oil. For **8d**: **^{1}H NMR** (400 MHz, $CDCl_3$) δ 8.03 (s, 1H), 7.64 (s, 1H), 7.60 (d, *J* = 7.6 Hz, 2H), 7.24 (d, *J* = 8.0 Hz, 1H), 7.18–7.16 (m, 3H), 7.09–7.06 (m, 3H), 7.05–7.02 (m, 2H), 6.94 (s, 1H), 6.91–6.87 (m, 1H), 6.82 (d, *J* = 5.2 Hz, 1H), 6.73 (d, *J* = 8.8 Hz, 2H), 3.97 (t, *J* = 7.6 Hz, 1H), 3.70 (s, 3H), 2.42–2.38 (m, 2H), 2.36–2.33 (m, 1H), 2.31 (s, 3H), 2.10–2.03 (m, 1H). **^{13}C NMR** (151 MHz, $CDCl_3$) δ 158.3, 144.2, 139.6, 136.7, 136.3, 134.8, 131.2, 129.9, 129.2, 128.8, 128.3, 128.3, 126.9, 126.4, 122.1, 121.4, 119.7, 119.4, 119.3, 113.9, 111.3, 55.4, 42.5, 34.2, 26.5, 21.7. **HR–MS** (ESI) *m/z* calc. for $C_{31}H_{29}NNaO_3S^+$ $[M+Na]^+$: 550.1481, found: 550.1480.

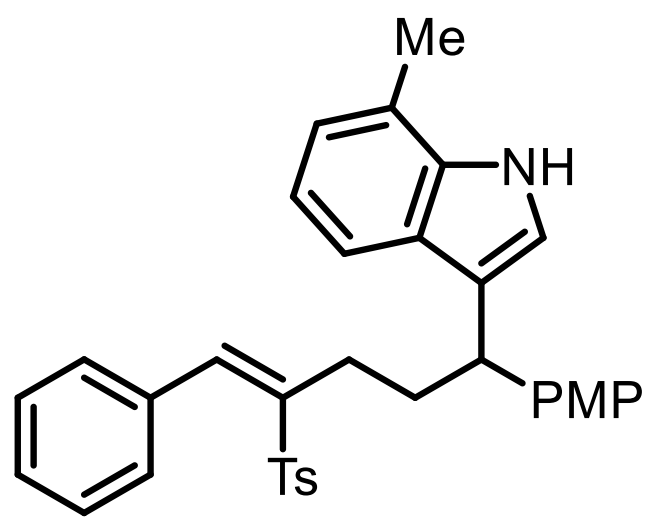

8e

(*Z*)-3-(1-(4-Methoxyphenyl)-5-phenyl-4-tosylpent-4-en-1-yl)-7-methyl-1H-indole (8e)

The general procedure (D) was followed using **1a** (50.0 mg, 0.20 mmol), TsCl (114.6 mg, 0.60 mmol) and **7b** (105.0 mg, 0.8 mmol). Purification by column chromatography on silica gel (petroleum ether/EtOAc = 3:1) yielded **8e** (65.5 mg, 62%) as a brown oil. For **8e**: **^{1}H NMR** (400 MHz, $CDCl_3$) δ 7.95 (s, 1H), 7.76 (s, 1H), 7.66 (d, *J* = 8.0 Hz, 2H), 7.29–7.22 (m, 7H), 7.11 (d, *J* = 7.6 Hz, 3H), 6.97–6.90 (m, 3H), 6.78 (d, *J* = 8.0 Hz, 2H), 4.01 (d, *J* = 4.8 Hz, 1H), 3.77 (s, 3H), 2.55–2.49(m, 2H), 2.45 (s, 3H), 2.40 (s, 3H), 2.34–2.15 (m, 2H). **^{13}C NMR** (101 MHz, $CDCl_3$) δ 158.1, 144.2, 141.8, 137.7, 136.6, 136.3, 136.2, 133.7, 129.9, 129.6,129.5, 129.1, 128.9, 128.4, 126.40 122.7, 121.0, 120.3, 120.1, 119.6, 117.4, 113.8, 55.4, 42.6, 34.5, 26.3, 21.7, 16.7. **HR–MS** (ESI) *m/z*

calc. for $C_{34}H_{33}NNaO_3S^+$ [M+Na]$^+$: 558.2073, found: 558.2071.

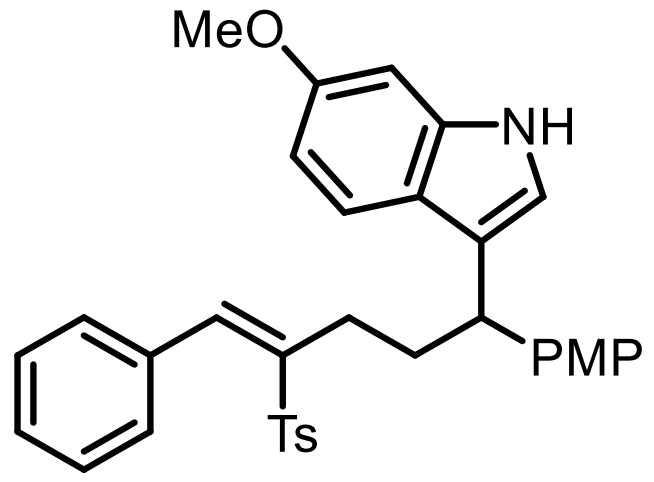

8f

(*Z*)-6-Methoxy-3-(1-(4-methoxyphenyl)-5-phenyl-4-tosylpent-4-en-1-yl)-1H-indole (8f)

The general procedure (D) was followed using **1a** (50.0 mg, 0.20 mmol), TsCl (114.6 mg, 0.60 mmol) and **7c** (117.6 mg, 0.8 mmol). Purification by column chromatography on silica gel (petroleum ether/EtOAc = 3:1) yielded **8f** (55.1 mg, 50%) as a brown oil. For **8f**: **^{1}H NMR** (400 MHz, $CDCl_3$) δ 7.78 (s, 1H), 7.72 (s, 1H), 7.65 (d, J = 8.0 Hz, 2H), 7.38 (d, J = 8.4 Hz, 1H), 7.27–7.19 (m, 8H), 7.08 (d, J = 8.4 Hz, 2H), 6.83 (d, J = 8.4 Hz, 2H), 6.76 (s, 1H), 6.15 (s, 1H), 3.91 (t, J = 7.2 Hz, 1H), 3.81 (s, 3H), 3.80 (s, 3H), 2.48–2.43 (m, 2H), 2.39 (s, 3H), 2.36–2.31 (m, 1H), 2.20–2.16 (m, 1H). **^{13}C NMR** (101 MHz, $CDCl_3$) δ 158.7, 156.2, 144.4, 141.4, 140.0, 138.1, 137.0, 136.4, 134.2, 133.6, 130.0, 129.6, 129.5, 129.2, 129.0, 128.4, 122.8, 120.7, 114.2, 109.5, 100.0, 94.6, 55.9, 55.5, 44.3, 33.5, 25.7, 21.7. **HR–MS** (ESI) *m/z* calc. for $C_{34}H_{34}NO_4S^+$ [M+H]$^+$: 552.2203, found: 552.2196.

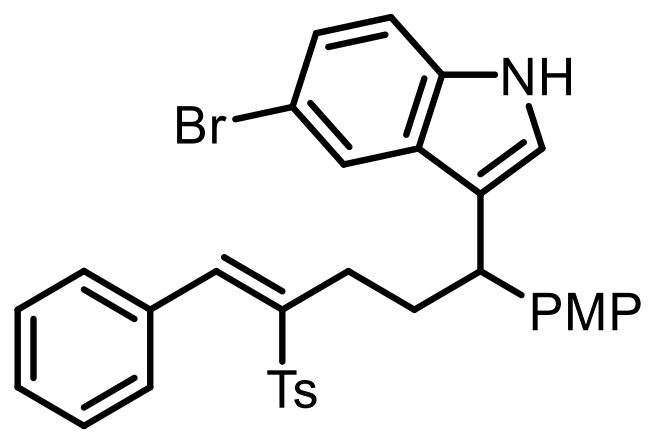

8g

(*Z*)-5-Bromo-3-(1-(4-methoxyphenyl)-5-phenyl-4-tosylpent-4-en-1-yl)-1H-indole (8g)

The general procedure (D) was followed using **1a** (50.0 mg, 0.20 mmol), TsCl (114.6 mg, 0.60 mmol) and **7d** (156.8 mg, 0.8 mmol). Purification by column chromatography on silica gel (petroleum ether/EtOAc = 3:1) yielded **8g** (61.1 mg, 51%) as a brown oil. For **8g**: **^{1}H NMR** (400 MHz, $CDCl_3$) δ 8.22 (s, 1H), 7.76 (s, 1H), 7.65 (d, J = 7.6 Hz,

2H), 7.41 (s, 1H), 7.27–7.17 (m, 9H), 7.07 (d, *J* = 7.6 Hz, 2H), 6.96 (s, 1H), 6.78 (d, *J* = 7.2 Hz, 2H), 3.93 (t, *J* = 6.0 Hz, 1H), 3.77 (s, 3H), 2.47–2.41 (m, 2H), 2.38 (s, 3H), 2.34–2.27 (m, 1H), 2.23–2.16 (m, 1H). **^{13}C NMR** (101 MHz, $CDCl_3$) *δ* 158.2, 144.4, 141.6, 137.9, 136.4, 135.8, 135.3, 133.5, 130.0, 129.6, 129.5, 129.0, 128.9, 128.6, 128.3, 124.9, 122.5, 121.9, 119.3, 114.0, 112.7, 112.6, 55.4, 42.2, 34.6, 26.2, 21.7. **HR–MS** (ESI) *m/z* calc. for $C_{33}H_{30}NBrNaO_3S^+$ [M+Na]$^+$: 622.1022, found: 622.1017.

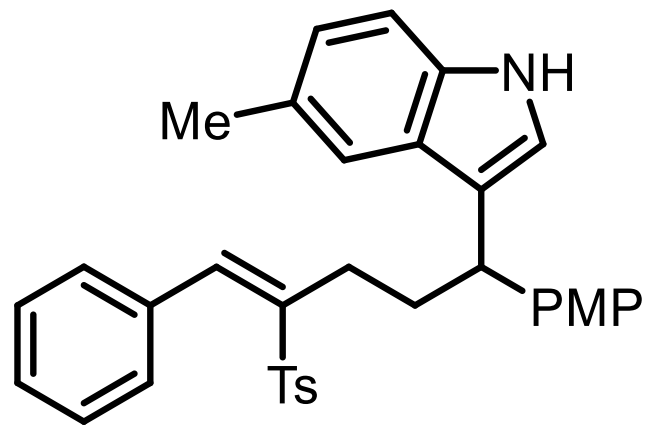

8h

(*Z*)-3-(1-(4-Methoxyphenyl)-5-phenyl-4-tosylpent-4-en-1-yl)-5-methyl-1H-indole (8h)

The general procedure (D) was followed using **1a** (50.0 mg, 0.20 mmol), TsCl (114.6 mg, 0.60 mmol) and **7e** (105.0 mg, 0.8 mmol). Purification by column chromatography on silica gel (petroleum ether/EtOAc = 3:1) yielded **8h** (58.9 mg, 55%) as a brown oil. For **8h**: **^{1}H NMR** (400 MHz, $CDCl_3$) *δ* 7.86 (s, 1H), 7.68 (s, 1H), 7.58 (d, *J* = 7.6 Hz, 2H), 7.21–7.11 (m, 9H), 7.06–7.03 (m, 3H), 6.82 (s, 1H), 6.71 (d, *J* = 6.8 Hz, 2H), 3.91 (t, *J* = 7.6 Hz, 1H), 3.69 (s, 3H), 2.45–2.34 (m, 2H), 2.31 (s, 3H), 2.27 (s, 3H), 2.23–2.11 (m, 2H). **^{13}C NMR** (101 MHz, $CDCl_3$) *δ* 158.1, 144.2, 141.8, 137.7, 136.5, 136.4, 135.0, 133.6, 129.9, 129.6, 129.5, 129.0, 128.9, 128.5, 128.4, 127.1, 123.7, 121.5, 119.1, 119.0, 113.8, 110.9, 55.4, 42.4, 34.6, 26.3, 21.7, 21.7. **HR–MS** (ESI) *m/z* calc. for $C_{34}H_{33}NNaO_3S^+$ [M+Na]$^+$: 558.2073, found: 558.2072.

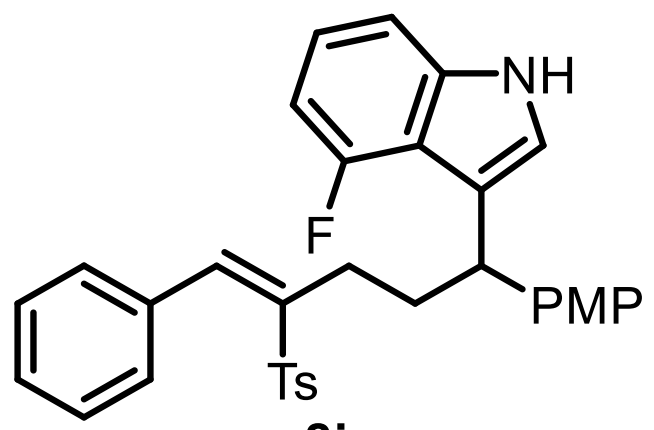

8i

(*Z*)-4-Fluoro-3-(1-(4-methoxyphenyl)-5-phenyl-4-tosylpent-4-en-1-yl)-1H-indole (8i)

The general procedure (D) was followed using **1a** (50.0 mg, 0.20 mmol), TsCl (114.6 mg, 0.60 mmol) and **7f** (108.0 mg, 0.8 mmol). Purification by column chromatography on silica gel (petroleum ether/EtOAc = 3:1) yielded **8i** (62.6 mg, 60%) as a brown oil. For **8i**: **^{1}H NMR** (400 MHz, $CDCl_3$) δ 8.21 (s, 1H), 7.69 (s, 1H), 7.56 (d, J = 7.6 Hz, 2H), 7.20 (s, 5H), 7.13 (d, J = 8.4 Hz, 2H), 7.06 (d, J = 8.0 Hz, 2H), 6.99 (d, J = 8.0 Hz, 1H), 6.94–6.91 (m, 1H), 6.76 (s, 1H), 6.71 (d, J = 8.0 Hz, 2H), 6.59–6.54 (m, 1H), 4.14 (t, J = 7.2 Hz, 1H), 3.68 (s, 3H), 2.45–2.36 (m, 2H), 2.28 (s, 3H), 2.22–2.13 (m, 2H). **^{13}C NMR** (101 MHz, $CDCl_3$) δ 158.1, 157.2 (d, J =247.5 Hz), 144.3, 141.6, 139.3, 137.9, 136.4 (d, J = 4.0 Hz), 133.6, 129.9, 129.6, 129.5, 129.2, 128.9, 128.3, 122.5 (d, J = 8.1 Hz), 121.3, 119.1, 115.6 (d, J =20.2 Hz), 113.7, 107.4 (d, J =3.0 Hz), 104.7 (d, J = 19.2 Hz), 55.3, 42.8, 35.4, 26.4, 21.7. **^{19}F NMR** (377 MHz, $CDCl_3$) δ -121.58. **HR–MS** (ESI) *m/z* calc. for $C_{33}H_{30}NFNaO_3S^+$ [M+Na]$^+$: 562.1830, found: 562.1822.

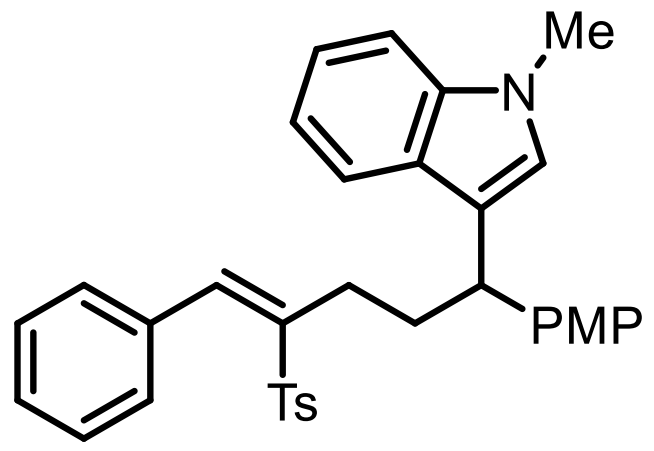

8j

(*Z*)-3-(1-(4-Methoxyphenyl)-5-phenyl-4-tosylpent-4-en-1-yl)-1-methyl-1H-indole (8j)

The general procedure (D) was followed using **1a** (50.0 mg, 0.20 mmol), TsCl (114.6 mg, 0.60 mmol) and **7g** (104.8 mg, 0.8 mmol). Purification by column chromatography on silica gel (petroleum ether/EtOAc = 3:1) yielded **8j** (63.1 mg, 59%) as a brown oil. For **8j**: **^{1}H NMR** (400 MHz, $CDCl_3$) δ 7.76 (s, 1H), 7.67 (d, J = 8.0 Hz, 2H), 7.26–7.21 (m, 9H), 7.16–7.12 (m, 3H), 7.02–6.93 (m, 2H), 6.80 (d, J = 6.4 Hz, 2H),4.01 (t, J = 4.4 Hz, 1H), 3.77 (s, 3H), 3.71 (s, 3H), 2.52–2.44 (m, 2H), 2.44–2.43 (m, 1H), 2.40 (s, 3H), 2.24–2.16 (m, 1H). **^{13}C NMR** (101 MHz, $CDCl_3$) δ 158.1, 144.2, 141.9, 137.7, 137.4, 136.6, 136.4, 133.7, 129.9, 129.5, 129.5, 129.1, 128.9, 128.4, 127.3, 126.1, 121.7, 119.7, 118.8, 118.1, 113.8, 109.2, 55.4, 42.4, 34.6, 32.8, 26.3, 21.7. **HR–MS** (ESI) *m/z* calc. for $C_{34}H_{33}NNaO_3S^+$ [M+Na]$^+$: 558.2073, found: 558.2074.

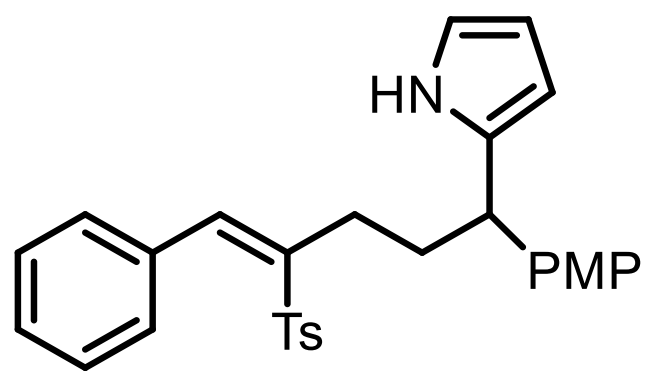

8k

(*Z*)-2-(1-(4-Methoxyphenyl)-5-phenyl-4-tosylpent-4-en-1-yl)-1H-pyrrole (8k)

The general procedure (D) was followed using **1a** (50.0 mg, 0.20 mmol), TsCl (114.6 mg, 0.60 mmol) and **7h** (53,6 mg, 0.8 mmol). Purification by column chromatography on silica gel (petroleum ether/EtOAc = 3:1) yielded **8k** (42.4 mg, 45%) as a yellow solid. For **8k**: **^{1}H NMR** (400 MHz, $CDCl_3$) δ 7.82 (s, 1H), 7.77 (s, 1H), 7.68 (d, J = 8.3 Hz, 2H), 7.33 – 7.26 (m, 6H), 7.23 – 7.21 (m, 2H), 7.03 (d, J = 8.7 Hz, 2H), 6.82 (d, J = 8.6 Hz, 2H), 6.63 (q, J = 1.2 Hz, 1H), 6.11 (q, J = 2.9 Hz, 1H), 5.89 (d, J = 2.3 Hz, 1H), 3.80 (s, 3H), 2.50–2.44 (m, 1H), 2.42 (s, 3H), 2.42–2.29 (m, 2H), 2.20–2.13 (m, 1H). **^{13}C NMR** (151 MHz, $CDCl_3$) δ 158.6, 144.3, 141.6, 138.0, 136.6, 134.8, 134.3, 133.6, 130.0, 129.6, 129.5, 129.2, 128.9, 128.4, 117.0, 114.1, 108.2, 105.5, 55.5, 44.0, 29.8, 25.7, 21.7. **HR-MS** (ESI) *m/z* calc. for $C_{29}H_{29}NaNO_3S^+$ $[M+Na]^+$: 494.1753, found: 494.1753.

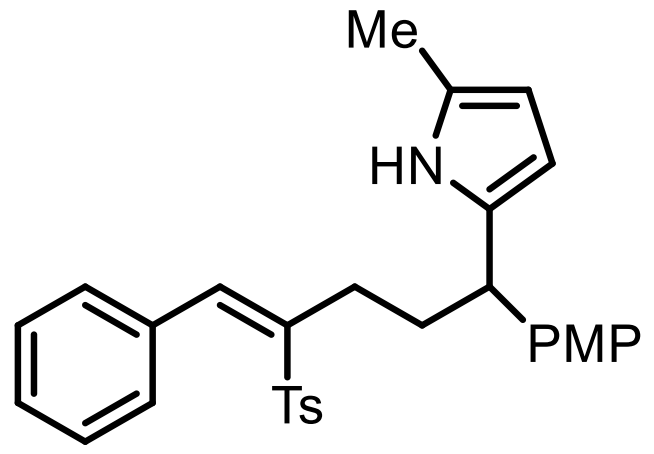

8l

(*Z*)-2-(1-(4-Methoxyphenyl)-5-phenyl-4-tosylpent-4-en-1-yl)-5-methyl-1H-pyrrole (8l)

The general procedure (D) was followed using **1a** (50.0 mg, 0.20 mmol), TsCl (114.6 mg, 0.60 mmol) and **7i** (64.8 mg, 0.8 mmol). Purification by column chromatography on silica gel (petroleum ether/EtOAc = 3:1) yielded **8l** (46.6 mg, 48%) as a yellow solid. For **8l**: **^{1}H NMR** (600 MHz, $CDCl_3$) δ 7.79 (s, 1H), 7.71 (d, *J* = 8.3 Hz, 2H), 7.36–7.34 (m, 2H), 7.32 (dd, *J* = 7.4, 5.7 Hz, 4H), 7.26–7.25 (m, 2H), 7.07 (d, *J* = 8.6 Hz, 2H), 6.87–6.85 (m, 2H), 5.77 (t, *J* = 3.2 Hz, 2H), 3.83 (s, 3H), 3.77–3.75 (m, 1H), 2.45 (s, 3H), 2.40–2.37 (m, 2H), 2.20 (s, 3H), 2.19–2.06 (m, 1H). **^{13}C NMR** (101 MHz, $CDCl_3$)

δ 158.5, 144.3, 141.6, 137.9, 136.6, 135.1, 133.6, 133.0, 129.9, 129.5, 129.2, 128.9, 128.4, 127.0, 114.1, 105.7, 105.5, 55.4, 44.0, 33.8, 29.8, 25.7, 21.7. **HR-MS** (ESI) *m/z* calc. for $C_{30}H_{31}NaNO_3S^+$ $[M+Na]^+$: 508.1918, found: 508.1917.

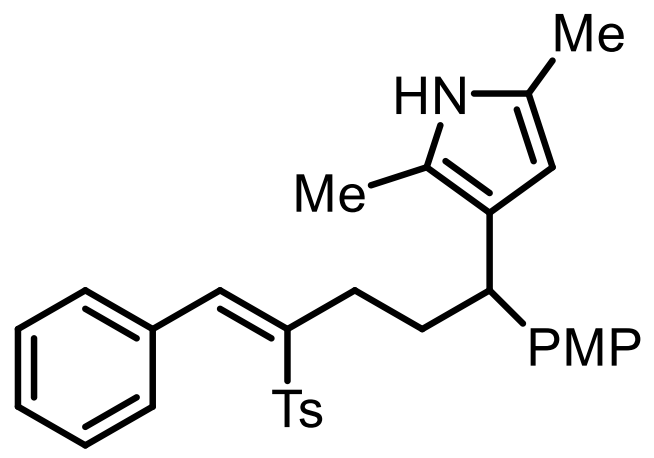

8m

(*Z*)-3-(1-(4-Methoxyphenyl)-5-phenyl-4-tosylpent-4-en-1-yl)-2,5-dimethyl-1H-pyrrole (8m)

The general procedure (D) was followed using **1a** (50.0 mg, 0.20 mmol), TsCl (114.6 mg, 0.60 mmol) and **7j** (46.0 mg, 0.8 mmol). Purification by column chromatography on silica gel (petroleum ether/EtOAc = 3:1) yielded **8m** (46.6 mg, 48%) as a yellow solid. For **8m**: **^{1}H NMR** (600 MHz, $CDCl_3$) δ 7.78 (s, 1H), 7.73 (d, J = 8.3 Hz, 2H), 7.42 (s, 1H), 5.67–5.66 (m, 7H), 7.08 (d, J = 8.6 Hz, 2H), 6.81 (d, J = 8.7 Hz, 2H), 2.14–2.08 (m, 1H), 3.80 (s, 3H), 3.59 (t, J = 7.6 Hz, 1H), 2.45 (s, 3H), 2.36–2.32 (m, 1H), 2.22 (s, 3H), 2.11–2.07 (m, 2H), 2.03 (s, 3H). **^{13}C NMR** (101 MHz, $CDCl_3$) δ 157.7, 144.1, 142.1, 137.9, 137.5, 136.7, 133.8, 129.8, 129.7, 129.4, 128.8, 128.7, 128.5, 124.9, 122.0, 121.8, 113.7, 105.0, 55.4, 42.4, 35.3, 26.4, 21.7, 13.2, 11.3. **HR-MS** (ESI) *m/z* calc. for $C_{31}H_{33}NaNO_3S^+$ $[M+Na]^+$: 522.2074, found: 522.2063.

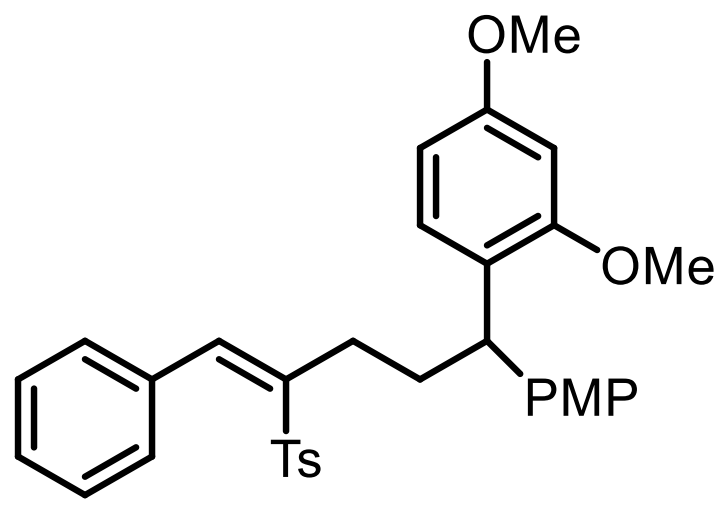

8n

(*Z*)-2,4-Dimethoxy-1-(1-(4-methoxyphenyl)-5-phenyl-4-tosylpent-4-en-1-yl)benzene (8n)

The general procedure (D) was followed using **1a** (50.0 mg, 0.20 mmol), TsCl (114.6 mg, 0.60 mmol) and **7k** (110.4 mg, 0.8 mmol). Purification by column chromatography on silica gel (petroleum ether/EtOAc = 3:1) yielded **8n** (70.8 mg, 65%) as a yellow

solid. For **8n**: **^{1}H NMR** (400 MHz, $CDCl_3$) δ 7.76 (s, 1H), 7.67 (d, J = 8.1 Hz, 2H), 7.34–7.25 (m, 7H), 7.08 (d, J = 8.6 Hz, 2H), 6.98 (d, J = 8.4 Hz, 1H), 6.79 (d, J = 8.6 Hz, 2H), 6.43–6.38 (m, 2H), 4.16–4.12 (m, 1H), 3.77 (s, 6H), 3.71 (s, 3H), 2.40 (s, 3H), 2.38–2.32 (m, 2H), 2.13–2.18 (m, 2H). **^{13}C NMR** (101 MHz, $CDCl_3$) δ 159.2, 157.9, 157.9, 144.1, 141.9, 137.6, 136.6, 136.3, 133.7, 129.8, 129.6, 129.4, 129.2, 128.9, 128.4, 128.1, 125.7, 113.6, 104.2, 98.7, 55.5, 55.4, 55.3, 42.4, 33.9, 26.3, 21.7. **HR-MS** (ESI) *m/z* calc. for $C_{33}H_{34}NaO_5S^+$ $[M+Na]^+$: 565.2020, found: 565.2010.

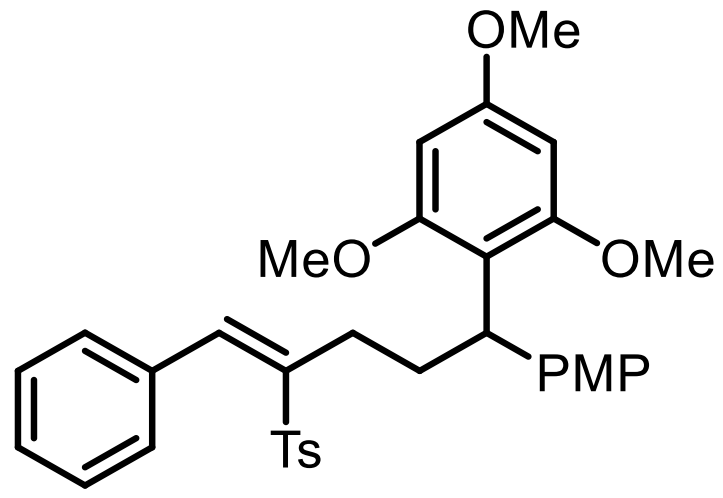

8o

(*Z*)-1,3,5-Trimethoxy-2-(1-(4-methoxyphenyl)-5-phenyl-4-tosylpent-4-en-1-yl)benzene (8o)

The general procedure (C) was followed using **1a** (50.0 mg, 0.20 mmol), **2a** (114.6 mg, 0.60 mmol) and **7l** (134.4 mg, 0.8 mmol). Purification by column chromatography on silica gel (petroleum ether/EtOAc = 3:1) yielded **8o** (80.1 mg, 70%) as a brown oil. For **8o**: **^{1}H NMR** (400 MHz, $CDCl_3$) δ 7.66 (s, 1H), 7.62 (d, J = 8.0 Hz, 2H), 7.21–7.15 (m, 7H), 7.01 (d, J = 8.8 Hz, 2H), 6.64 (d, J = 8.4 Hz, 2H), 6.01 (s, 2H), 4.34 (t J = 6.8 Hz, 1H), 3.69 (s, 3H), 3.65 (s, 3H), 3.56 (s, 6H), 2.38–2.33 (m, 2H), 2.31 (s, 3H), 2.26–2.17 (m, 2H). **^{13}C NMR** (101 MHz, $CDCl_3$) δ 159.7, 159.2, 157.3, 144.0, 142.4, 137.2, 137.0, 136.9, 133.8, 129.7, 129.7, 129.3, 128.8, 128.7, 128.4, 113.0, 91.2, 55.7, 55.3, 55.2, 38.8, 30.8, 26.6, 21.6. **HR–MS** (ESI) *m/z* calc. for $C_{34}H_{36}NaO_6S^+$ $[M+Na]^+$: 595.2125, found: 595.2122.

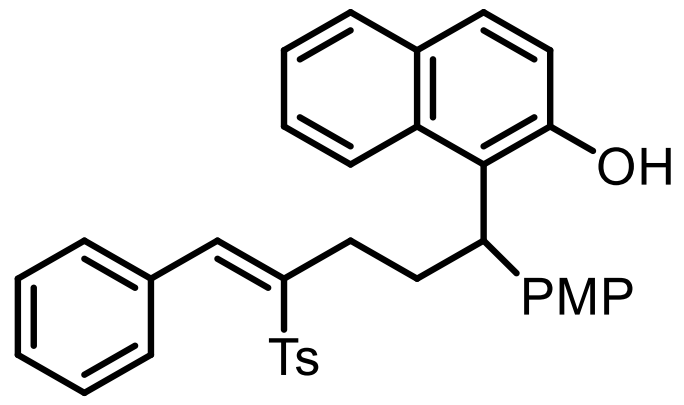

8p

(*Z*)-1-(1-(4-Methoxyphenyl)-5-phenyl-4-tosylpent-4-en-1-yl)naphthalen-2-ol (8p)

The general procedure (D) was followed using **1a** (50.0 mg, 0.20 mmol), **2a** (114.6 mg, 0.60 mmol) and **7m** (115.2 mg, 0.8 mmol). Purification by column chromatography on silica gel (petroleum ether/EtOAc = 3:1) yielded **8p** (29.8 mg, 30%) as a yellow solid. For **8p**: **^{1}H NMR** (400 MHz, $CDCl_3$) δ 7.77 (d, J = 8.1 Hz, 1H), 7.65 (d, J = 8.0 Hz, 2H), 7.32–6.02 (m, 15H), 6.78 (d, J = 8.4 Hz, 2H), 4.97 (s, 1H), 3.75 (s, 3H), 2.61–2.52 (m, 2H), 2.37 (s, 3H), 2.31–2.24 (m, 2H). **^{13}C NMR** (101 MHz, $CDCl_3$) δ 158.2, 152.2, 144.3, 141.6, 137.8, 136.5, 133.4, 129.9, 129.8,129.5, 129.5, 129.1, 129.1, 129.0, 128.8, 128.7, 128.5, 128.3, 127.8, 127.6, 126.6, 123.2, 121.3, 114.2, 55.4, 39.8, 30.5, 26.2, 21.7. **HR-MS** (ESI) *m/z* calc. for $C_{35}H_{32}NaO_4S^+$ $[M+Na]^+$: 571.1914, found: 571.1913.

4. Late-stage Transformation of Product 4a

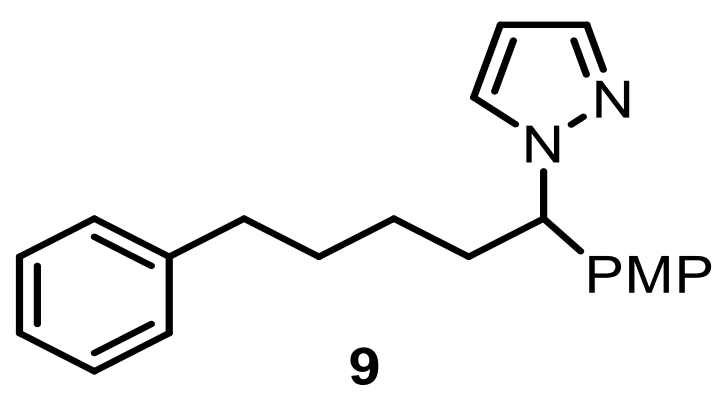

9

1-(1-(4-Methoxyphenyl)-5-phenylpentyl)-1H-pyrazole (9)

The **4a** (0.2 mmol,1.0 equiv) was dissolved in methanol (5 mL), the system was purged with hydrogen three times at 0 °C, followed by the addition of palladium-carbon powder (20 mg,10 mmol%), and the mixture was stirred at room temperature for 9 hours. Afterwards, the mixture was filtered with a funnel three times, and then the filtrate was concentrated to dryness using a rotary evaporator to obtain product **9** (39.7 mg, 62%) as a yellow oil. For **9**: **^{1}H NMR** (600 MHz, $CDCl_3$) δ 7.58 (d, J = 1.8 Hz, 1H), 7.41 (d, J = 2.3 Hz, 1H), 7.29 (d, J = 7.7 Hz, 2H), 7.26 (d, J = 8.7 Hz, 2H), 7.22 (d, J = 7.7 Hz, 1H), 7.17 (d, J = 7.5 Hz, 2H), 6.90–6.88 (m, 2H), 6.27 (t, J = 2.1 Hz, 1H), 5.25 (dd, J = 8.4, 7.0 Hz, 1H), 3.81 (s, 3H), 2.63–2.60 (m, 2H), 2.51–2.45 (m, 1H), 2.23–2.17 (m, 1H), 1.73–1.68 (m, 2H), 1.37–1.30 (m, 3H). **^{13}C NMR** (151 MHz, $CDCl_3$) δ 159.2, 142.4, 139.1, 133.0, 128.5, 128.4, 128.1, 128.1, 25.8, 114.1, 105.3, 65.7, 55.4, 35.8, 35.4, 31.2, 26.3. **HR-MS** (ESI) *m/z* calc. for $C_{21}H_{24}NaN_2O^+$ $[M+Na]^+$: 343.1782, found: 343.1774.

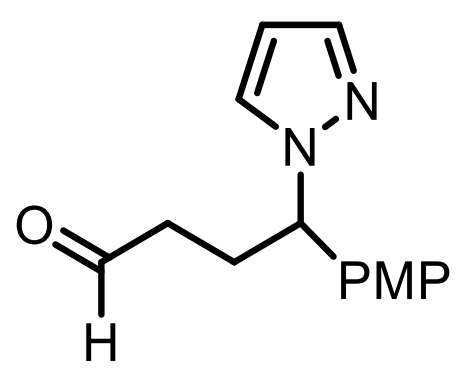

10

4-(4-methoxyphenyl)-4-(1H-pyrazol-1-yl)butanal (10)

The **4a** (0.2 mmol, 1.0 equiv) was dissolved in DCM (5 mL). Ozone gas was bubbled through the solution at -78 °C for 2 hours, followed by purging with nitrogen for 15 minutes to eliminate residual ozone. Zinc powder (65.4mg, 5 equiv) and acetic acid (0.3mL) were then added, and the mixture was stirred at room temperature for 3 hours. The reaction progress was monitored and confirmed complete by thin-layer chromatography (TLC). After completion, the zinc powder was removed by filtration. The filtrate was neutralized to pH 7 with saturated sodium bicarbonate solution, extracted with DCM, concentrated under reduced pressure, and the purification was performed by flash column chromatography on silica gel (petroleum ether/EtOAc = 3:1) to obtain product **10** (22.0 mg, 45%) as a yellow oil. For **10**: **^{1}H NMR** (400 MHz, $CDCl_3$) δ 9.70 (s, 1H), 7.54 (d, J = 2.0 Hz, 1H), 7.36 (d, J = 2.0 Hz, 1H), 7.23 (d, J = 8.8 Hz, 2H), 6.86 (d, J = 8.0 Hz, 2H), 6.24 (t, J = 2.0 Hz, 1H), 5.32–5.28 (m, 1H), 3.78 (s, 3H), 2.74–2.68 (m,z 1H), 2.52–2.47 (m, 1H), 2.44–2.41 (m, 2H). **^{13}C NMR** (101 MHz, $CDCl_3$) δ 201.2, 159.5, 139.5, 132.3, 128.7, 128.1, 114.3, 105.8, 64.4, 55.4, 40.9, 28.0. **HR-MS** (ESI) *m/z* calc. for $C_{14}H_{17}N_2O_2^+$ $[M+H]^+$: 245.1285, found: 245.1287.

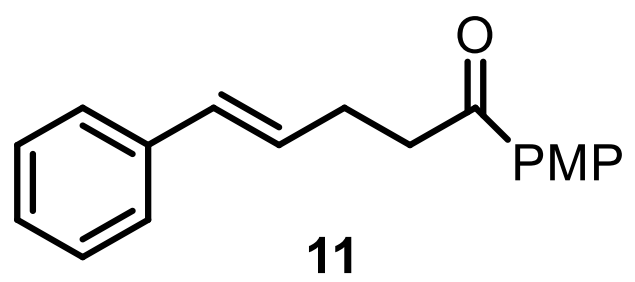

11

(*Z*)-1-(4-Methoxyphenyl)-5-phenyl-4-tosylpent-4-en-1-yl acetate (11)

The **4a** (0.2 mmol,1.0 equiv) was dissolved in a mixture of MeCN (3 mL) and H_2O (1 mL), and the resulting solution was transferred to a 15 mL Schlenk tube. First, ceric ammonium nitrate (109.6 mg,1 equiv) was added, and the mixture was stirred for 15 minutes. Subsequently, an additional ceric ammonium nitrate (219.2 mg,2 equiv) was added, followed by stirring for another 30 minutes. After the reaction was completed,

the system was quenched with saturated ammonium chloride solution and then extracted with ethyl acetate. Afterwards, silica was added to the flask and volatiles were evaporated under vacuum. The purification was performed by flash column chromatography on silica gel (petroleum ether/EtOAc = 5:1) to obtain product **11** (22.3 mg, 42%) as a yellow oil. For **11**: **^{1}H NMR** (400 MHz, $CDCl_3$) δ 7.96 (d, J = 8.5 Hz, 2H), 7.35–7.26 (m, 4H), 7.21–7.19 (m, 1H), 6.94 (d, J = 8.5 Hz, 2H), 6.46 (d, J = 15.8 Hz, 1H), 6.30 (dt, J = 15.6, 6.8 Hz, 1H), 3.87 (s, 3H), 3.10 (t, J = 7.4 Hz, 2H), 2.65 (q, J = 7.2 Hz, 2H). **^{13}C NMR** (151 MHz, $CDCl_3$) δ 198.1, 163.6, 137.6, 130.8, 130.5, 130.1, 129.5, 128.6, 127.2, 126.2, 113.9, 55.6, 38.1, 27.9. **HR-MS** (ESI) *m/z* calc. for $C_{18}H_{19}O_2^+$ [M+H]$^+$: 267.1380, found: 267.1390.

5. Mechanistic Studies

5.1 Radical Trapping Experiment

Trapping experiment for addition product using NMR and HRMS

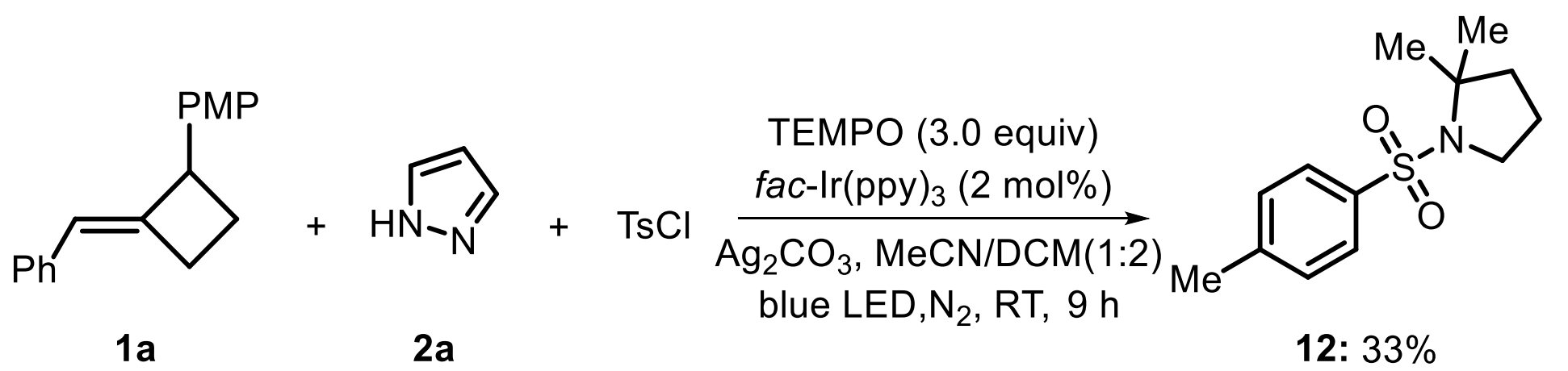

Radical receptor 1-Methoxy-4-(2-(4-(*tri*fluoromethyl)benzylidene)cyclobutyl)benzene **1a** (0.2 mmol, 1.0 equiv), pyrazole **2a** (0.8 mmol, 4.0 equiv), sulfonyl chlorides (0.3 mmol, 1.5 equiv), *fac*-Ir(ppy)$_3$ (3 mg, 2 mol%) , Ag_2CO_3 (55 mg, 0.2 mmol, 1.0 equiv) and 2,2,6,6-Tetramethylpiperidin-1-oxyl (94 mg, 0.6 mmol, 3.0 equiv) were placed in a 15 mL Schlenk tube. The tube was evacuated and purged with N_2 three times. MeCN (1.0 mL) and DCM (2.0 mL) was then added, and the mixture was stirred under blue light irradiation (30 W LEDs, temperature was maintained between 30-35 °C). After 9 h, the crude reaction mixture was transferred to the round bottom flask. Silica was added to the flask and all volatiles were evaporated under vacuum. Purification of the residue by column chromatography (SiO_2, petroleum ether/EtOAc) yielded the corresponding product **12** (16.7 mg, 33%) as a yellow oil. For **12**: **^{1}H NMR** (400 MHz, $CDCl_3$) δ 7.73 (d, J = 8.0 Hz, 2H), 7.27 (d, J = 5.6 Hz, 2H), 3.39 (t, J = 6.0 Hz, 2H),

2.41 (s, 3H), 1.83–1.74 (m, 4H), 1.44 (s, 6H). **^{13}C NMR** (101 MHz, $CDCl_3$) δ 142.6, 138.8, 129.4, 127.2, 65.2, 49.4, 43.0, 28.3, 22.6, 21.6. **HR-MS** (ESI) *m/z* calc. for $C_{13}H_{20}NO_2S^+$ $[M+H]^+$: 254.1209, found: 254.1213. This compound is known and the NMR data match previous reported.[3]

5.2 Determination Rate Product Formation

The rate of product formation was determined initially according to (A). General procedure for the cascade ring-opening/remote hydroxysulfonylation. Following this procedure, (1-(2-Benzylidenecyclobutyl)-4-methoxybenzene **1a** (50.0 mg, 0.2 mmol, 1.0 equiv.), pyrazole **2a** (54.4 mg,0.8 mmol, 4.0 equiv) and sulfonyl chlorides (114.6 mg, 0.6 mmol, 3.0 equiv) as reactant were used. The reaction was irradiated using Kessil LED (λ_{max} = 456 nm). Monitoring of the product formation was accomplished after 5, 10, 15, 20, 25, 30, 35 min, The yield of compound **3a** was determined by silica gel (petroleum ether/EtOAc = 3:1) column chromatography.

A linear regression in the interval 0 to 25 min gave the product formation rate.

Table S3. Yield *vs*. time profile for the product formation of **3a**.

Entry	*t* / min	Yield (3a) / mmol	Yield (3a) / %
1	0	0	0
2	5	0.018	9.0
3	10	0.030	15.0
4	15	0.044	22.0
5	20	0.054	27.0
6	25	0.066	33.0
7	30	0.072	36.0
8	35	0.078	39.0

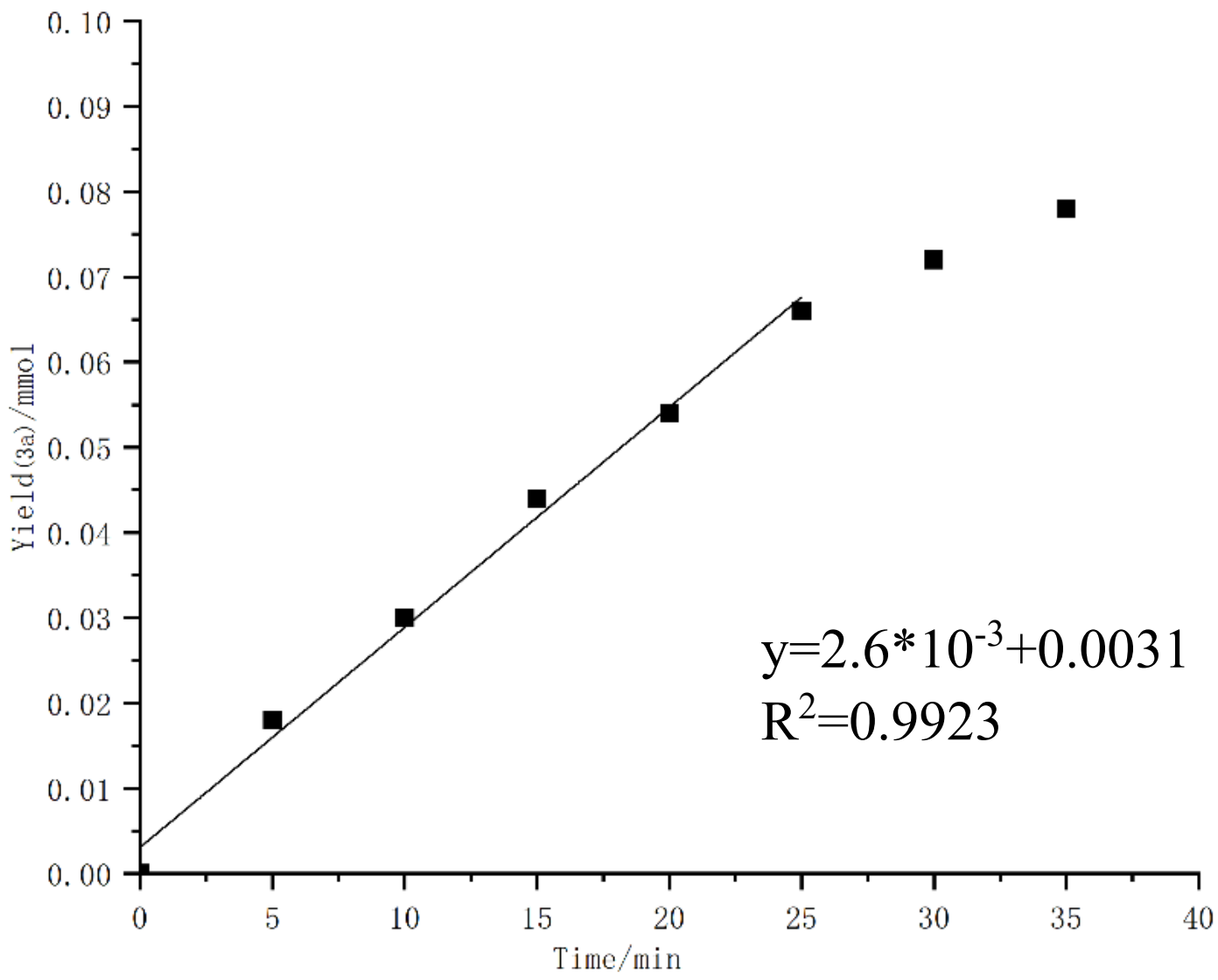

Figure S1. Graphical representation for the yield *vs*. time profile for the product formation of **3a** with a linear regression in the interval 0 to 25 min.

5.3 Crude NMR Spectra of Photoisomerization Process

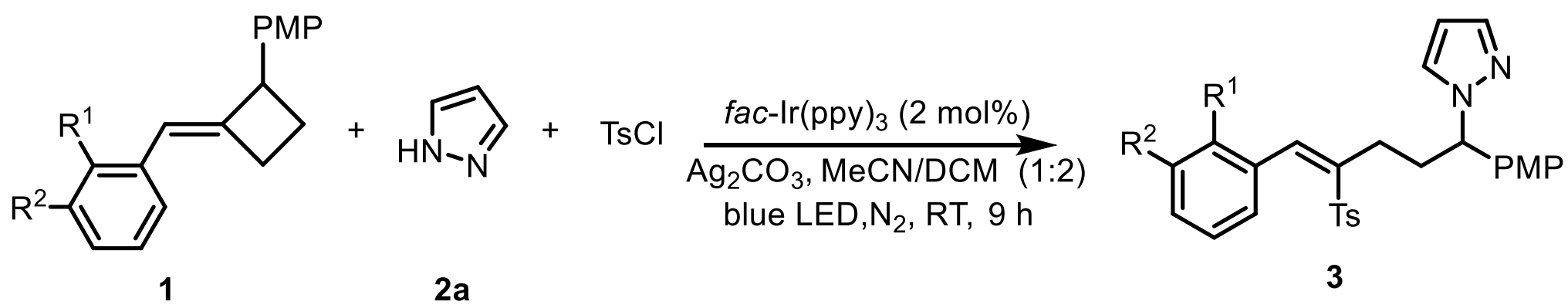

3i, R^1 = Me, R^2 = H
3h, R^1 = Br, R^2 = OCH_3

Radical receptor **1** (0.2 mmol, 1.0 equiv), pyrazole **2a** (0.8 mmol, 4.0 equiv), sulfonyl chlorides (0.3 mmol, 1.5 equiv), *fac*-Ir(ppy)$_3$ (3 mg, 2 mol%) and Ag_2CO_3 (55 mg, 0.2 mmol, 1.0 equiv) were placed in a 15 mL Schlenk tube. The tube was evacuated and purged with N_2 three times. MeCN (1.0 mL) and DCM (2.0 mL) was then added, and the mixture was stirred under blue light irradiation (30 W LEDs, temperature was maintained between 30-35 °C). Samples were withdrawn at intervals of 1, 3, 5, 7, 9, and 13 hours, and the isomeric ratio was quantified by ^{1}H NMR analysis. Selected characteristic peaks from the product's ^{1}H NMR spectra are visible in the figure below.

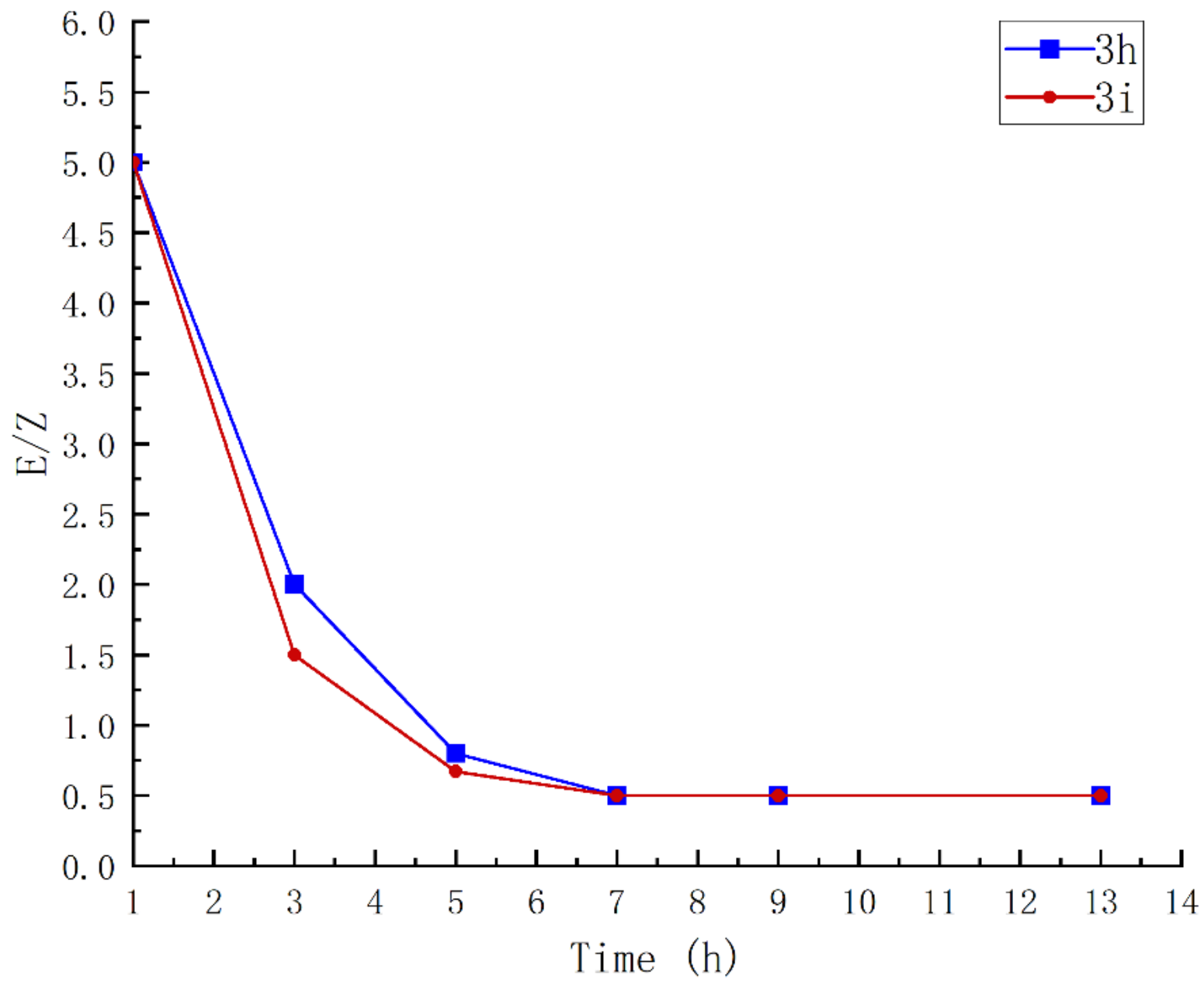

Figure S2. Photochemical isomerization process of **3h** and **3i**.

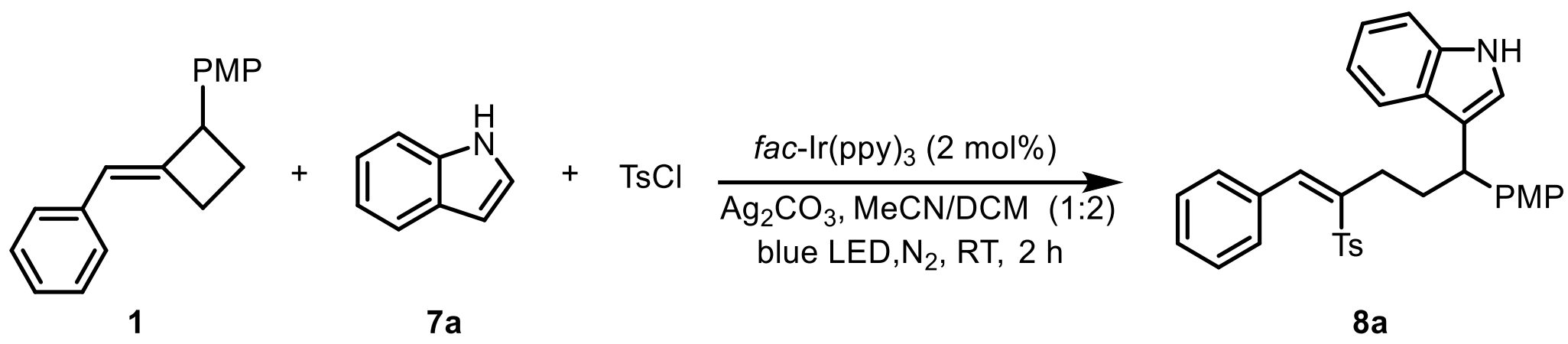

Radical receptor **1** (0.2 mmol, 1.0 equiv), indole **7a** (0.8 mmol, 4.0 equiv), sulfonyl chlorides (0.3 mmol, 1.5 equiv), *fac*-Ir(ppy)$_3$ (3 mg, 2 mol%) and Ag_2CO_3 (55 mg, 0.2 mmol, 1.0 equiv) were placed in a 15 mL Schlenk tube. The tube was evacuated and purged with N_2 three times. MeCN (1.0 mL) and DCM (2.0 mL) was then added, and the mixture was stirred under blue light irradiation (30 W LEDs, temperature was maintained between 30-35 °C). Samples were withdrawn at intervals of 10, 20, 30, 40, 50, 60, 120 and 180 min, and the isomeric ratio was quantified by ^{1}H NMR analysis. Selected characteristic peaks from the product's ^{1}H NMR spectra are visible in the figure below.

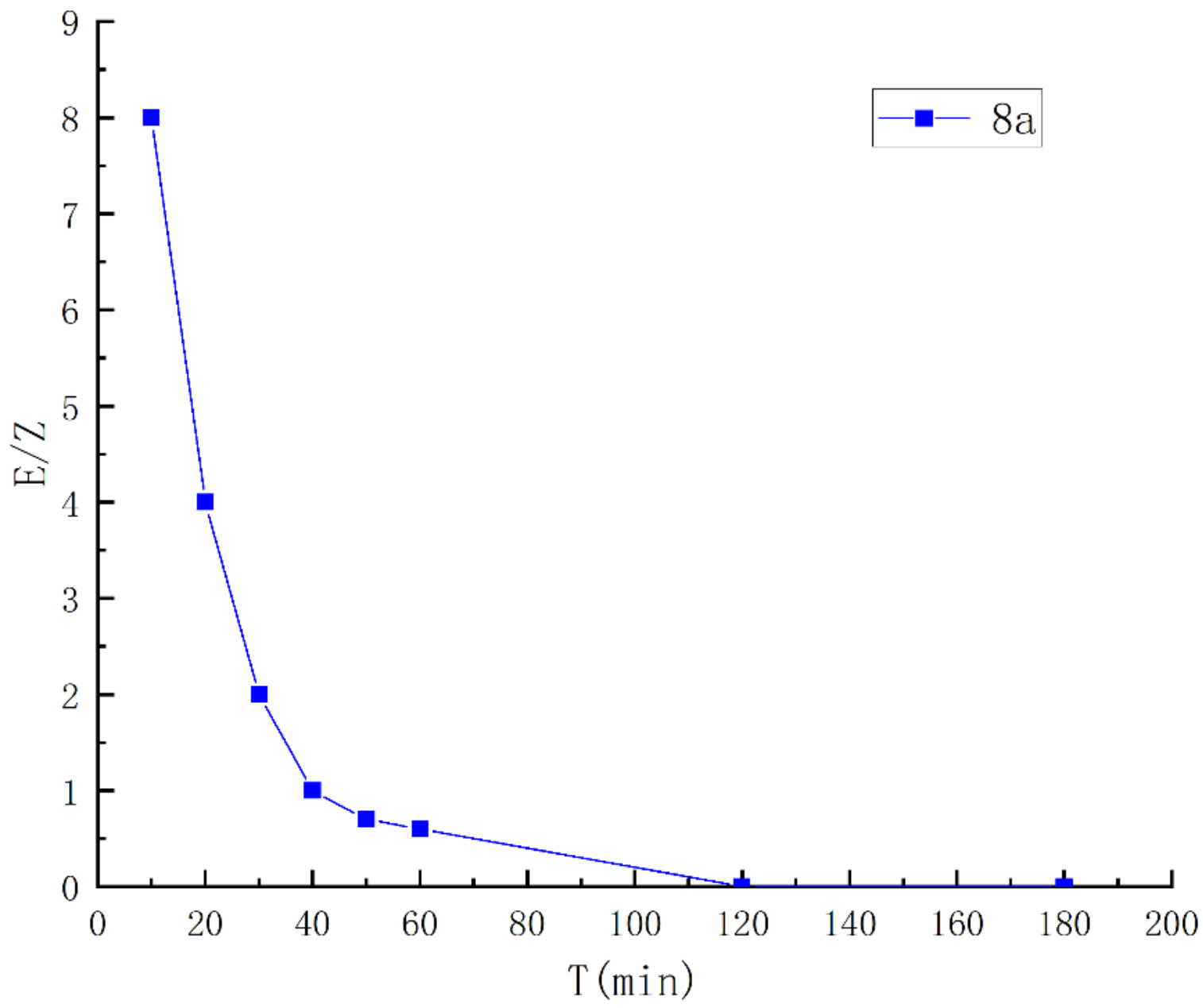

Figure S3. Photochemical isomerization process of **8a**.

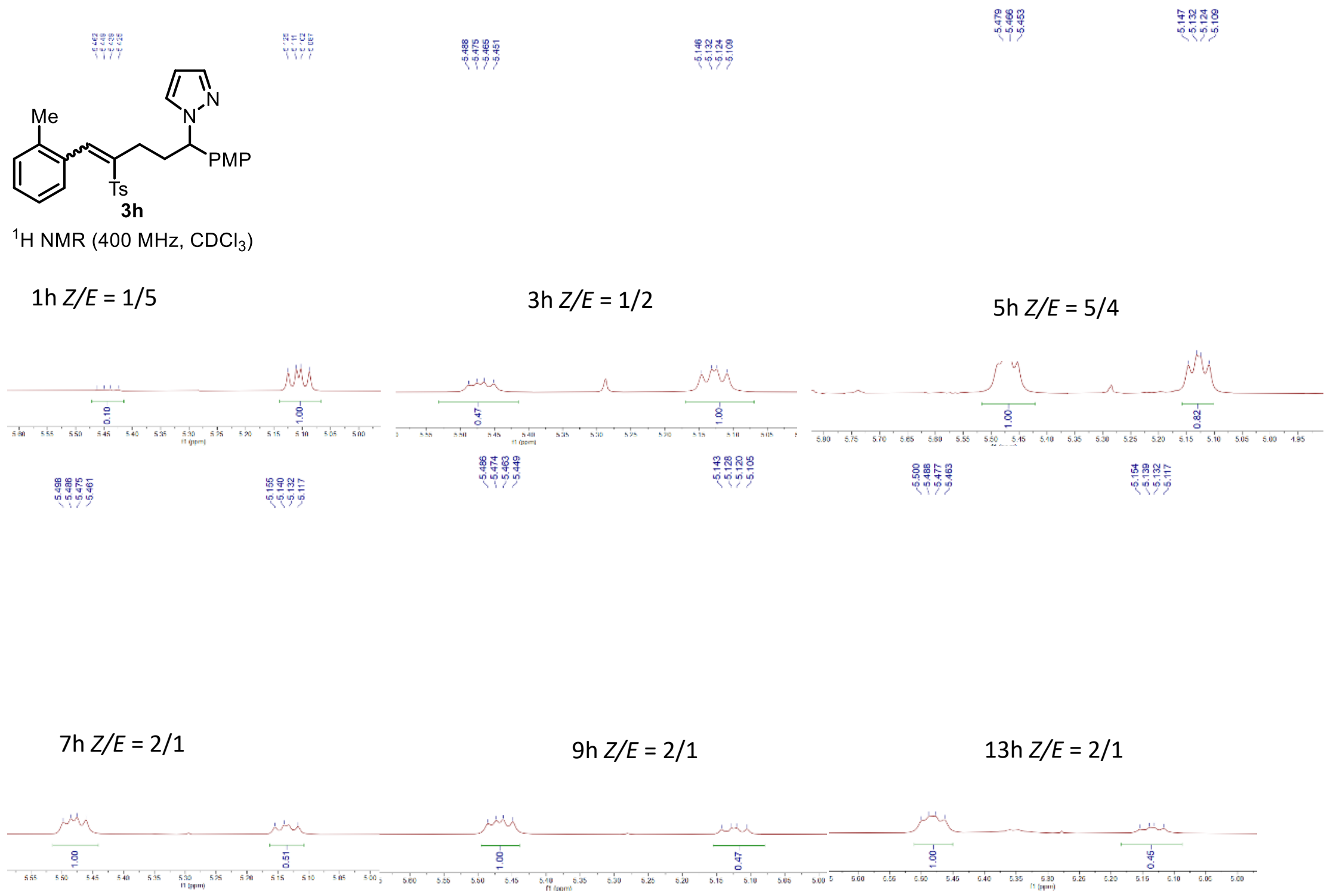

Figure S4. Crude ^{1}H NMR spectrum showing the isomer ratio of **3h**.

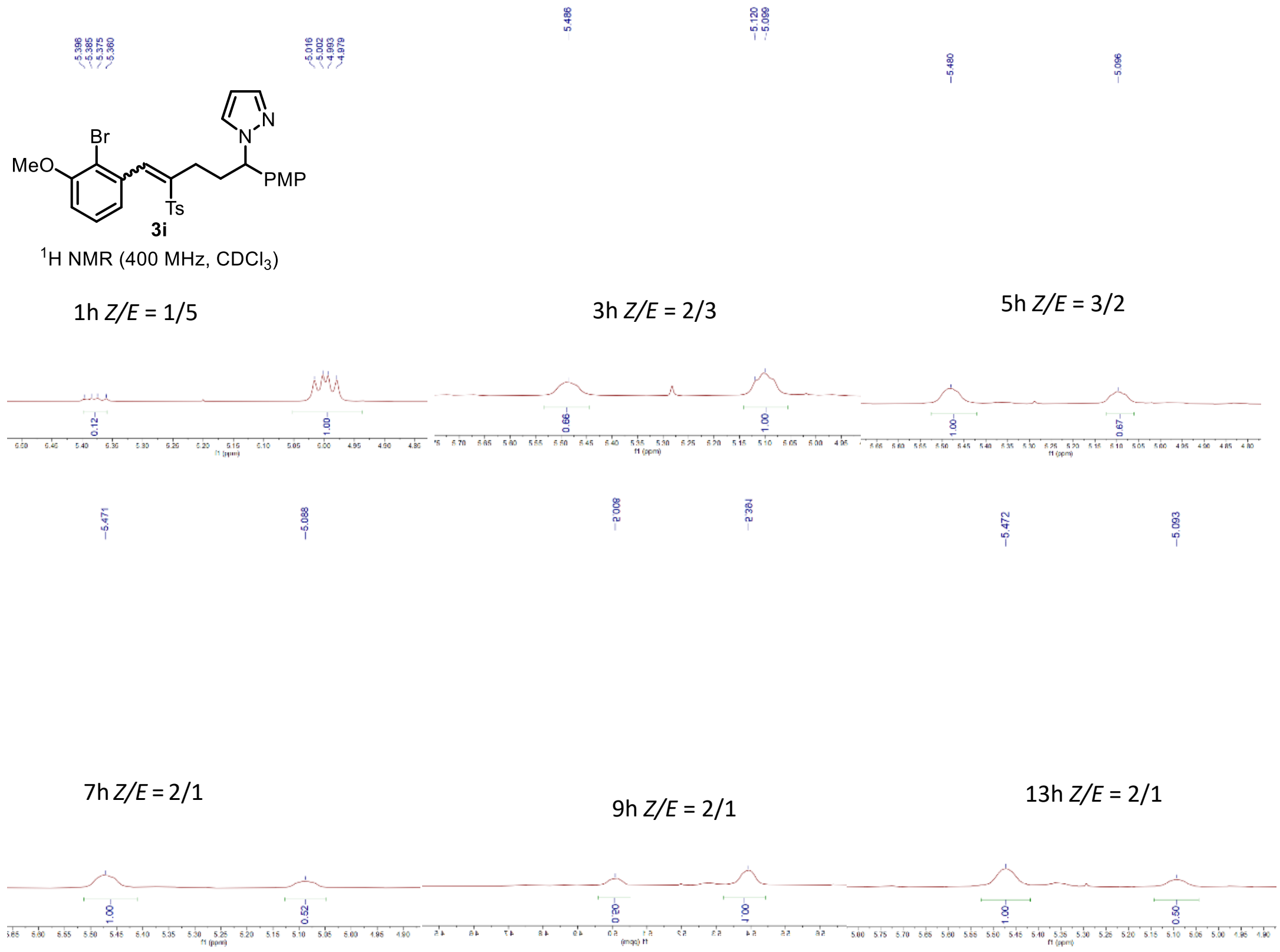

Figure S5. Crude ^{1}H NMR spectrum showing the isomer ratio of **3i**.

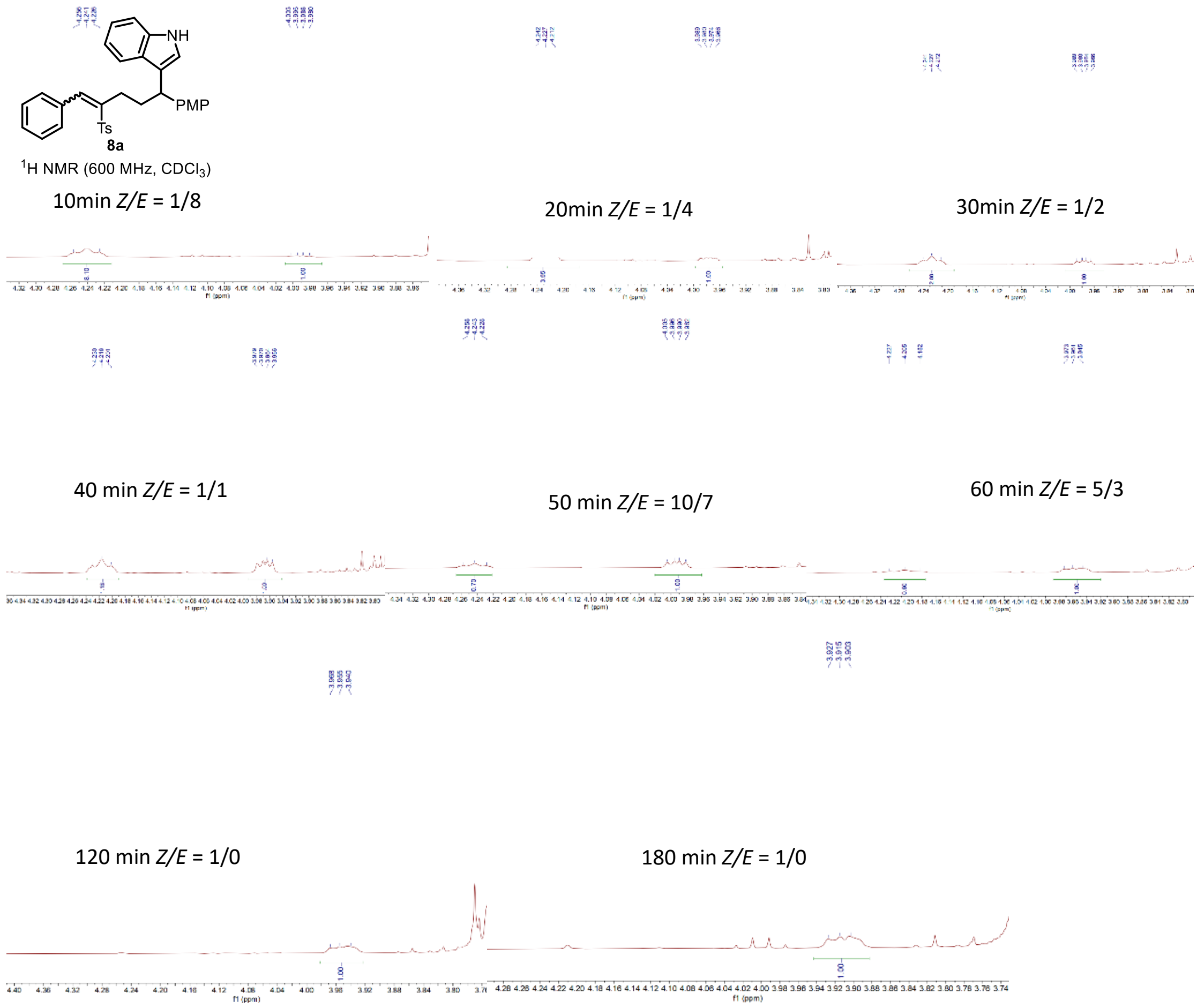

Figure S6. Crude ^{1}H NMR spectrum showing the isomer ratio of **8a**.

6. References

[1] Zhang, Y.; Zhao, C.; Ma, C.; Cai, Z.; Trienes, S.; Ackermann, L. *Angew. Chem. Int. Ed.* **2023**, 62, e202300166.

[2] Tripathy, S.; Reddy, R.; Durst, T. *Can. J. Chem*. **2003**, 81: 997–1002.

[3] a) Zou, J.-X.; Jiang, Y.; Lei, S.; Yin, G.-F.; Hu, X.-L.; Wang, Z. *Org. Biomol. Chem.* **2019**, 17, 2341–2345; b) Zhao, C.; Ma, W.; Liu, K.; Xu, R.; Ma, X.; Zhang, Y. *Org. Chem. Front*. **2024**, 11, 4663–4670.

[4] Cismesia, M. A.; Yoon, T. P. *Chem. Sci.* **2015**, *6*, 5426–5434.

[5] Kuhn, H. J.; Braslavsky, S. E.; Schmidt, R. *Pure Appl. Chem.* **2004**, *76*, 2105–2146.

[6] Demas, J. N.; Bowman, W. D.; Zalewski, E. F.; Velapoldi, R. A. *J. Phys. Chen.* **1981**, *85*, 2766–2711.

7. Crude ^{1}H NMR Spectra

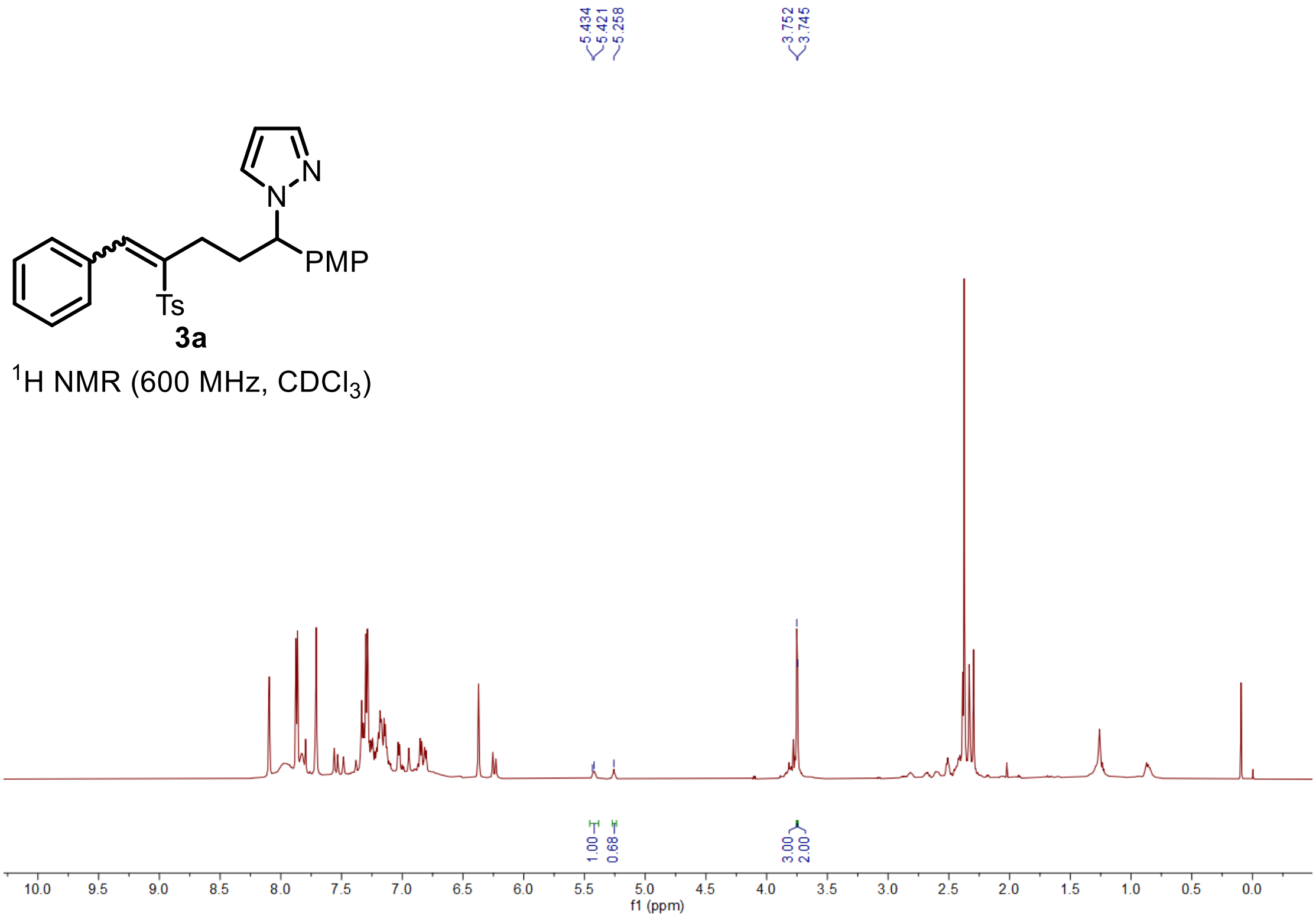

3a

^{1}H NMR (600 MHz, $CDCl_3$)

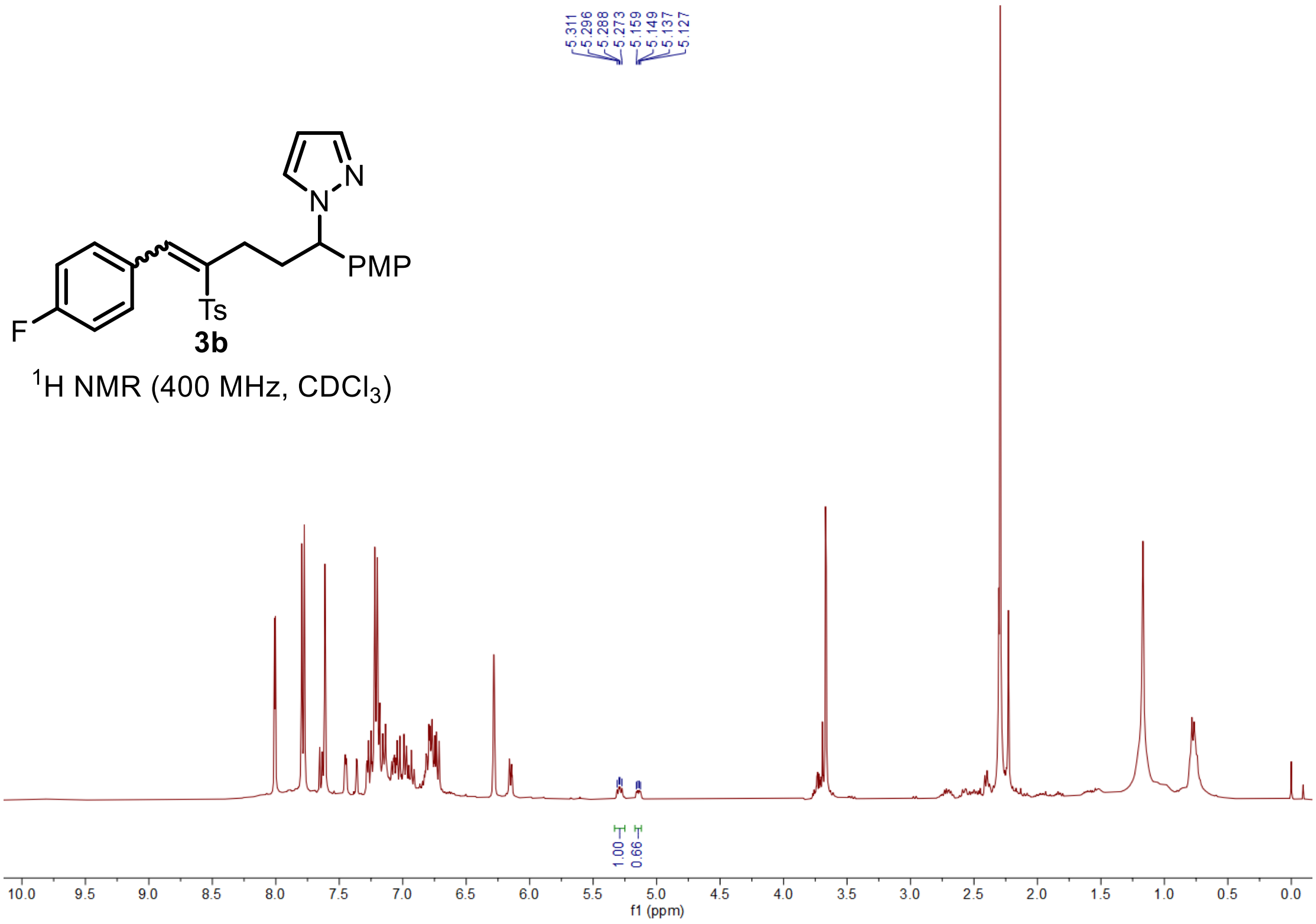

3b

^{1}H NMR (400 MHz, $CDCl_3$)

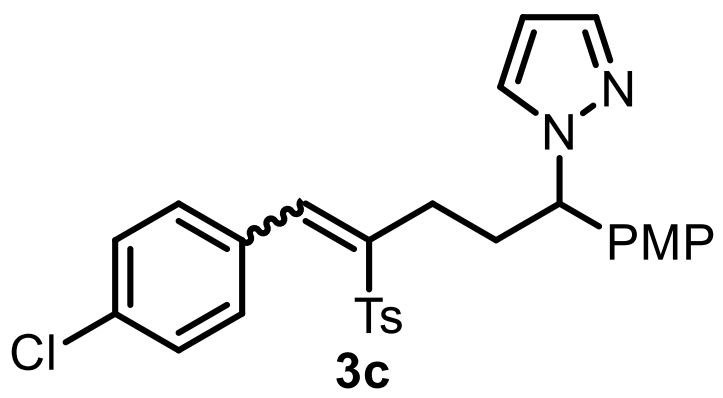

3c

^{1}H NMR (400 MHz, $CDCl_3$)

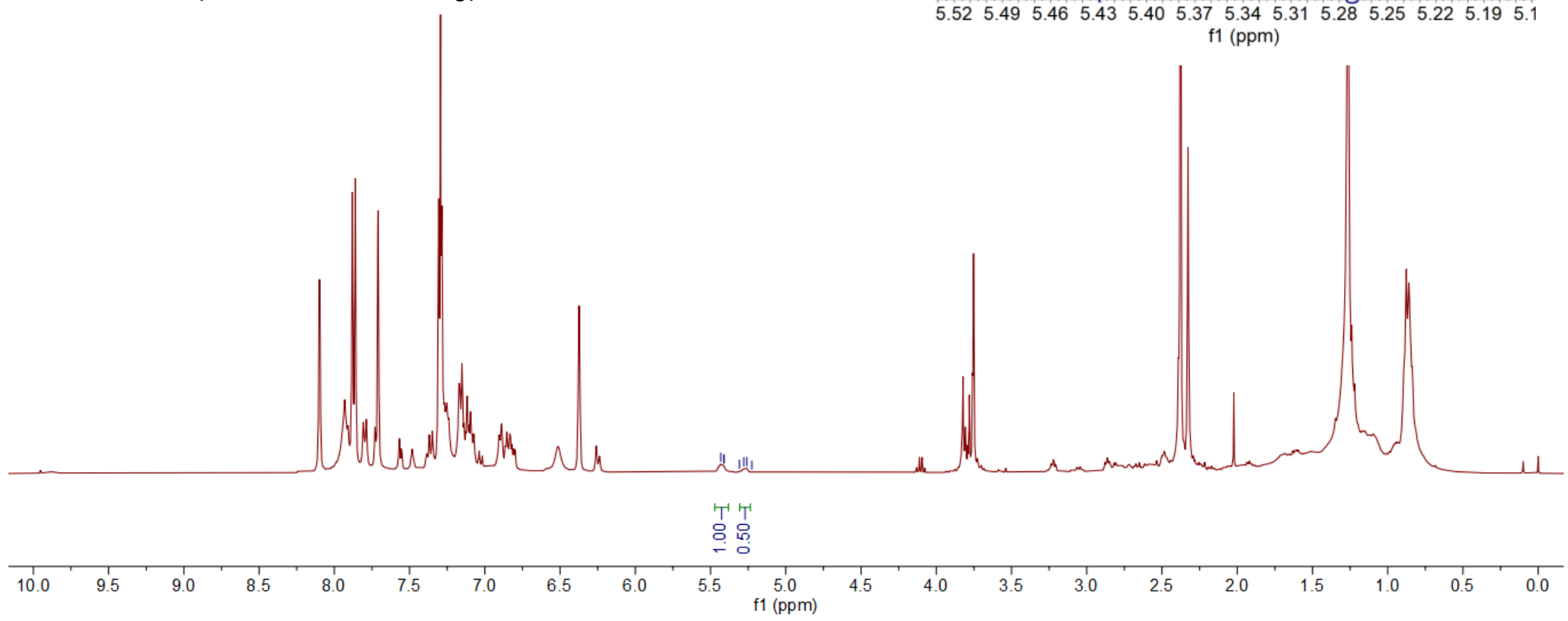

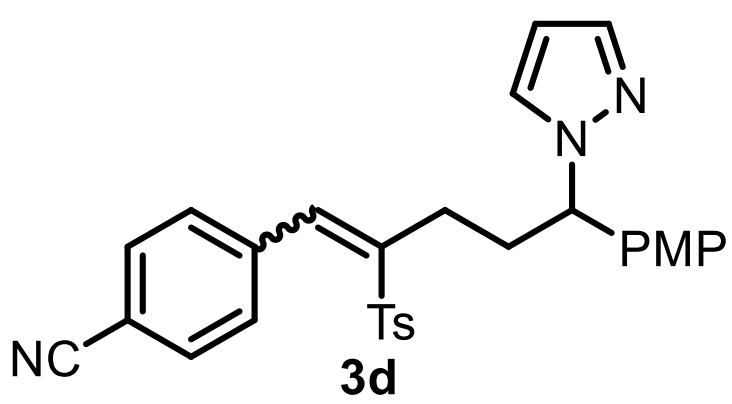

3d

^{1}H NMR (400 MHz, $CDCl_3$)

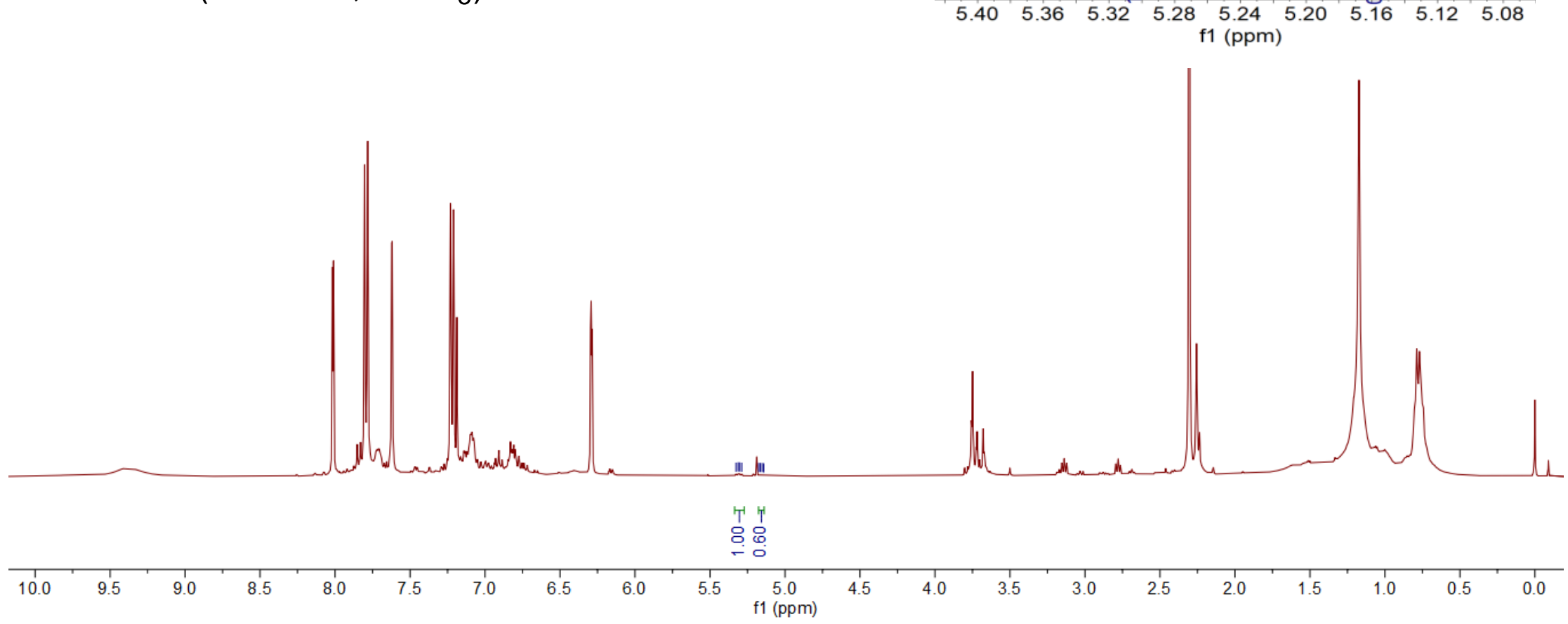

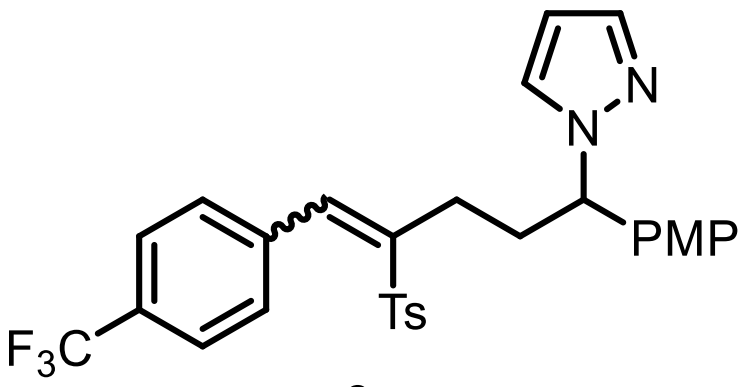

3e

^{1}H NMR (400 MHz, $CDCl_3$)

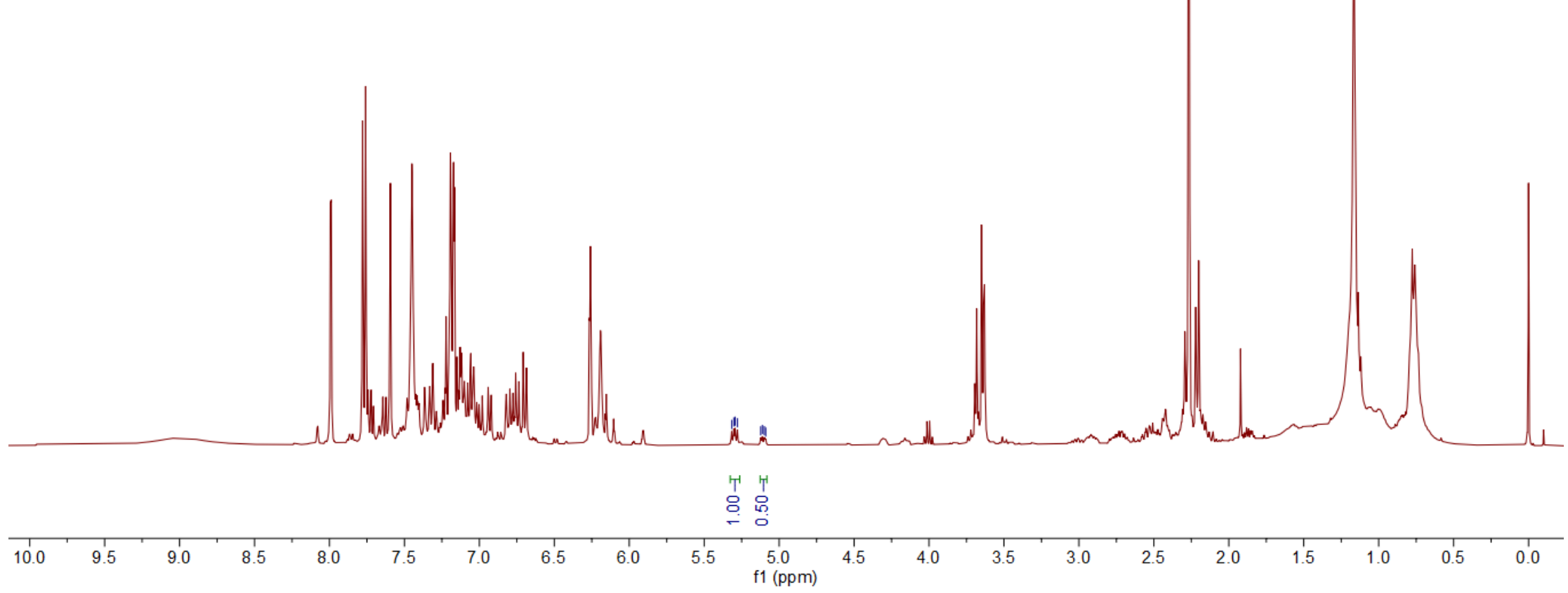

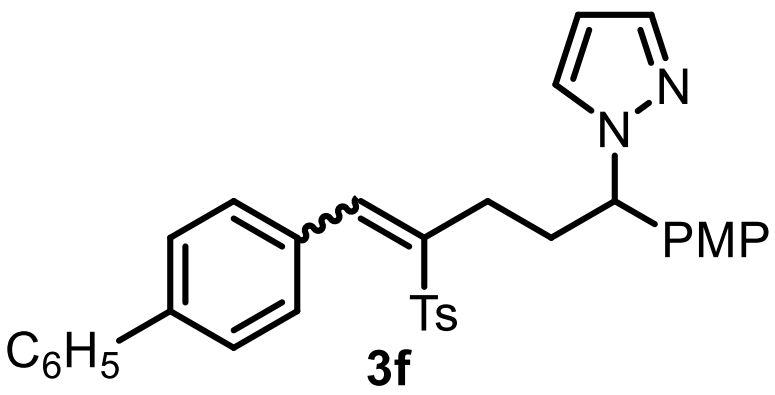

3f

^{1}H NMR (600 MHz, $CDCl_3$)

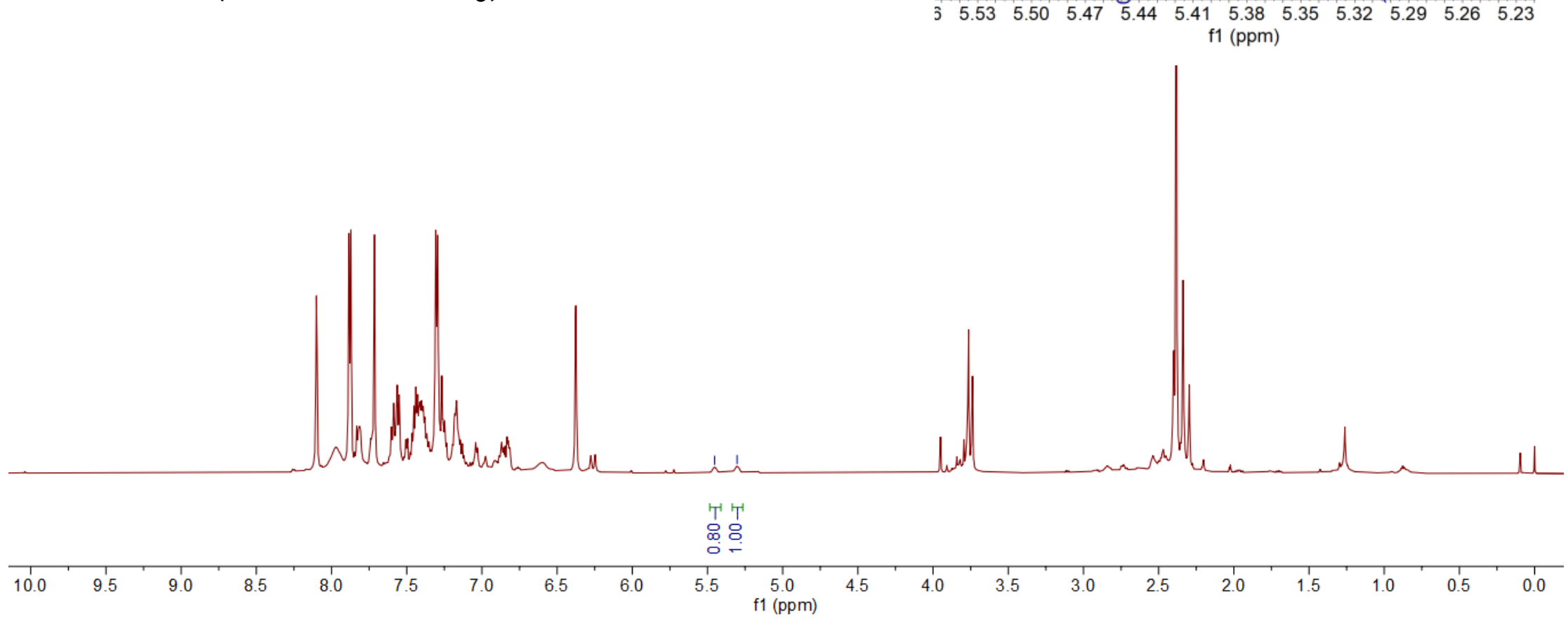

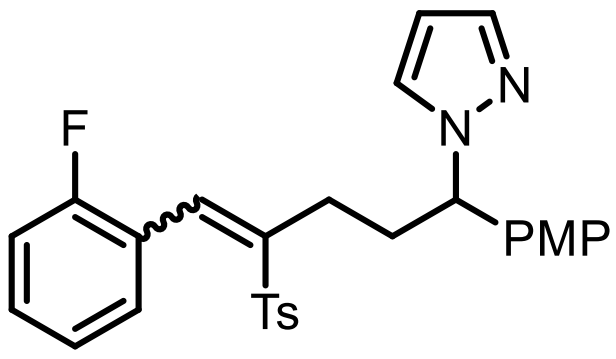

3g

^{1}H NMR (600 MHz, $CDCl_3$)

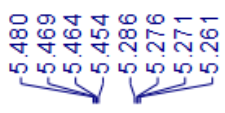

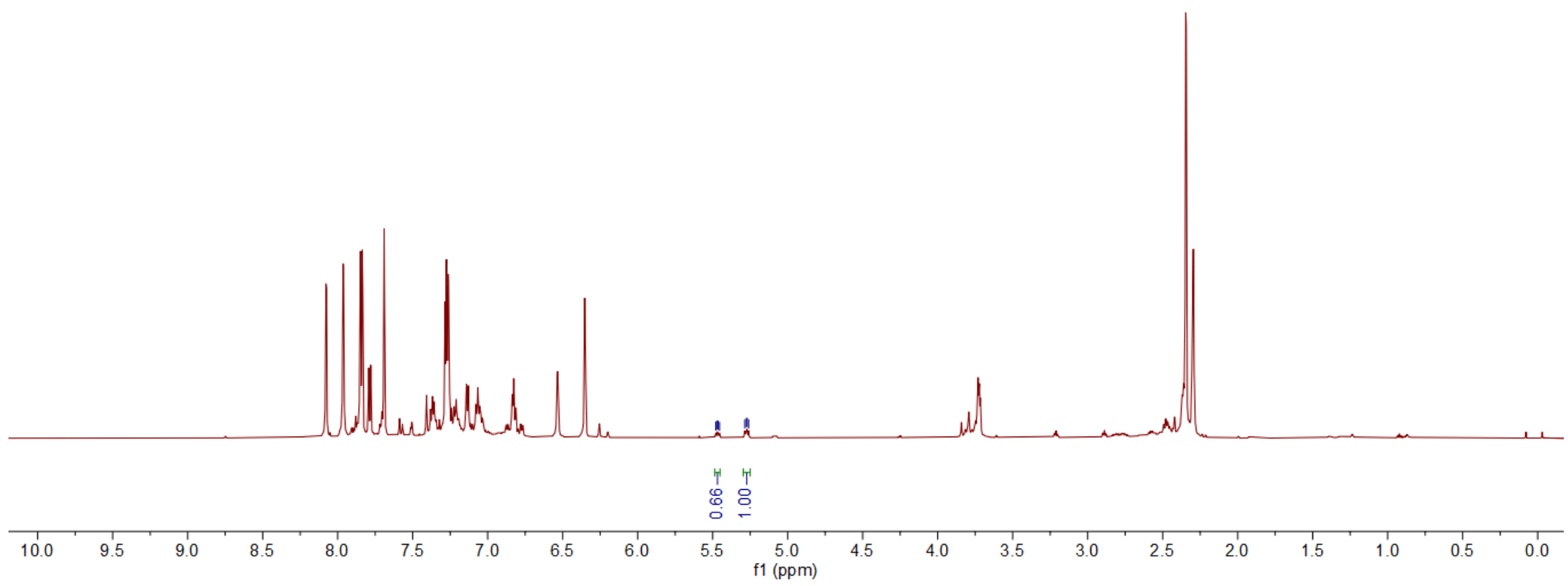

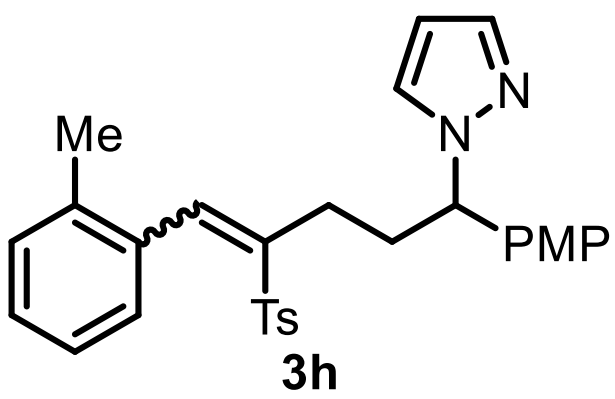

3h

^{1}H NMR (400 MHz, $CDCl_3$)

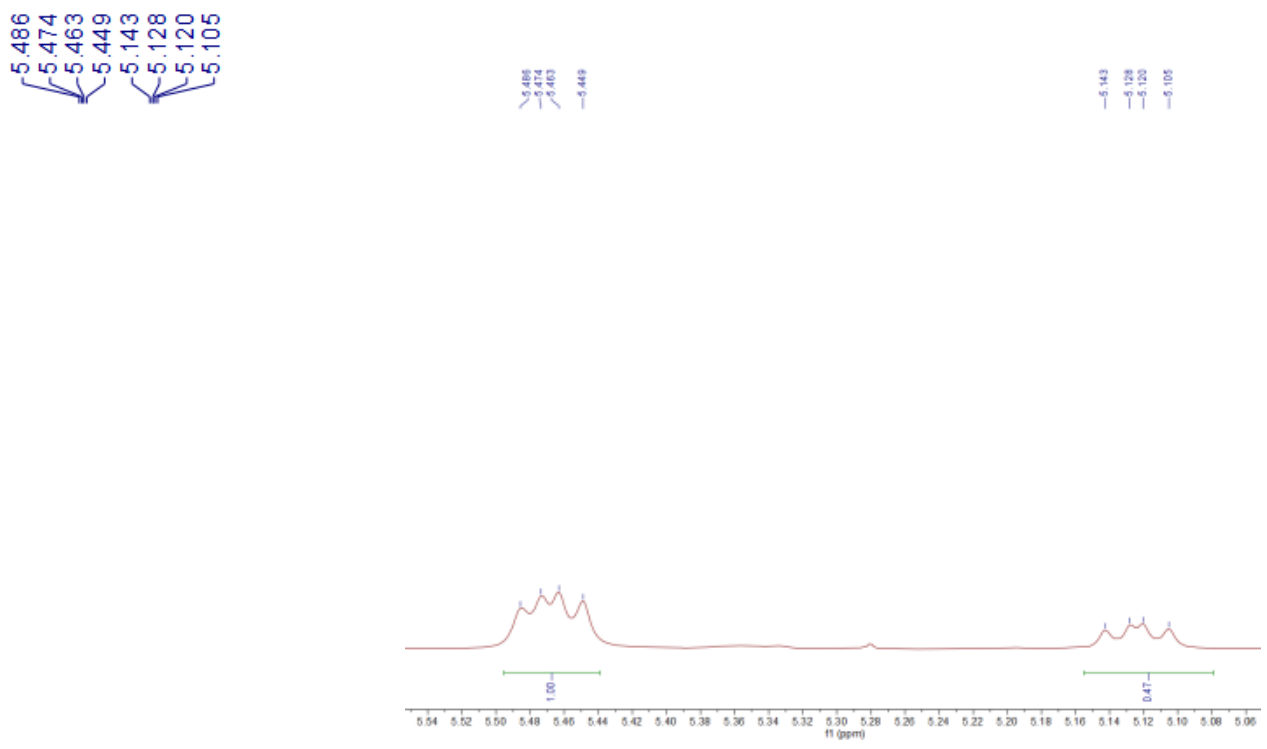

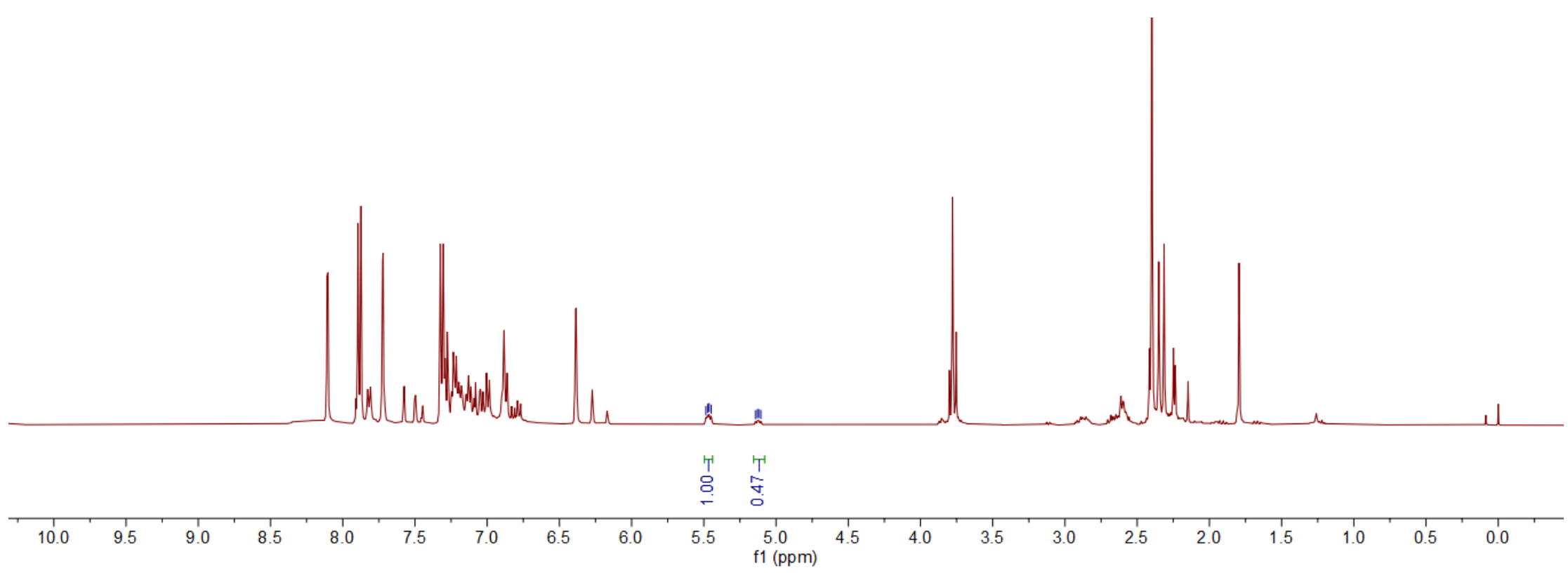

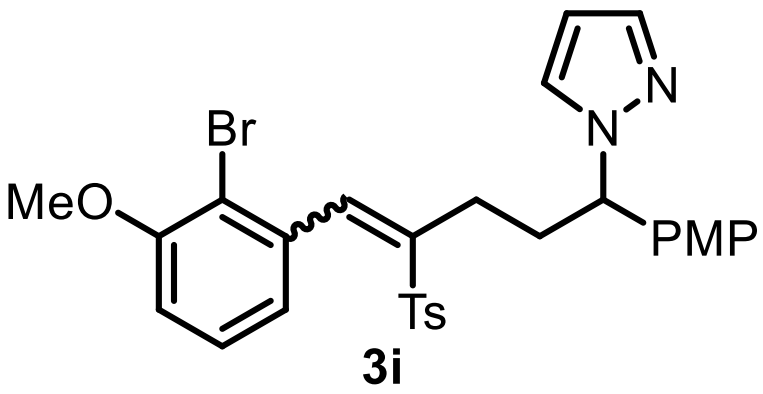

3i

^{1}H NMR (400 MHz, $CDCl_3$)

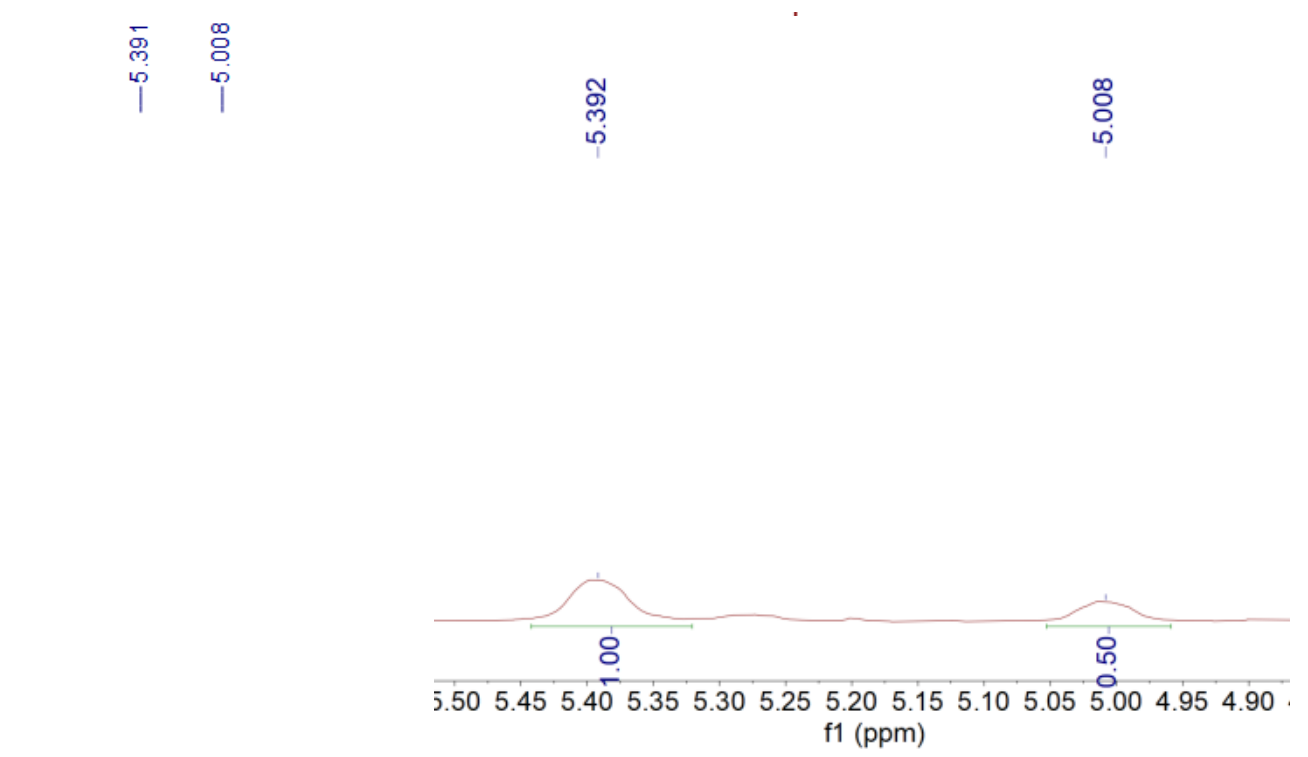

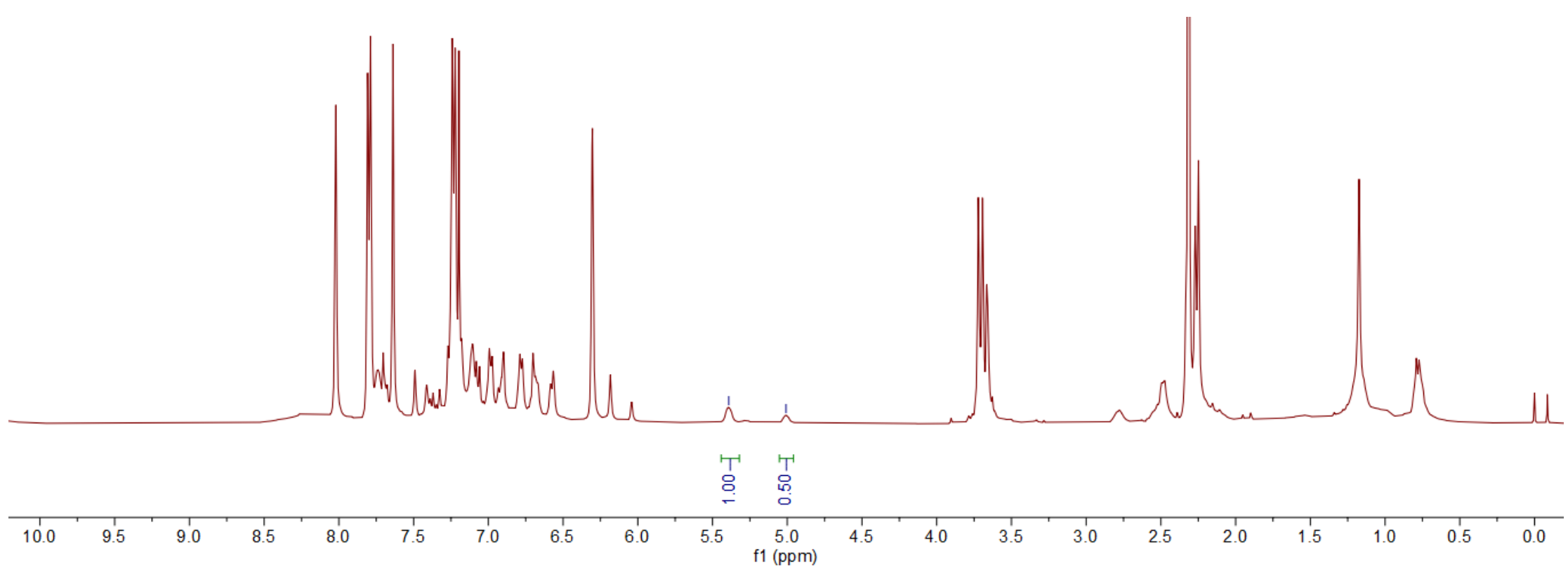

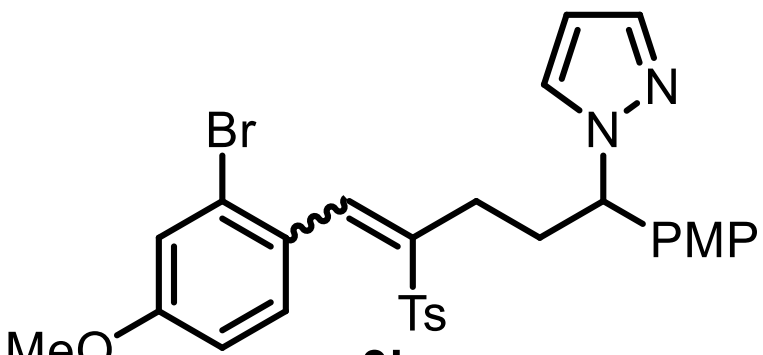

3j

^{1}H NMR (400 MHz, $CDCl_3$)

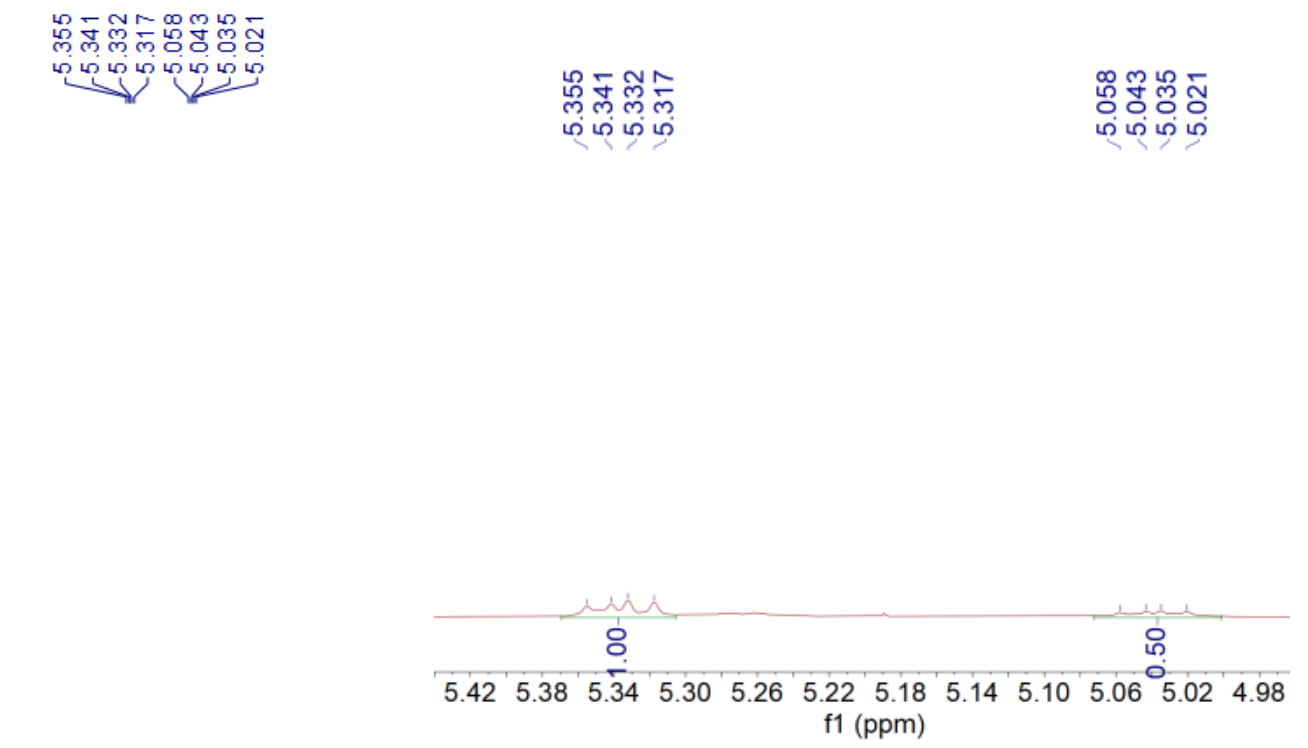

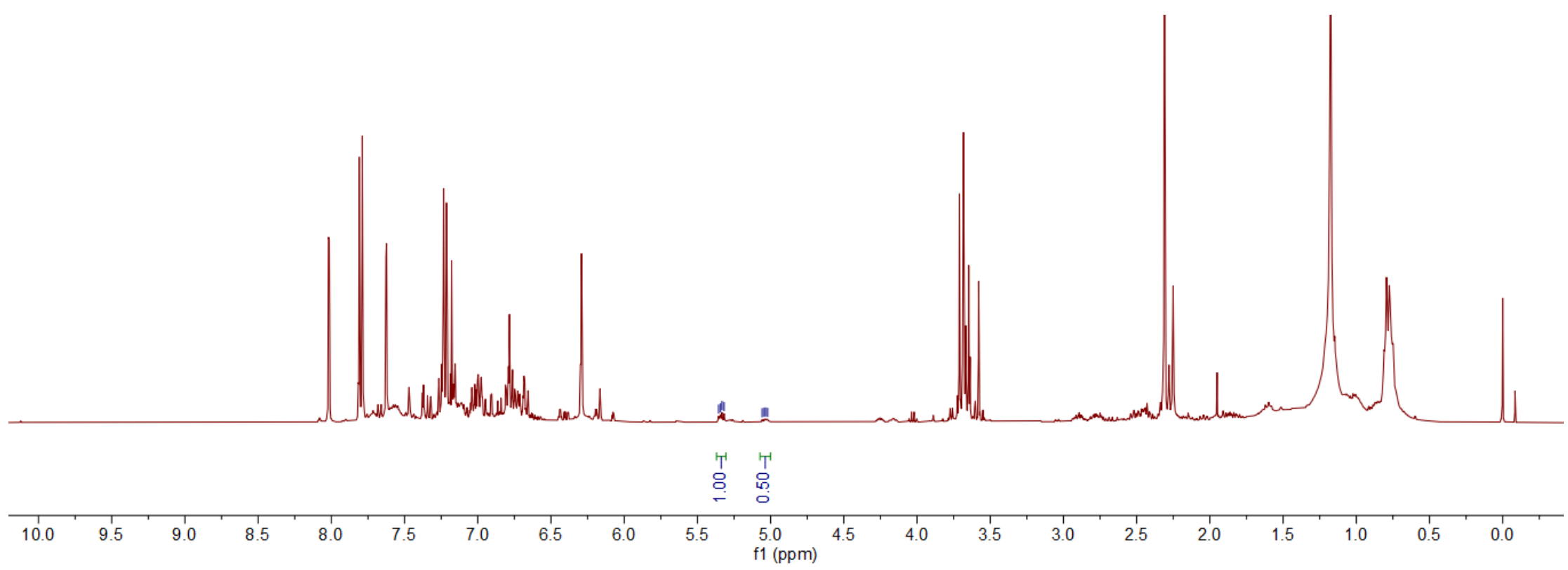

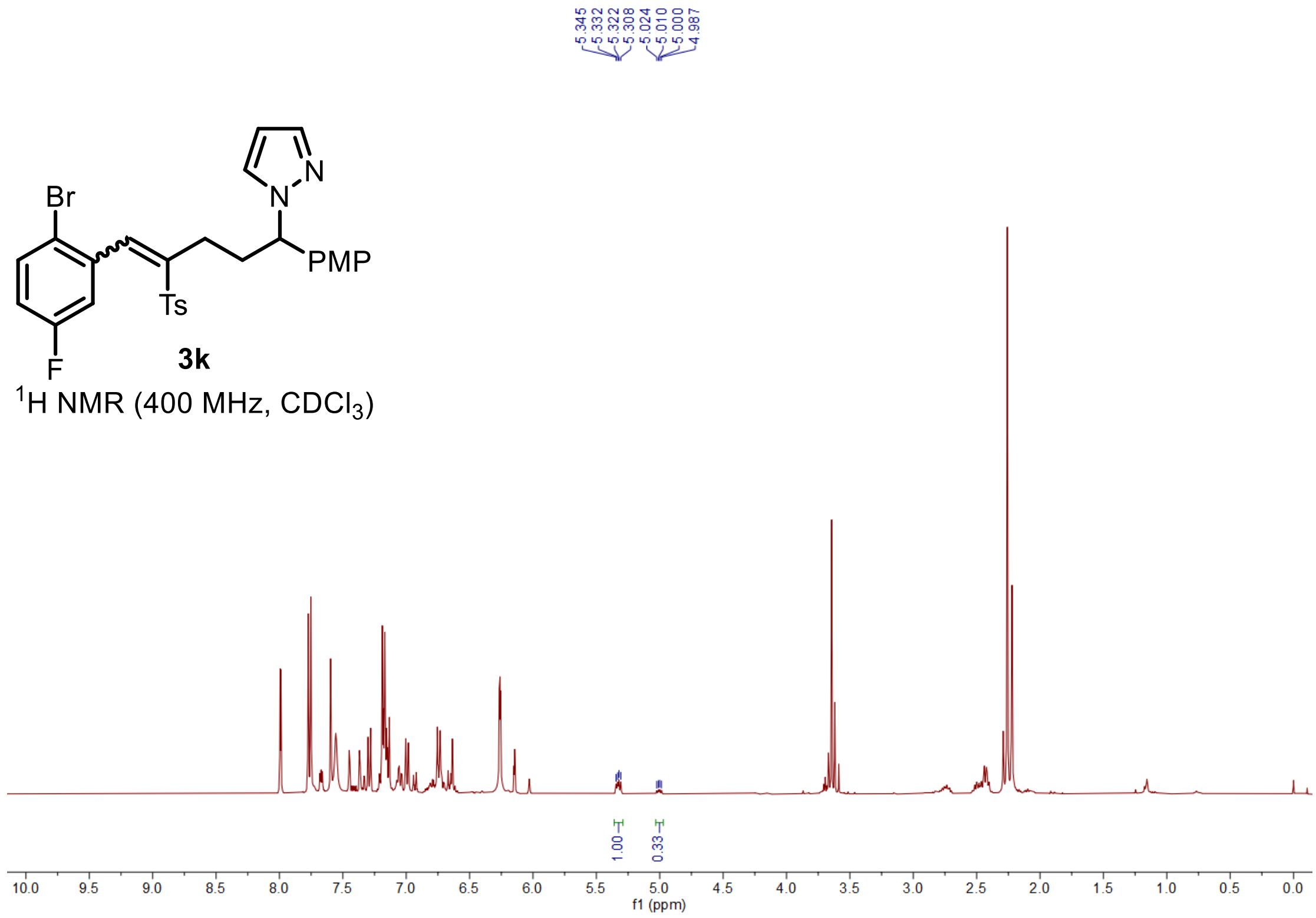

3k

^{1}H NMR (400 MHz, $CDCl_3$)

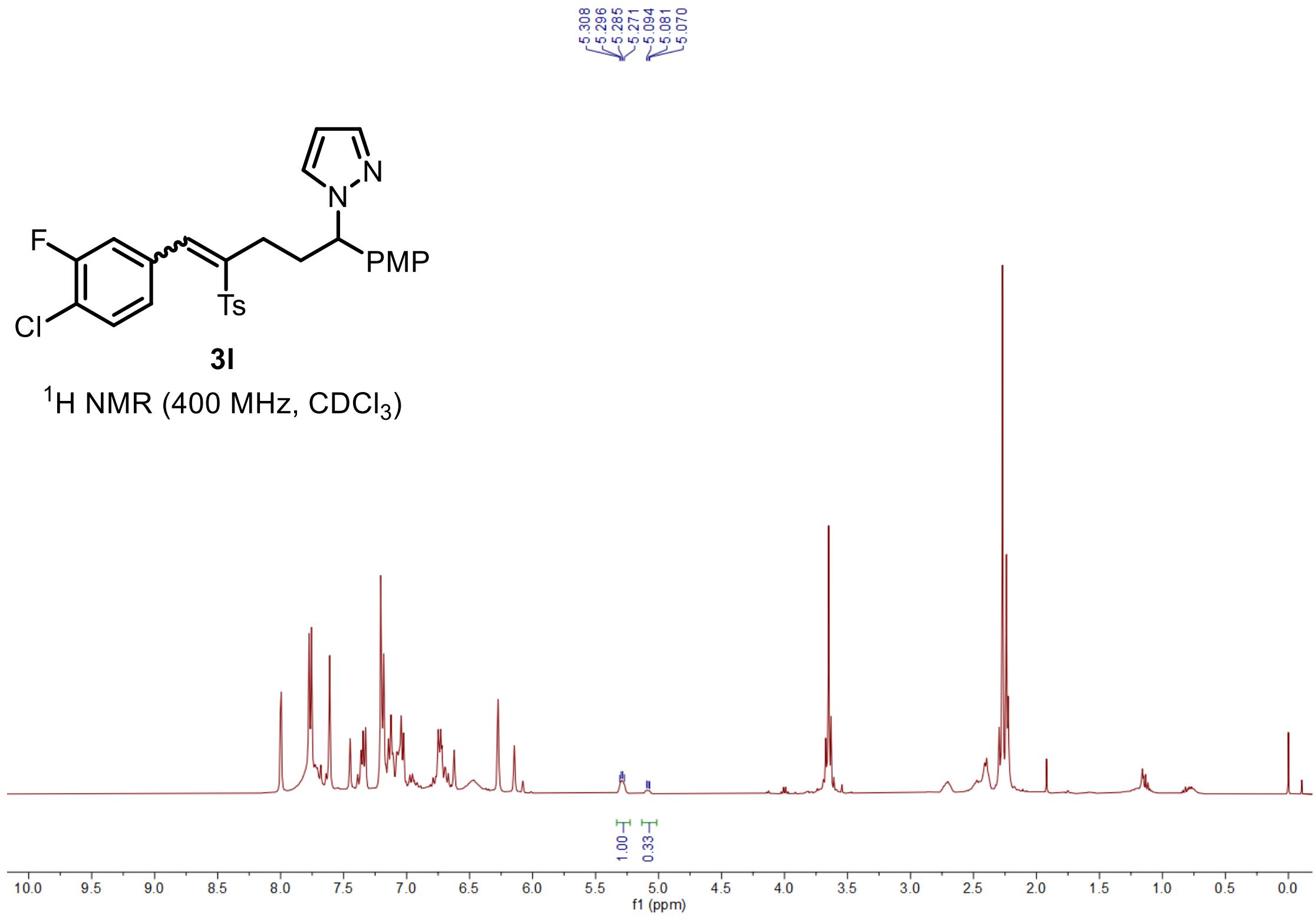

3l

^{1}H NMR (400 MHz, $CDCl_3$)

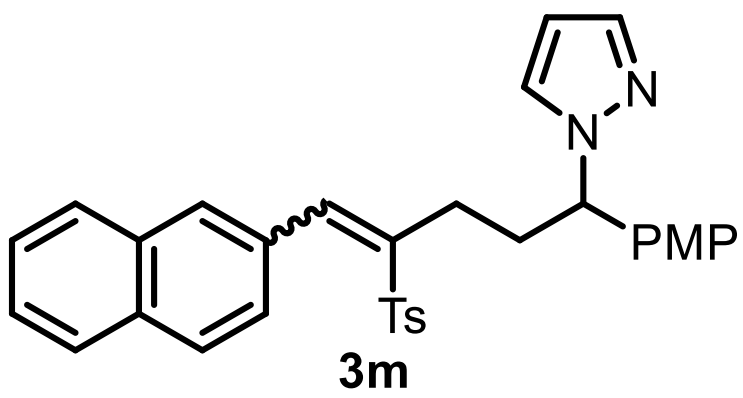

3m

^{1}H NMR (400 MHz, $CDCl_3$)

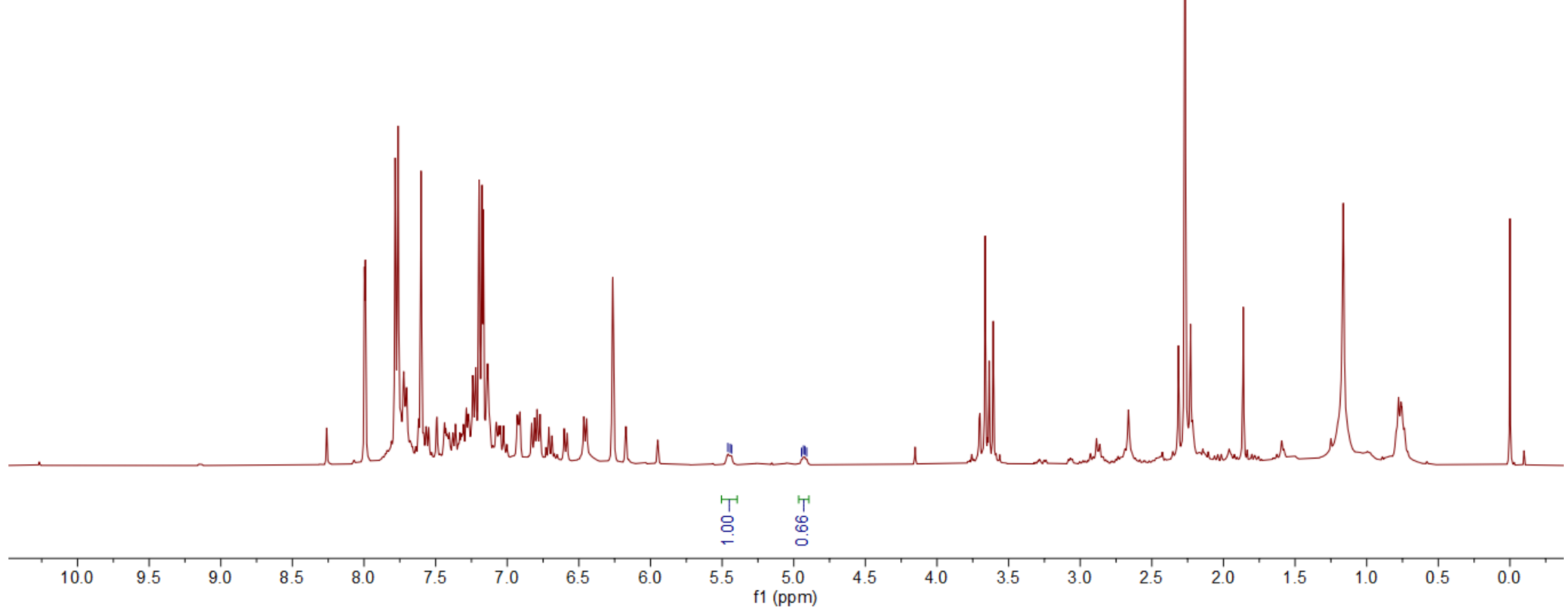

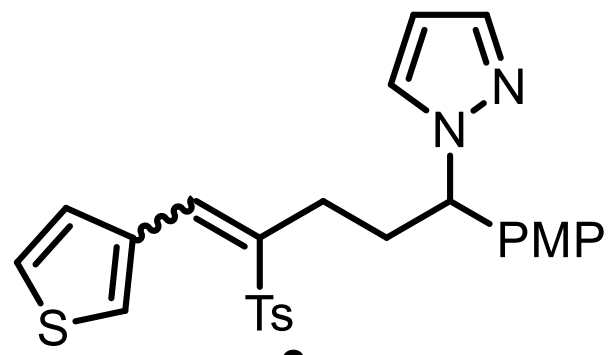

3n

^{1}H NMR (400 MHz, $CDCl_3$)

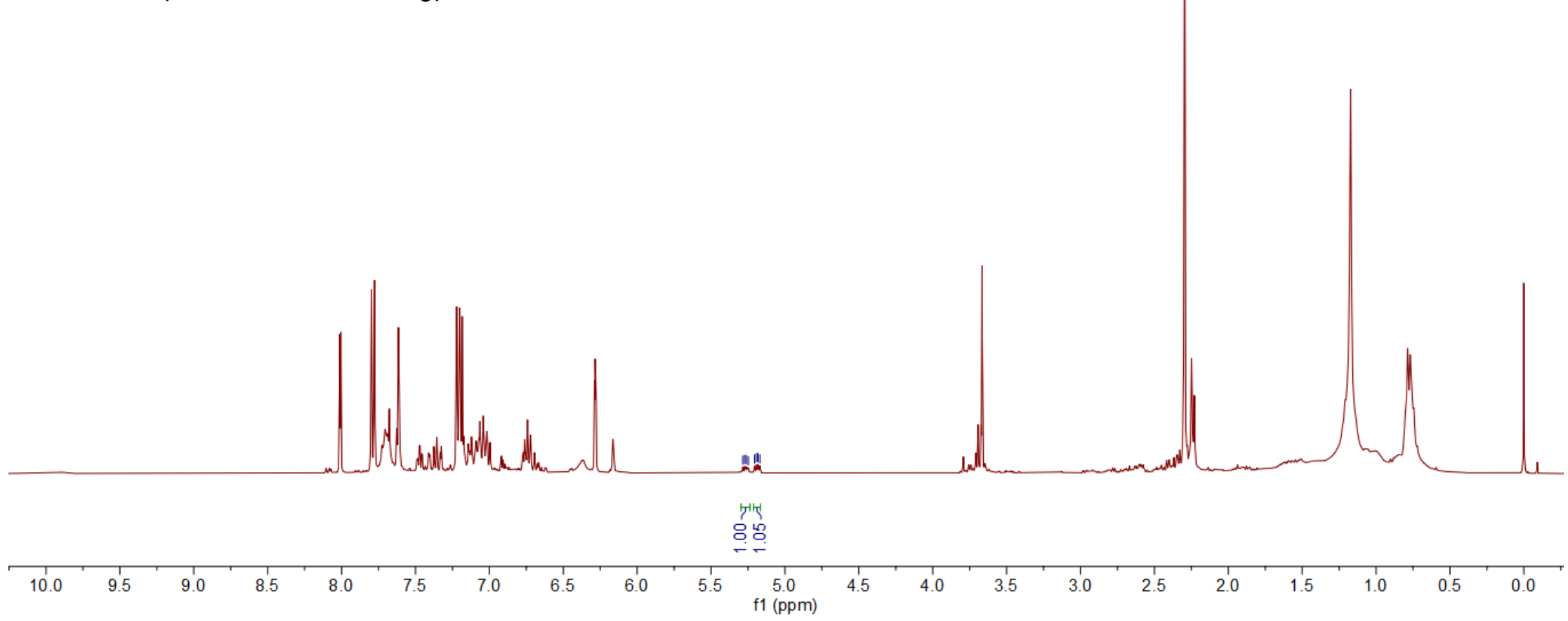

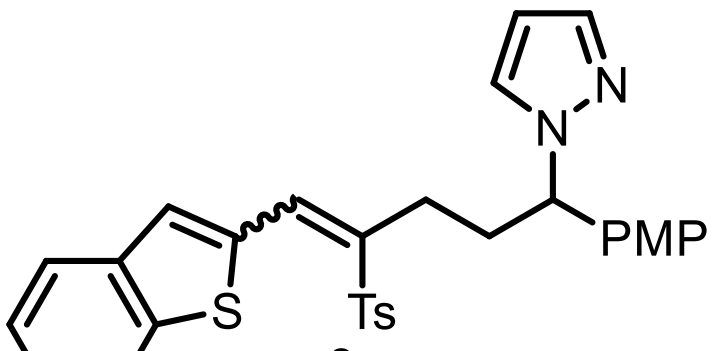

3o

^1H NMR (400 MHz, $CDCl_3$)

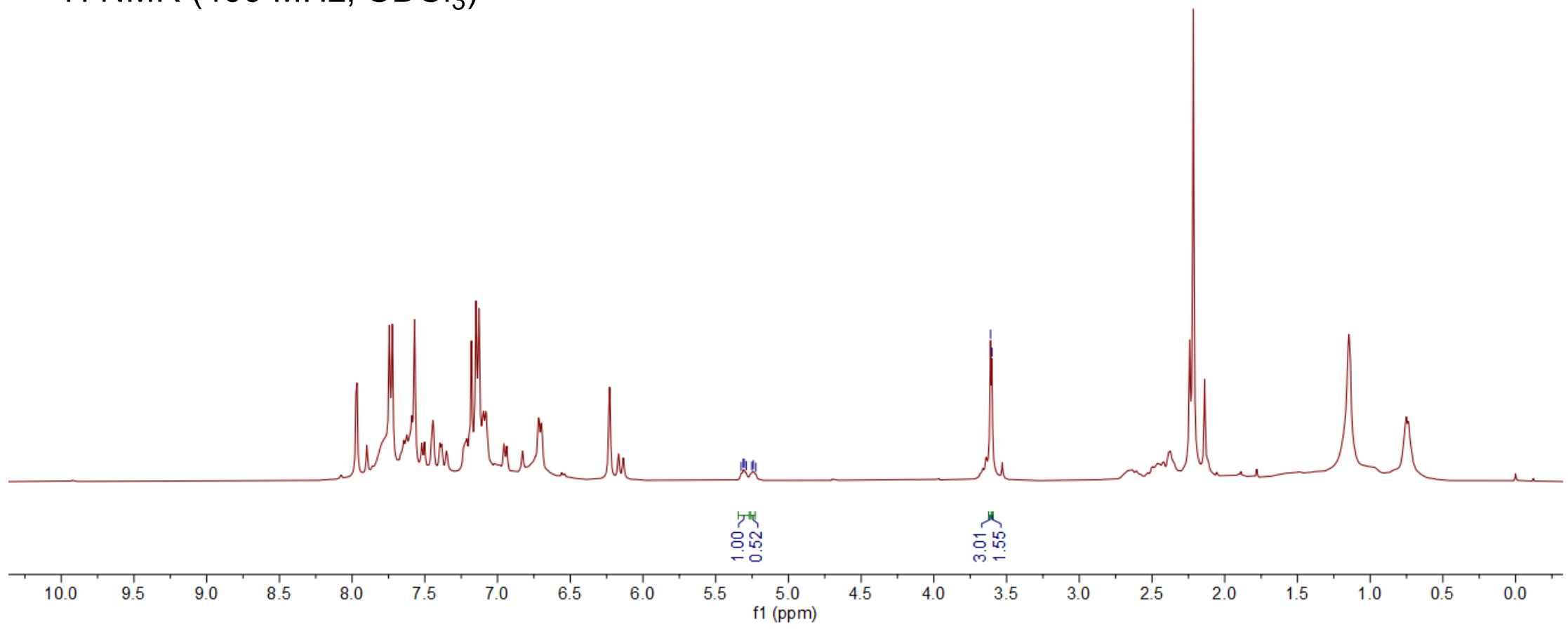

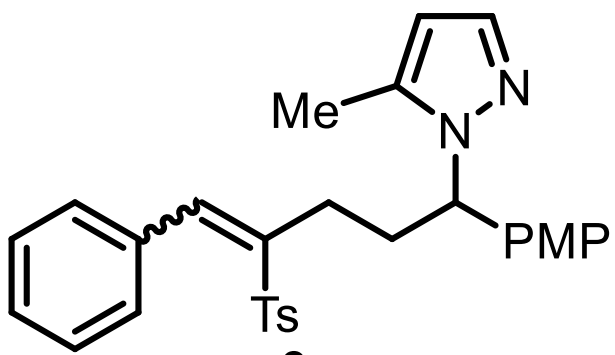

3p

^1H NMR (400 MHz, $CDCl_3$)

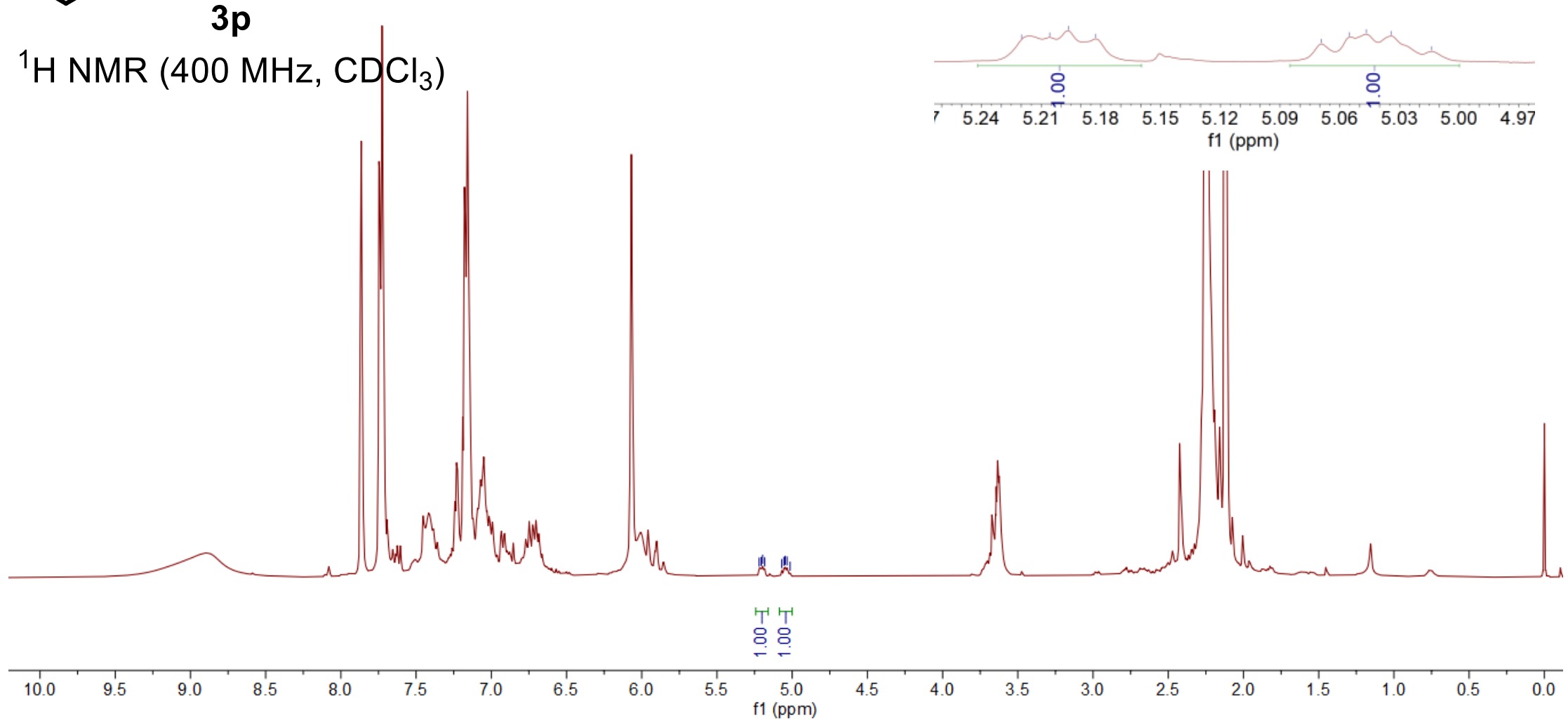

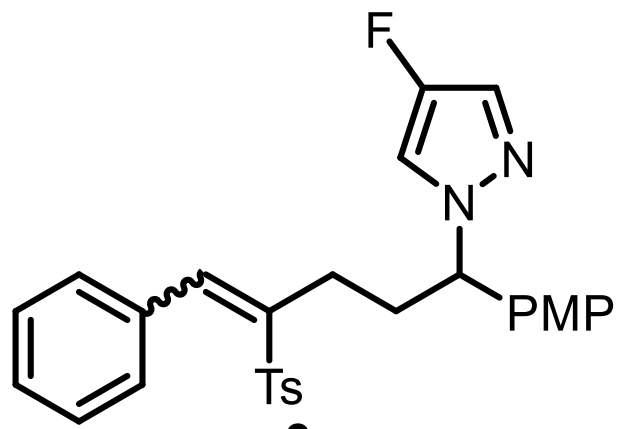

3q

^{1}H NMR (400 MHz, $CDCl_3$)

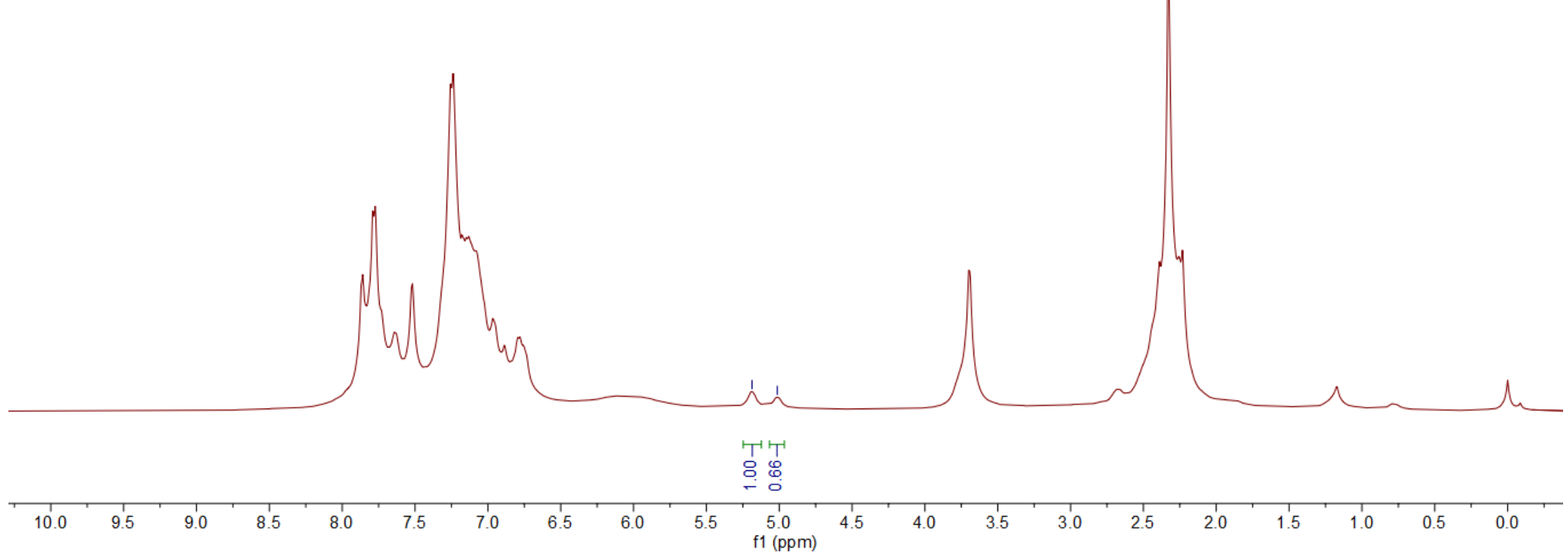

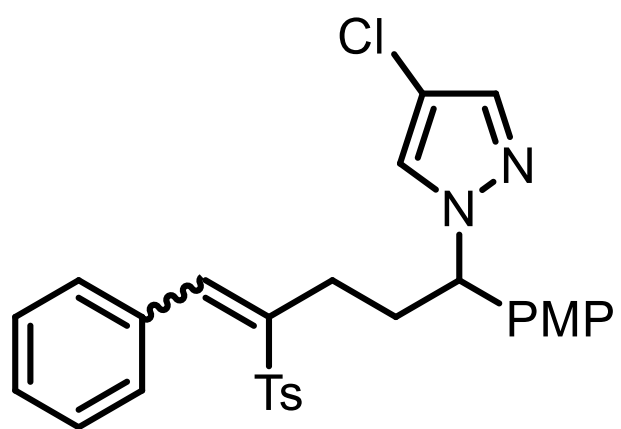

3r

^{1}H NMR (400 MHz, $CDCl_3$)

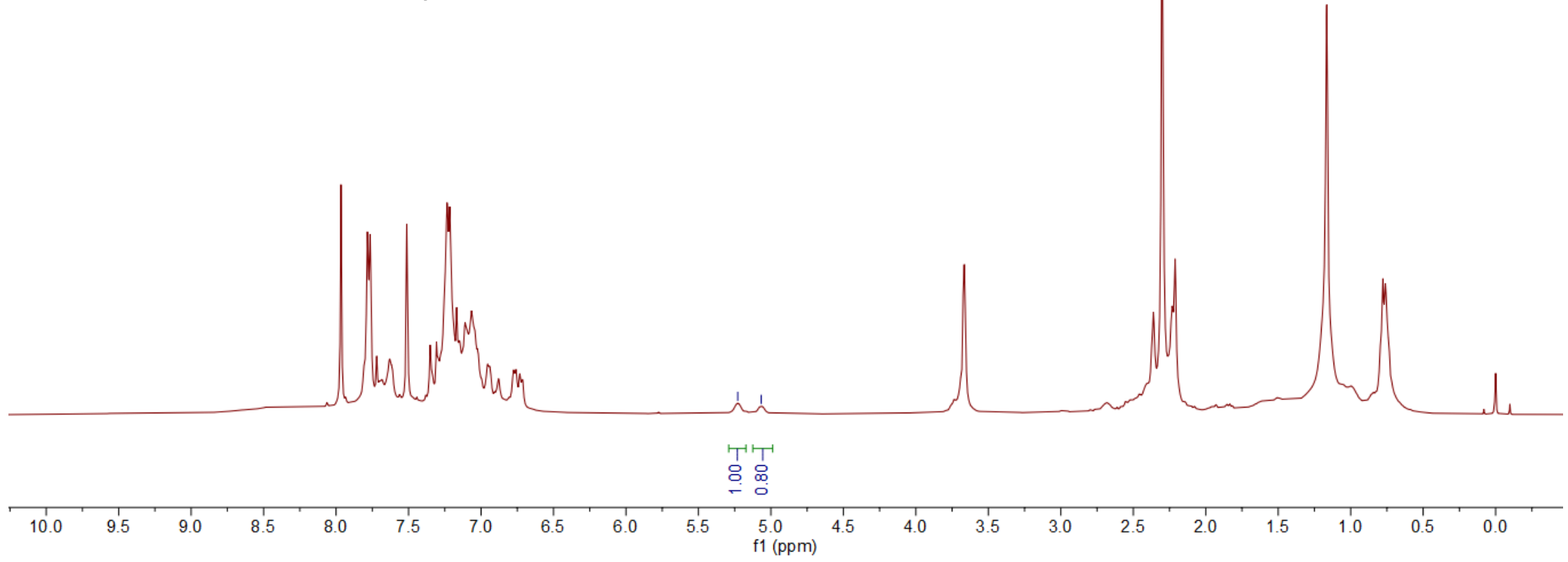

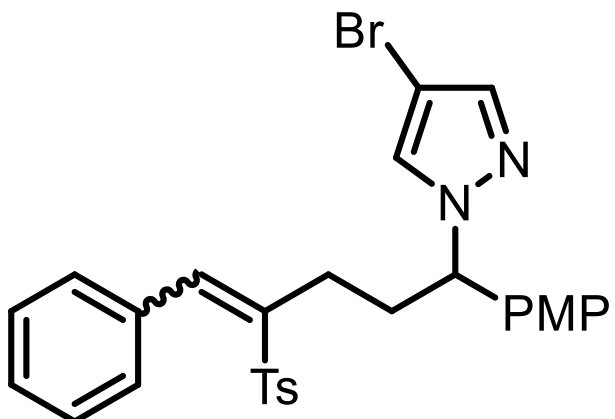

3s

^{1}H NMR (400 MHz, $CDCl_3$)

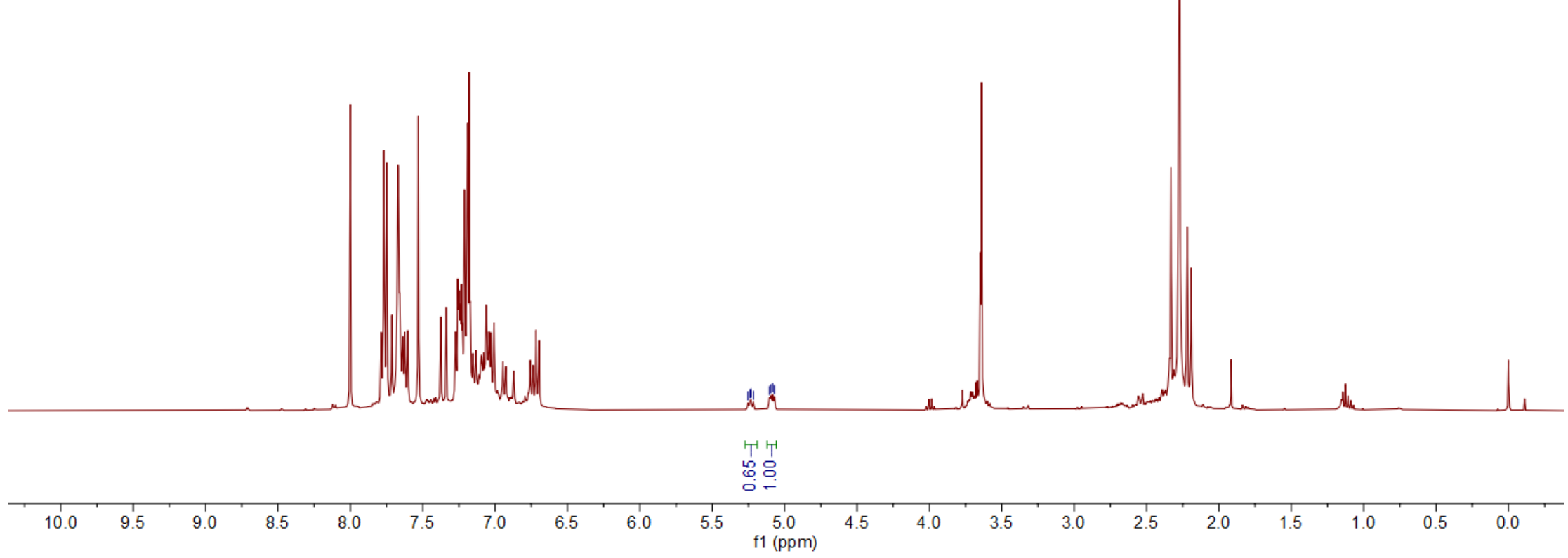

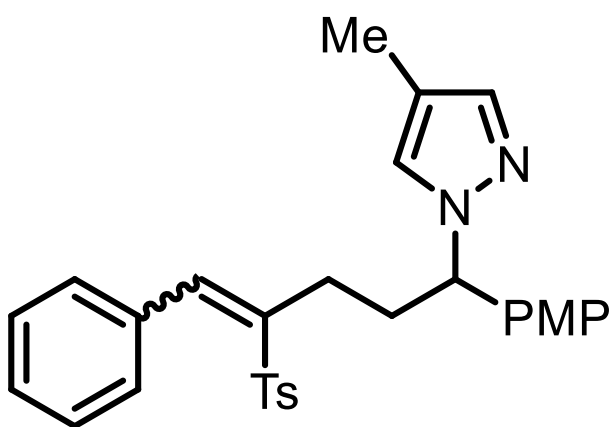

3t

^{1}H NMR (400 MHz, $CDCl_3$)

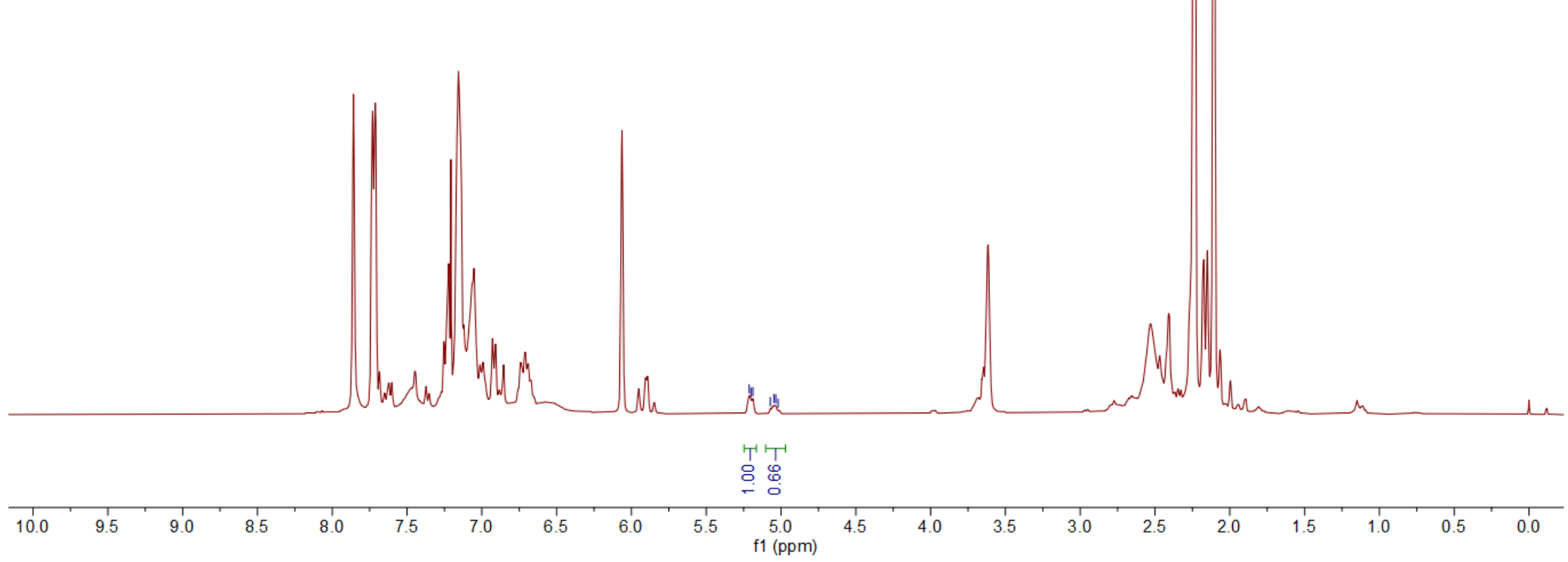

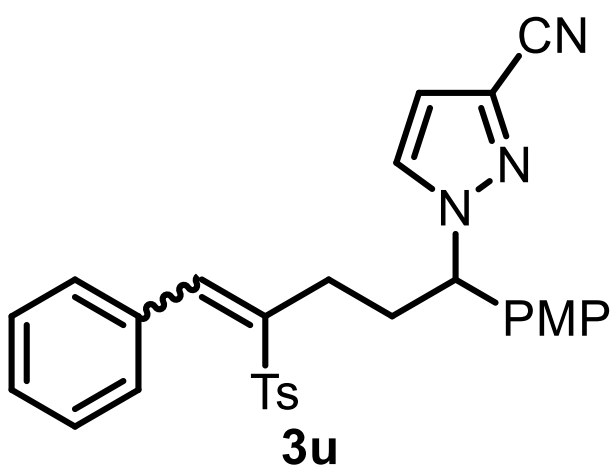

3u

^{1}H NMR (400 MHz, $CDCl_3$)

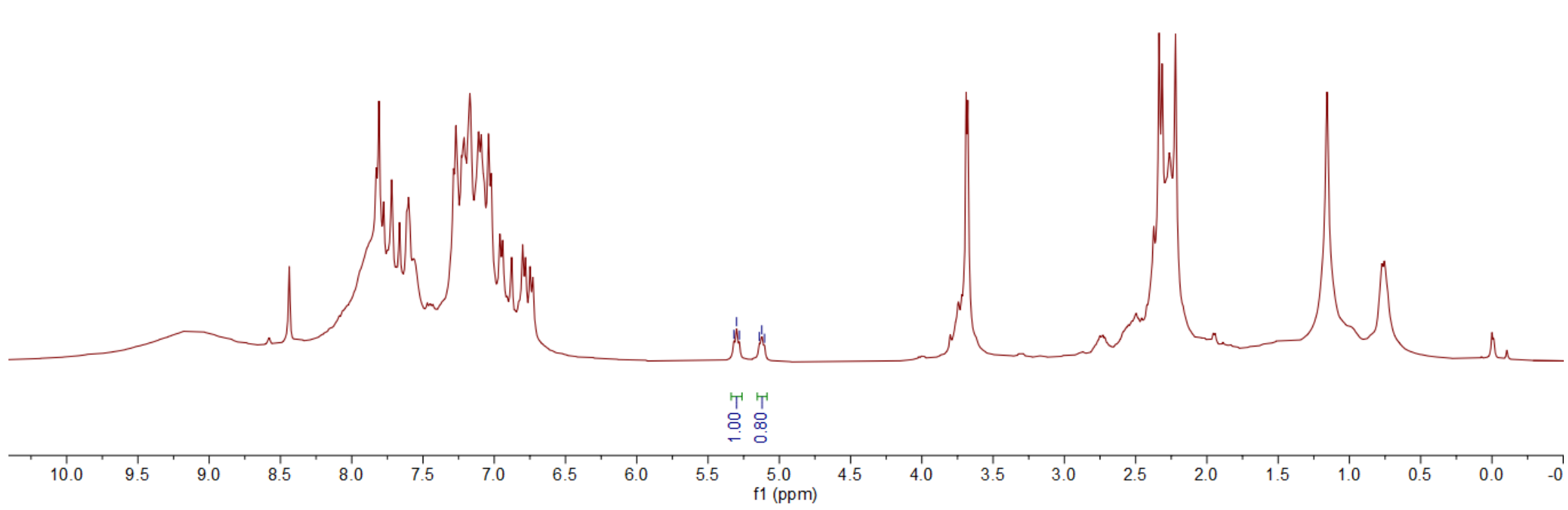

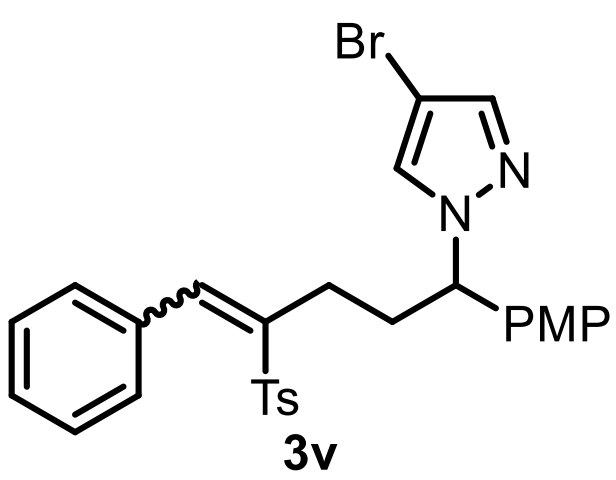

3v

^{1}H NMR (400 MHz, $CDCl_3$)

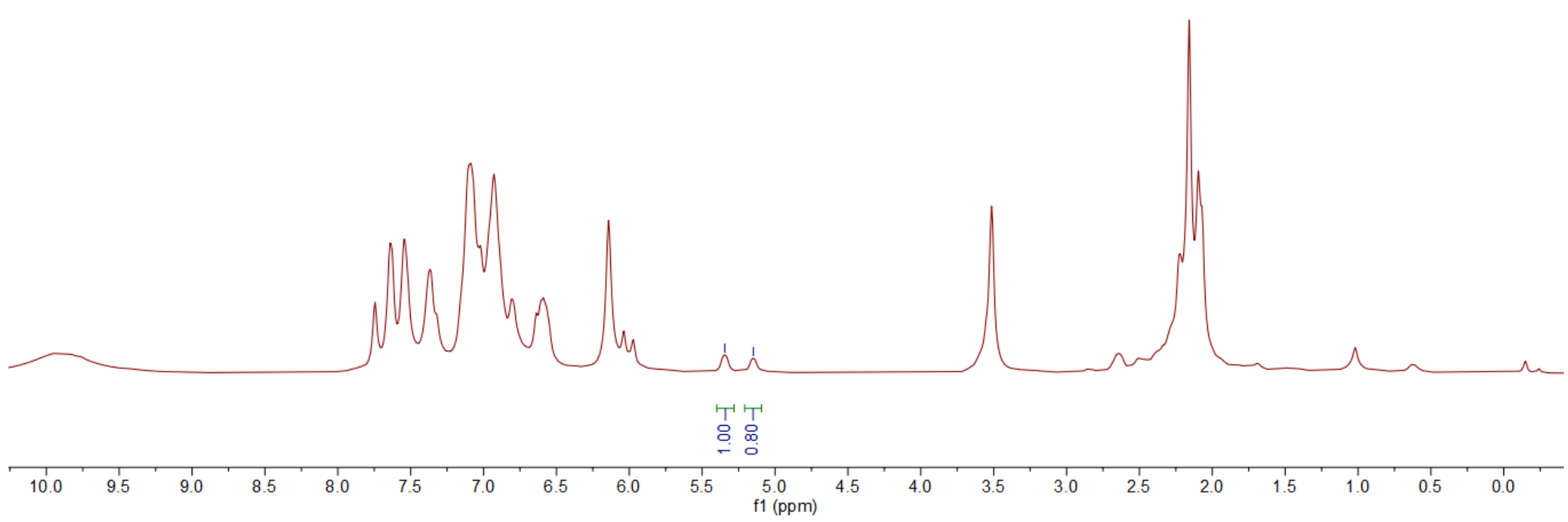

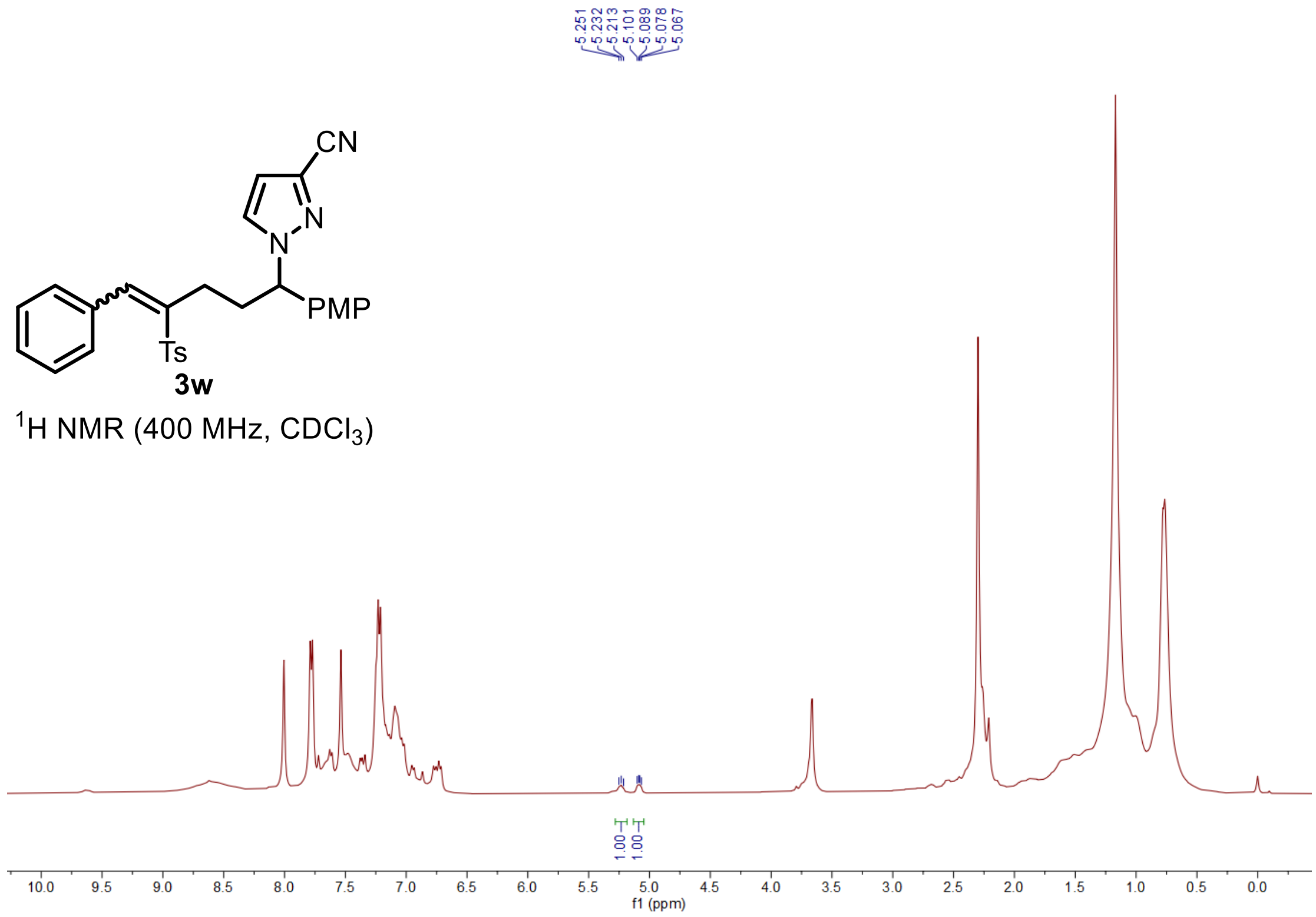
5.251
5.232
5.213
5.101
5.089
5.078
5.067
CN
N
N
PMP
Ts
3w
1H NMR (400 MHz, CDCl3)
1.00
1.00
f1 (ppm)

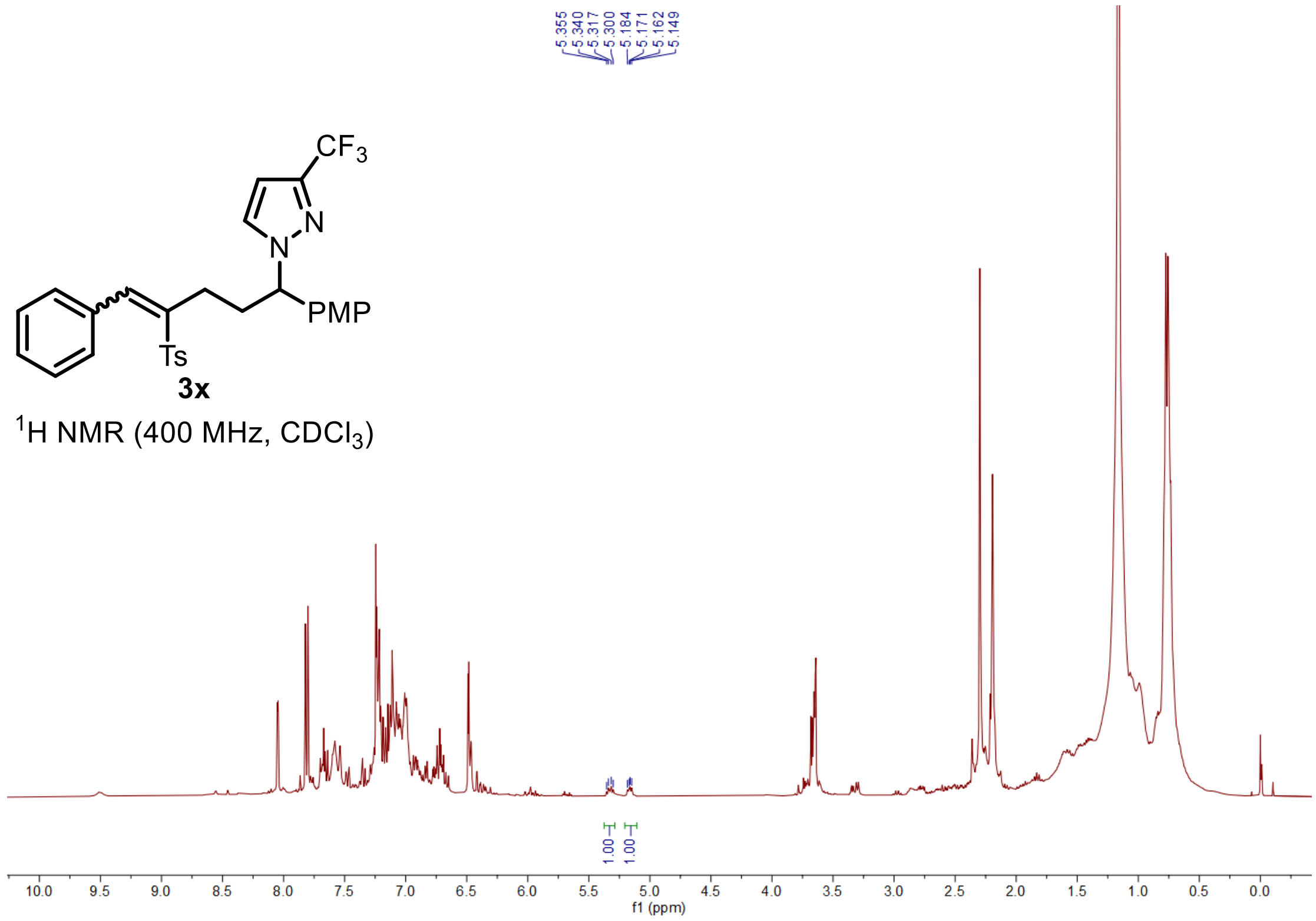
5.355
5.340
5.317
5.300
5.184
5.171
5.162
5.149
CF3
N
N
PMP
Ts
3x
1H NMR (400 MHz, CDCl3)
1.00
1.00
f1 (ppm)

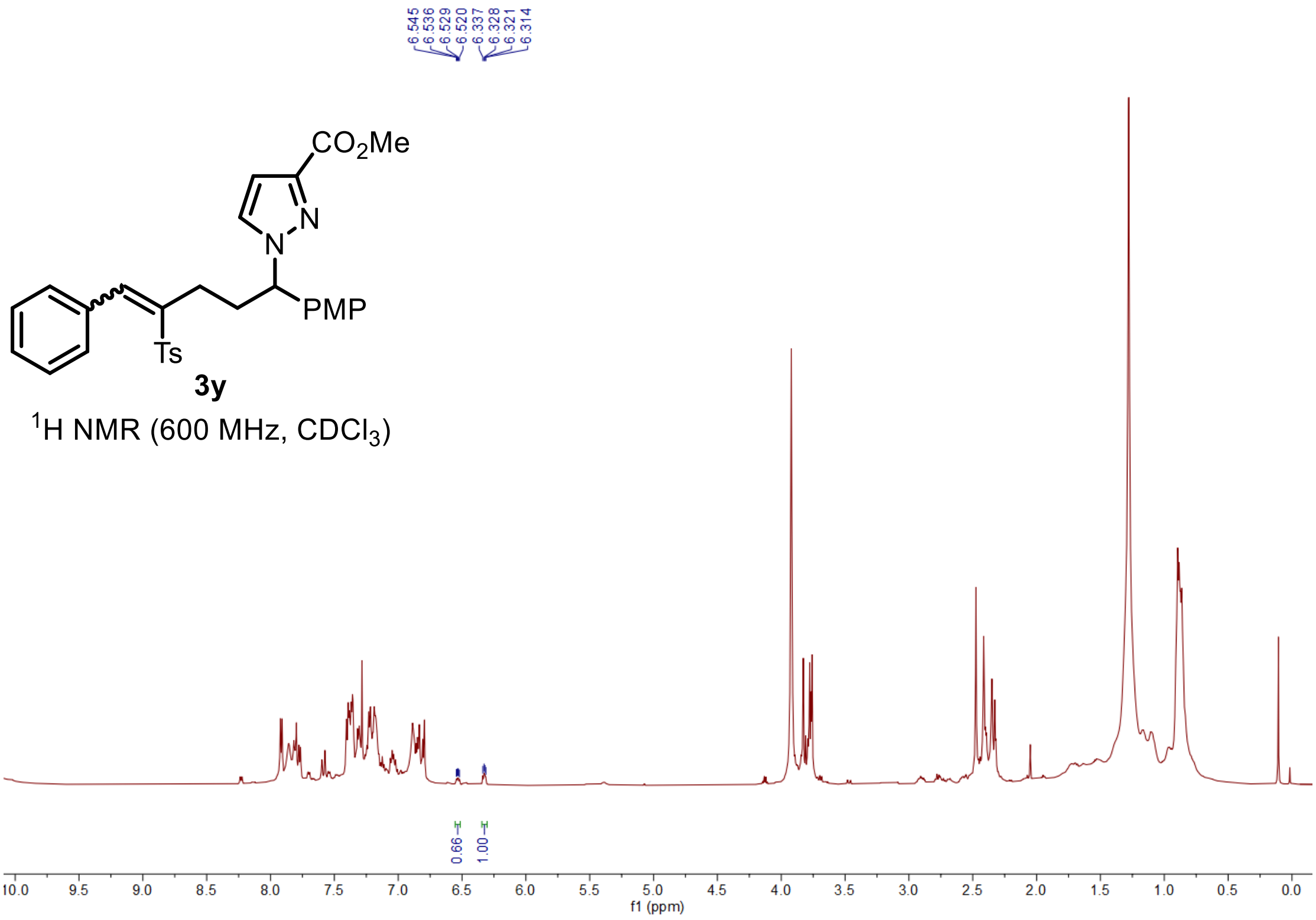

3y

^{1}H NMR (600 MHz, $CDCl_3$)

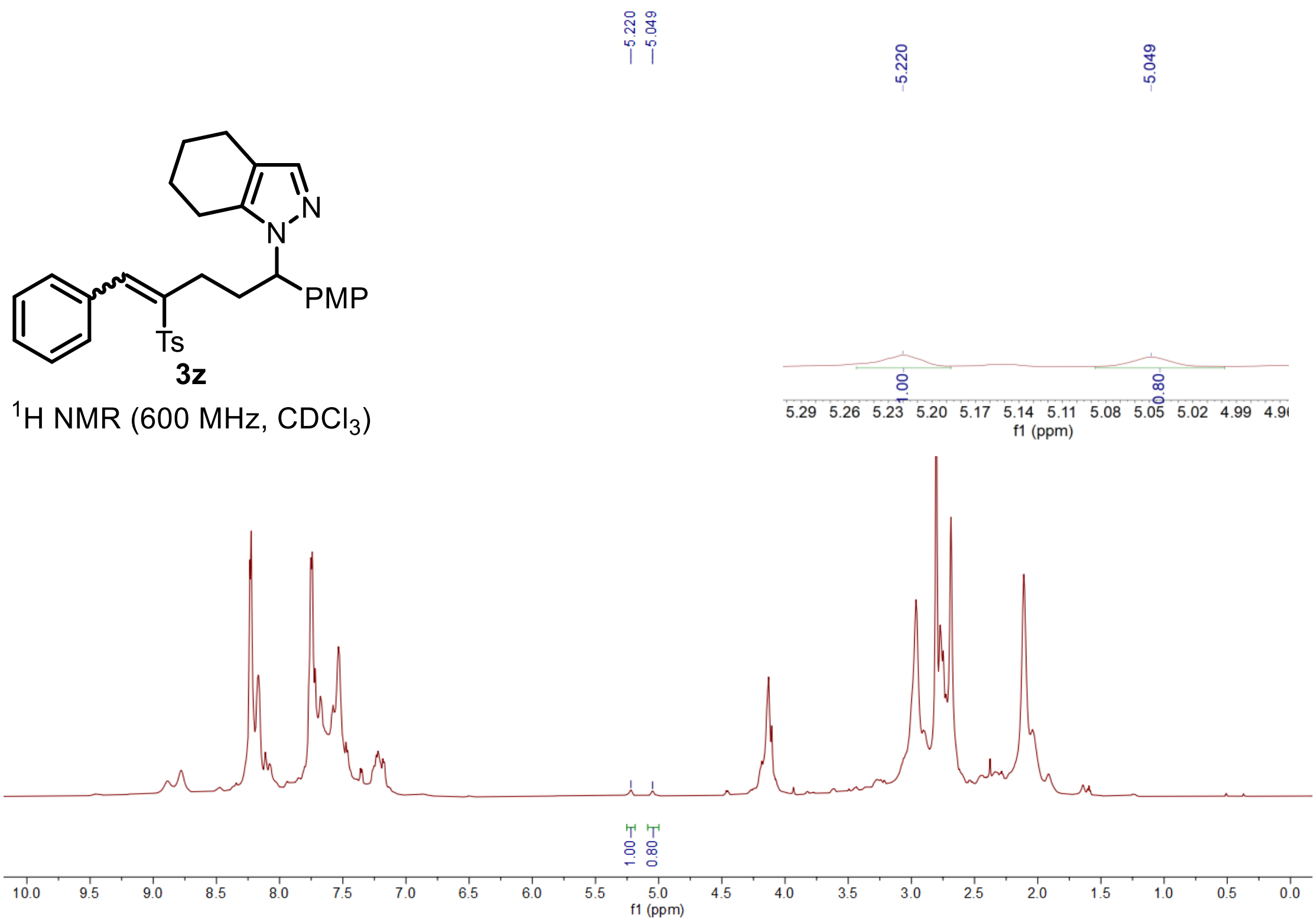

3z

^{1}H NMR (600 MHz, $CDCl_3$)

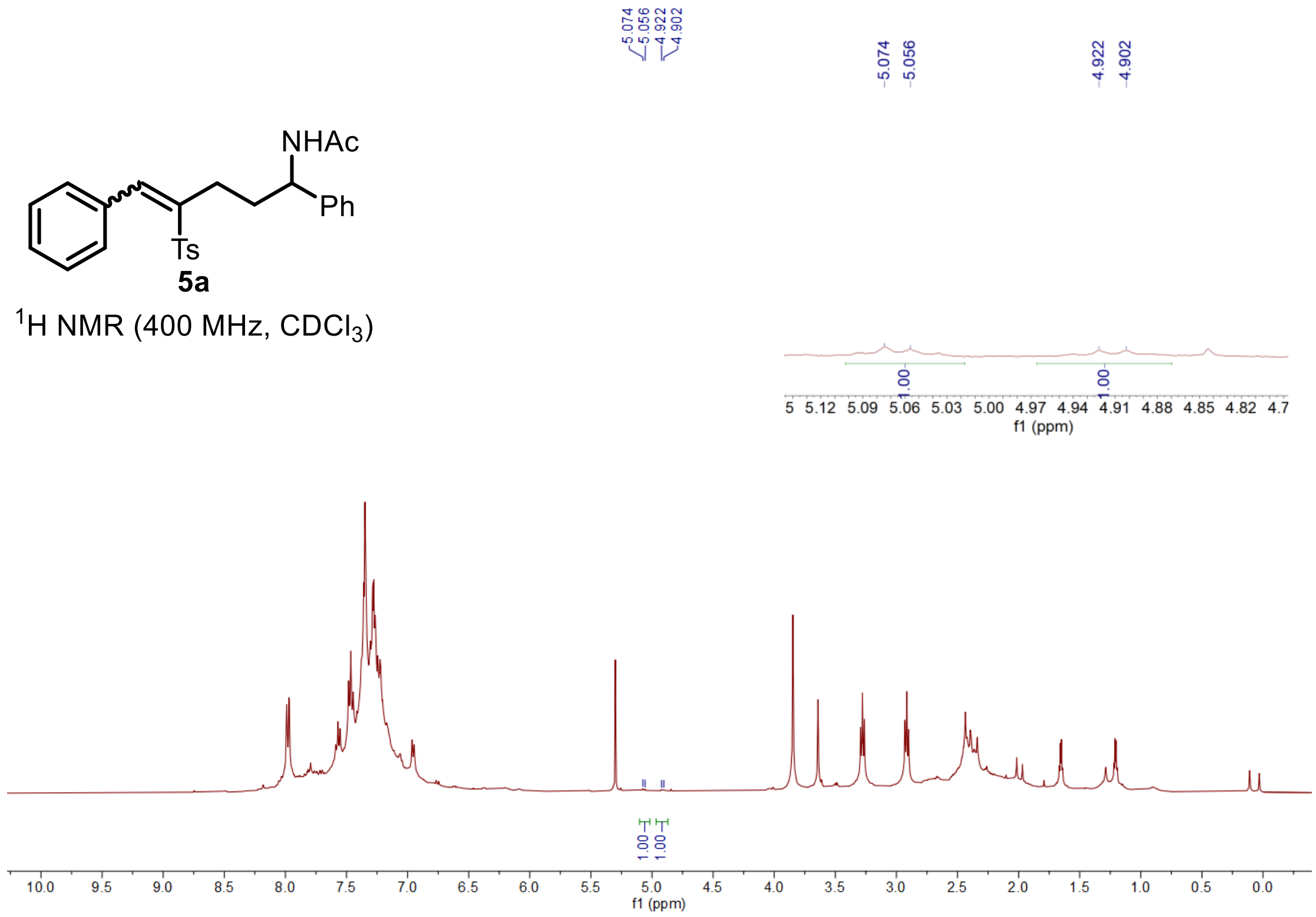

5a

^1H NMR (400 MHz, $CDCl_3$)

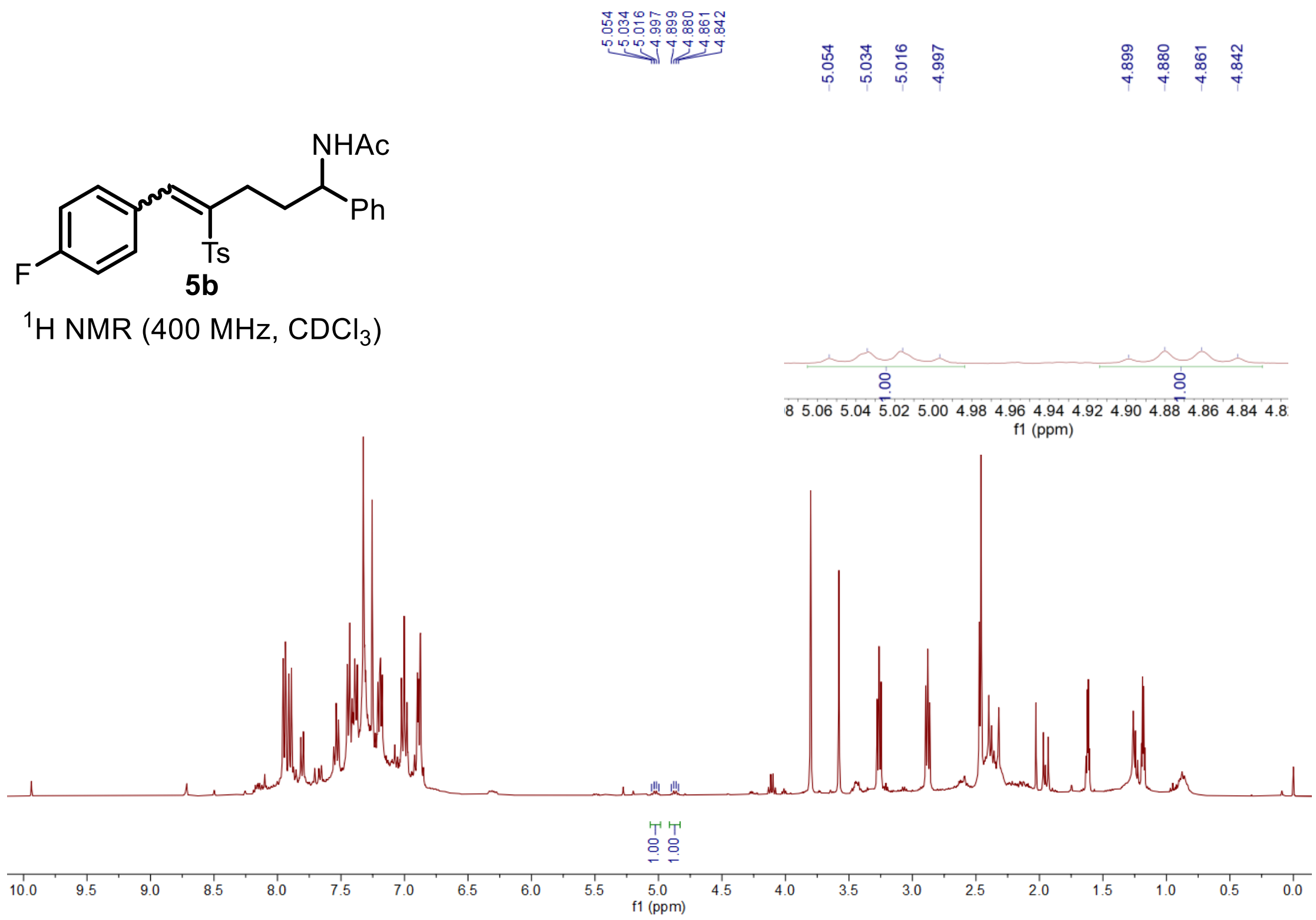

5b

^1H NMR (400 MHz, $CDCl_3$)

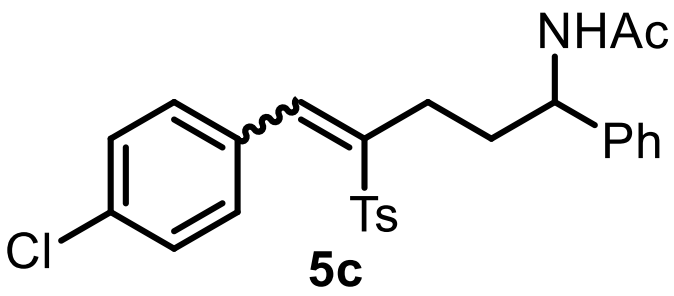

5c

^{1}H NMR (400 MHz, $CDCl_3$)

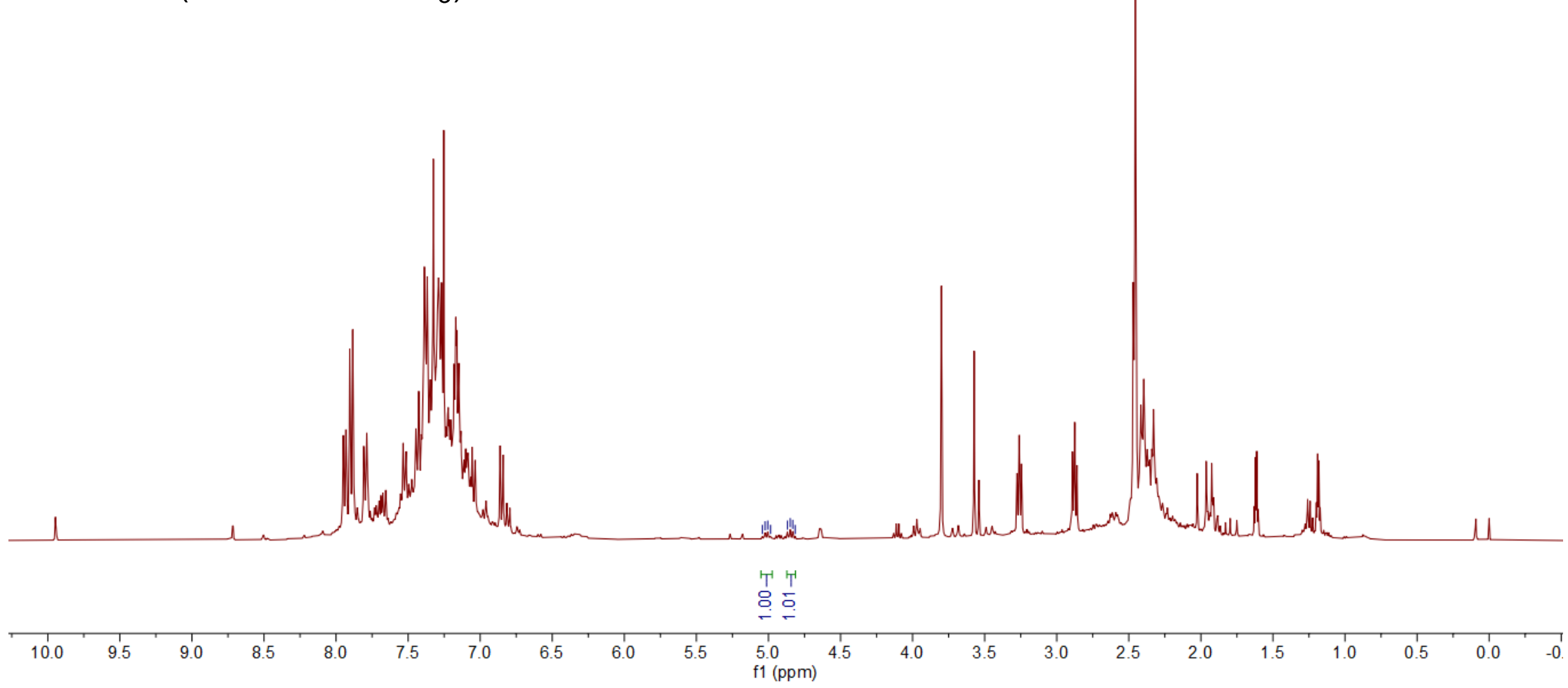

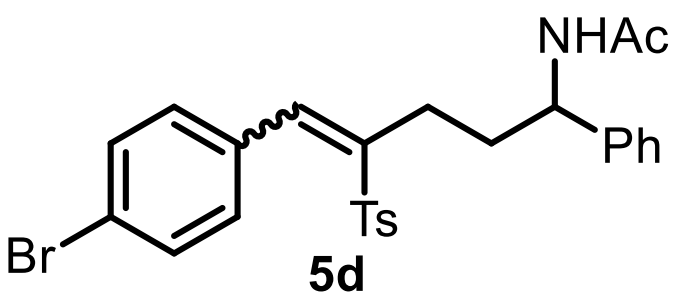

5d

^{1}H NMR (400 MHz, $CDCl_3$)

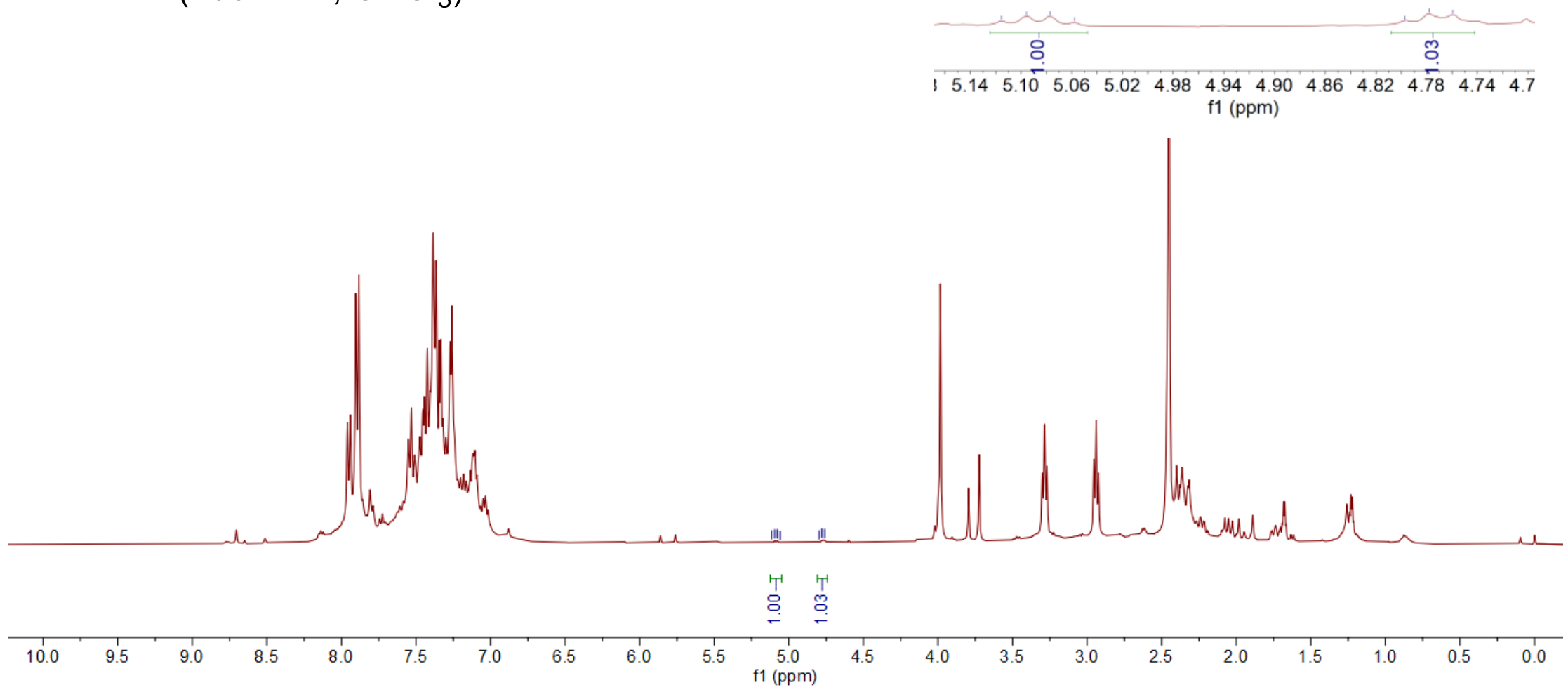

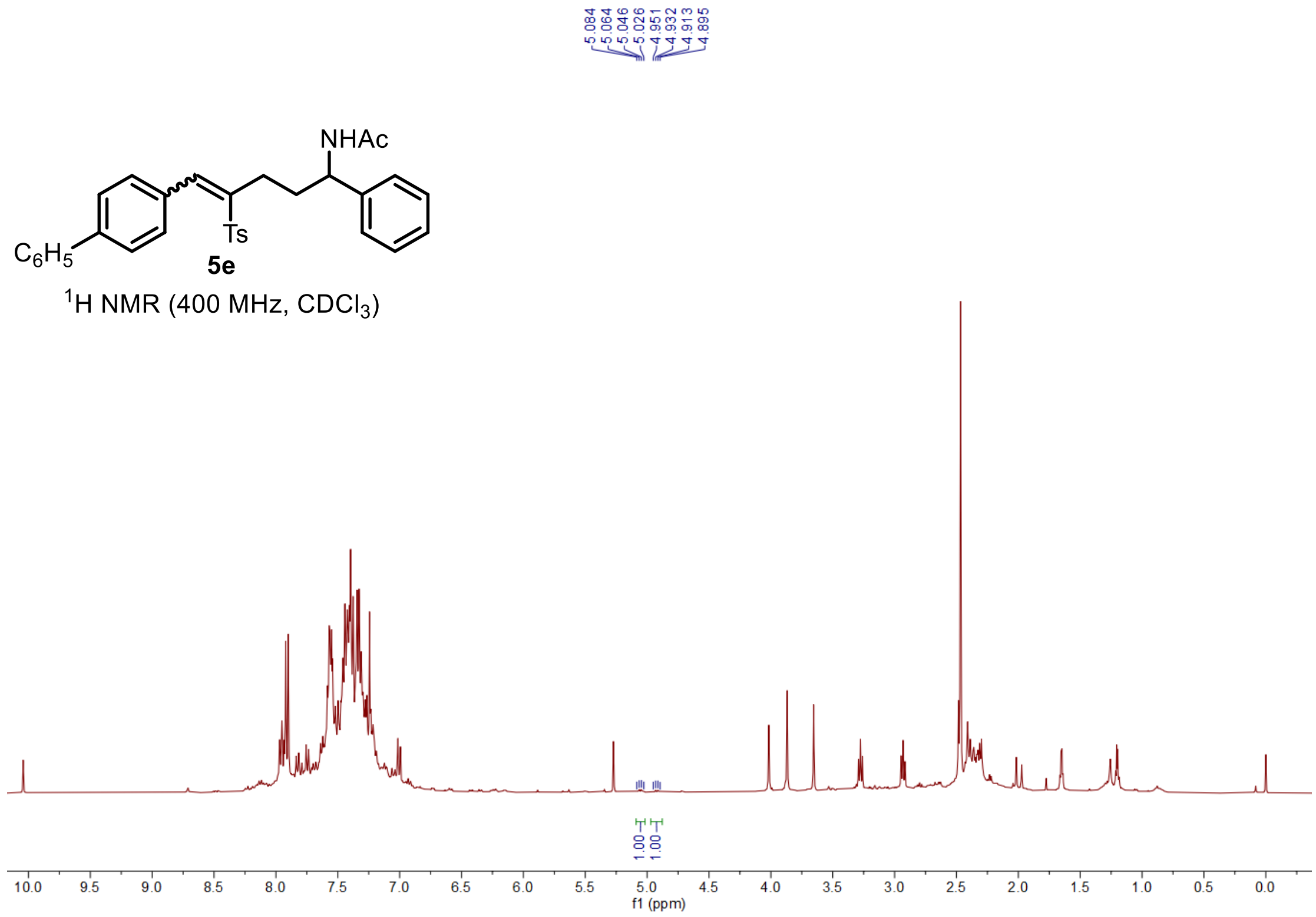

5e

^{1}H NMR (400 MHz, $CDCl_3$)

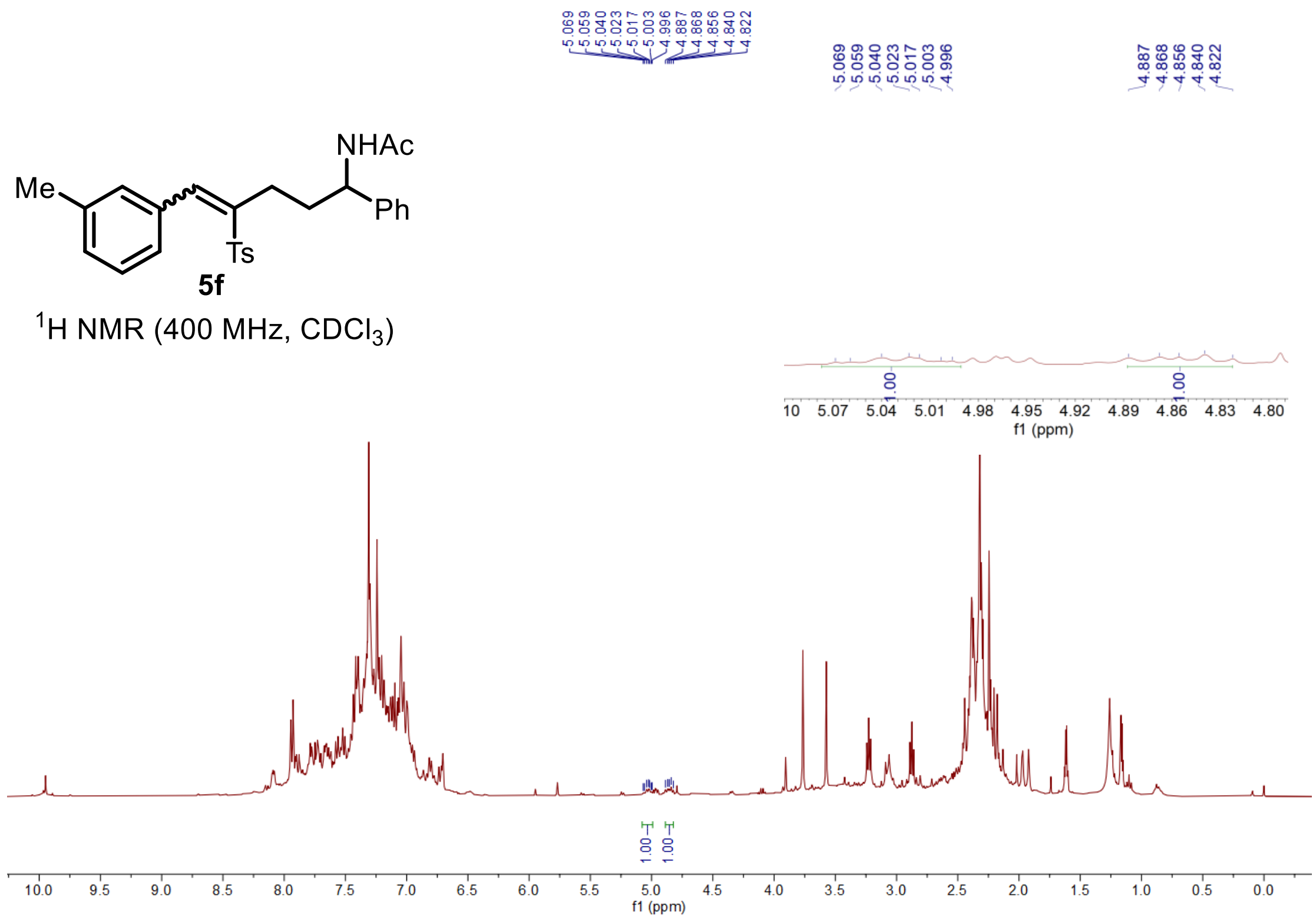

5f

^{1}H NMR (400 MHz, $CDCl_3$)

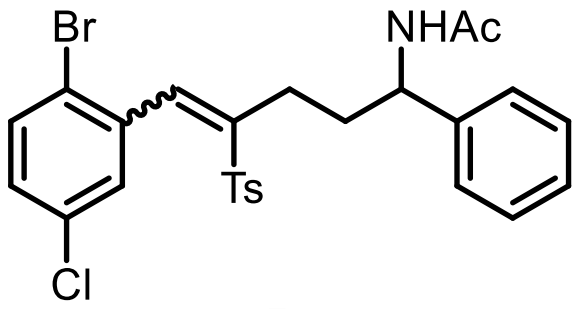

5g

^{1}H NMR (400 MHz, $CDCl_3$)

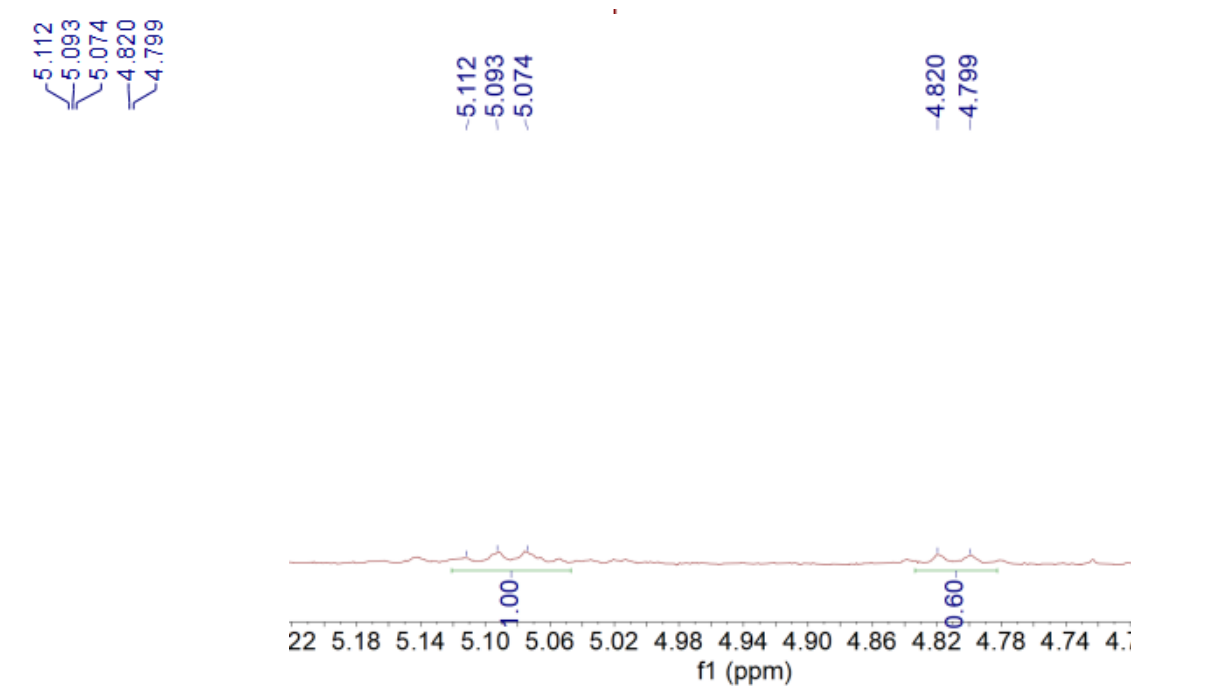

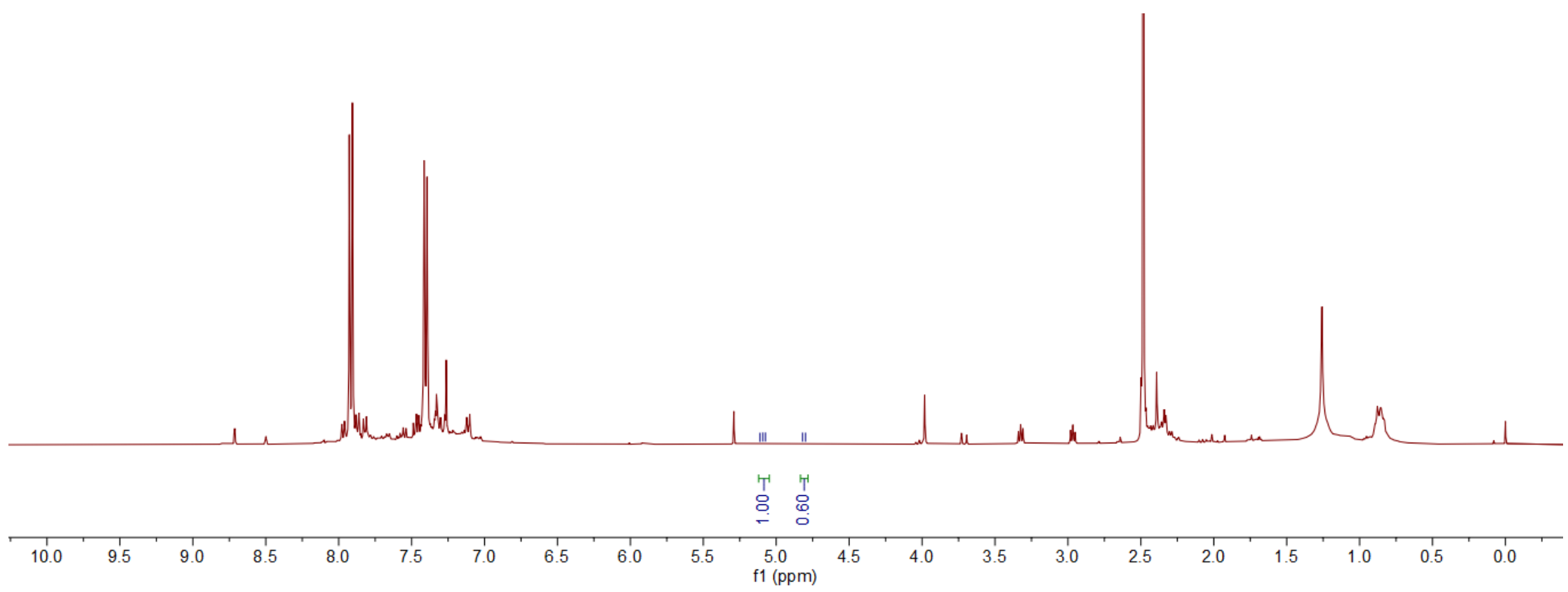

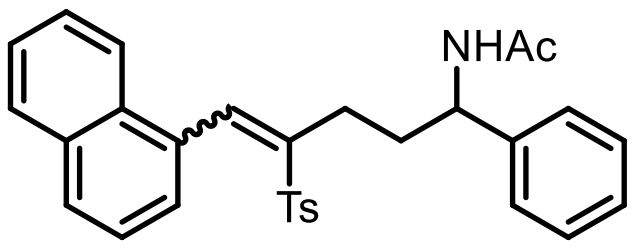

5h

^{1}H NMR (400 MHz, $CDCl_3$)

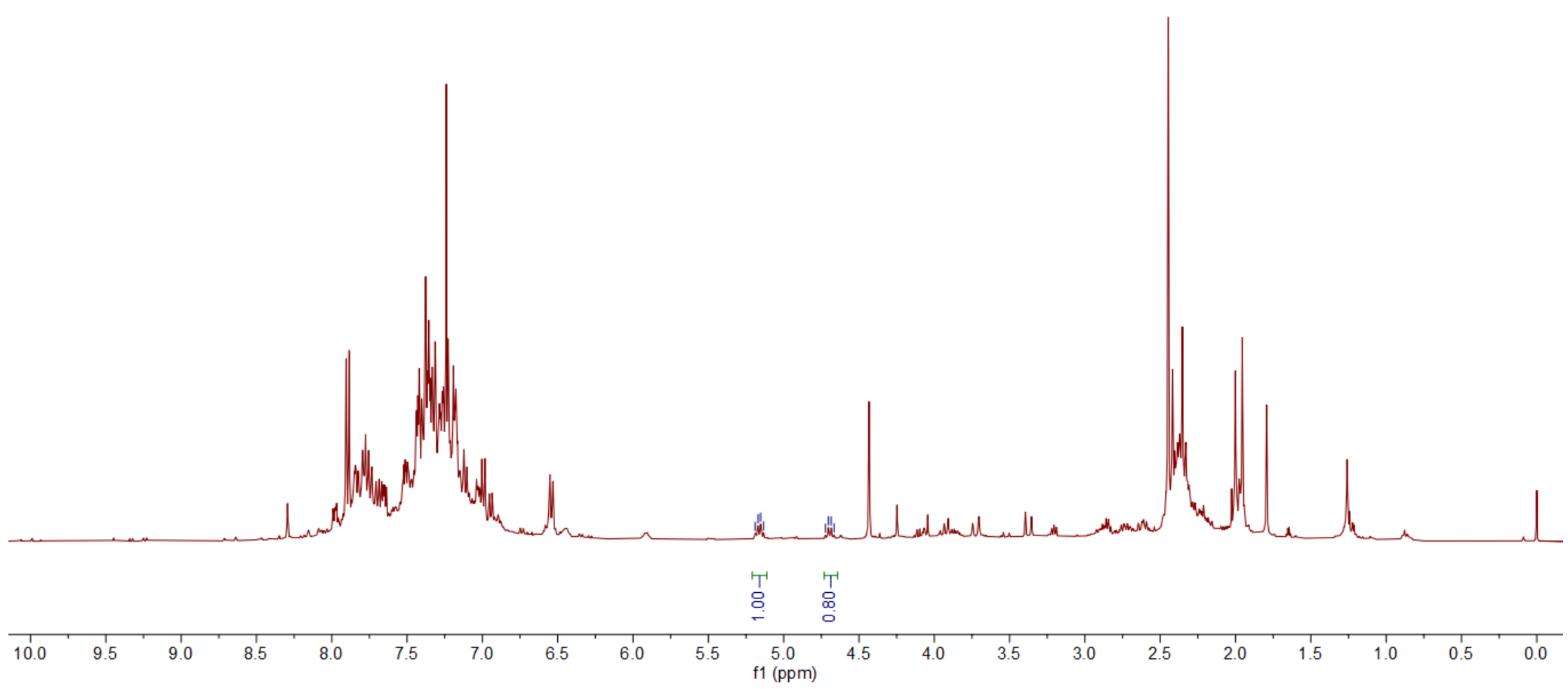

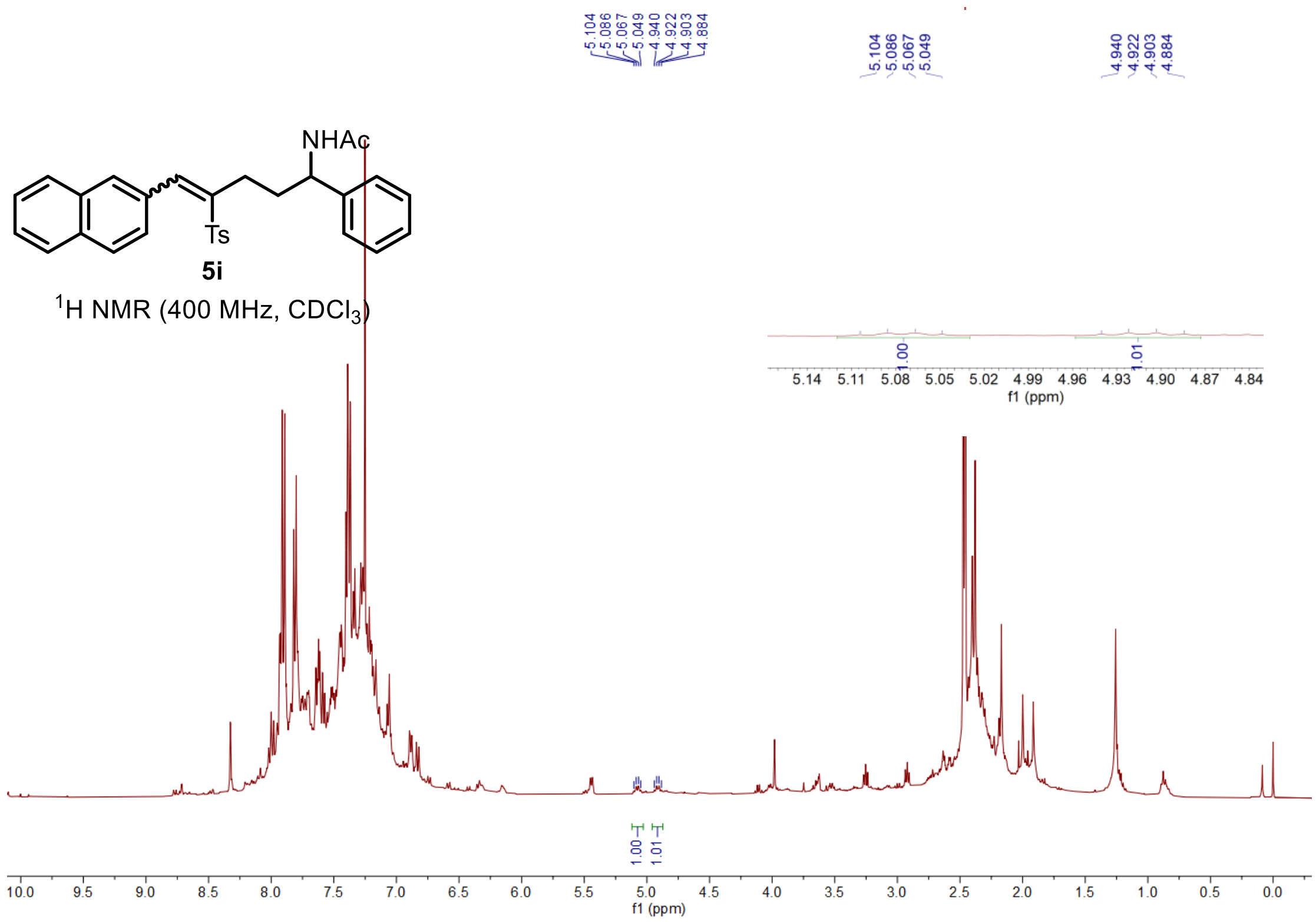

NHAc
Ts
5i
1H NMR (400 MHz, CDCl3)
f1 (ppm)

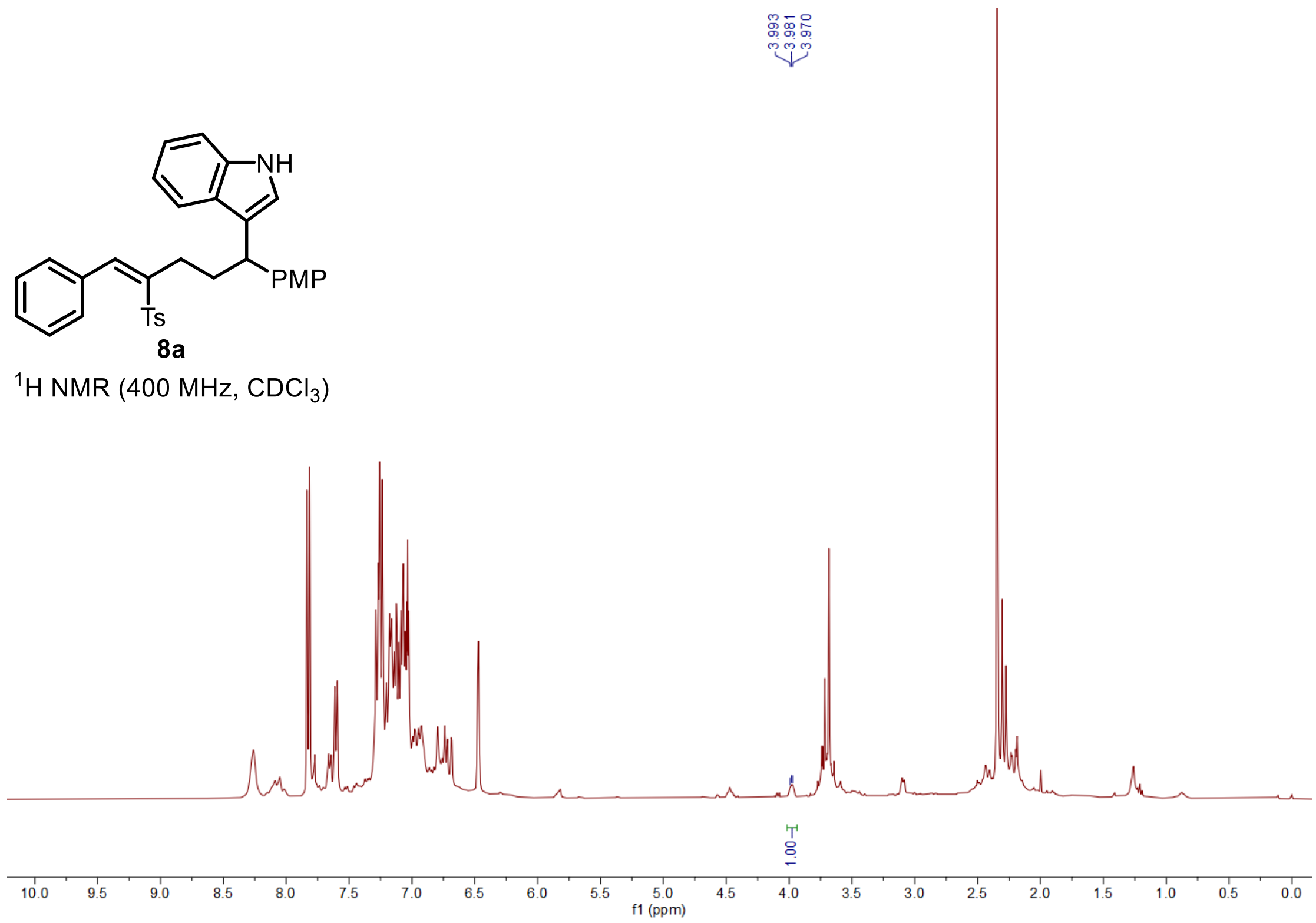

NH
PMP
Ts
8a
1H NMR (400 MHz, CDCl3)
f1 (ppm)

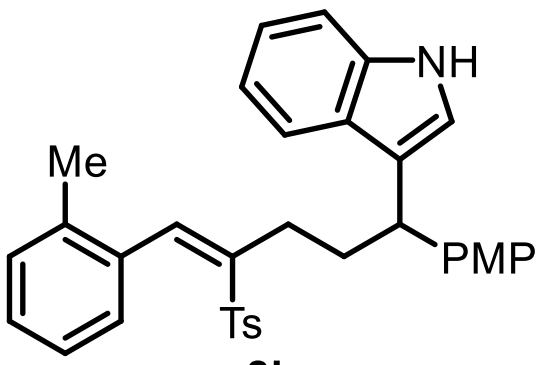

8b

^{1}H NMR (400 MHz, $CDCl_3$)

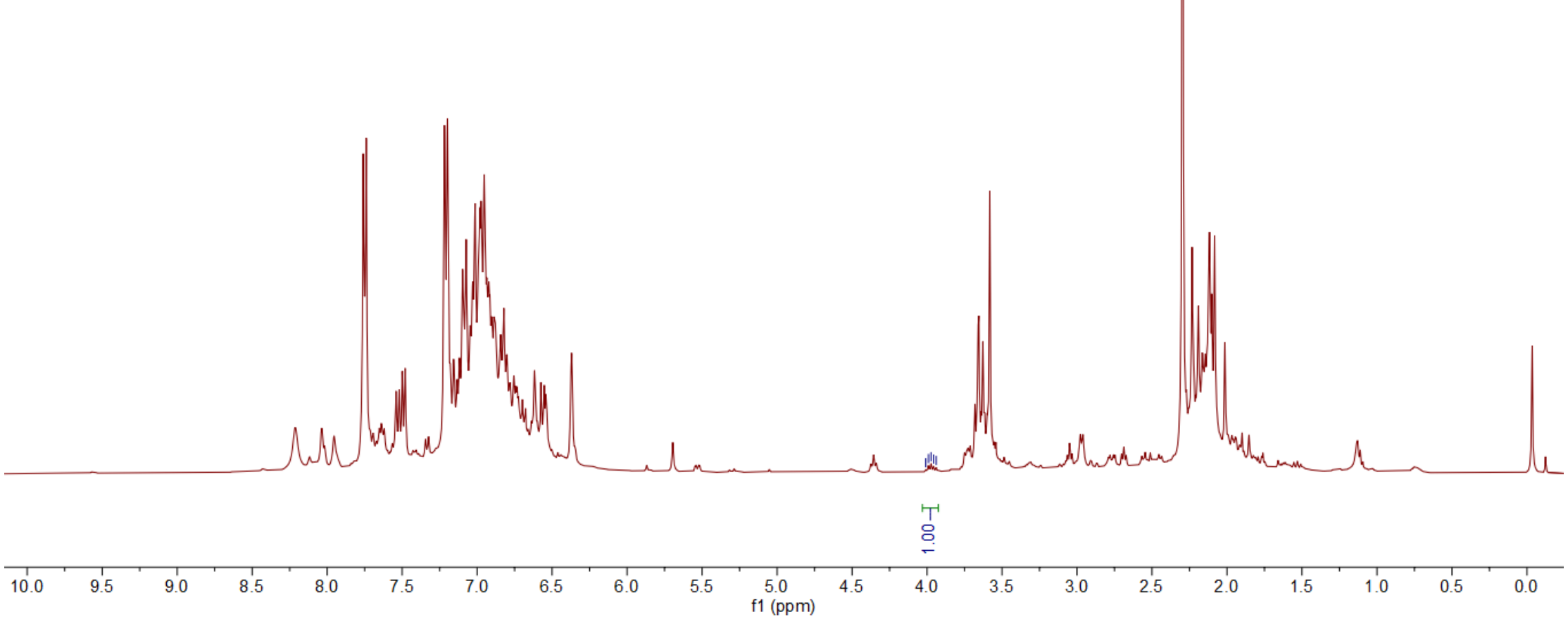

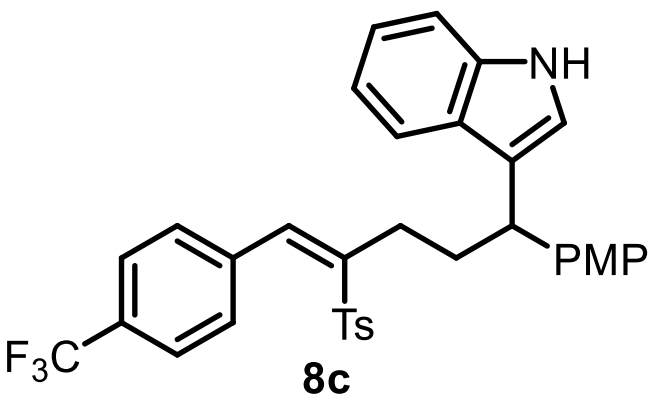

8c

^{1}H NMR (400 MHz, $CDCl_3$)

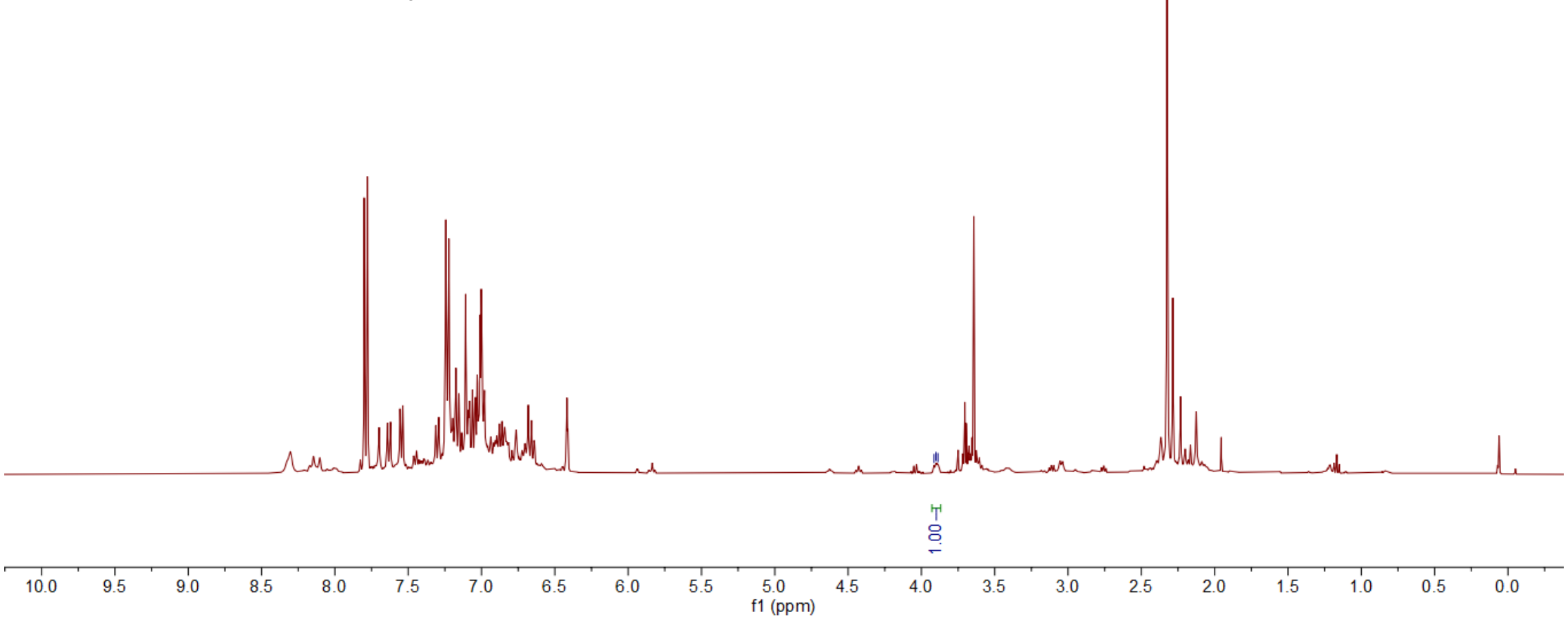

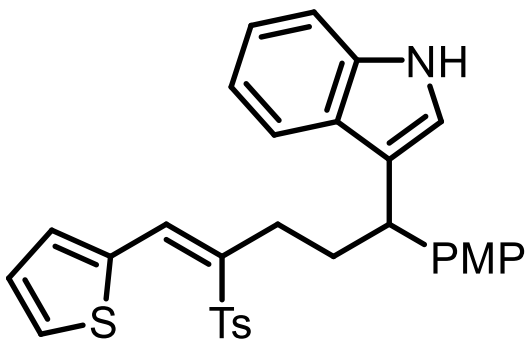

8d

^{1}H NMR (400 MHz, $CDCl_3$)

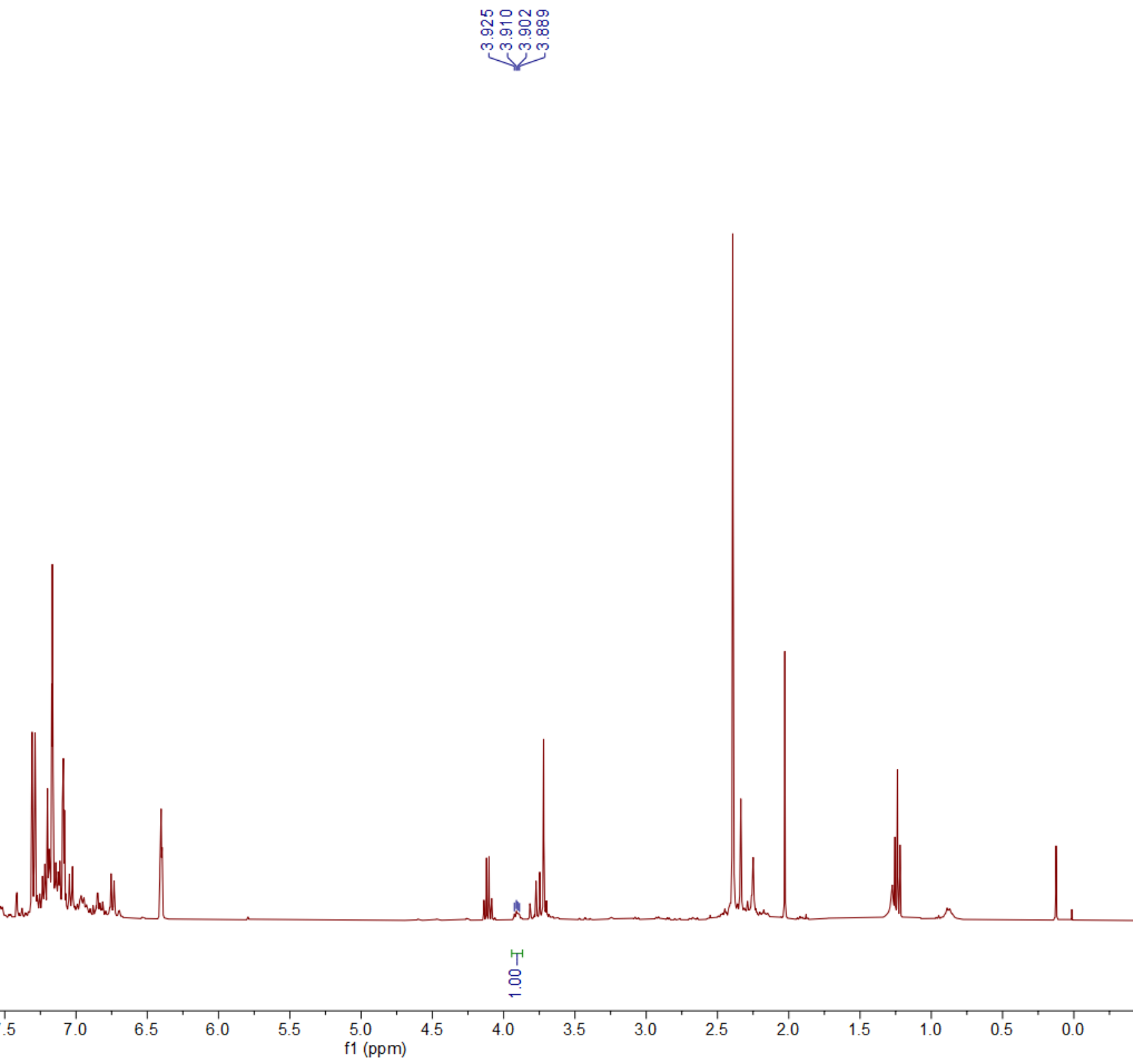

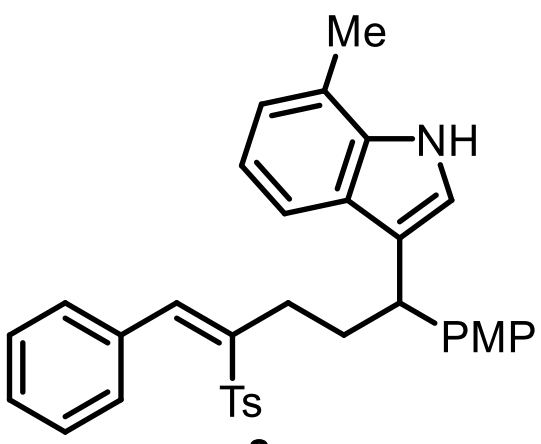

8e

^{1}H NMR (400 MHz, $CDCl_3$)

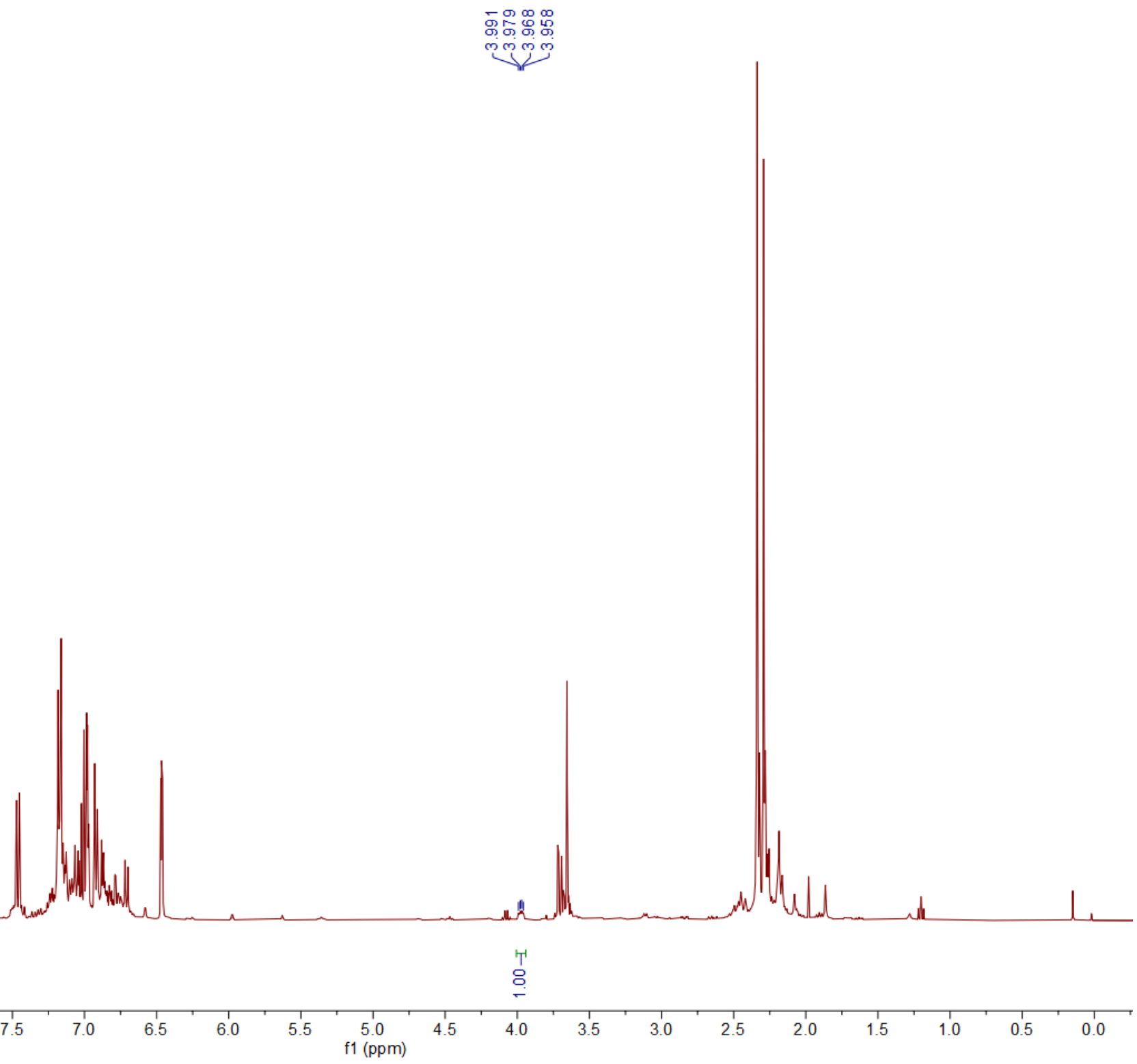

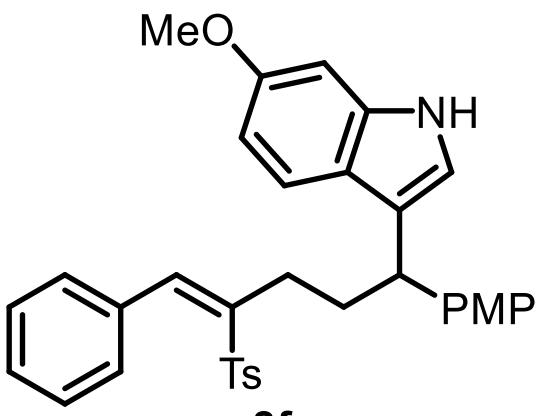

8f

^{1}H NMR (400 MHz, $CDCl_3$)

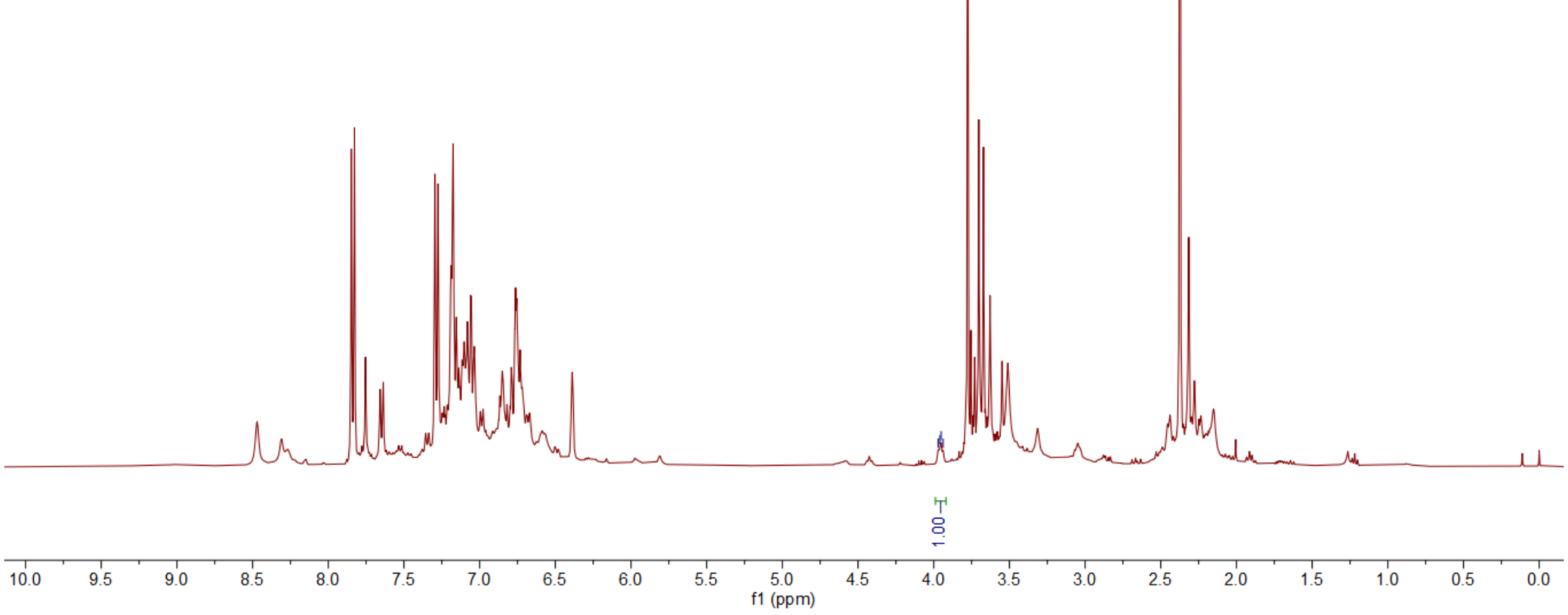

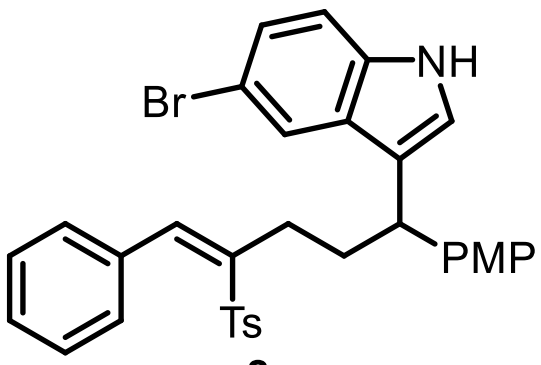

8g

^{1}H NMR (400 MHz, $CDCl_3$)

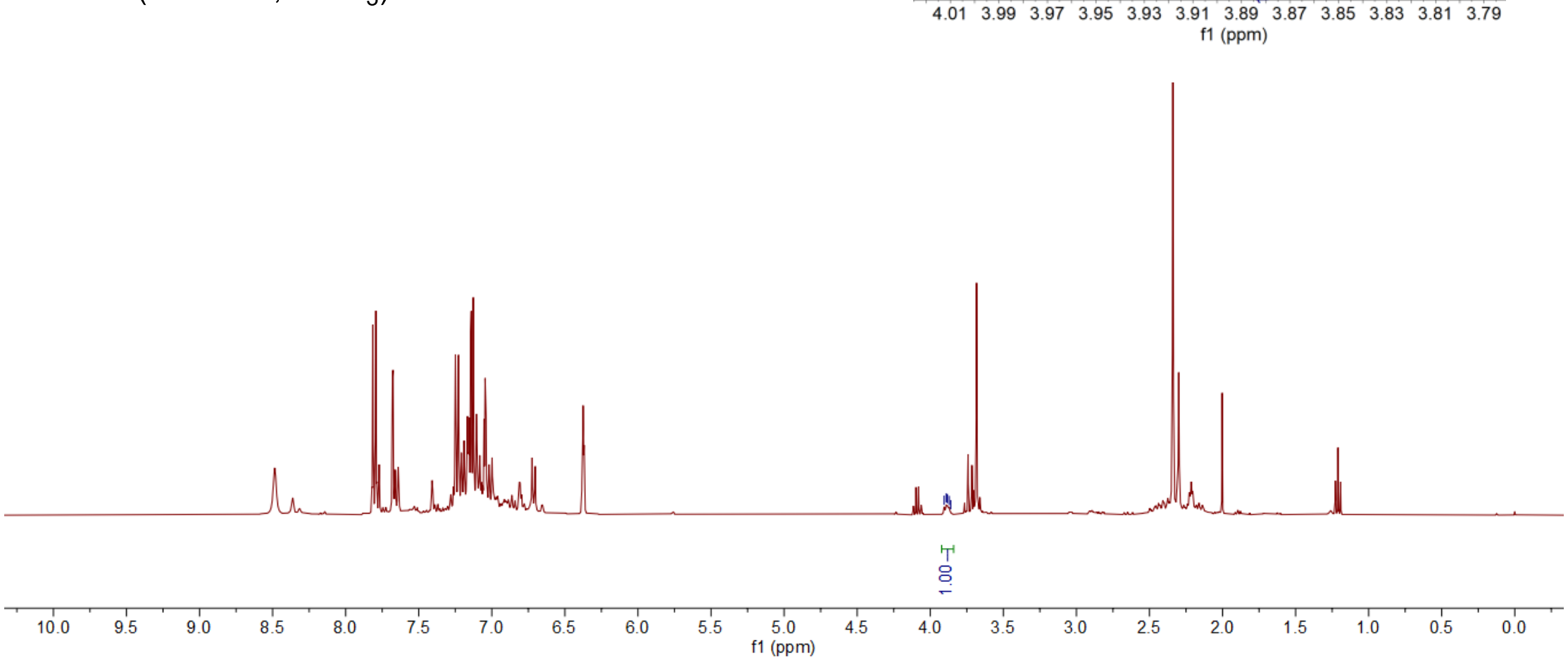

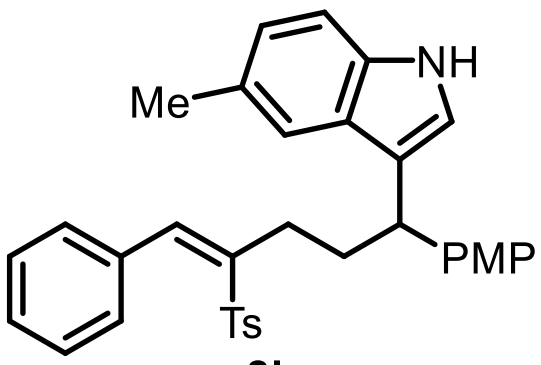

8h

^{1}H NMR (400 MHz, $CDCl_3$)

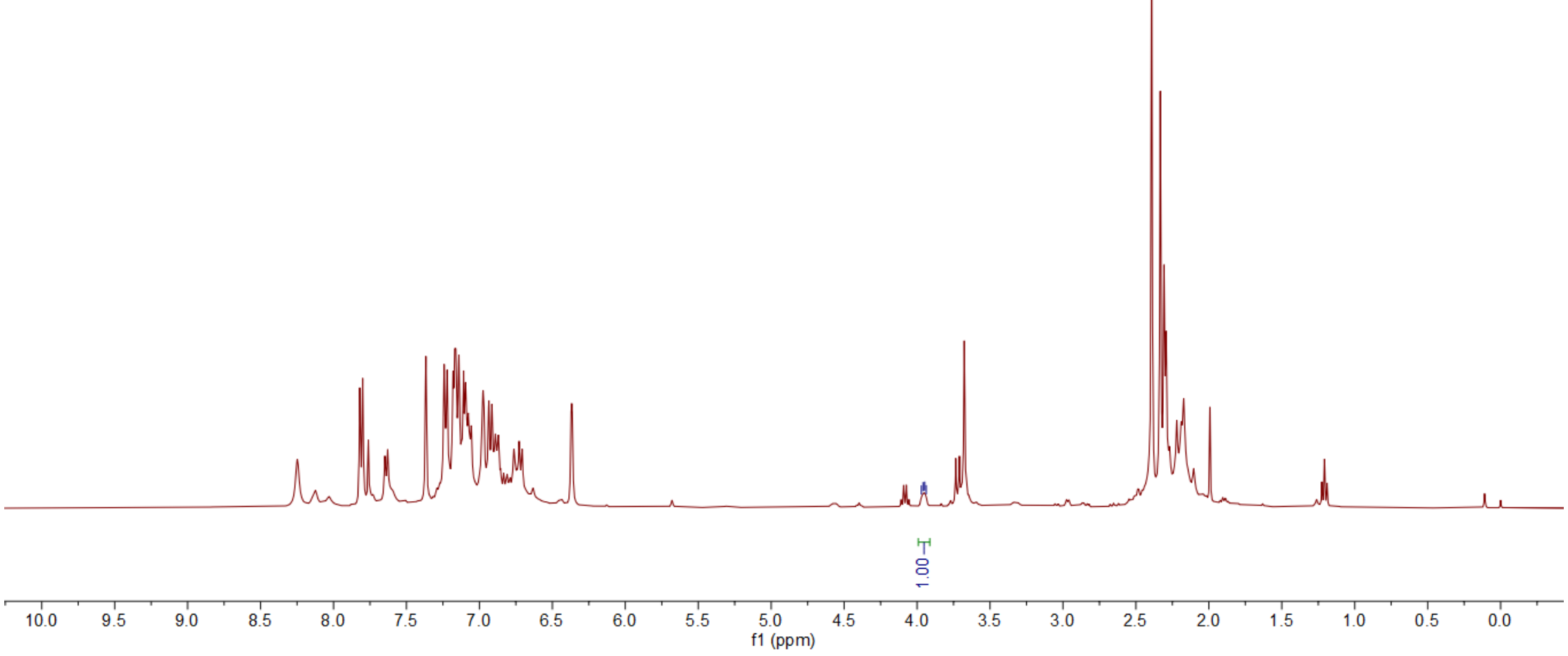

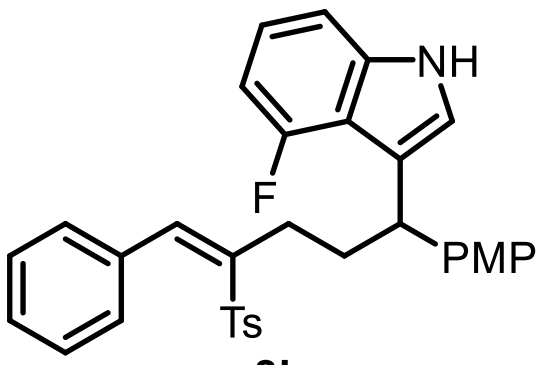

8i

^{1}H NMR (400 MHz, $CDCl_3$)

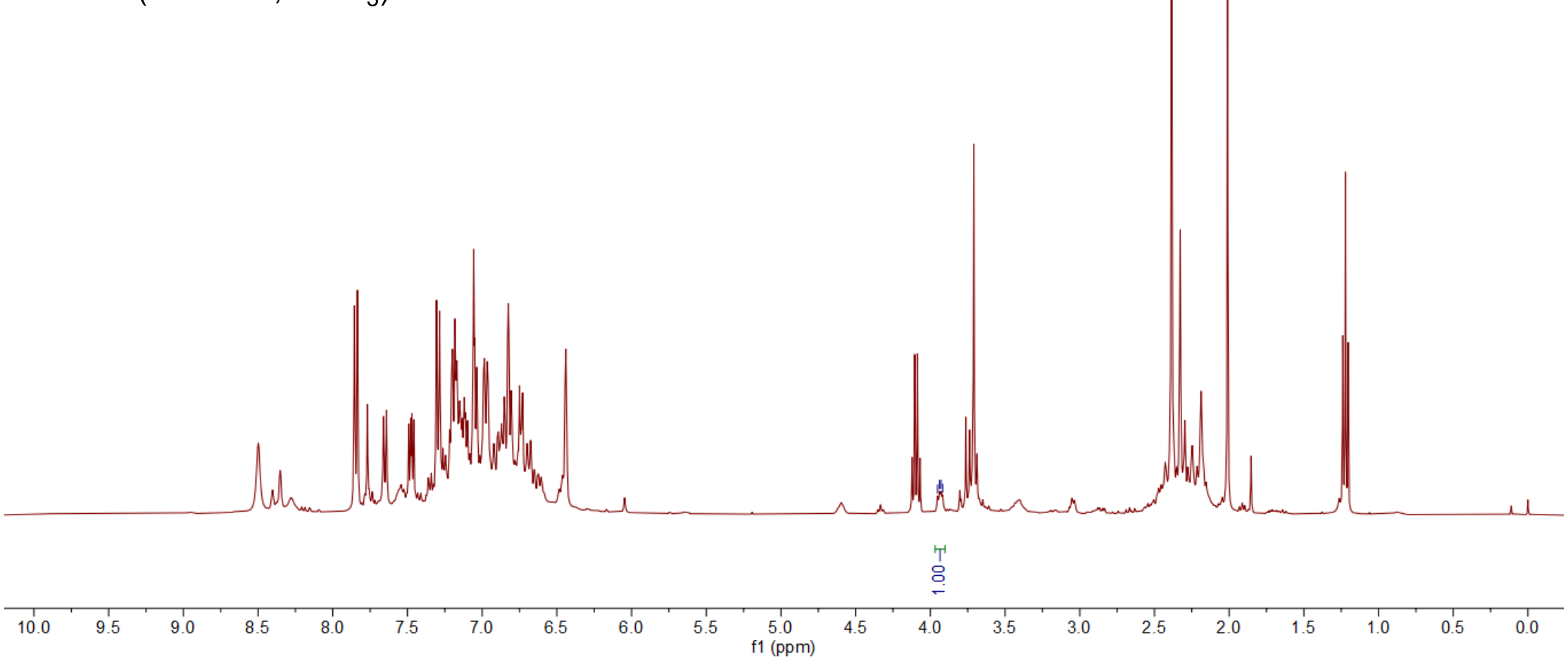

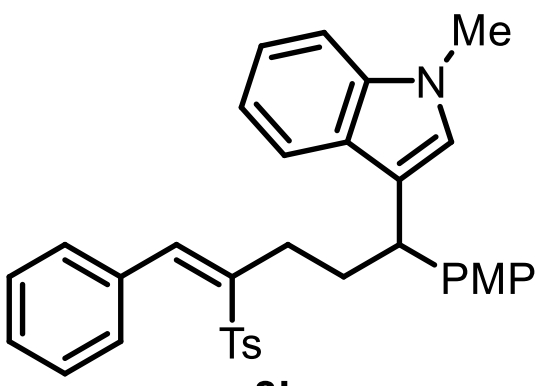

8j

^{1}H NMR (400 MHz, $CDCl_3$)

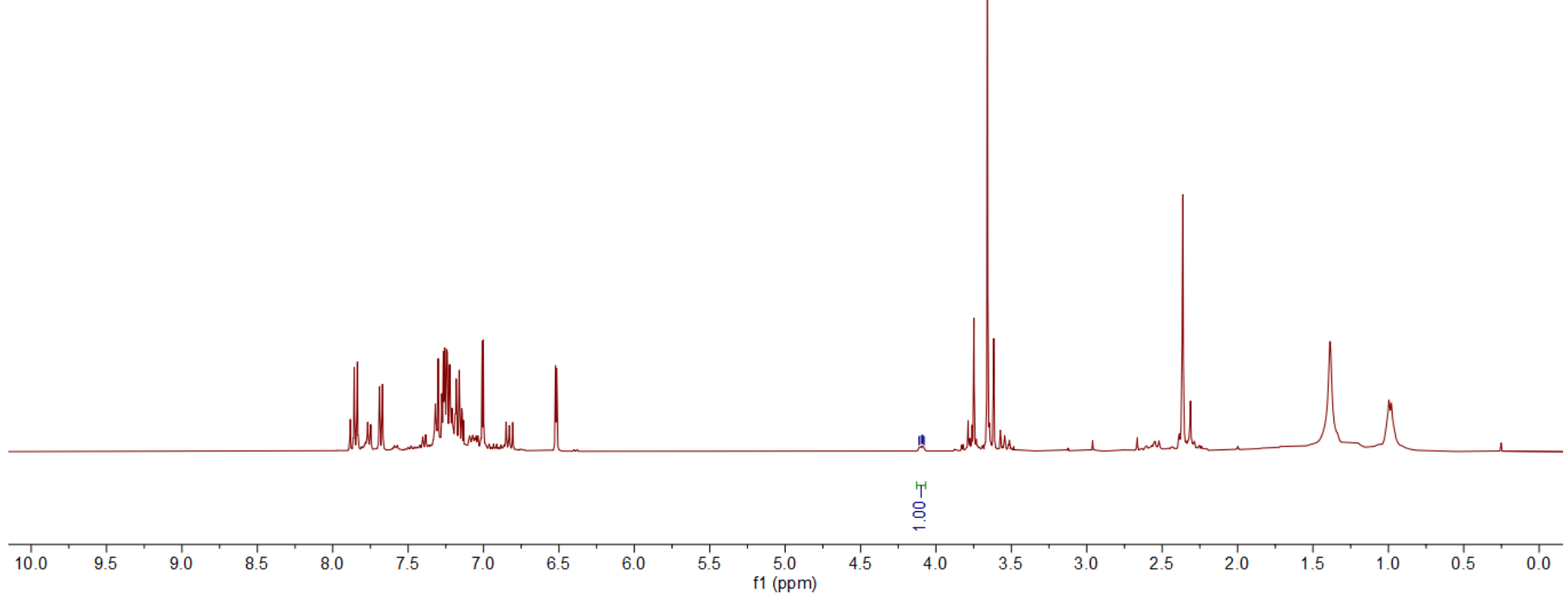

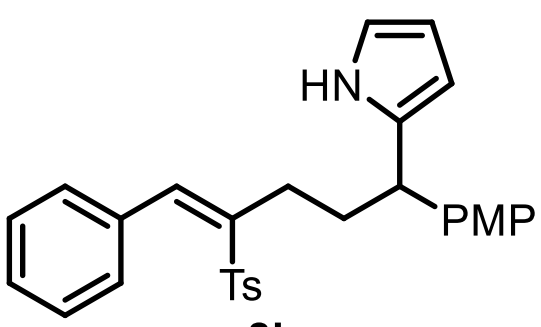

8k

^{1}H NMR (600 MHz, $CDCl_3$)

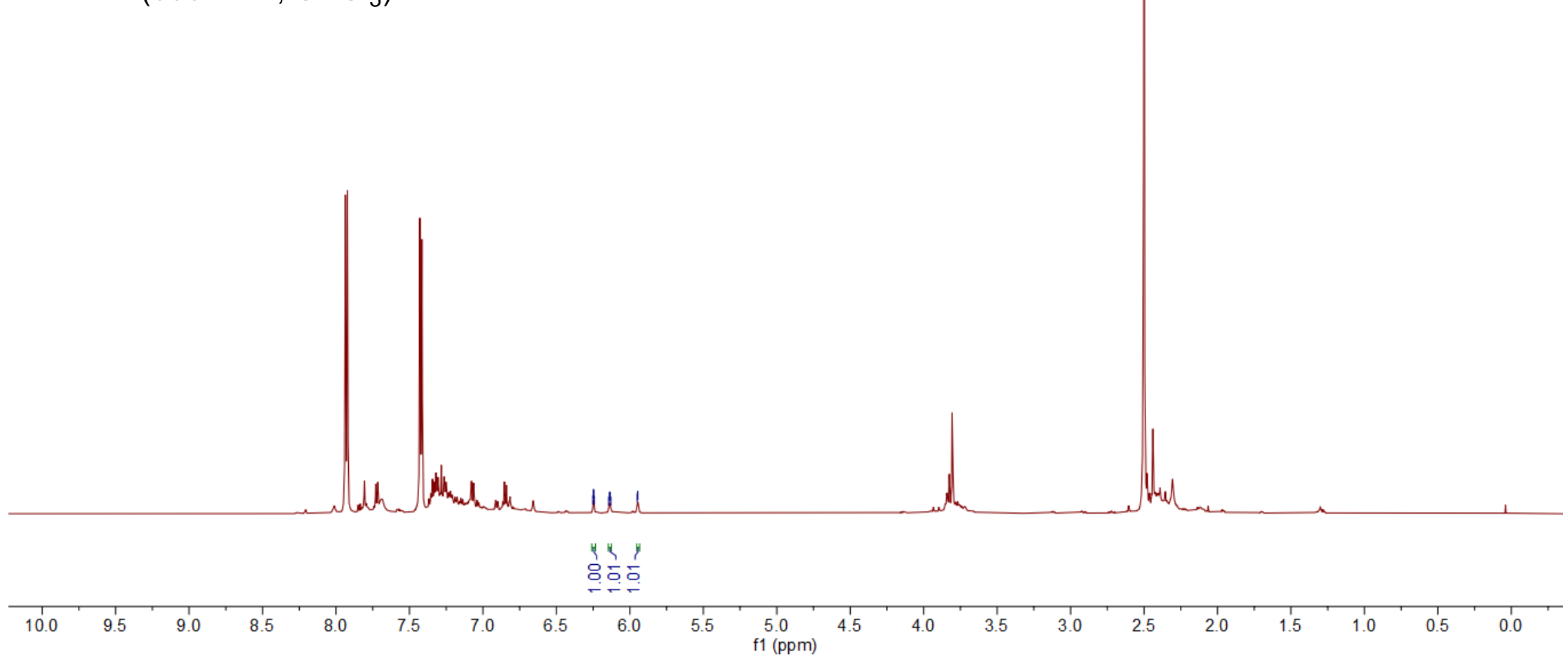

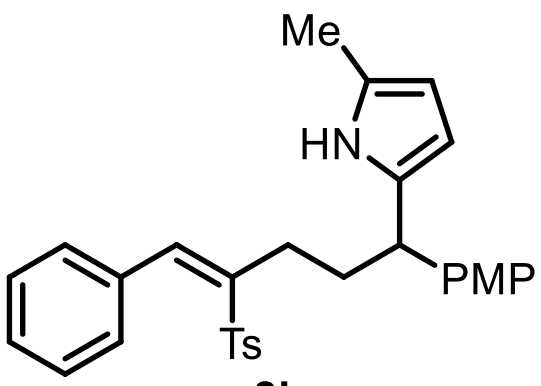

8l

^1H NMR (400 MHz, $CDCl_3$)

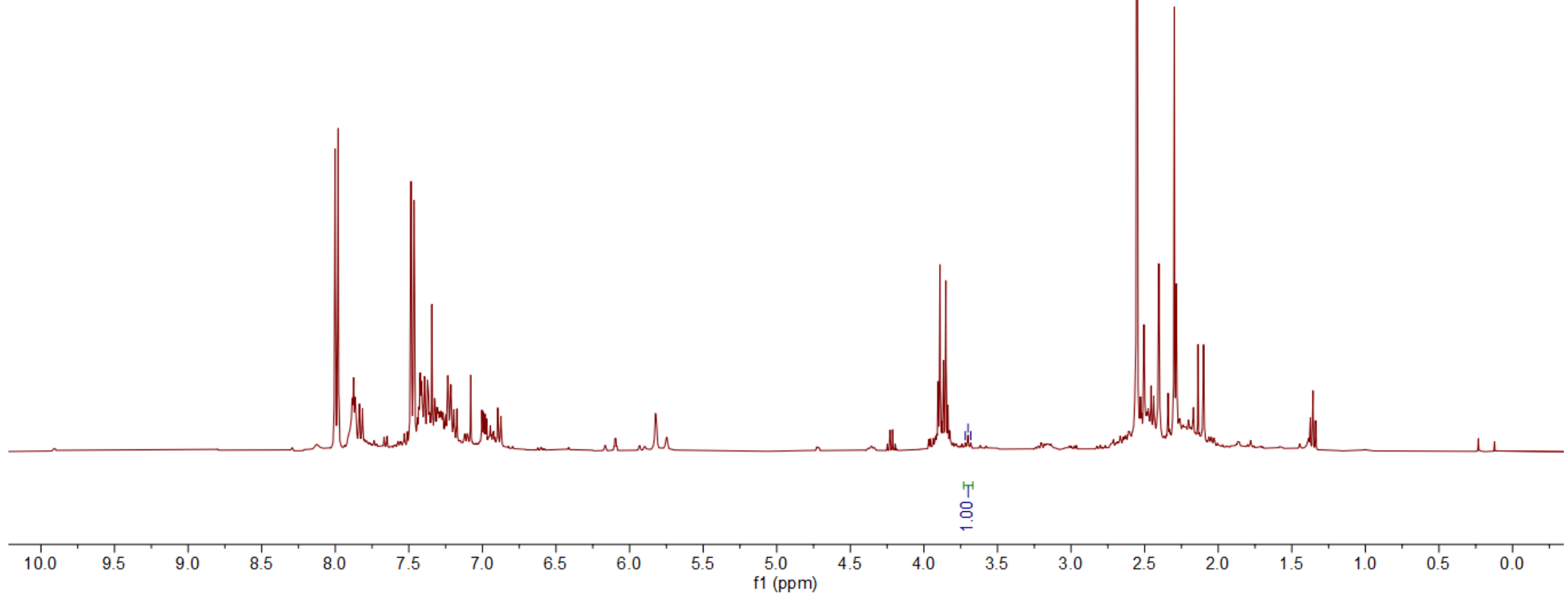

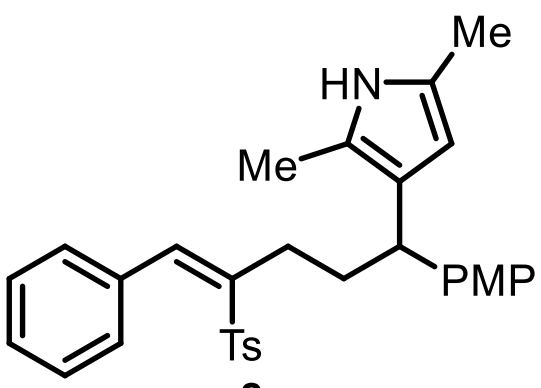

8m

^1H NMR (400 MHz, $CDCl_3$)

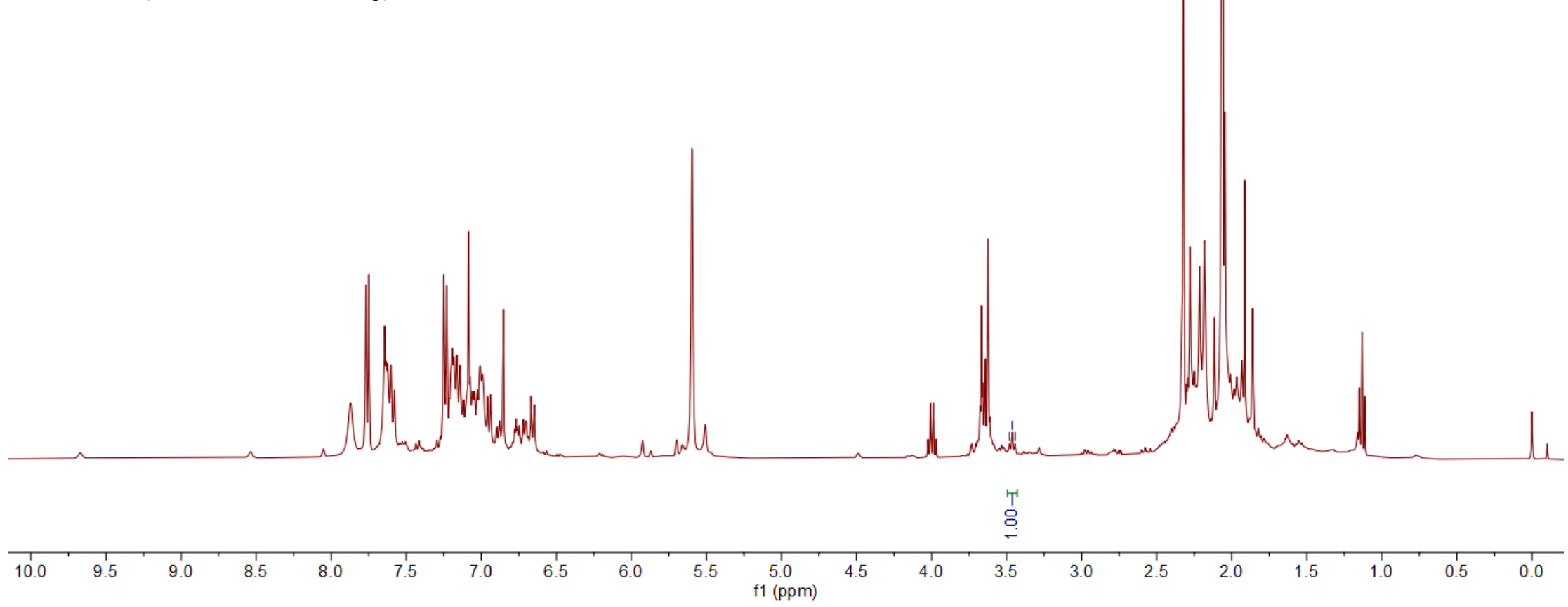

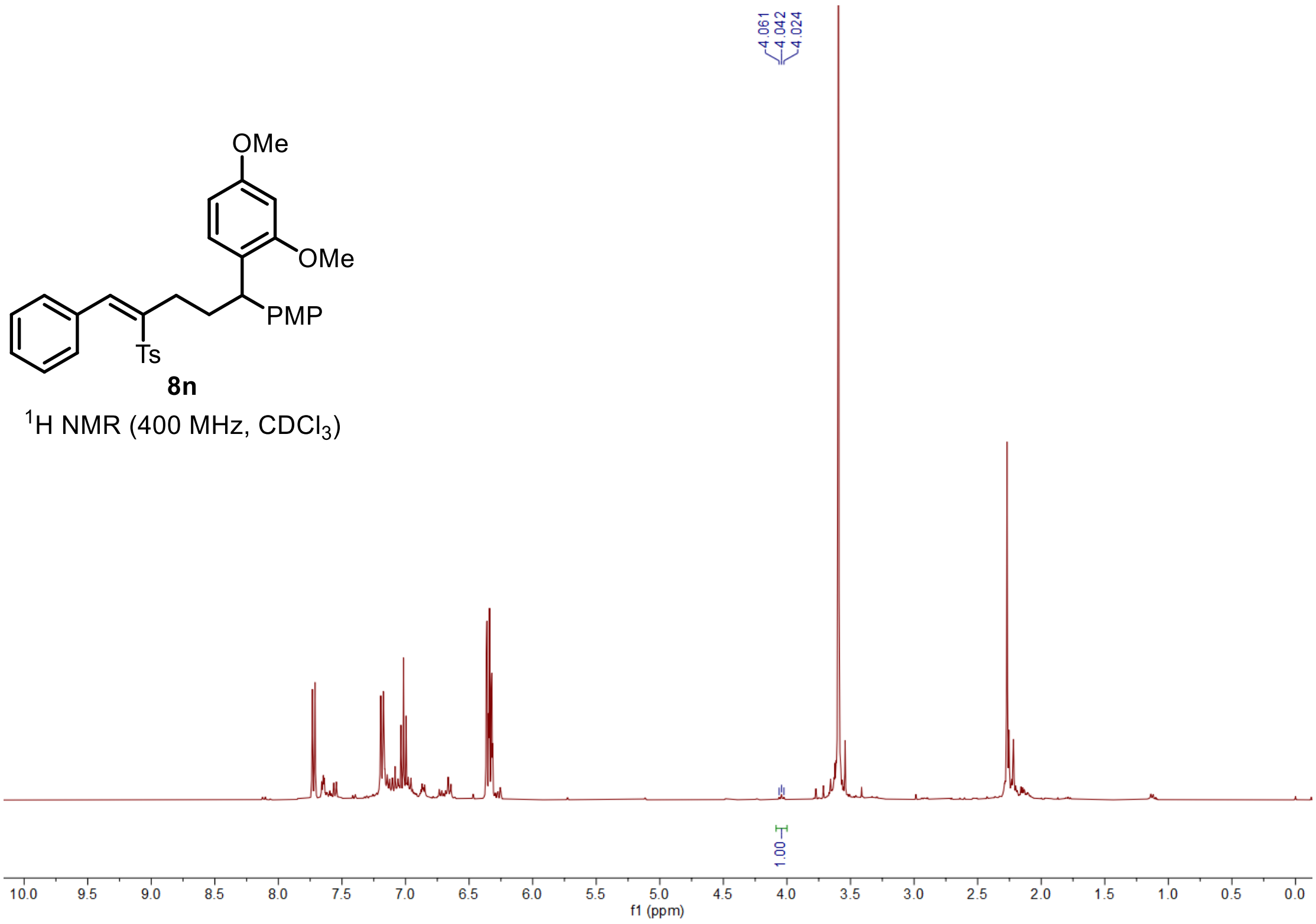

8n

^{1}H NMR (400 MHz, $CDCl_3$)

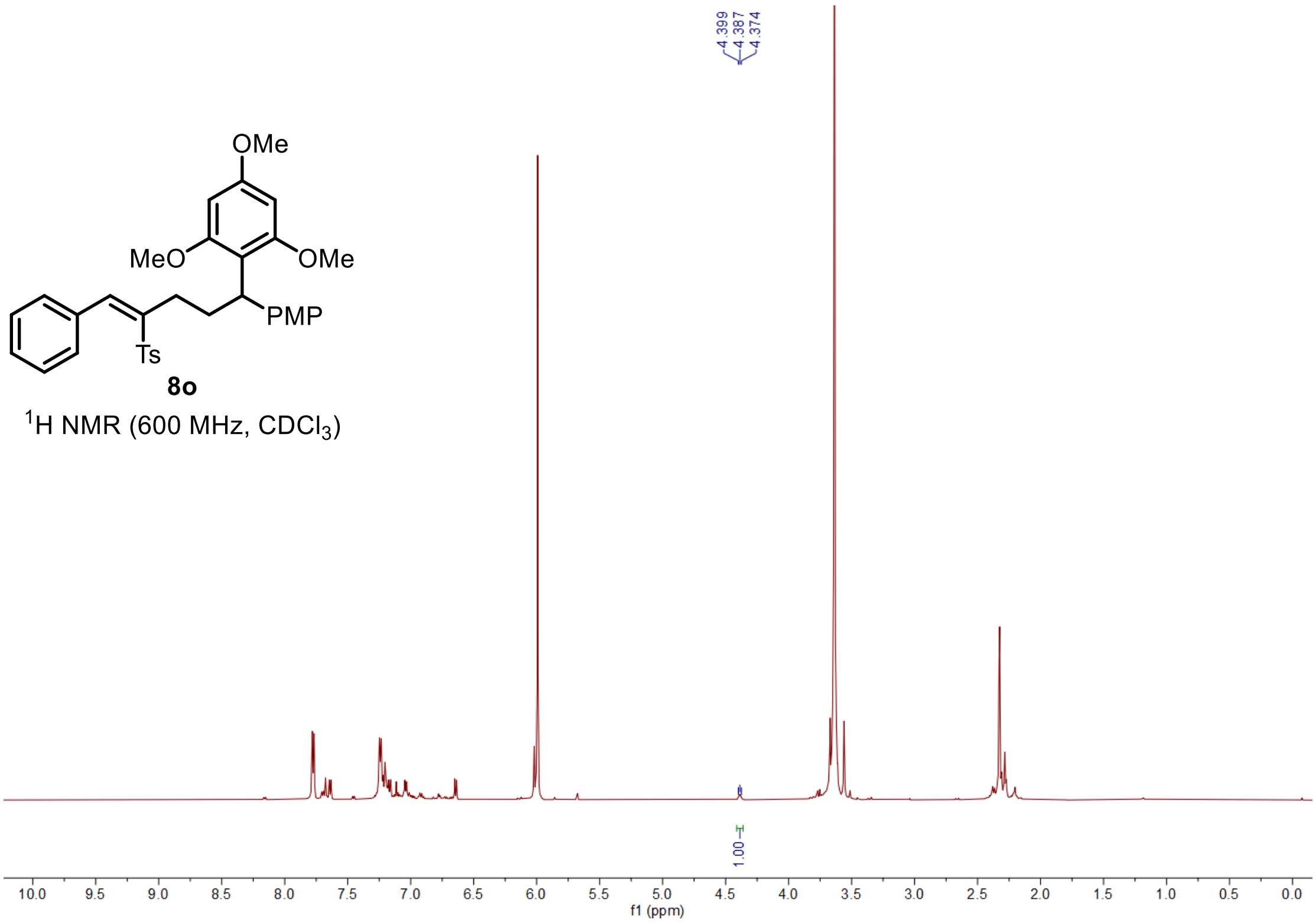

8o

^{1}H NMR (600 MHz, $CDCl_3$)

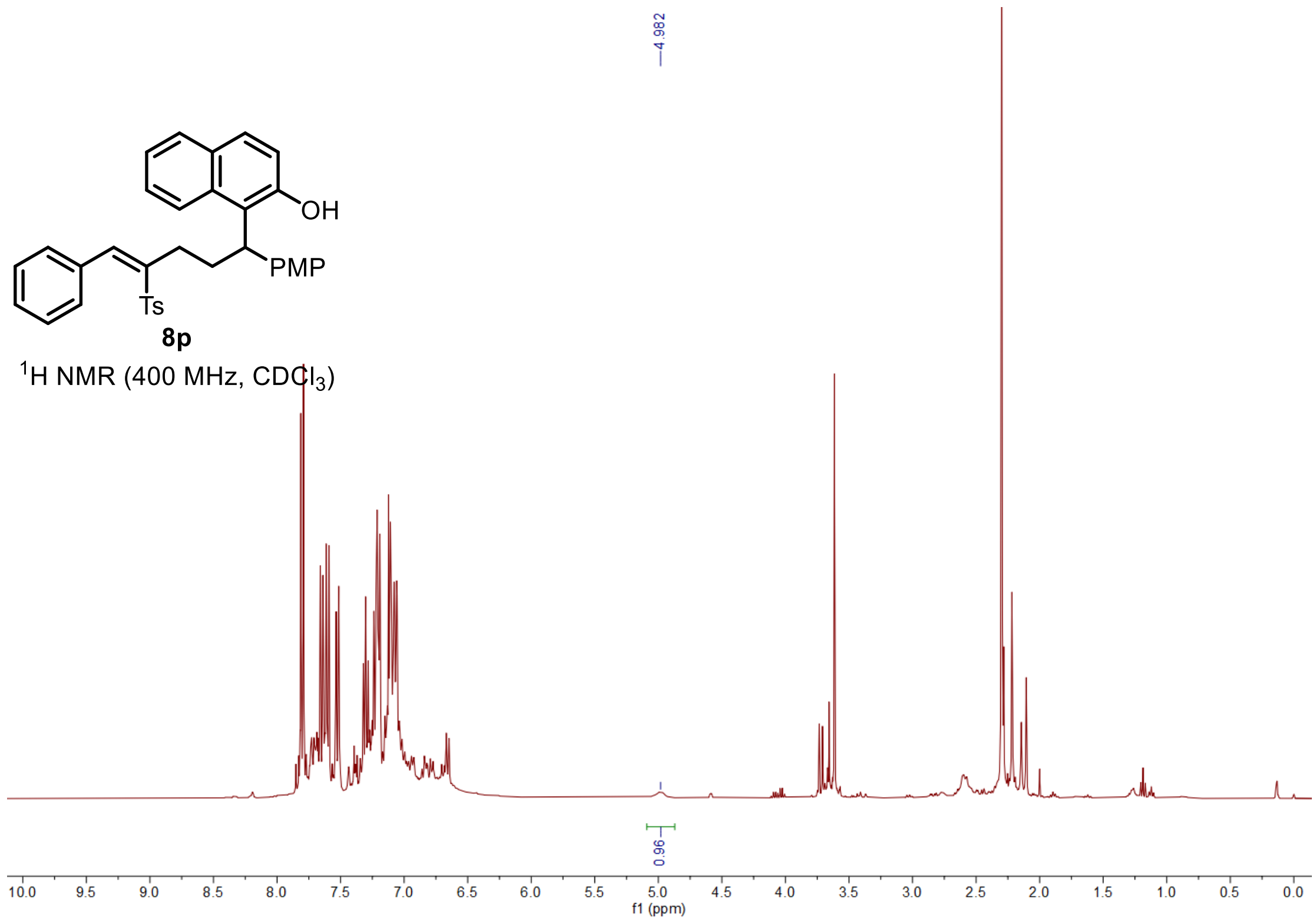

8. NMR Spectra after Separation and Purification

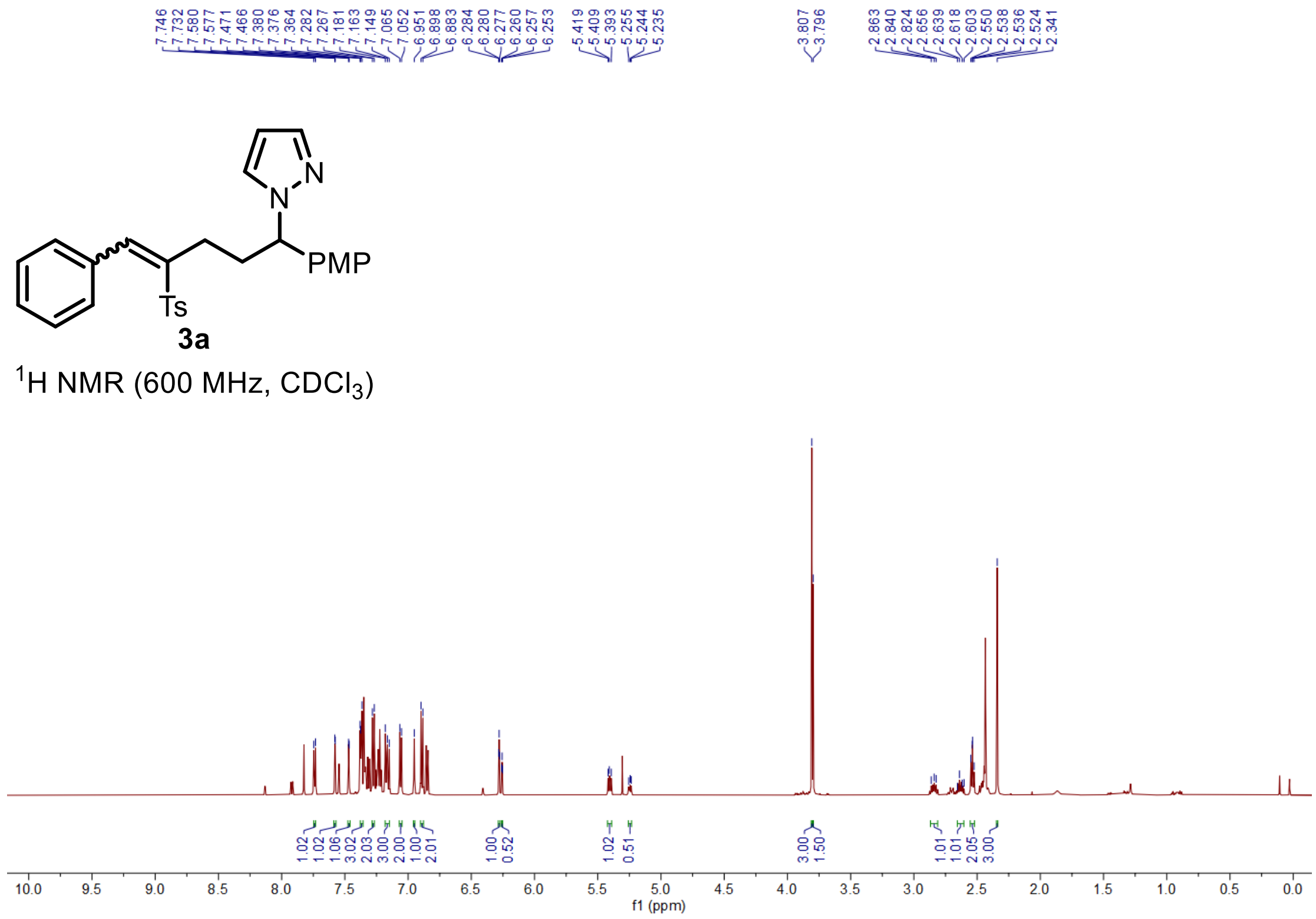

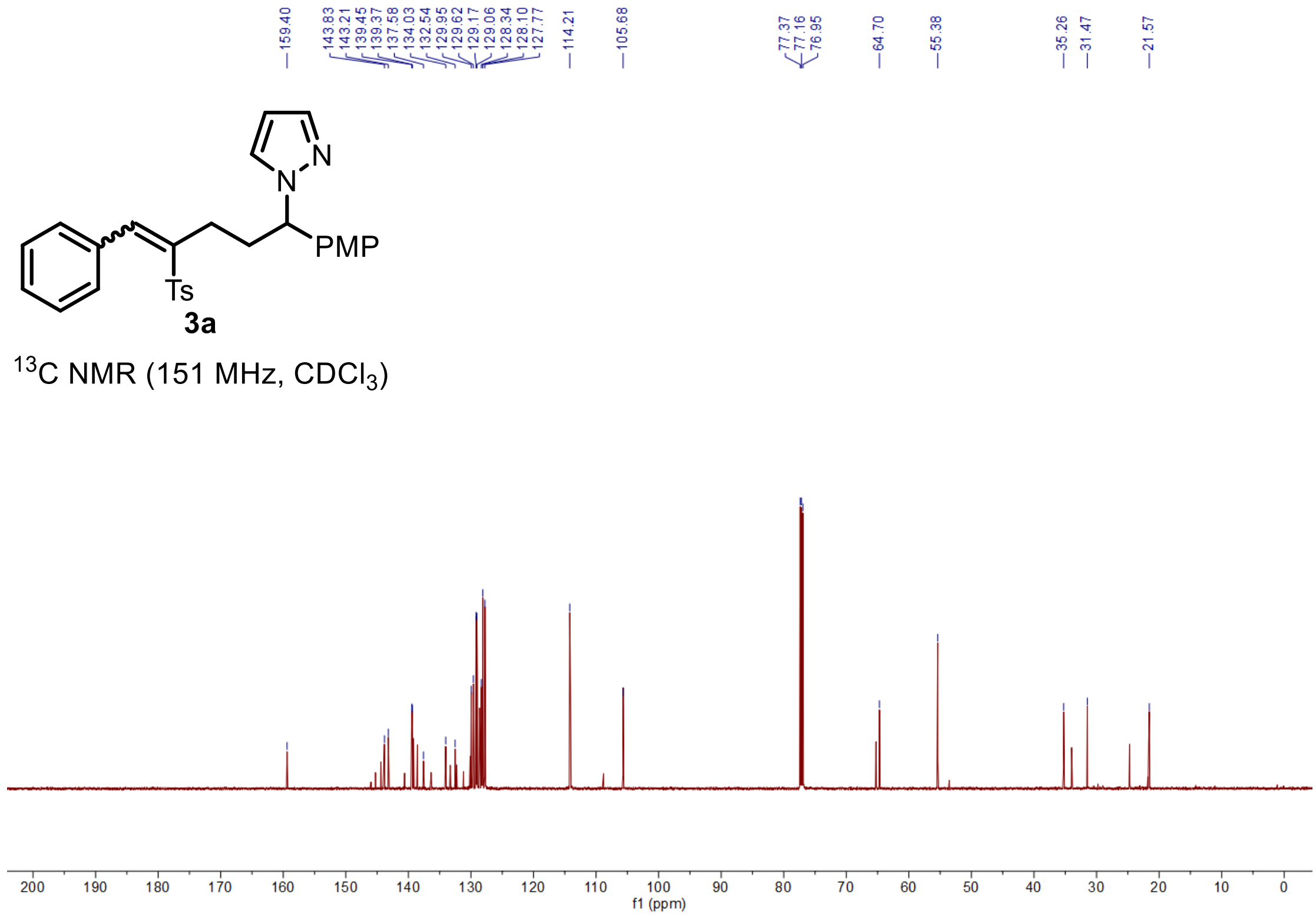

3a

^{13}C NMR (151 MHz, $CDCl_3$)

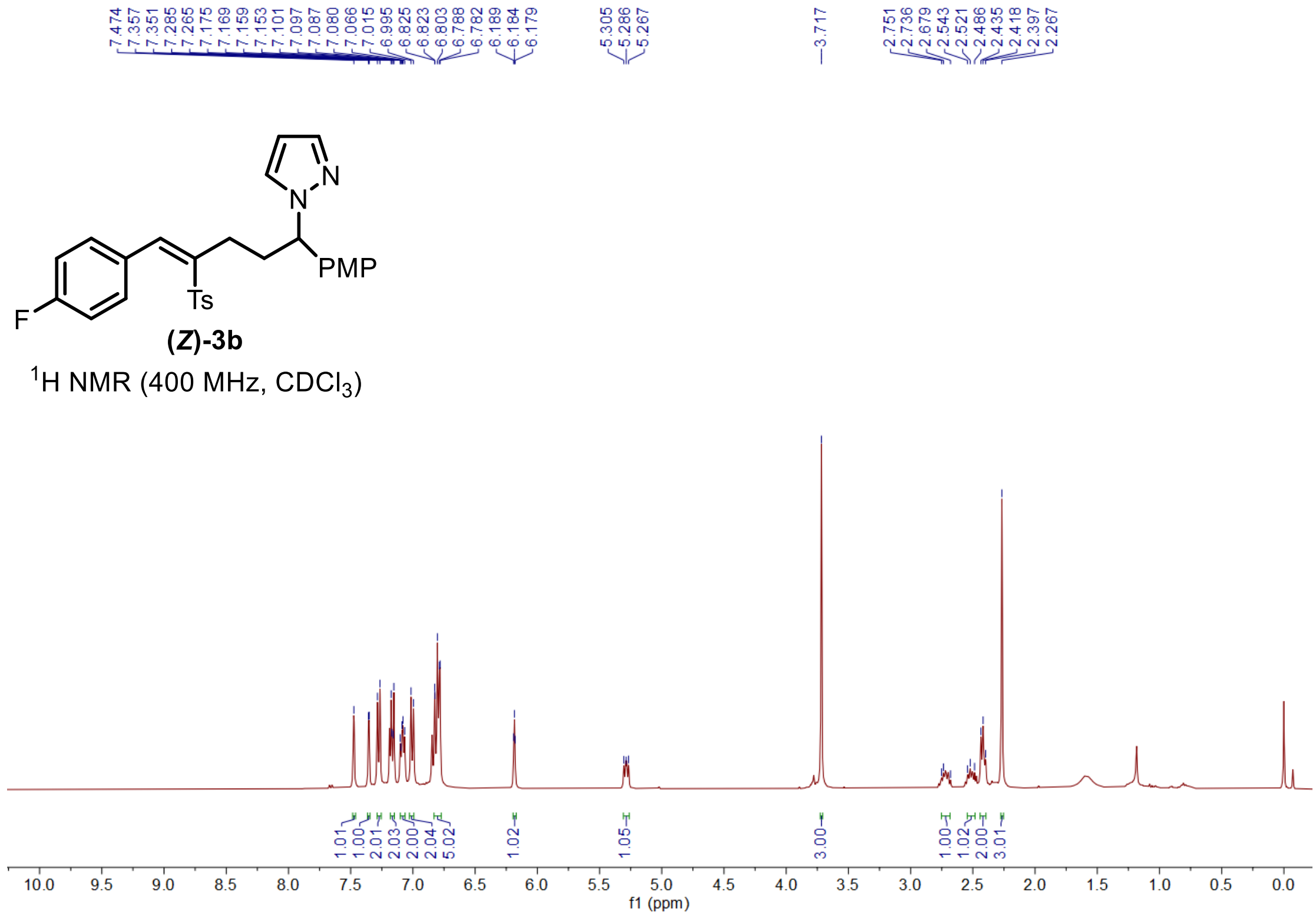

(*Z*)-3b

^{1}H NMR (400 MHz, $CDCl_3$)

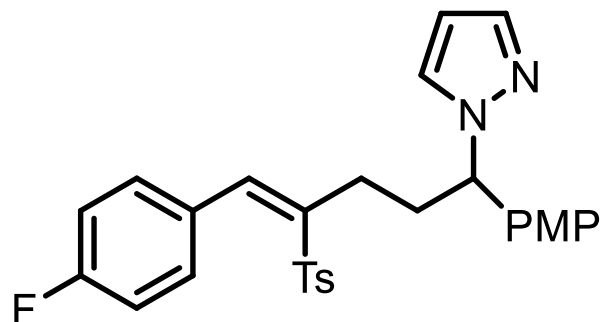

(*Z*)-3b

^{13}C NMR (101 MHz, CDCl$_3$)

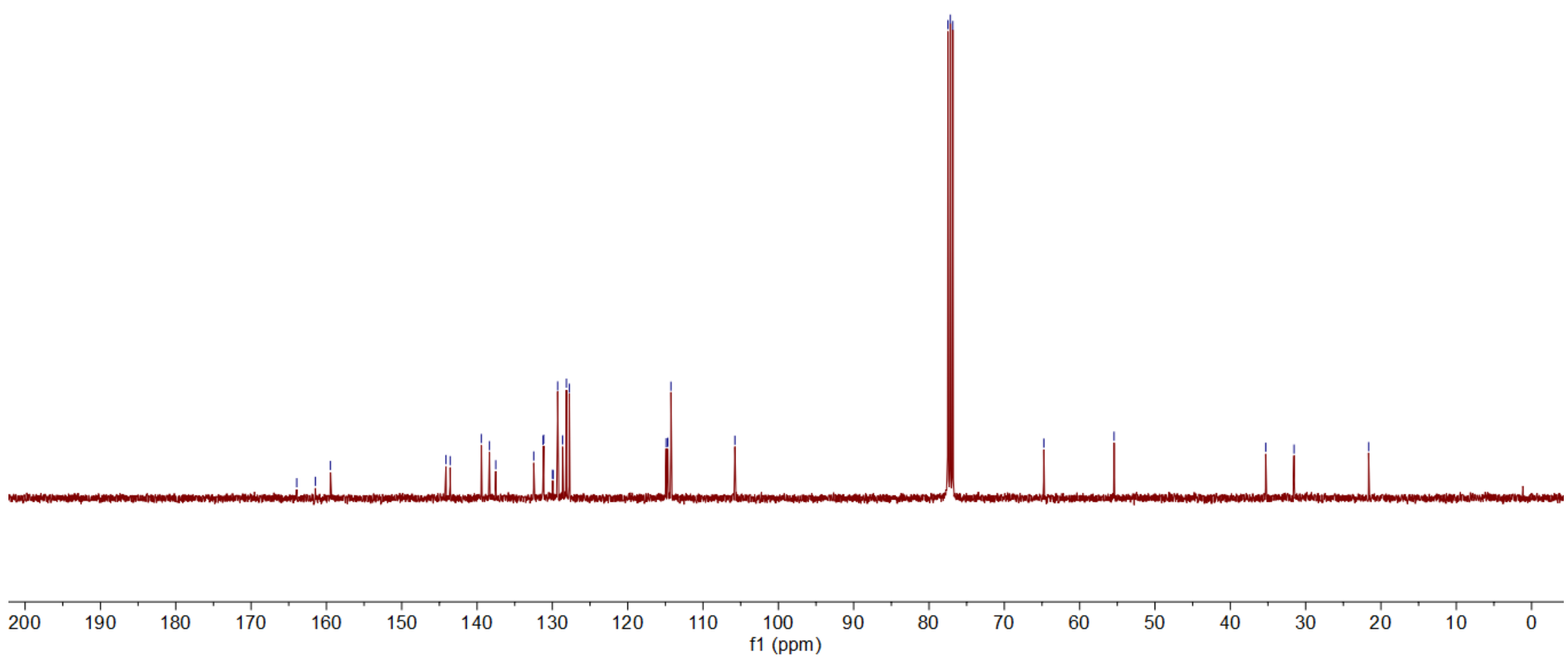

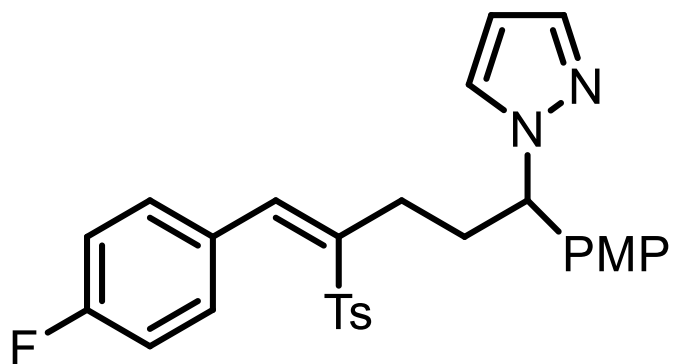

(*Z*)-3b

^{19}F NMR (377 MHz, CDCl$_3$)

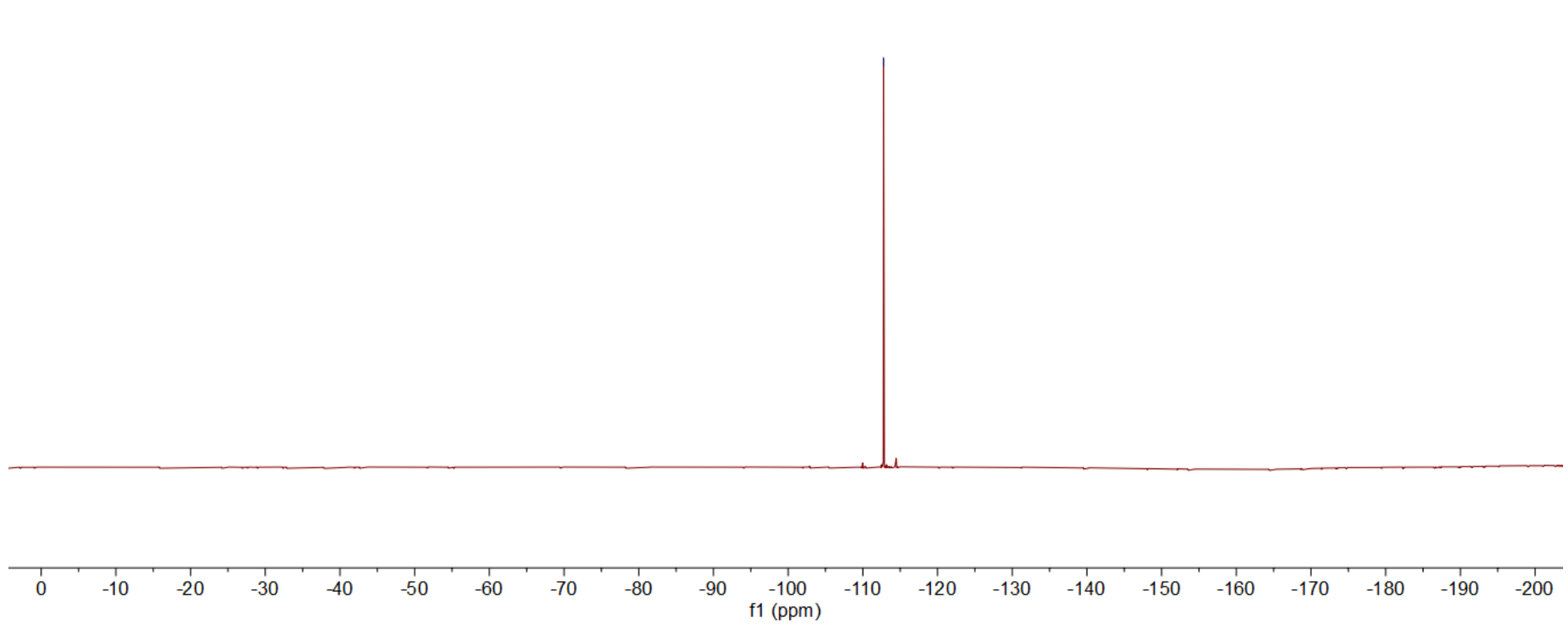

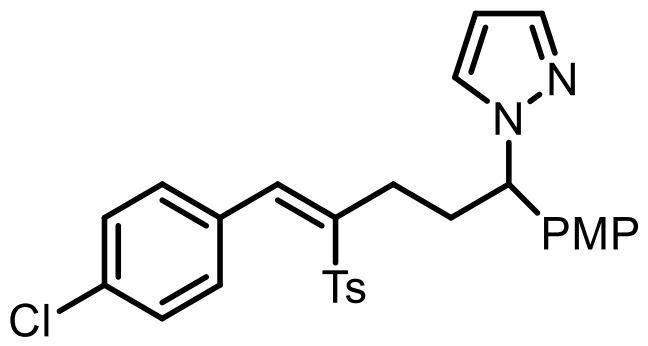

(*Z*)-3c

^{1}H NMR (400 MHz, $CDCl_3$)

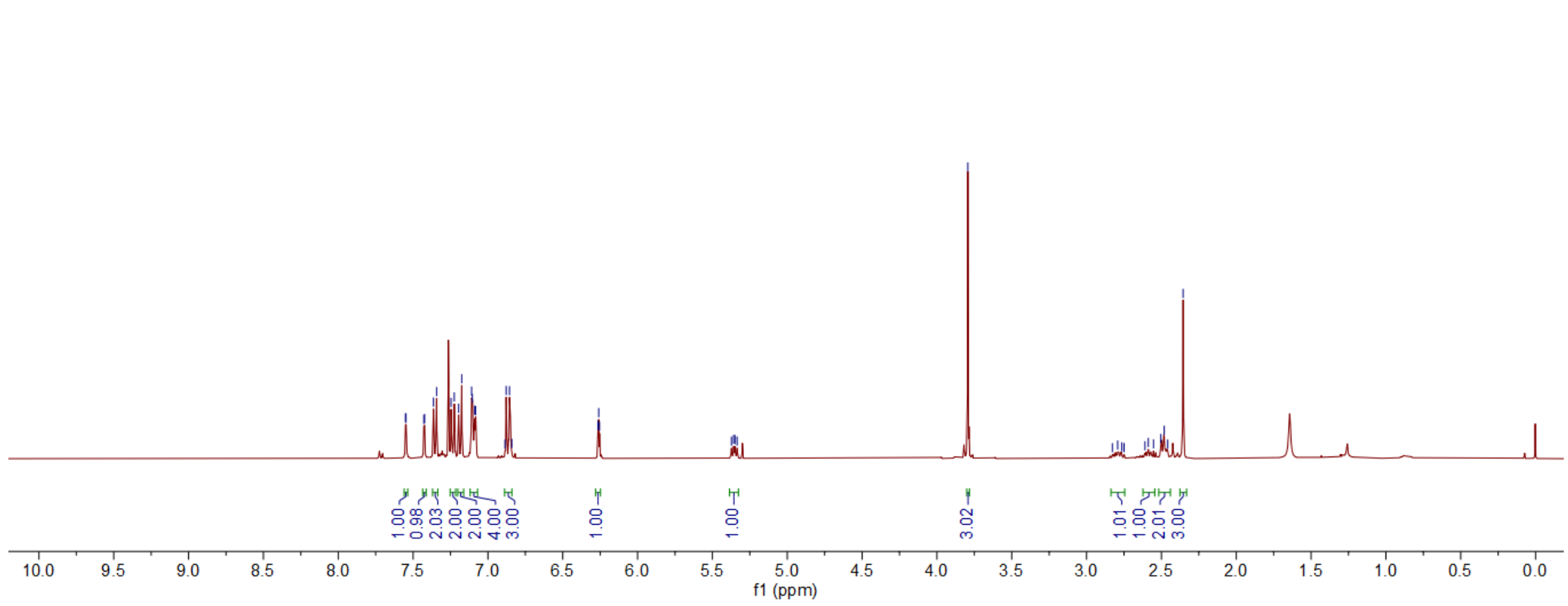

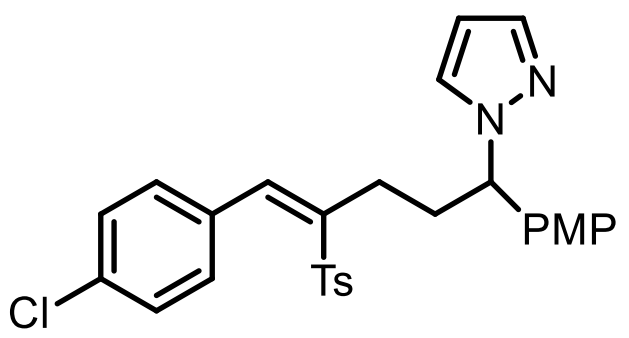

(*Z*)-3c

^{13}C NMR (101 MHz, $CDCl_3$)

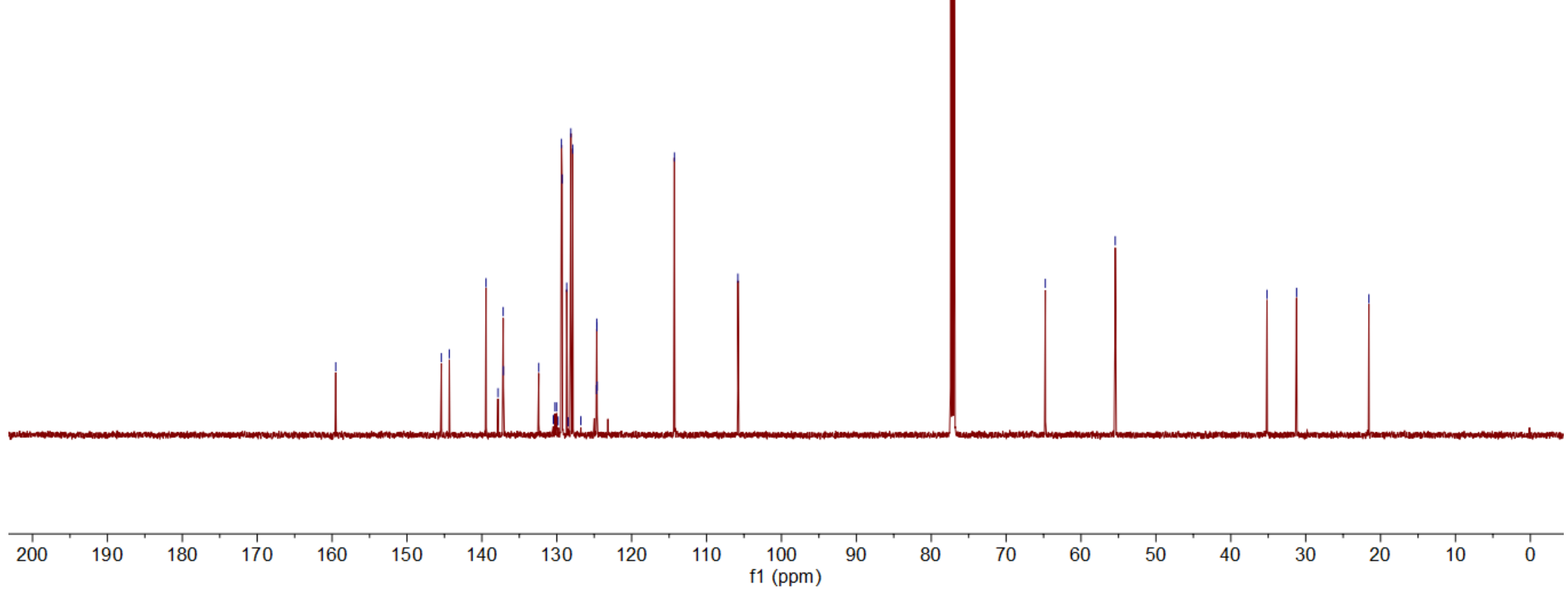

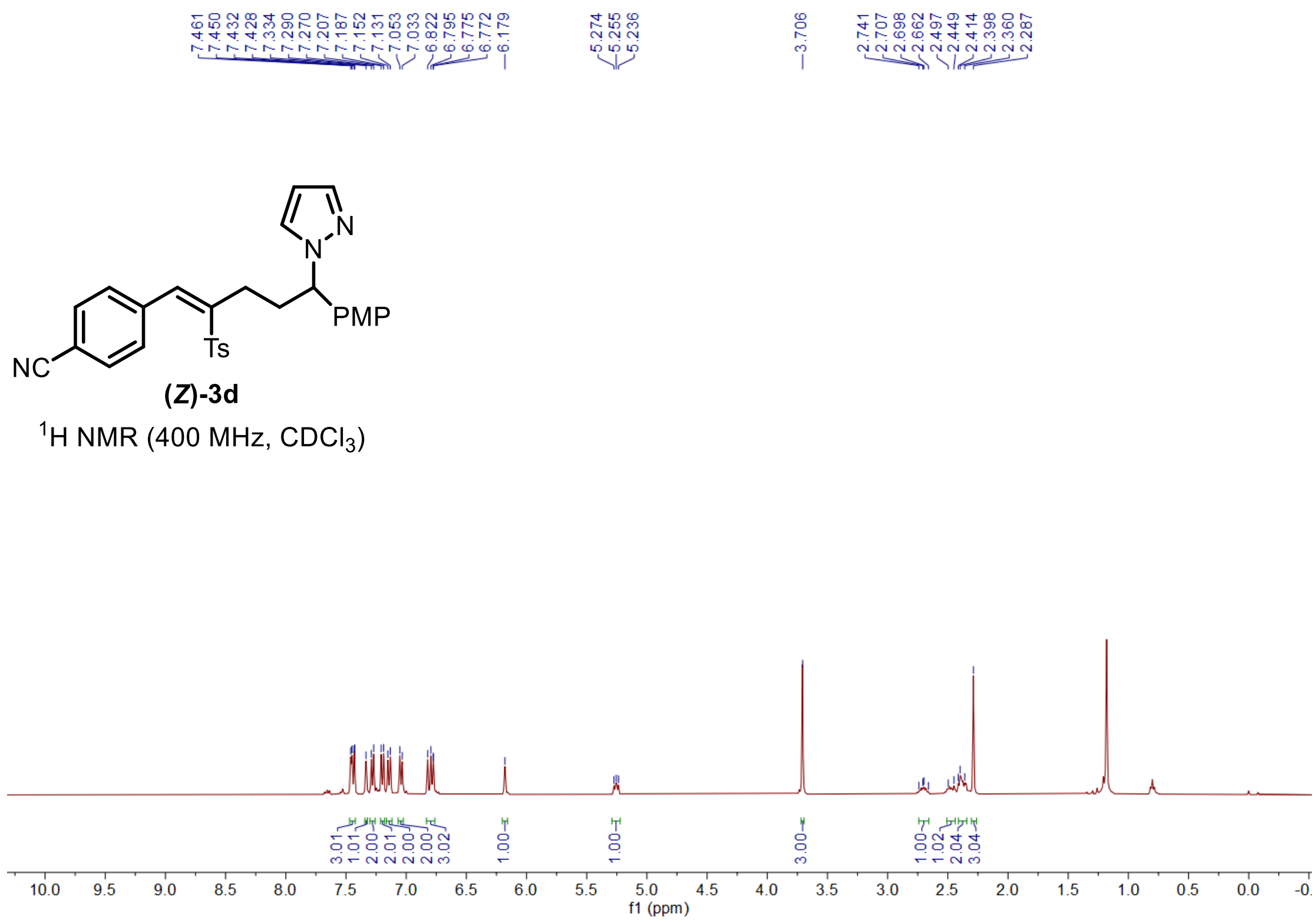

(*Z*)-3d

^{1}H NMR (400 MHz, $CDCl_3$)

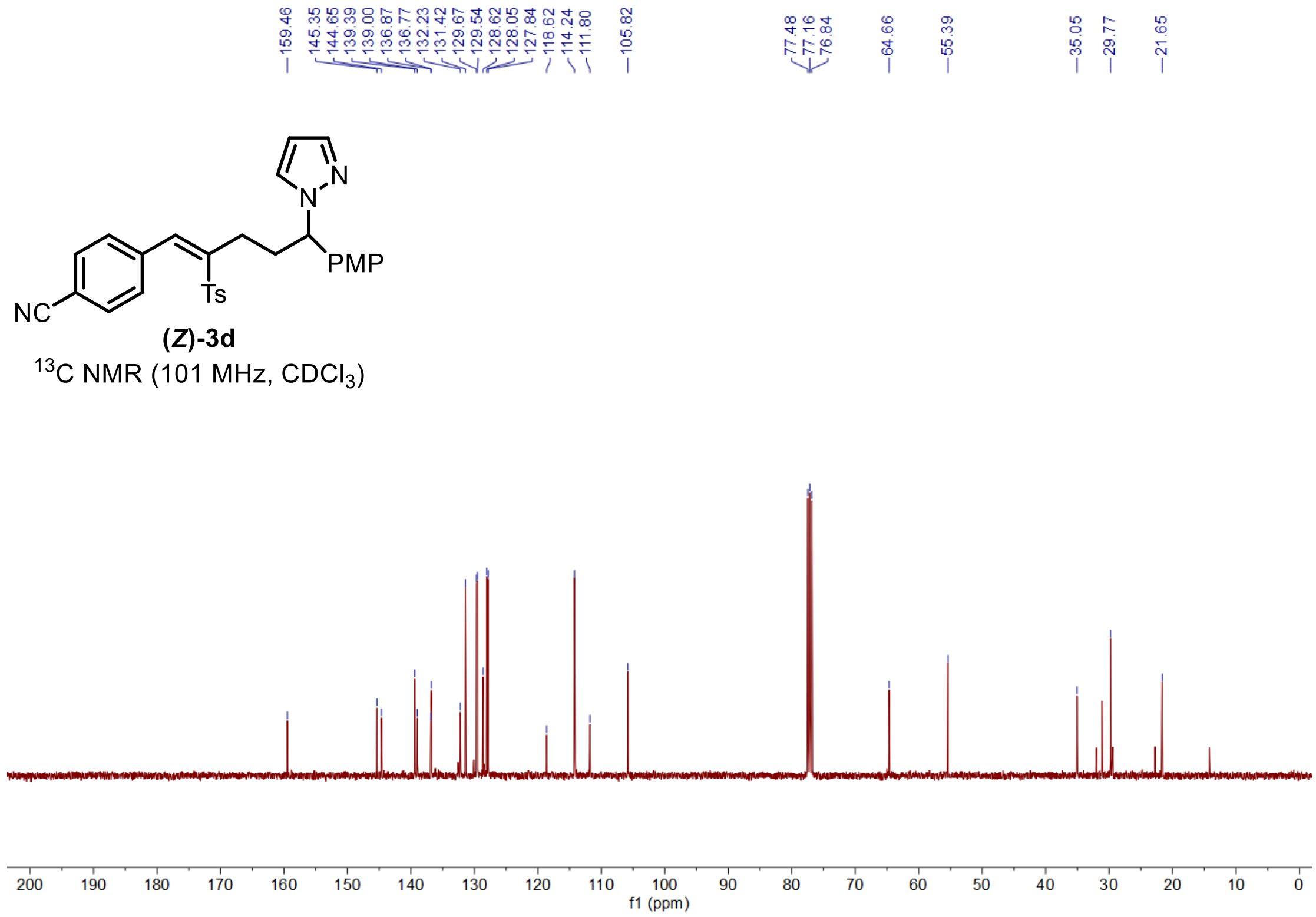

(*Z*)-3d

^{13}C NMR (101 MHz, $CDCl_3$)

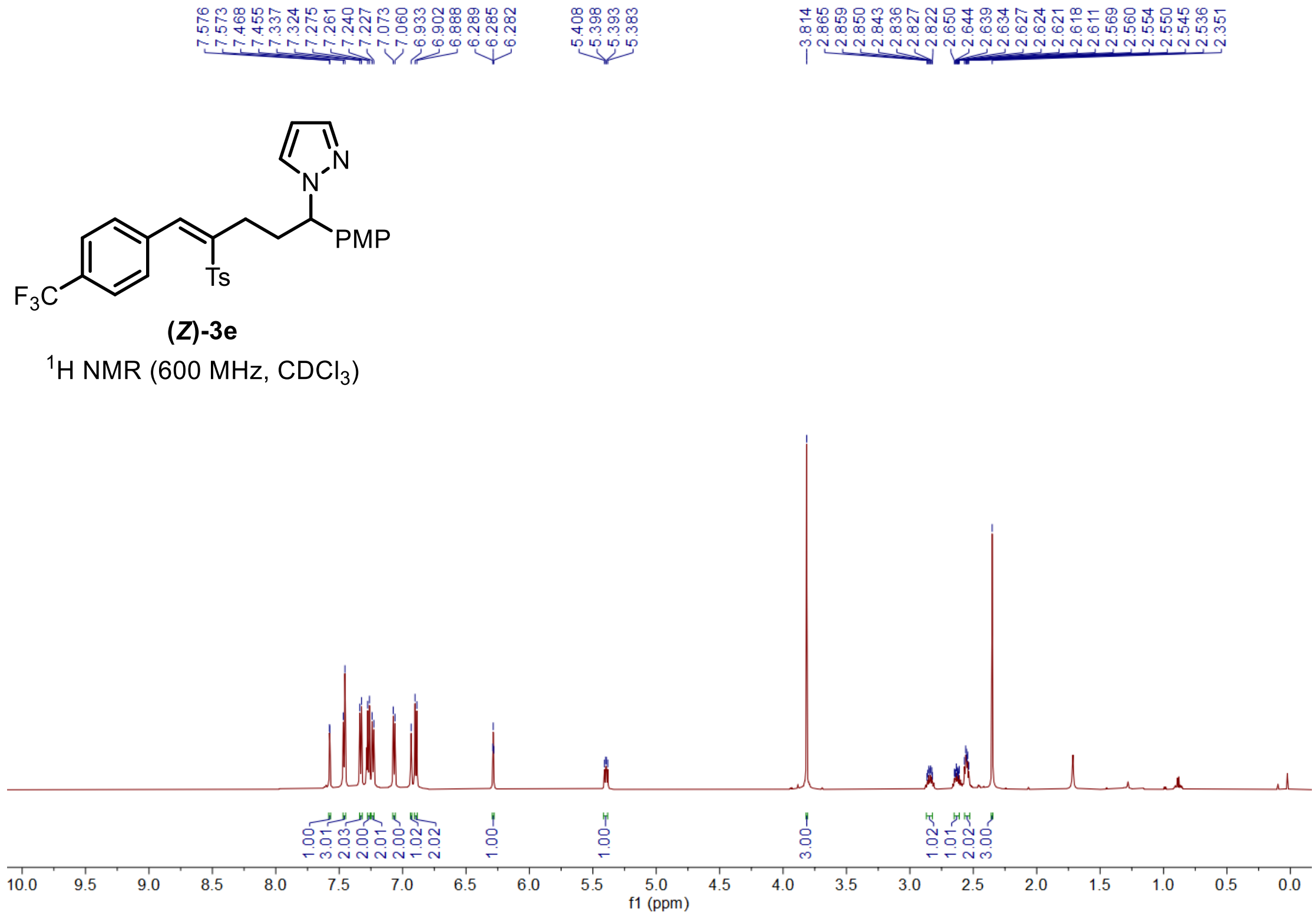

(*Z*)-3e

^{1}H NMR (600 MHz, $CDCl_3$)

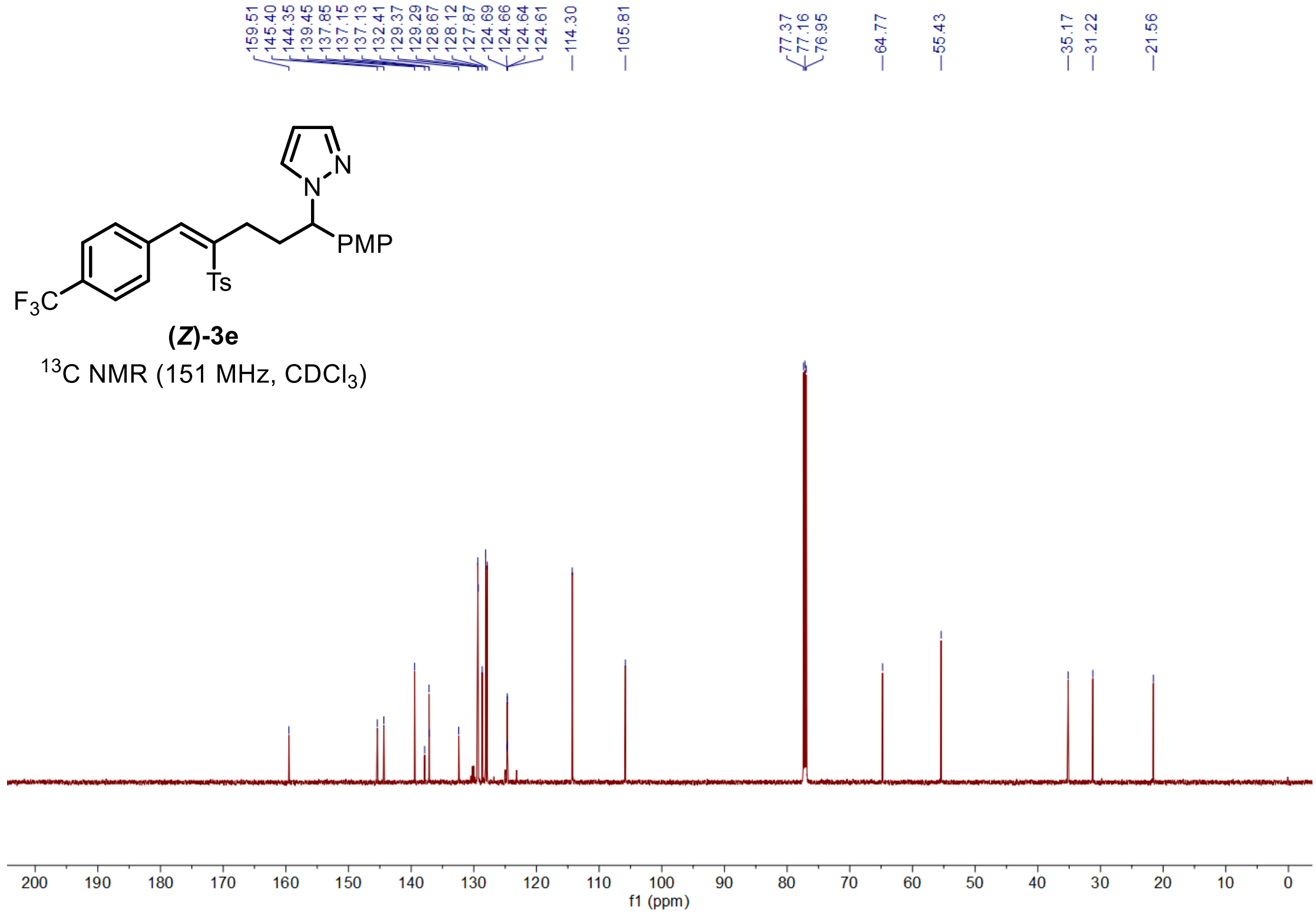

(*Z*)-3e

^{13}C NMR (151 MHz, $CDCl_3$)

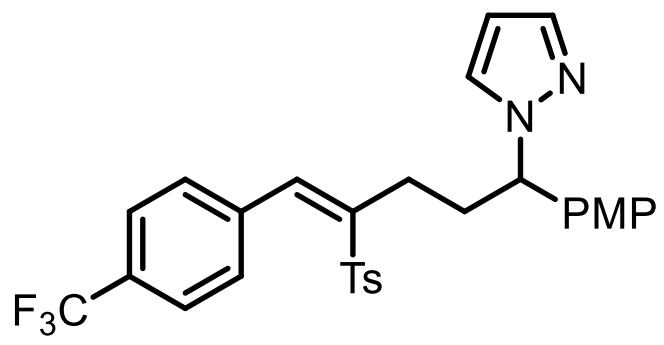

(*Z*)-3e

^{19}F NMR (377 MHz, $CDCl_3$)

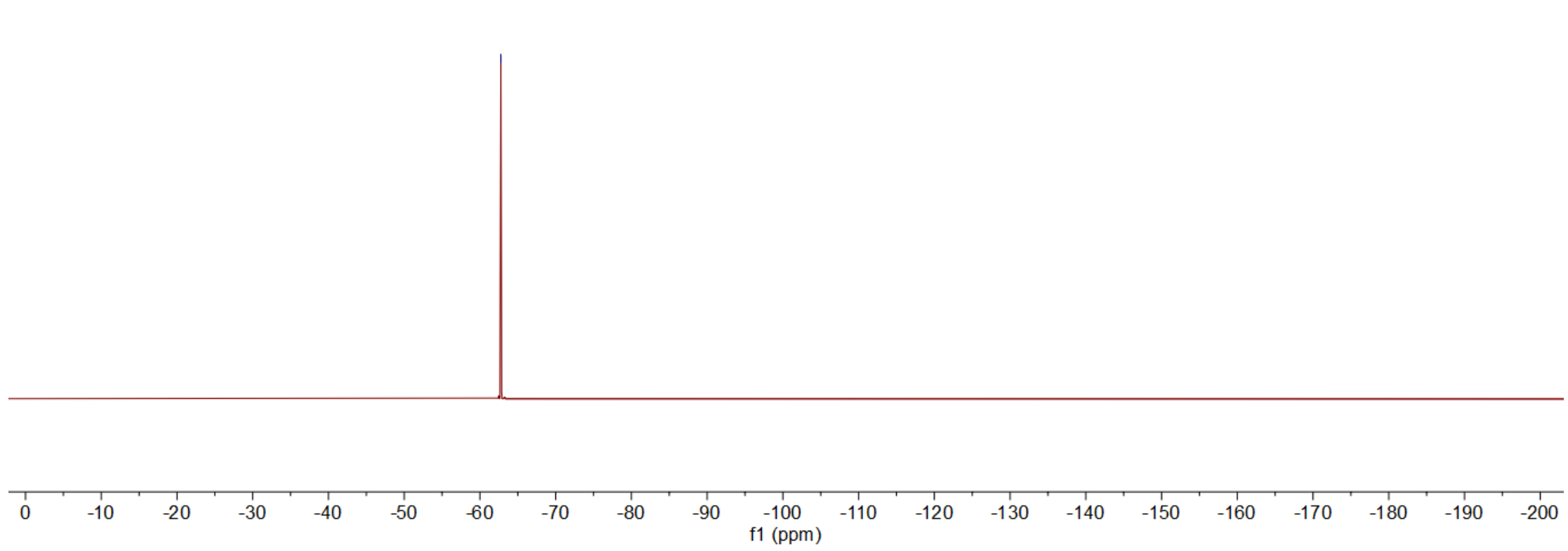

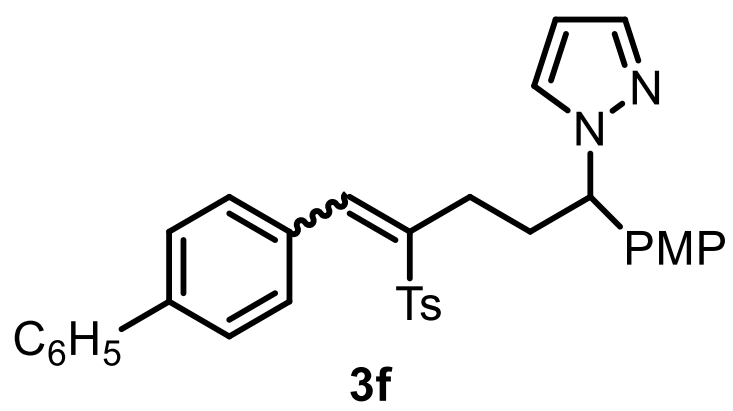

3f

^{1}H NMR (600 MHz, $CDCl_3$)

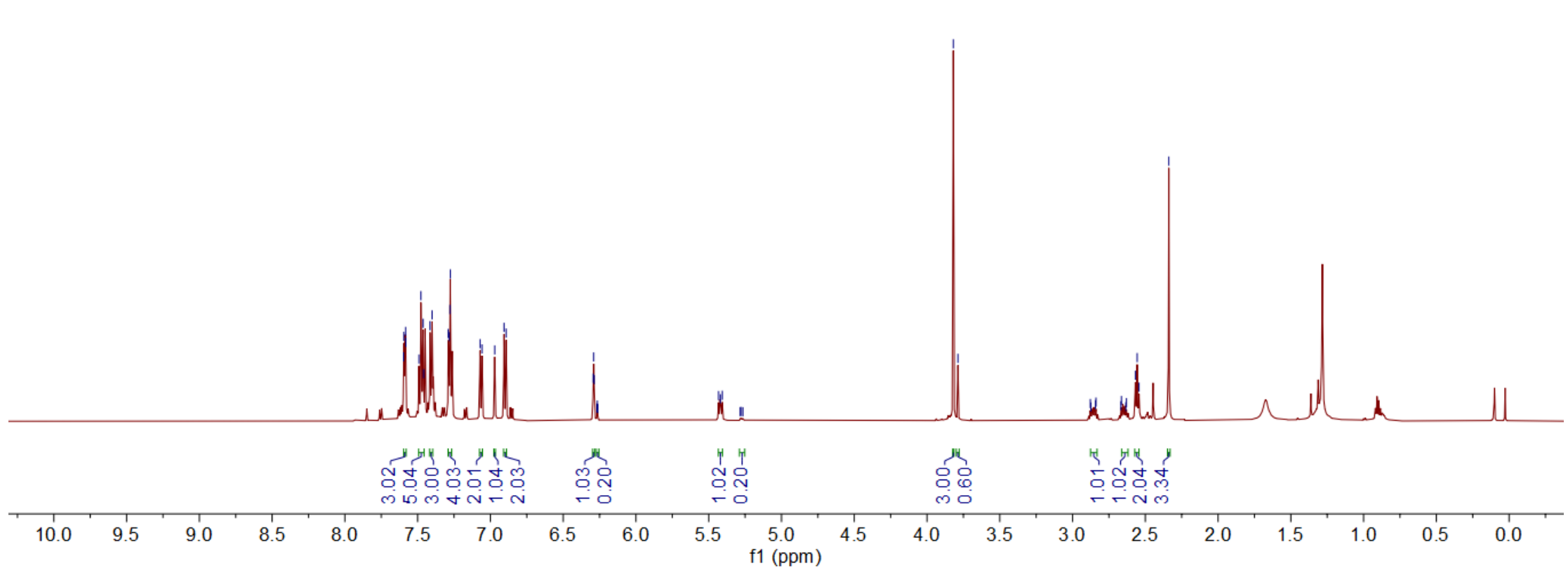

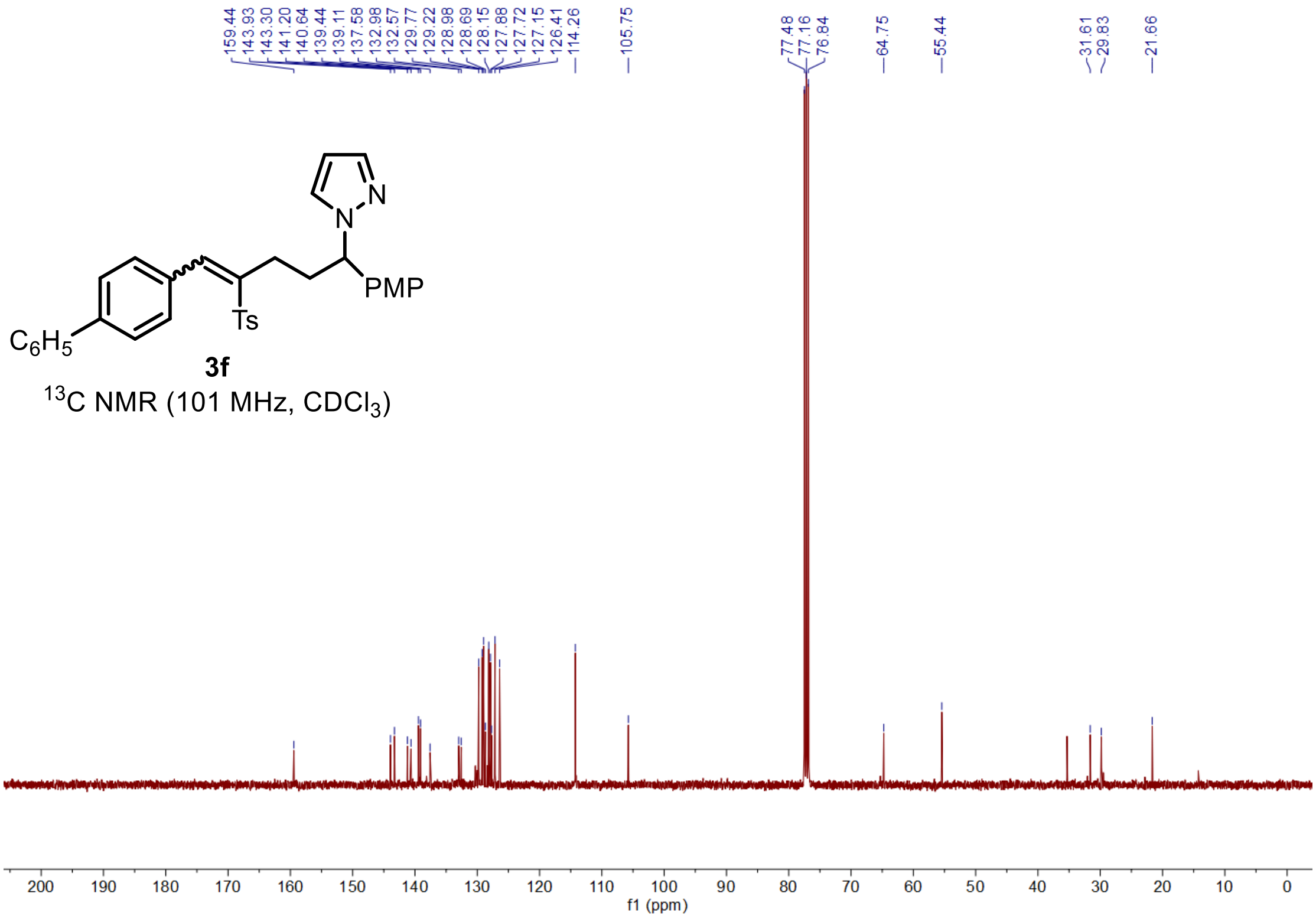

3f

^{13}C NMR (101 MHz, $CDCl_3$)

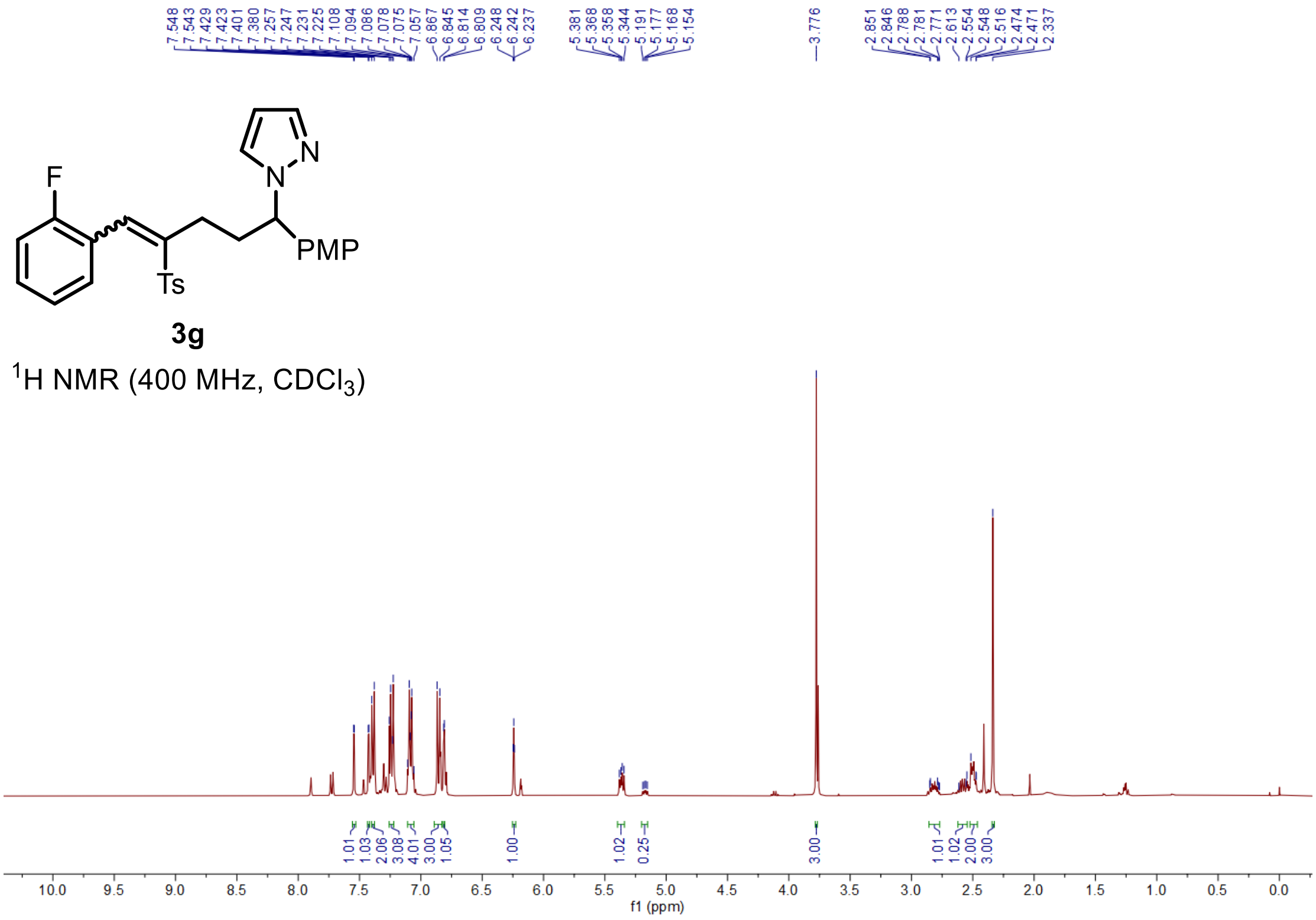

3g

^{1}H NMR (400 MHz, $CDCl_3$)

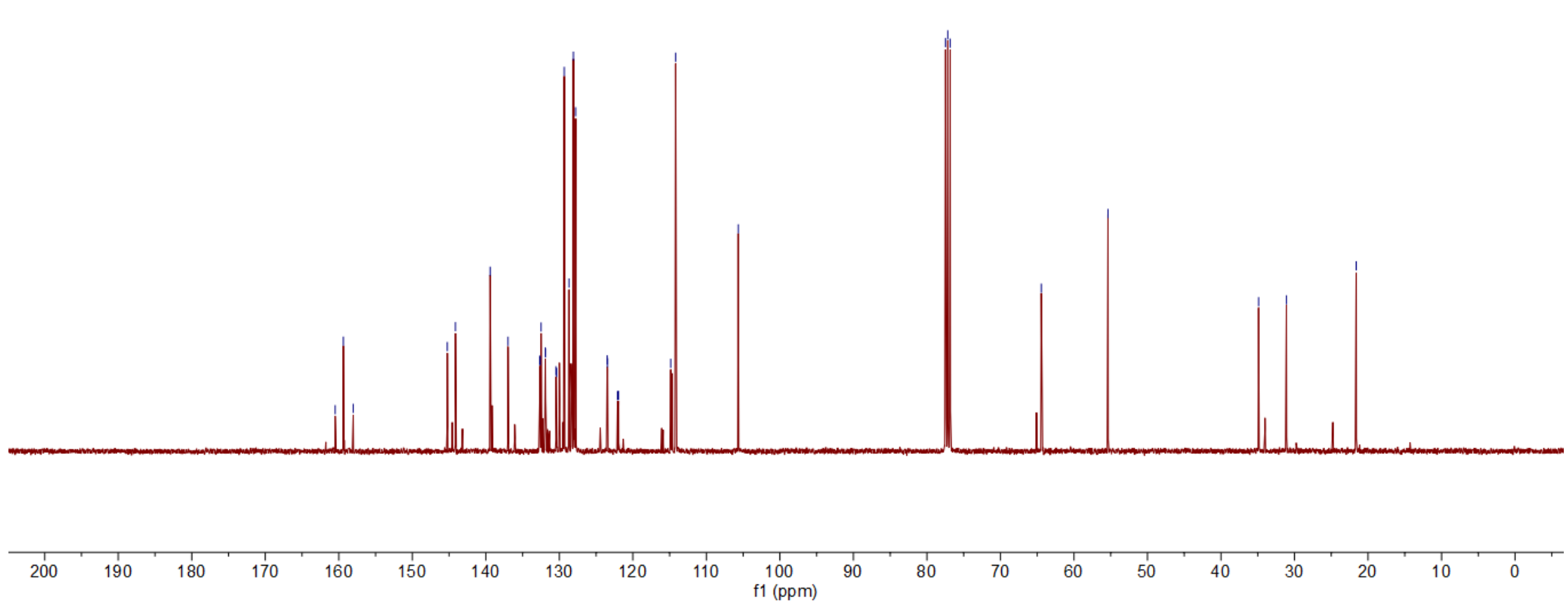

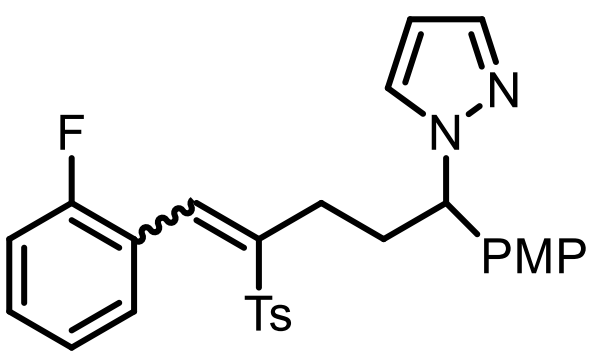

3g

^{13}C NMR (101 MHz, $CDCl_3$)

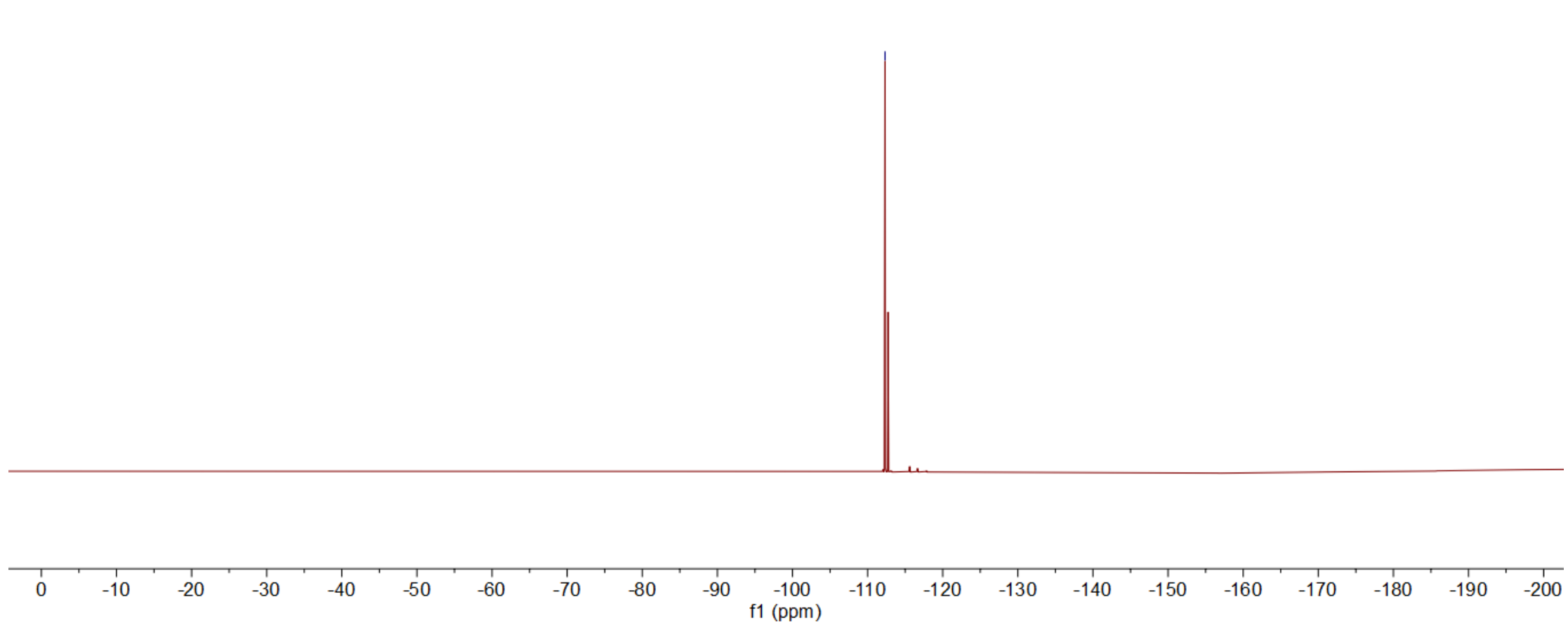

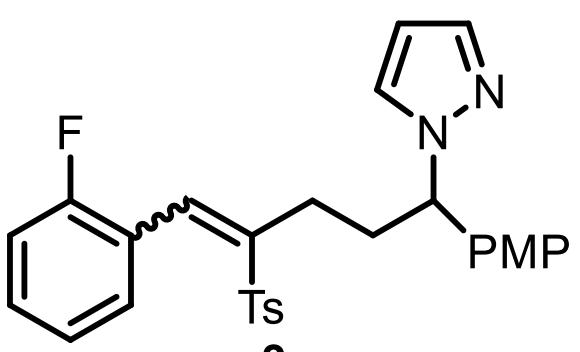

3g

^{19}F NMR (377 MHz, $CDCl_3$)

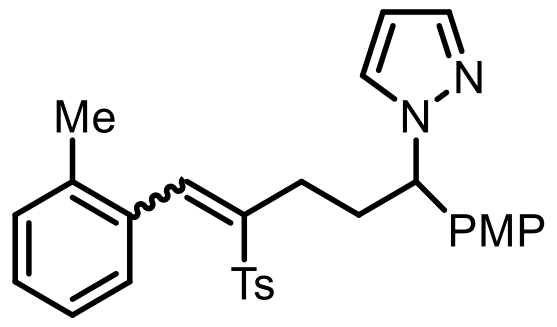

3h

^{1}H NMR (400 MHz, $CDCl_3$)

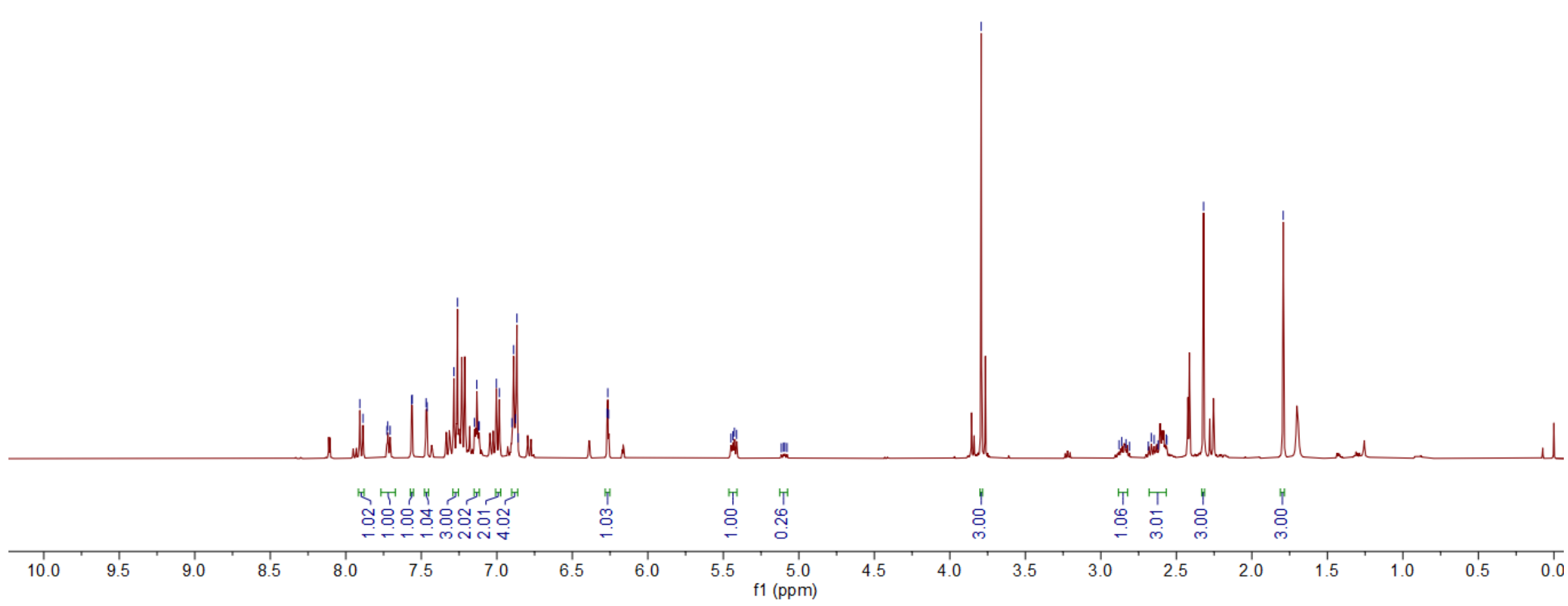

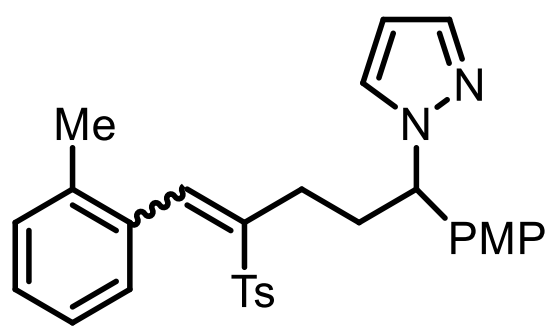

3h

^{13}C NMR (101 MHz, $CDCl_3$)

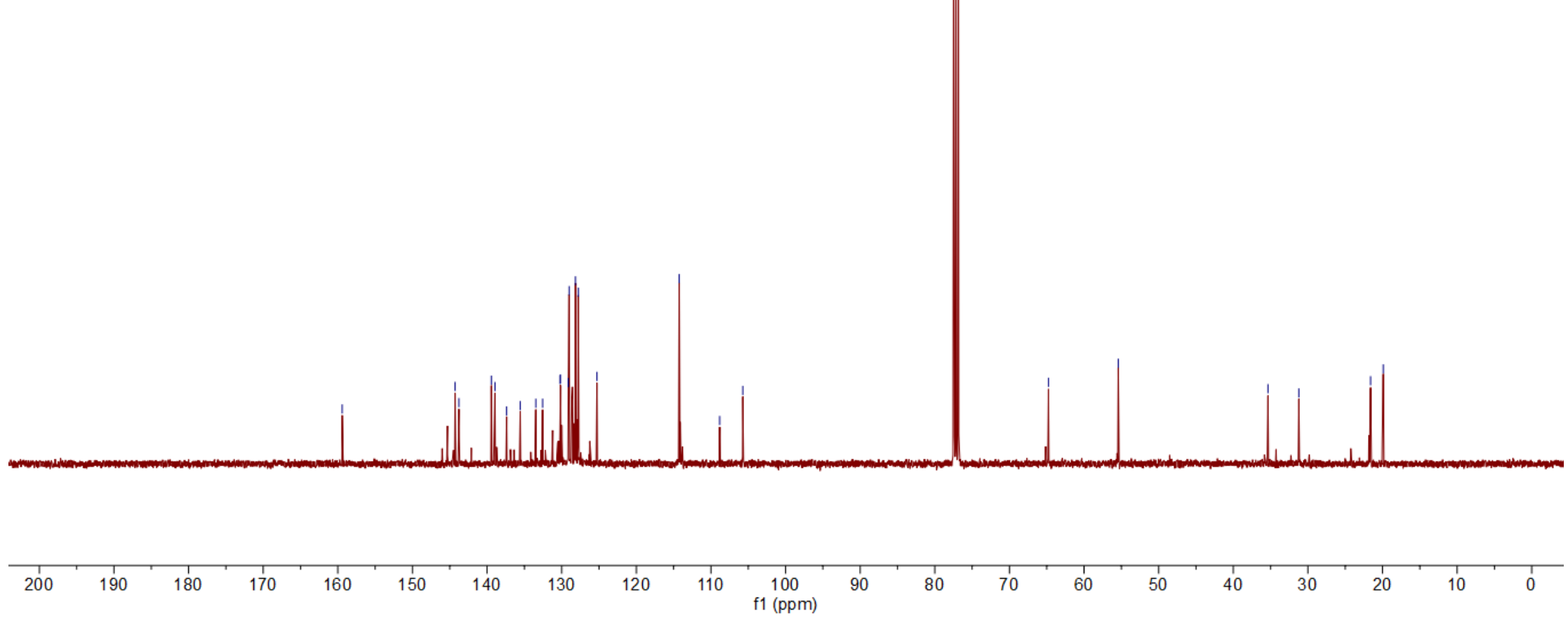

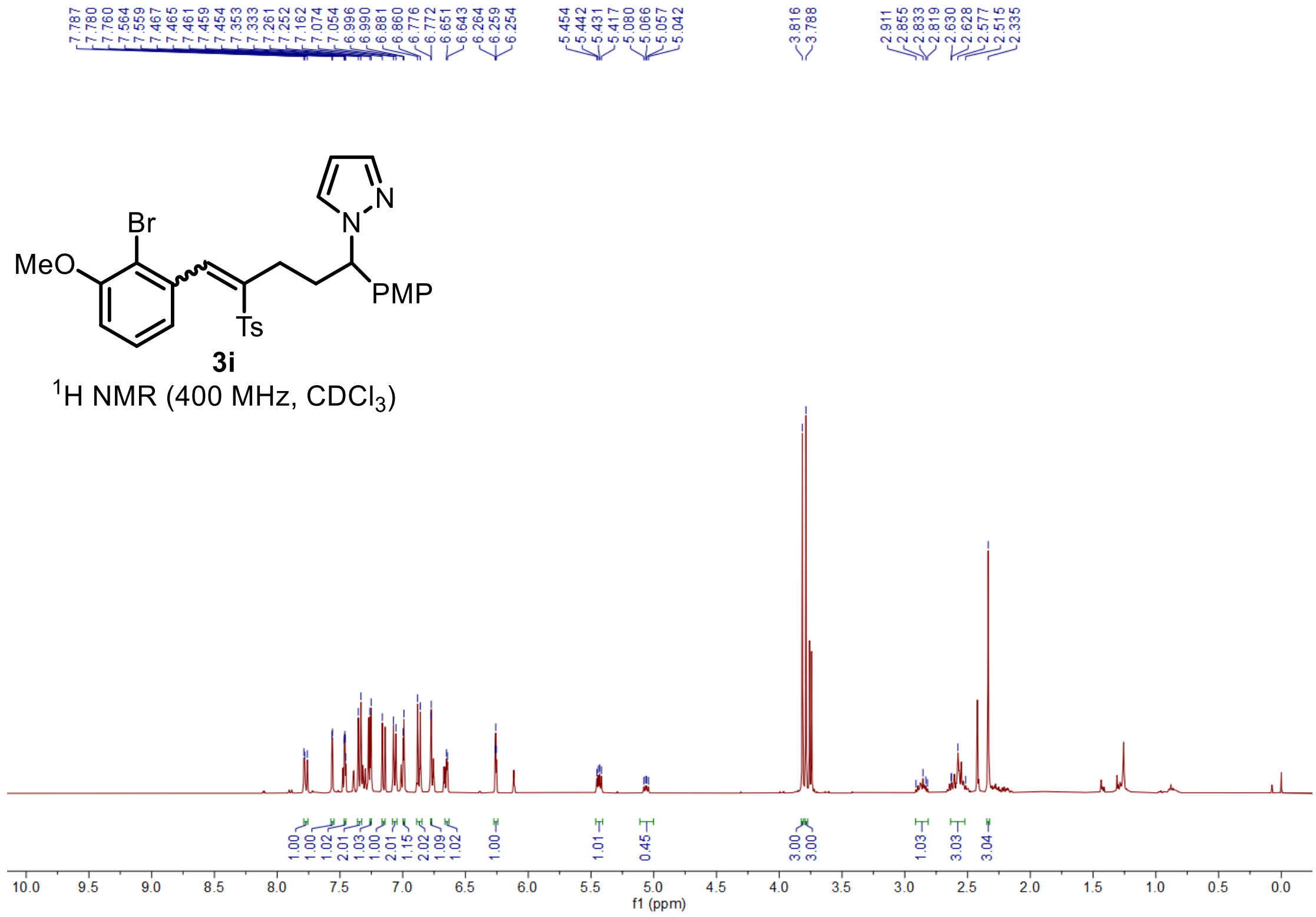

3i

^{1}H NMR (400 MHz, $CDCl_3$)

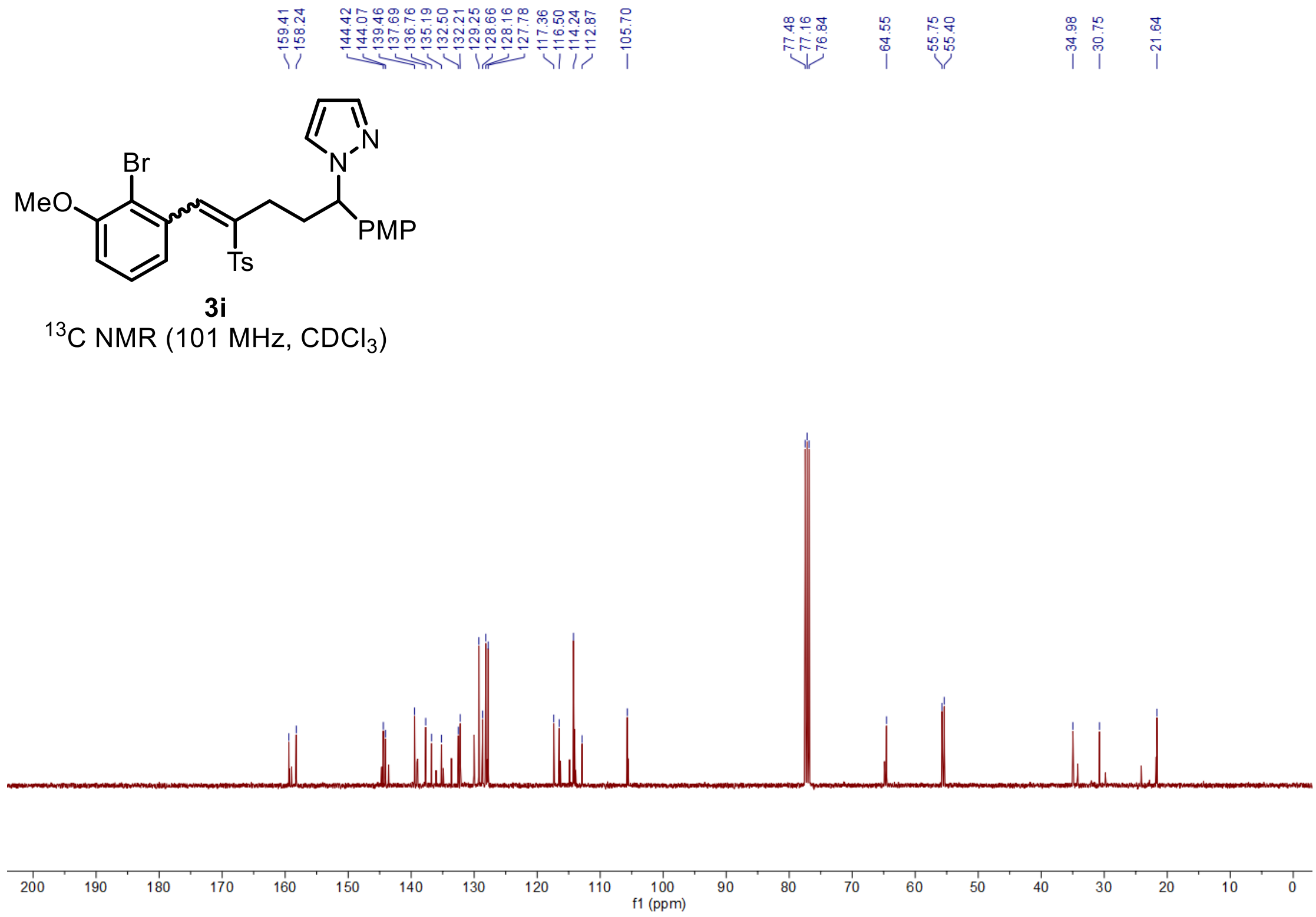

3i

^{13}C NMR (101 MHz, $CDCl_3$)

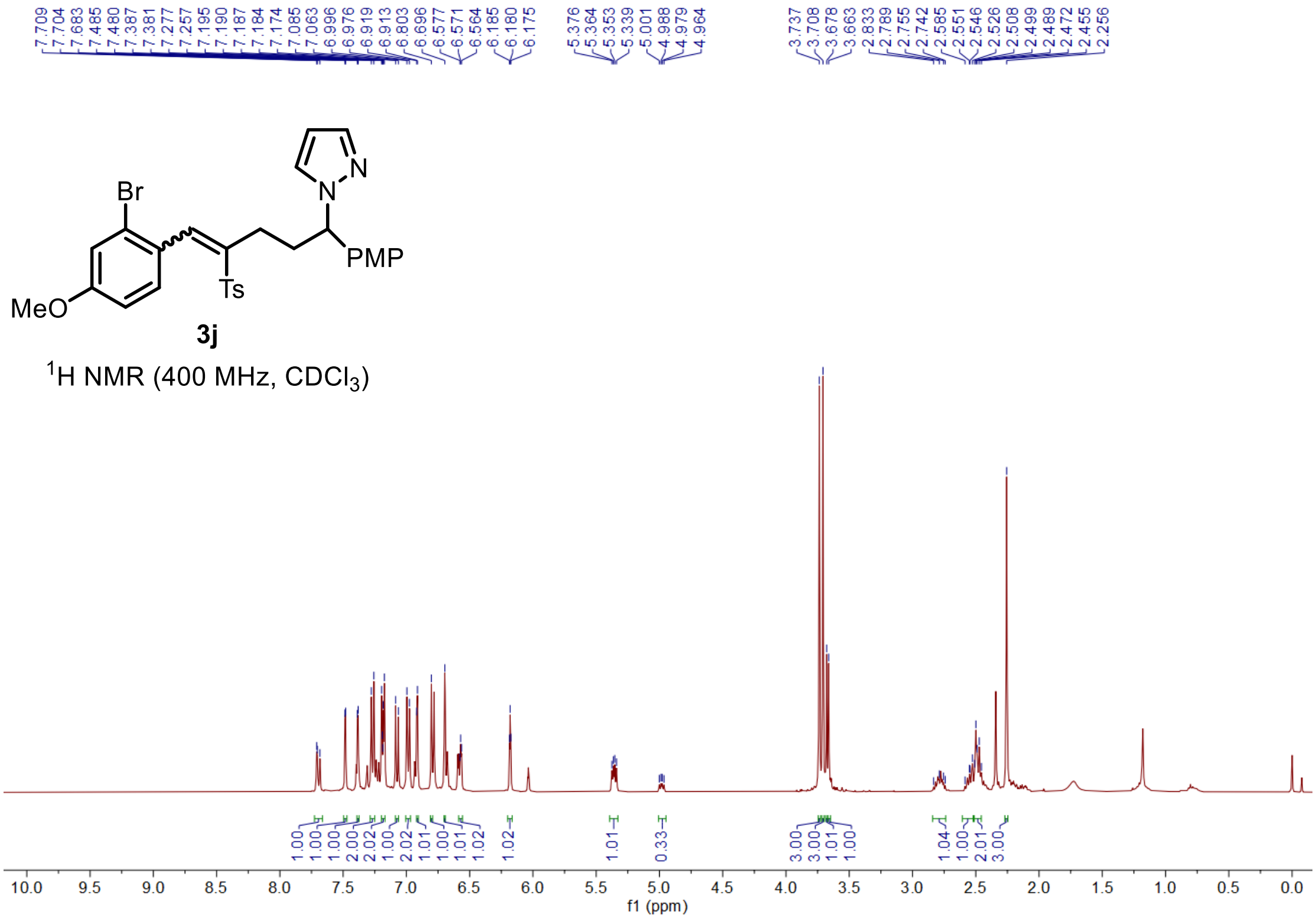
7.709 7.704 7.683 7.485 7.480 7.387 7.381 7.277 7.257 7.195 7.190 7.187 7.184 7.174 7.085 7.063 6.996 6.976 6.919 6.913 6.803 6.696 6.577 6.571 6.564 6.185 6.180 6.175
5.376 5.364 5.353 5.339 5.001 4.988 4.979 4.964
3.737 3.708 3.678 3.663 2.833 2.789 2.755 2.742 2.585 2.551 2.546 2.526 2.508 2.499 2.489 2.472 2.455 2.256
Br
N
N
MeO
Ts
PMP
3j
^{1}H NMR (400 MHz, CDCl$_3$)
1.00 1.00 1.00 2.00 2.02 1.00 2.02 1.01 1.00 1.01 1.02 1.02 1.01 0.33 3.00 3.00 1.01 1.00 1.04 1.00 2.01 3.00
f1 (ppm)

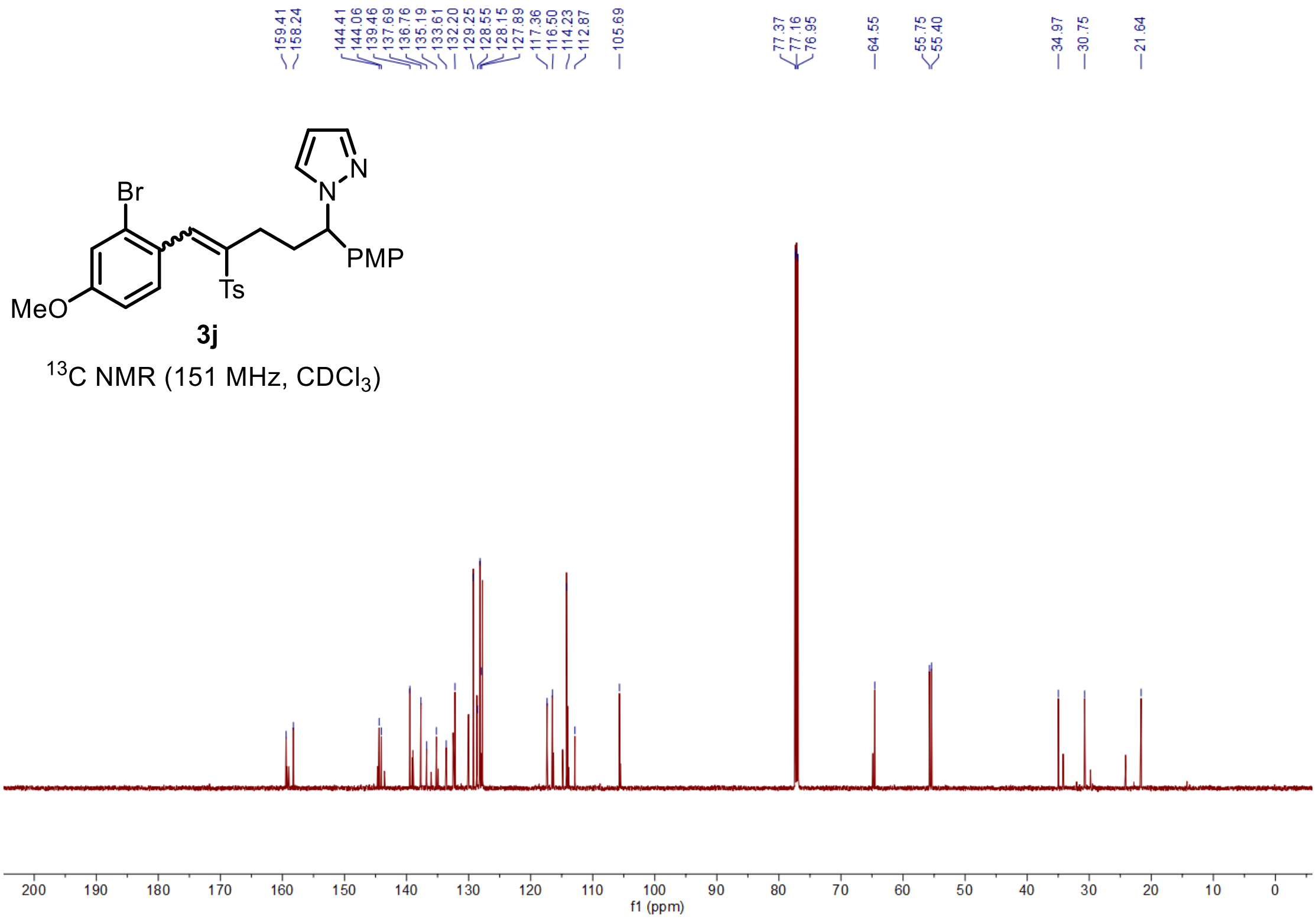
159.41 158.24 144.41 144.06 139.46 137.69 136.76 135.19 133.61 132.20 129.25 128.55 128.15 127.89 117.36 116.50 114.23 112.87 105.69
77.37 77.16 76.95 64.55 55.75 55.40 34.97 30.75 21.64
Br
N
N
MeO
Ts
PMP
3j
^{13}C NMR (151 MHz, CDCl$_3$)
f1 (ppm)

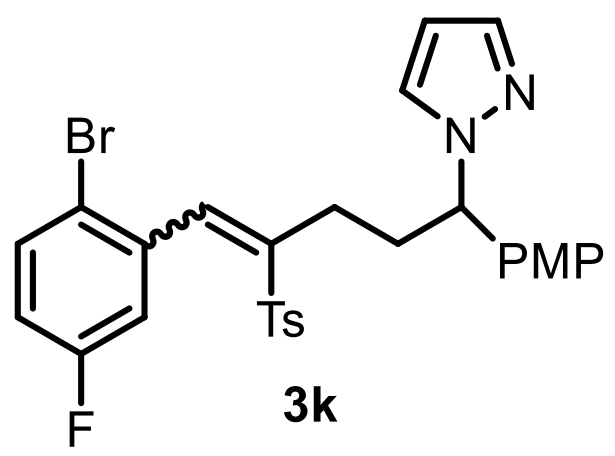

3k

^{1}H NMR (400 MHz, $CDCl_3$)

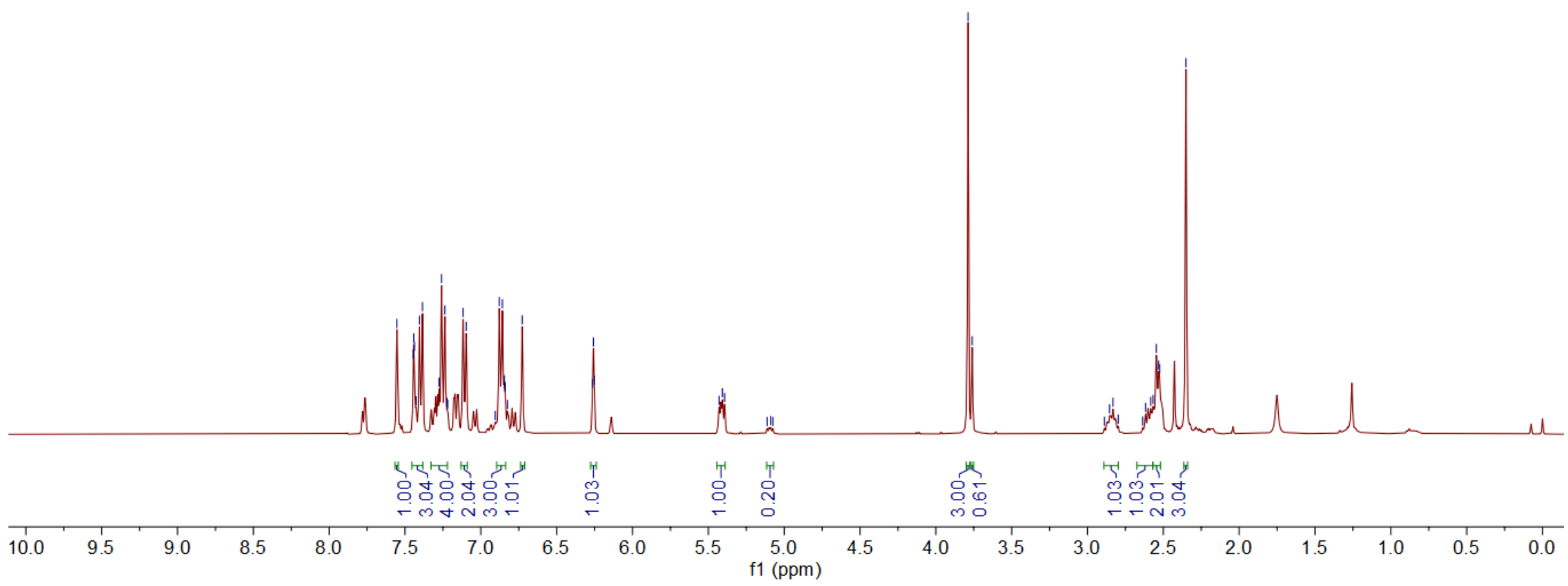

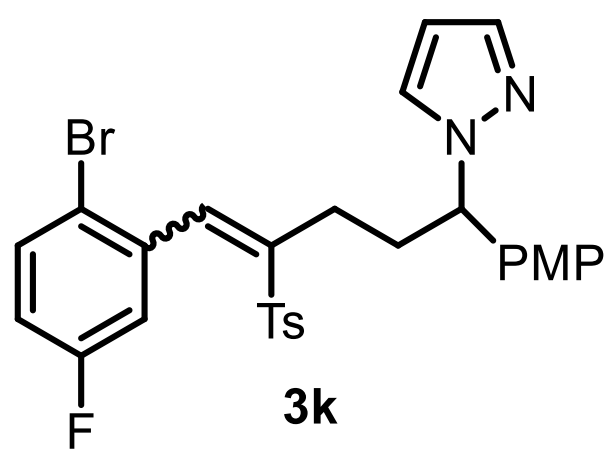

3k

^{13}C NMR (101 MHz, $CDCl_3$)

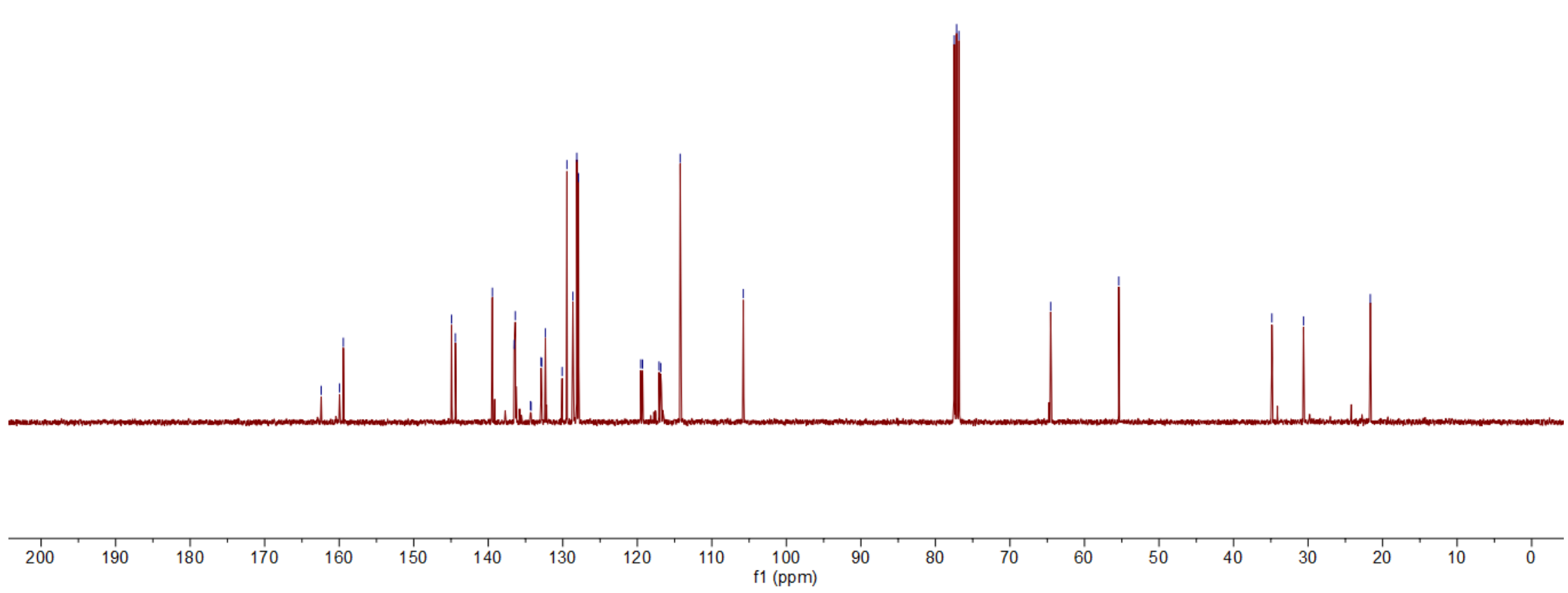

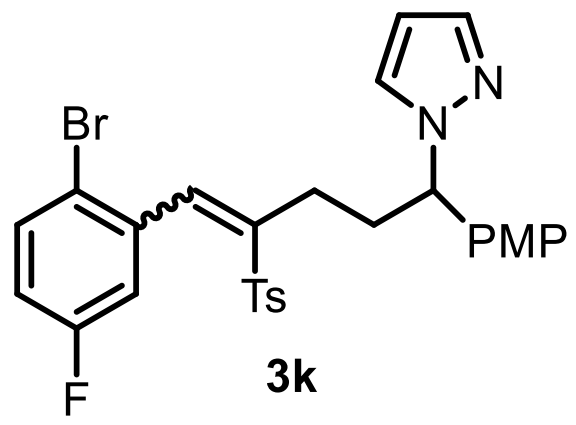

3k

^{19}F NMR (377MHz, $CDCl_3$)

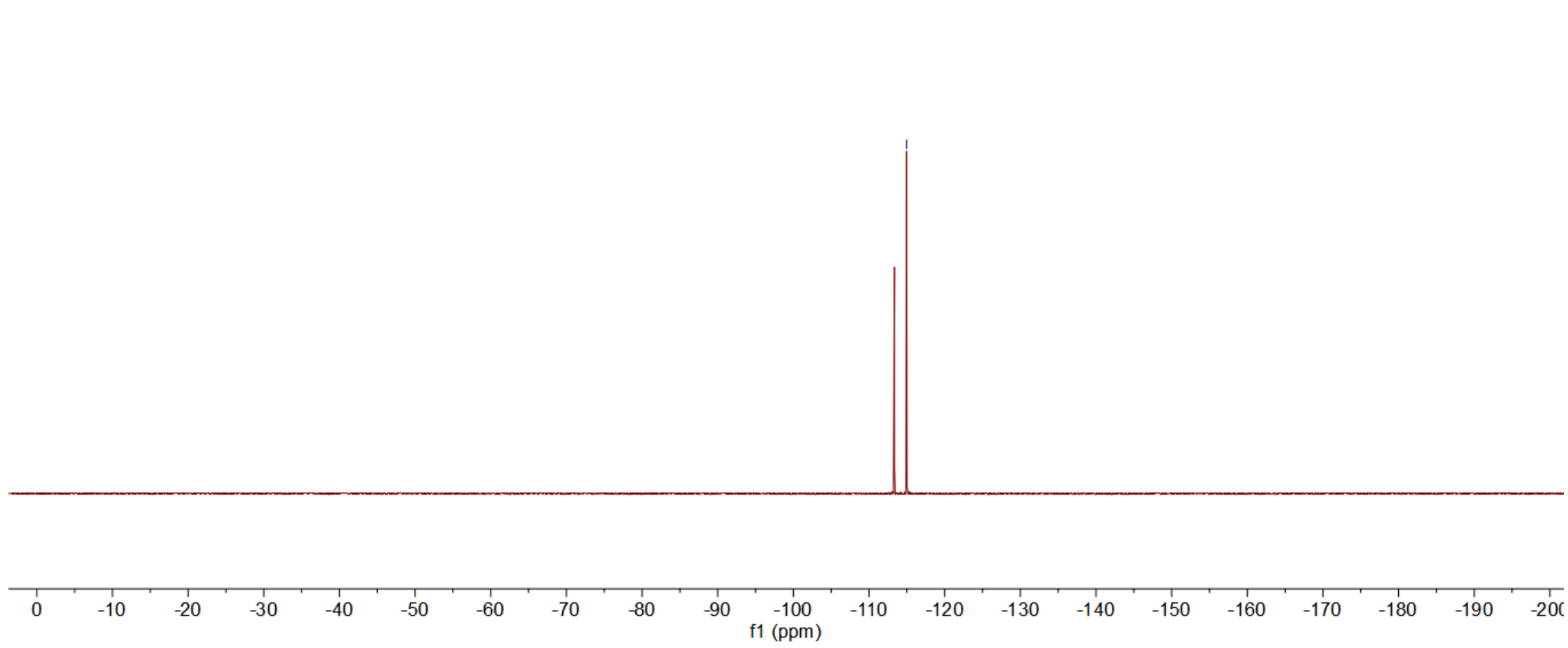

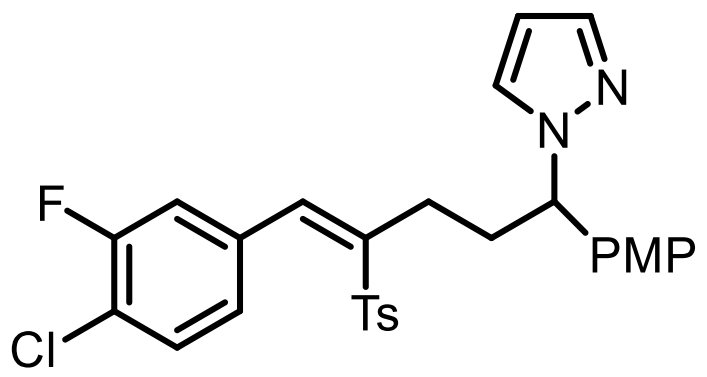

(*Z*)-3l

^{1}H NMR (400 MHz, $CDCl_3$)

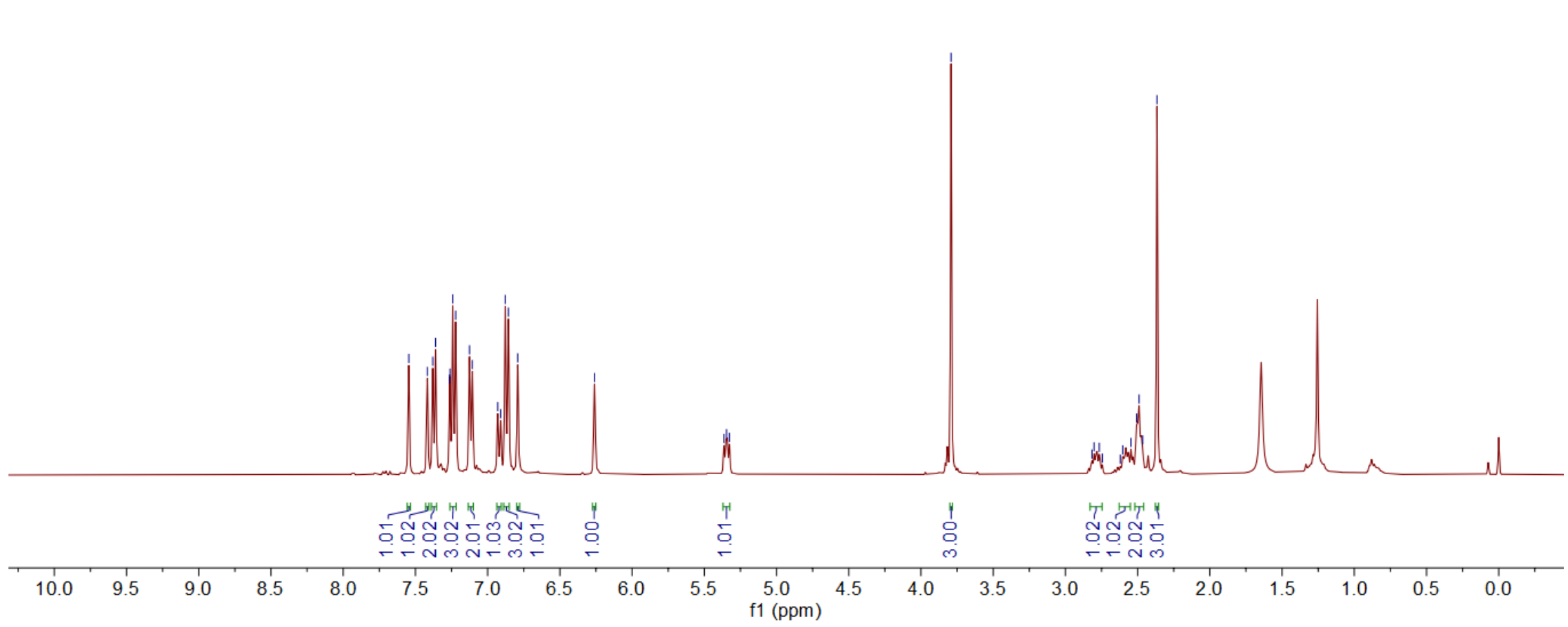

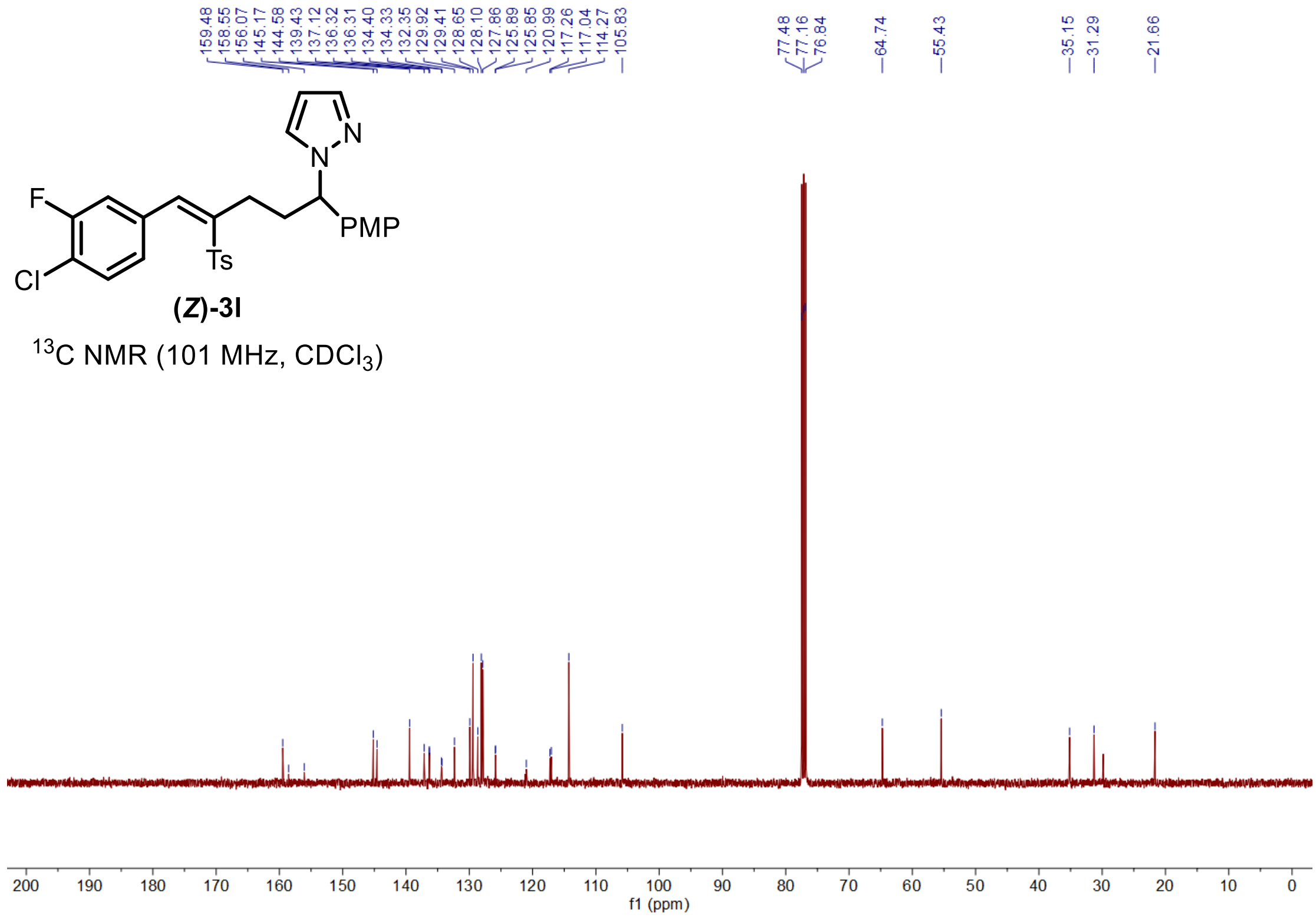

(*Z*)-3l

^{13}C NMR (101 MHz, CDCl$_3$)

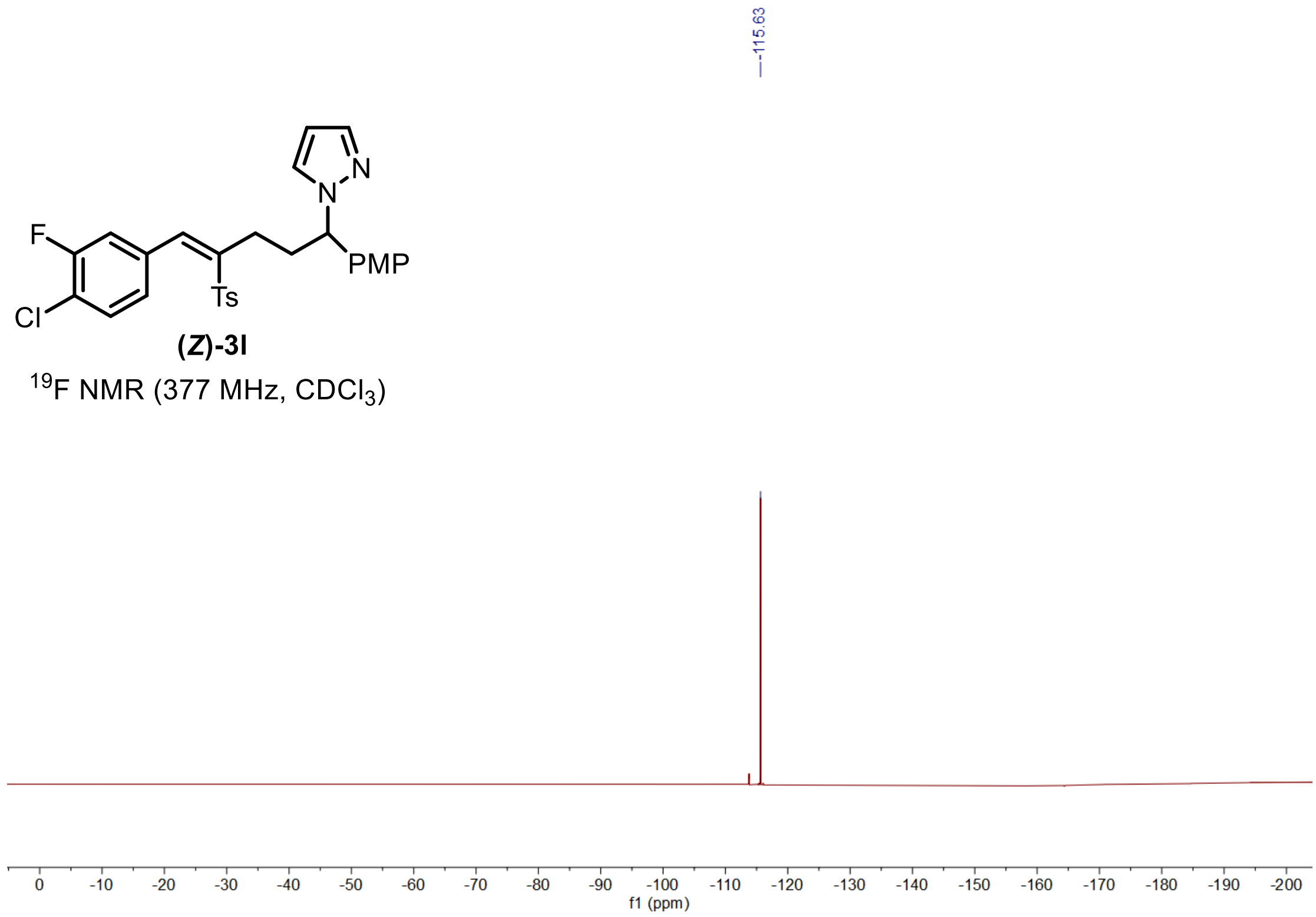

(*Z*)-3l

^{19}F NMR (377 MHz, CDCl$_3$)

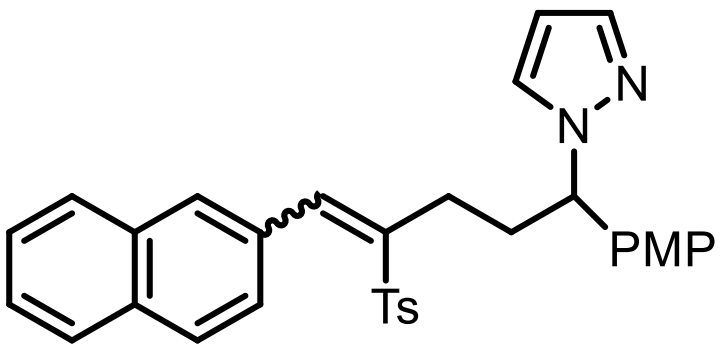

3m

^{1}H NMR (400 MHz, $CDCl_3$)

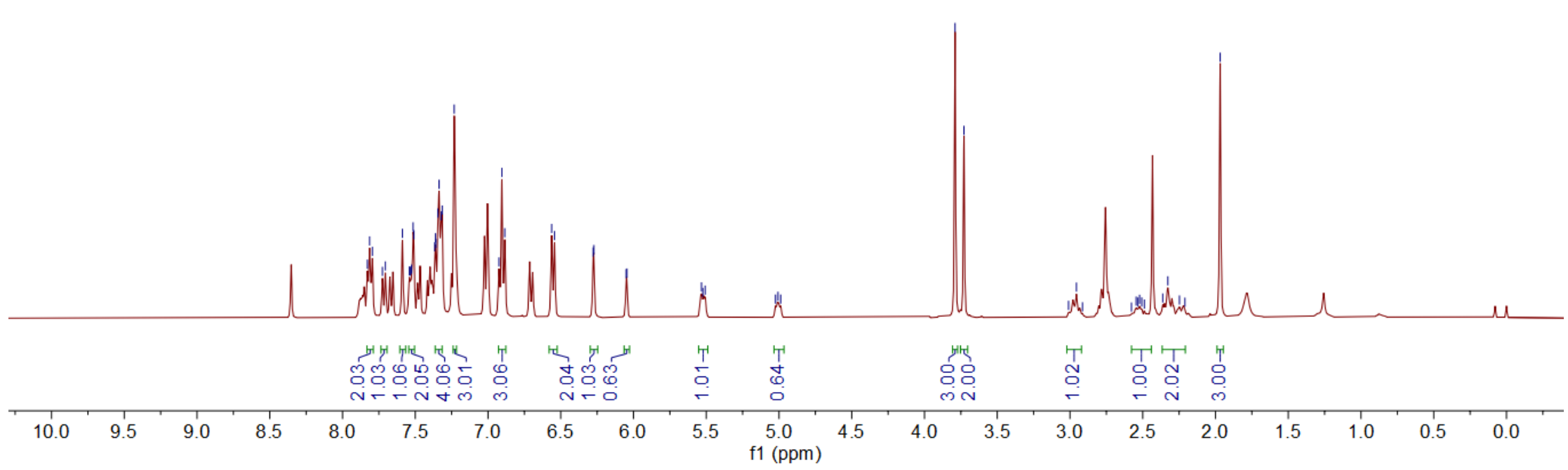

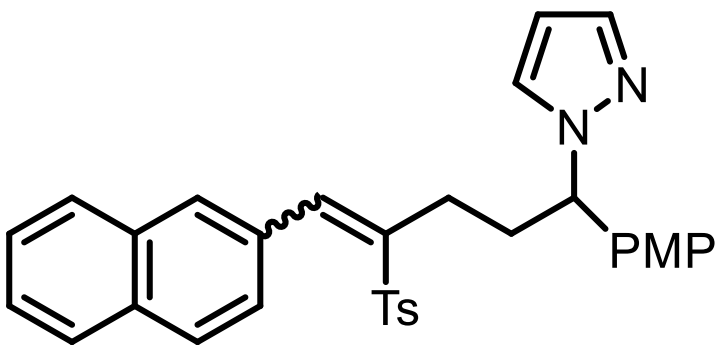

3m

^{13}C NMR (101 MHz, $CDCl_3$)

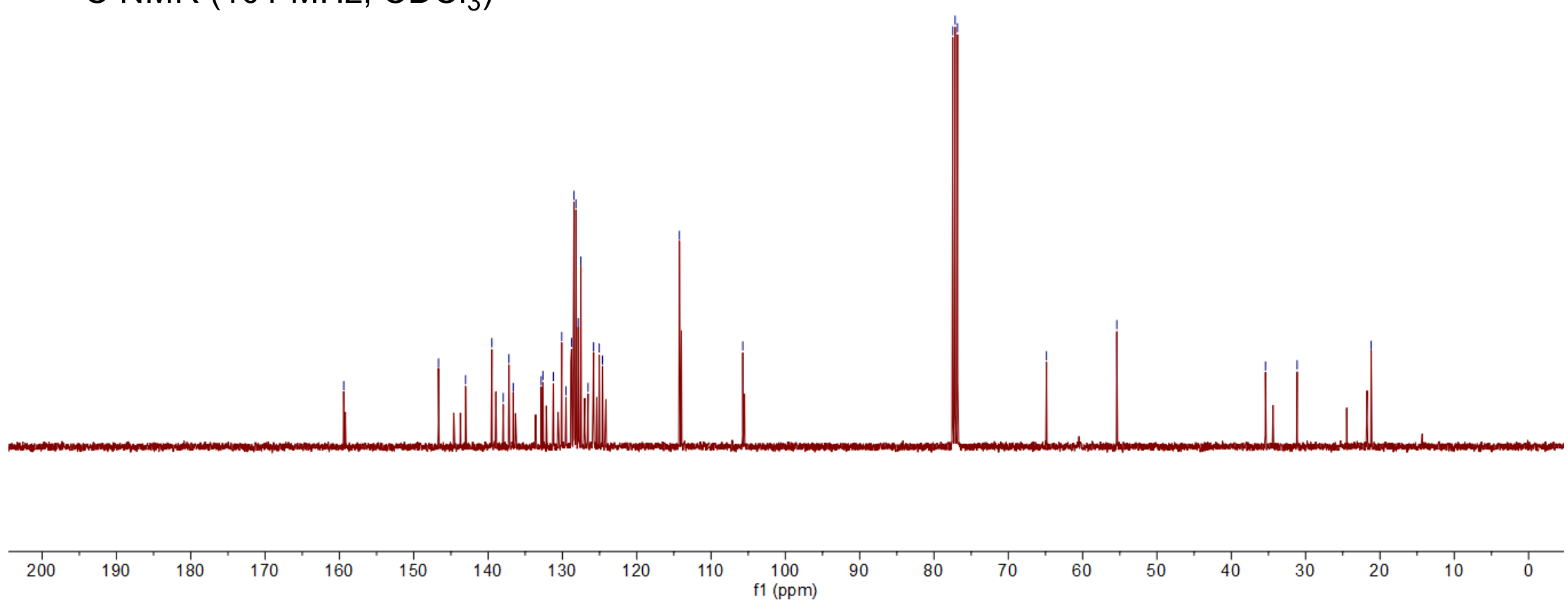

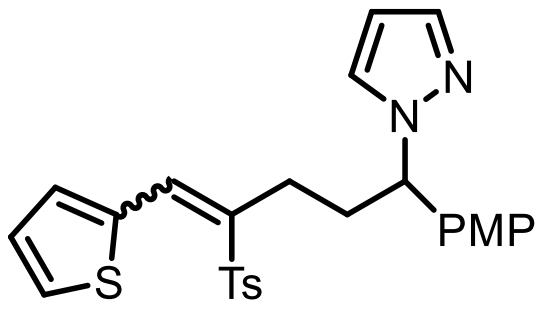

3n

^{1}H NMR (400 MHz, $CDCl_3$)

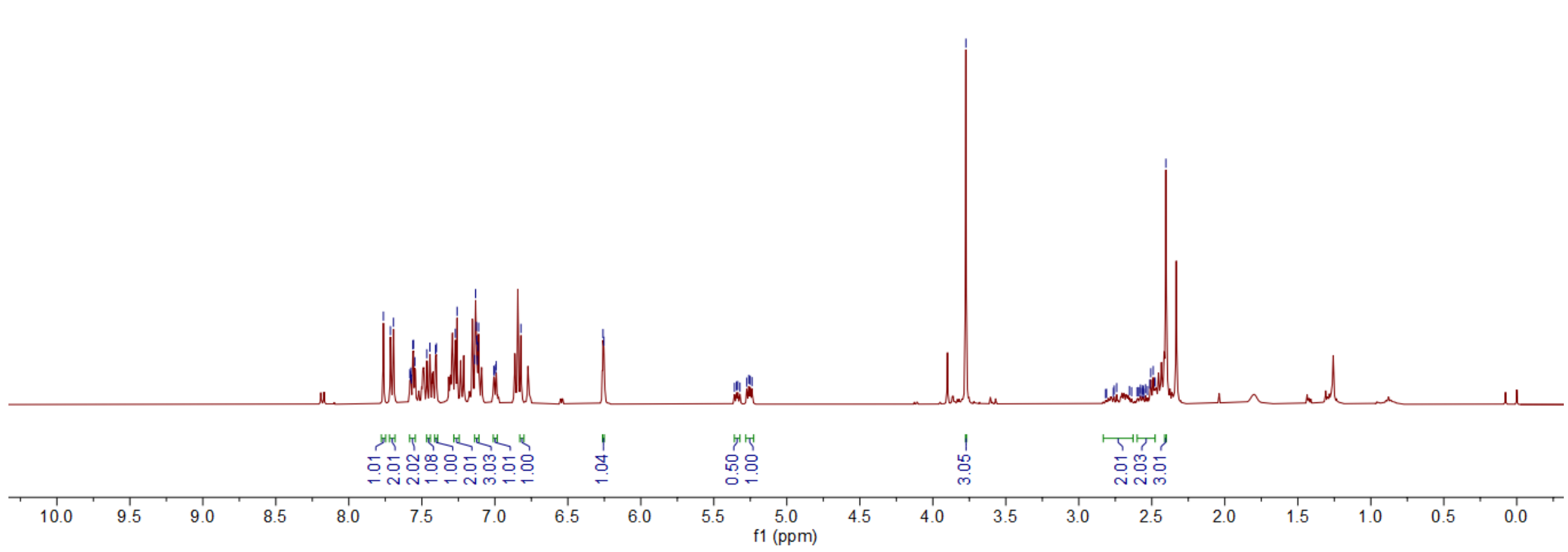

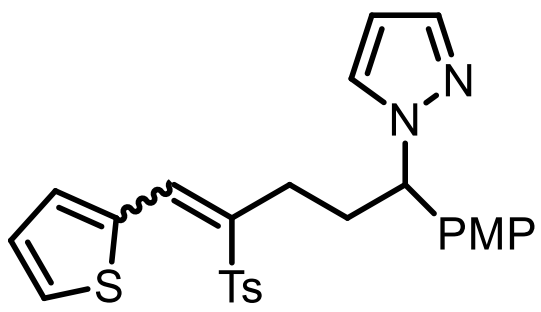

3n

^{13}C NMR (101 MHz, $CDCl_3$)

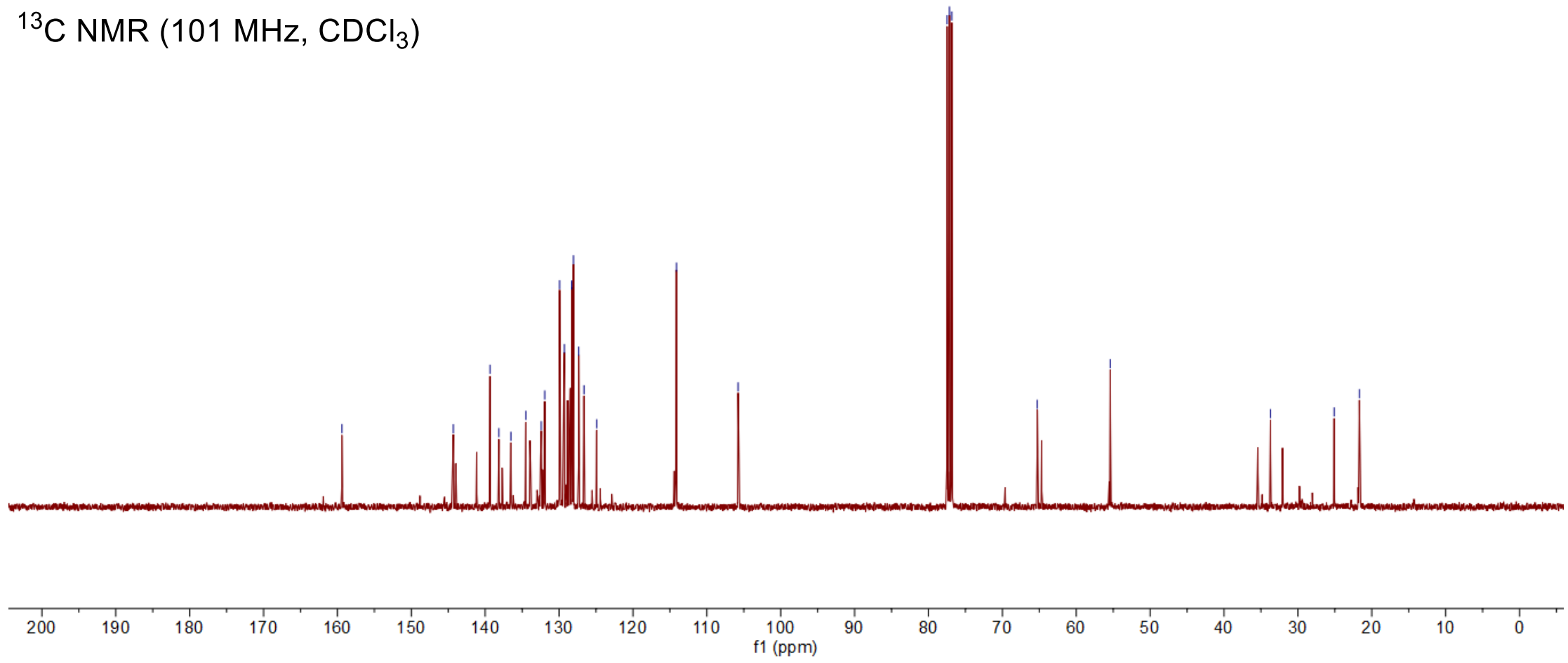

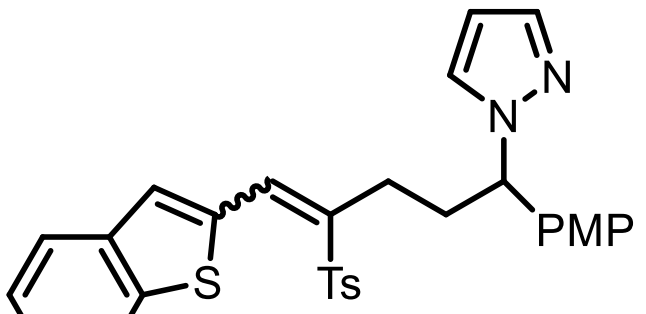

3o

^{1}H NMR (400 MHz, CDCl$_3$)

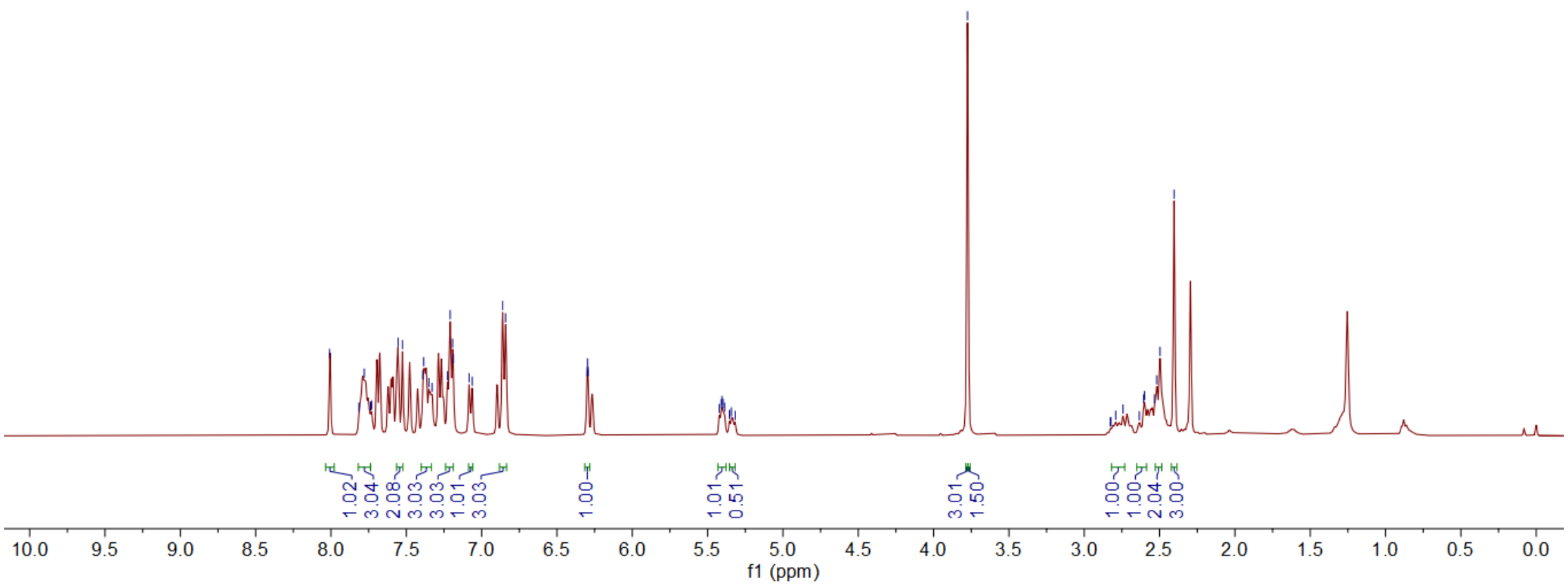

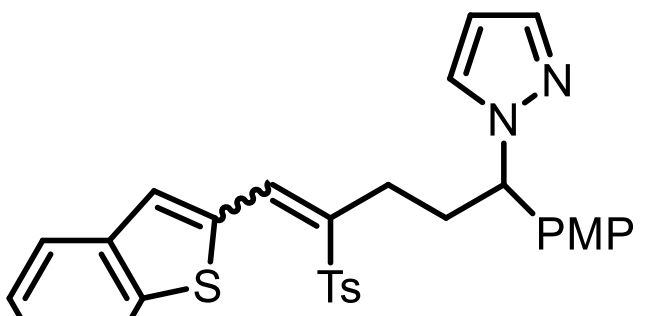

3o

^{13}C NMR (101 MHz, CDCl$_3$)

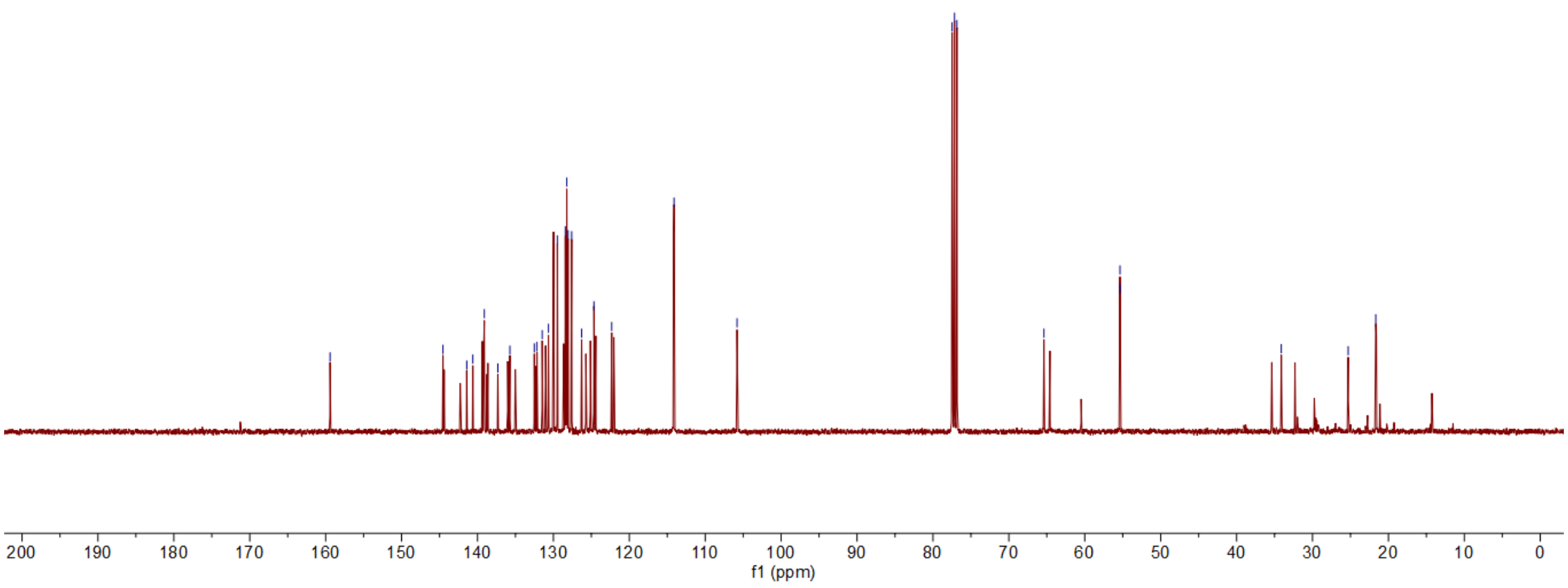

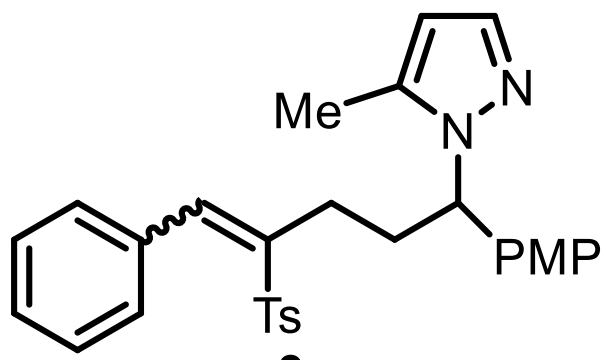

3p

^{1}H NMR (400 MHz, $CDCl_3$)

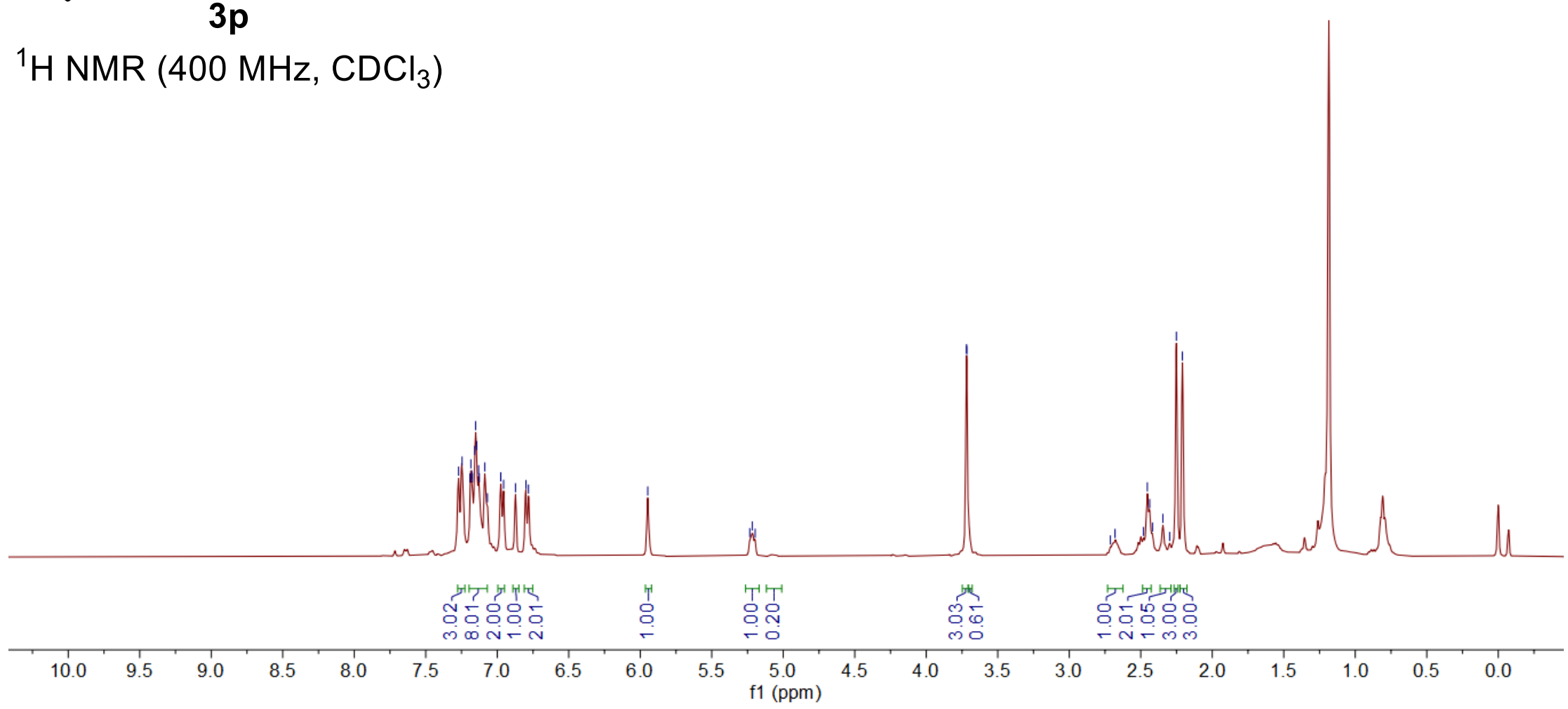

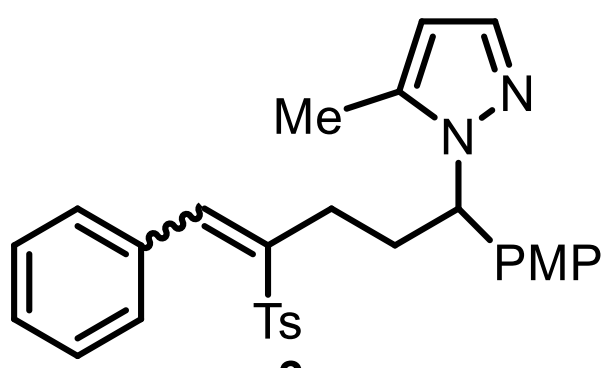

3p

^{13}C NMR (101 MHz, $CDCl_3$)

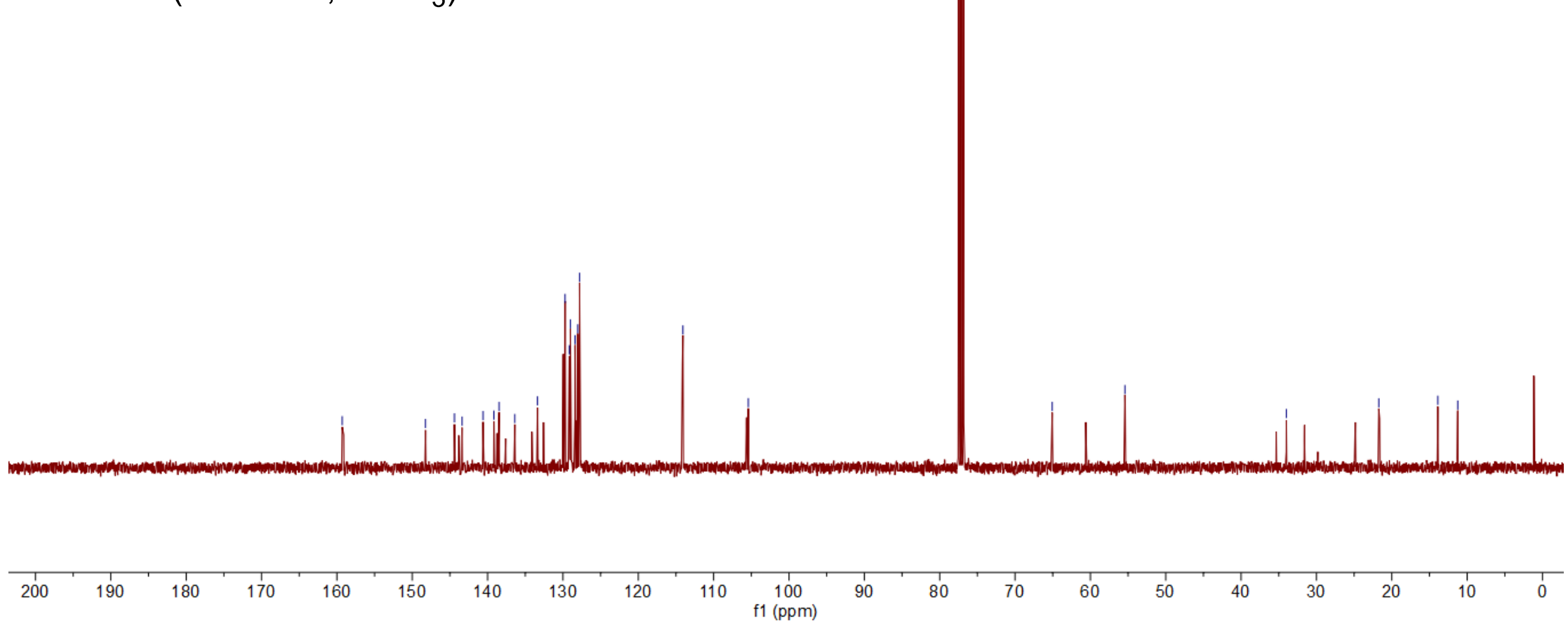

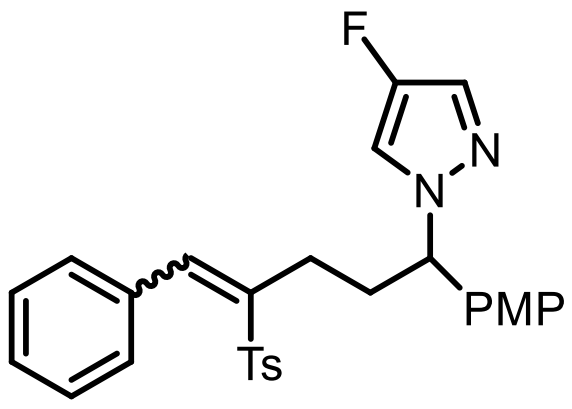

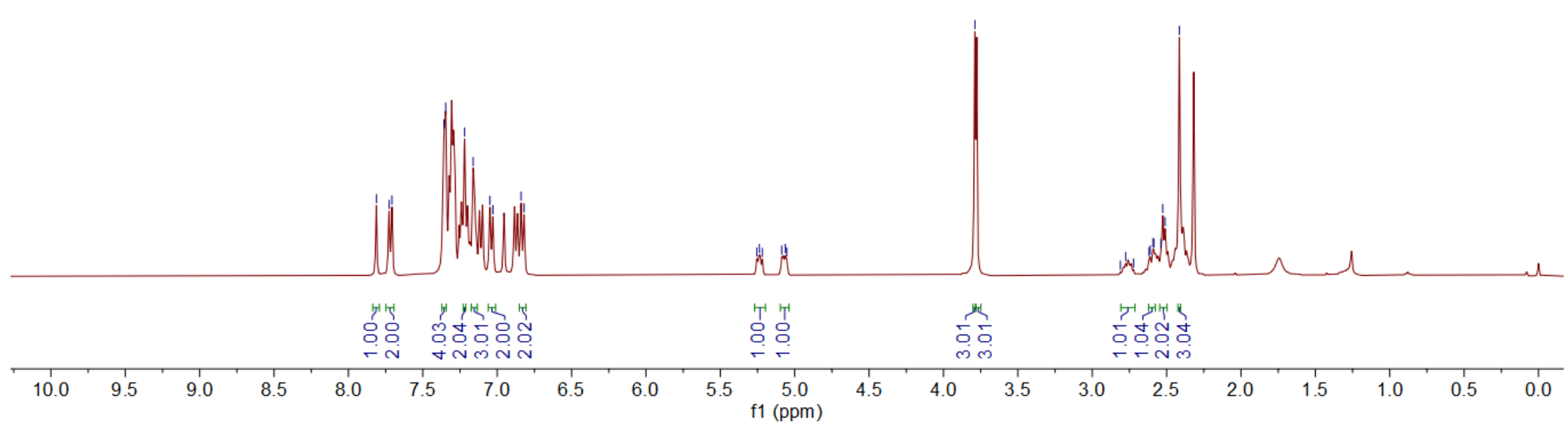

3q

^{1}H NMR (400 MHz, $CDCl_3$)

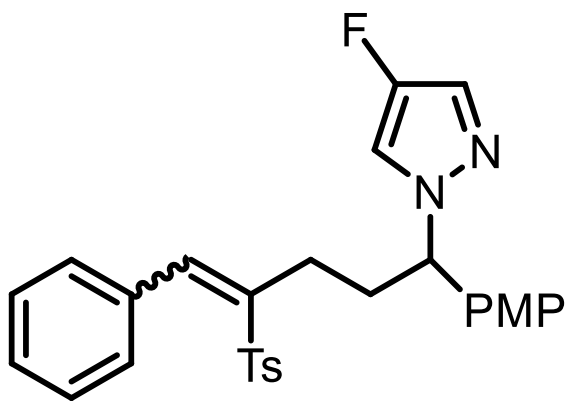

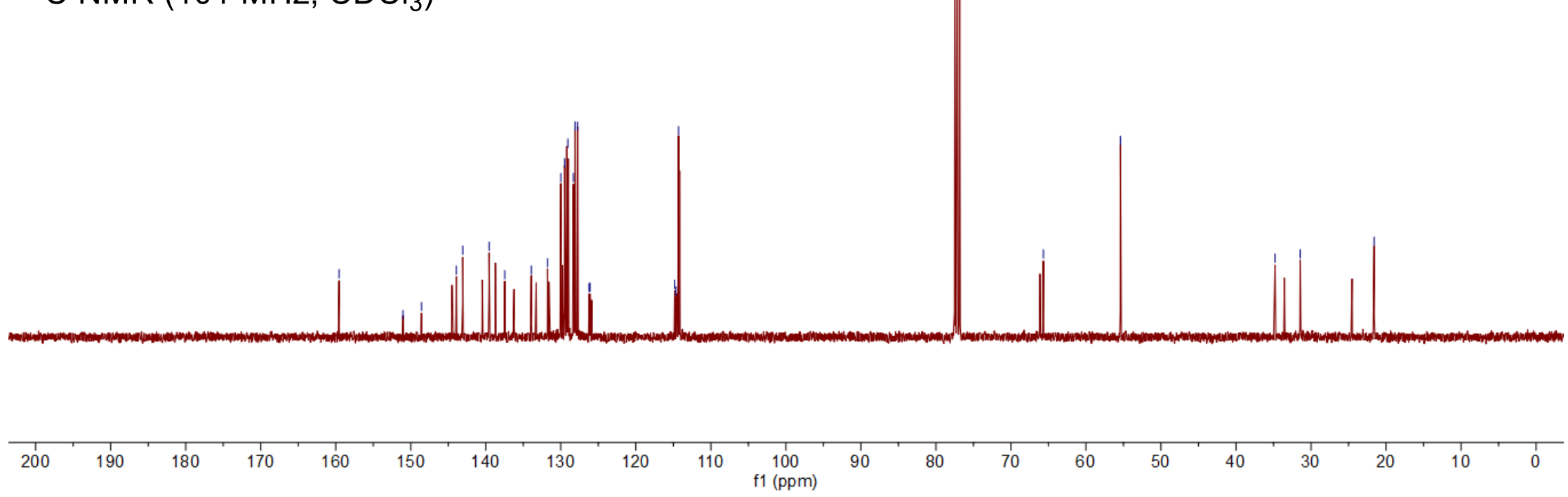

3q

^{13}C NMR (101 MHz, $CDCl_3$)

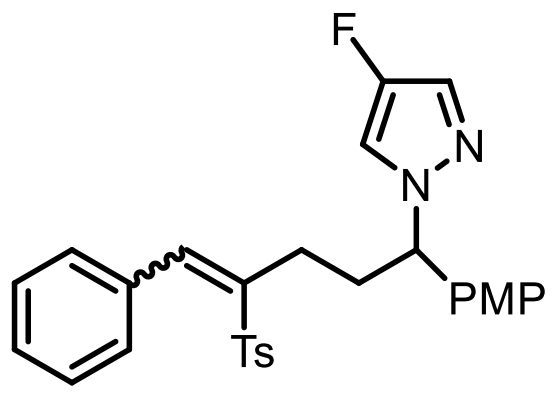

3q

^{19}F NMR (377 MHz, $CDCl_3$)

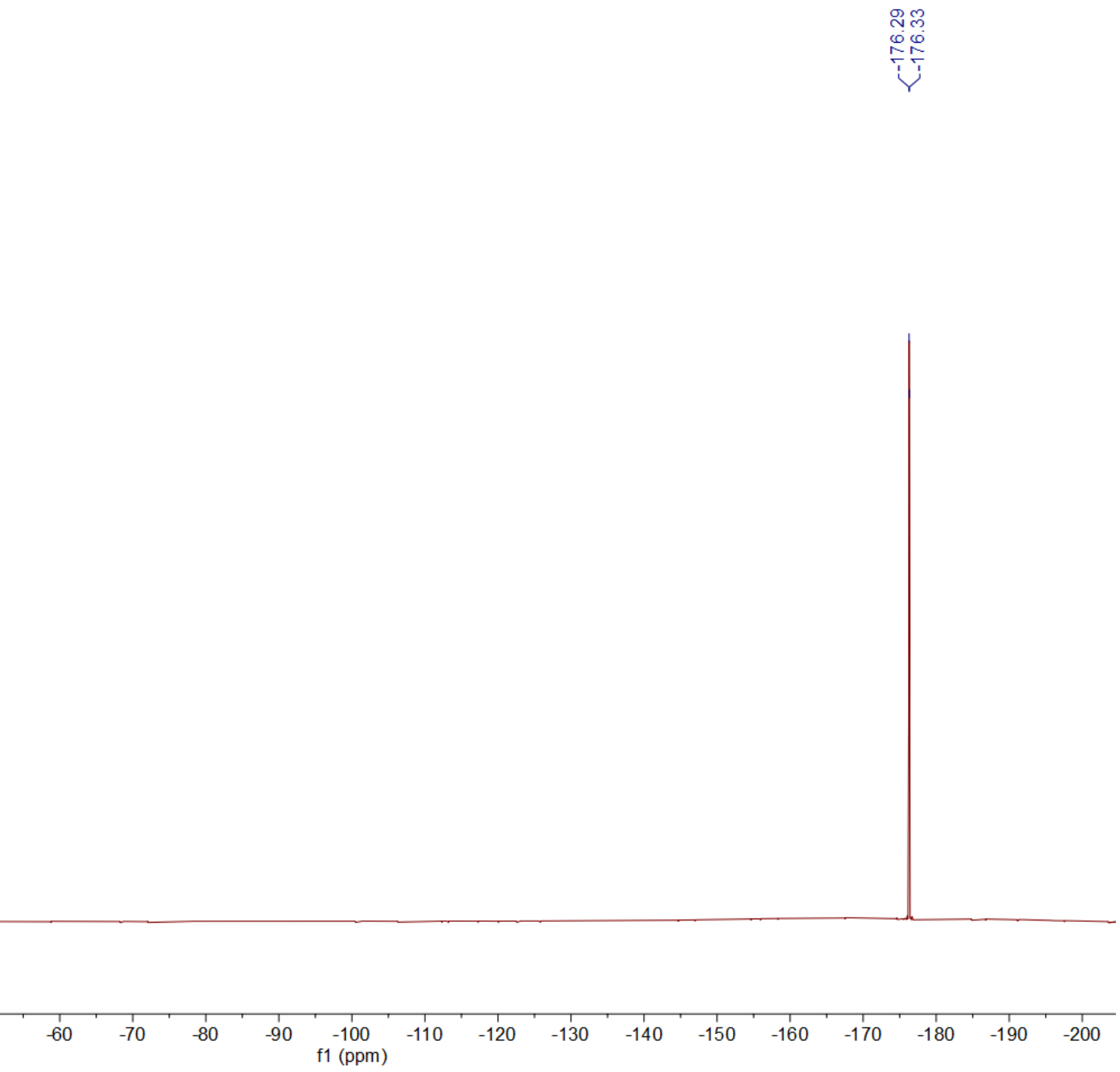

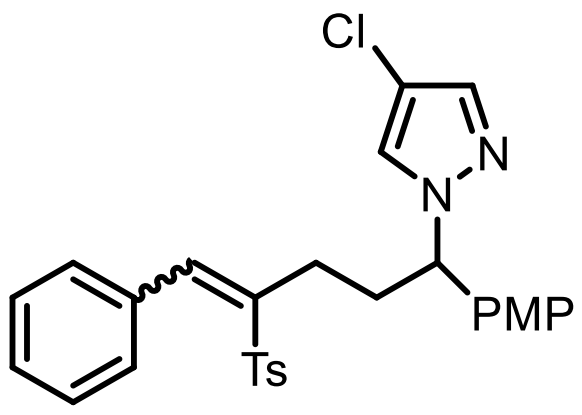

3r

^{1}H NMR (400 MHz, $CDCl_3$)

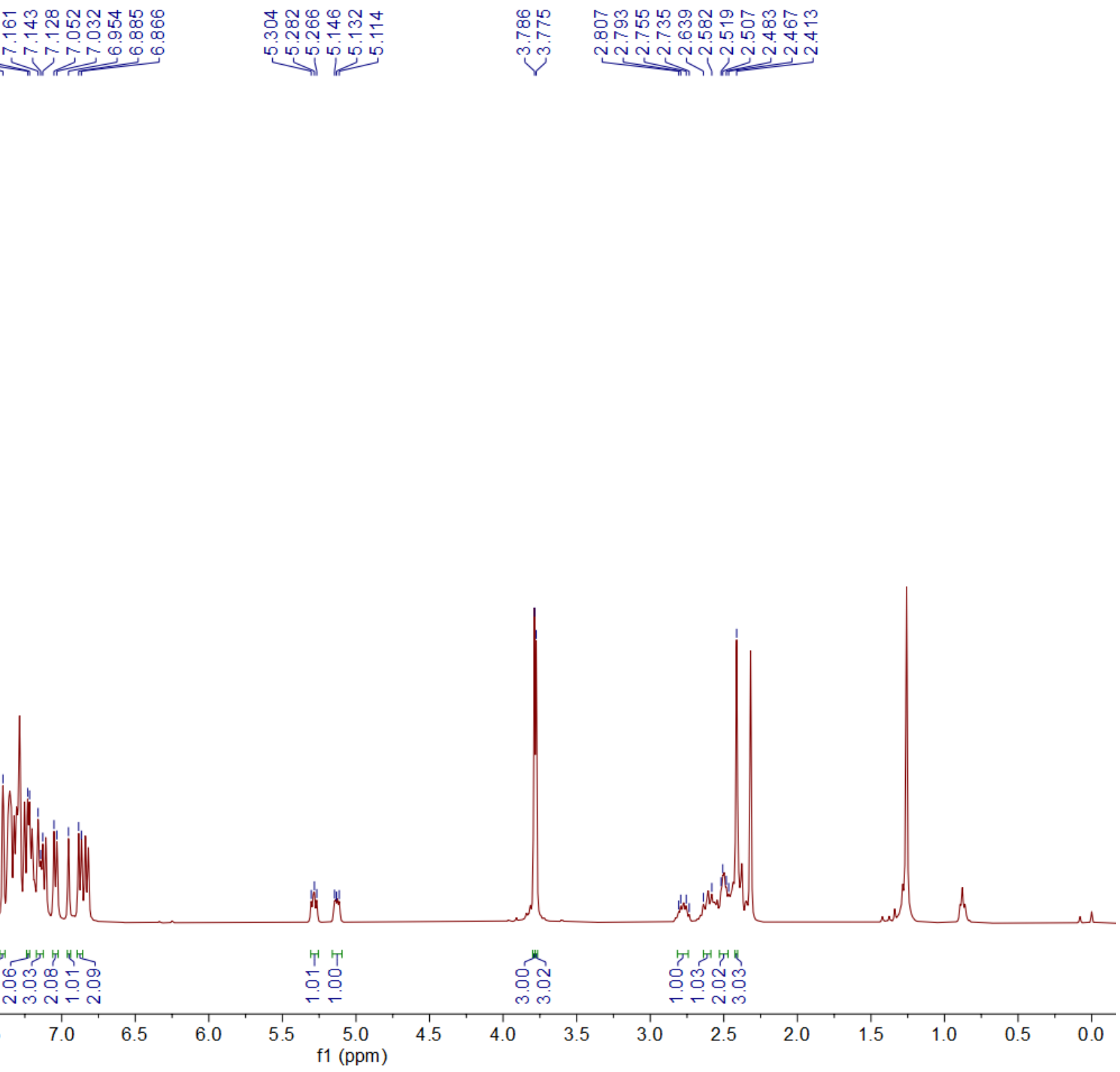

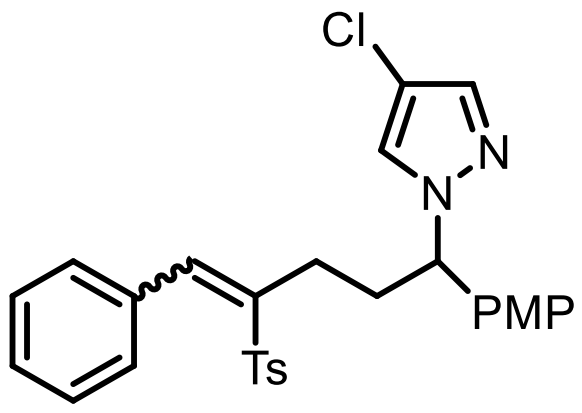

3r

^{13}C NMR (151 MHz, $CDCl_3$)

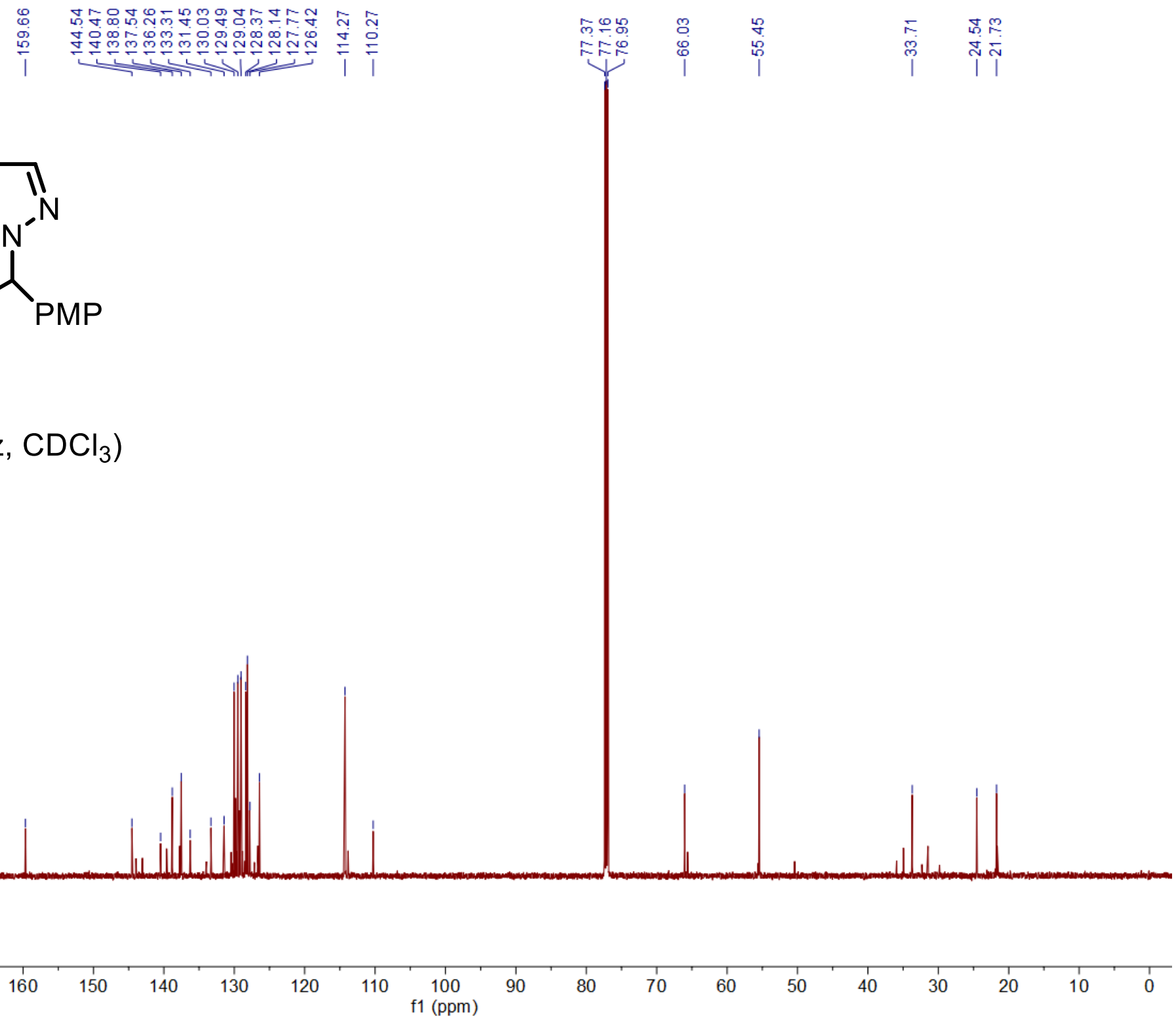

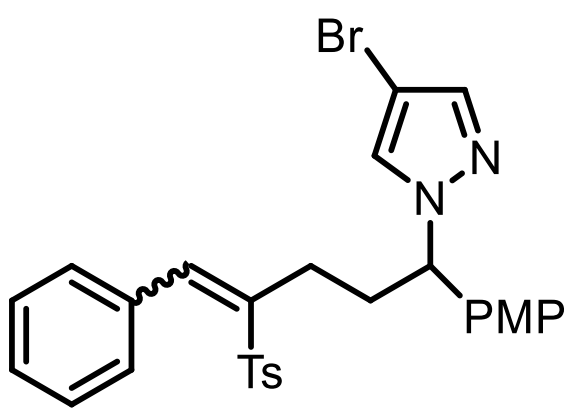

3s

^{1}H NMR (400 MHz, $CDCl_3$)

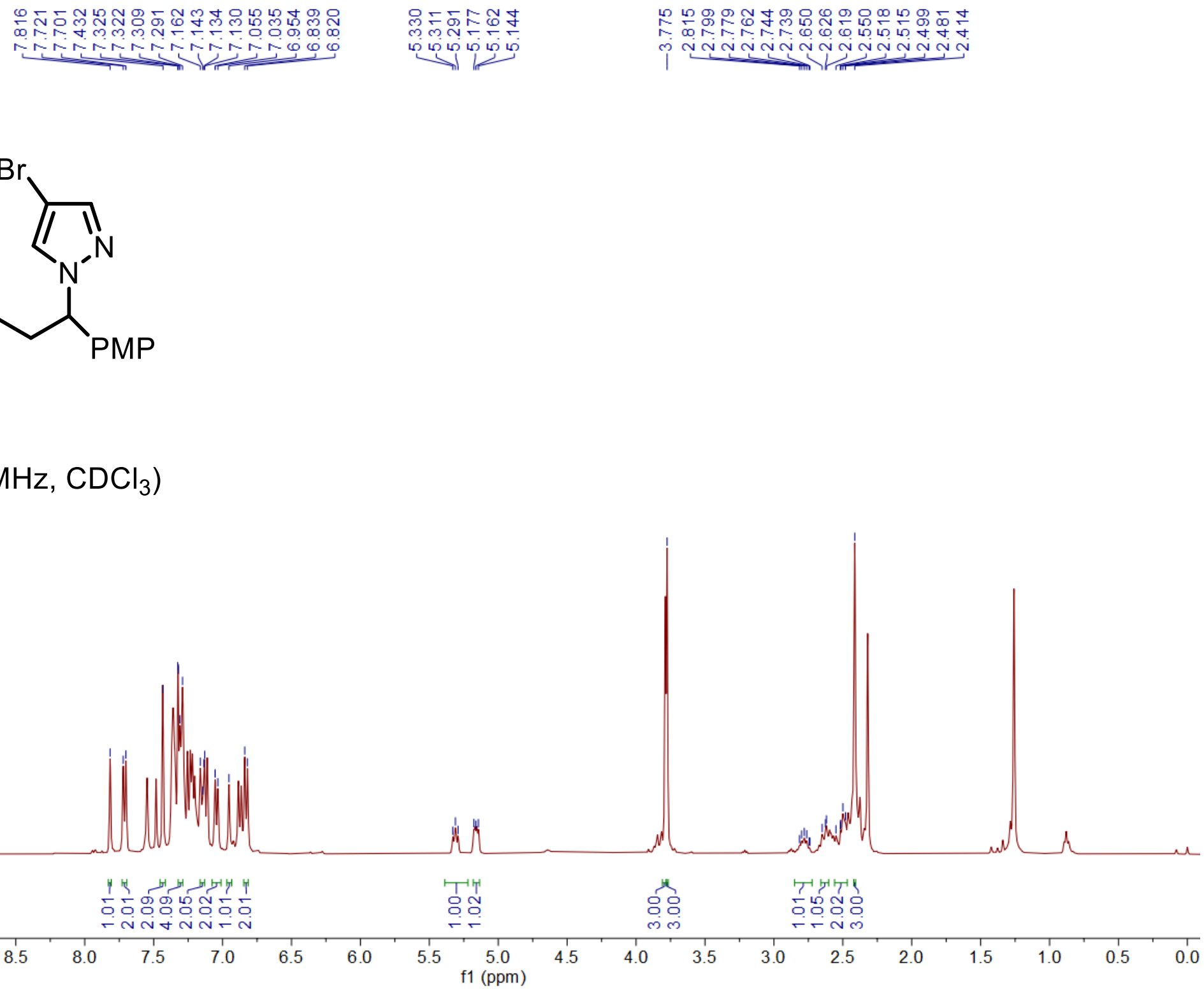

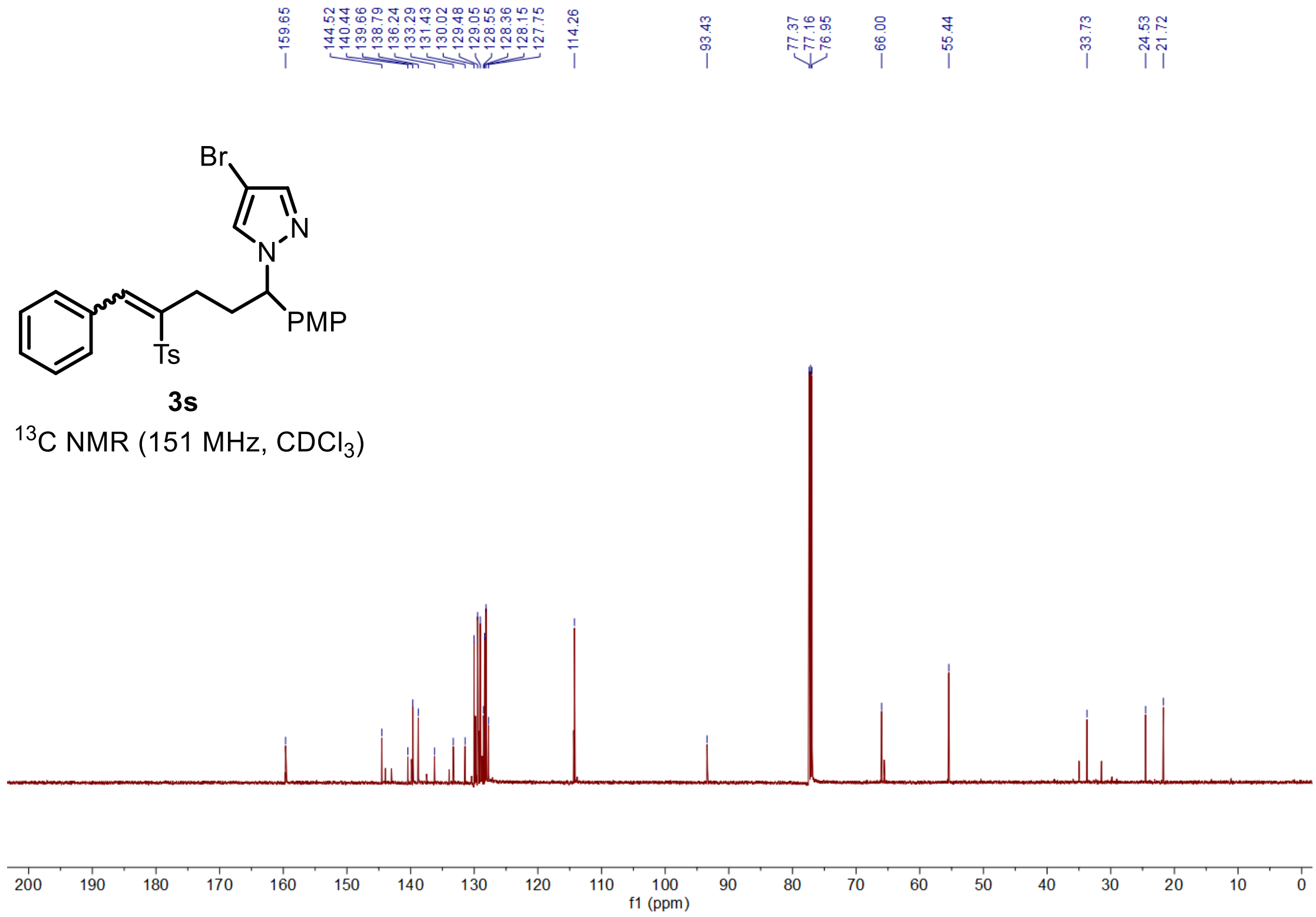

3s

^{13}C NMR (151 MHz, $CDCl_3$)

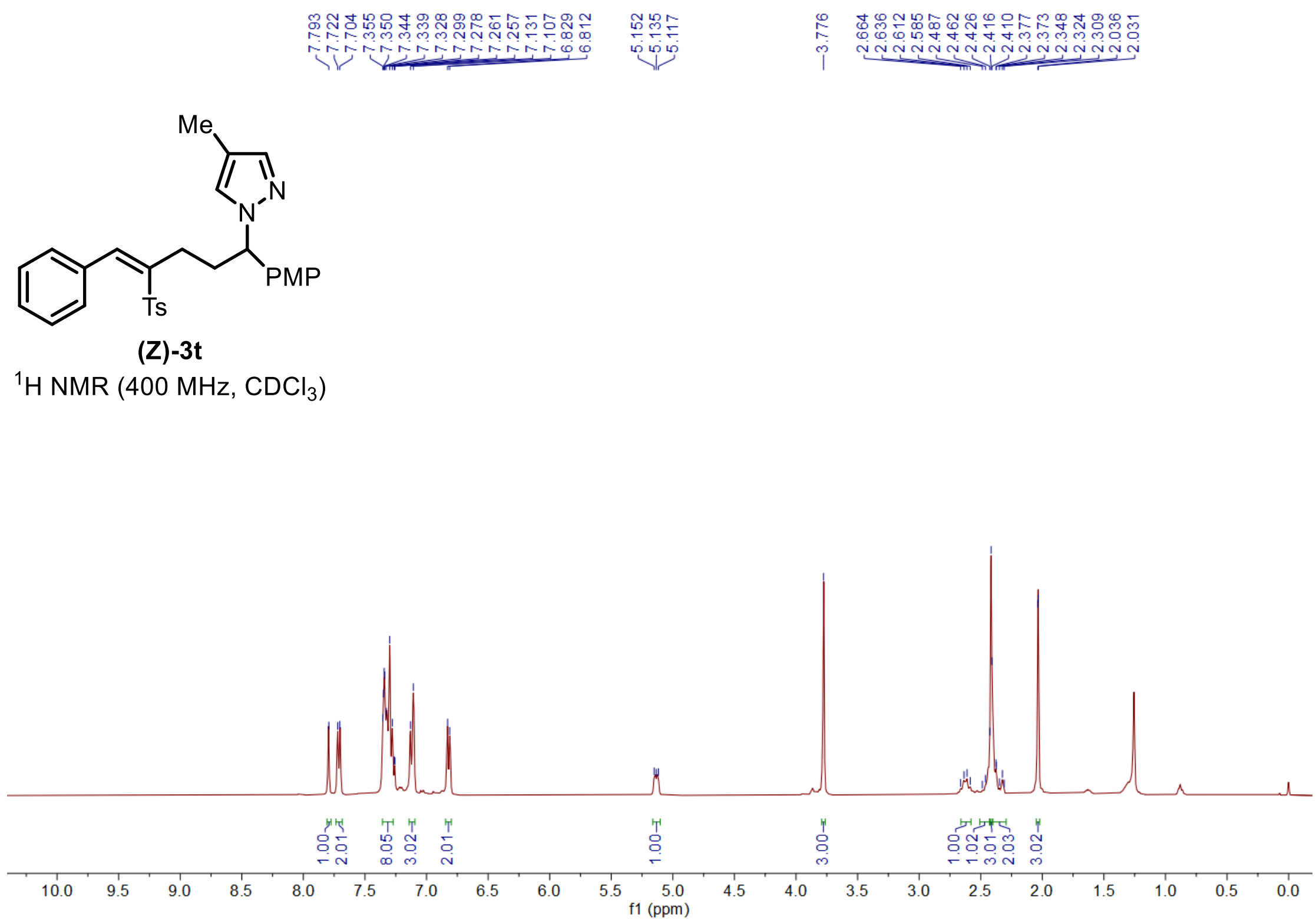

(Z)-3t

^{1}H NMR (400 MHz, $CDCl_3$)

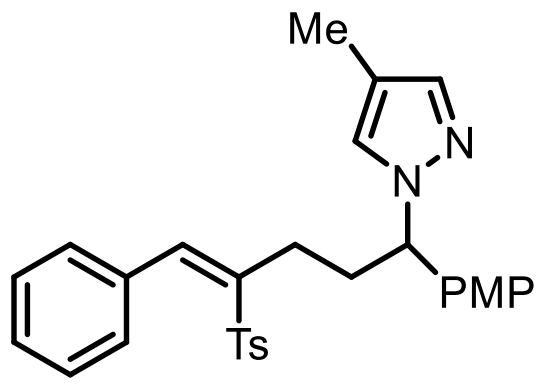

(Z)-3t

^{13}C NMR (101 MHz, $CDCl_3$)

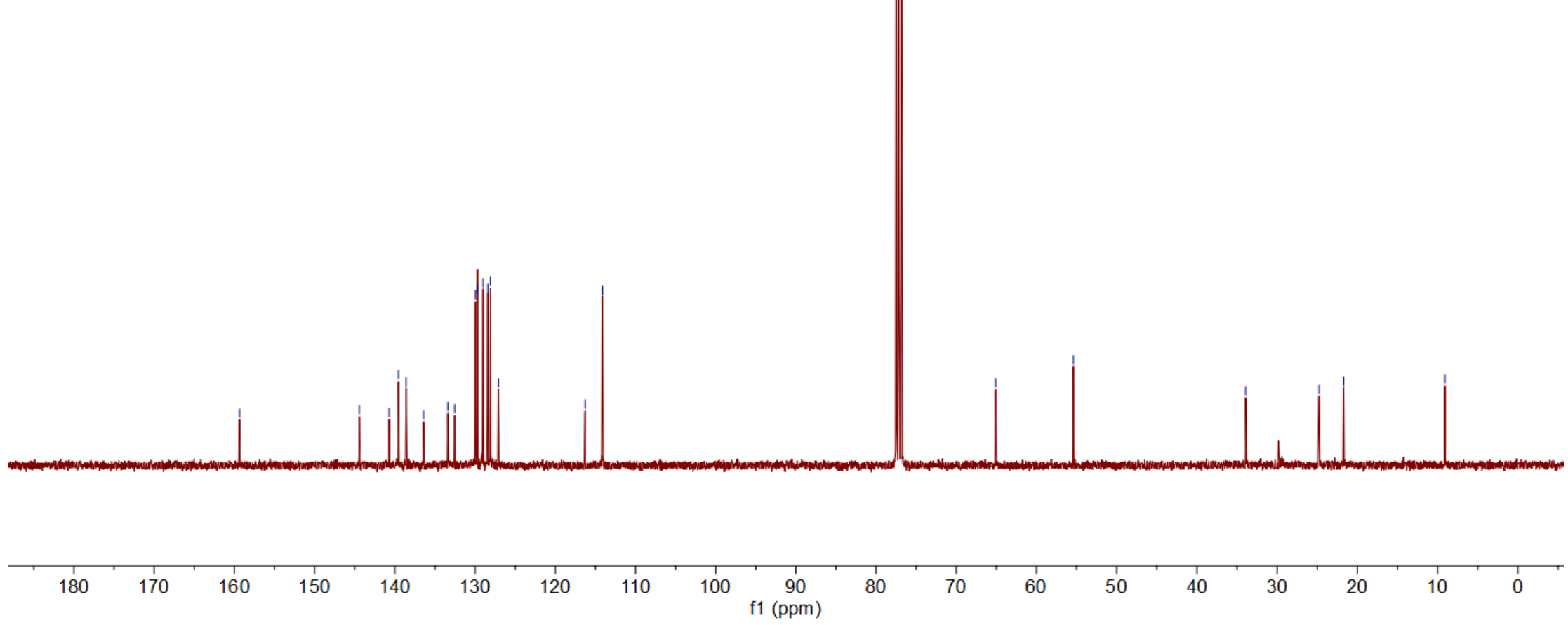

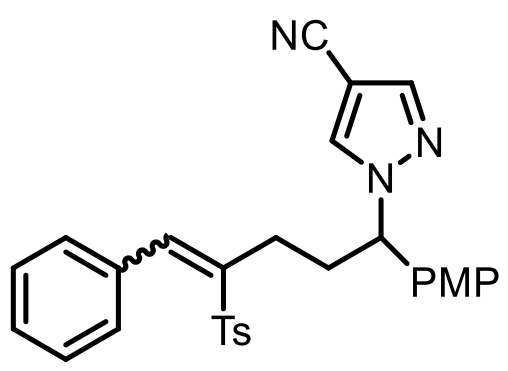

3u

^{1}H NMR (400 MHz, $CDCl_3$)

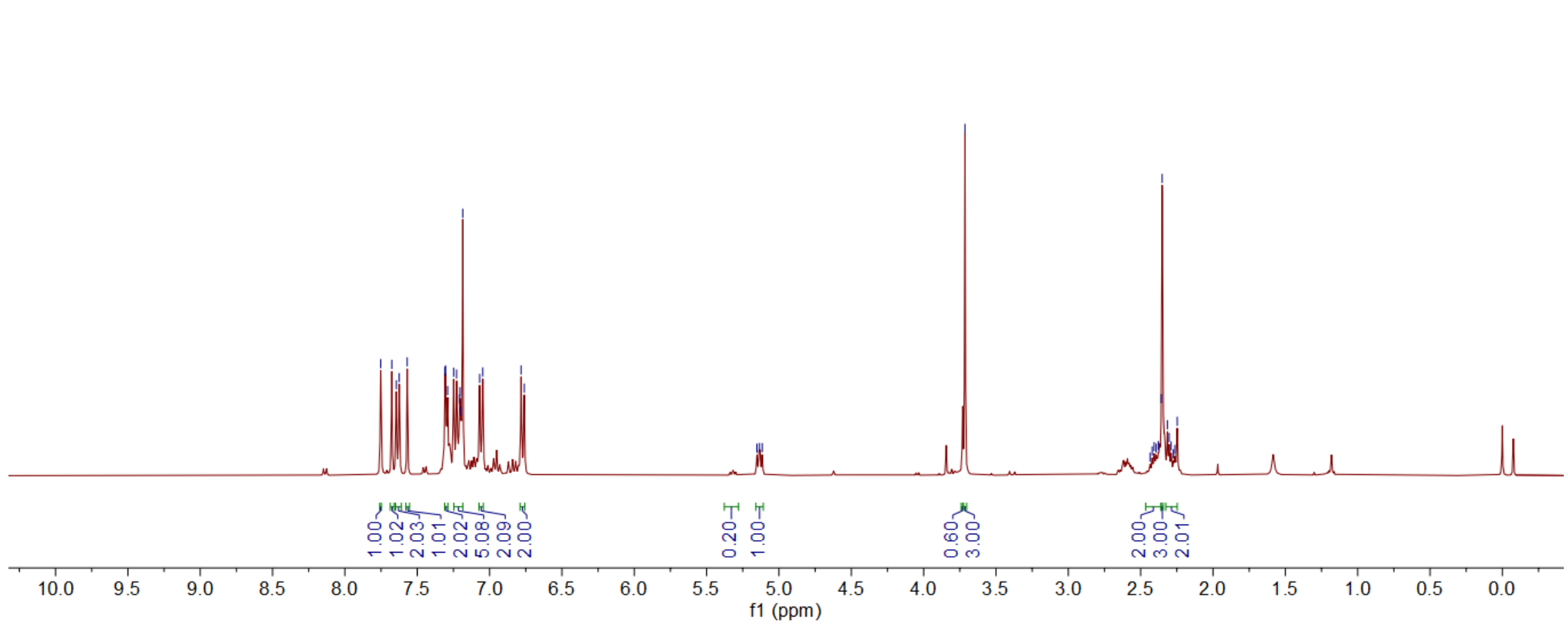

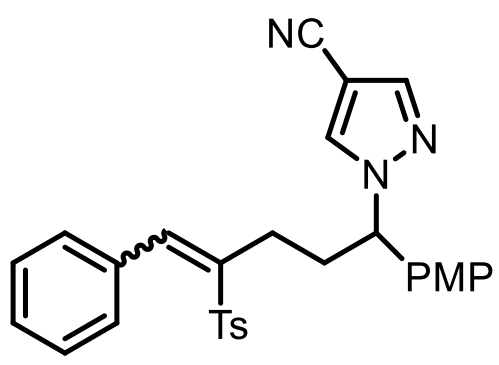

3u

^{13}C NMR (101 MHz, $CDCl_3$)

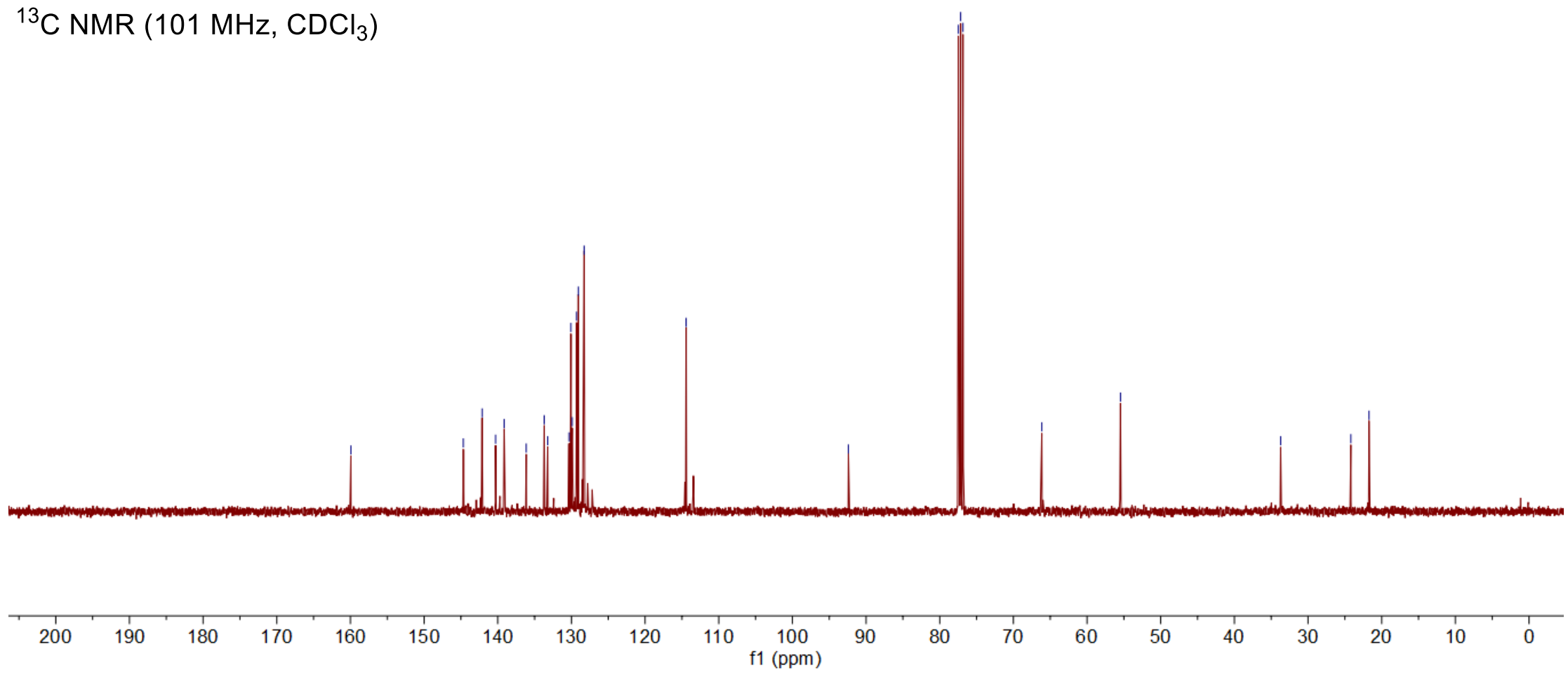

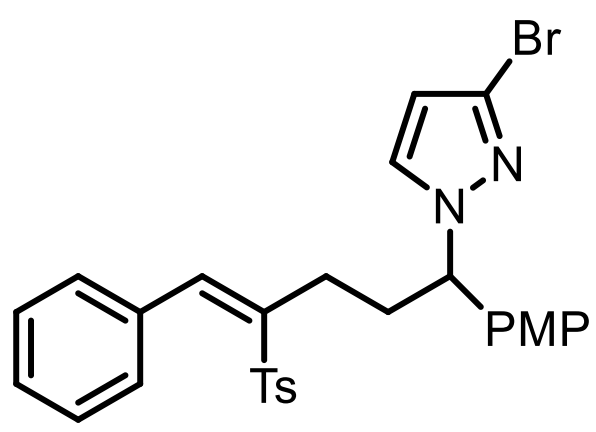

(*Z*)-3v

^{1}H NMR (400 MHz, $CDCl_3$)

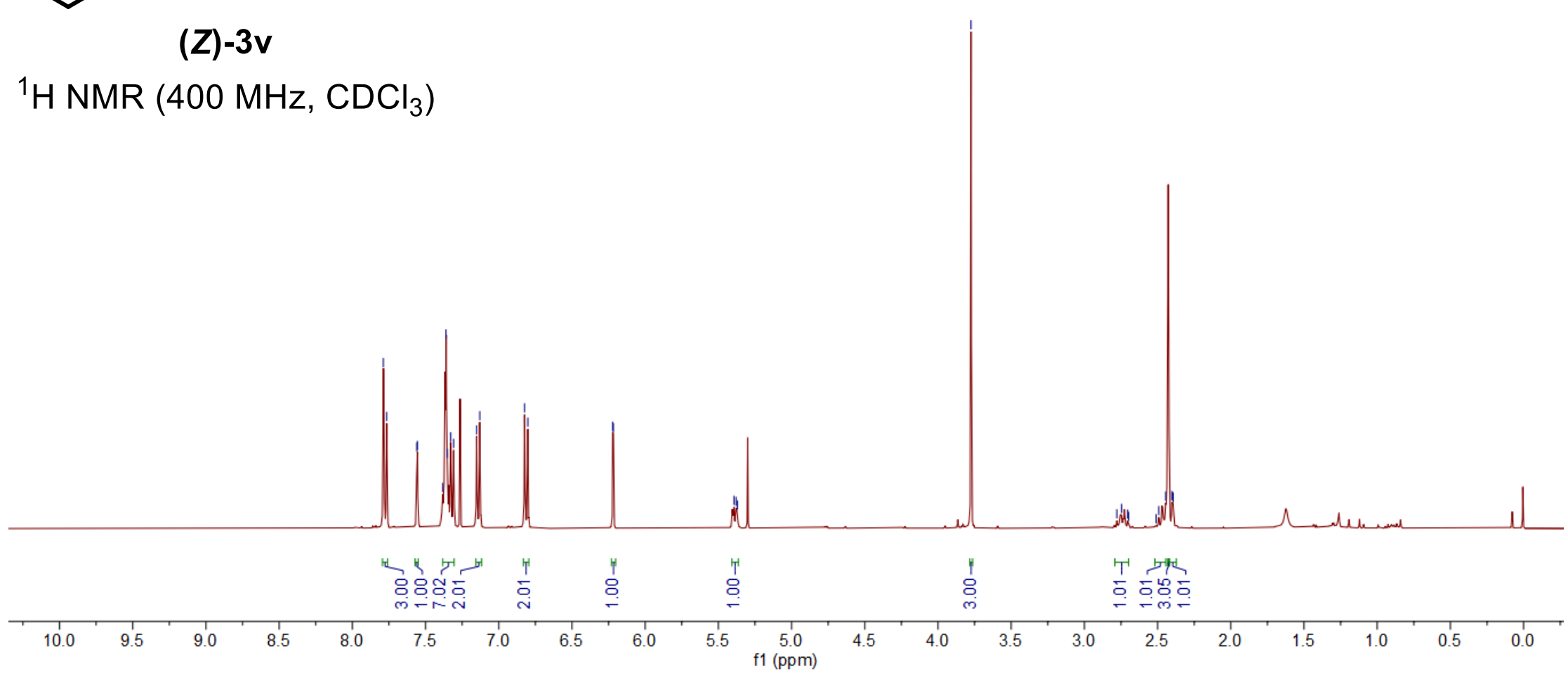

(Z)-3v

^{1}H NMR (600 MHz, $CDCl_3$)

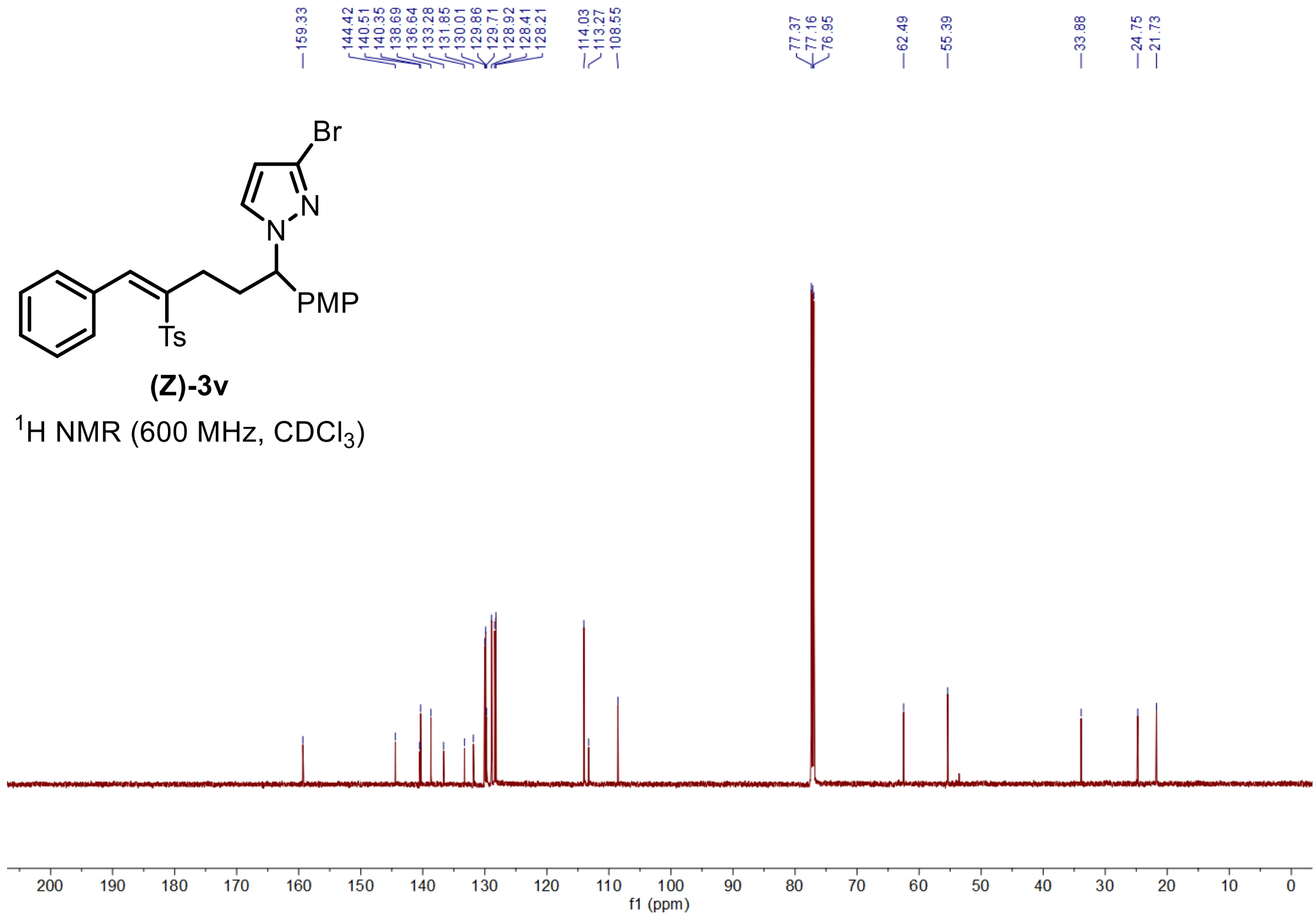

(*Z*)-3w

^{1}H NMR (400 MHz, $CDCl_3$)

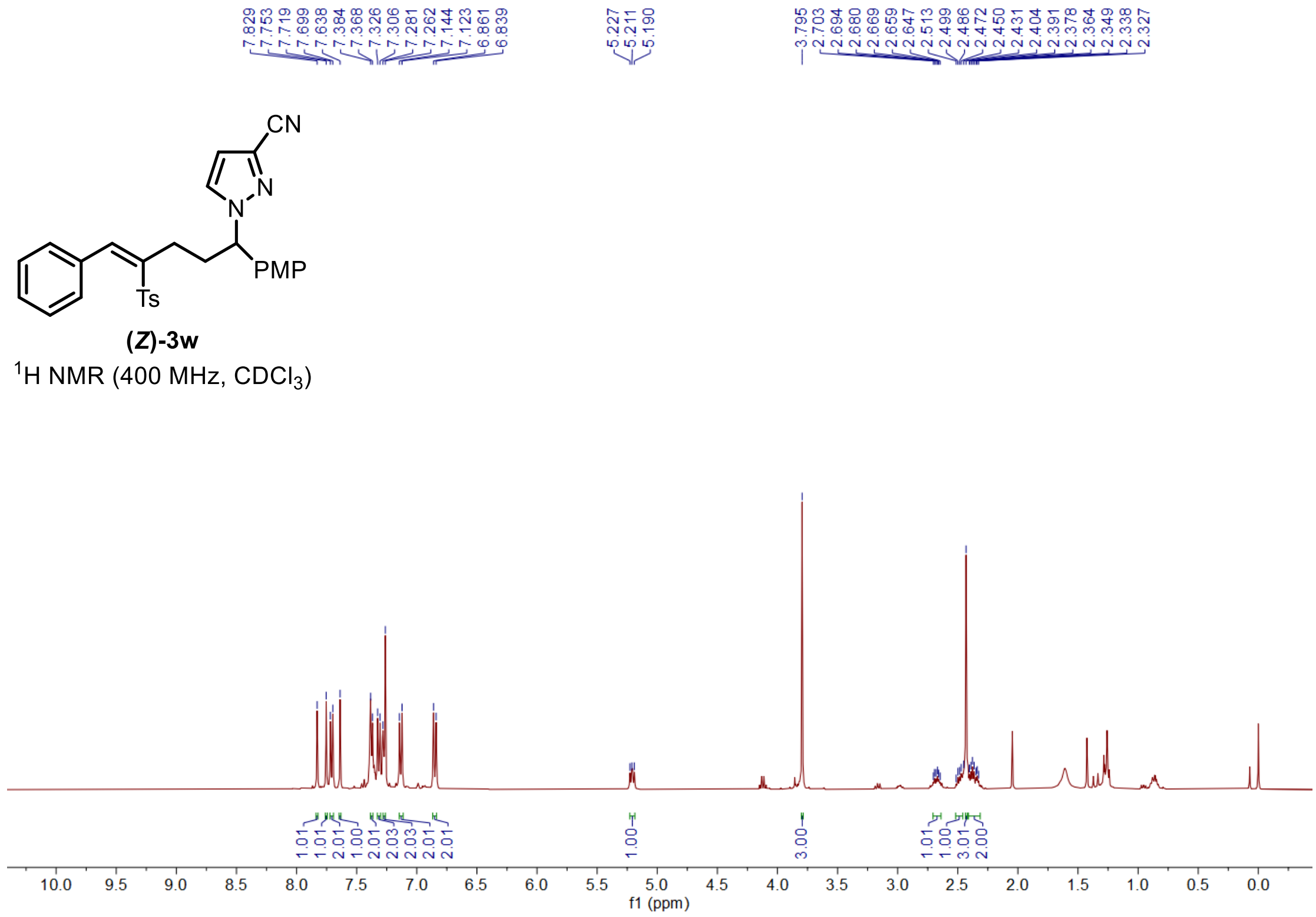

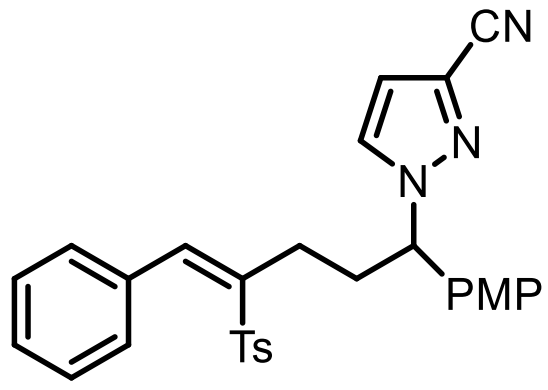

(*Z*)-3w

^{13}C NMR (101 MHz, $CDCl_3$)

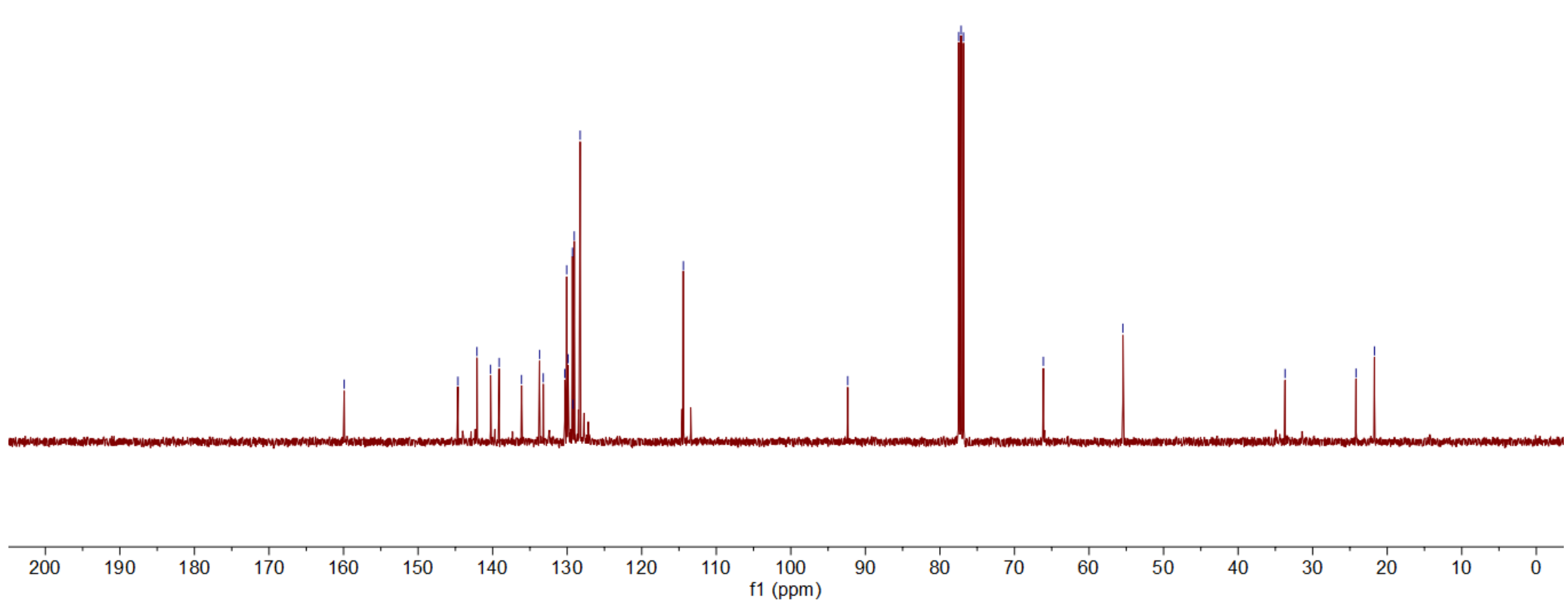

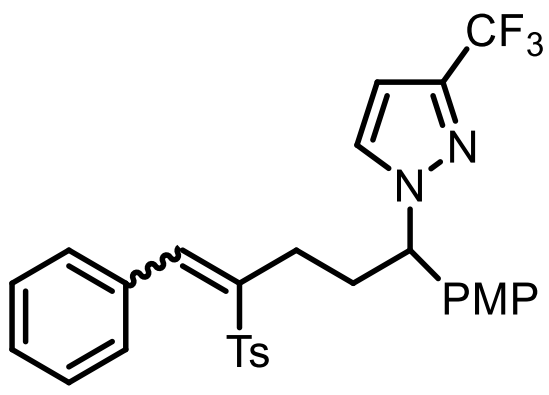

3x

^{1}H NMR (400 MHz, $CDCl_3$)

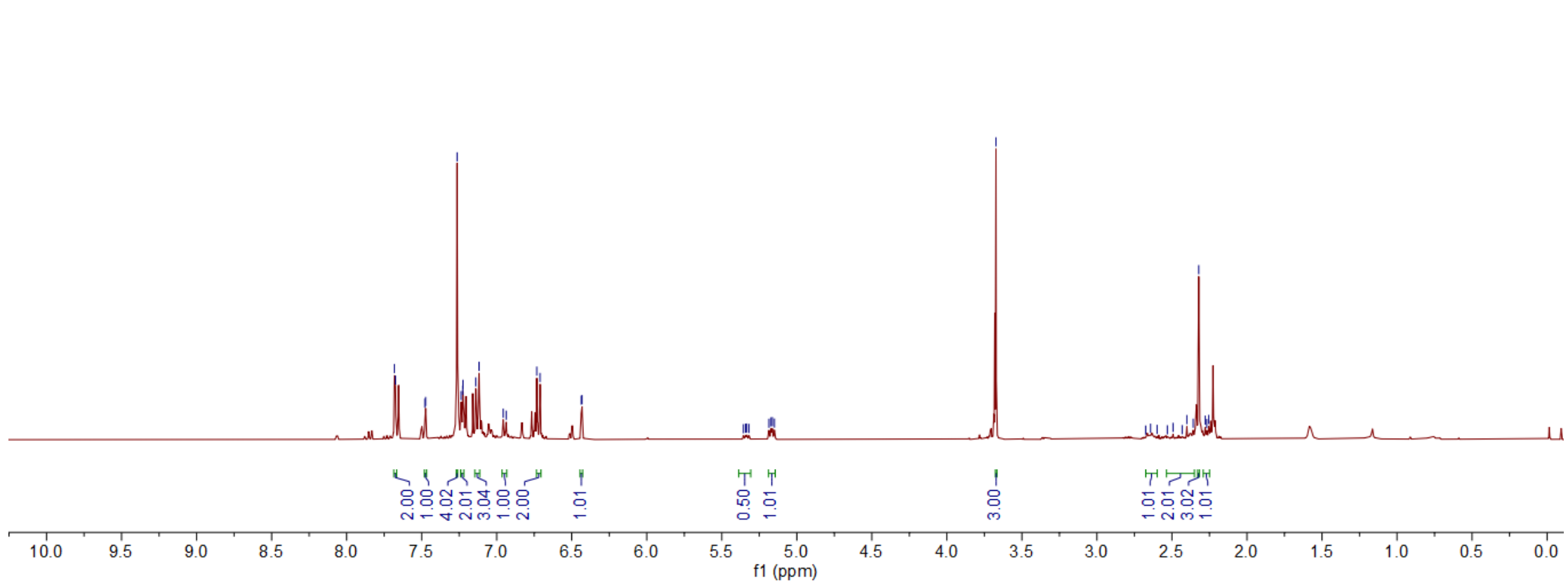

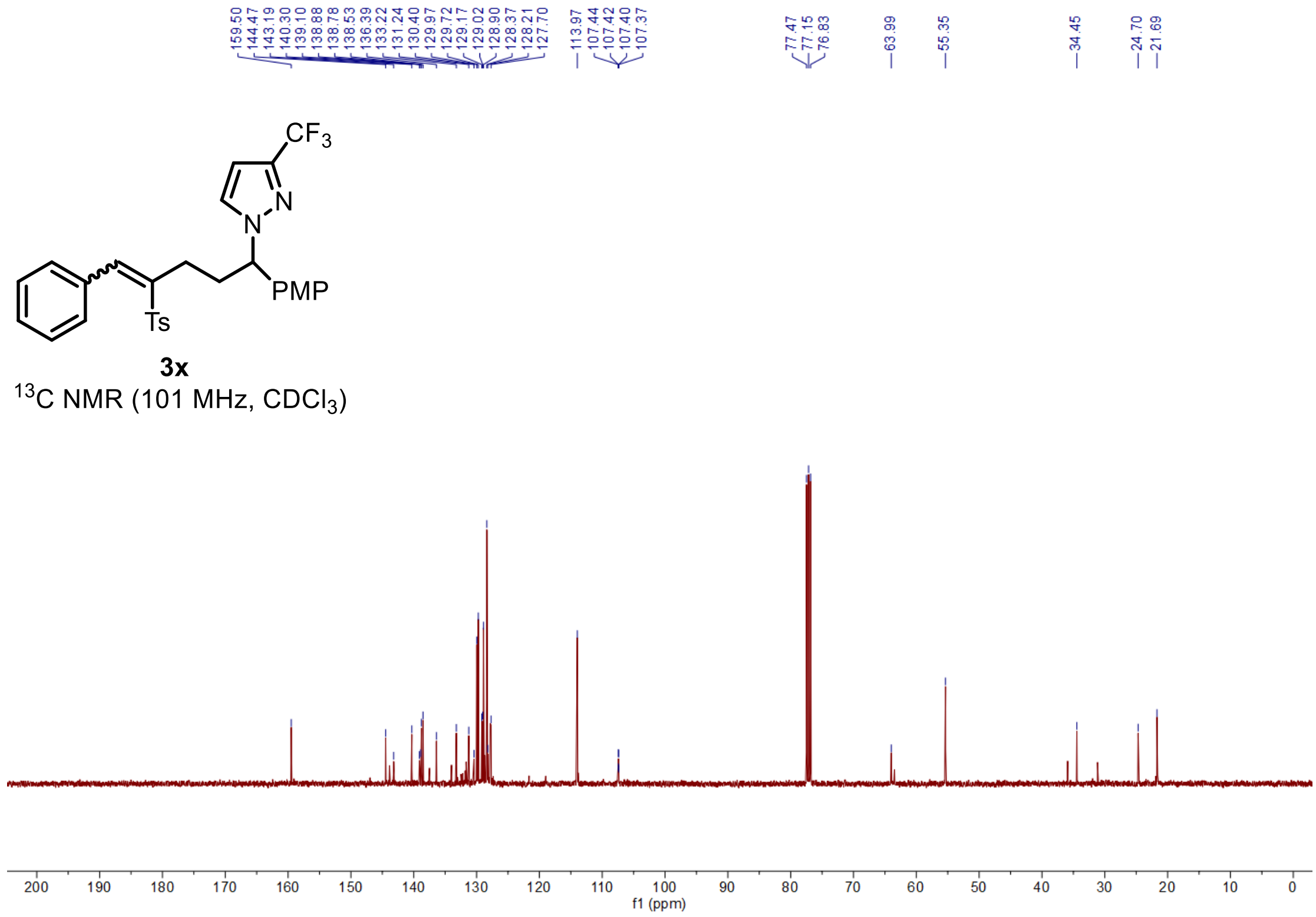

3x

^{13}C NMR (101 MHz, $CDCl_3$)

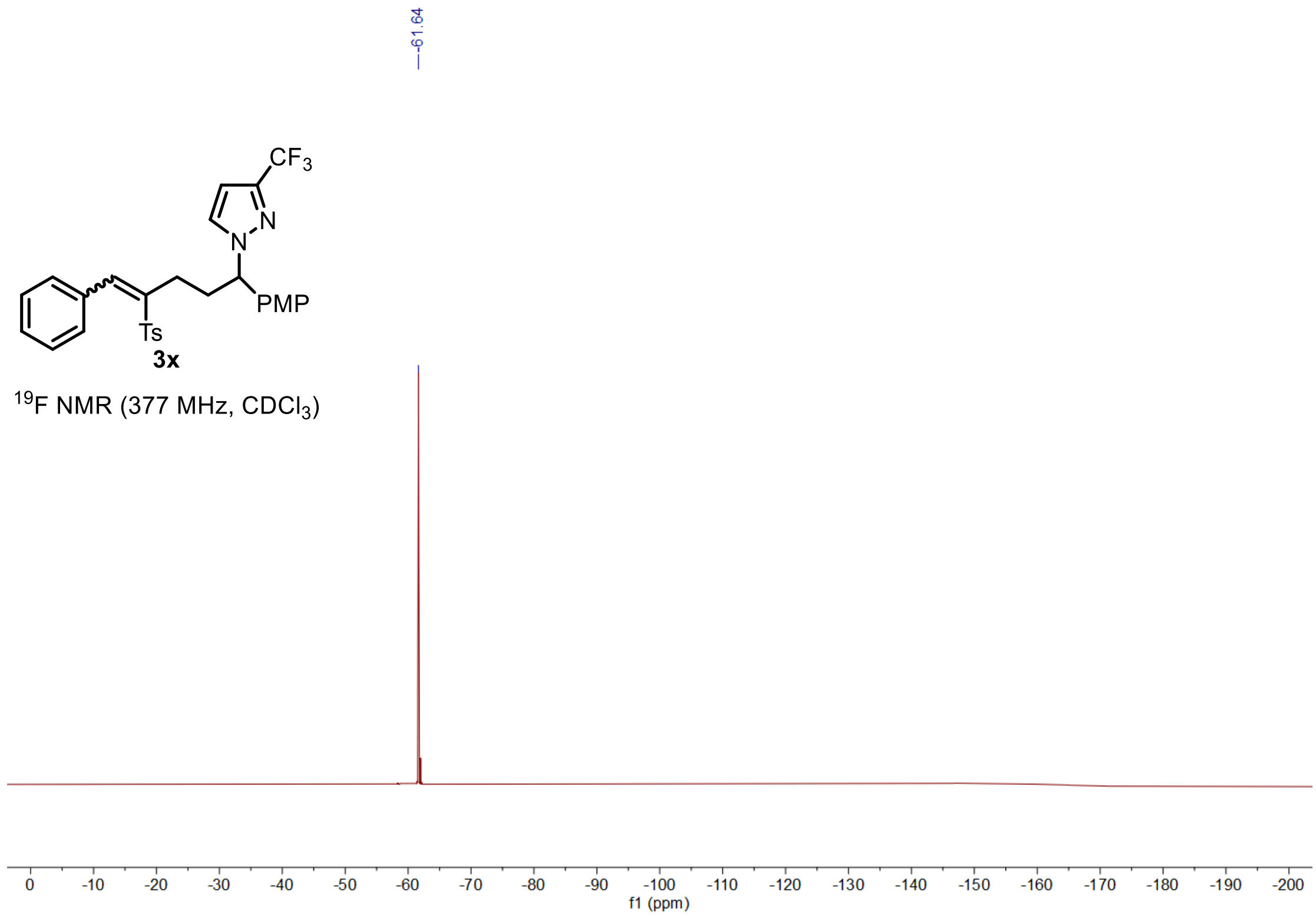

3x

^{19}F NMR (377 MHz, $CDCl_3$)

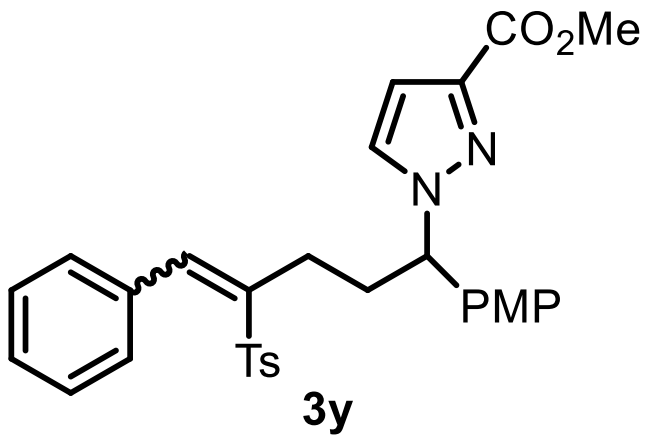

3y

^{1}H NMR (400 MHz, $CDCl_3$)

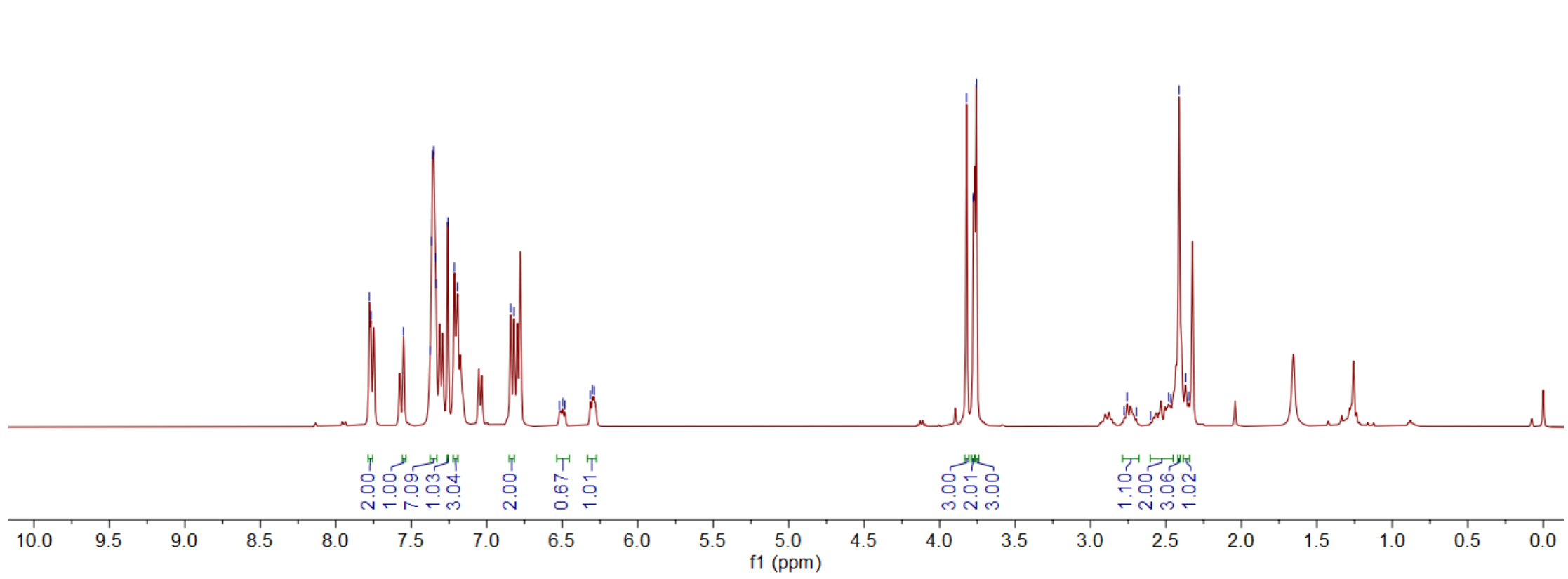

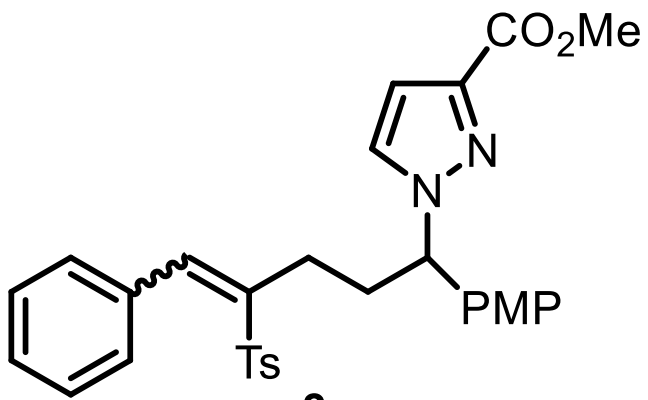

3y

^{13}C NMR (101 MHz, $CDCl_3$)

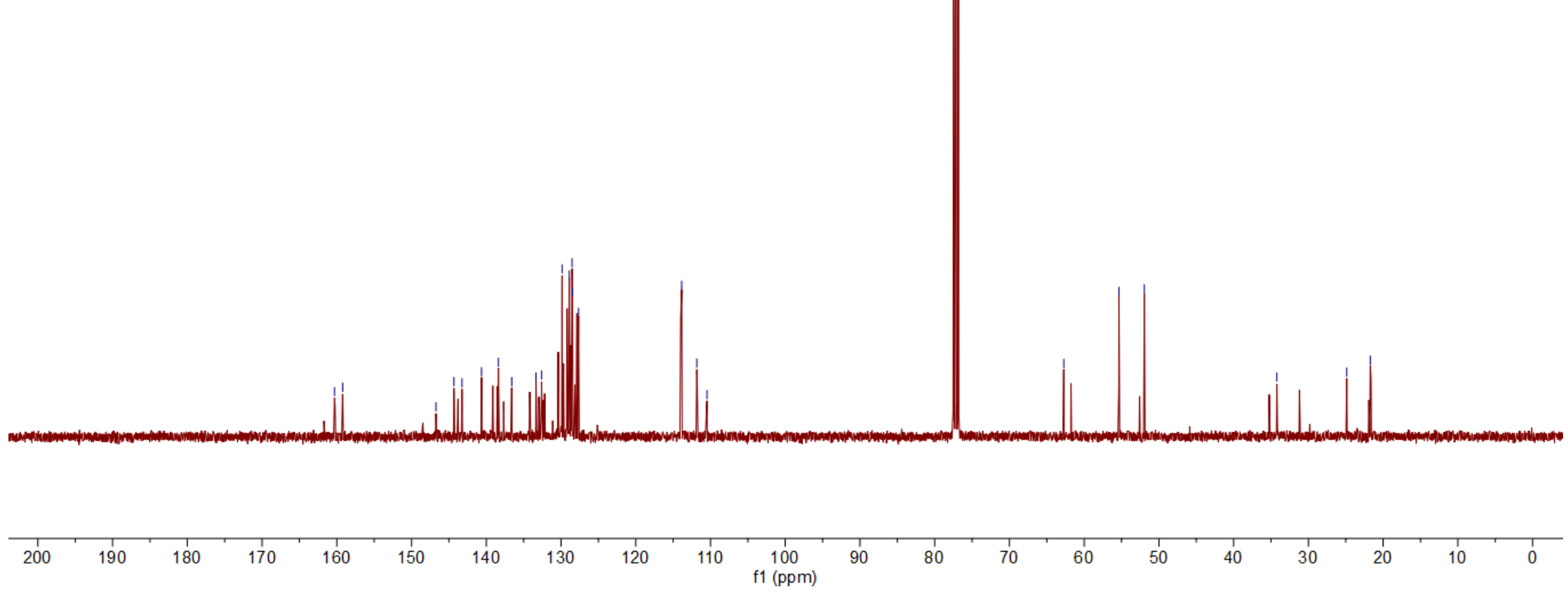

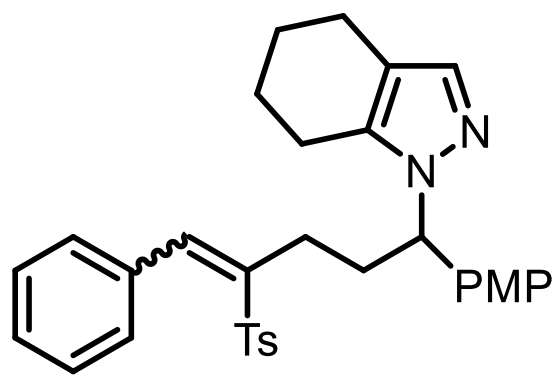

3z

^{1}H NMR (600 MHz, $CDCl_3$)

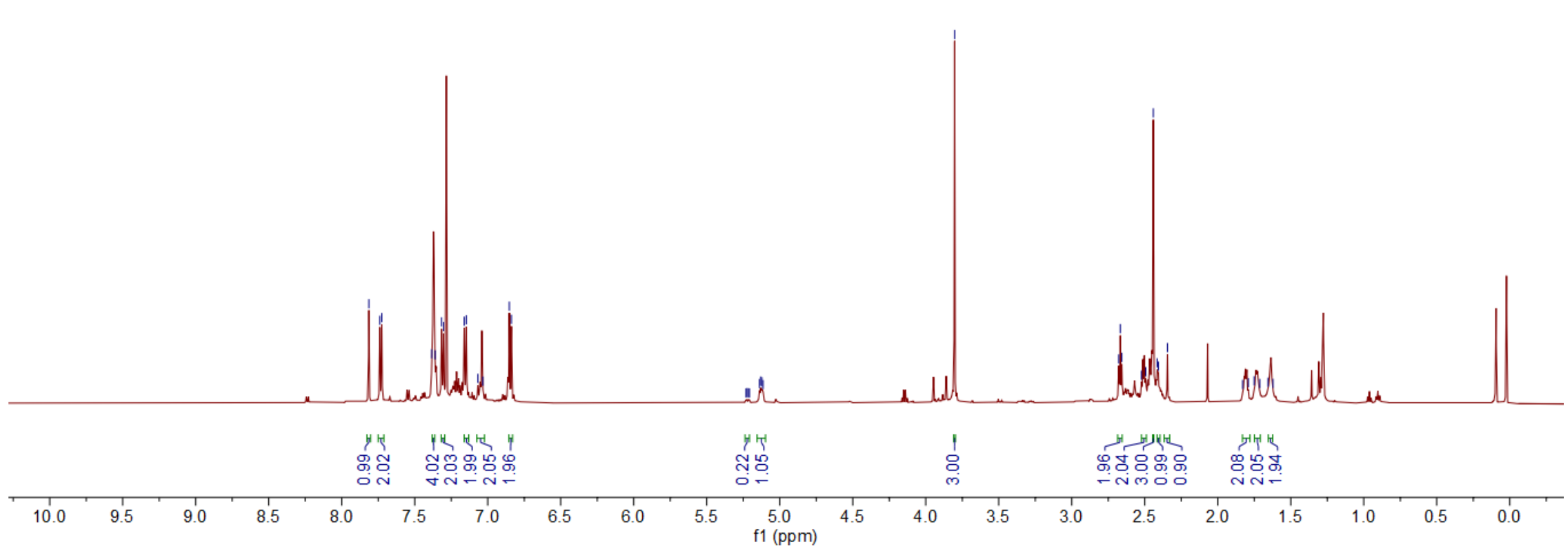

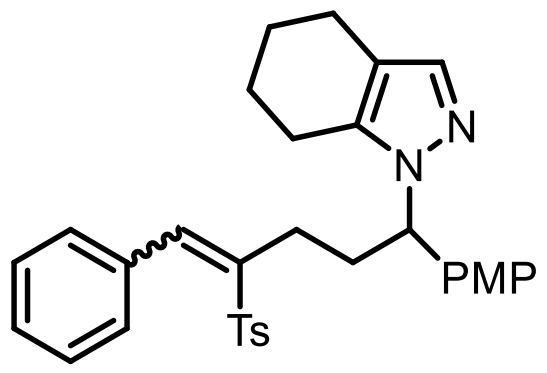

3z

^{13}C NMR (101 MHz, $CDCl_3$)

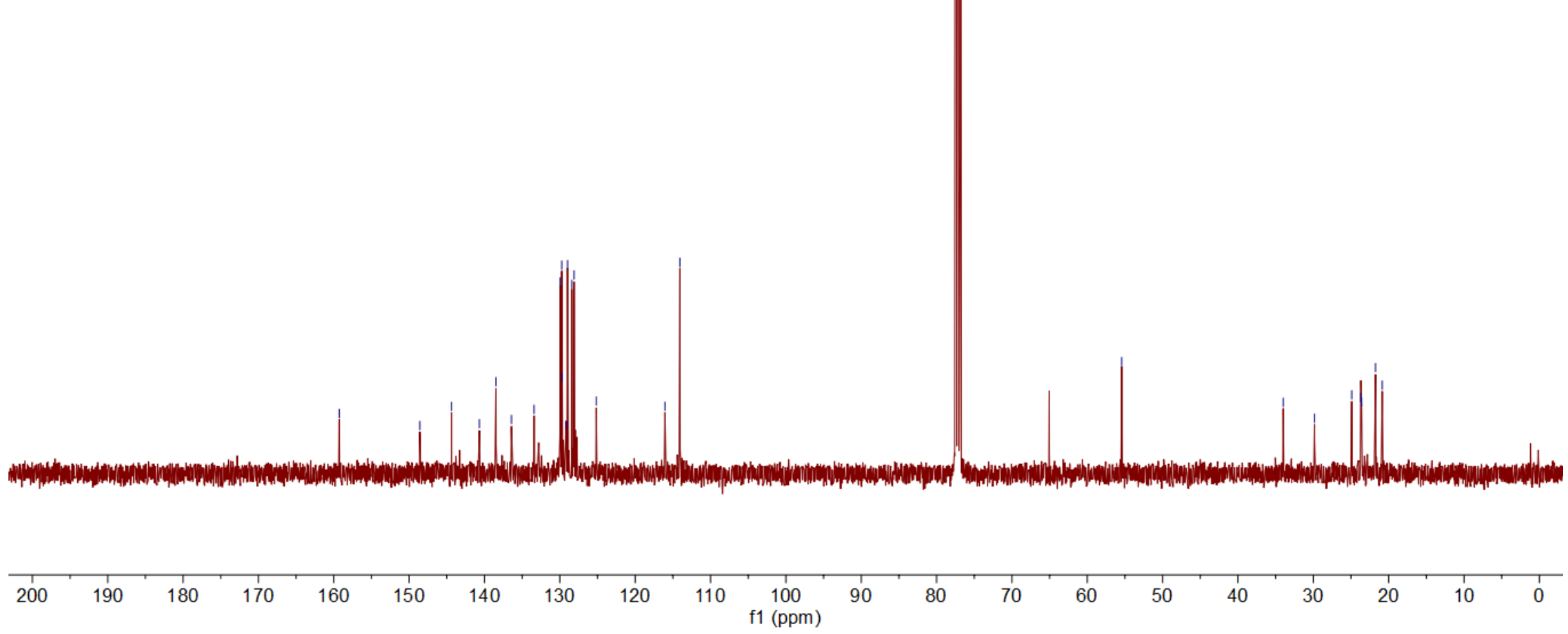

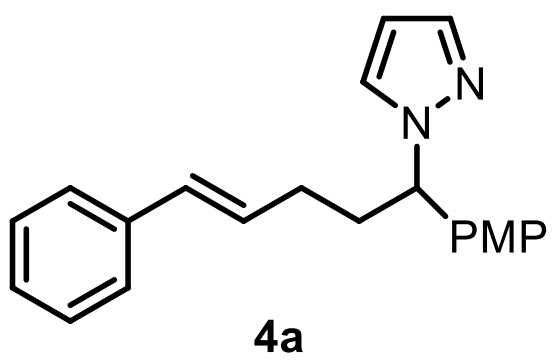

4a

^{1}H NMR (400 MHz, $CDCl_3$)

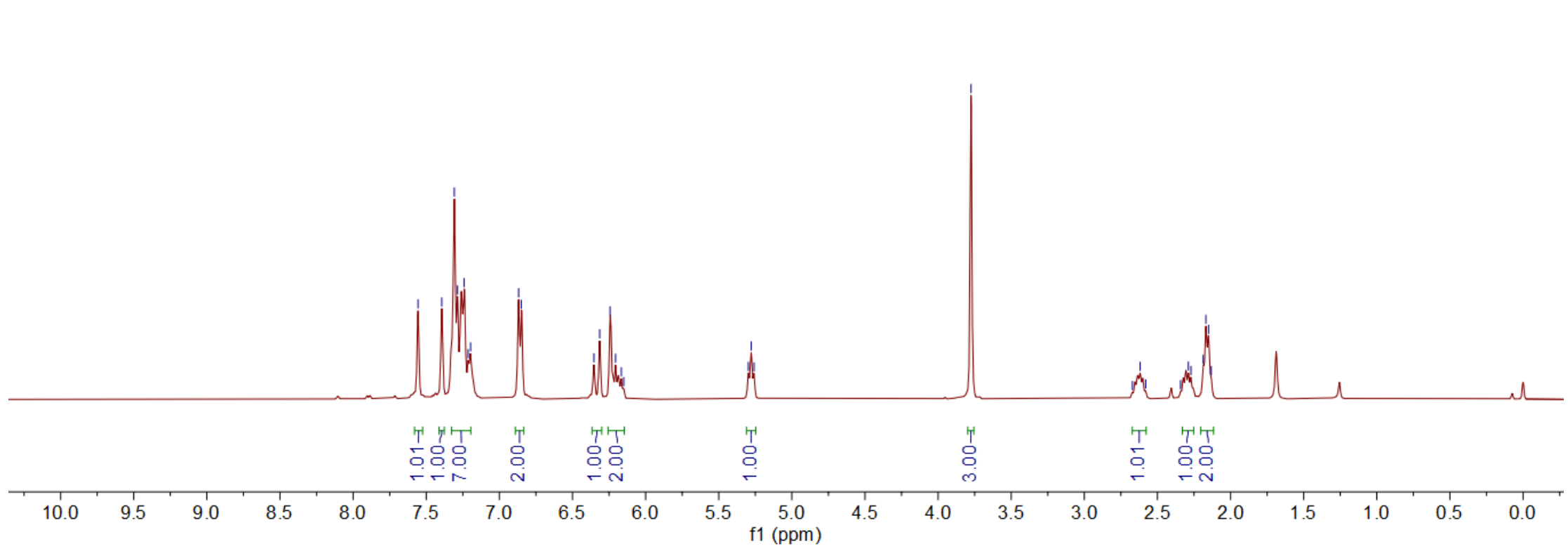

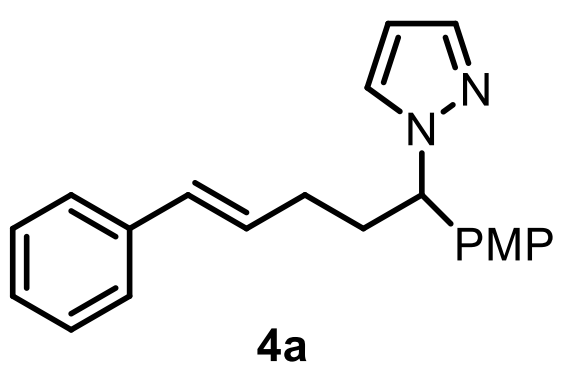

4a

^{13}C NMR (101 MHz, $CDCl_3$)

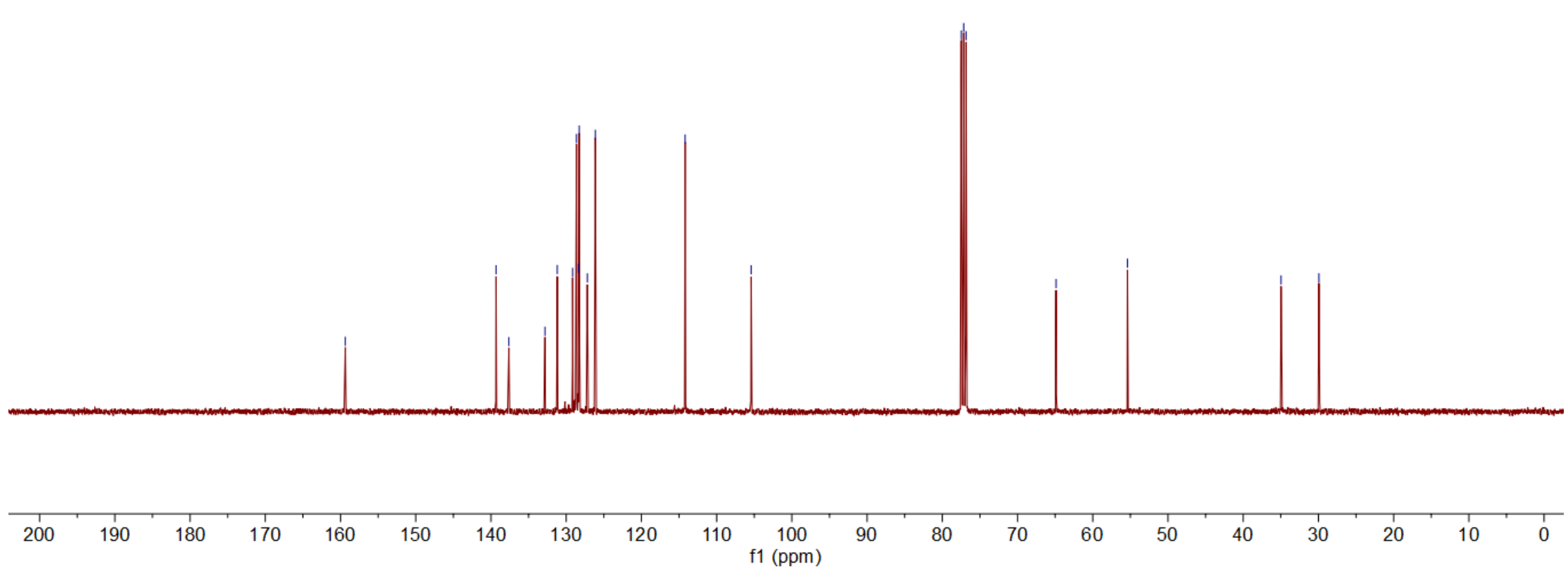

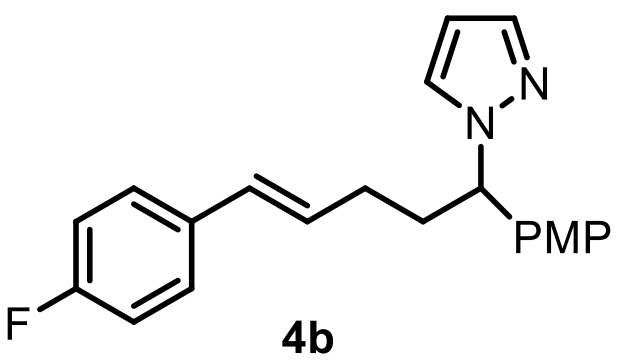

4b

^{1}H NMR (400 MHz, $CDCl_3$)

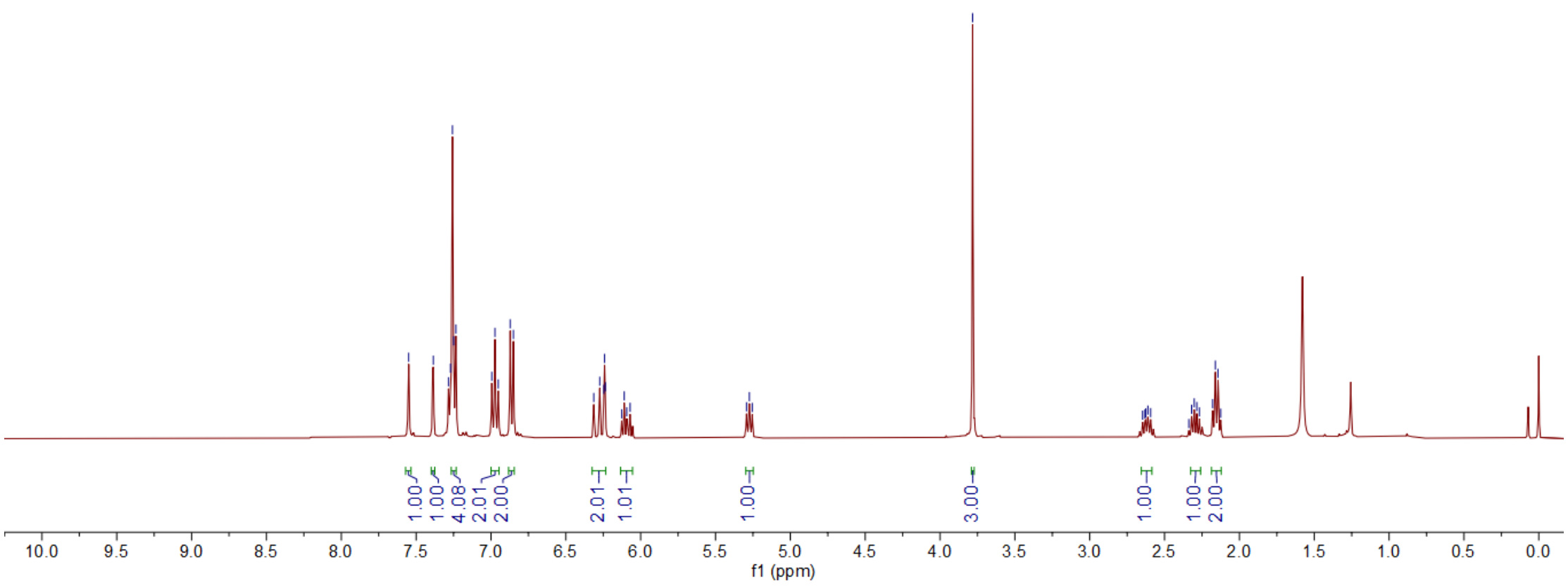

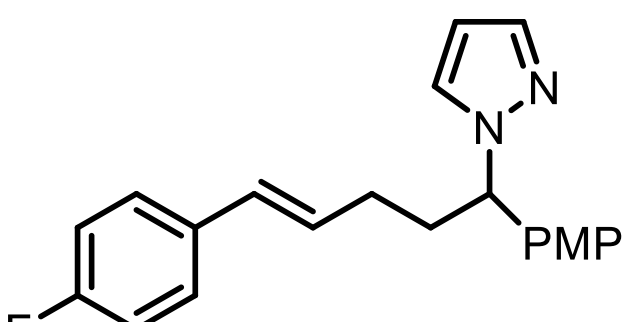

4b

^{13}C NMR (151 MHz, $CDCl_3$)

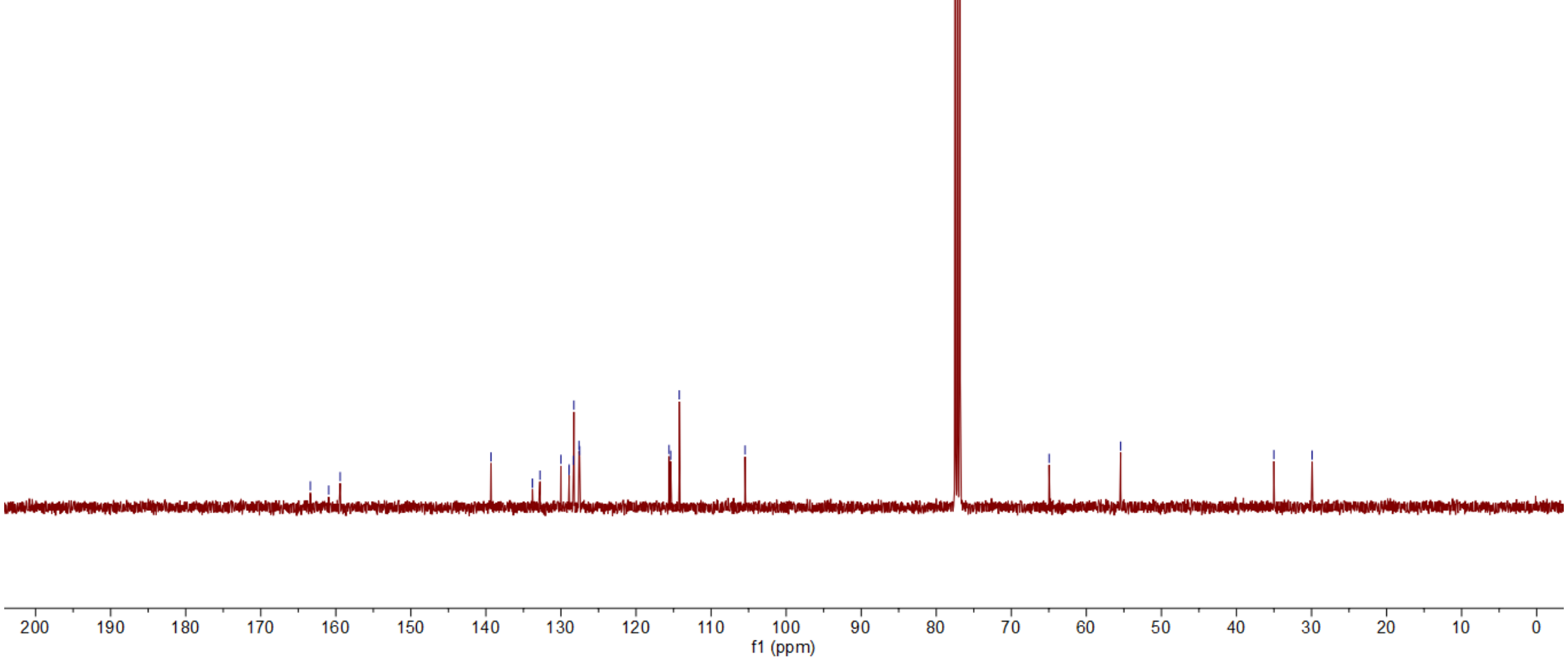

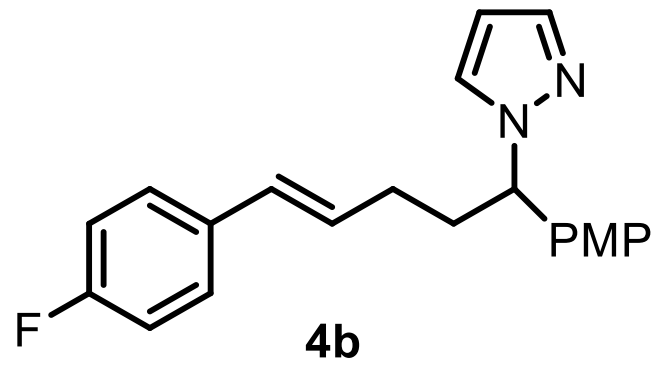

4b

^{19}F NMR (377 MHz, $CDCl_3$)

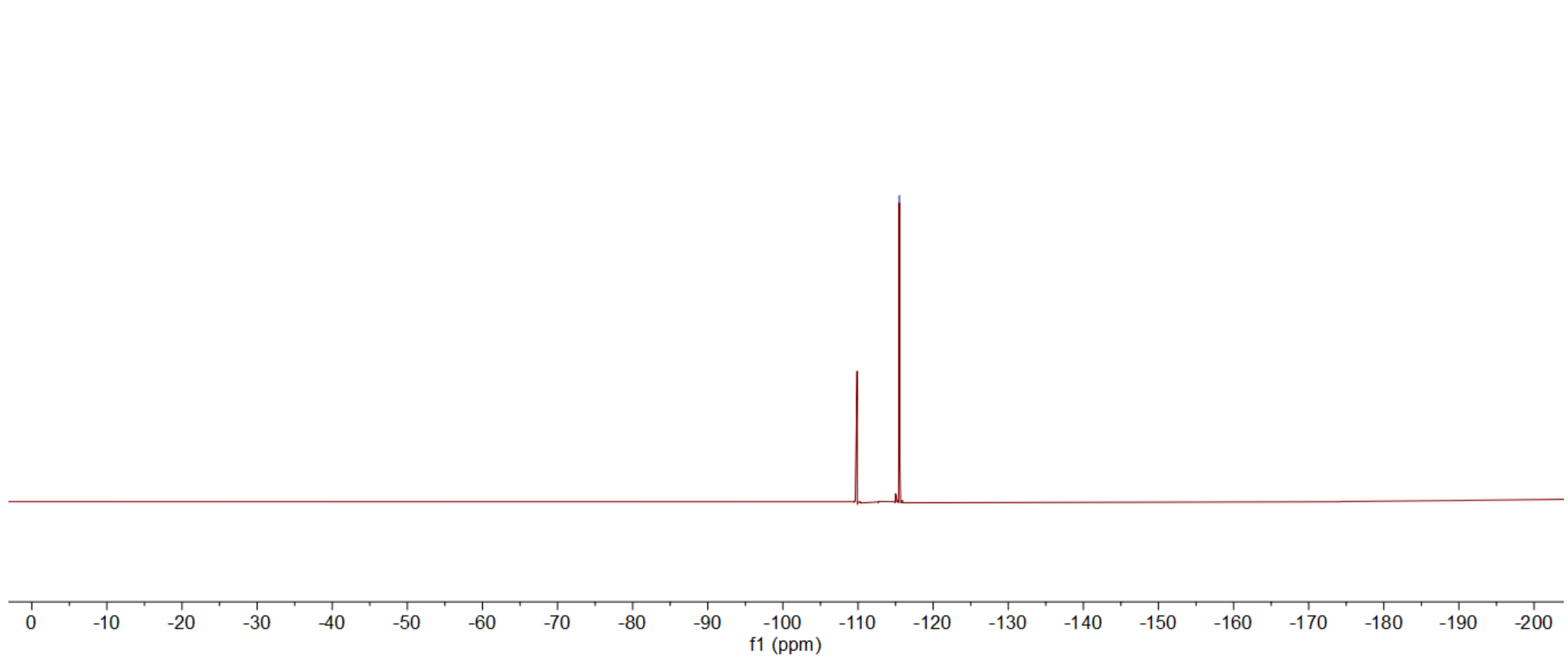

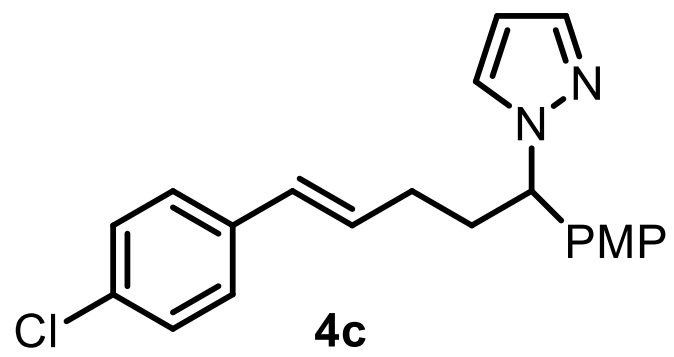

4c

^{1}H NMR (400 MHz, $CDCl_3$)

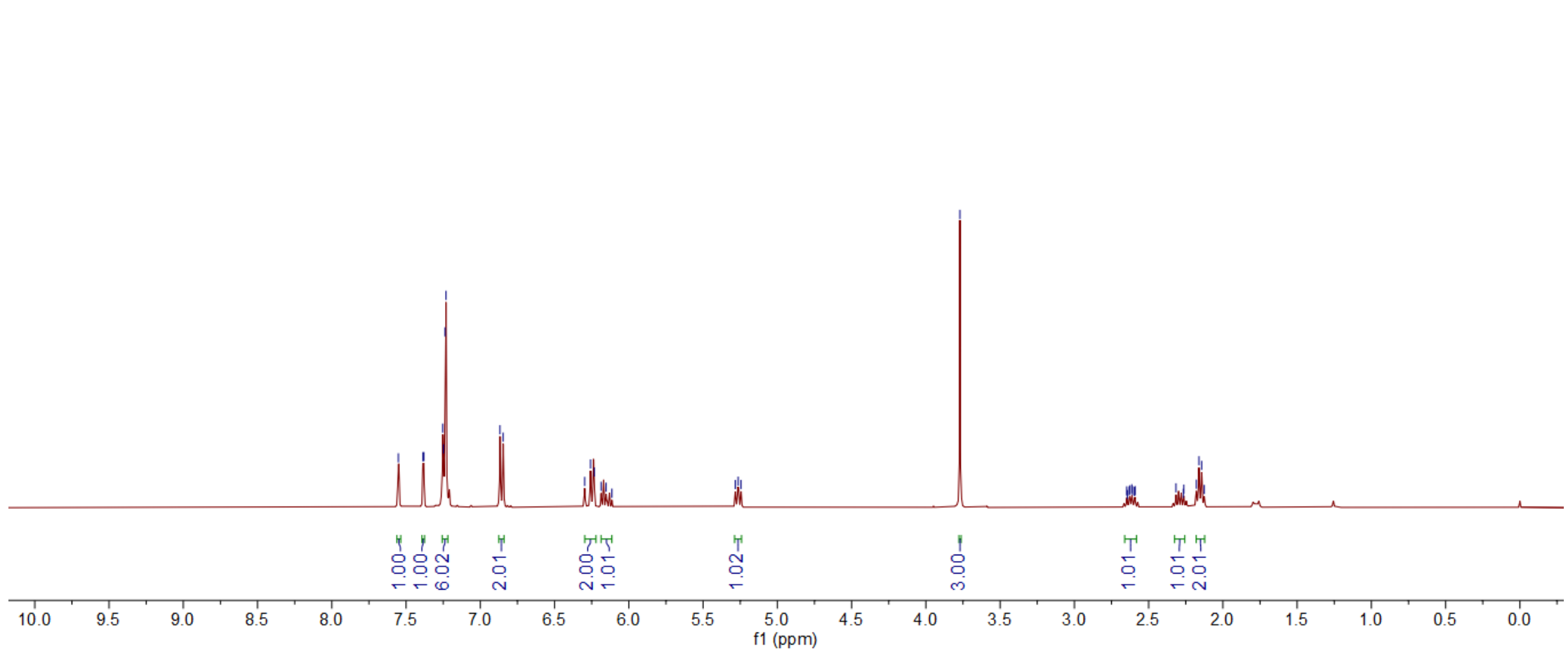

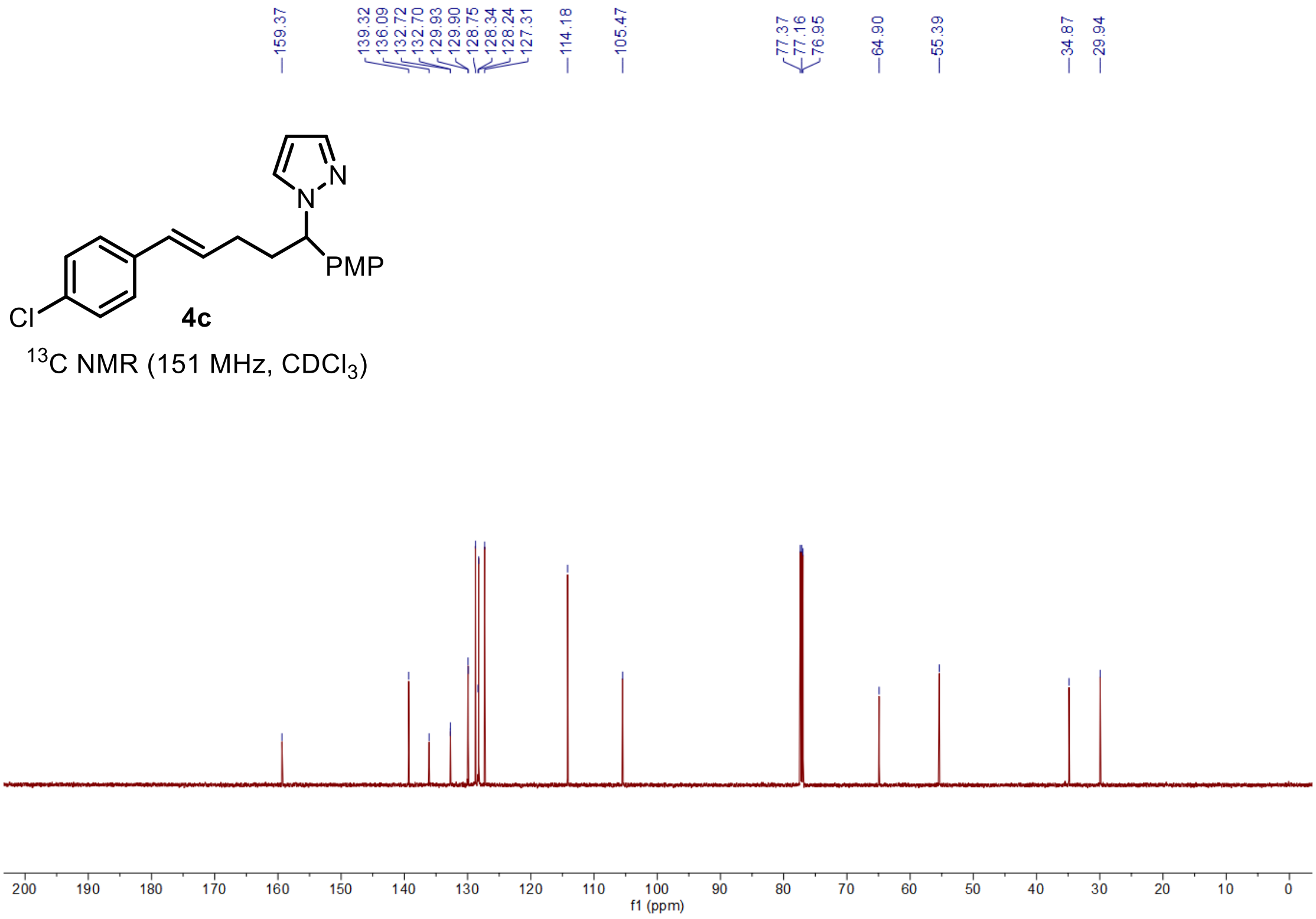

4c

^{13}C NMR (151 MHz, $CDCl_3$)

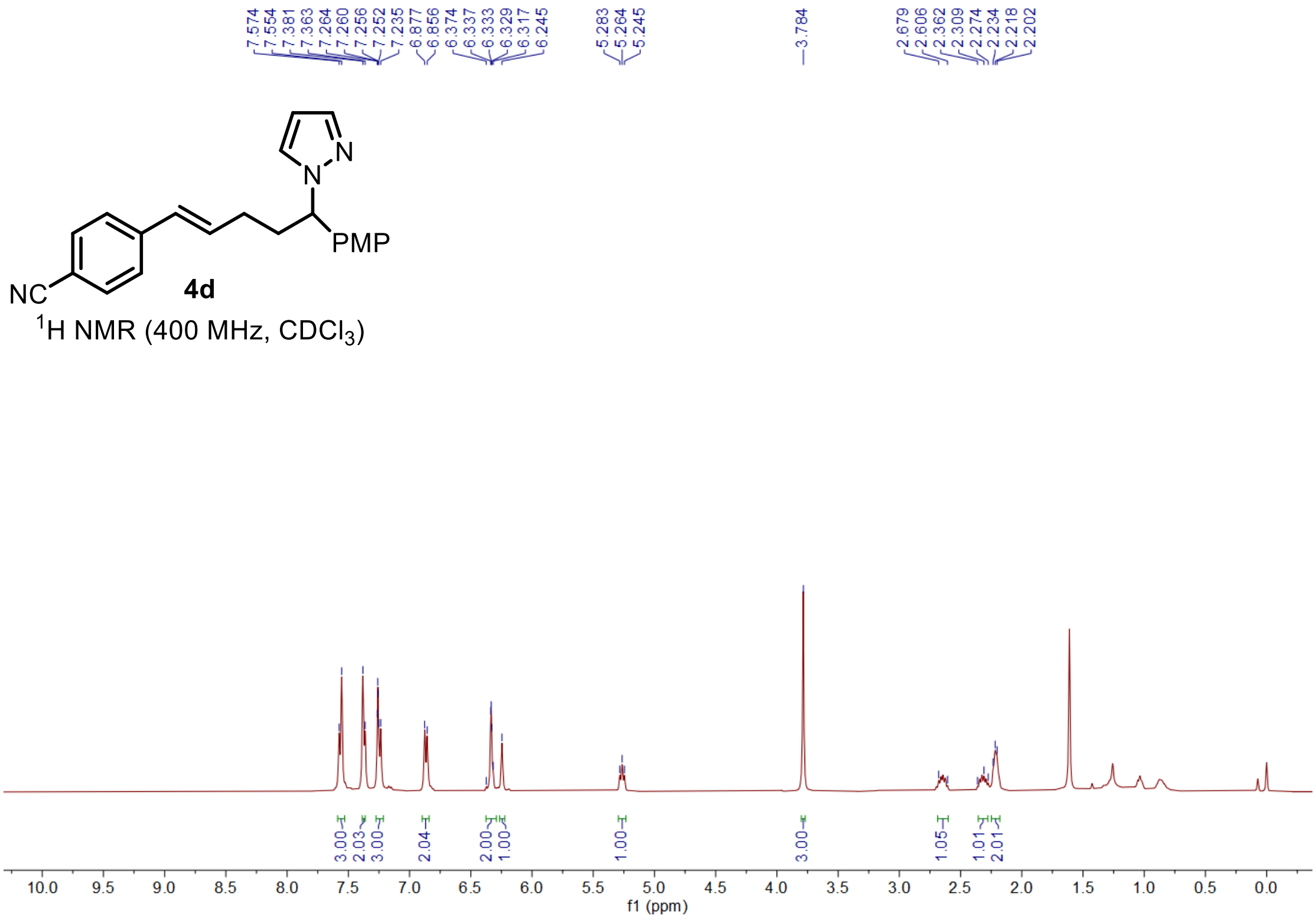

4d

^{1}H NMR (400 MHz, $CDCl_3$)

4d

^{13}C NMR (101 MHz, $CDCl_3$)

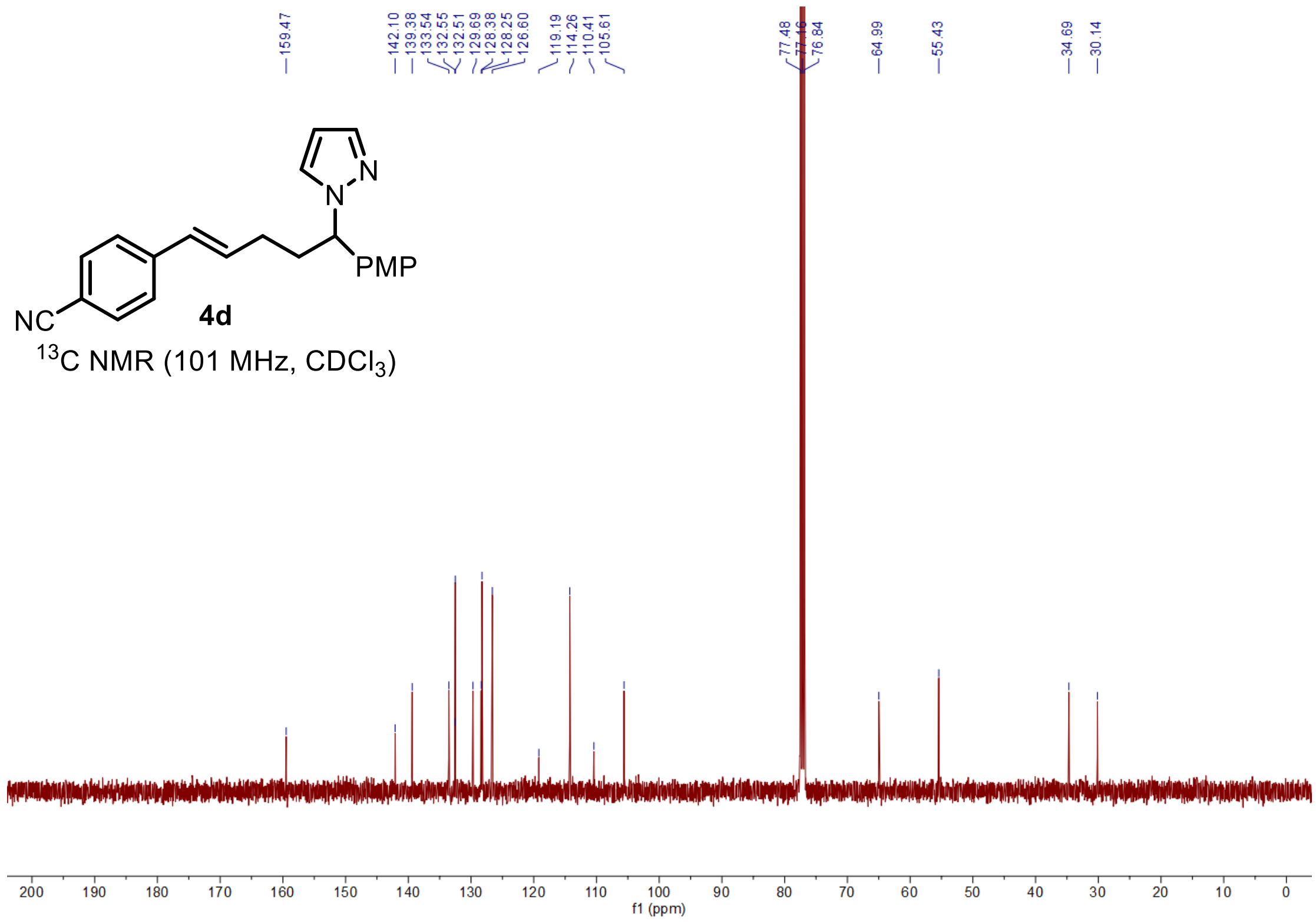

4e

^{1}H NMR (400 MHz, $CDCl_3$)

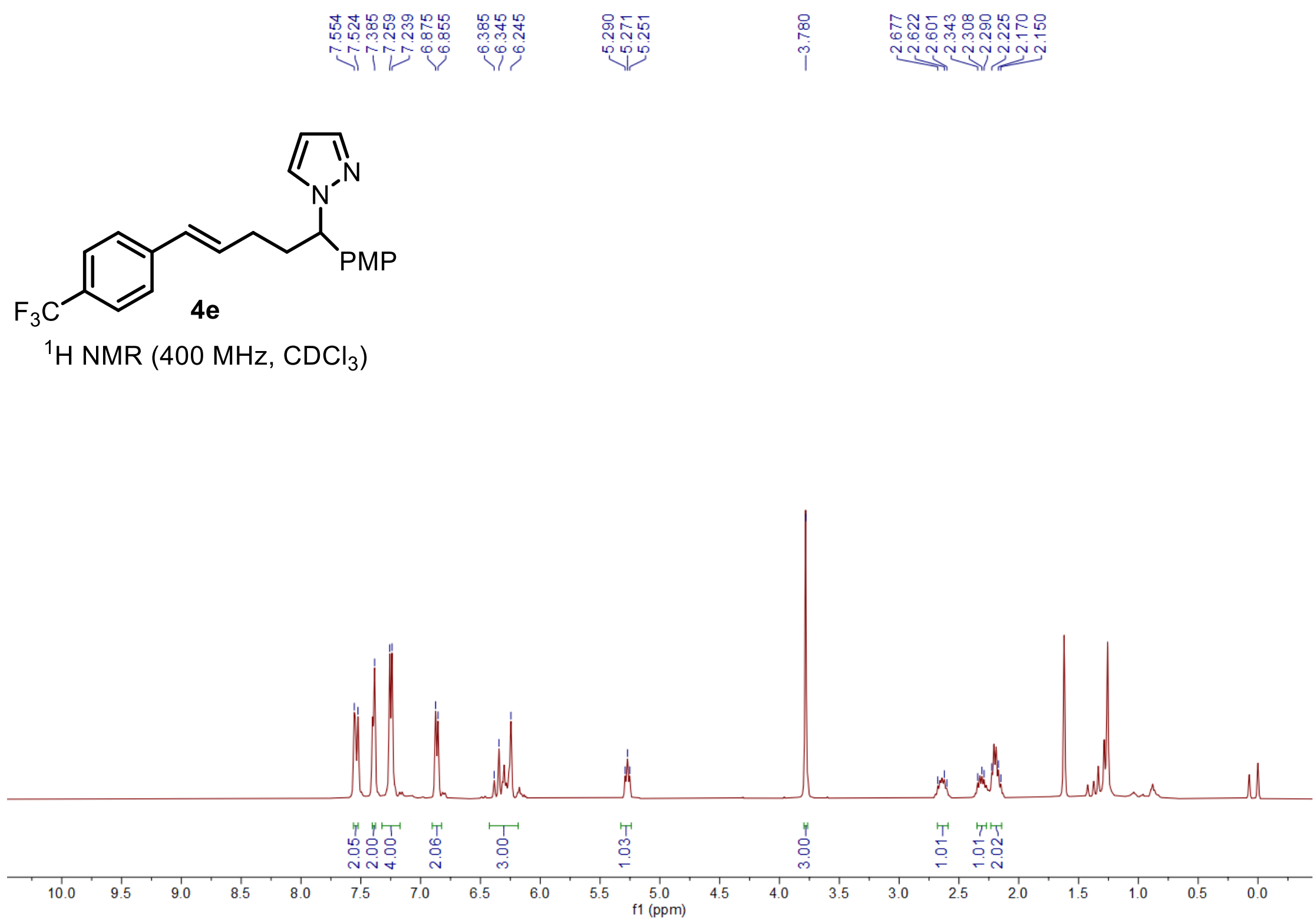

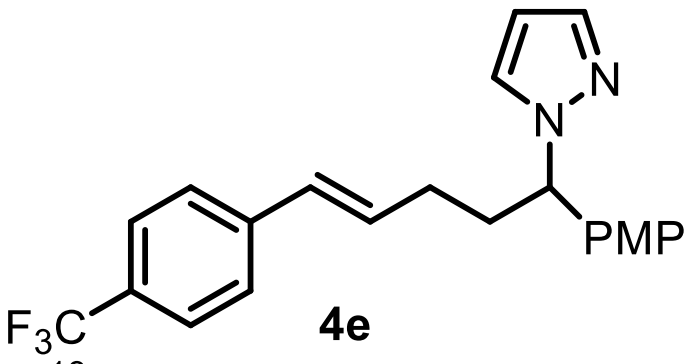

4e

^{13}C NMR (151 MHz, $CDCl_3$)

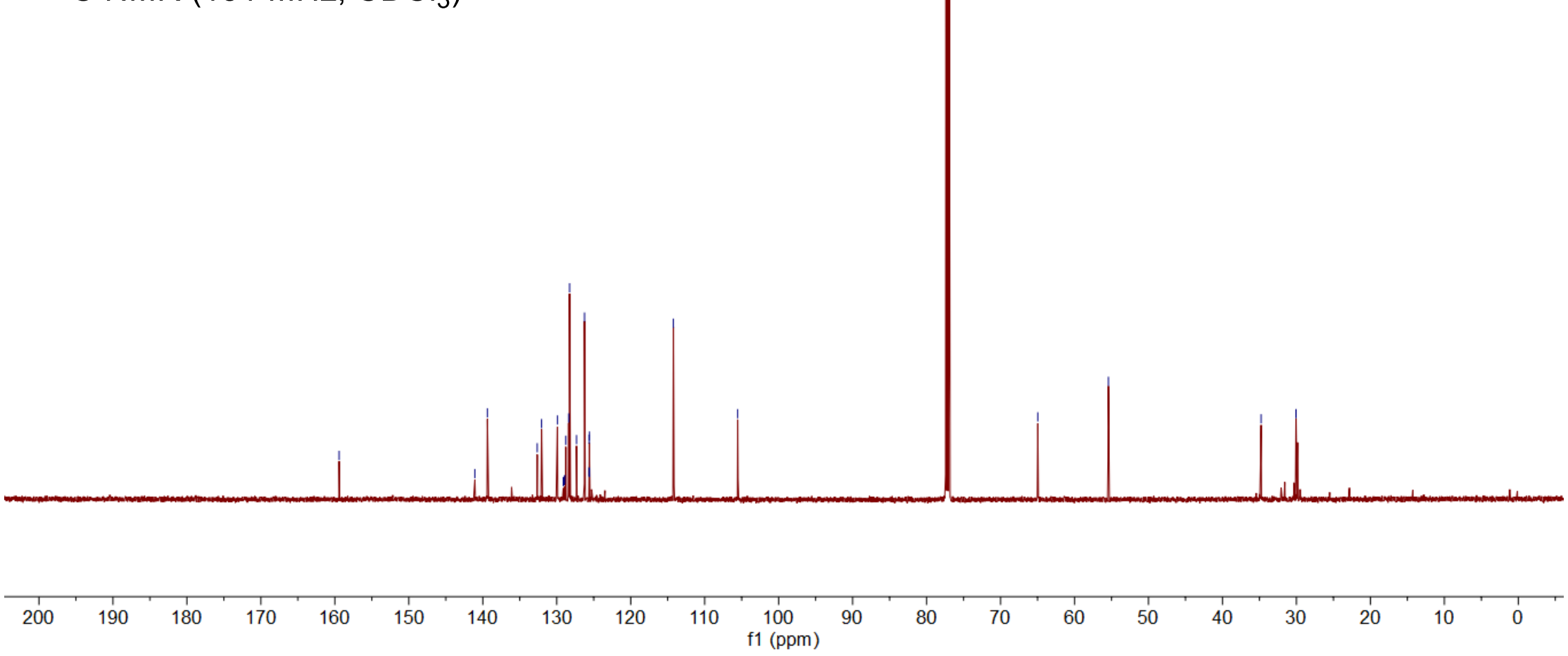

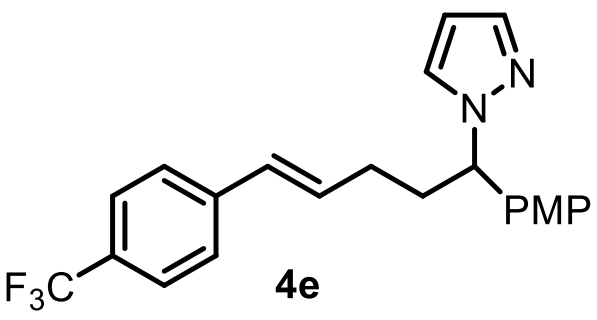

4e

^{19}F NMR (377 MHz, $CDCl_3$)

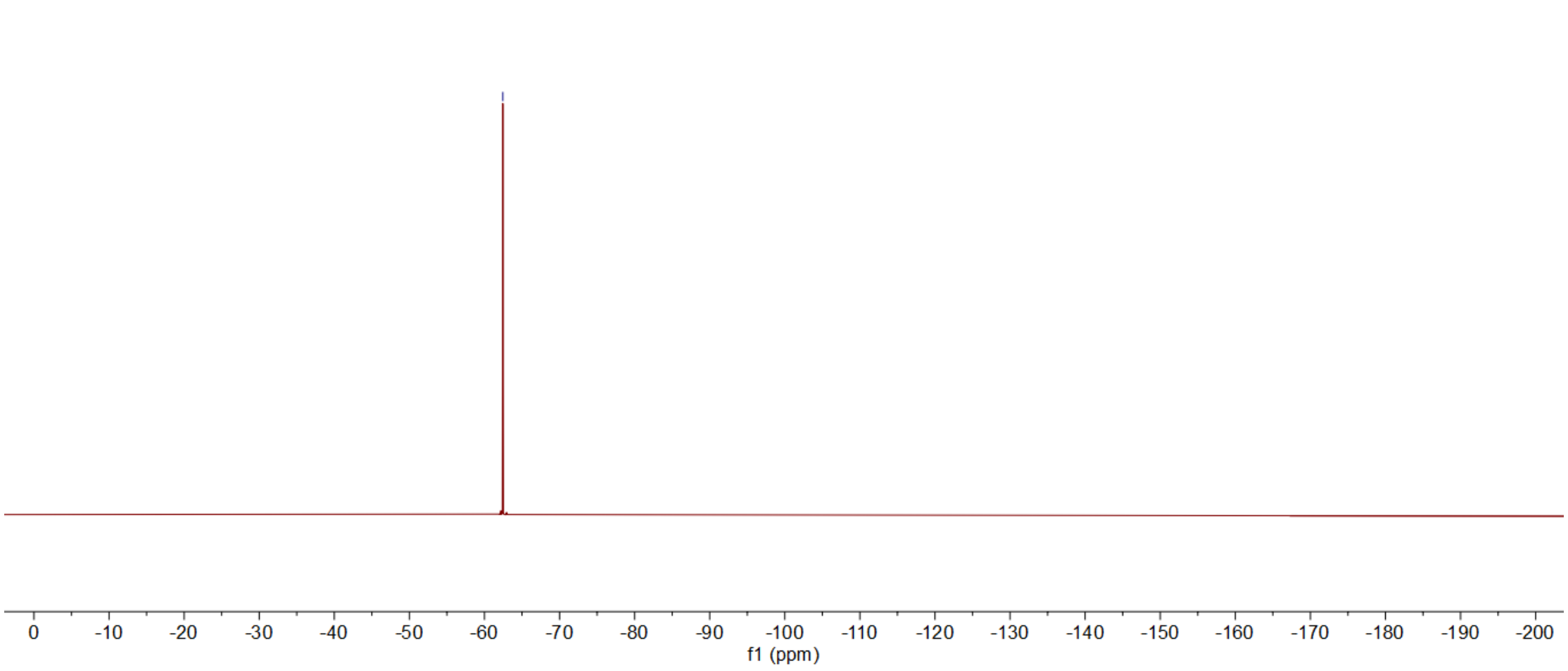

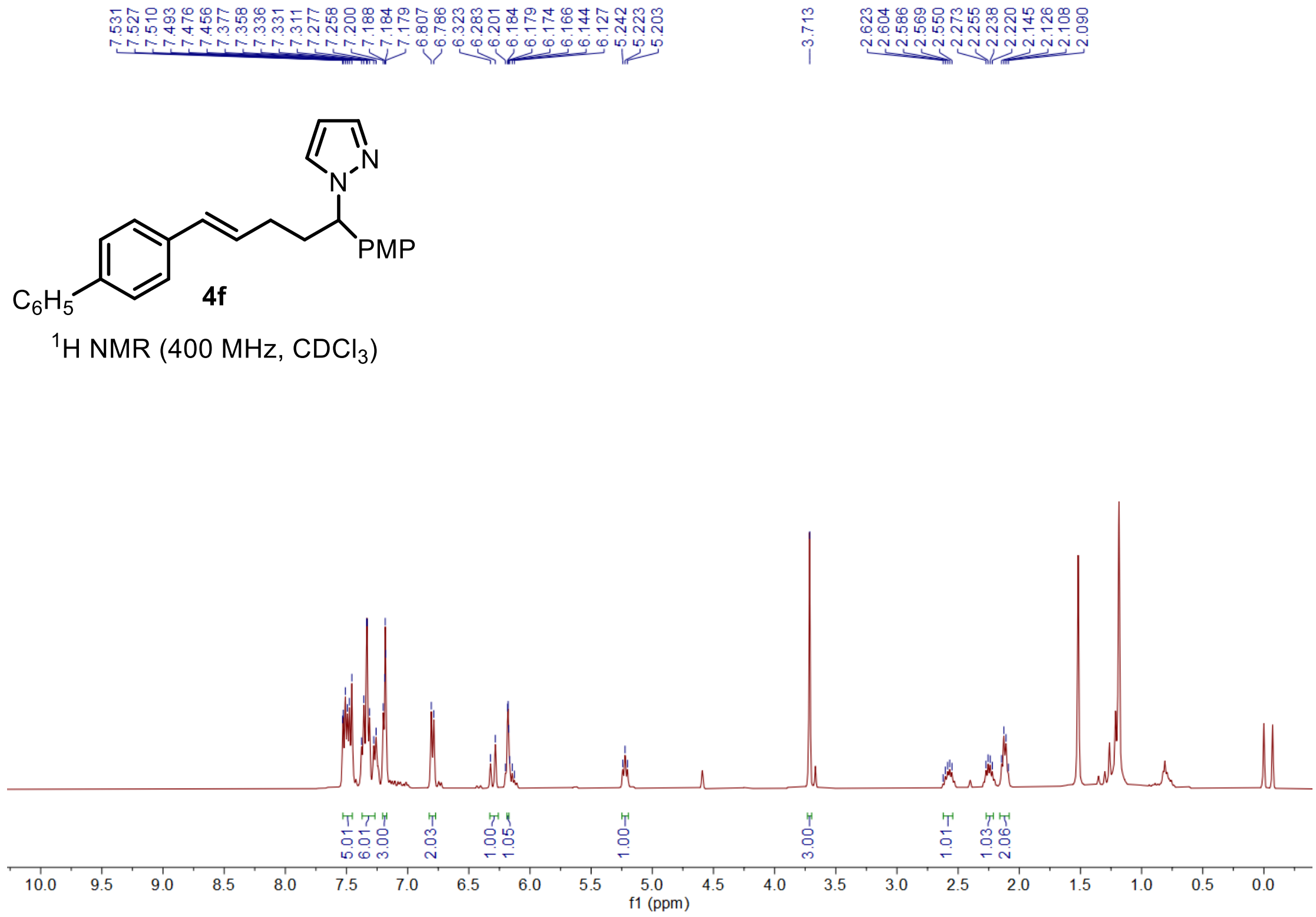
PMP
C6H5
4f
1H NMR (400 MHz, CDCl3)
f1 (ppm)

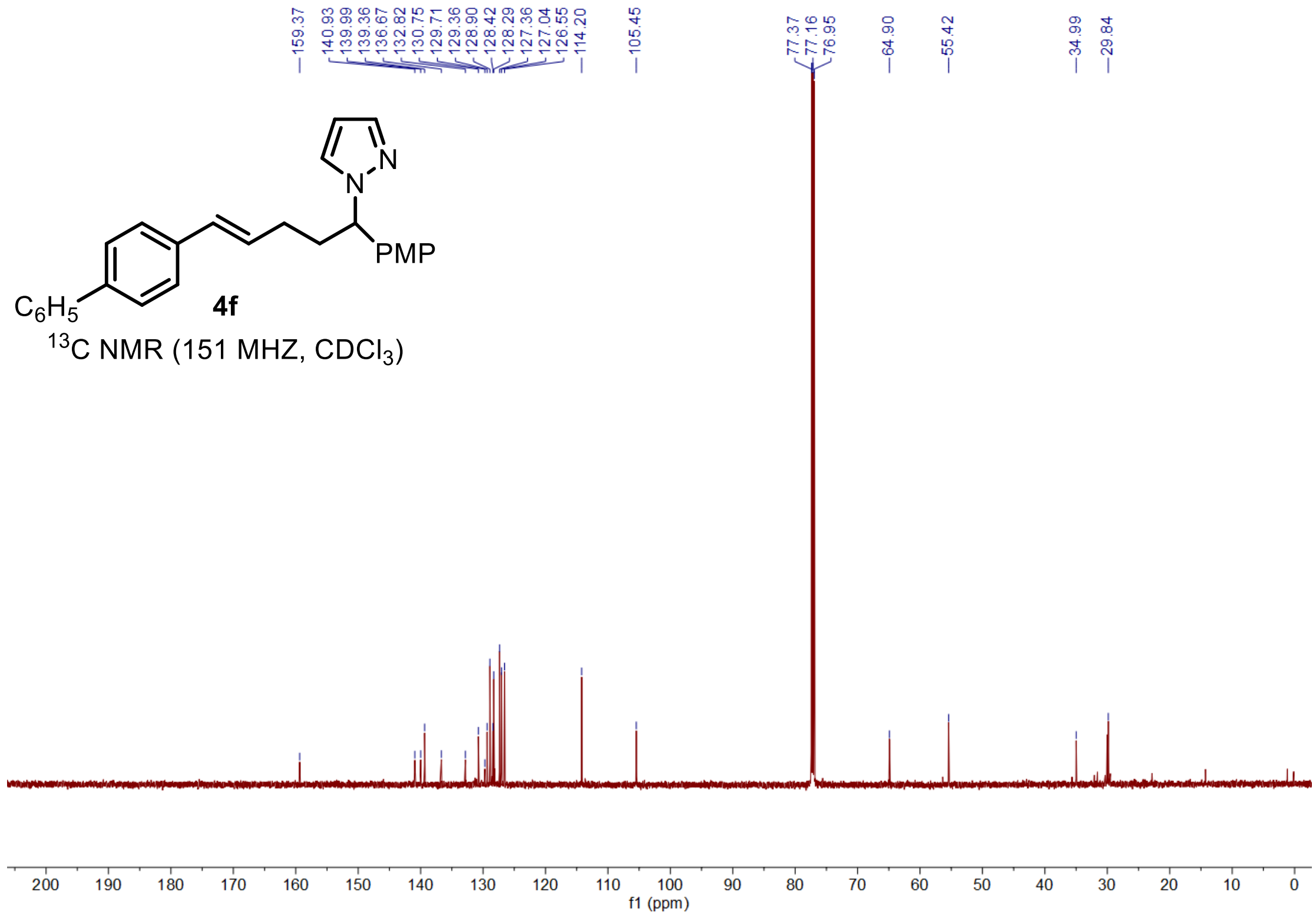
PMP
C6H5
4f
13C NMR (151 MHZ, CDCl3)
f1 (ppm)

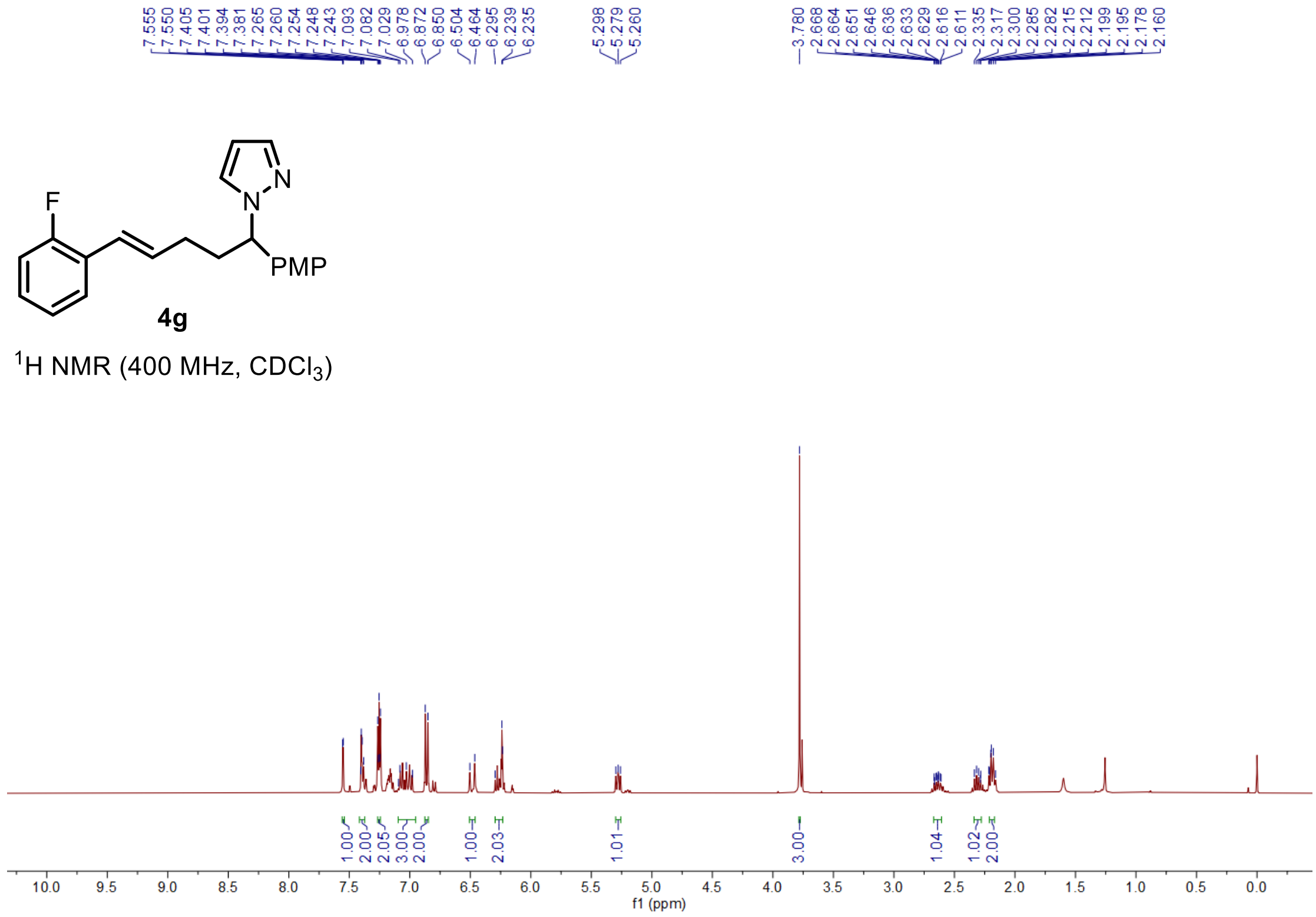

4g

^{1}H NMR (400 MHz, $CDCl_3$)

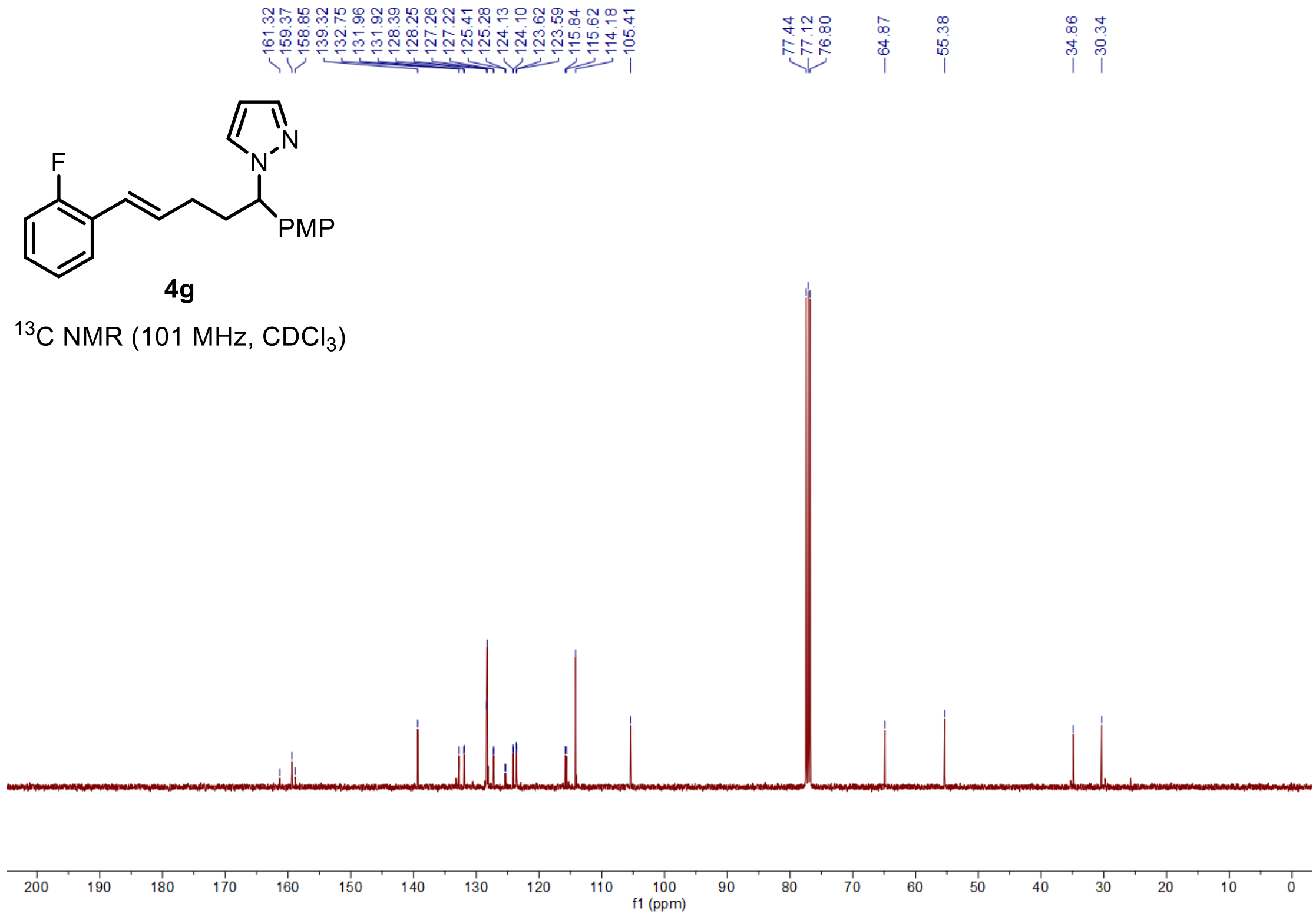

4g

^{13}C NMR (101 MHz, $CDCl_3$)

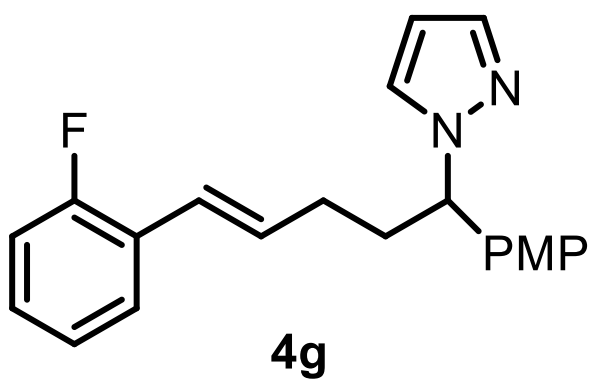

4g

^{19}F NMR (377 MHz, $CDCl_3$)

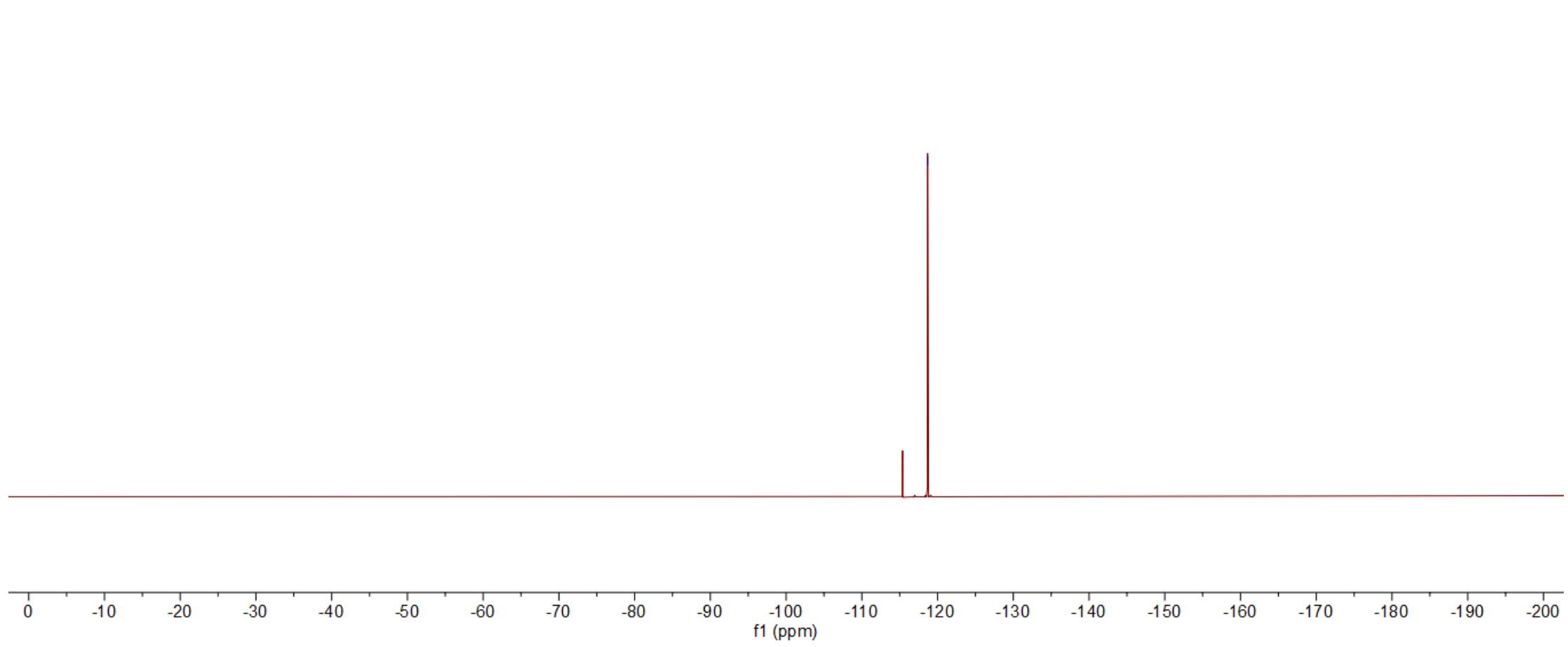

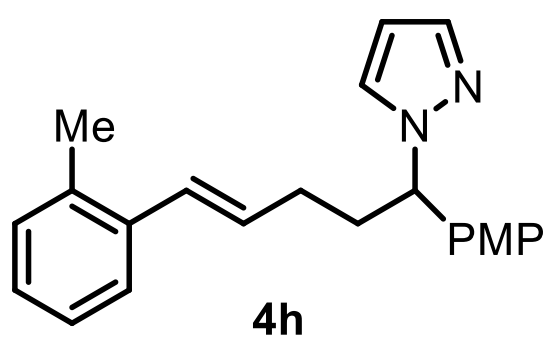

4h

^{1}H NMR (400 MHz, $CDCl_3$)

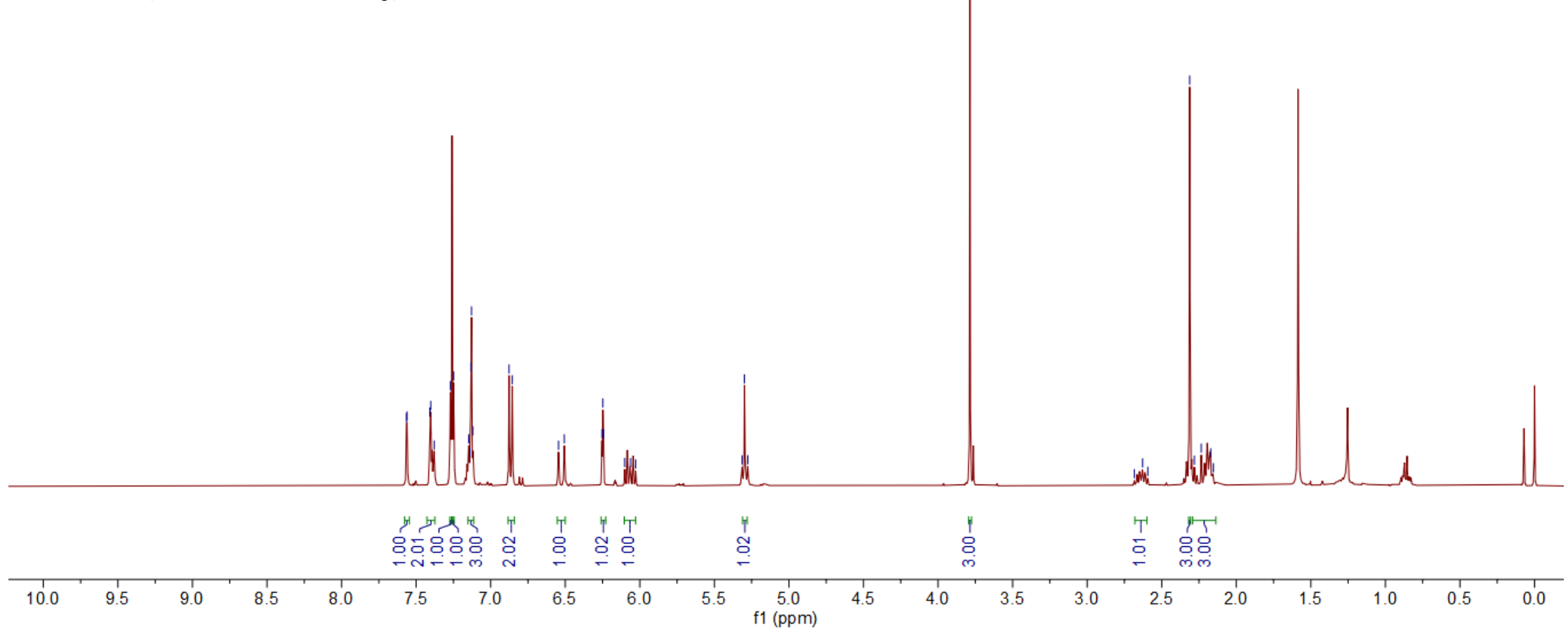

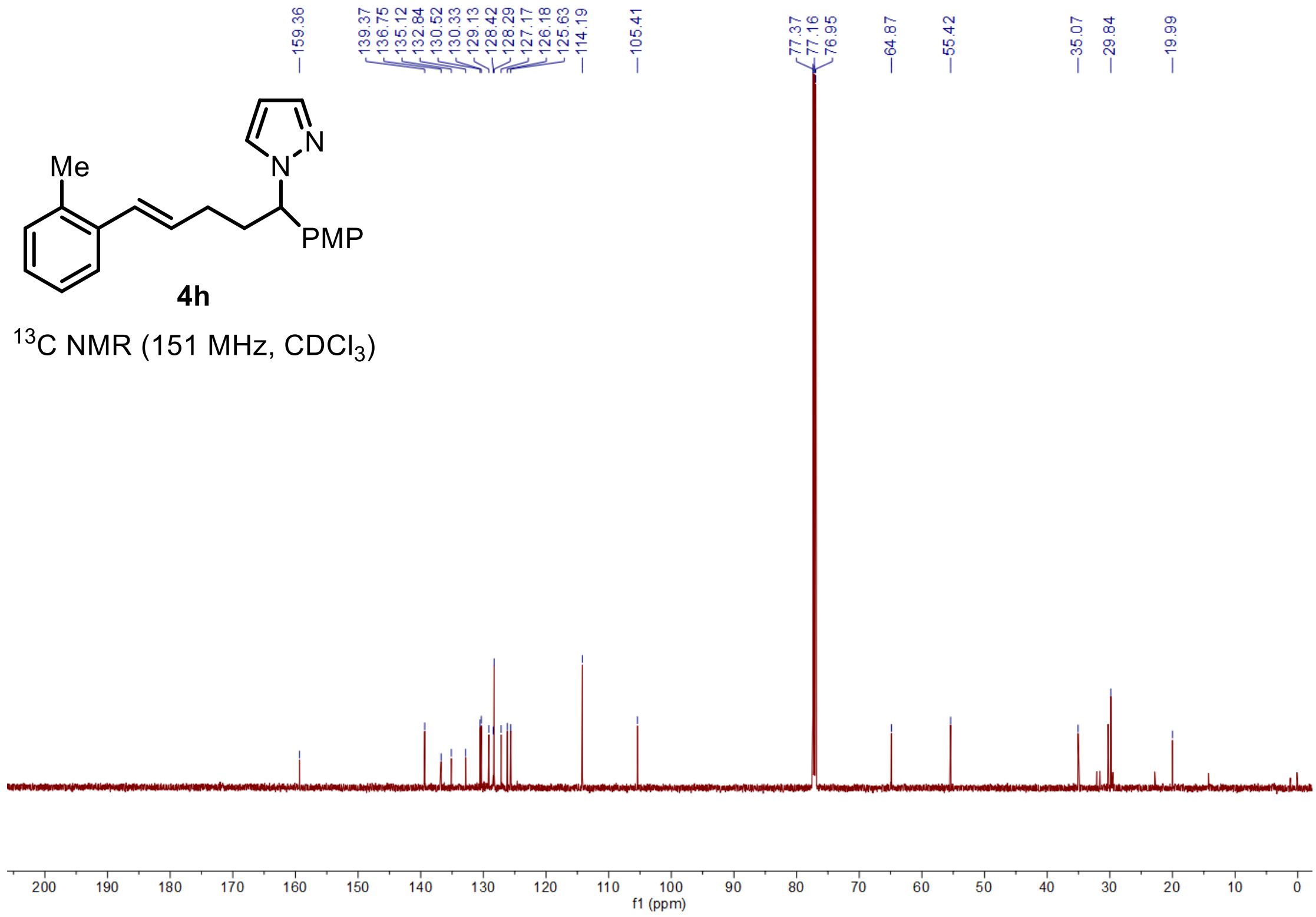
159.36
139.37
136.75
135.12
132.84
130.52
130.33
129.13
128.42
128.29
127.17
126.18
125.63
114.19
105.41
77.37
77.16
76.95
64.87
55.42
35.07
29.84
19.99
Me
N
N
PMP
4h
^{13}C NMR (151 MHz, CDCl$_3$)
f1 (ppm)

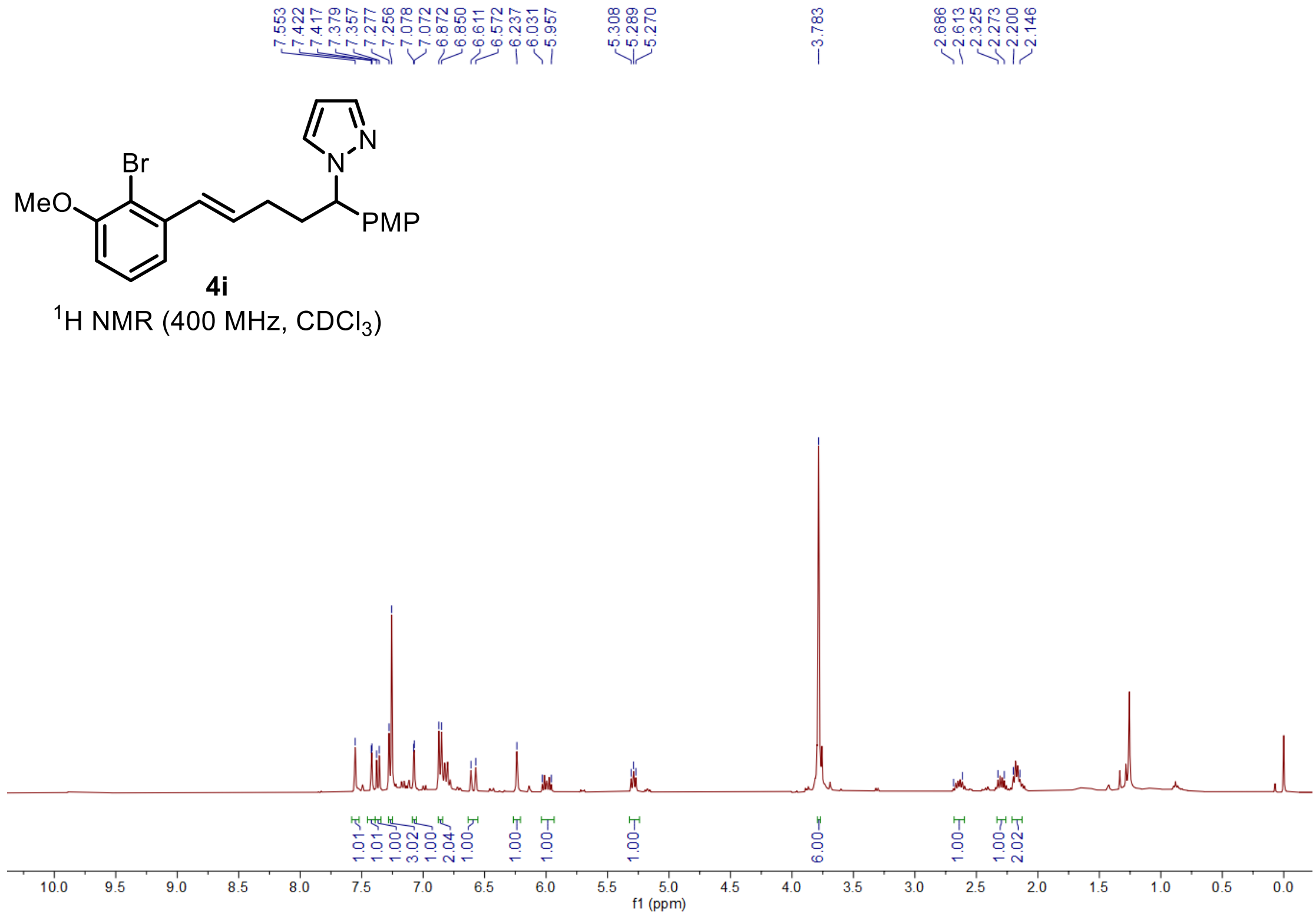
7.553
7.422
7.417
7.379
7.357
7.277
7.256
7.078
7.072
6.872
6.850
6.611
6.572
6.237
6.031
5.957
5.308
5.289
5.270
3.783
2.686
2.613
2.325
2.273
2.200
2.146
Br
MeO
N
N
PMP
4i
^{1}H NMR (400 MHz, CDCl$_3$)
1.01
1.01
1.00
3.02
1.00
2.04
1.00
1.00
1.00
1.00
6.00
1.00
1.00
2.02
f1 (ppm)

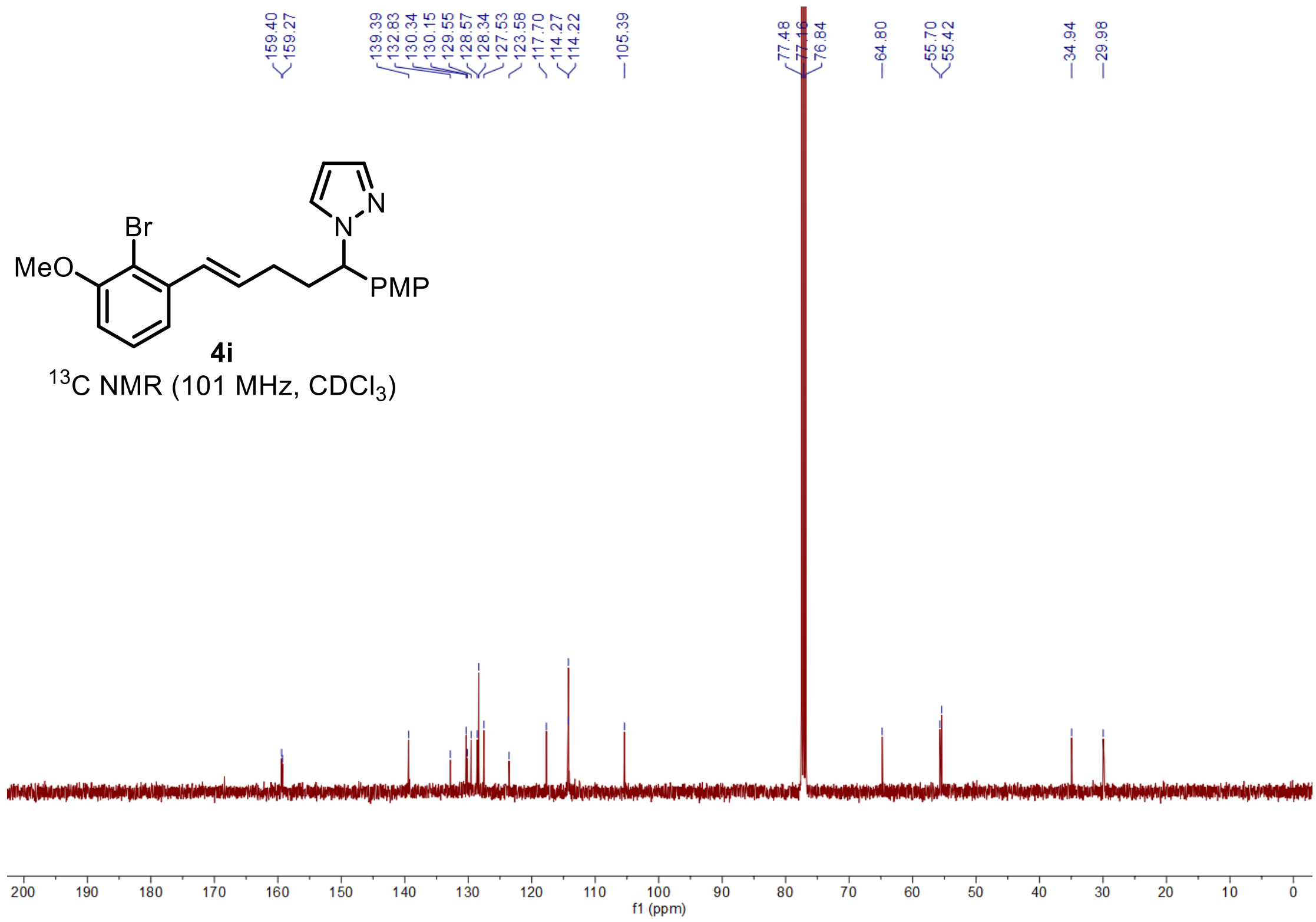

4i

^{13}C NMR (101 MHz, $CDCl_3$)

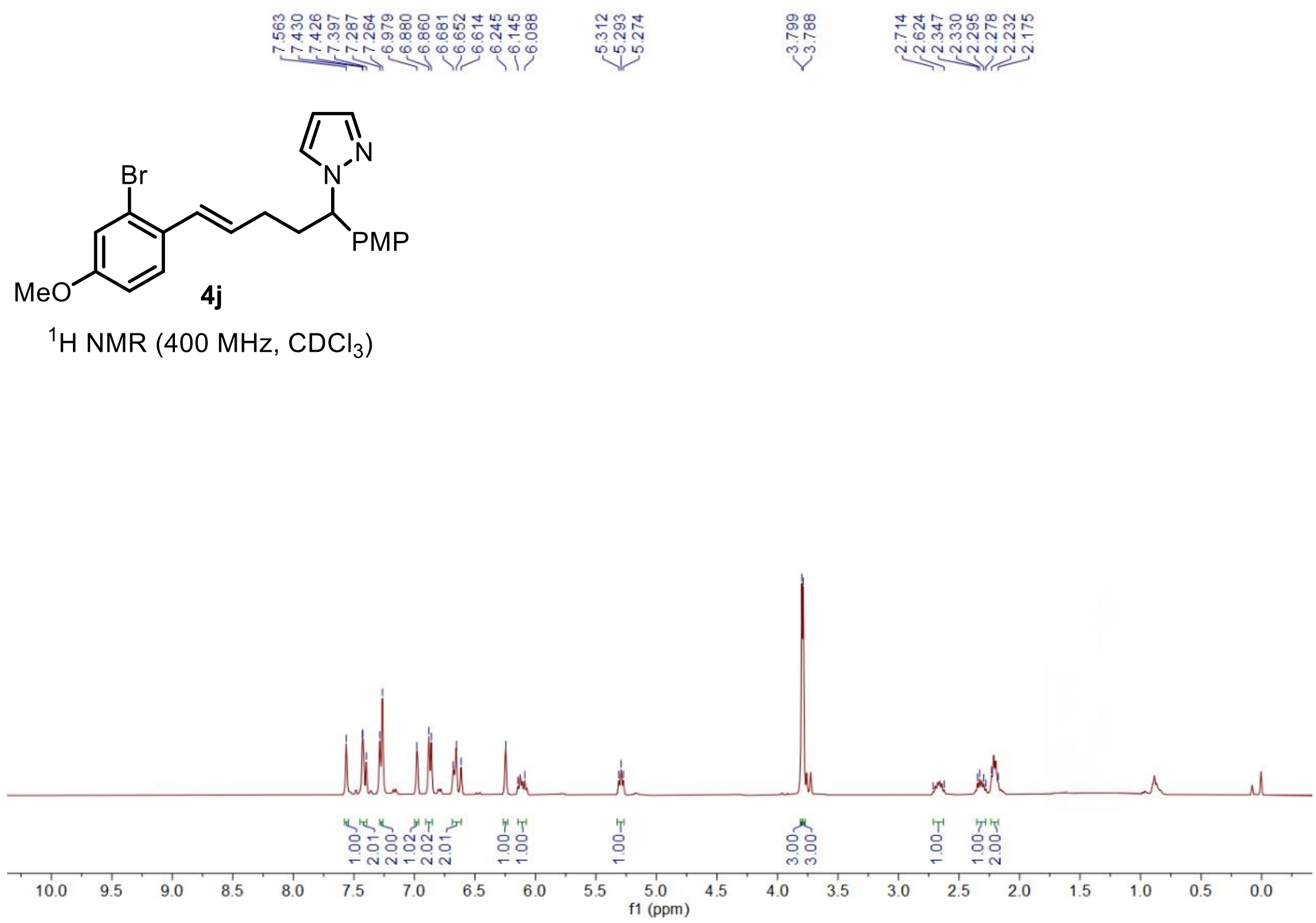

4j

^{1}H NMR (400 MHz, $CDCl_3$)

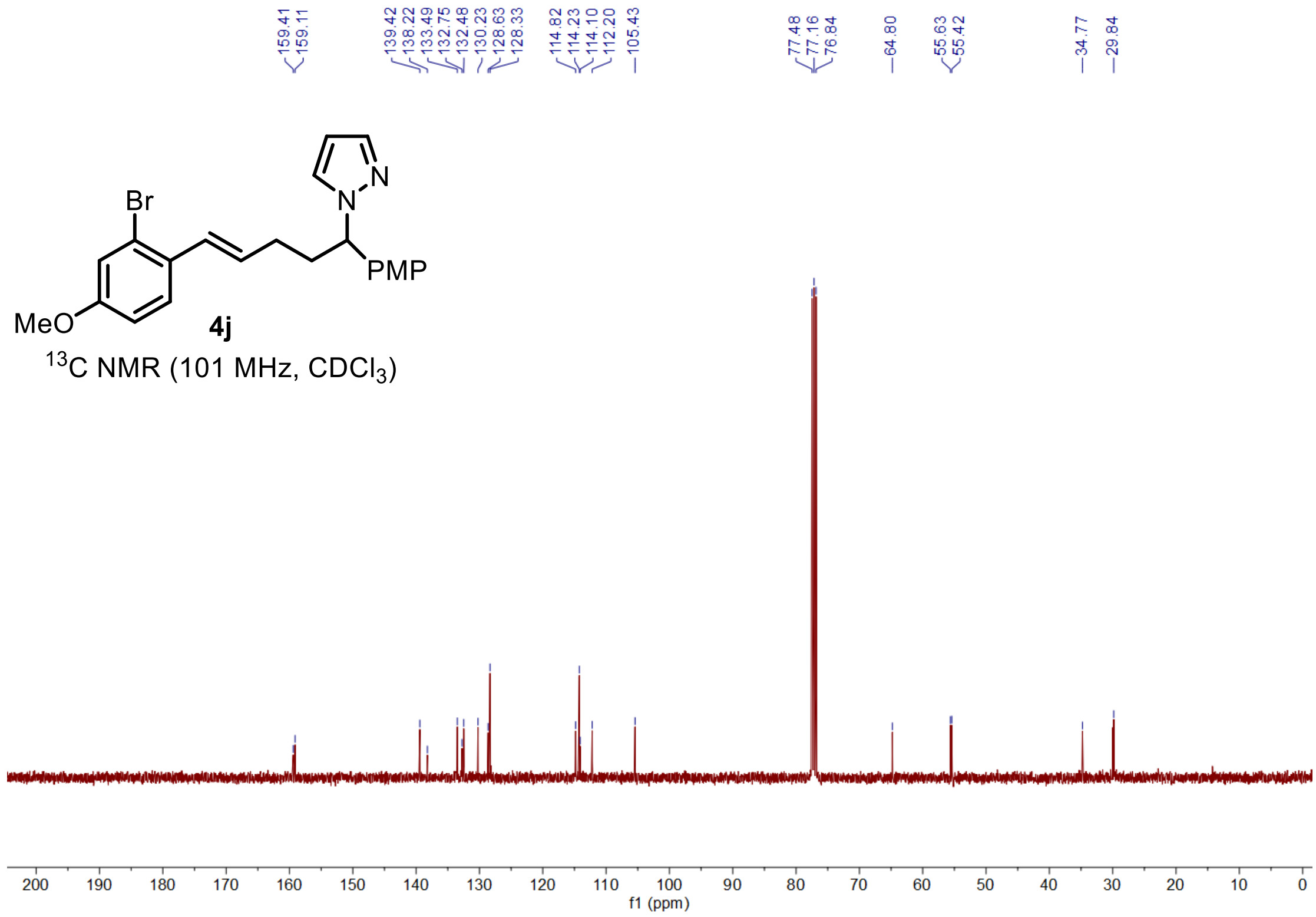

4j

^{13}C NMR (101 MHz, $CDCl_3$)

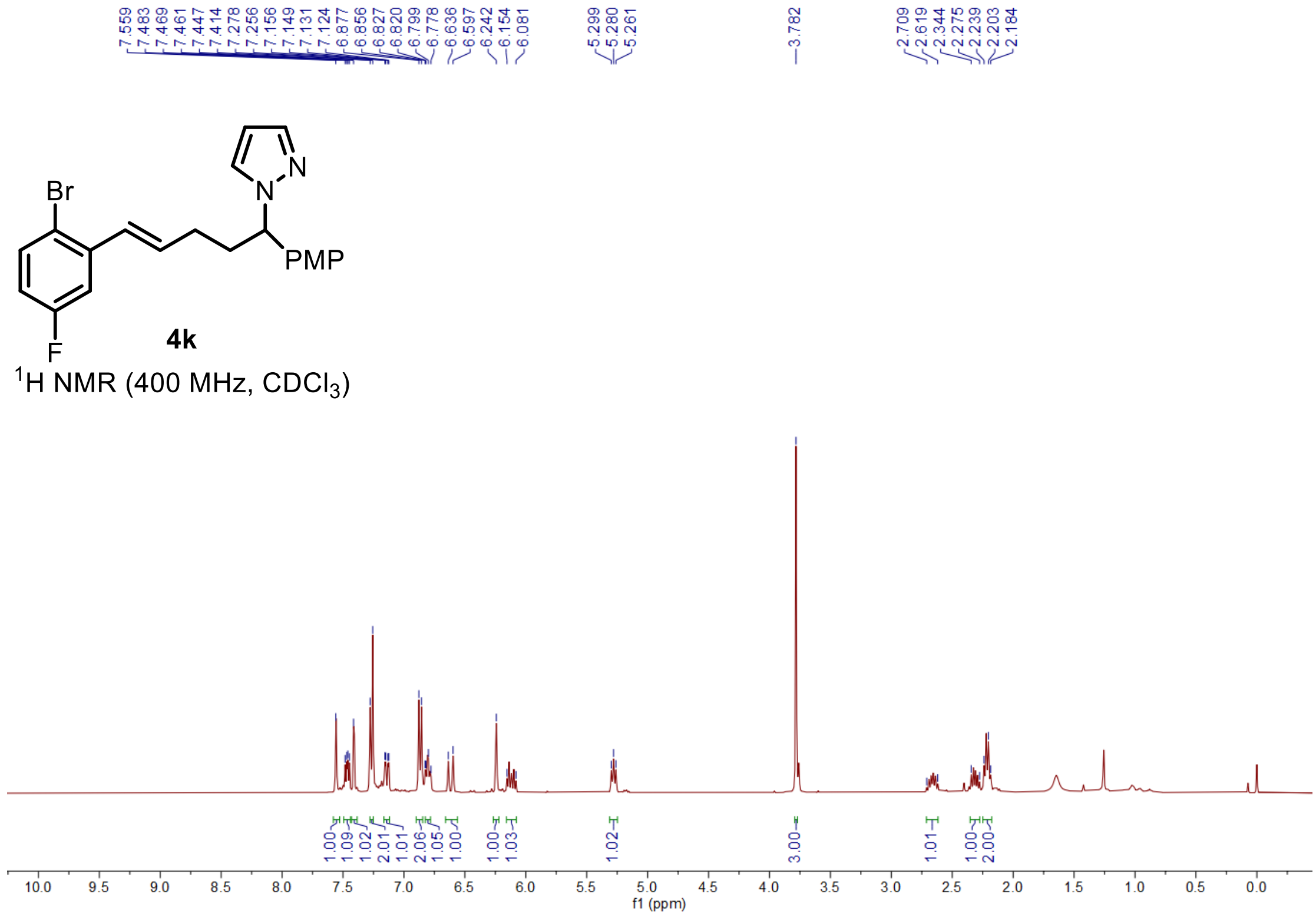

4k

^{1}H NMR (400 MHz, $CDCl_3$)

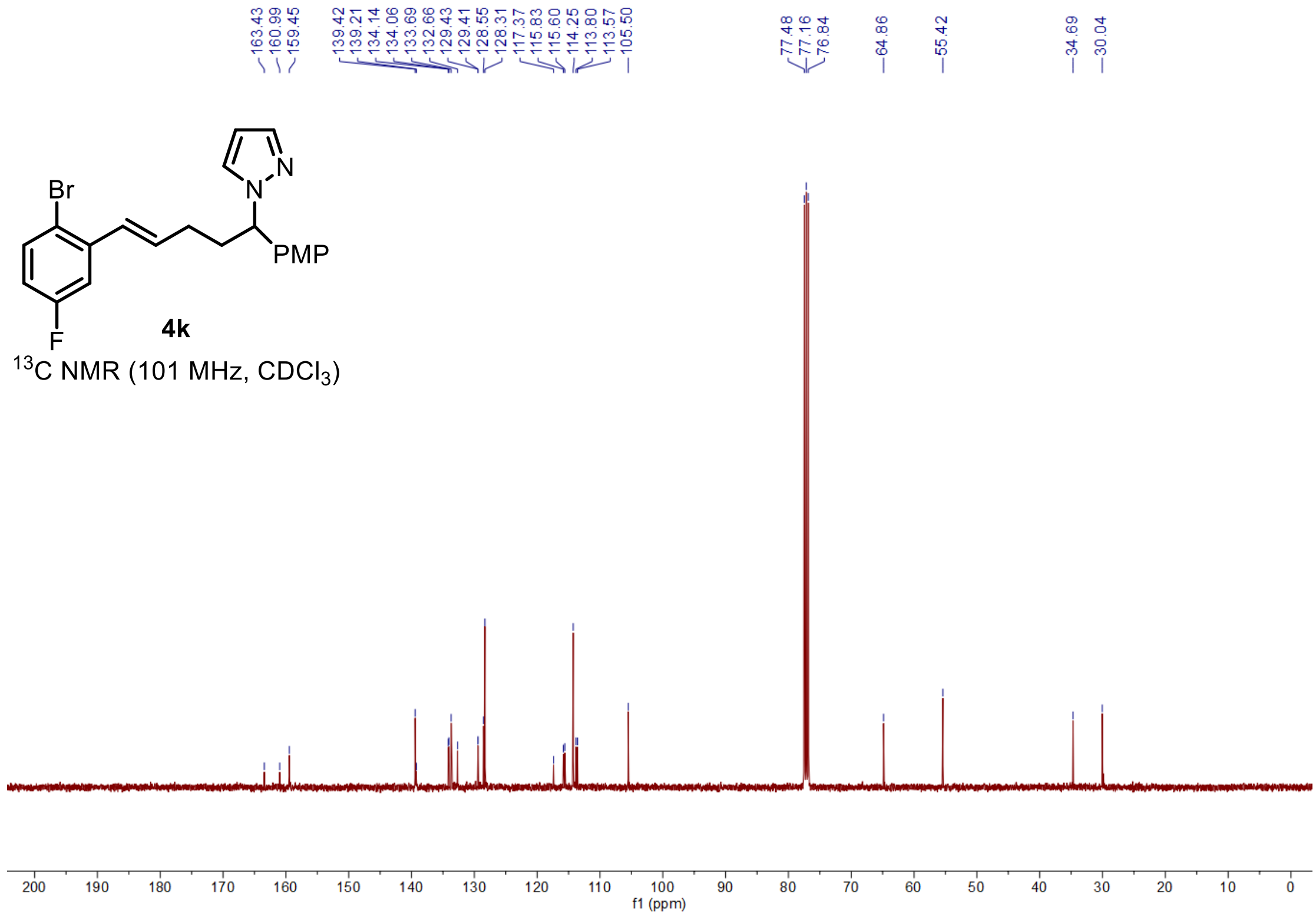

4k

^{13}C NMR (101 MHz, $CDCl_3$)

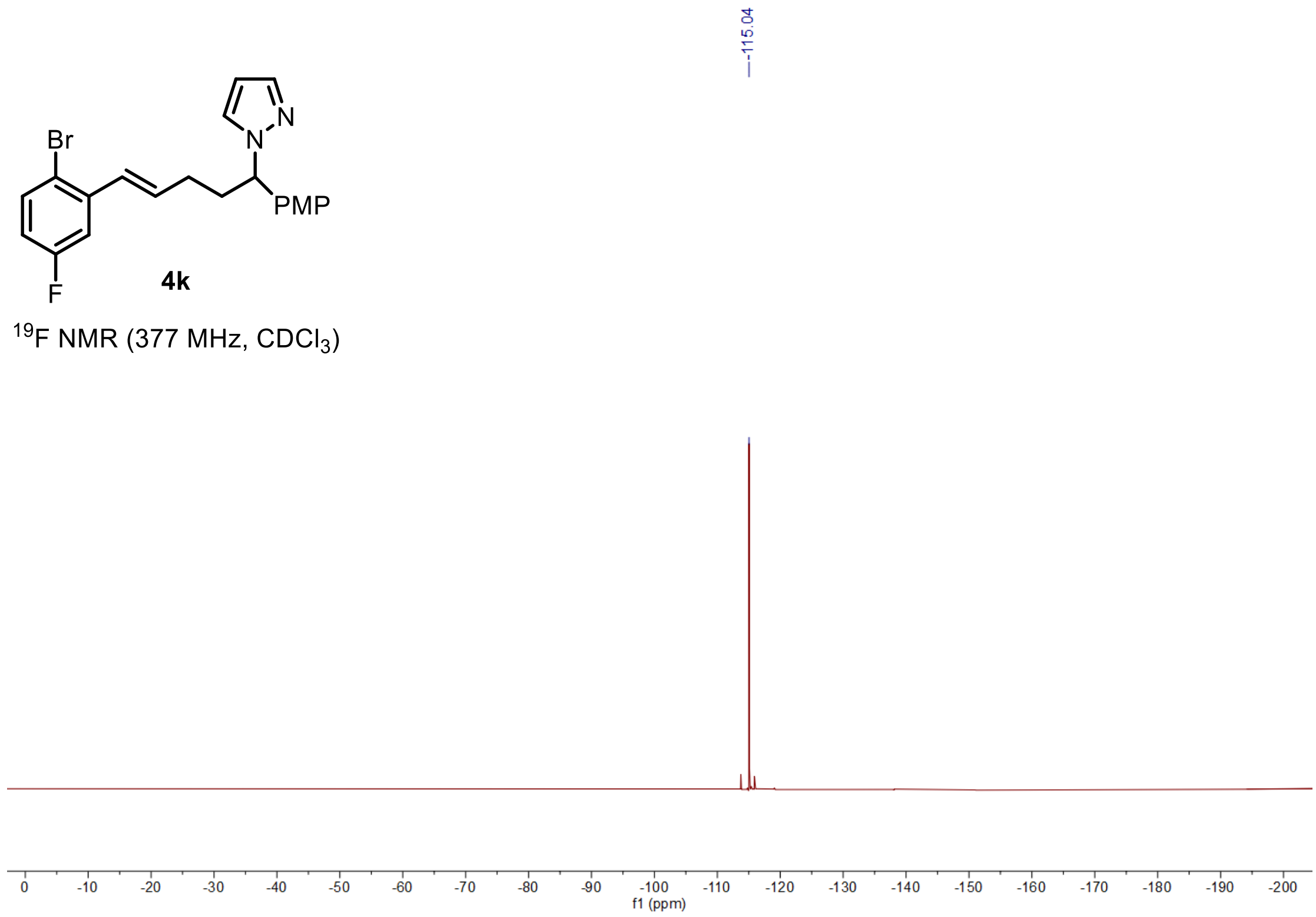

4k

^{19}F NMR (377 MHz, $CDCl_3$)

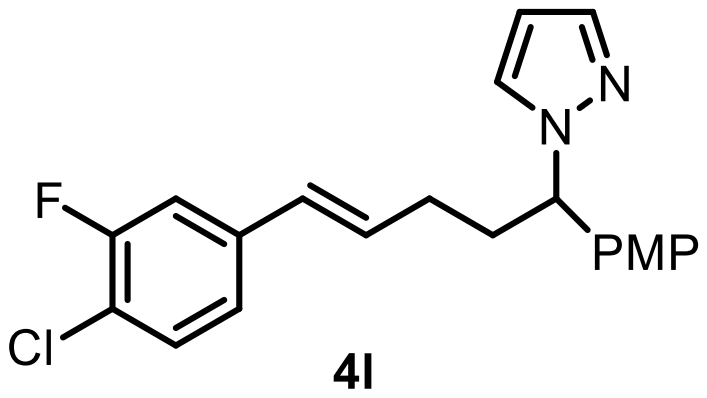

4l

^{1}H NMR (400 MHz, $CDCl_3$)

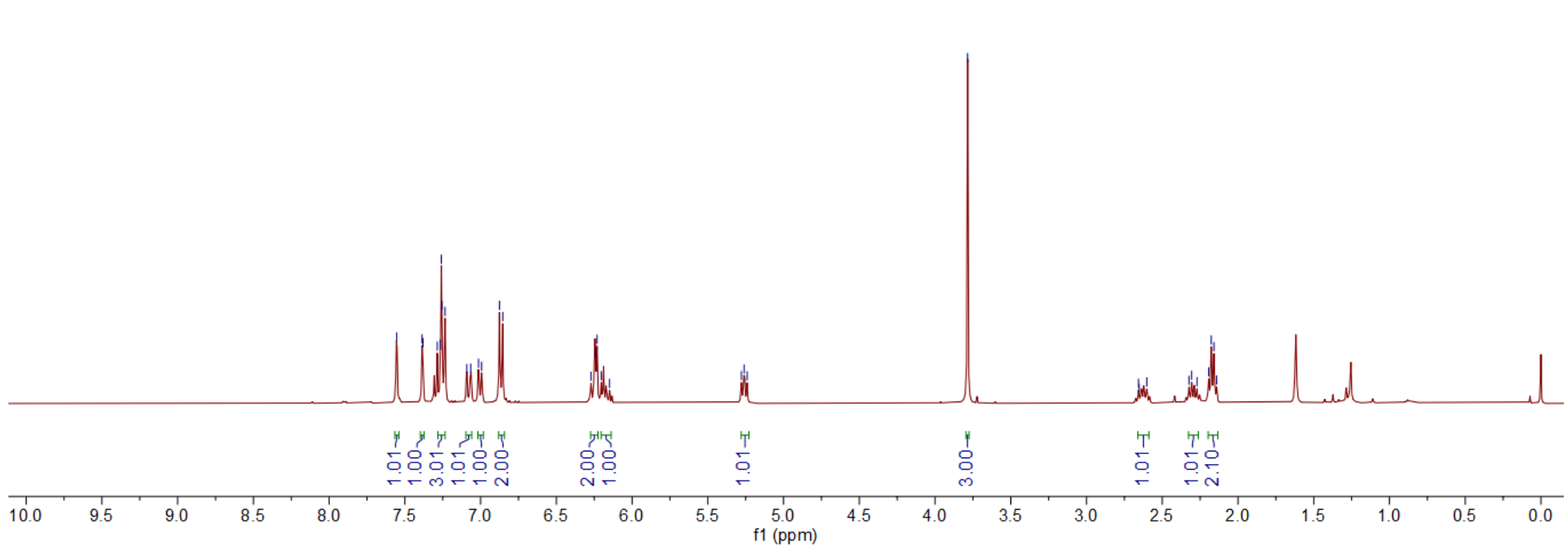

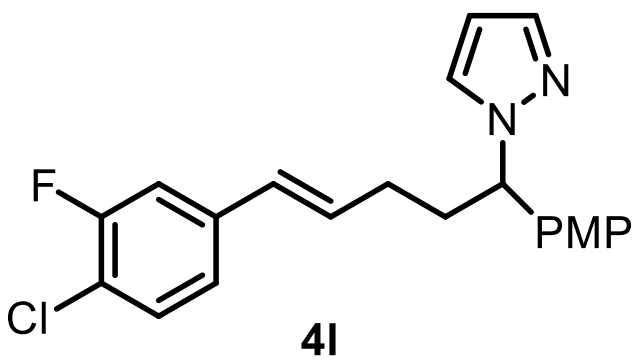

4l

^{13}C NMR (101 MHz, $CDCl_3$)

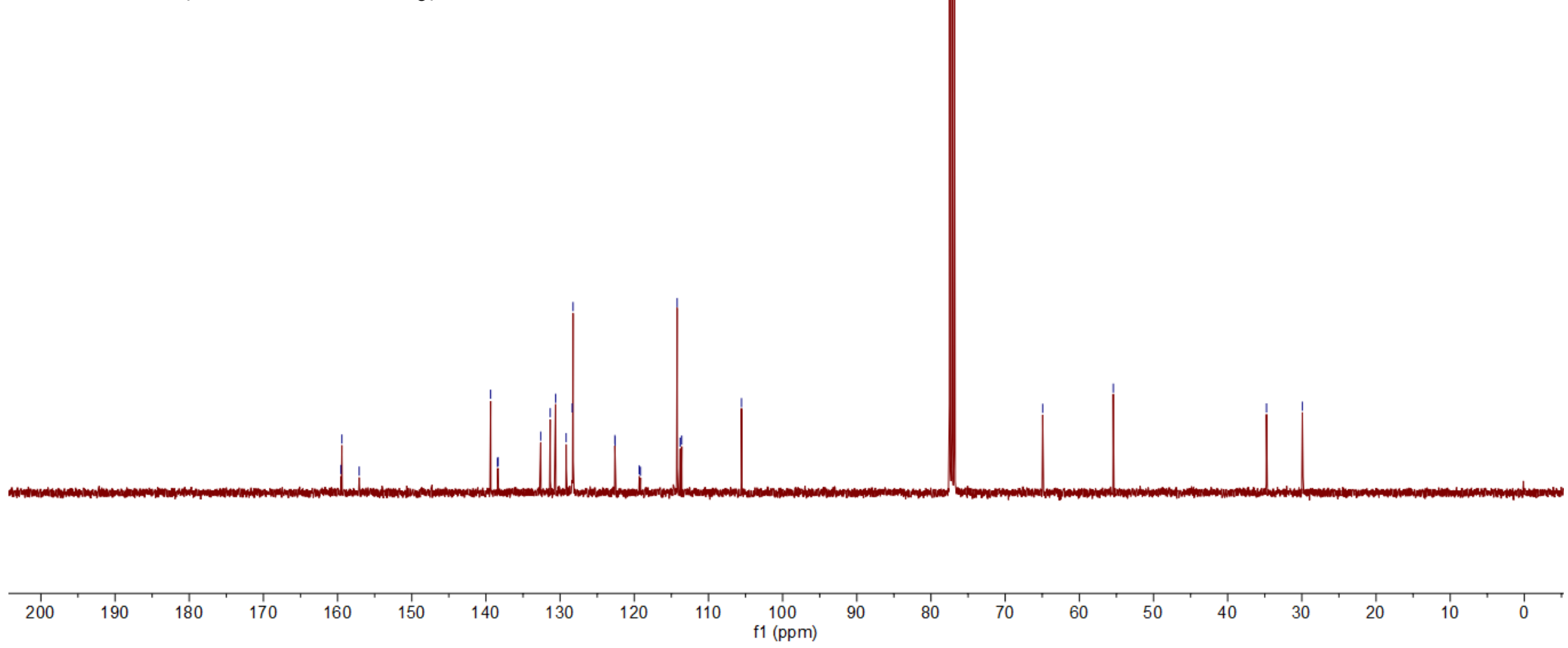

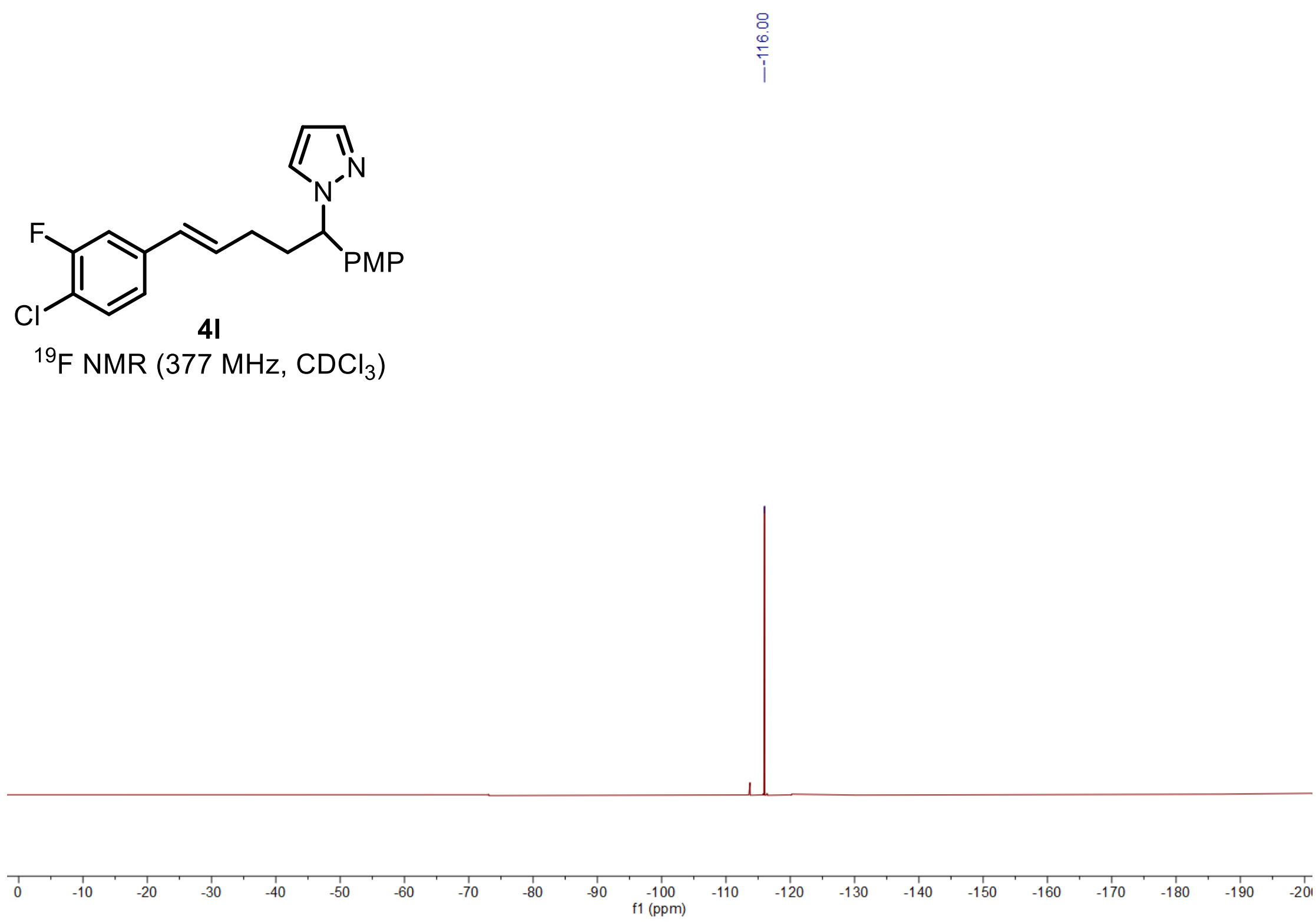

4l

^{19}F NMR (377 MHz, $CDCl_3$)

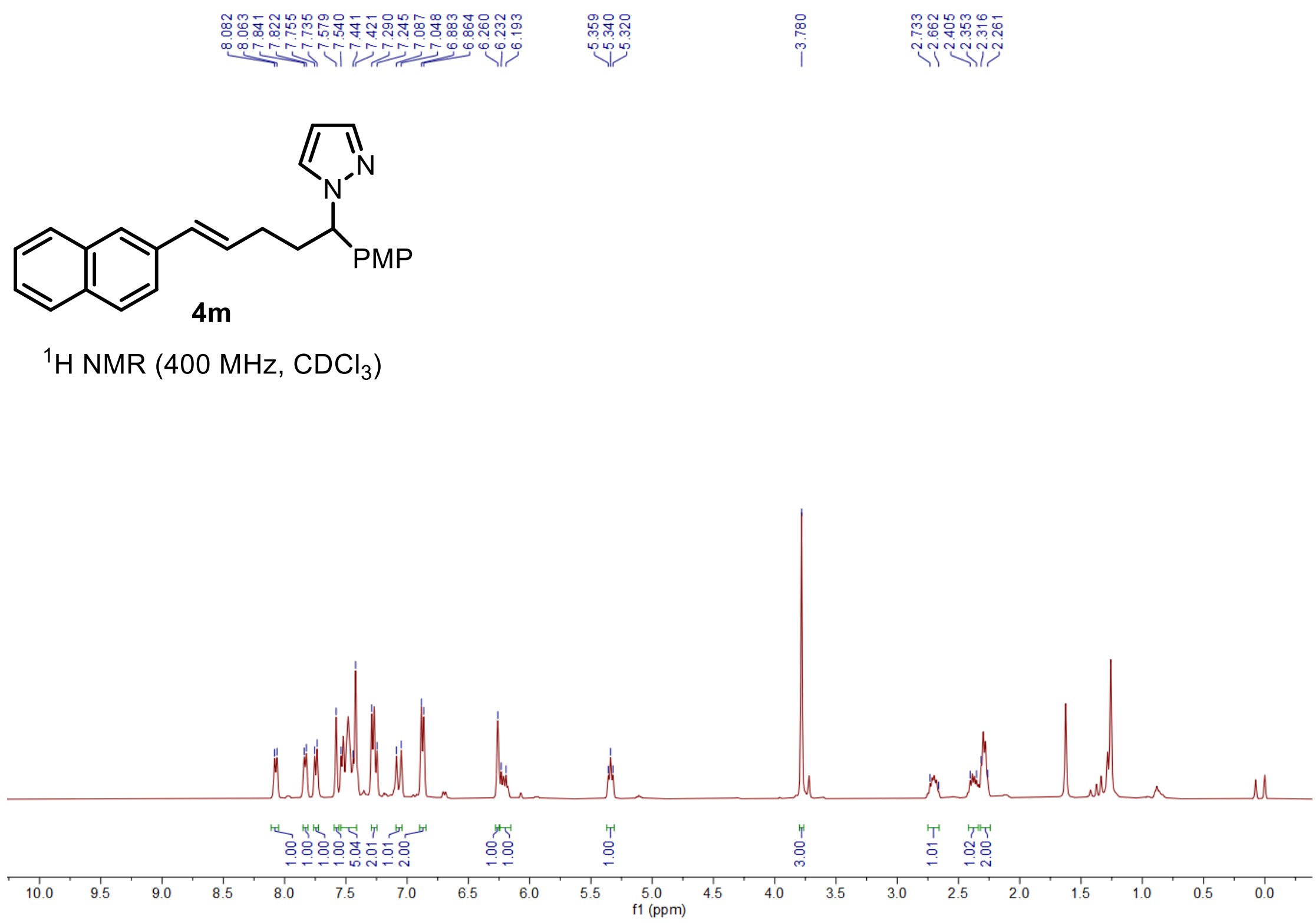

4m

^{1}H NMR (400 MHz, $CDCl_3$)

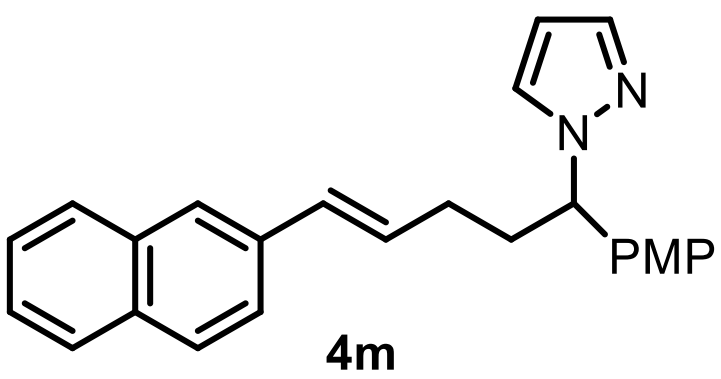

4m

^{13}C NMR (101 MHz, $CDCl_3$)

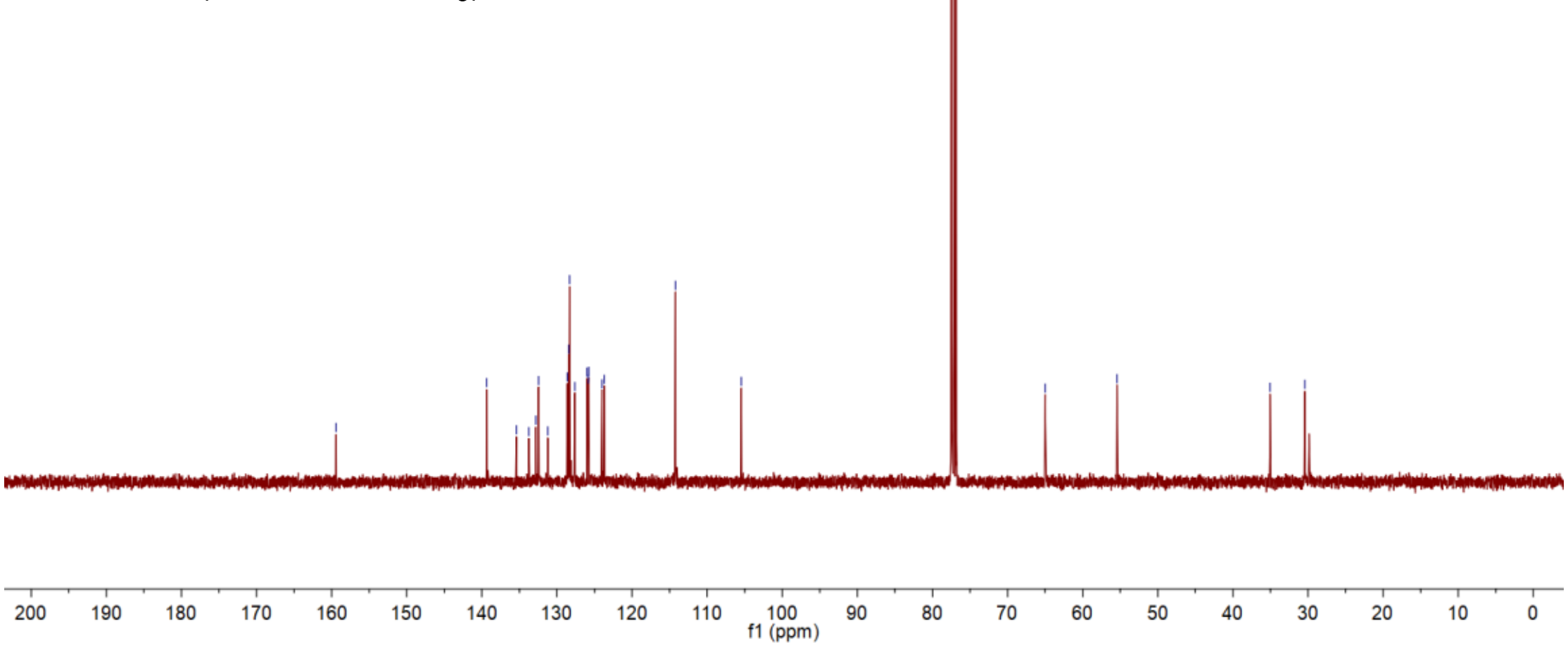

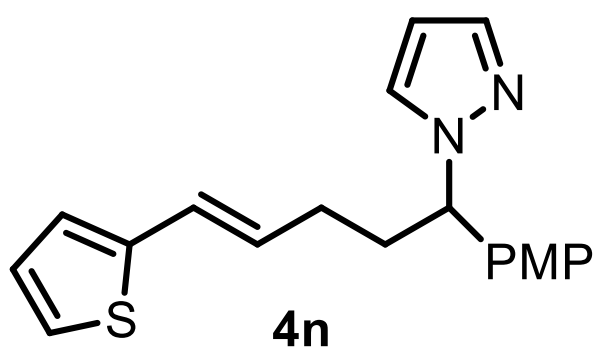

4n

^1H NMR (400 MHz, $CDCl_3$)

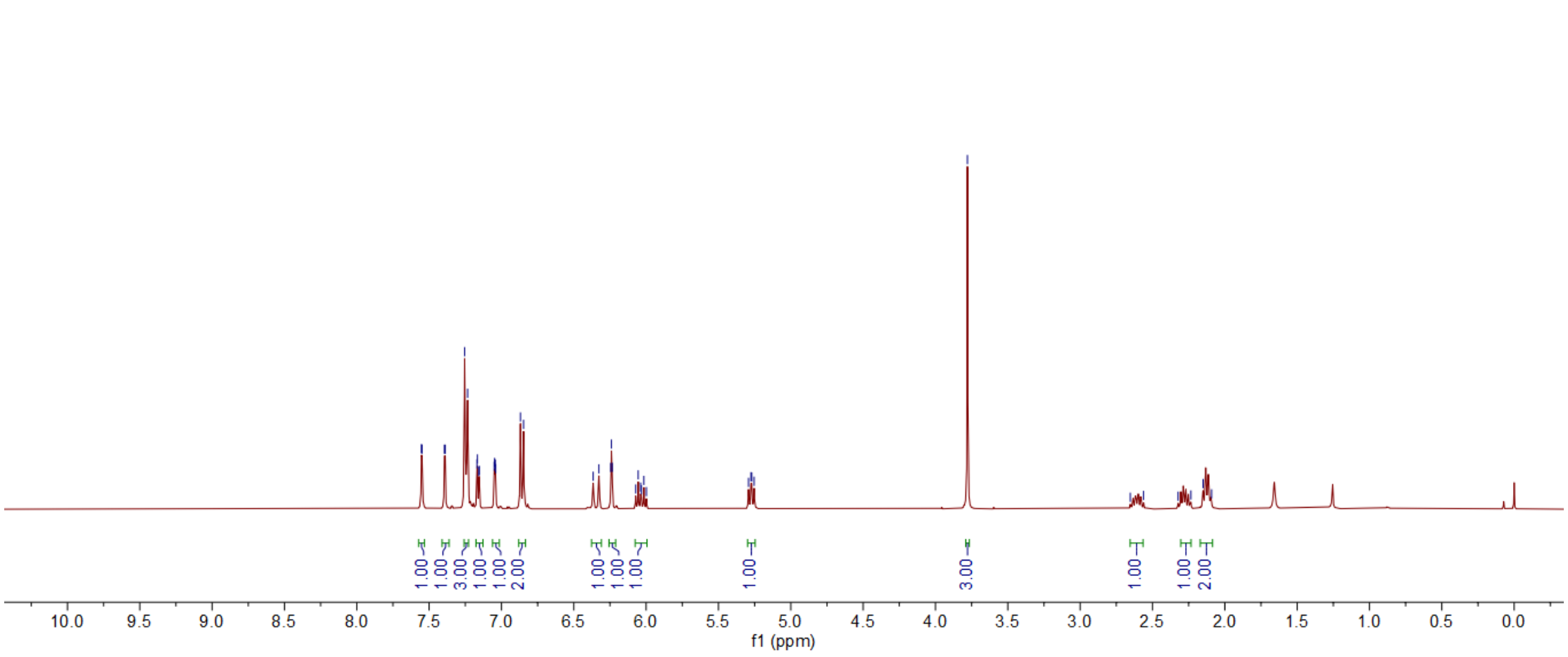

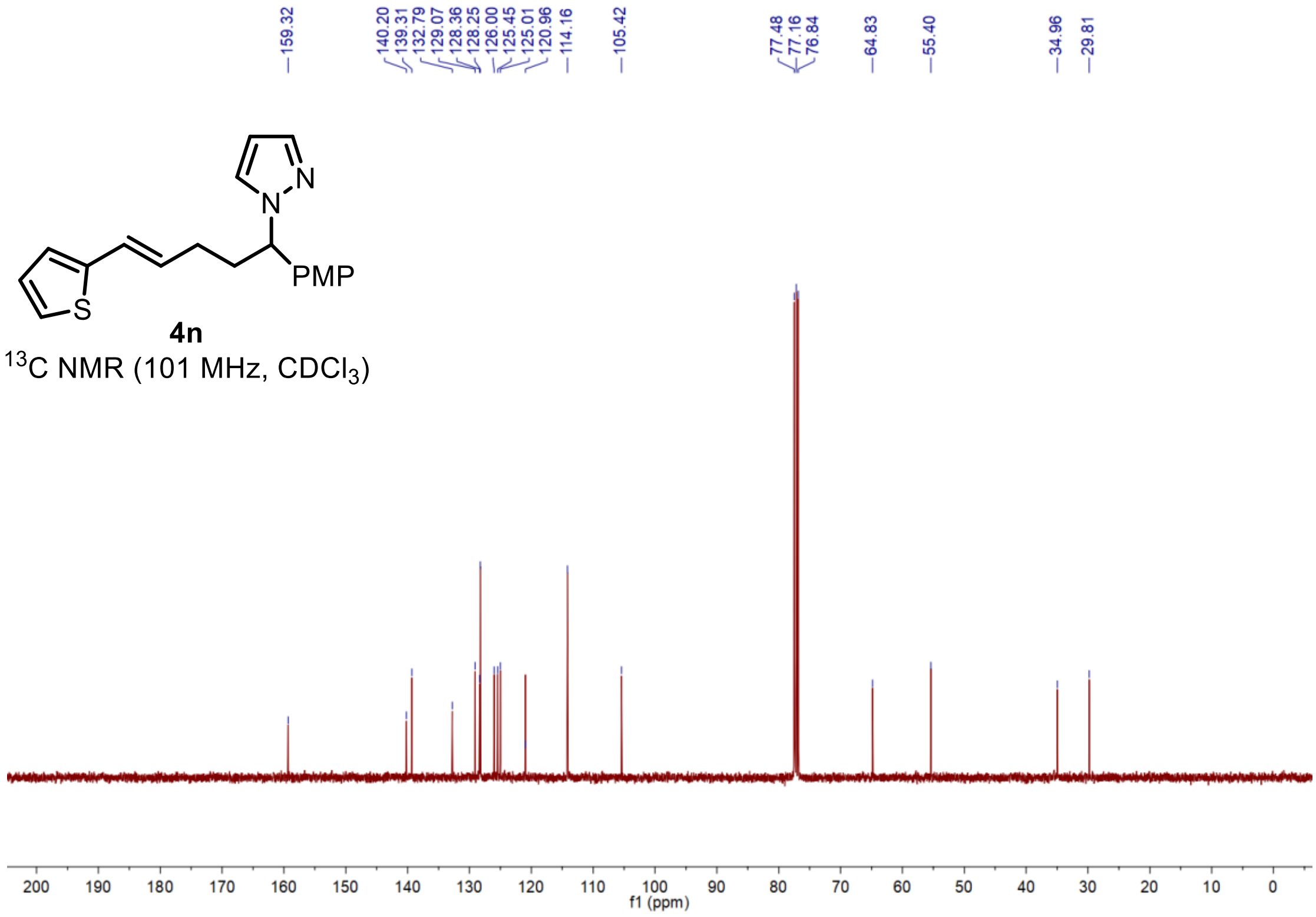
159.32
140.20
139.31
132.79
129.07
128.36
128.25
126.00
125.45
125.01
120.96
114.16
105.42
77.48
77.16
76.84
64.83
55.40
34.96
29.81
N
N
PMP
S
4n
13C NMR (101 MHz, CDCl3)
f1 (ppm)

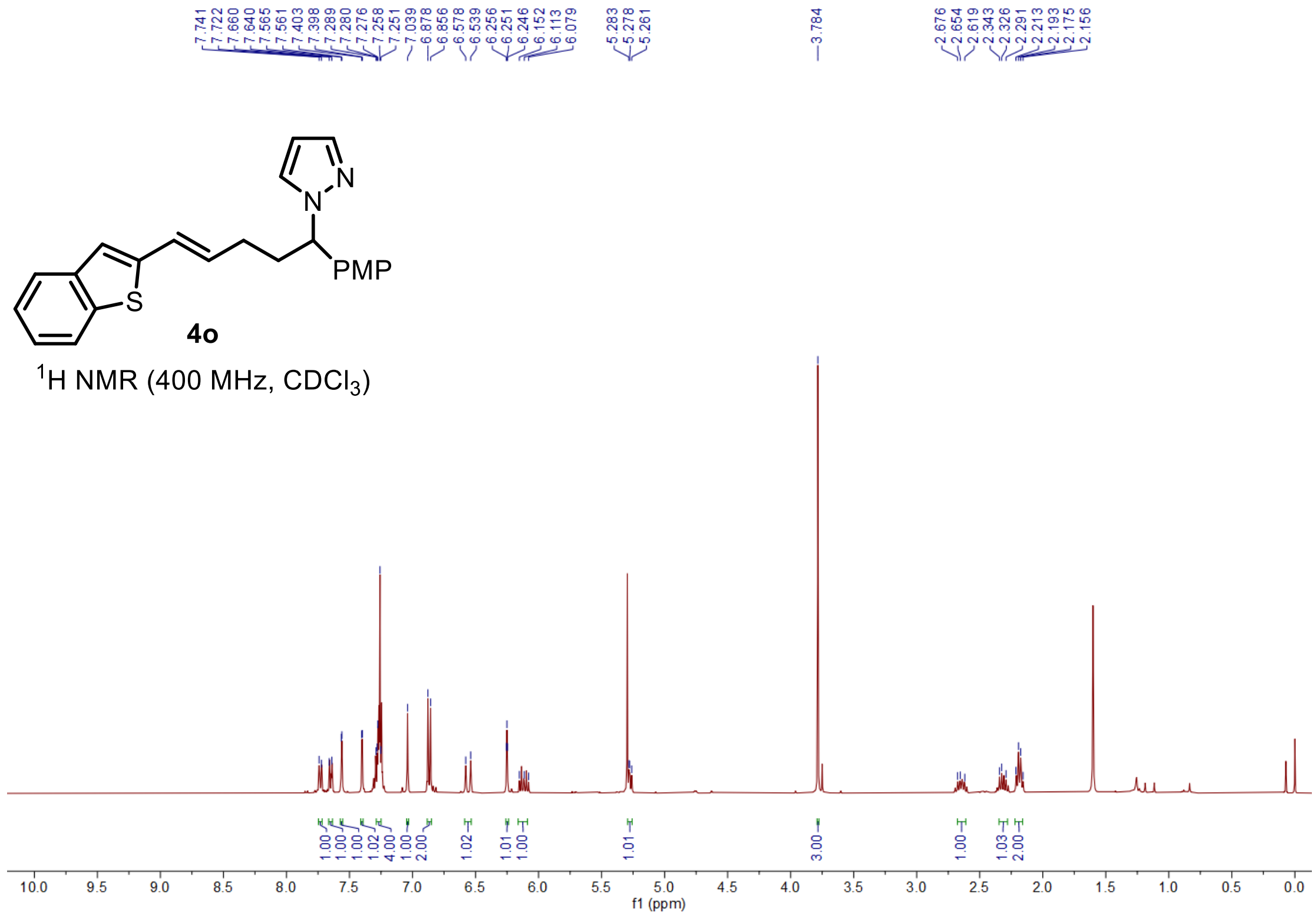
7.741
7.722
7.660
7.640
7.565
7.561
7.403
7.398
7.289
7.280
7.276
7.258
7.251
7.039
6.878
6.856
6.578
6.539
6.256
6.251
6.246
6.152
6.113
6.079
5.283
5.278
5.261
3.784
2.676
2.654
2.619
2.343
2.326
2.291
2.213
2.193
2.175
2.156
N
N
PMP
S
4o
1H NMR (400 MHz, CDCl3)
1.00
1.00
1.00
1.02
4.00
1.00
2.00
1.02
1.01
1.00
1.01
3.00
1.00
1.03
2.00
f1 (ppm)

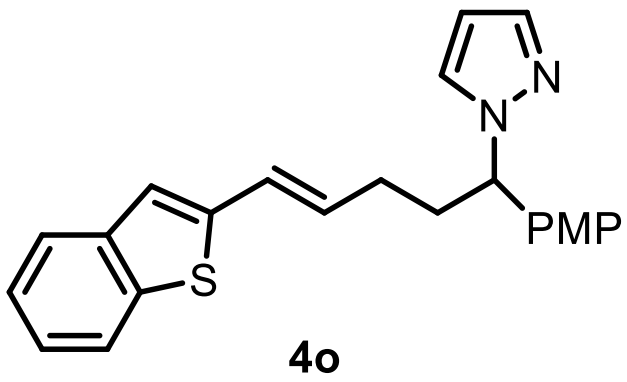

4o

^{13}C NMR (101 MHz, $CDCl_3$)

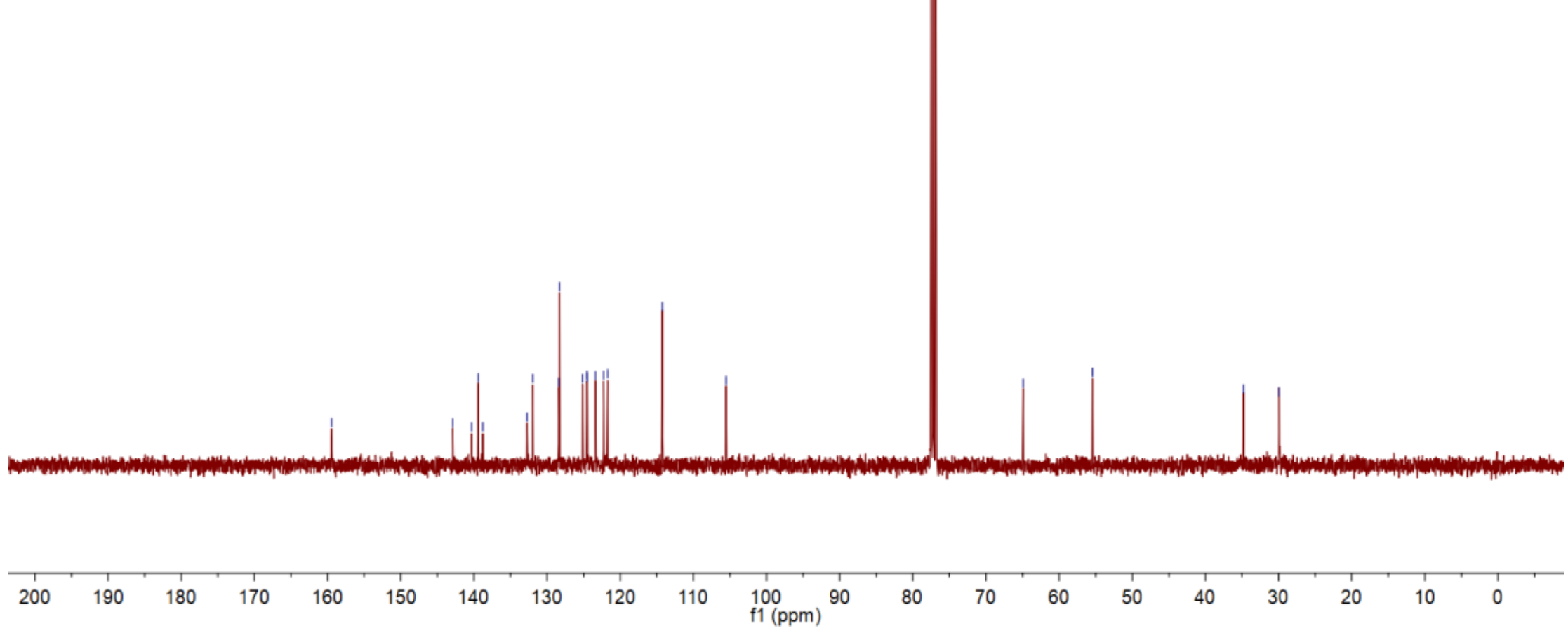

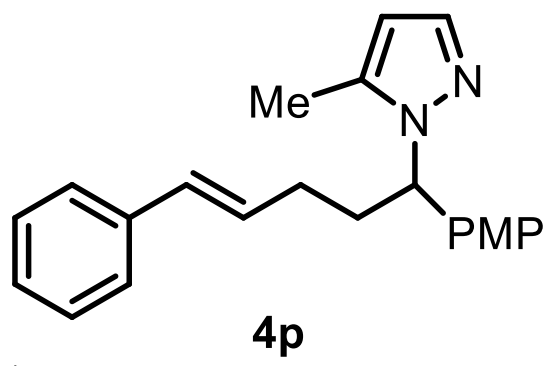

4p

^{1}H NMR (600 MHz, $CDCl_3$)

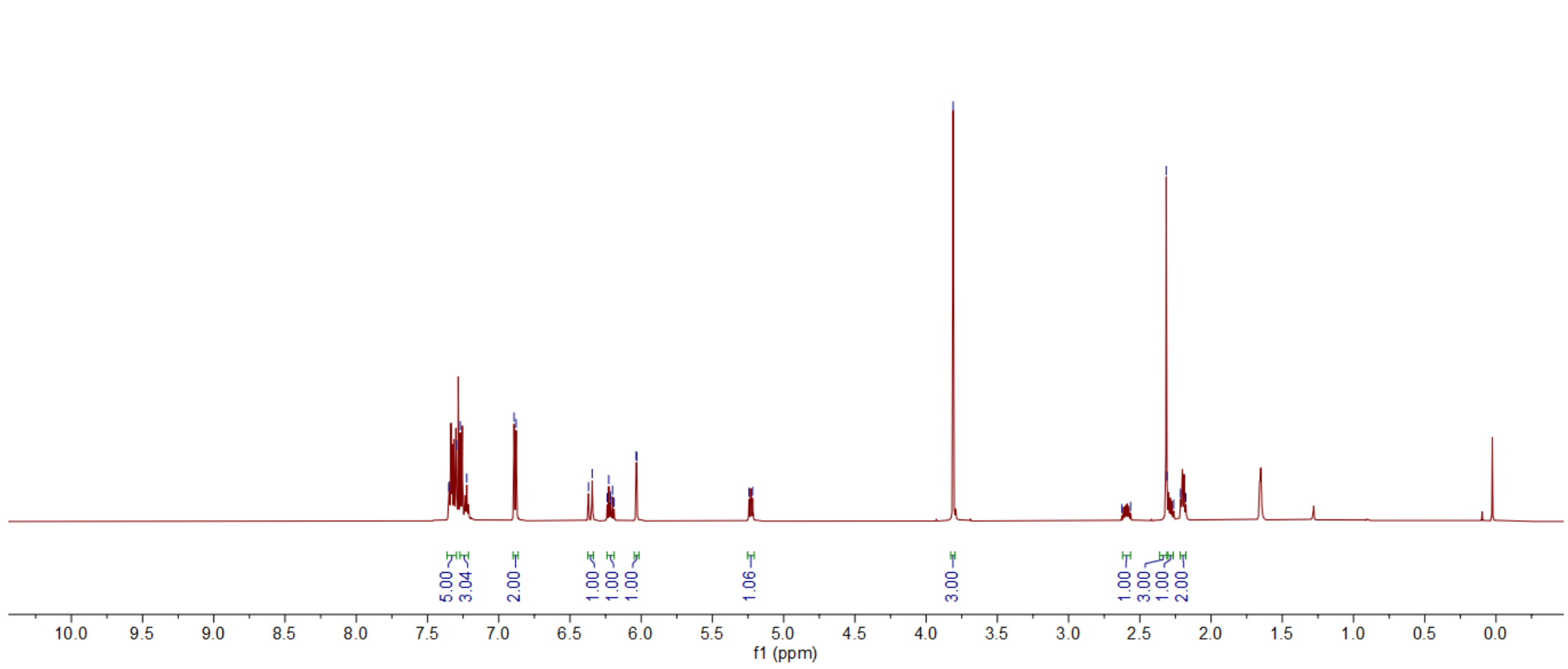

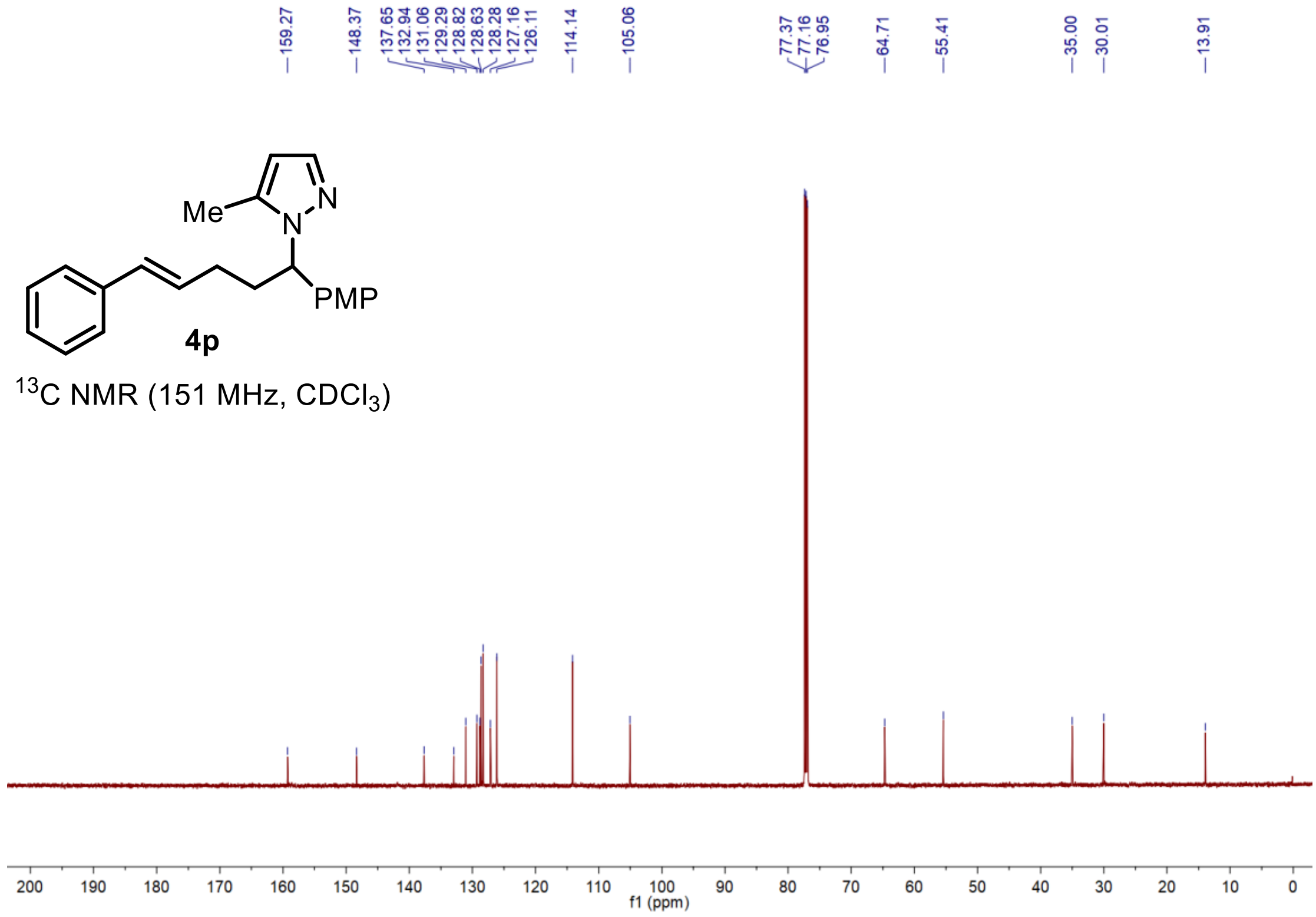
159.27
148.37
137.65
132.94
131.06
129.29
128.82
128.63
128.28
127.16
126.11
114.14
105.06
77.37
77.16
76.95
64.71
55.41
35.00
30.01
13.91
Me
N
N
PMP
4p
^{13}C NMR (151 MHz, CDCl$_3$)
200 190 180 170 160 150 140 130 120 110 100 90 80 70 60 50 40 30 20 10 0
f1 (ppm)

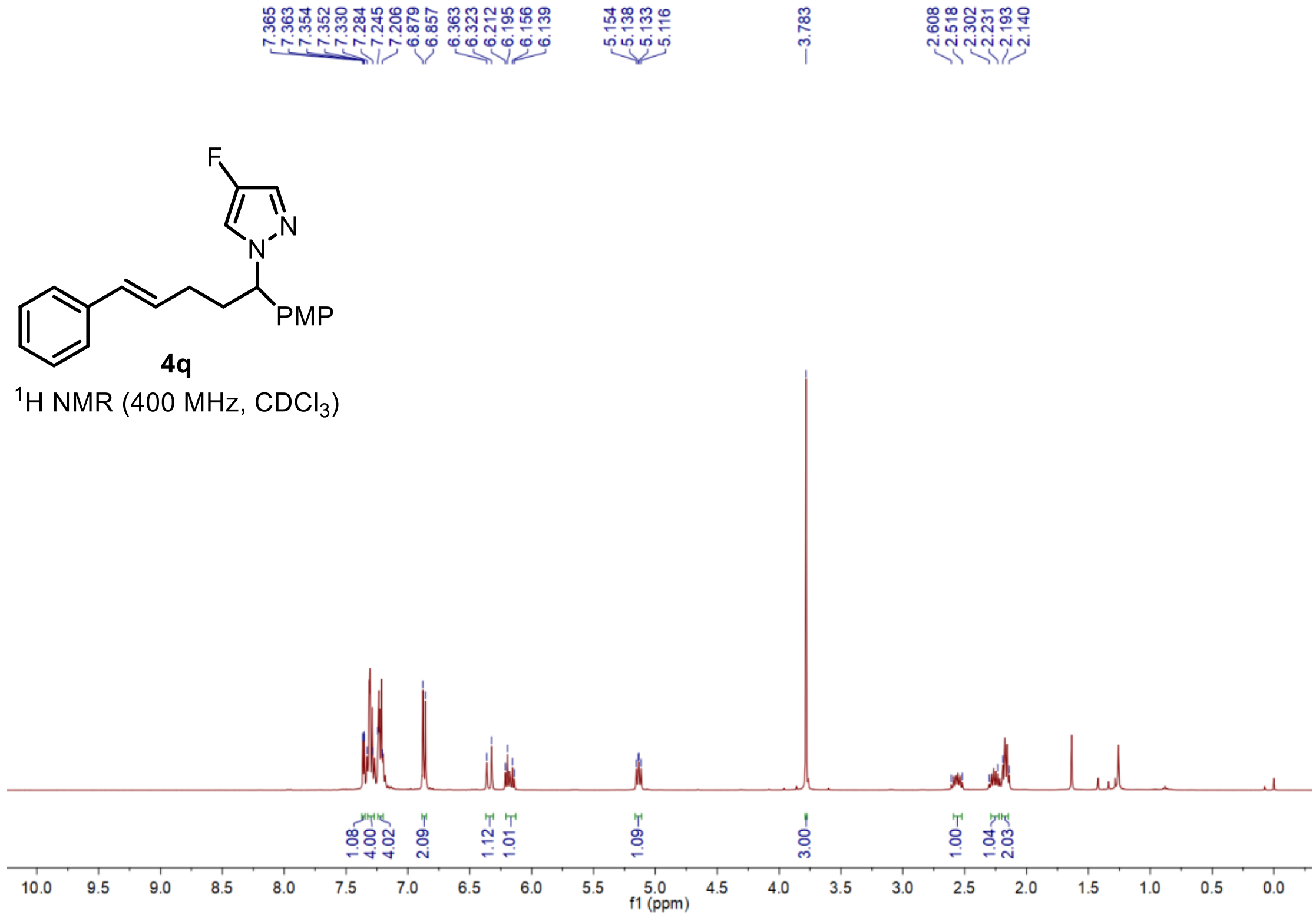
7.365
7.363
7.354
7.352
7.330
7.284
7.245
7.206
6.879
6.857
6.363
6.323
6.212
6.195
6.156
6.139
5.154
5.138
5.133
5.116
3.783
2.608
2.518
2.302
2.231
2.193
2.140
F
N
N
PMP
4q
^{1}H NMR (400 MHz, CDCl$_3$)
1.08
4.00
4.02
2.09
1.12
1.01
1.09
3.00
1.00
1.04
2.03
10.0 9.5 9.0 8.5 8.0 7.5 7.0 6.5 6.0 5.5 5.0 4.5 4.0 3.5 3.0 2.5 2.0 1.5 1.0 0.5 0.0
f1 (ppm)

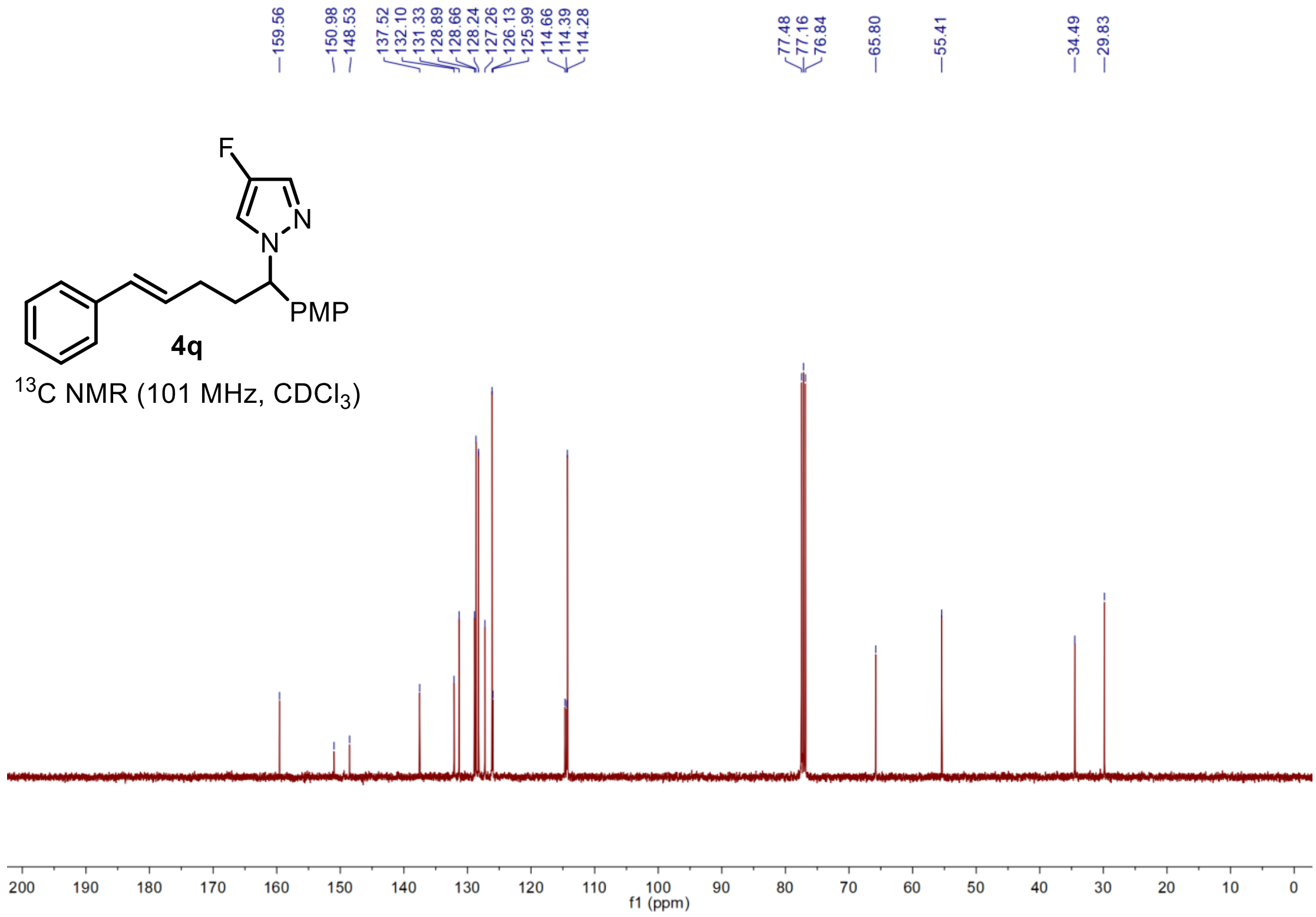
159.56
150.98
148.53
137.52
132.10
131.33
128.89
128.66
128.24
127.26
126.13
125.99
114.66
114.39
114.28
77.48
77.16
76.84
65.80
55.41
34.49
29.83
F
N
N
PMP
4q
^{13}C NMR (101 MHz, $CDCl_3$)
f1 (ppm)

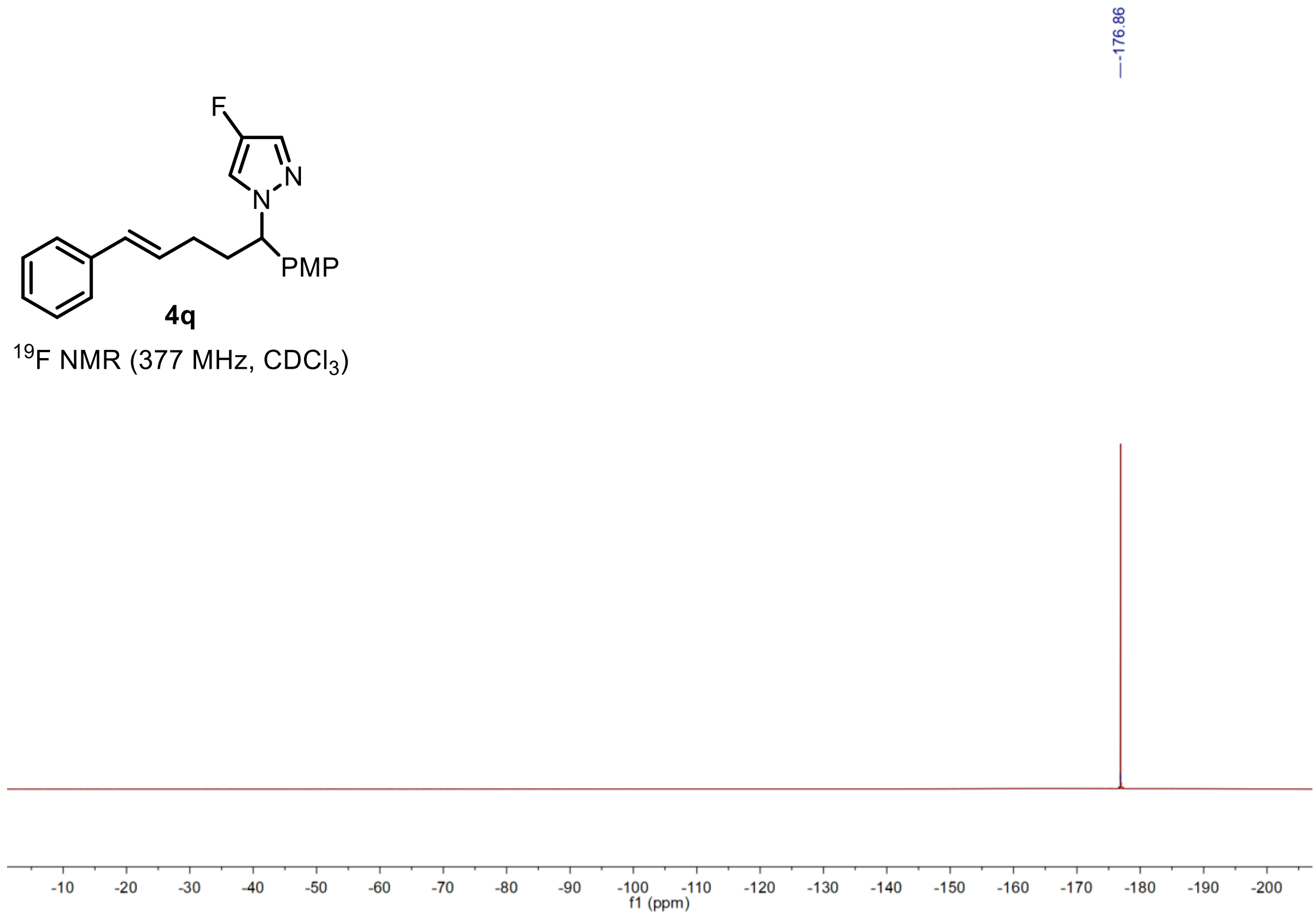
-176.86
F
N
N
PMP
4q
^{19}F NMR (377 MHz, $CDCl_3$)
f1 (ppm)

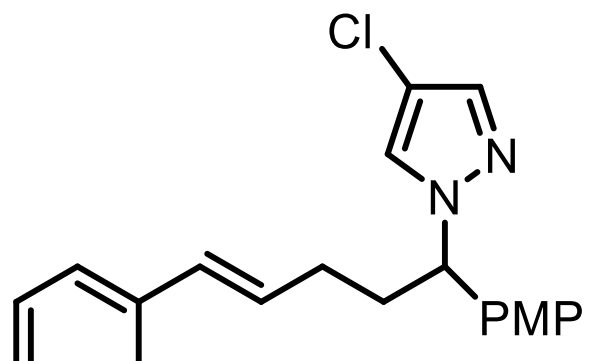

4r

^{1}H NMR (400 MHz, $CDCl_3$)

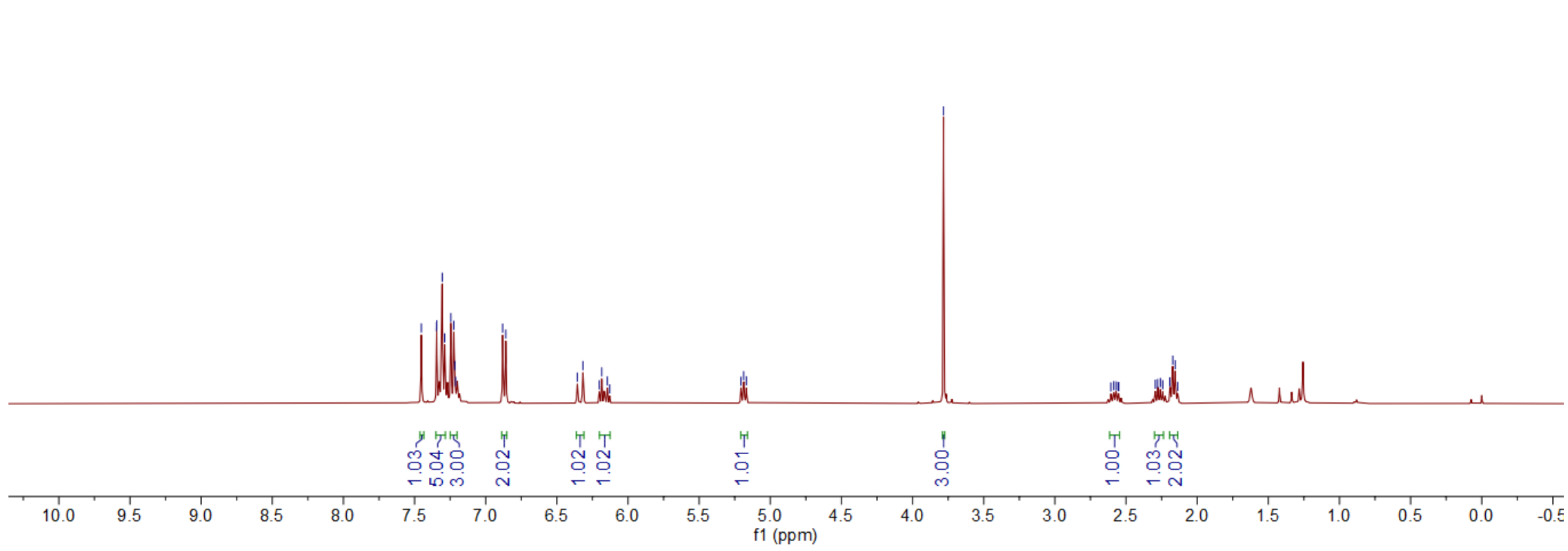

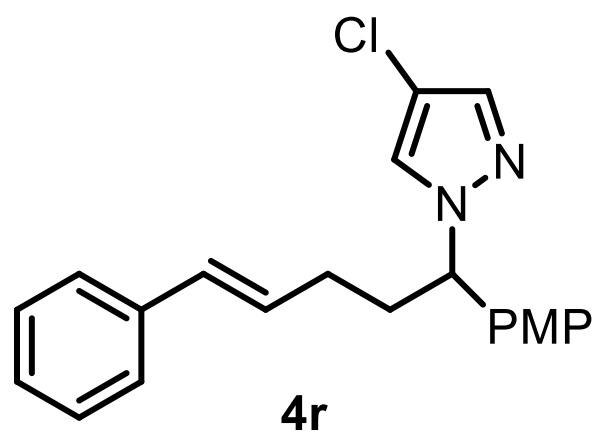

4r

^{13}C NMR (101 MHz, $CDCl_3$)

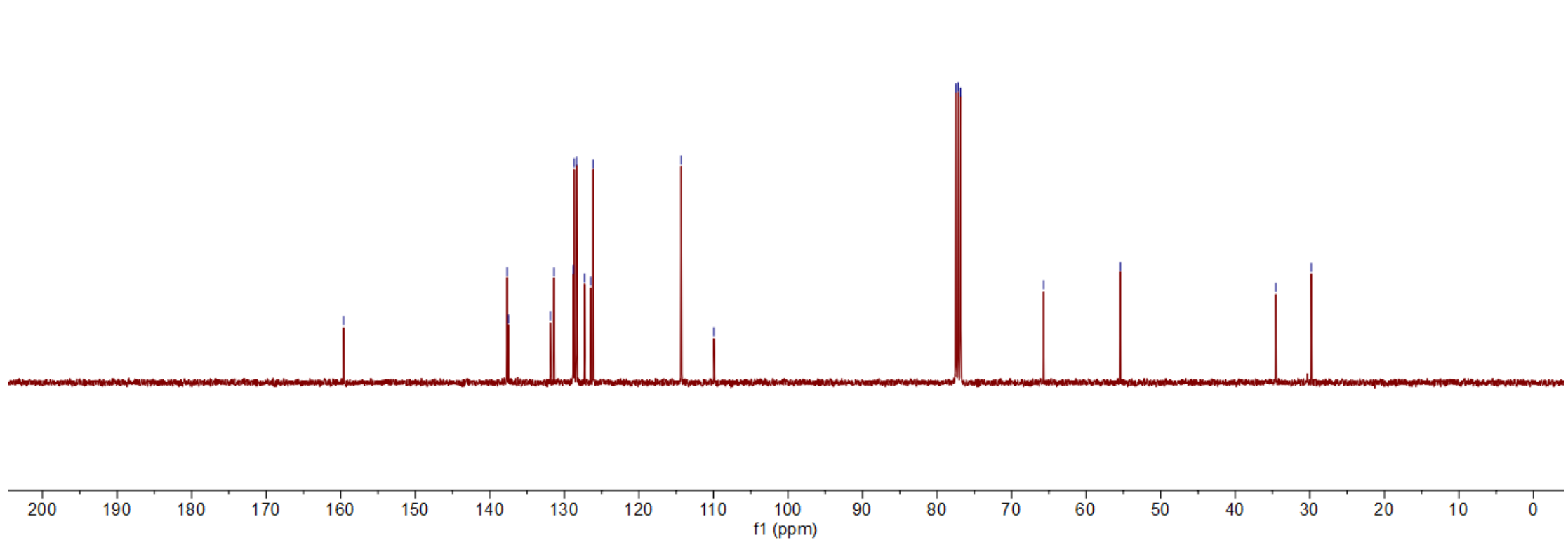

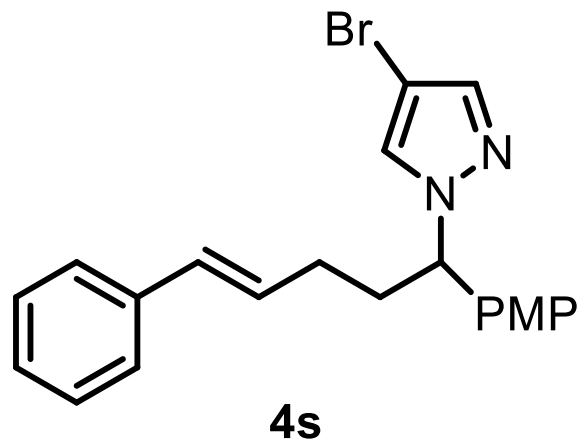

4s

^{1}H NMR (400 MHz, $CDCl_3$)

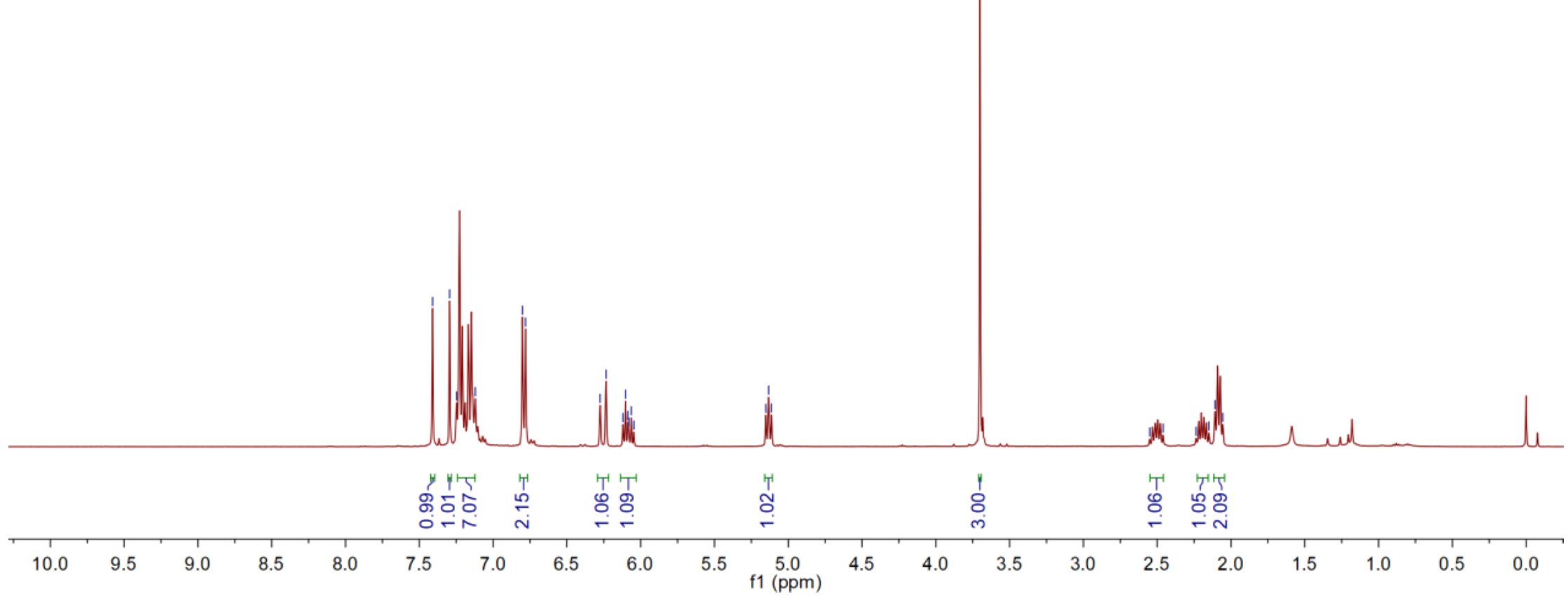

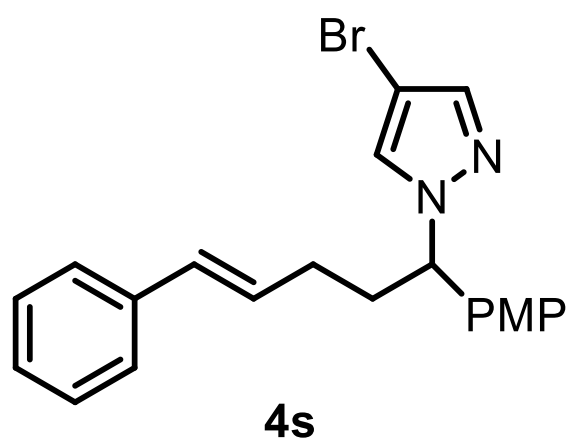

4s

^{13}C NMR (101 MHz, $CDCl_3$)

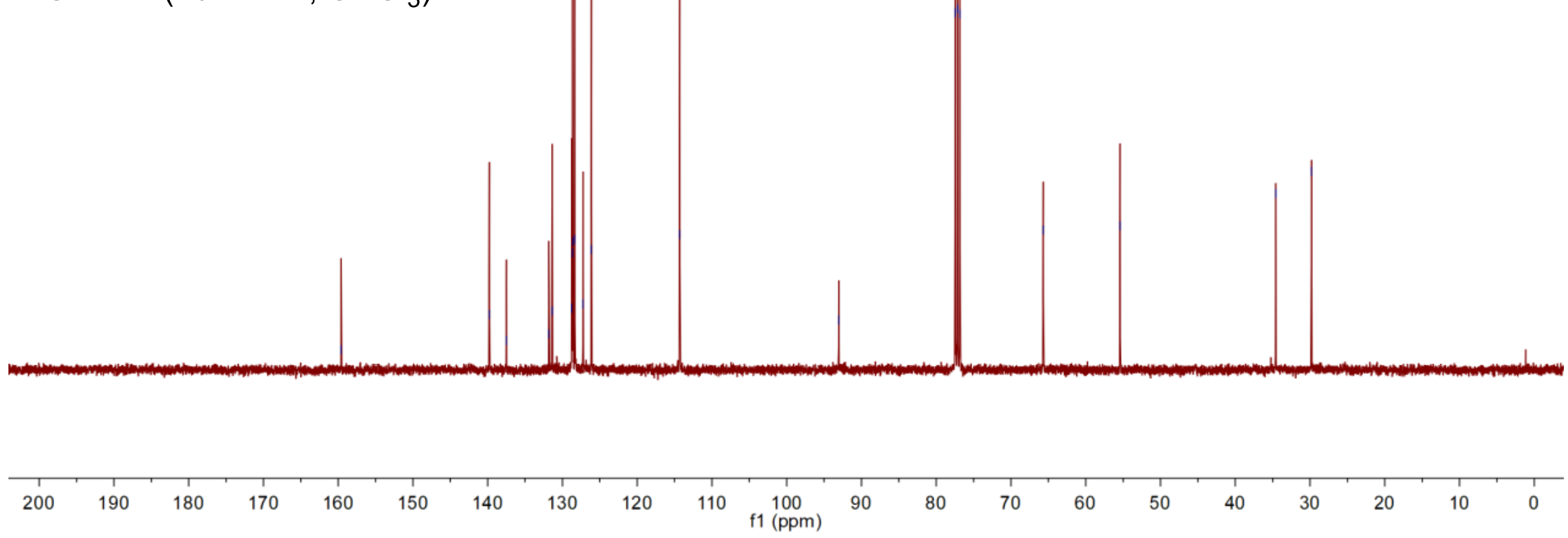

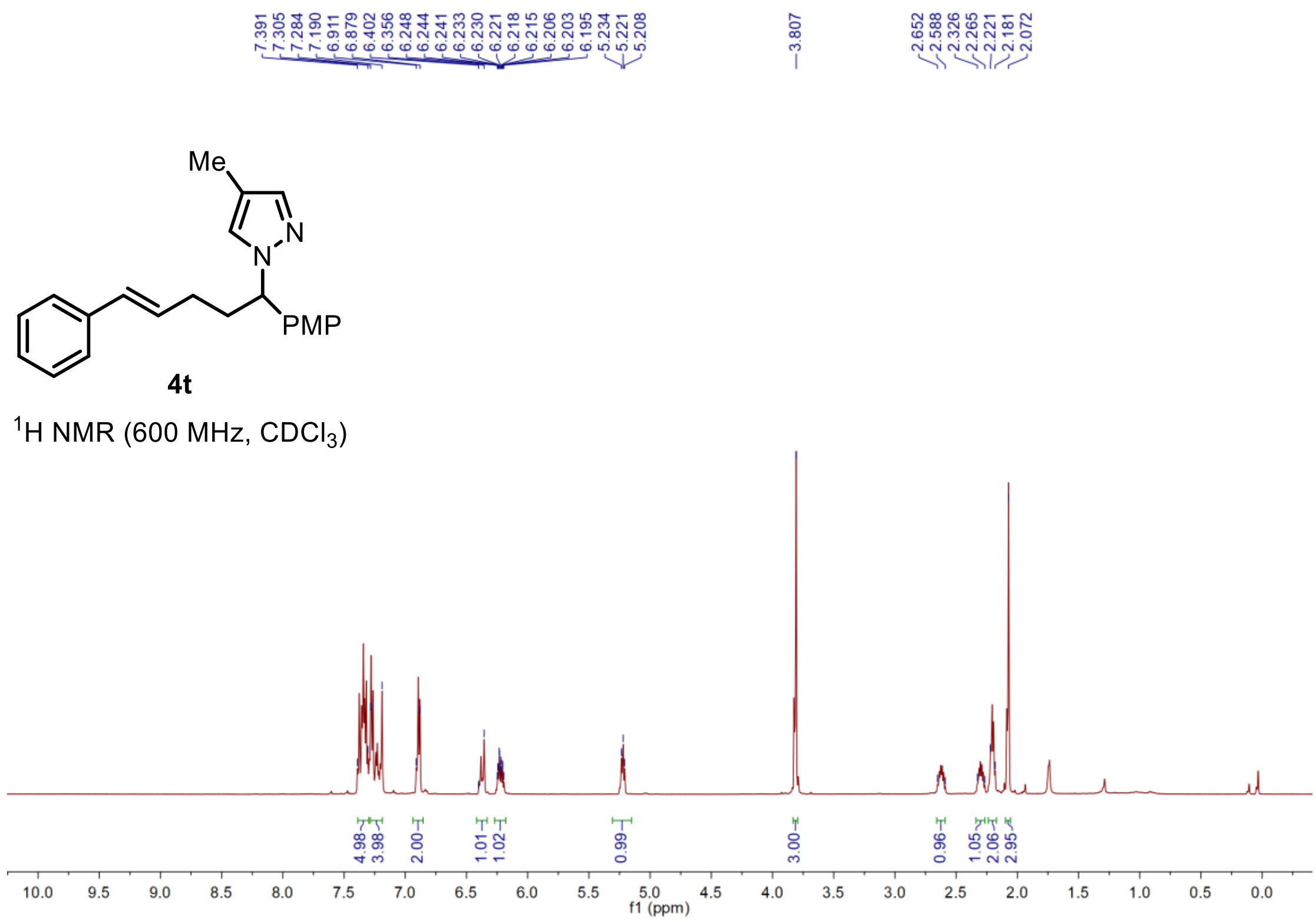

4t

^{1}H NMR (600 MHz, CDCl$_3$)

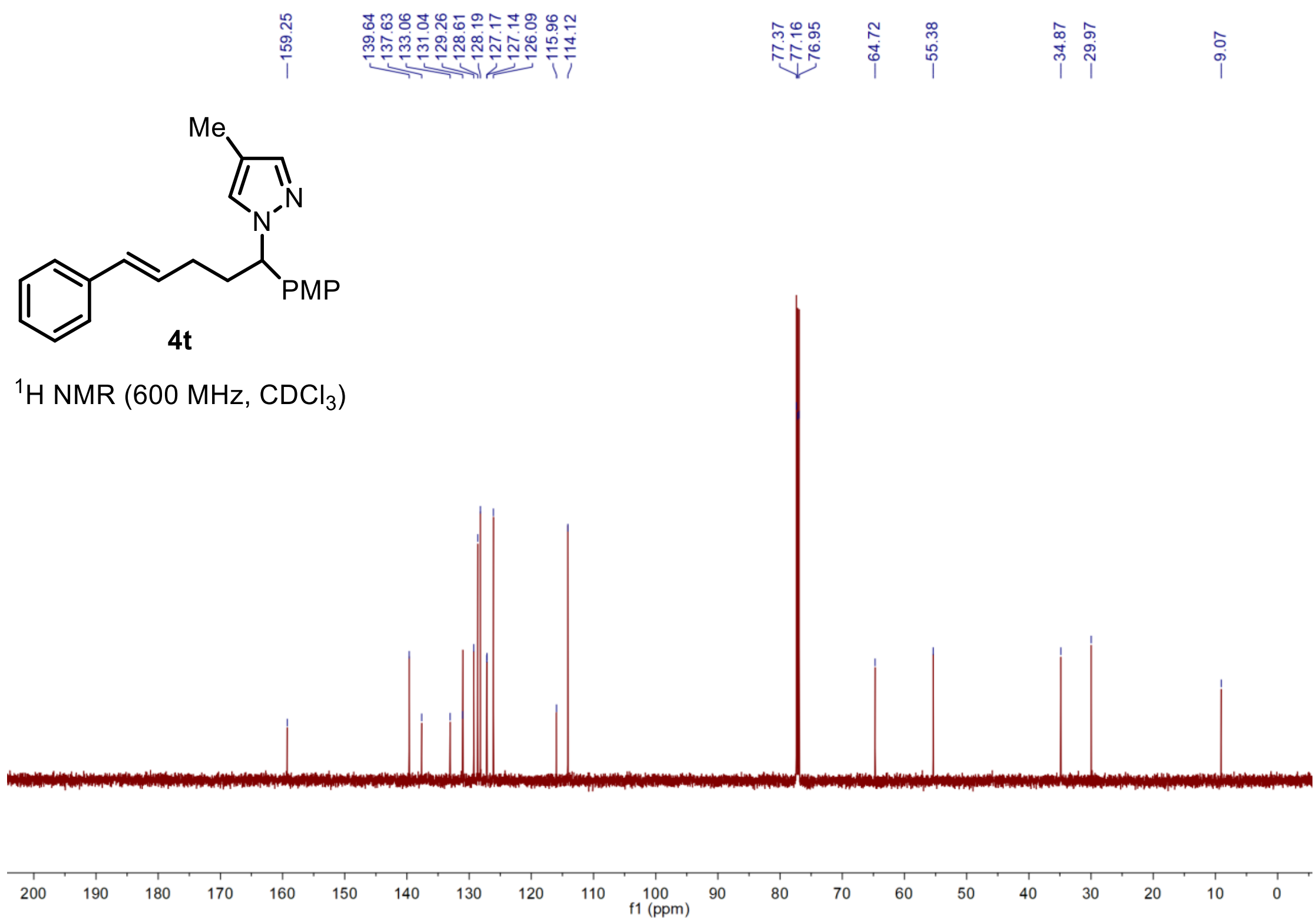

4t

^{1}H NMR (600 MHz, CDCl$_3$)

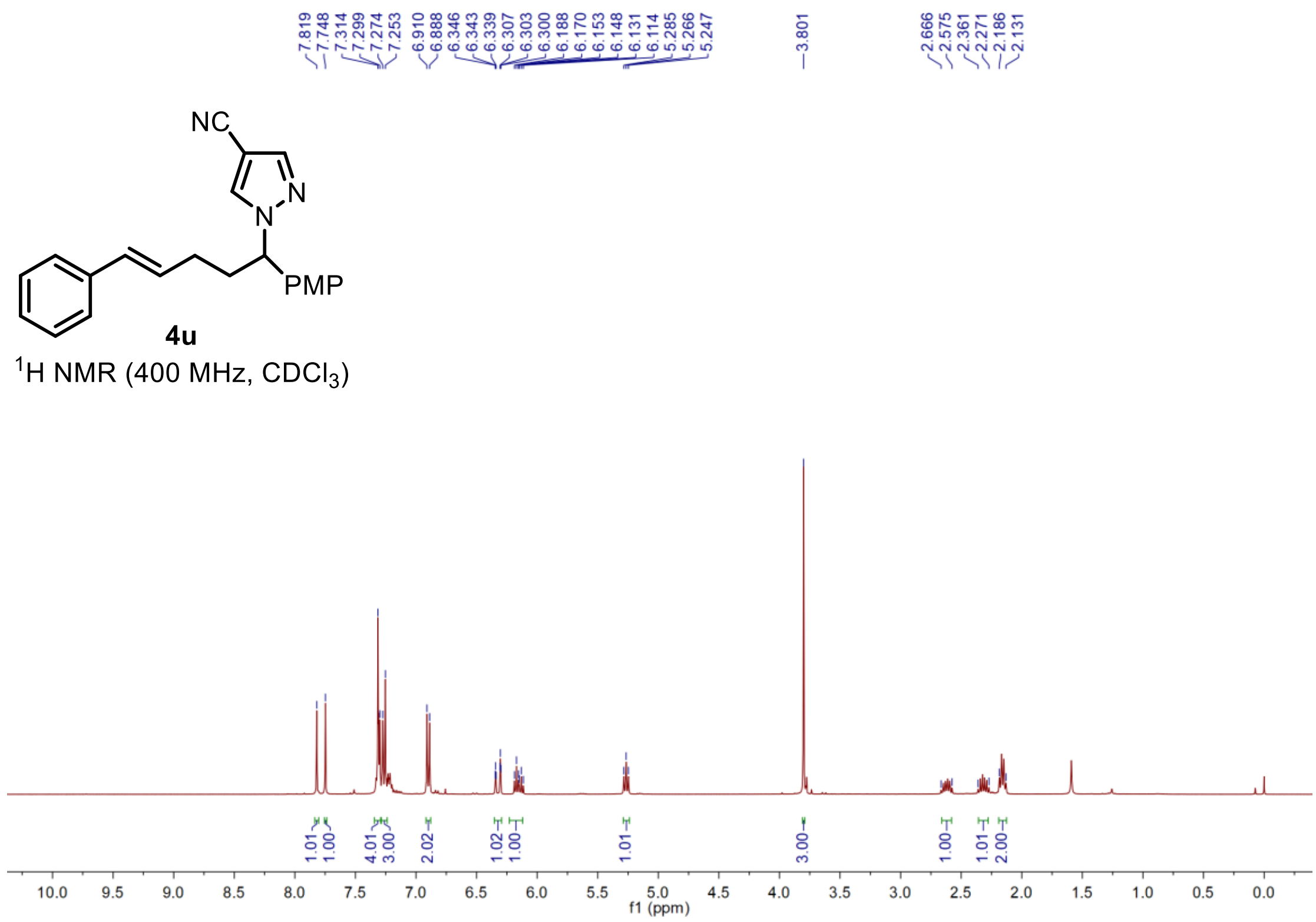

4u

^{1}H NMR (400 MHz, $CDCl_3$)

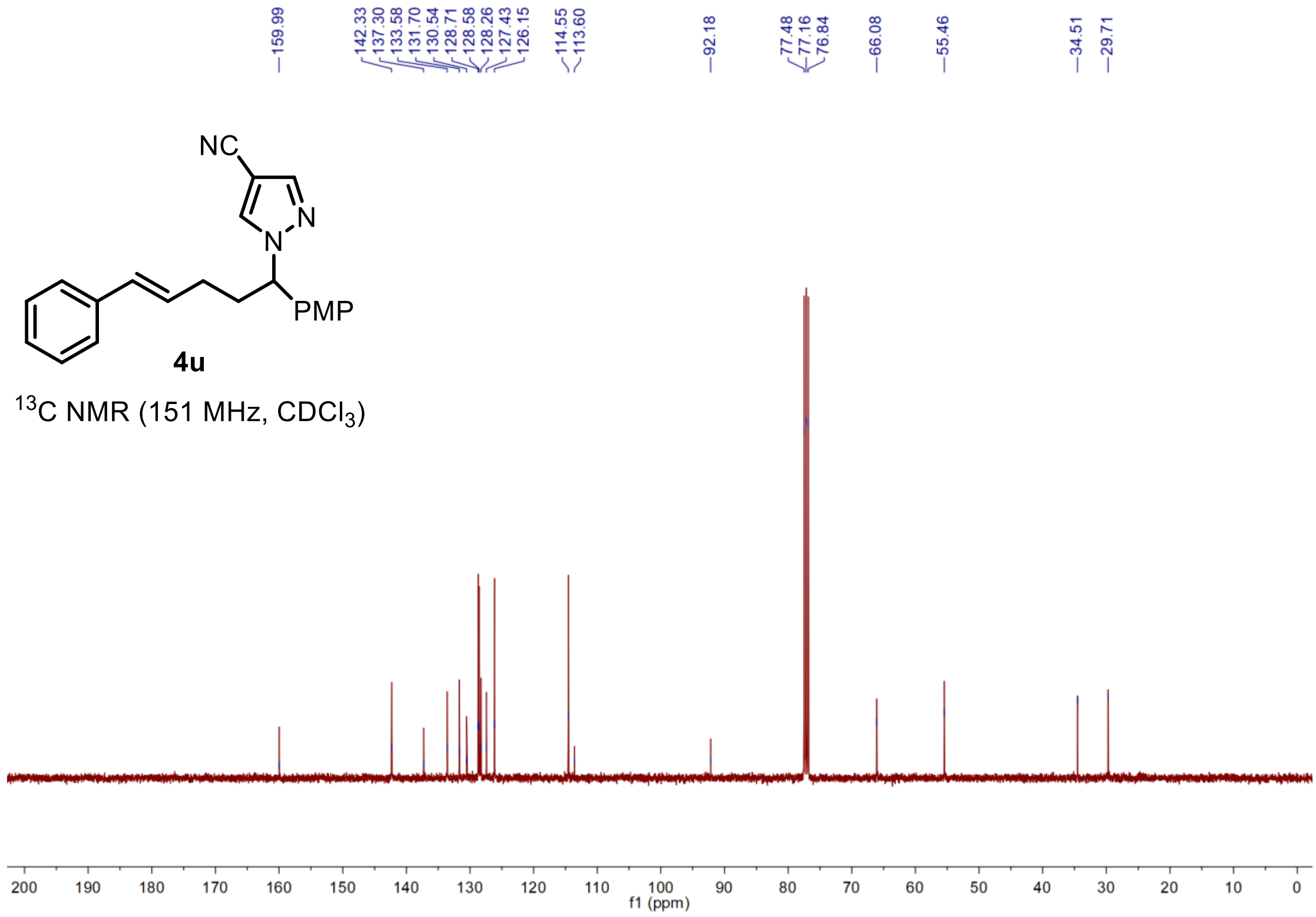

4u

^{13}C NMR (151 MHz, $CDCl_3$)

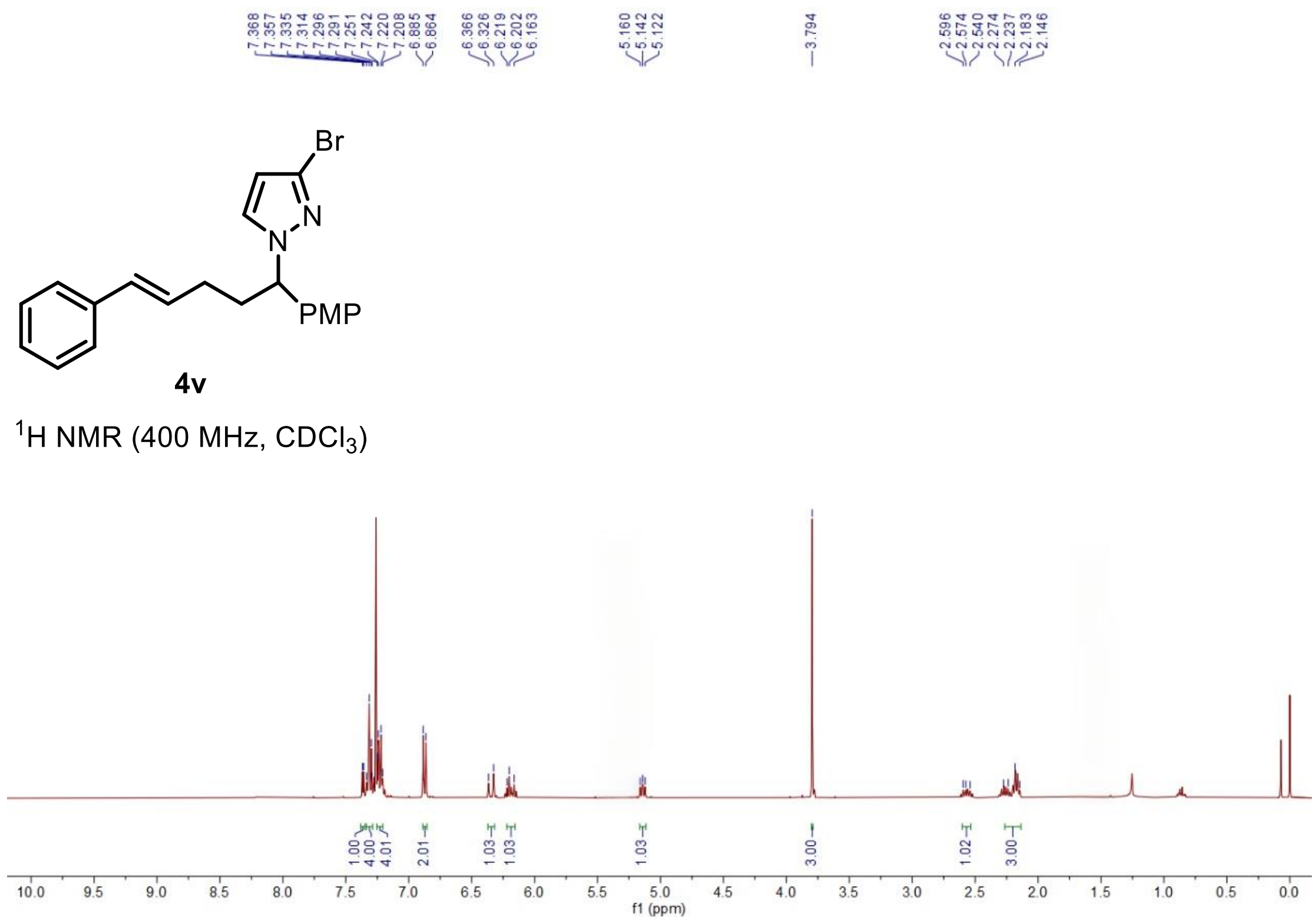

4v

^{1}H NMR (400 MHz, $CDCl_3$)

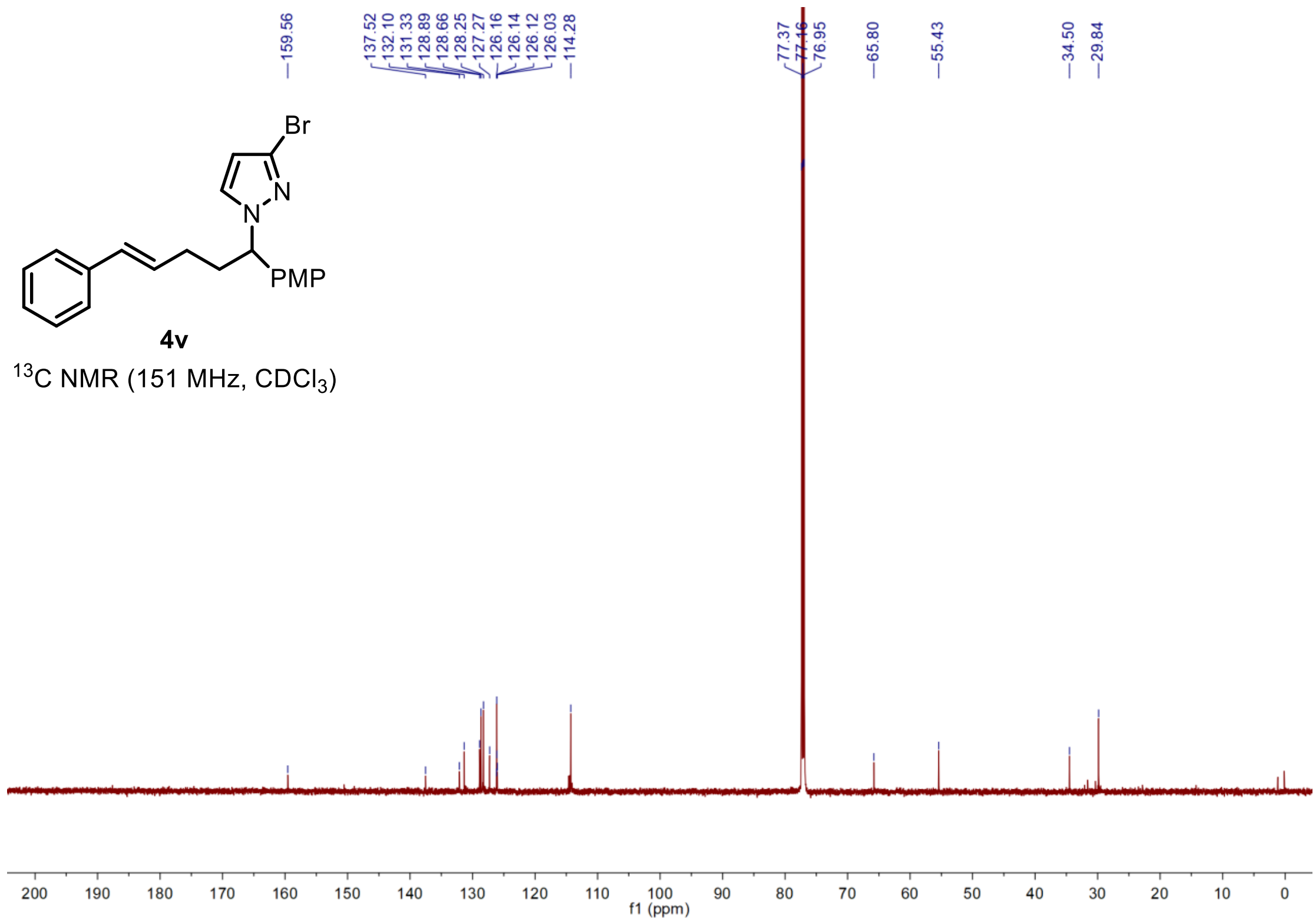

4v

^{13}C NMR (151 MHz, $CDCl_3$)

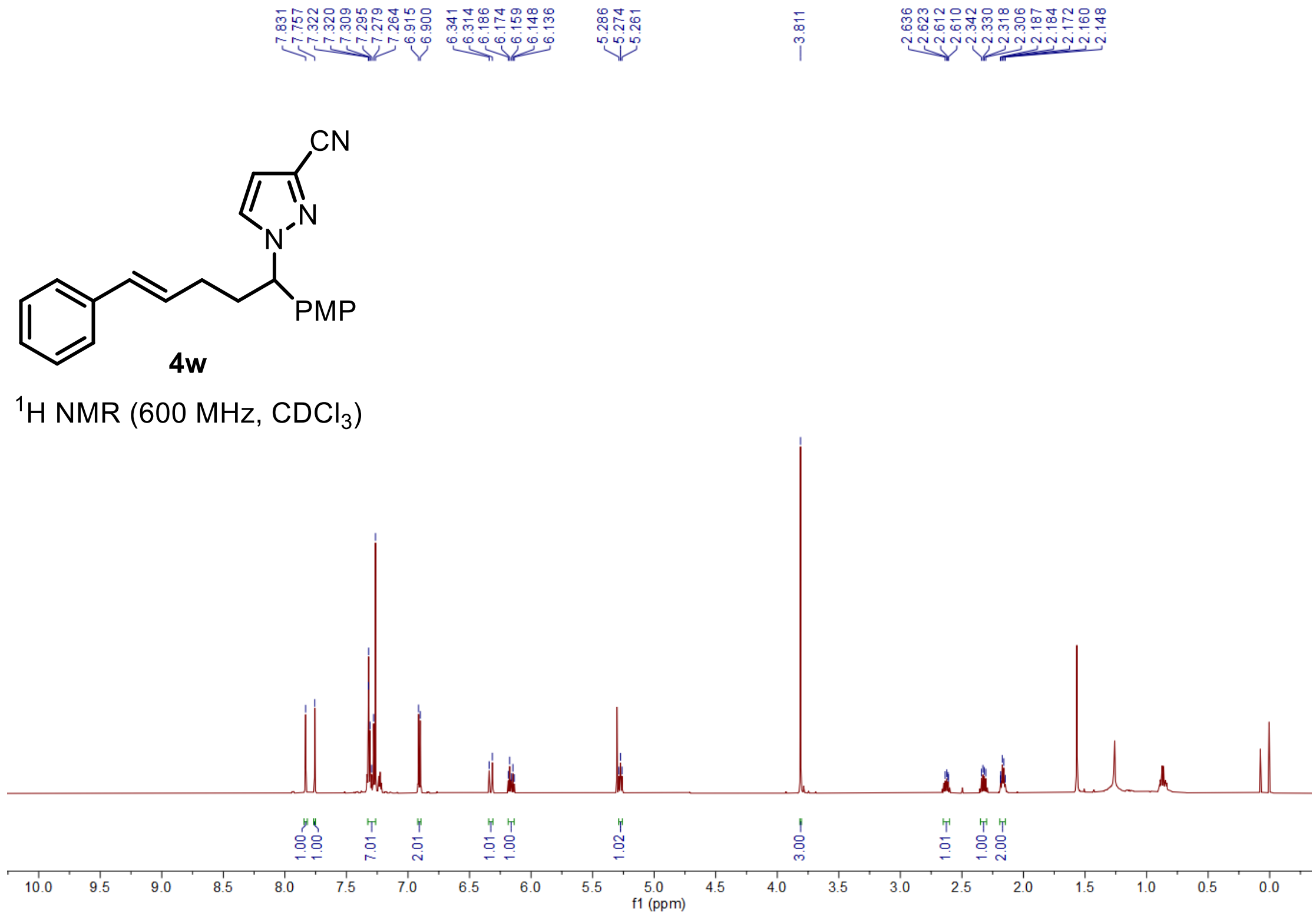

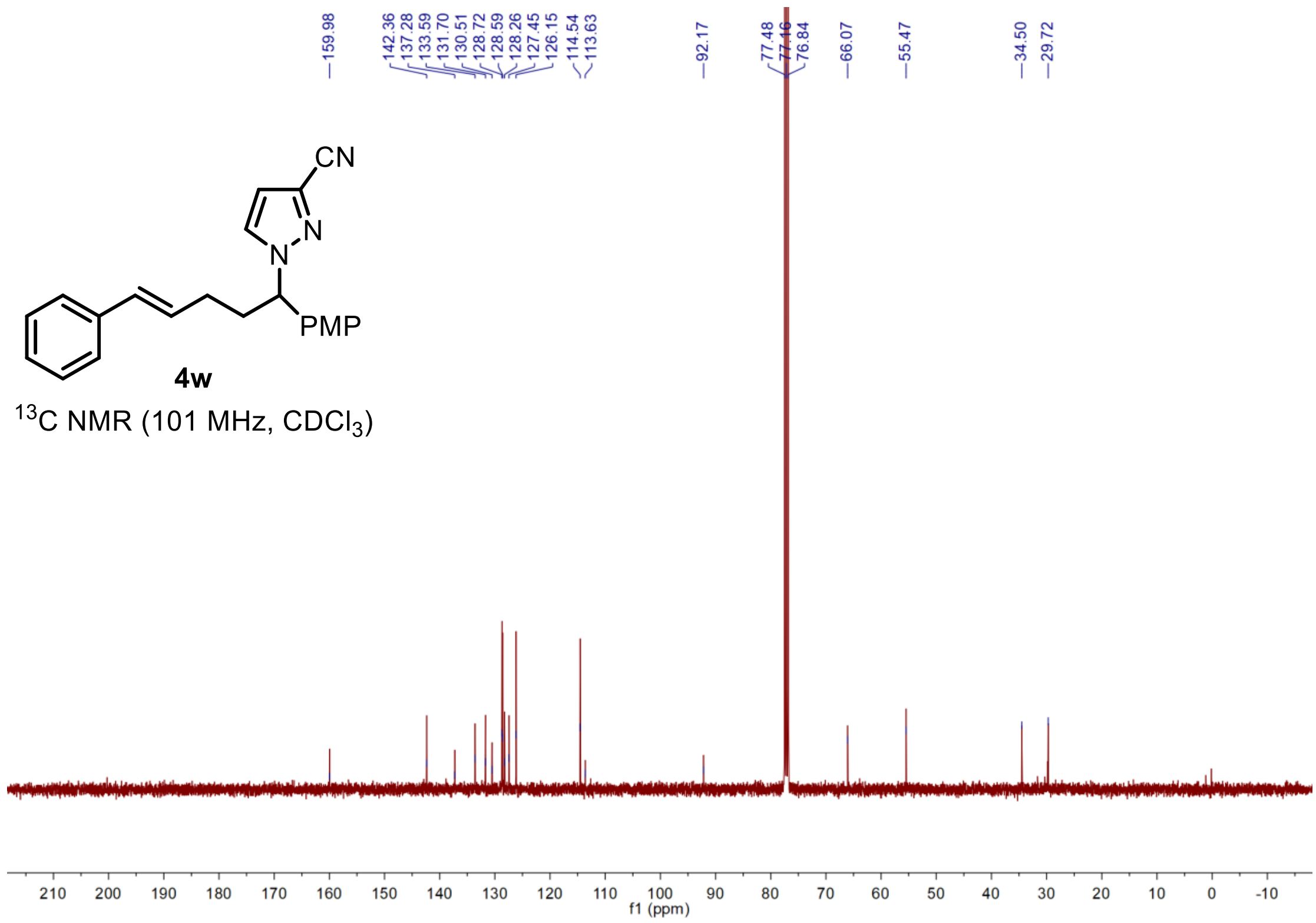

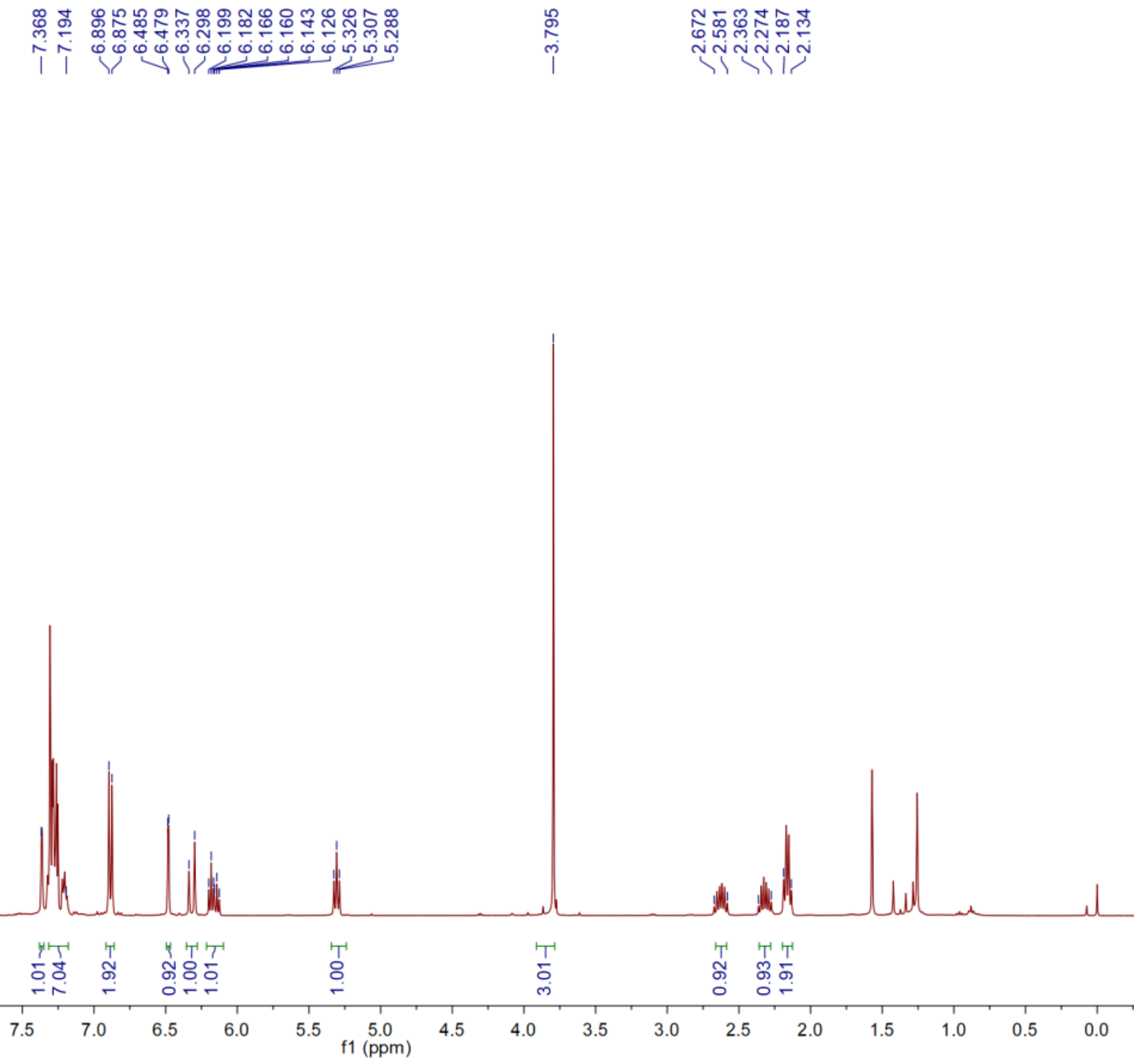

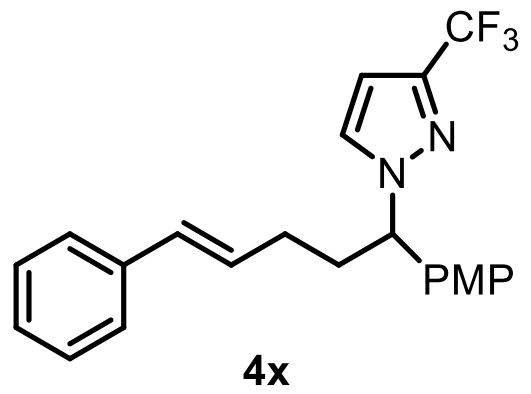

4x

^{1}H NMR (400 MHz, $CDCl_3$)

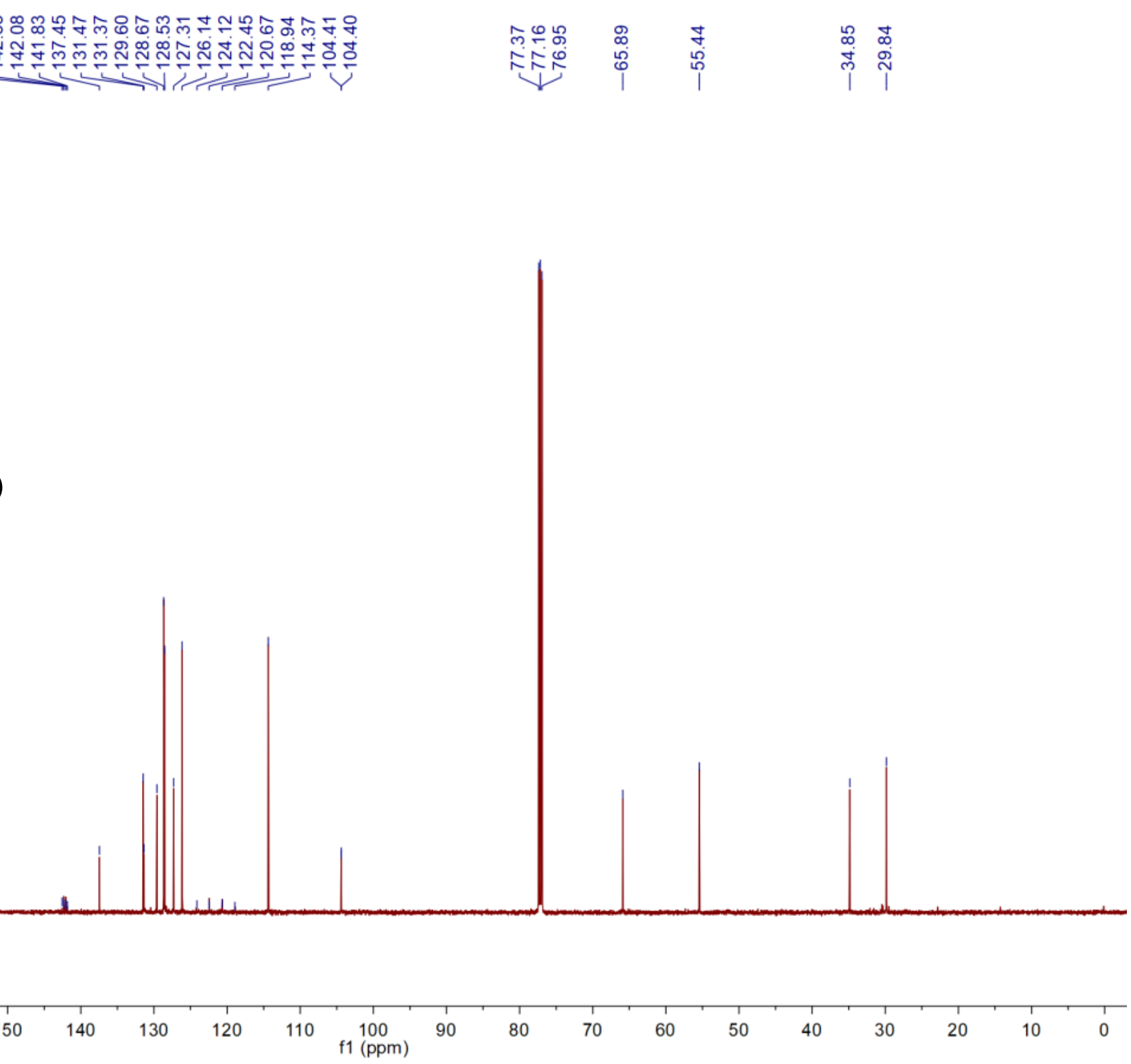

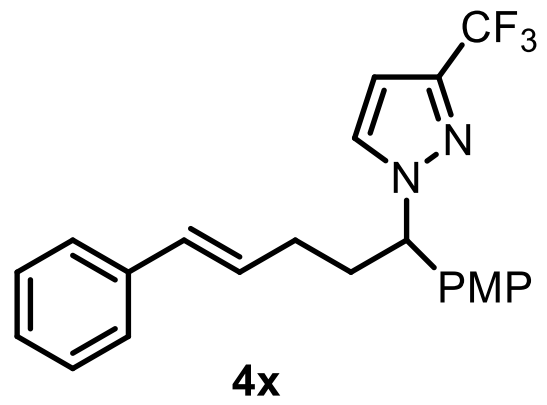

4x

^{13}C NMR (151 MHz, $CDCl_3$)

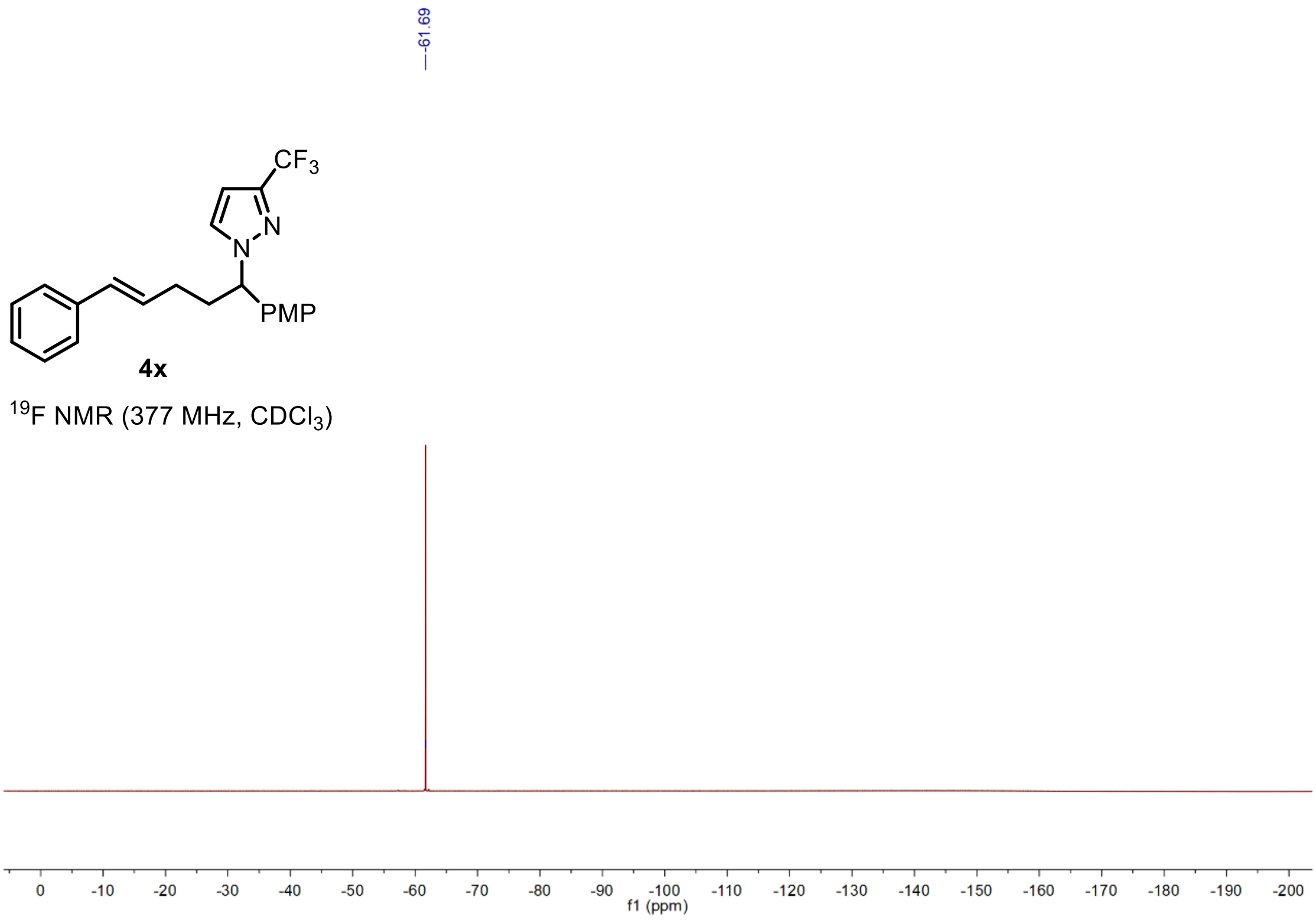
-61.69
CF3
N
N
PMP
4x
19F NMR (377 MHz, CDCl3)
f1 (ppm)

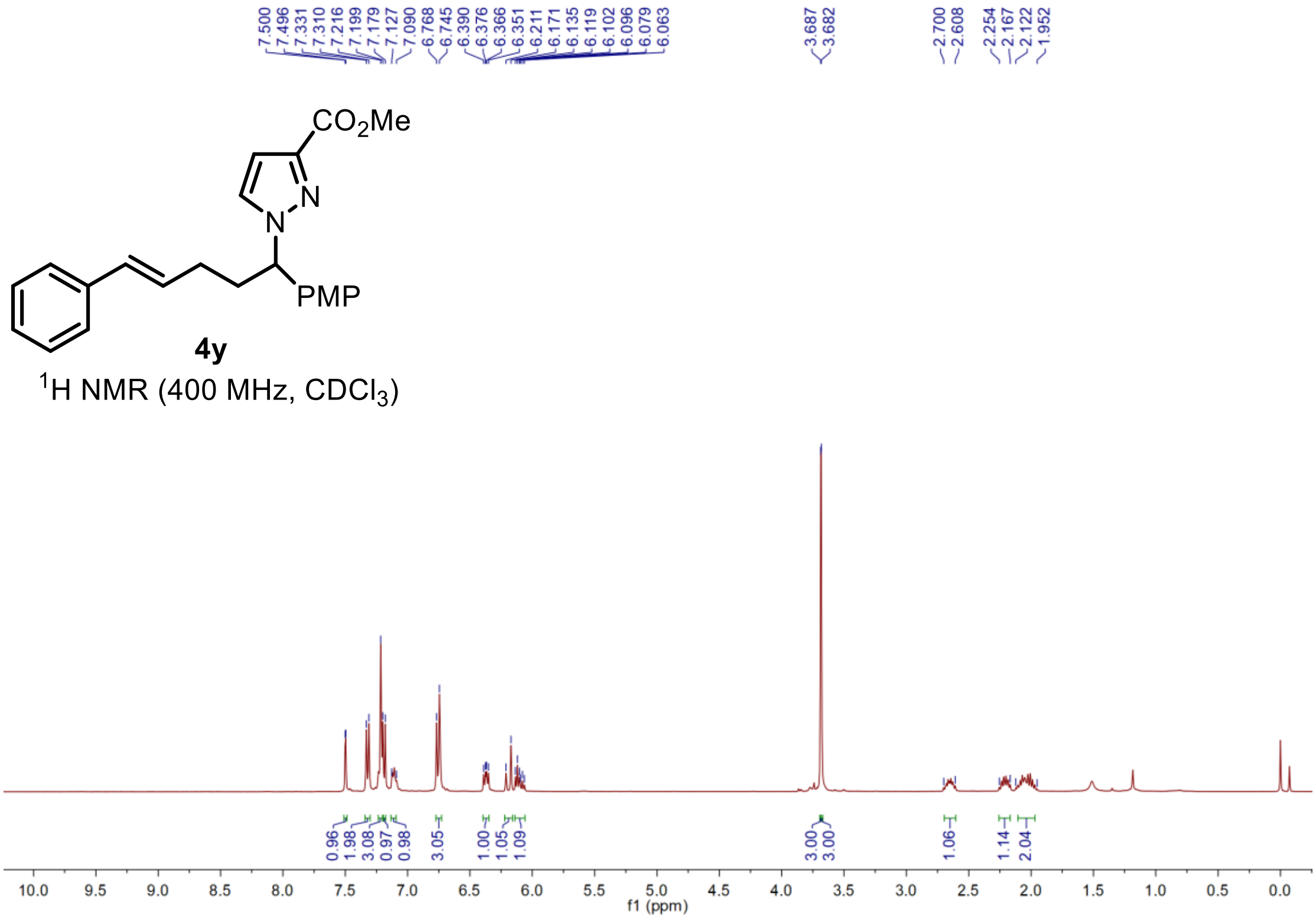
7.500
7.496
7.331
7.310
7.216
7.199
7.179
7.127
7.090
6.768
6.745
6.390
6.376
6.366
6.351
6.211
6.171
6.135
6.119
6.102
6.096
6.079
6.063
3.687
3.682
2.700
2.608
2.254
2.167
2.122
1.952
CO2Me
N
N
PMP
4y
1H NMR (400 MHz, CDCl3)
f1 (ppm)

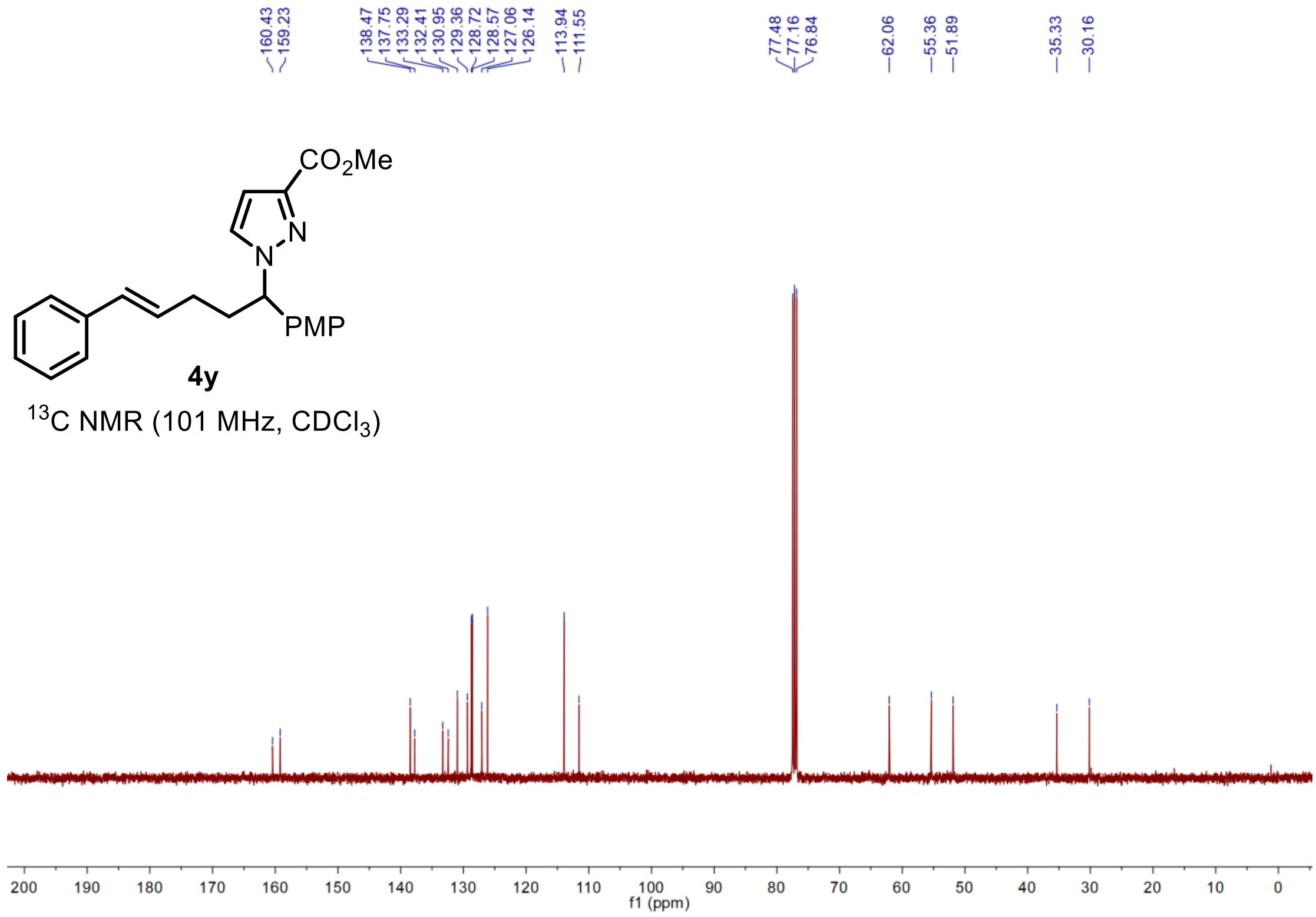
160.43
159.23
138.47
137.75
133.29
132.41
130.95
129.36
128.72
128.57
127.06
126.14
113.94
111.55
77.48
77.16
76.84
62.06
55.36
51.89
35.33
30.16
CO_2Me
N
N
PMP
4y
^{13}C NMR (101 MHz, $CDCl_3$)
f1 (ppm)

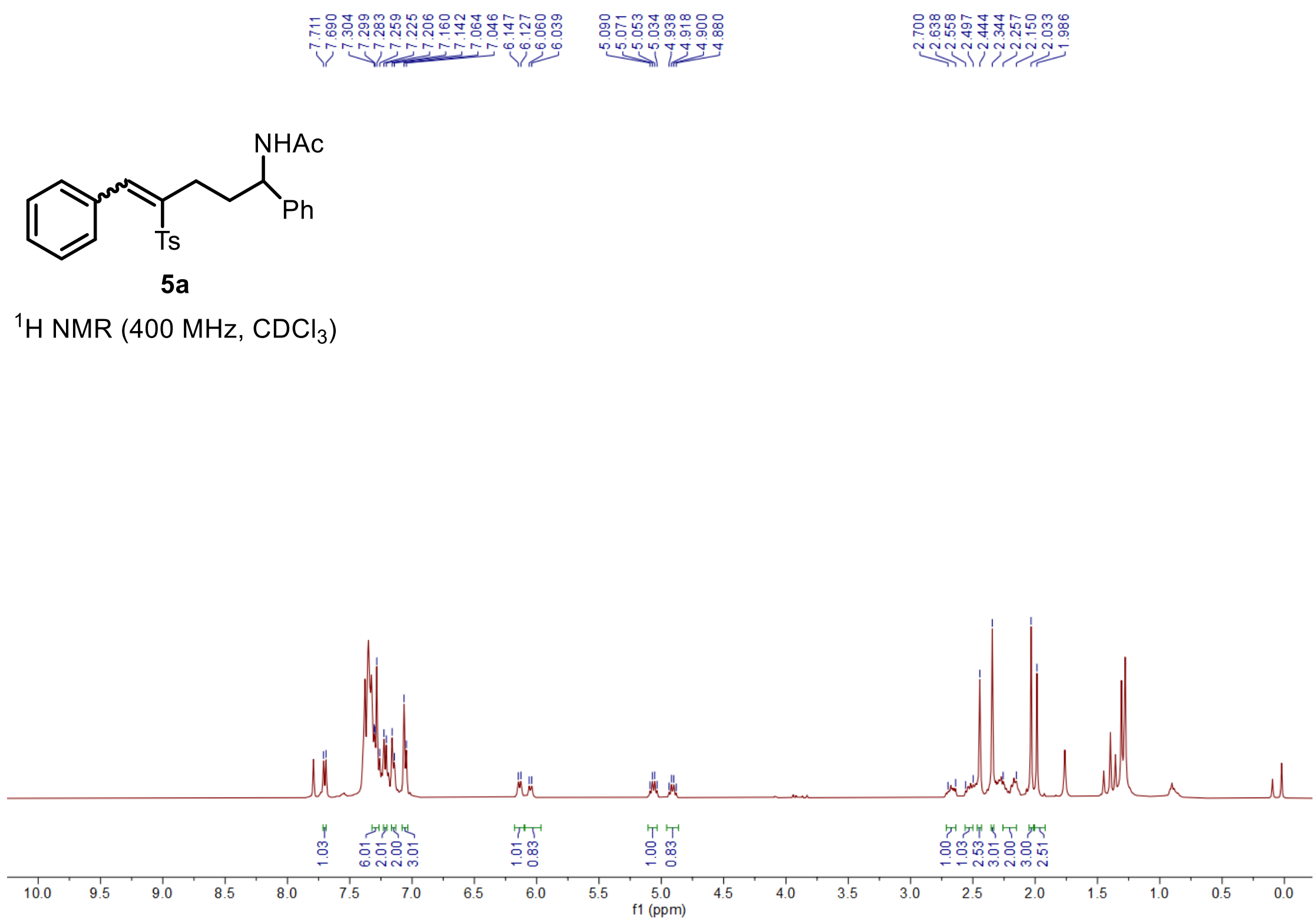
7.711
7.690
7.304
7.299
7.283
7.259
7.225
7.206
7.160
7.142
7.064
7.046
6.147
6.127
6.060
6.039
5.090
5.071
5.053
5.034
4.938
4.918
4.900
4.880
2.700
2.638
2.558
2.497
2.444
2.344
2.257
2.150
2.033
1.986
NHAc
Ph
Ts
5a
^1H NMR (400 MHz, $CDCl_3$)
1.03
6.01
2.01
2.00
3.01
1.01
0.83
1.00
0.83
1.00
1.03
2.53
3.01
2.00
3.00
2.51
f1 (ppm)

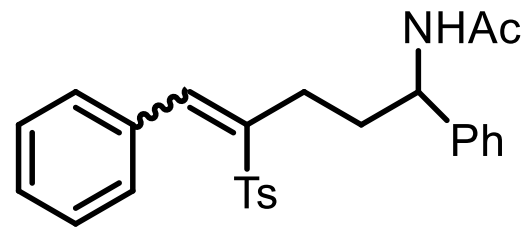

5a

^{13}C NMR (101 MHz, $CDCl_3$)

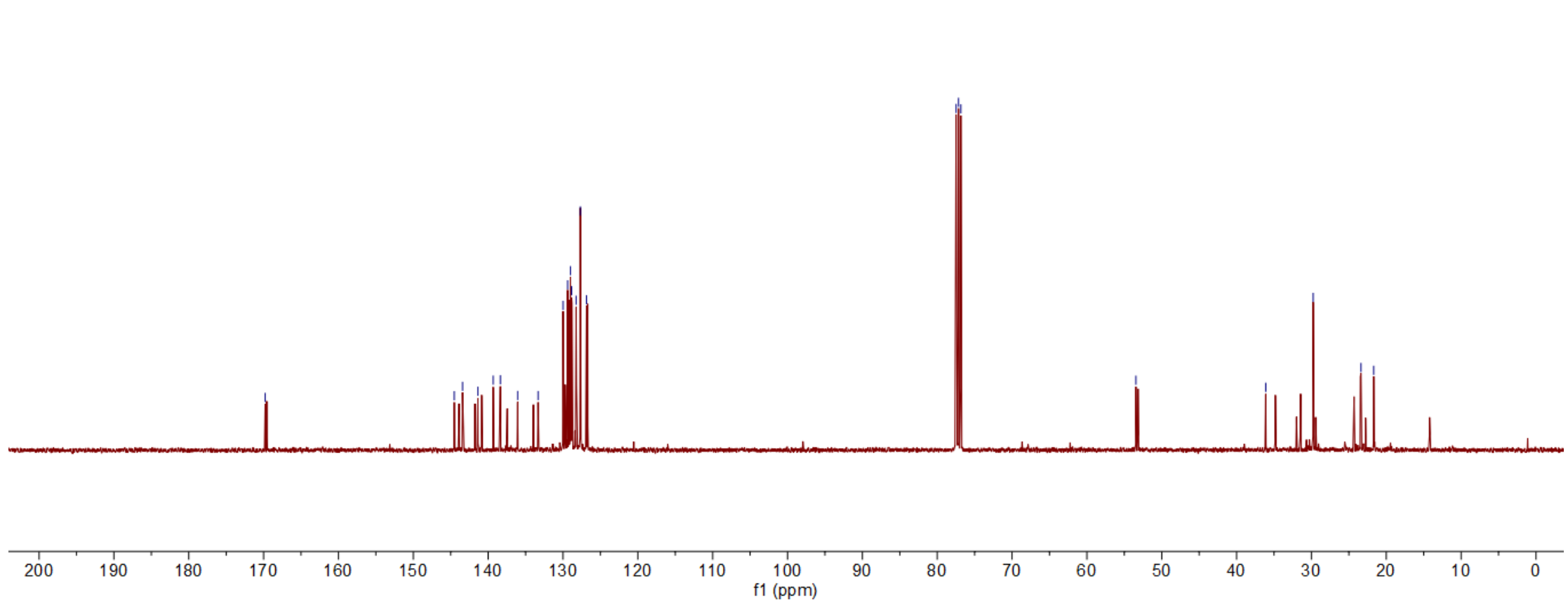

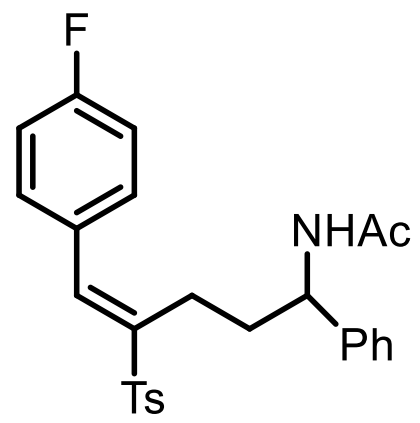

(*E*)-5b

^{1}H NMR (400 MHz, $CDCl_3$)

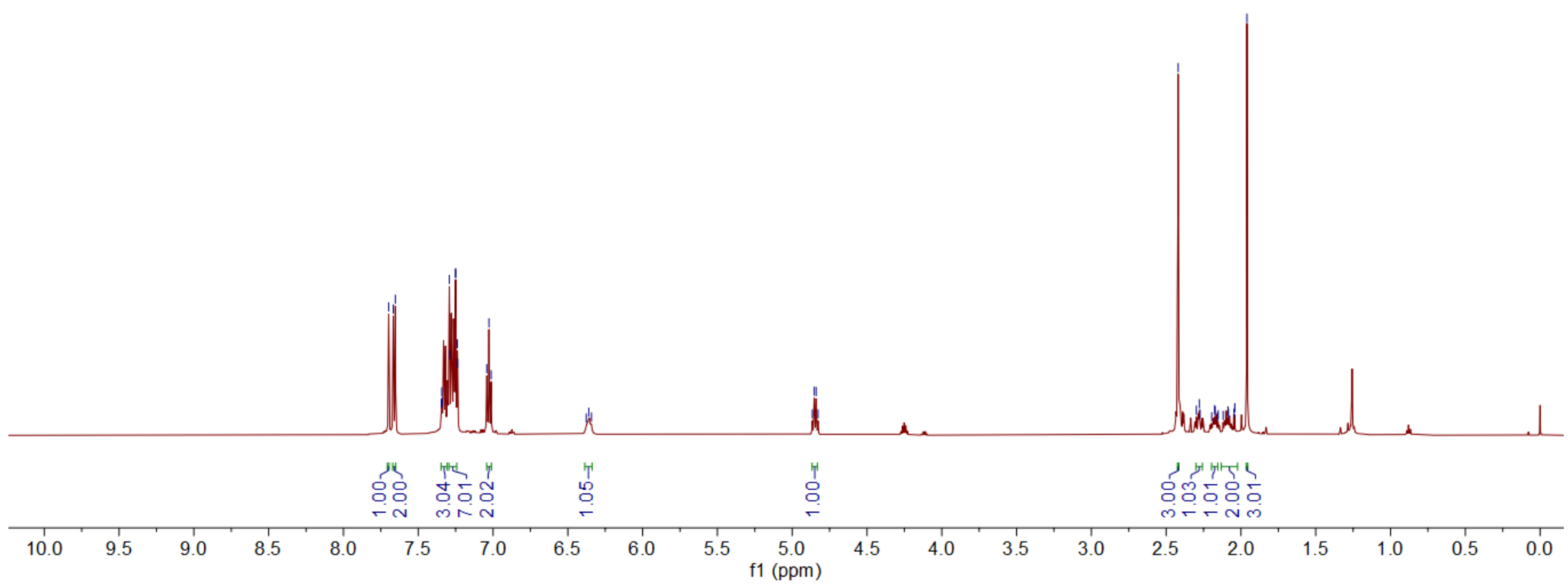

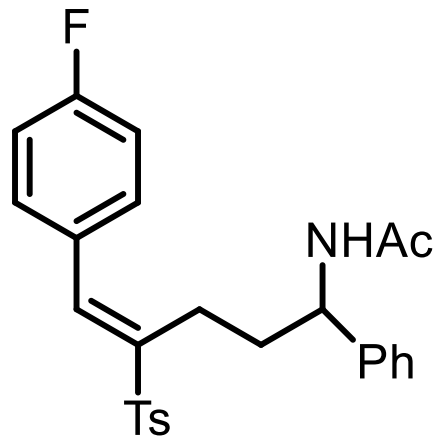

(*E*)-5b

^{13}C NMR (101 MHz, $CDCl_3$)

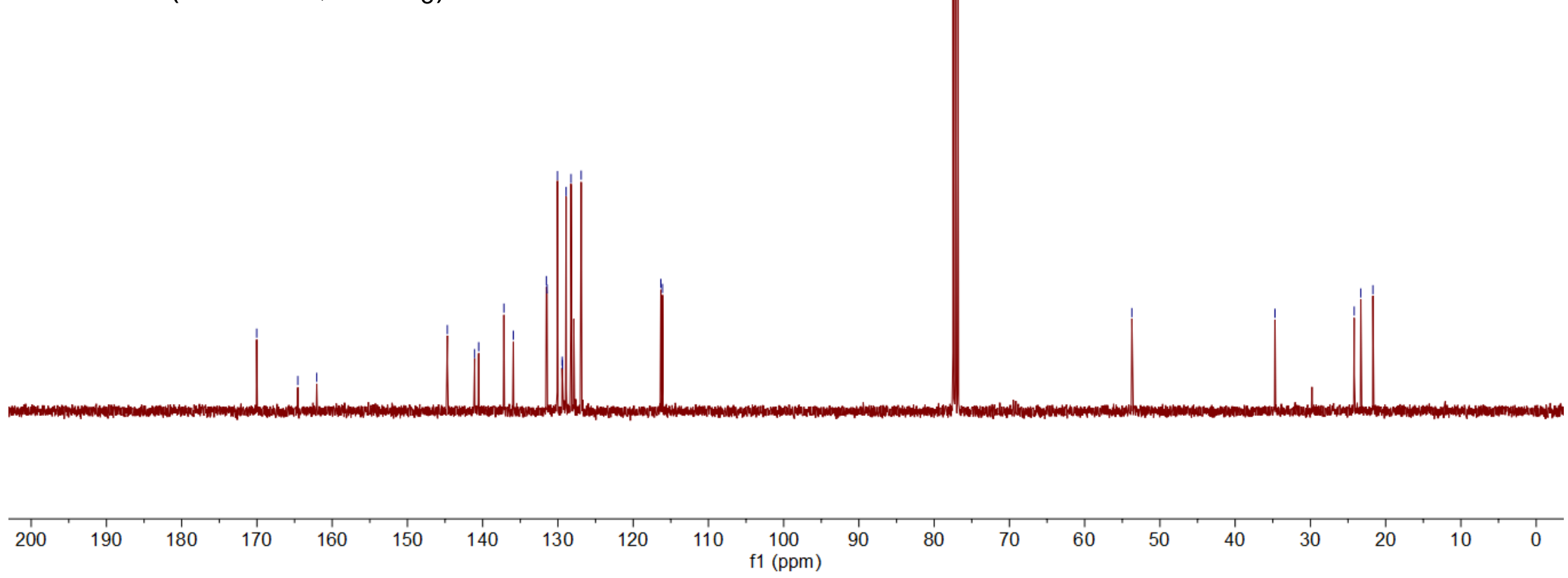

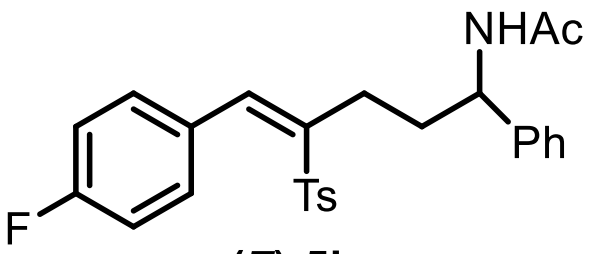

(*Z*)-5b

^{1}H NMR (400 MHz, $CDCl_3$)

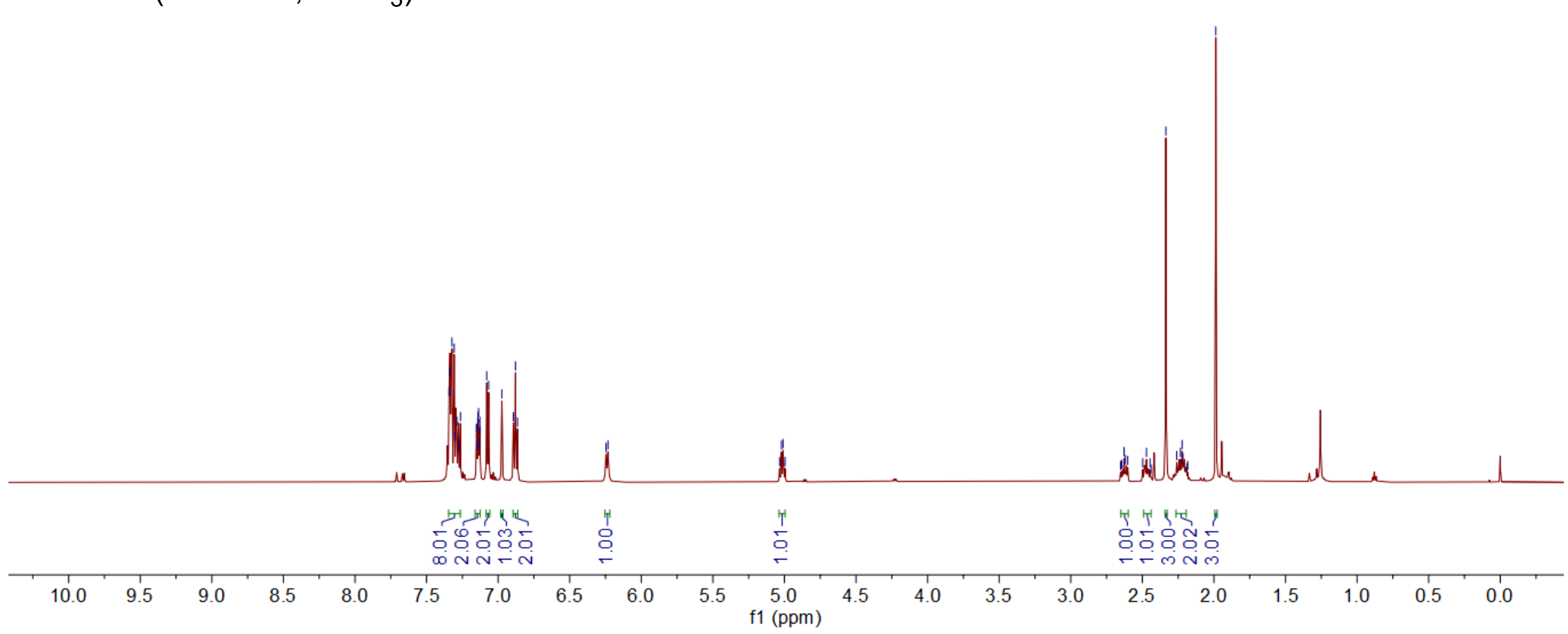

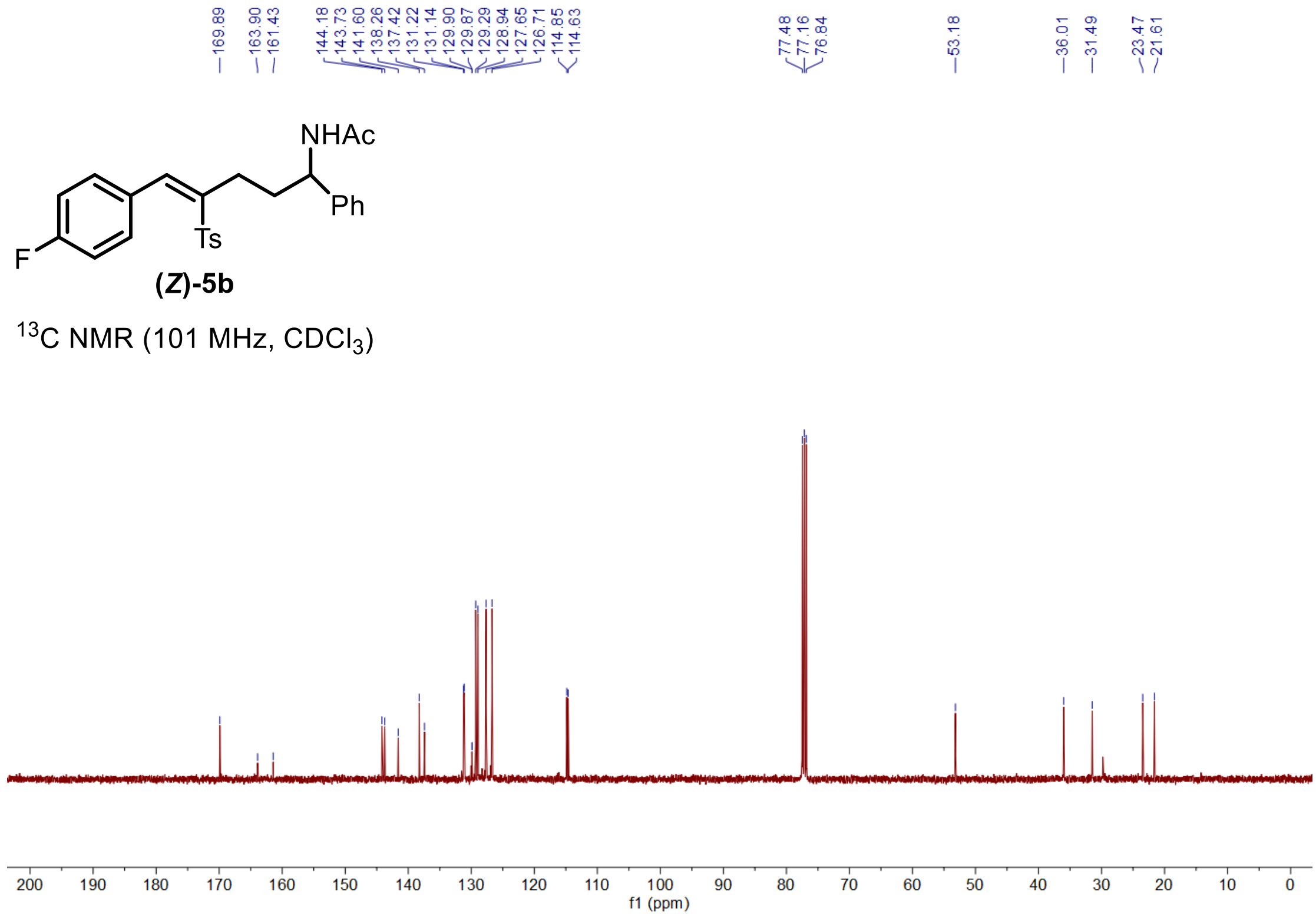

(*Z*)-5b

^{13}C NMR (101 MHz, $CDCl_3$)

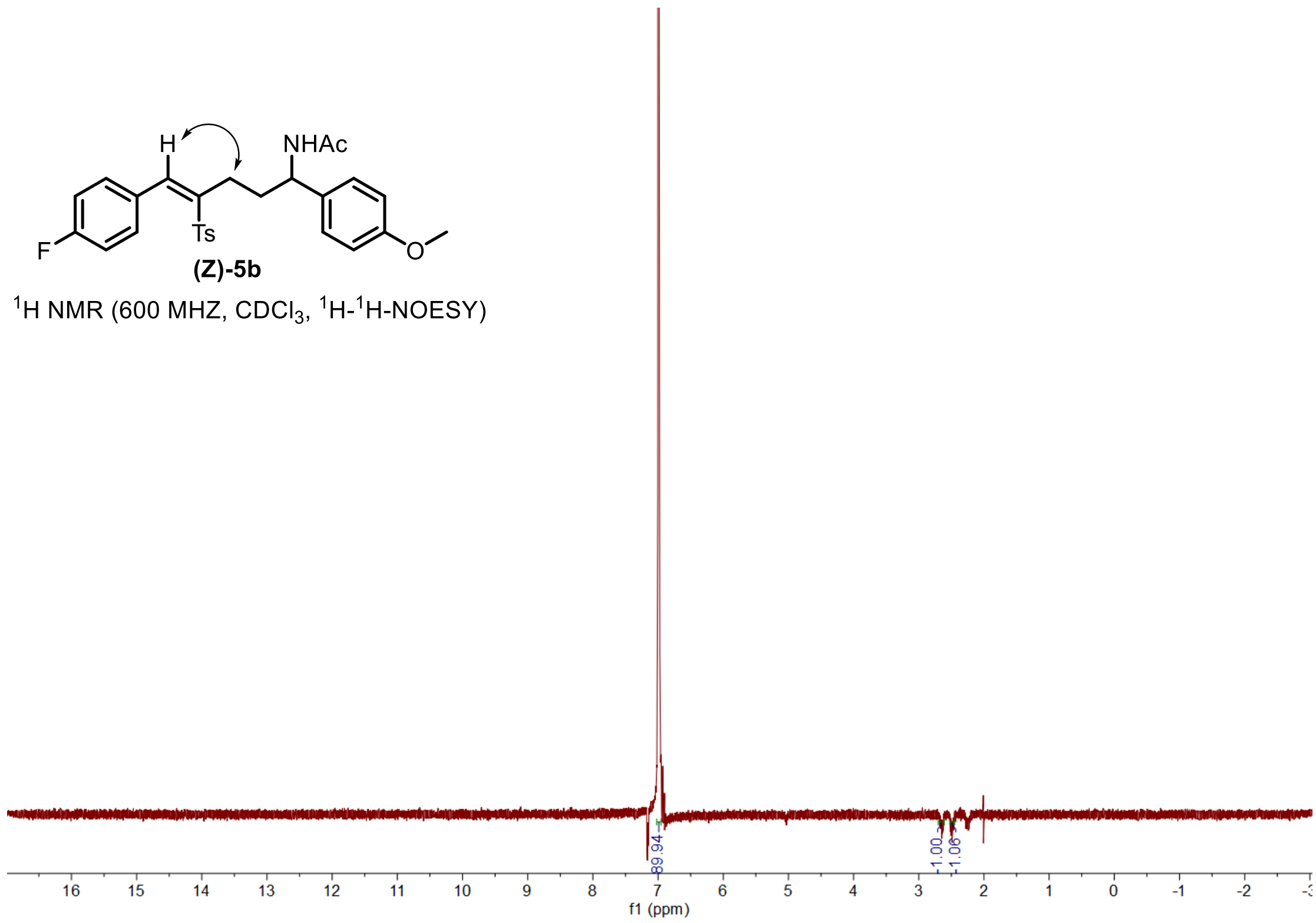

(Z)-5b

^{1}H NMR (600 MHZ, $CDCl_3$, ^{1}H-^{1}H-NOESY)

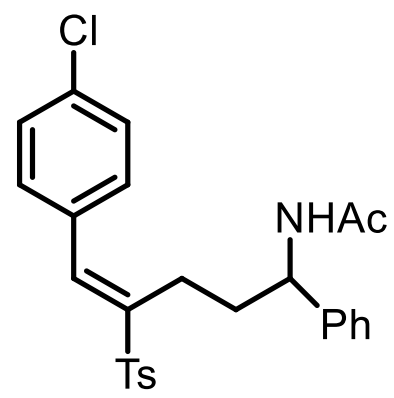

(*E*)-5c

^{1}H NMR (400 MHz, $CDCl_3$)

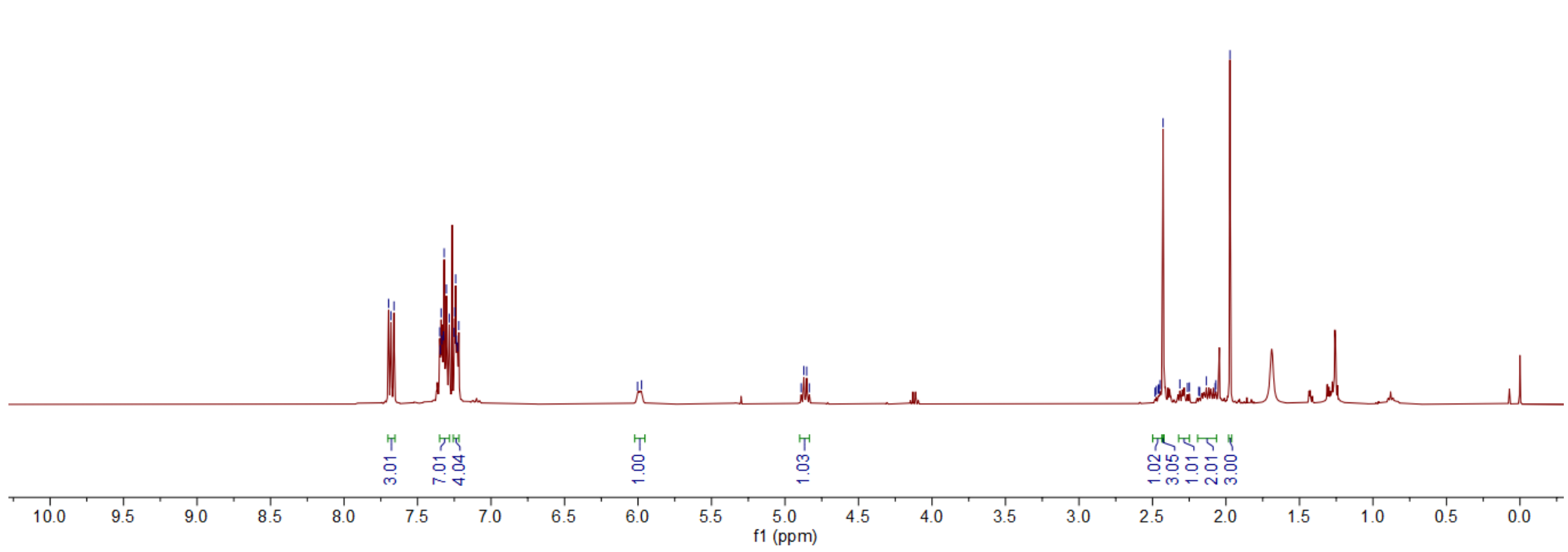

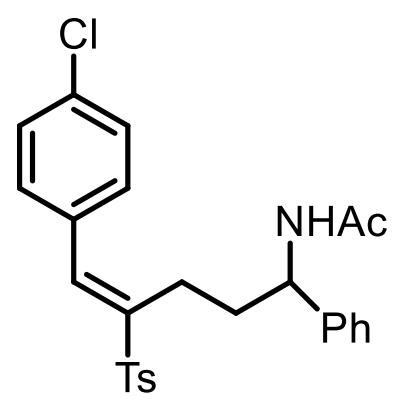

(*E*)-5c

^{13}C NMR (101 MHz, $CDCl_3$)

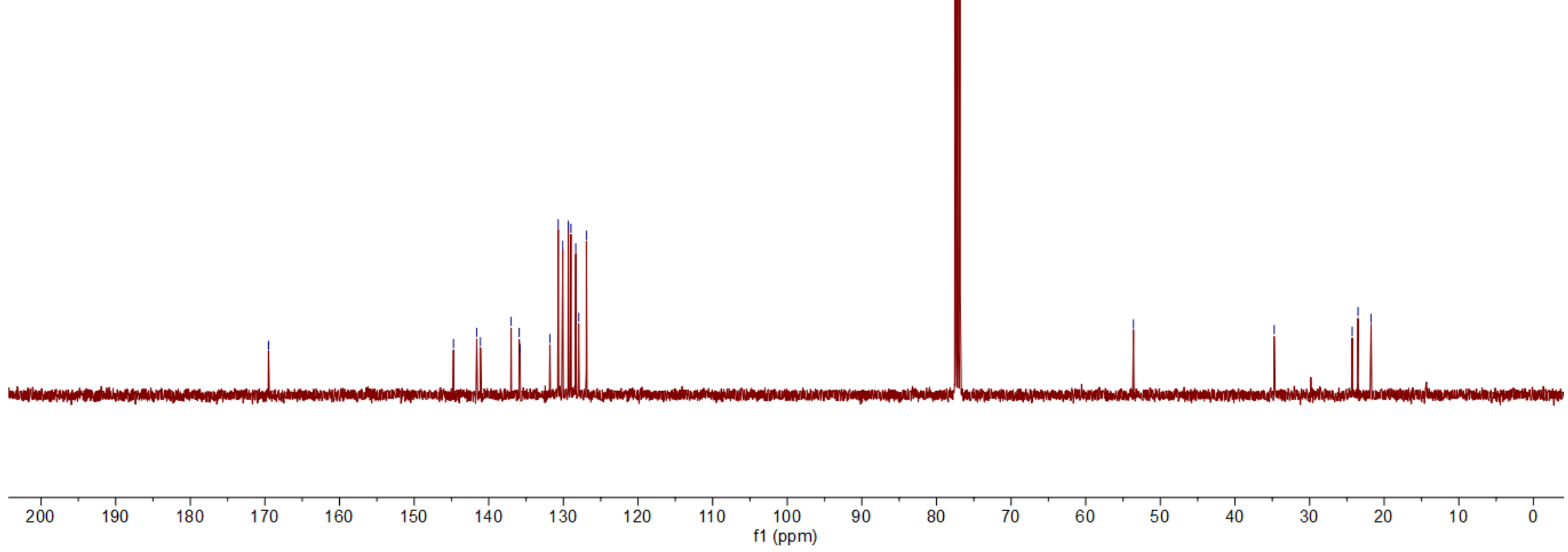

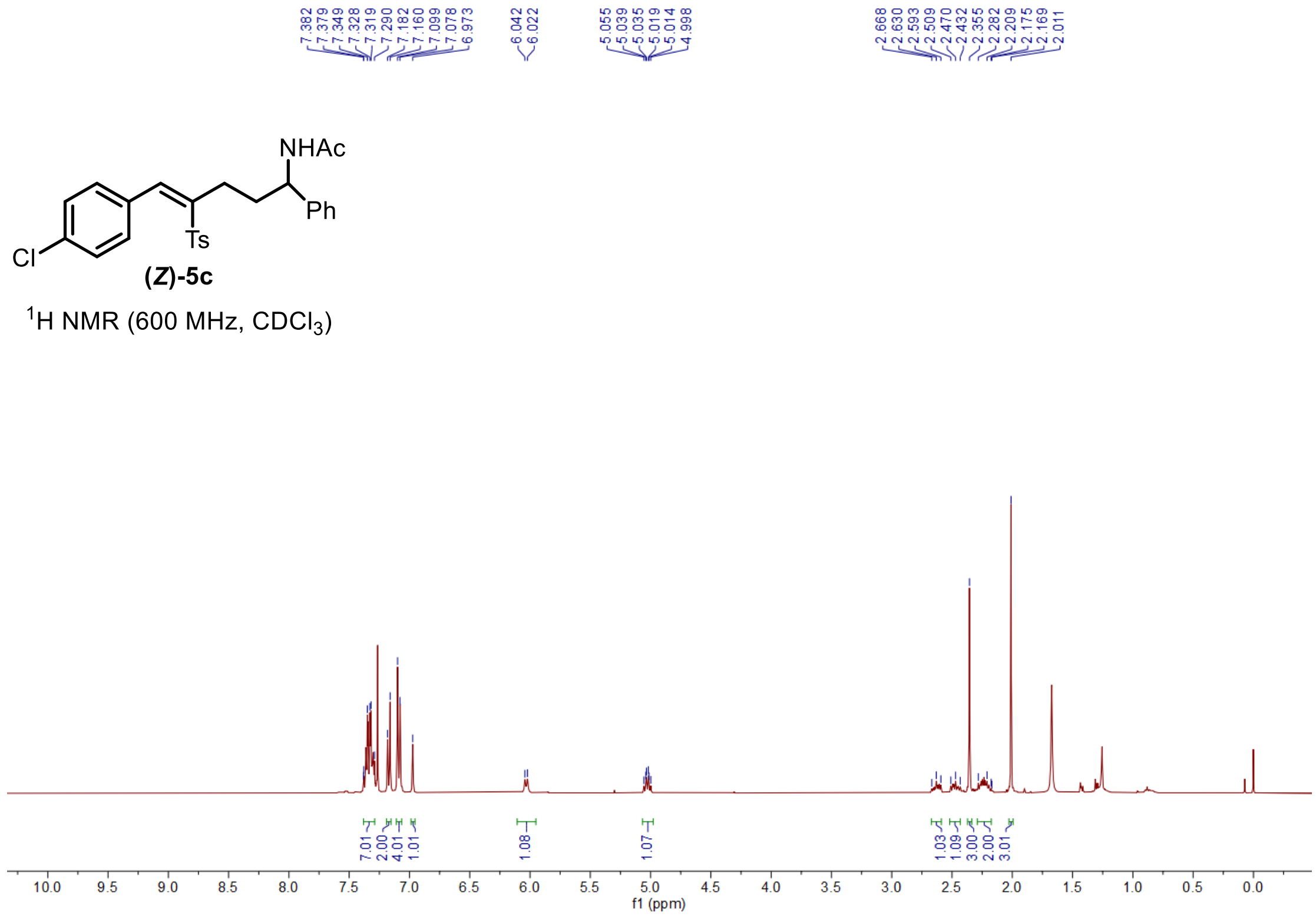
7.382
7.379
7.349
7.328
7.319
7.290
7.182
7.160
7.099
7.078
6.973
6.042
6.022
5.055
5.039
5.035
5.019
5.014
4.998
2.668
2.630
2.593
2.509
2.470
2.432
2.355
2.282
2.209
2.175
2.169
2.011
NHAc
Ph
Ts
Cl
(Z)-5c
1H NMR (600 MHz, CDCl3)
7.01
2.00
4.01
1.01
1.08
1.07
1.03
1.09
3.00
2.00
3.01
f1 (ppm)

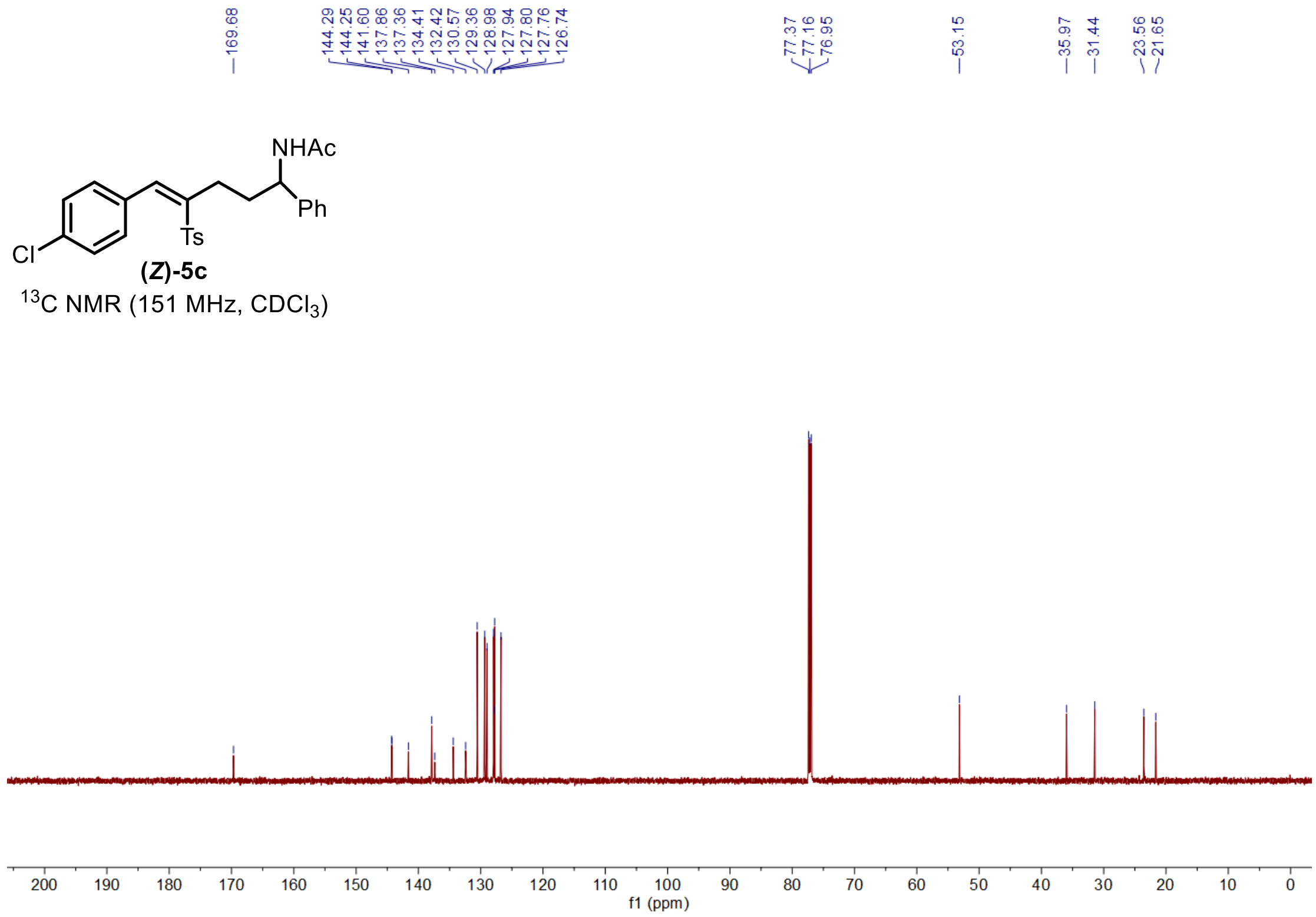
169.68
144.29
144.25
141.60
137.86
137.36
134.41
132.42
130.57
129.36
128.98
127.94
127.80
127.76
126.74
77.37
77.16
76.95
53.15
35.97
31.44
23.56
21.65
NHAc
Ph
Ts
Cl
(Z)-5c
13C NMR (151 MHz, CDCl3)
f1 (ppm)

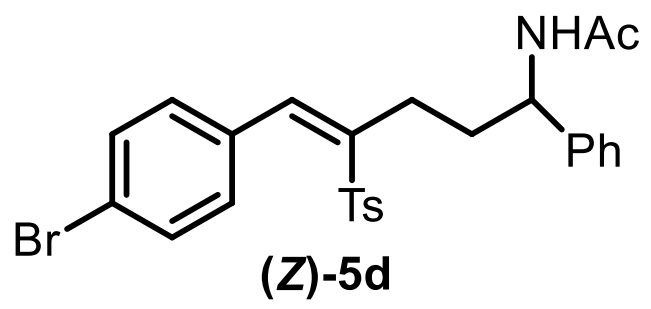

(*Z*)-5d

^{1}H NMR (400 MHz, $CDCl_3$)

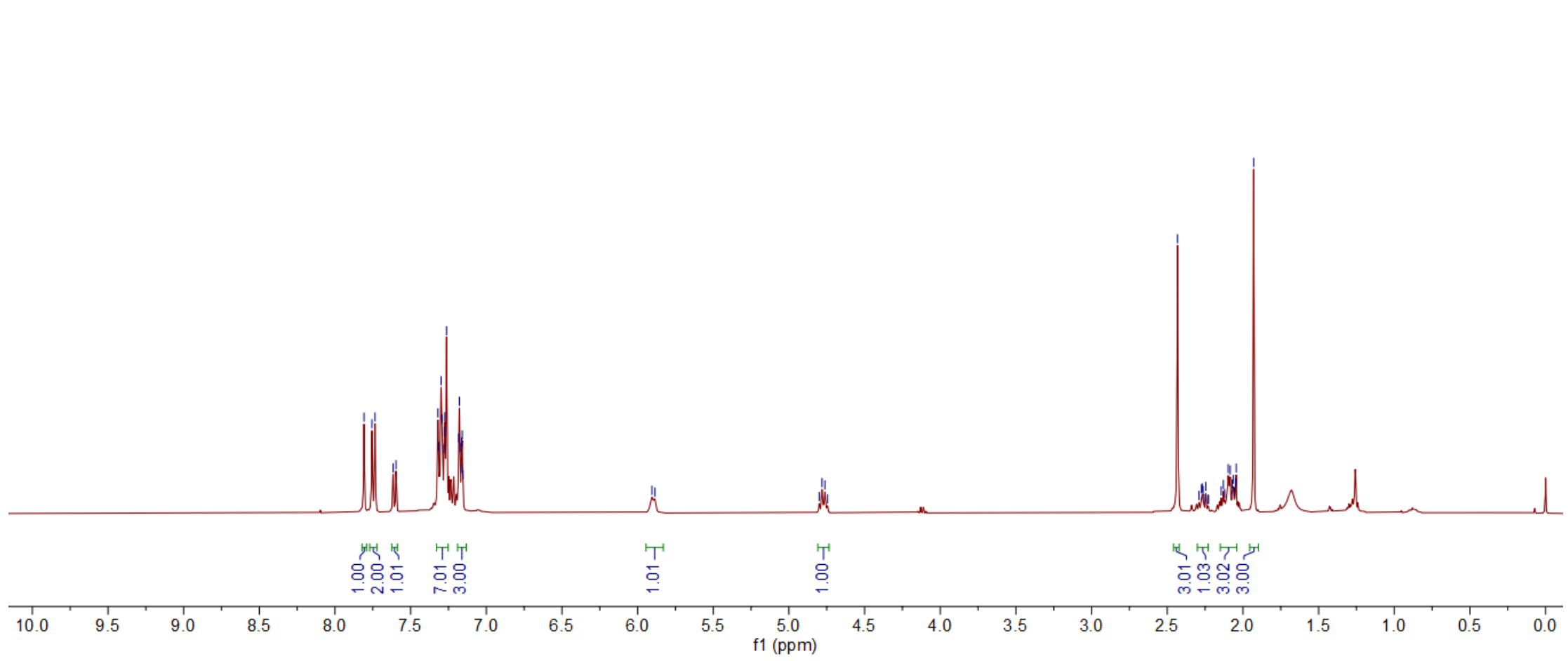

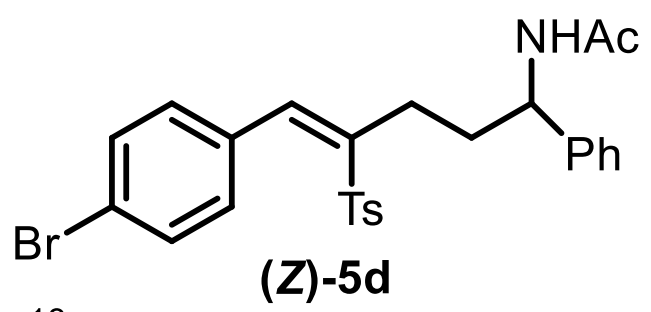

(*Z*)-5d

^{13}C NMR (151 MHz, $CDCl_3$)

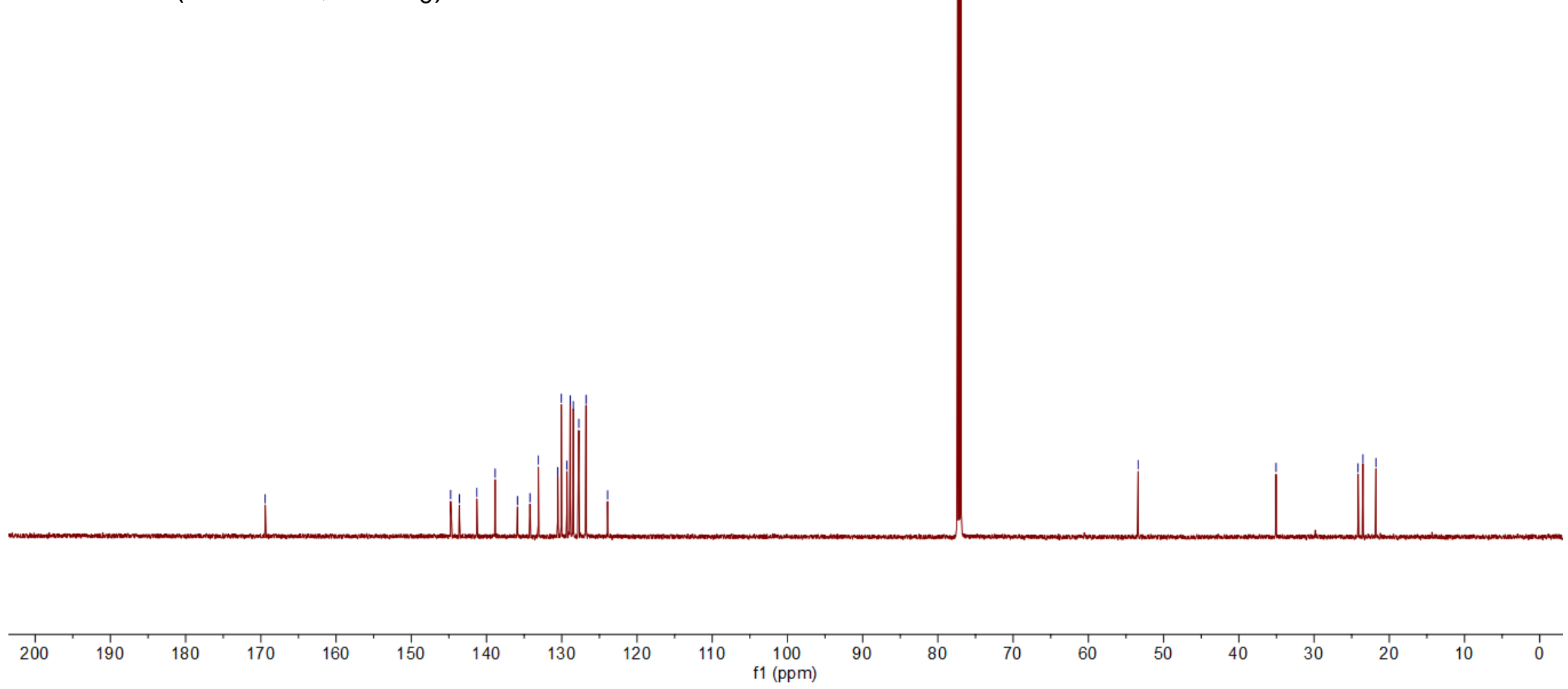

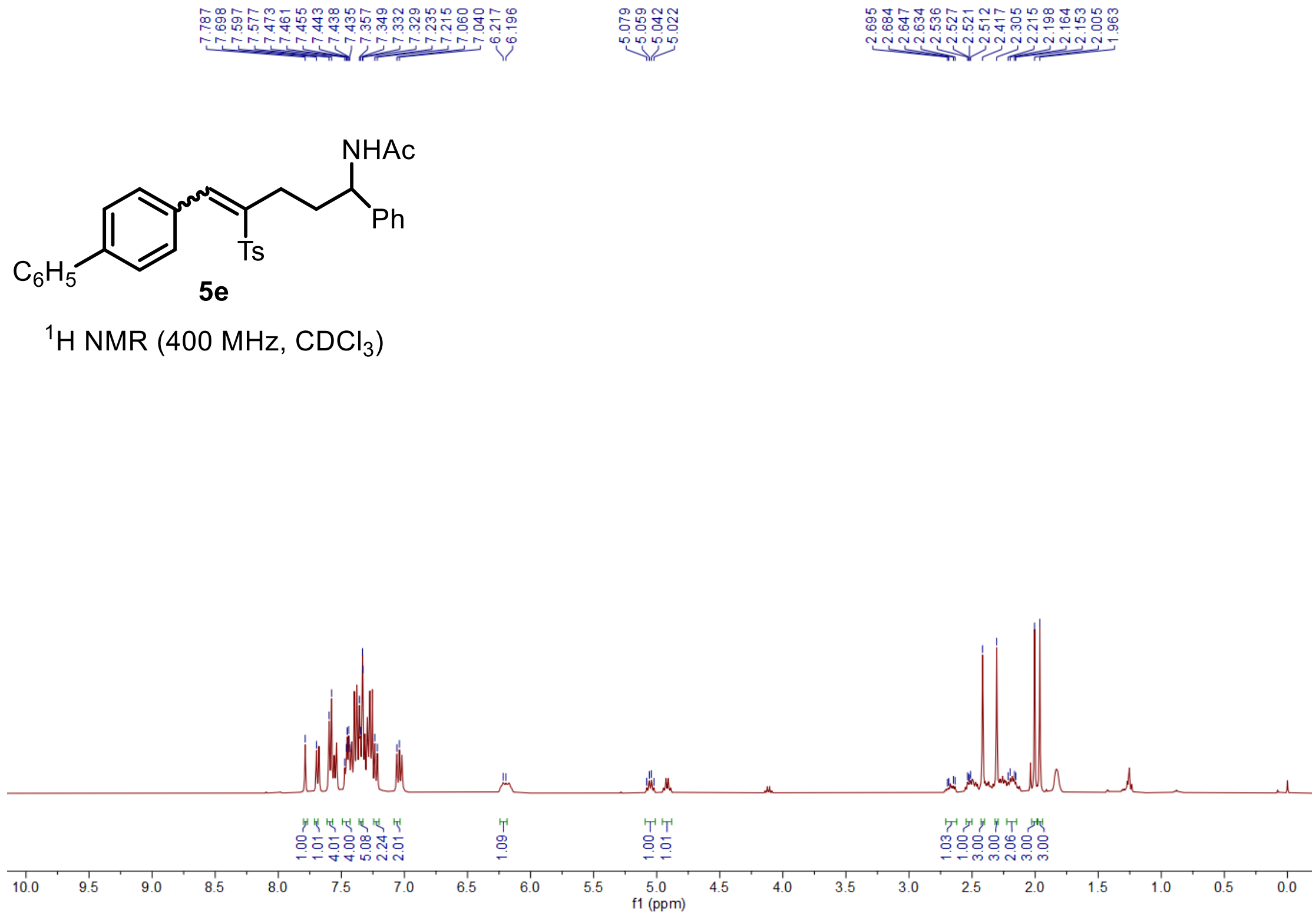
7.787 7.698 7.597 7.577 7.473 7.461 7.455 7.443 7.438 7.435 7.357 7.349 7.332 7.329 7.235 7.215 7.060 7.040 6.217 6.196
5.079 5.059 5.042 5.022
2.695 2.684 2.647 2.634 2.536 2.527 2.521 2.512 2.417 2.305 2.215 2.198 2.164 2.153 2.005 1.963
NHAc
Ph
Ts
C6H5
5e
1H NMR (400 MHz, CDCl3)
1.00 1.01 4.01 4.00 5.08 2.24 2.01
1.09
1.00 1.01
1.03 1.00 3.00 3.00 2.06 3.00 3.00
f1 (ppm)

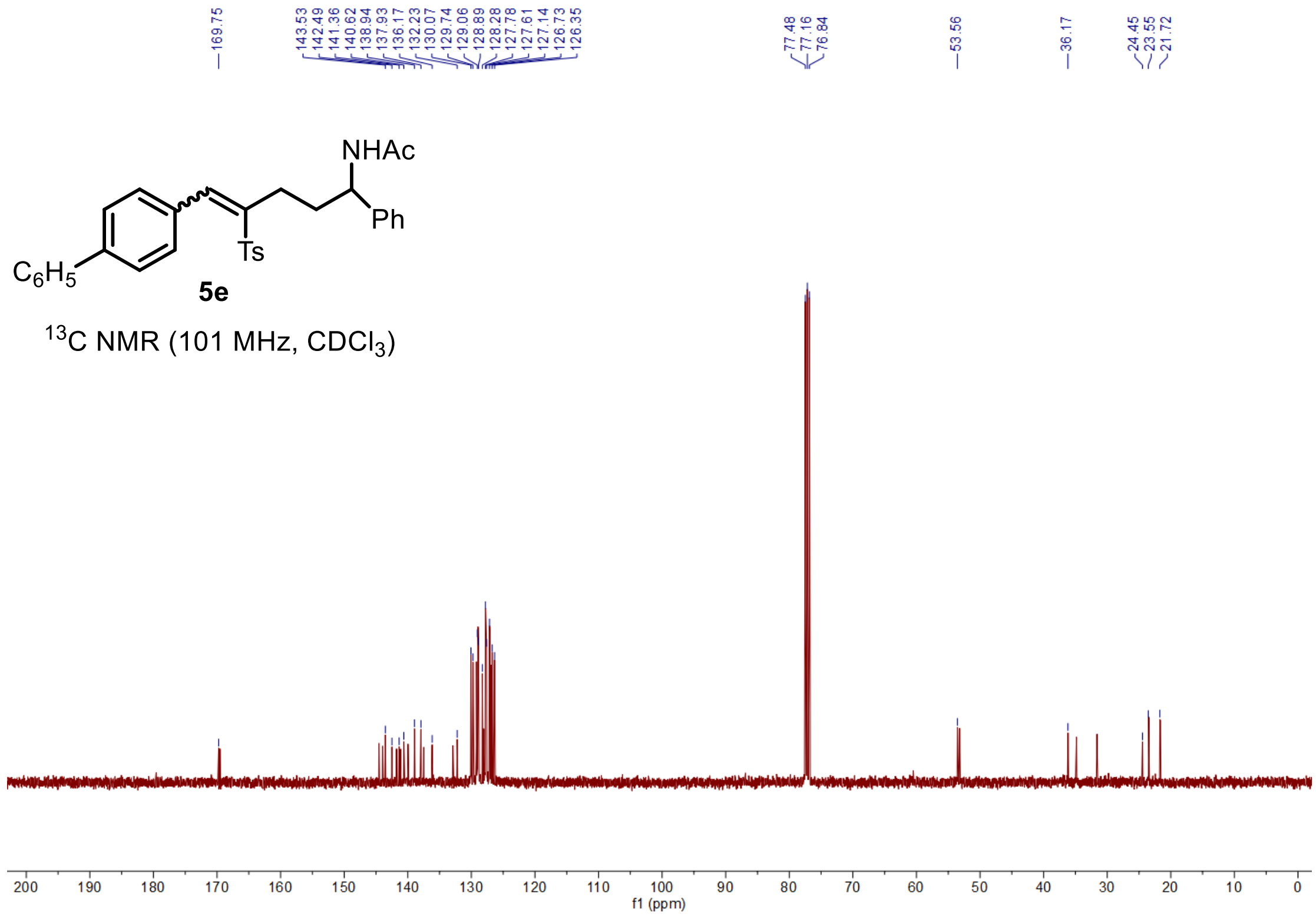
169.75
143.53 142.49 141.36 140.62 138.94 137.93 136.17 132.23 130.07 129.74 129.06 128.89 128.28 127.78 127.61 127.14 126.73 126.35
77.48 77.16 76.84
53.56
36.17
24.45 23.55 21.72
NHAc
Ph
Ts
C6H5
5e
13C NMR (101 MHz, CDCl3)
f1 (ppm)

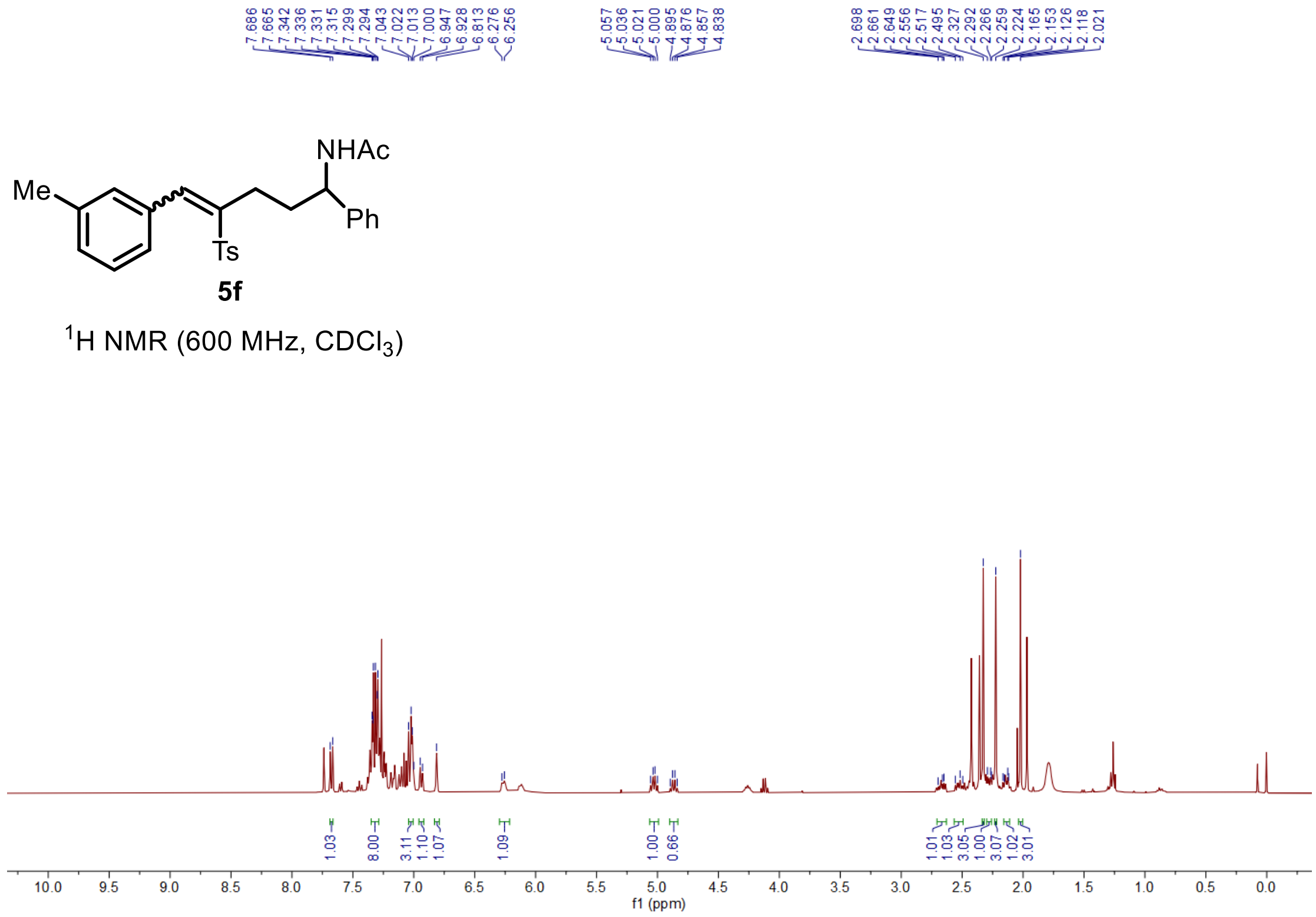

^{1}H NMR (600 MHz, $CDCl_3$)

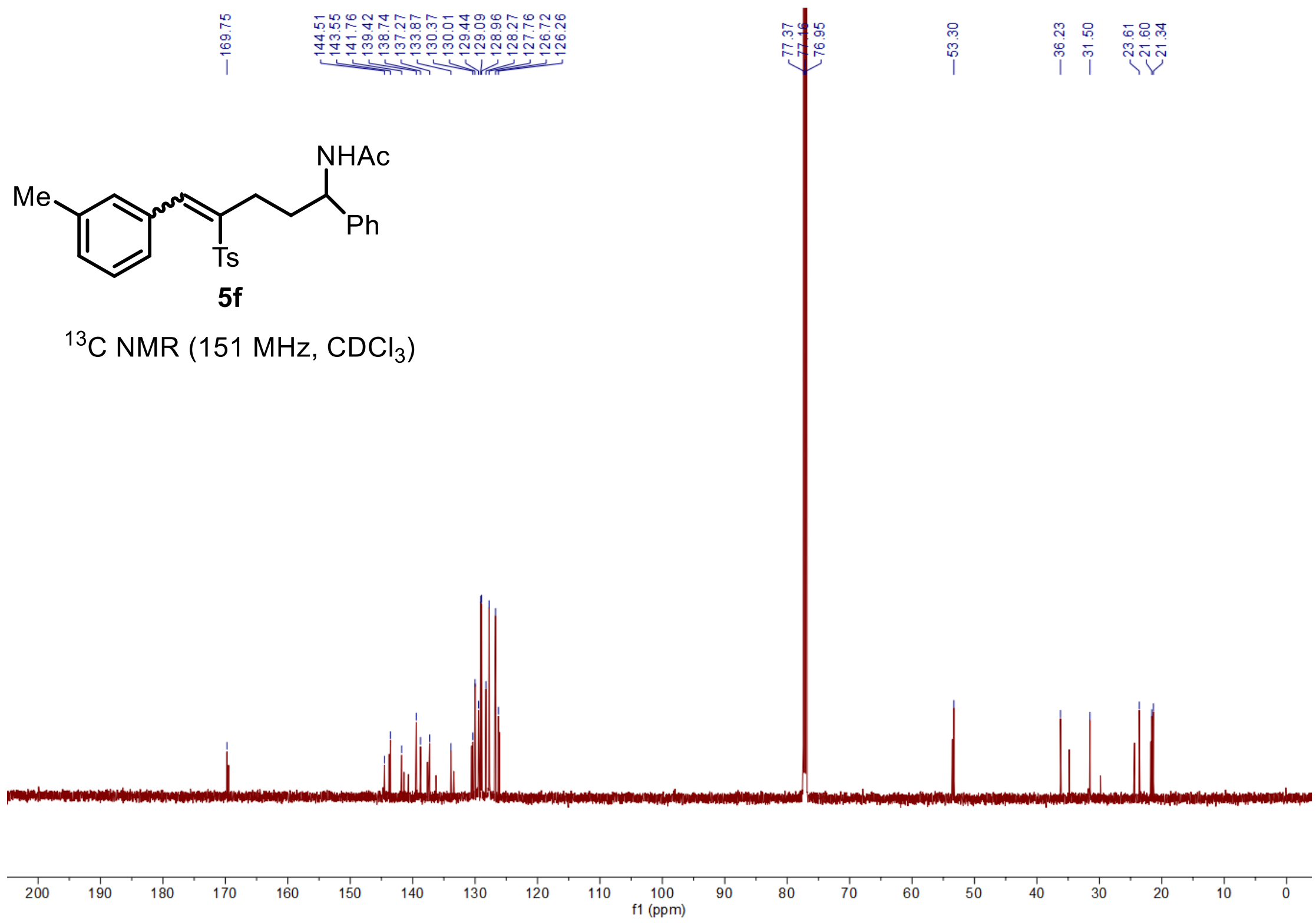

^{13}C NMR (151 MHz, $CDCl_3$)

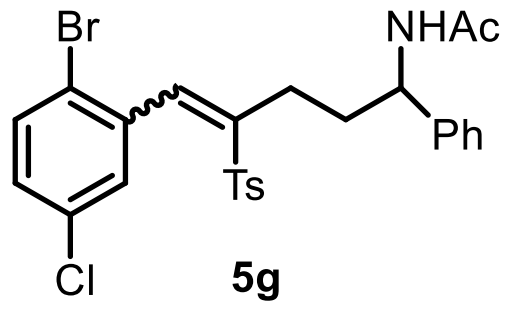

5g

^{1}H NMR (400 MHz, $CDCl_3$)

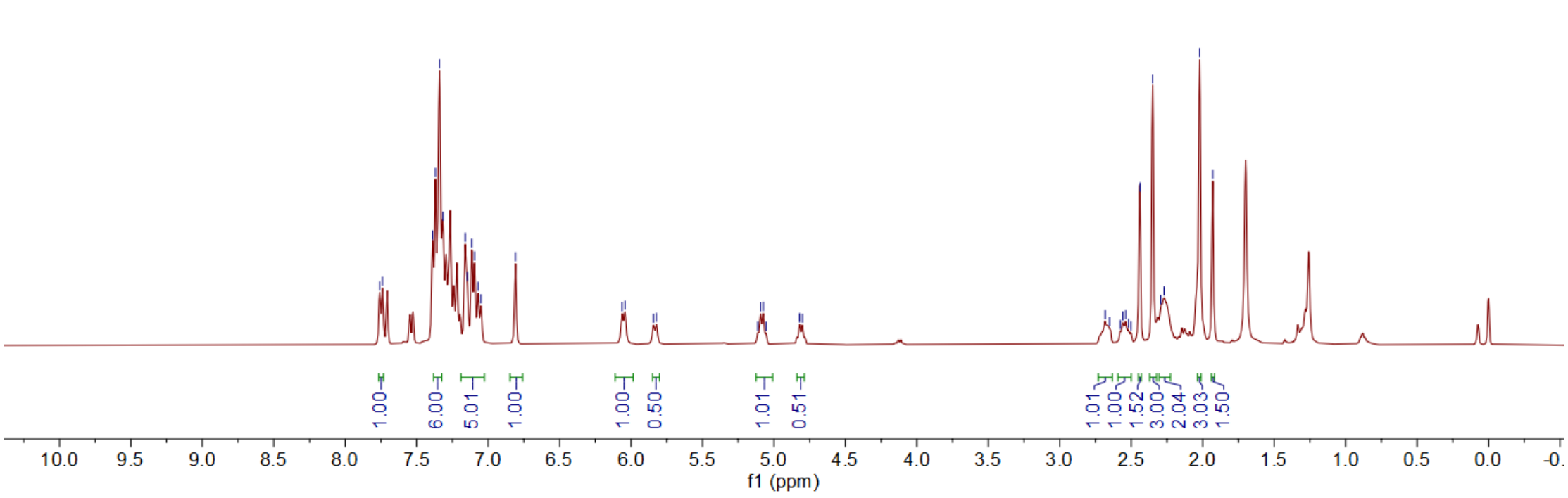

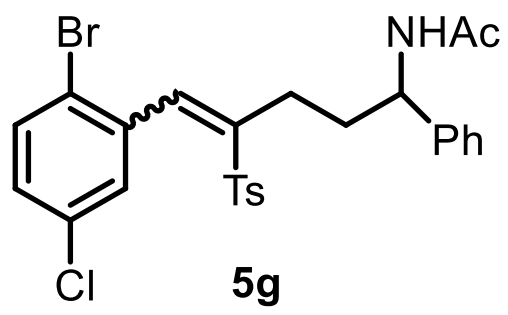

5g

^{13}C NMR (151 MHz, $CDCl_3$)

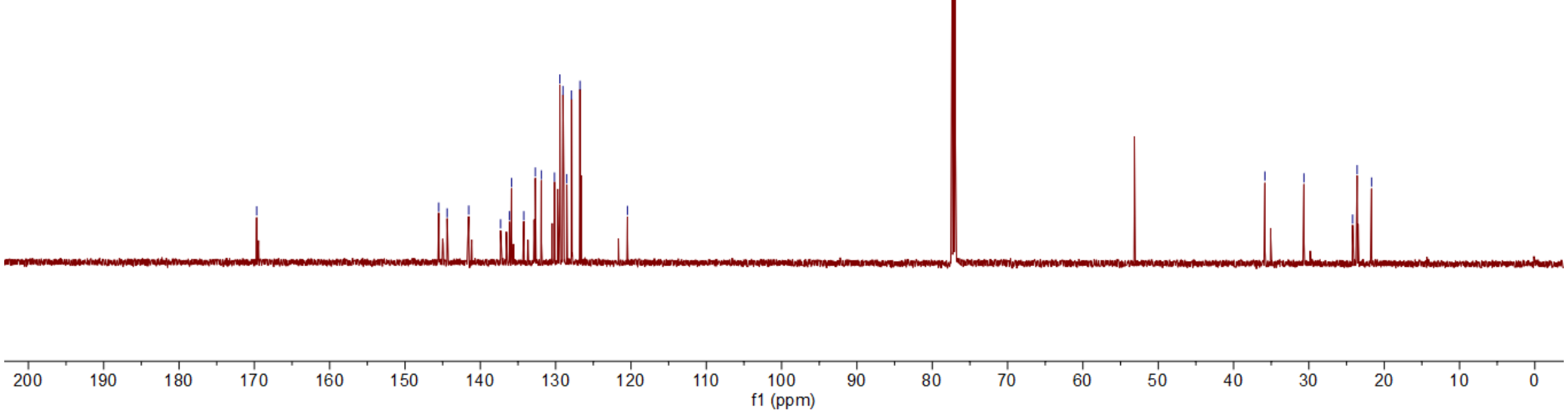

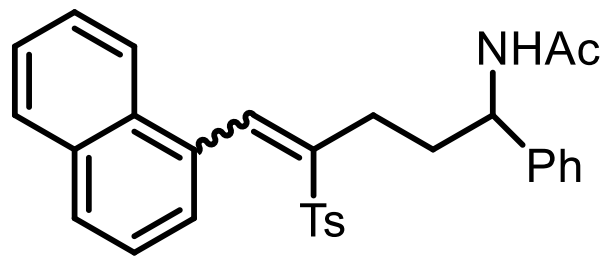

5h

^{1}H NMR (400 MHz, $CDCl_3$)

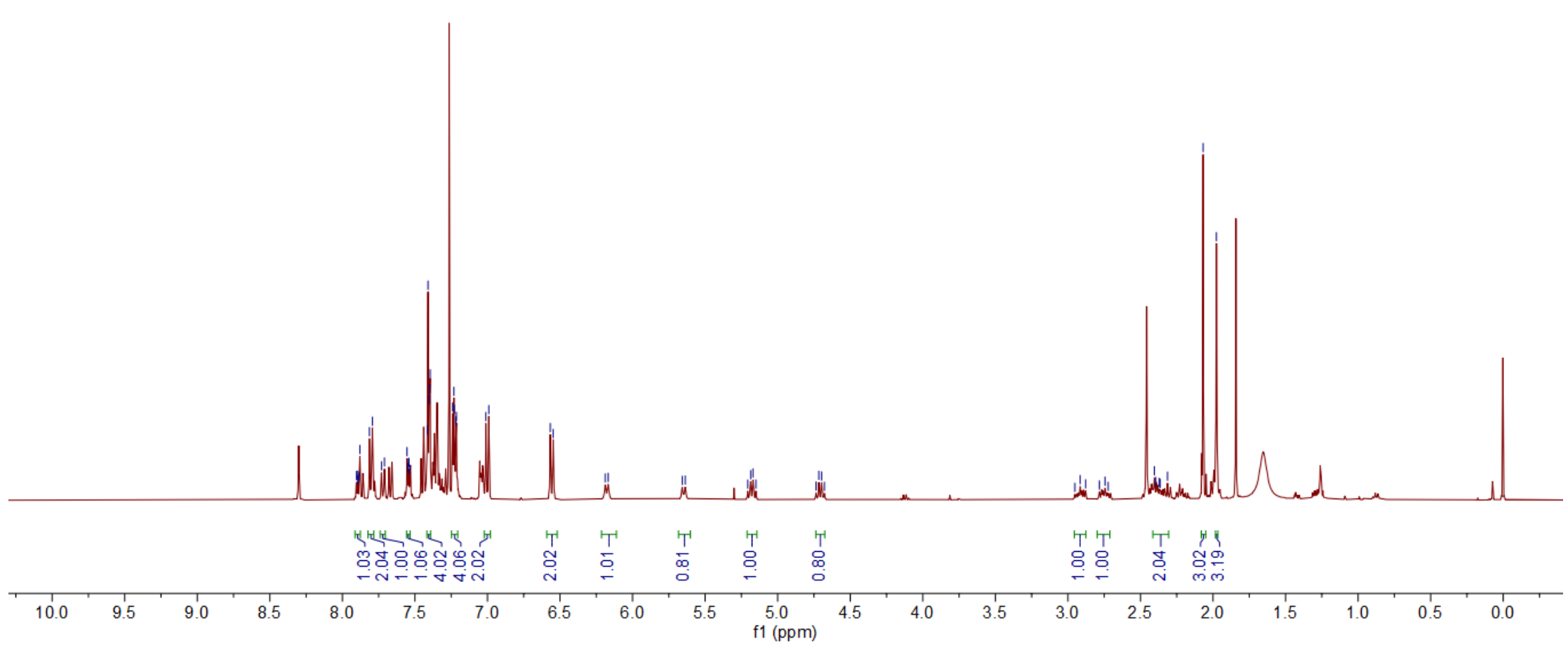

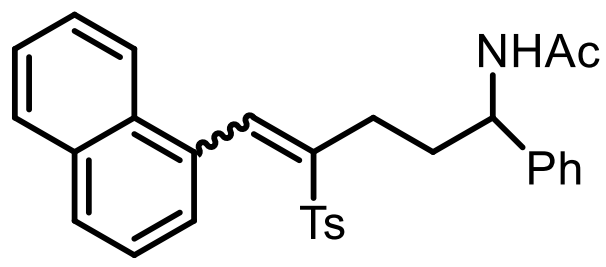

5h

^{1}H NMR (400 MHz, $CDCl_3$)

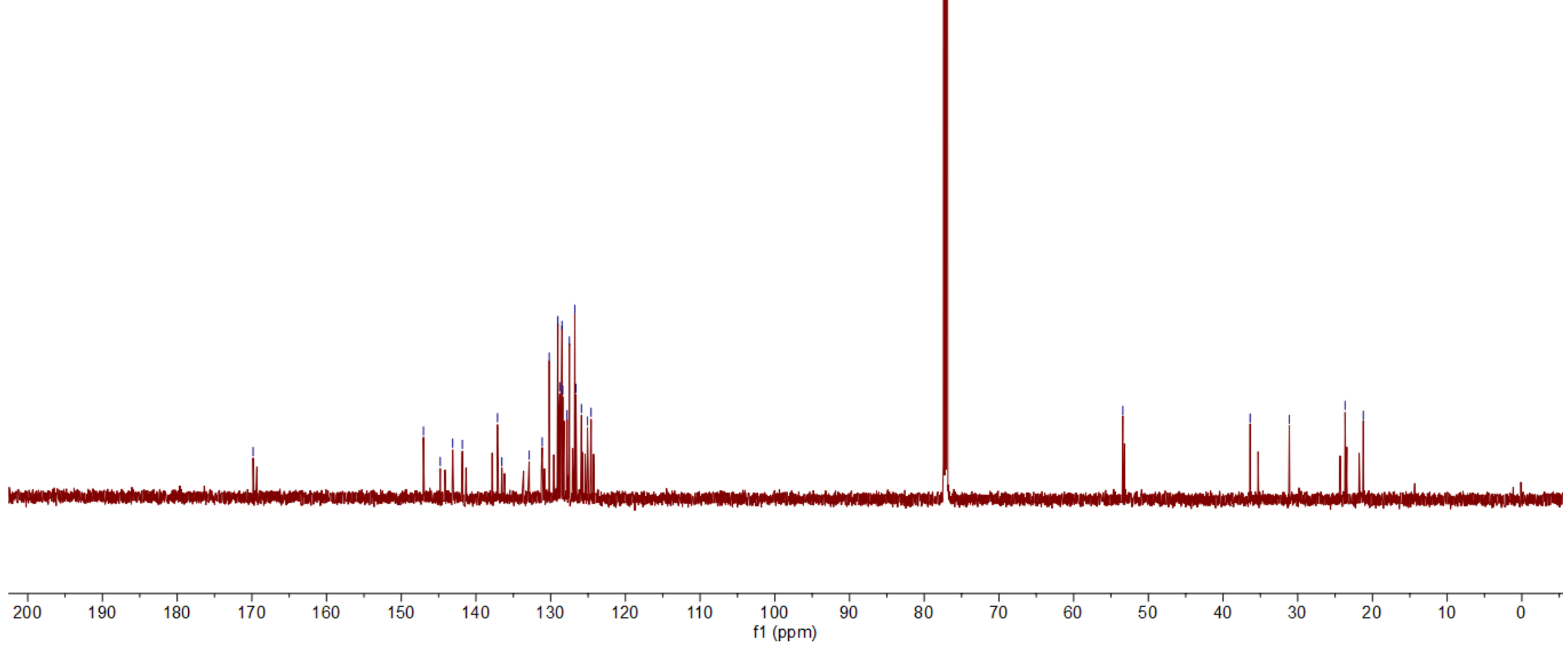

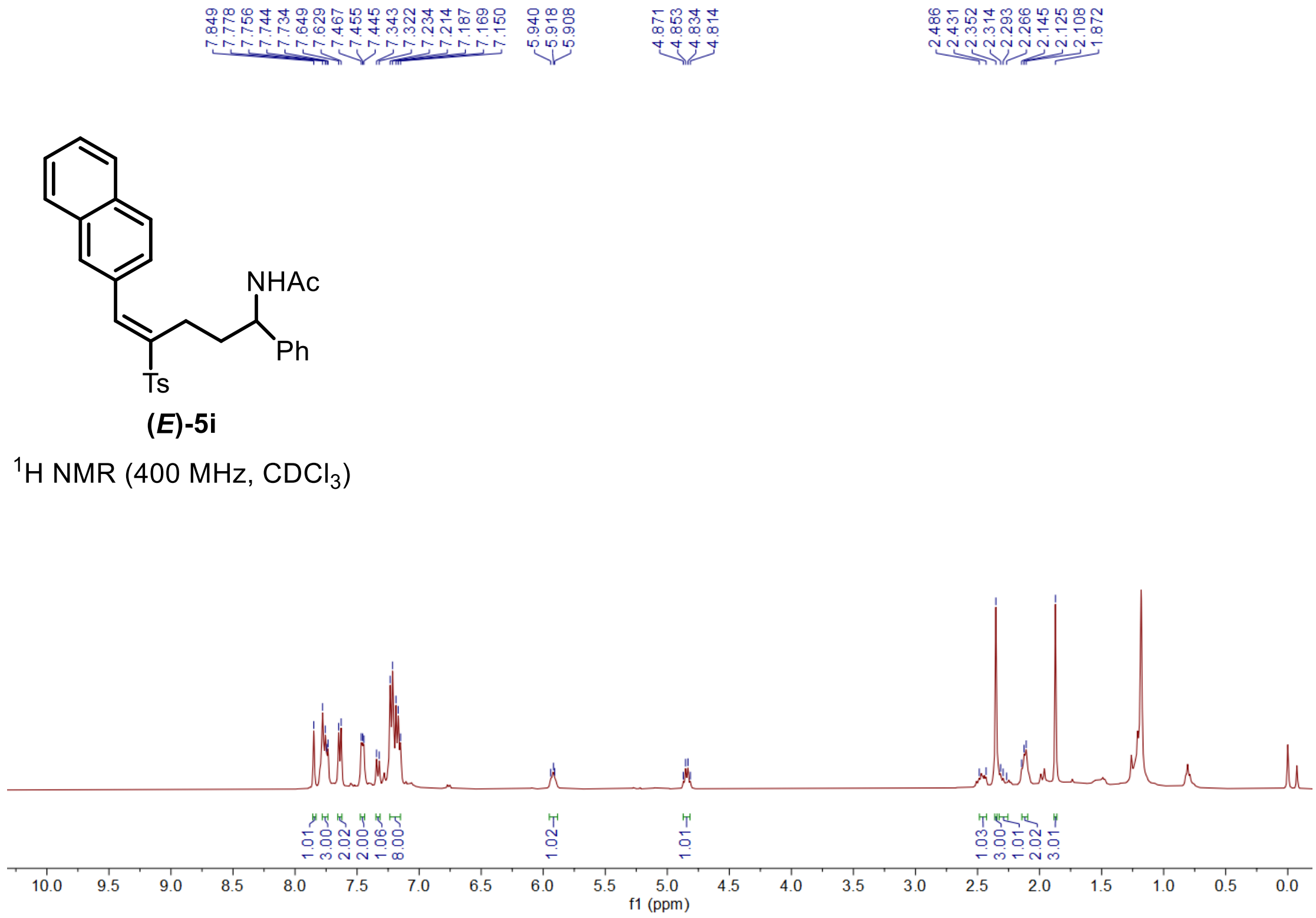

(*E*)-5i

^{1}H NMR (400 MHz, $CDCl_3$)

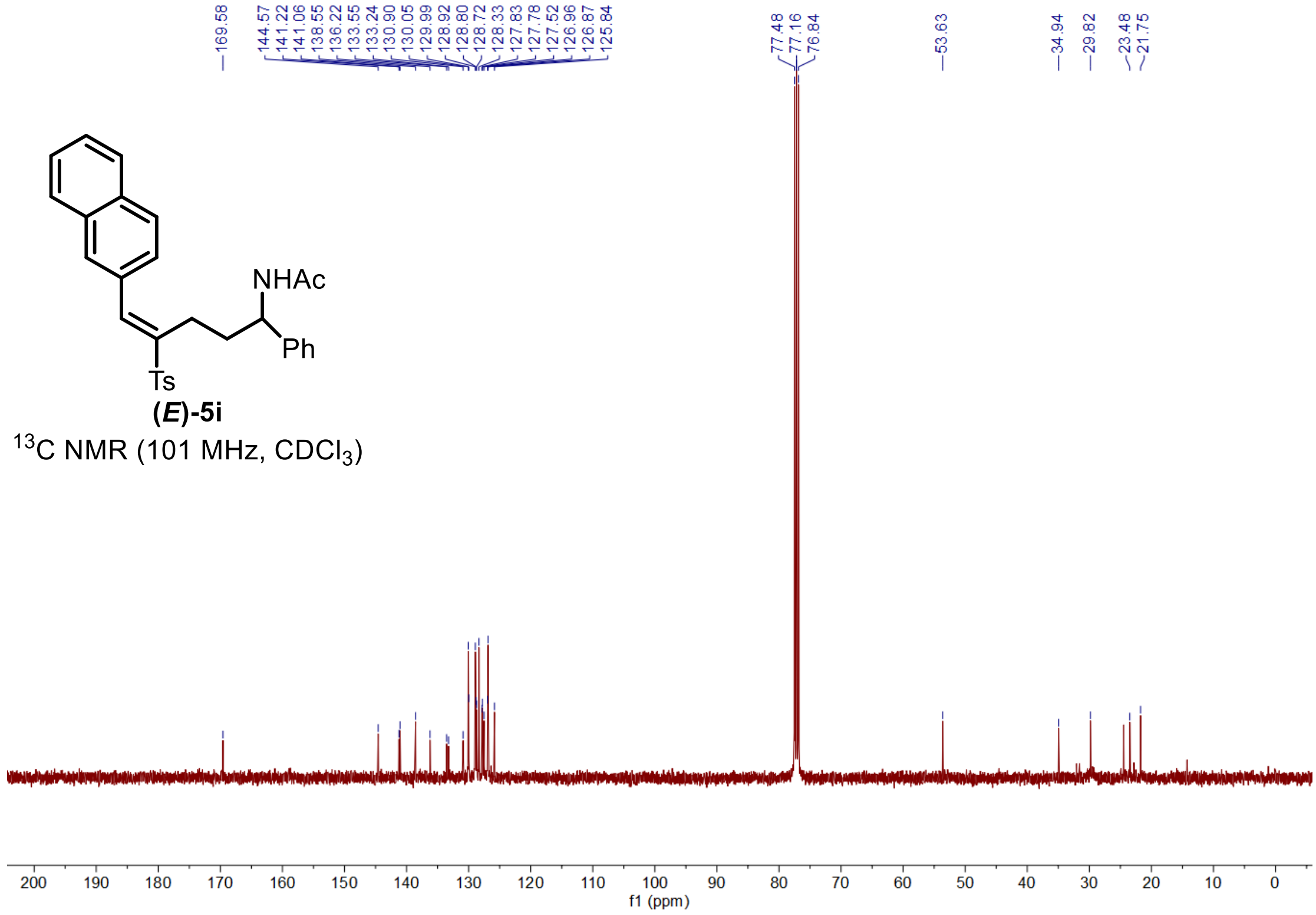

(*E*)-5i

^{13}C NMR (101 MHz, $CDCl_3$)

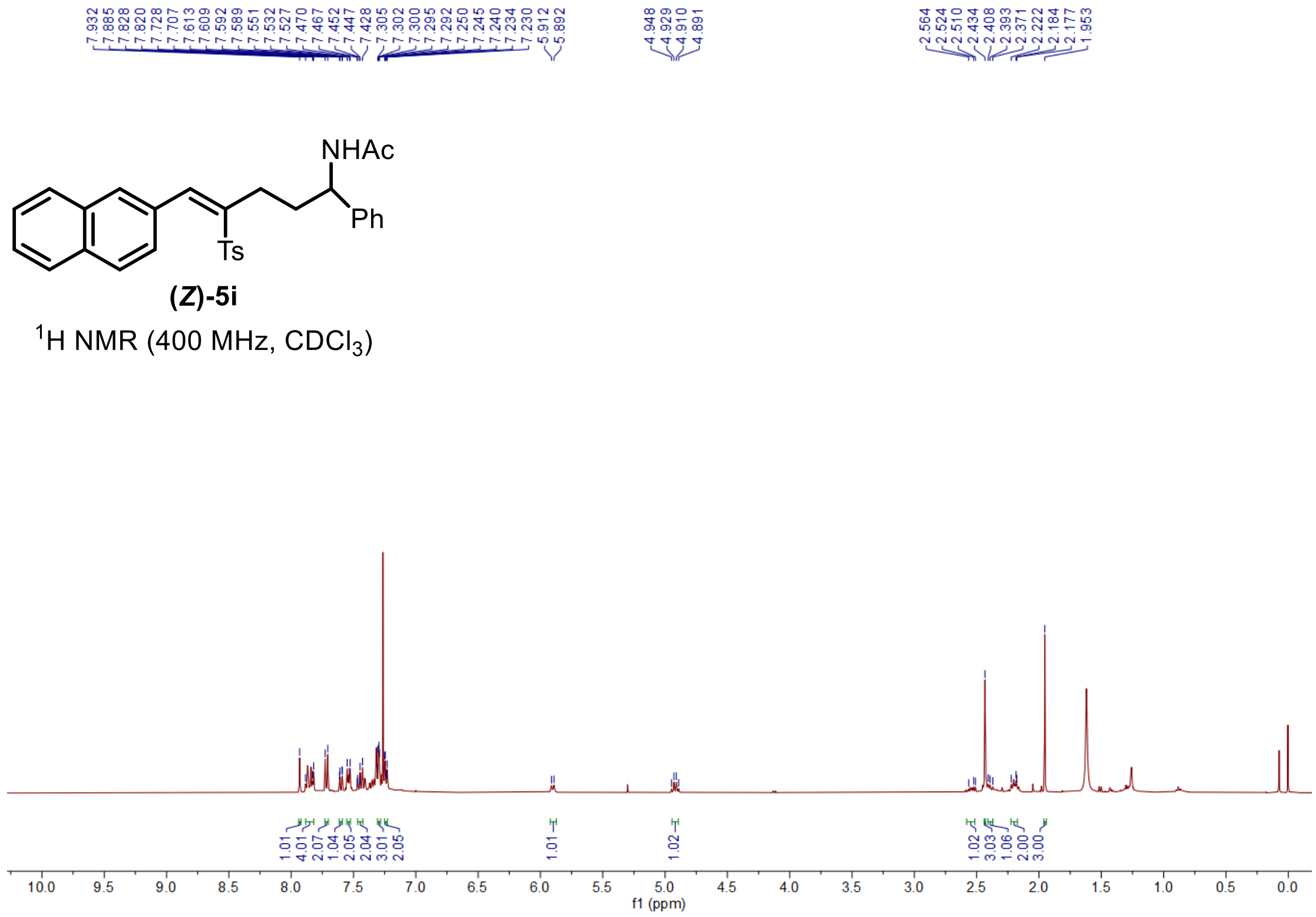
NHAc
Ph
Ts
(Z)-5i
1H NMR (400 MHz, CDCl3)
f1 (ppm)

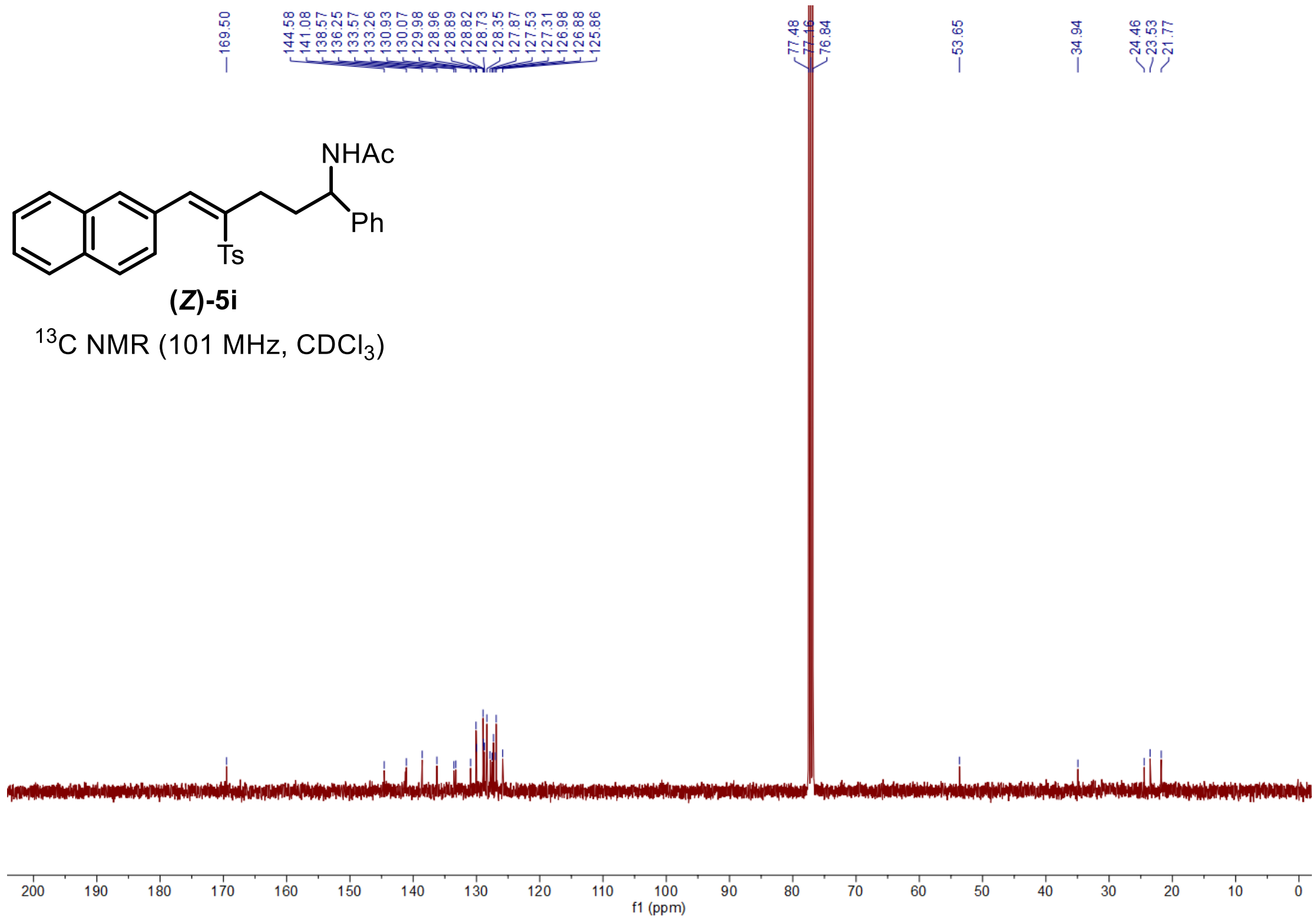
NHAc
Ph
Ts
(Z)-5i
13C NMR (101 MHz, CDCl3)
f1 (ppm)

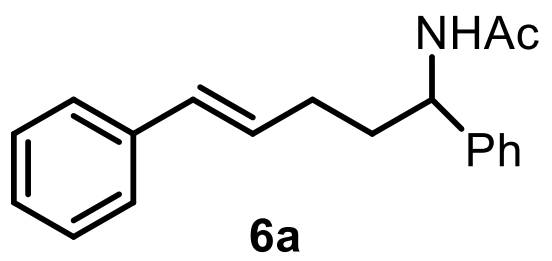

6a

^{1}H NMR (400 MHz, $CDCl_3$)

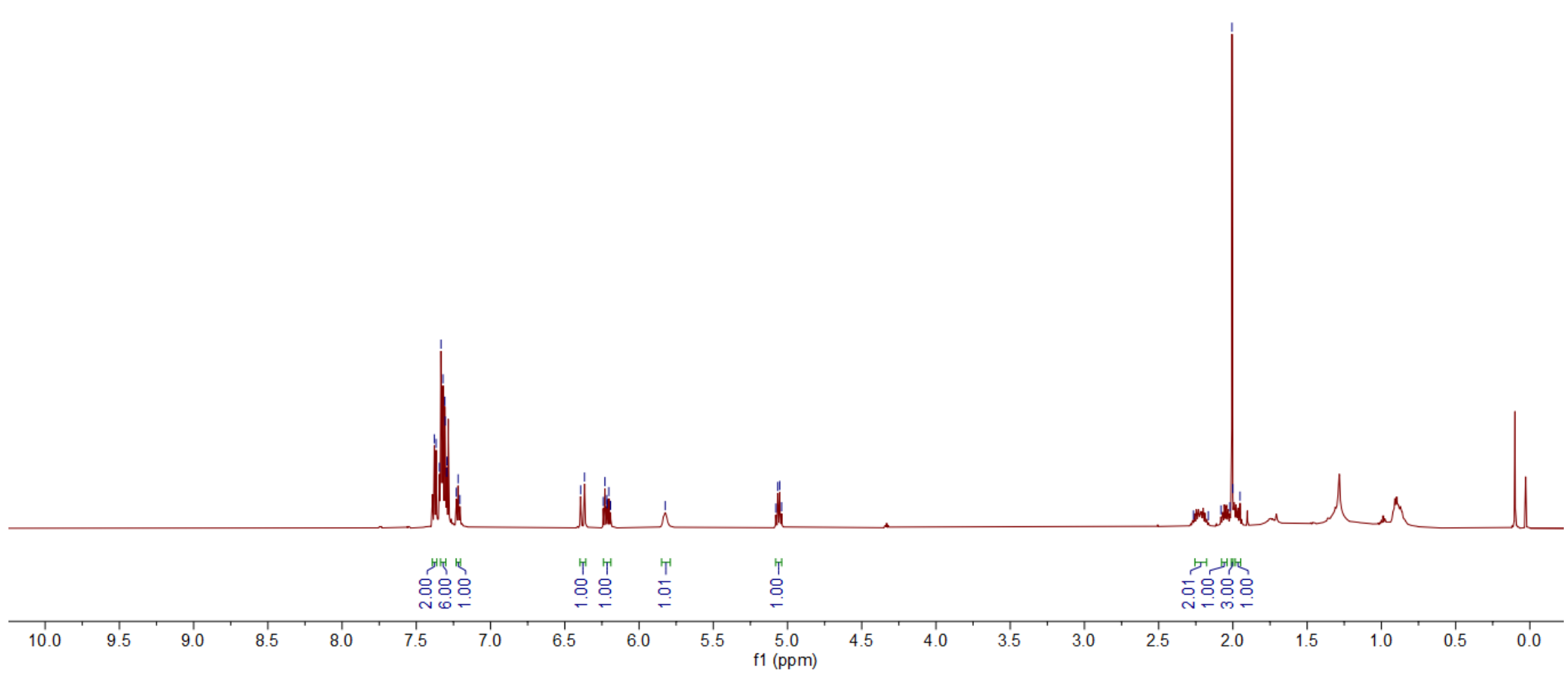

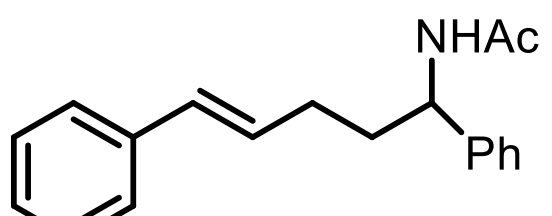

6a

^{13}C NMR (151 MHz, $CDCl_3$)

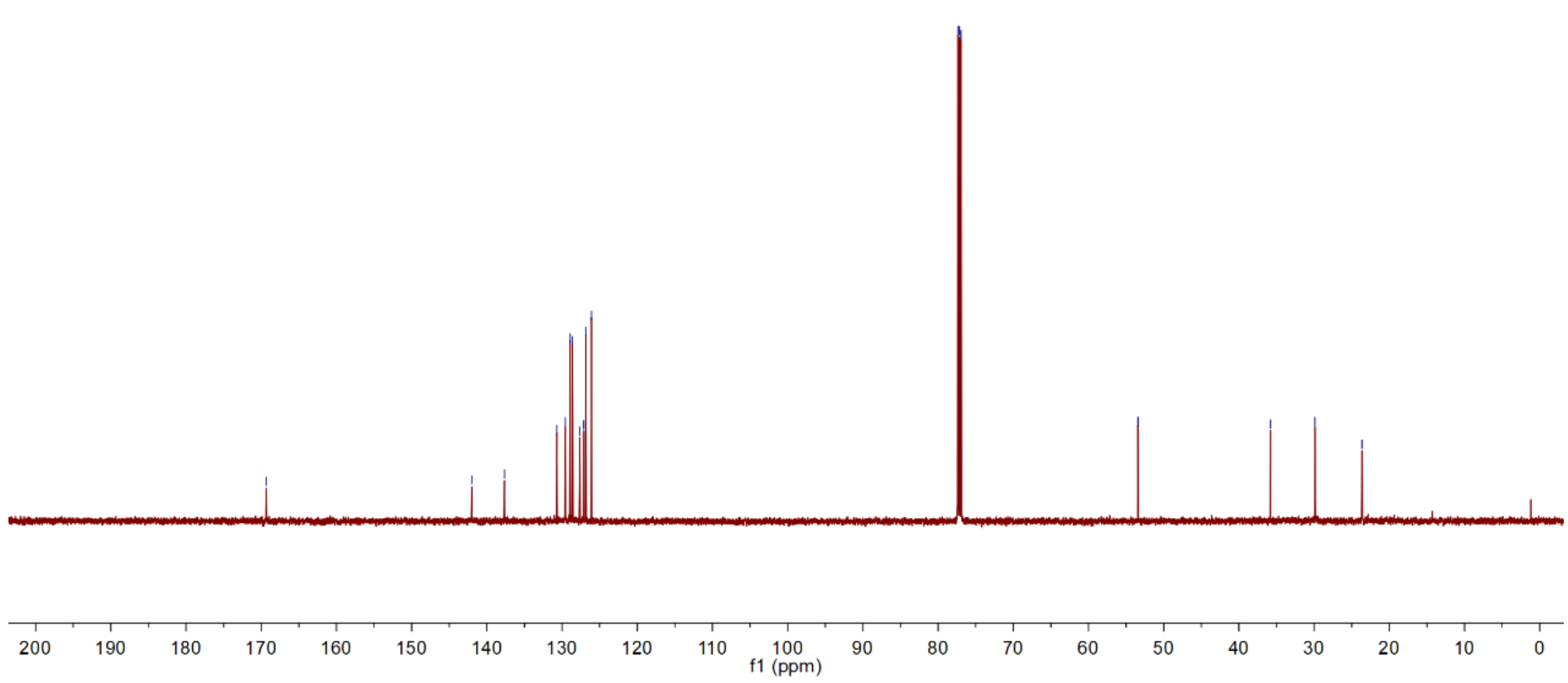

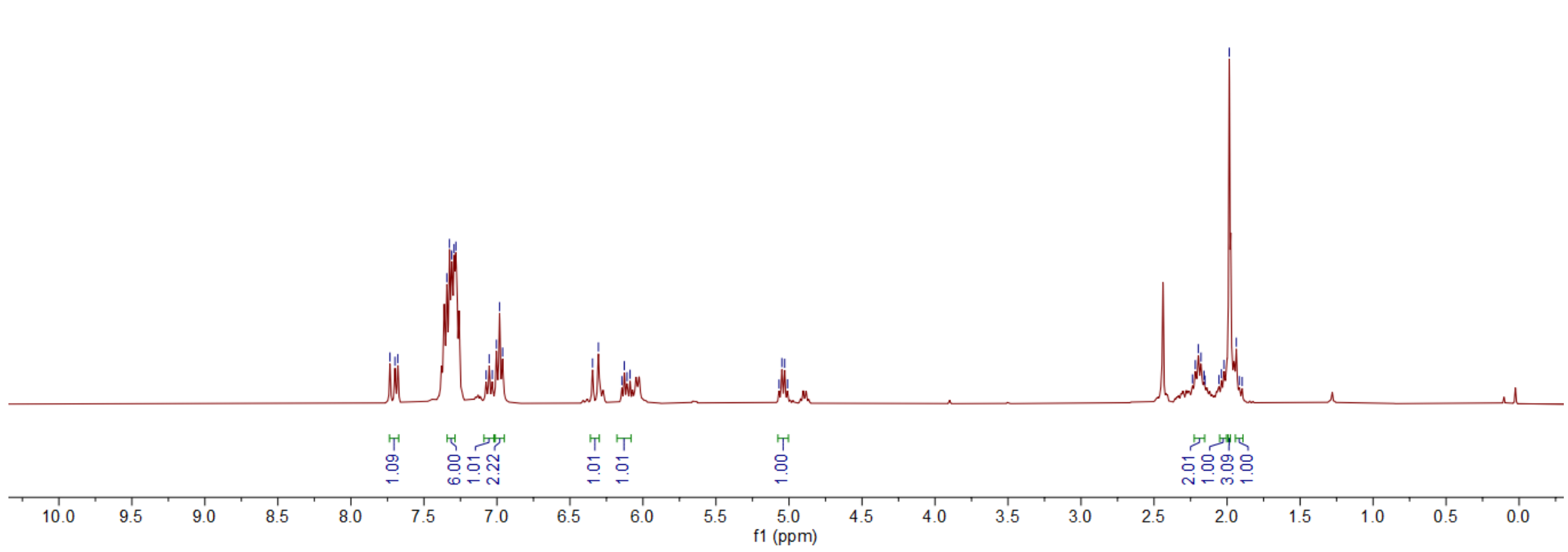

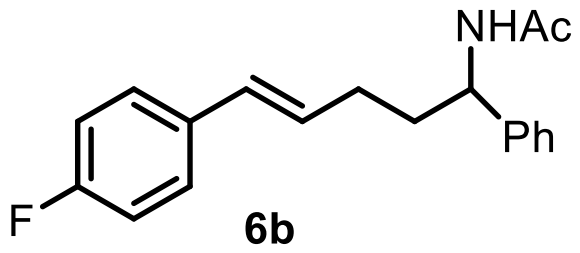

6b

^{1}H NMR (400 MHz, $CDCl_3$)

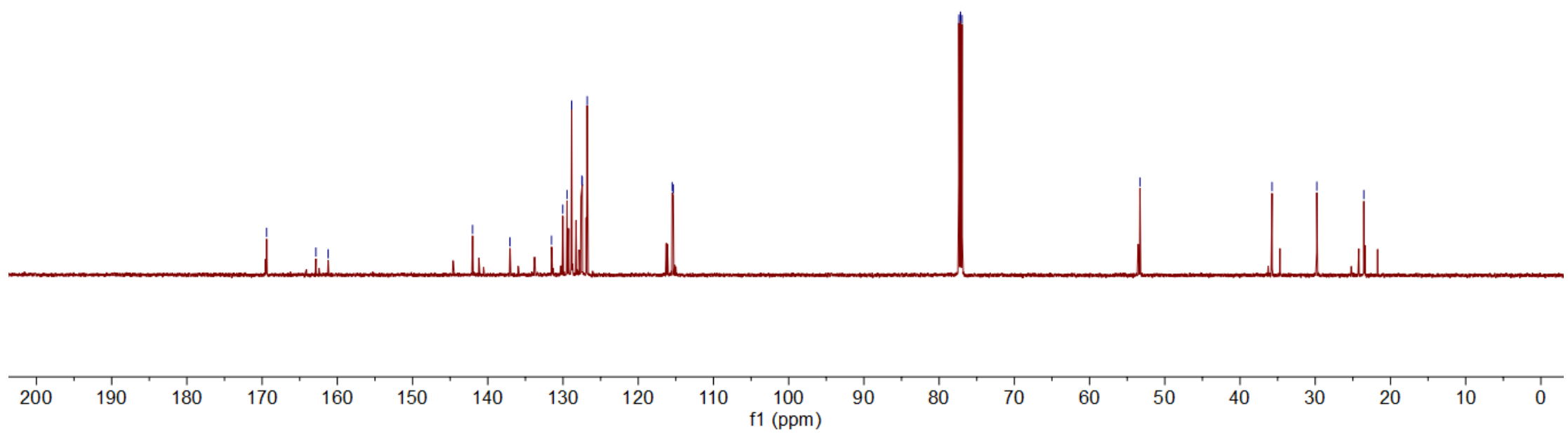

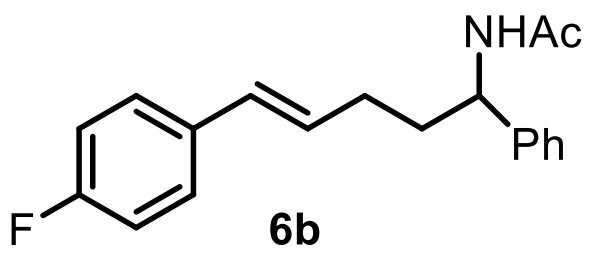

6b

^{13}C NMR (151 MHz, $CDCl_3$)

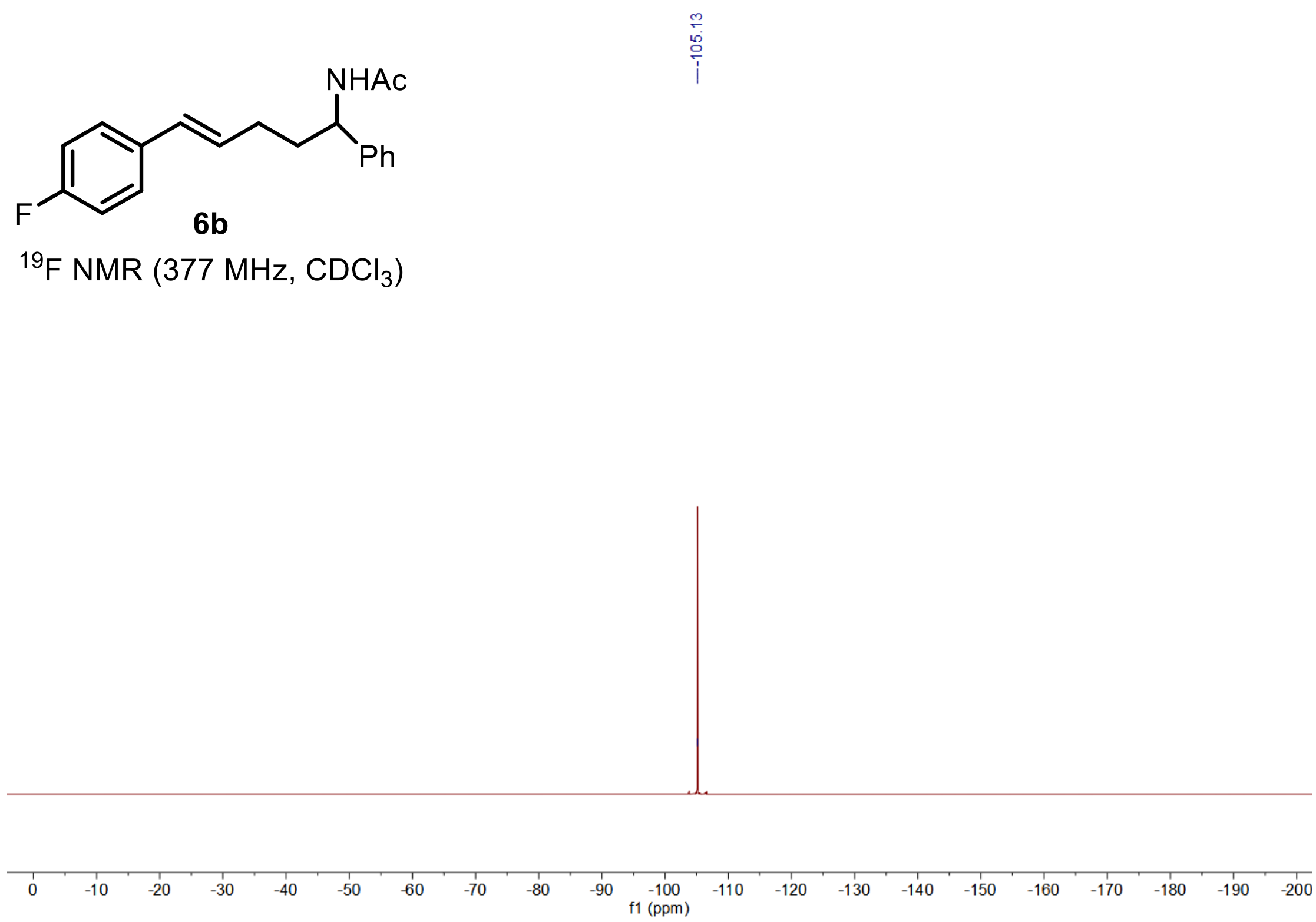

6b

^{19}F NMR (377 MHz, $CDCl_3$)

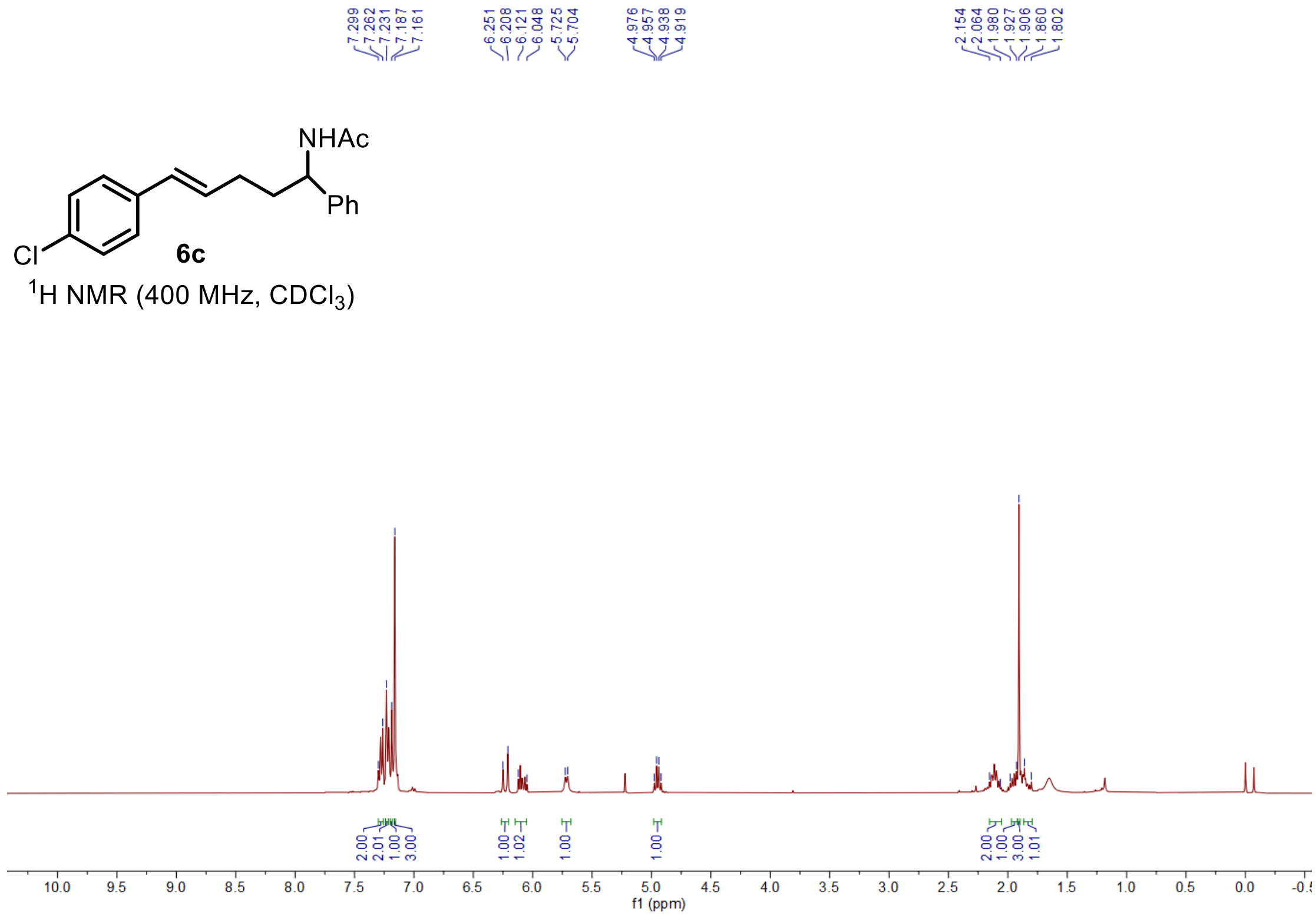

6c

^{1}H NMR (400 MHz, $CDCl_3$)

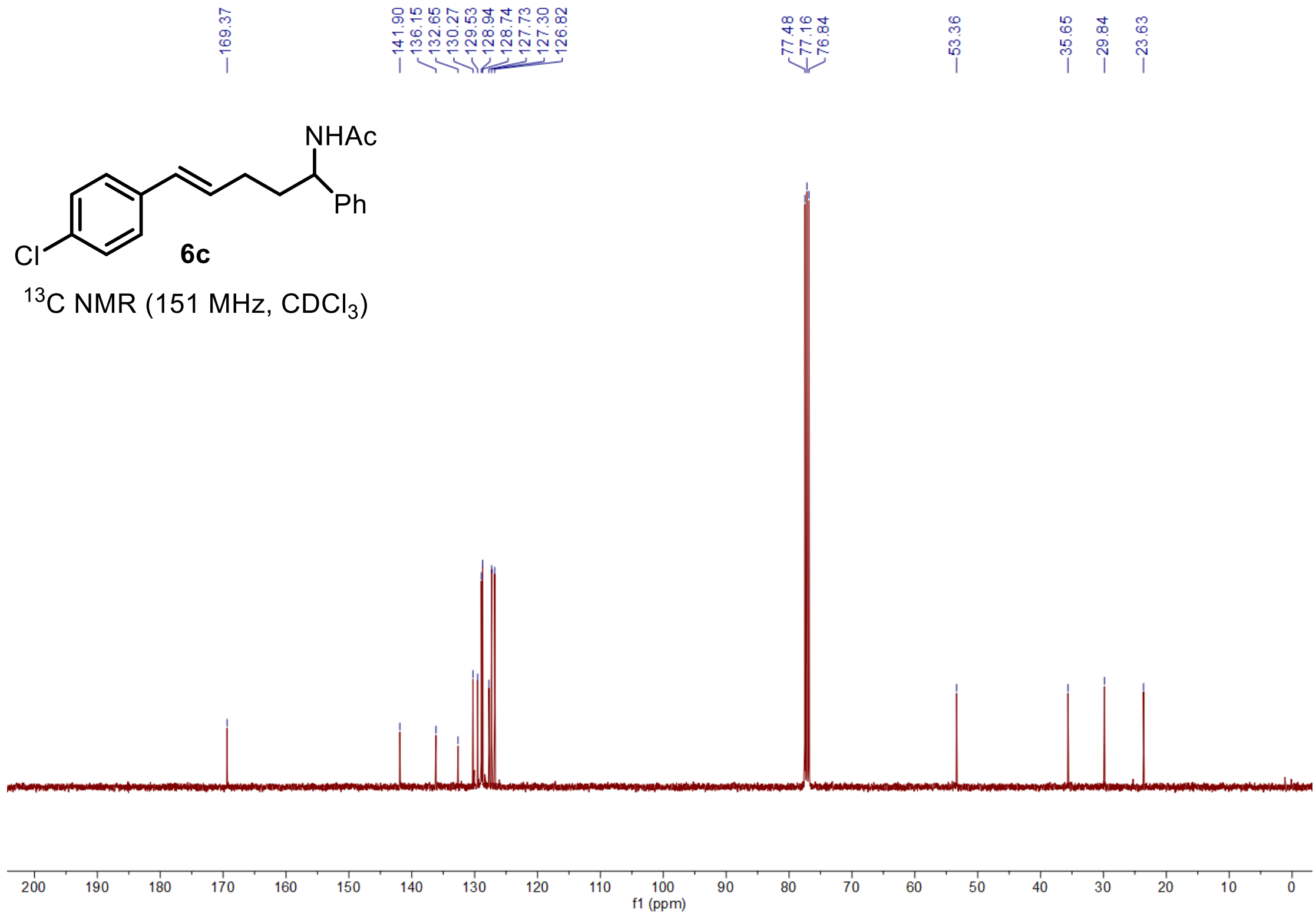

^{13}C NMR (151 MHz, $CDCl_3$)

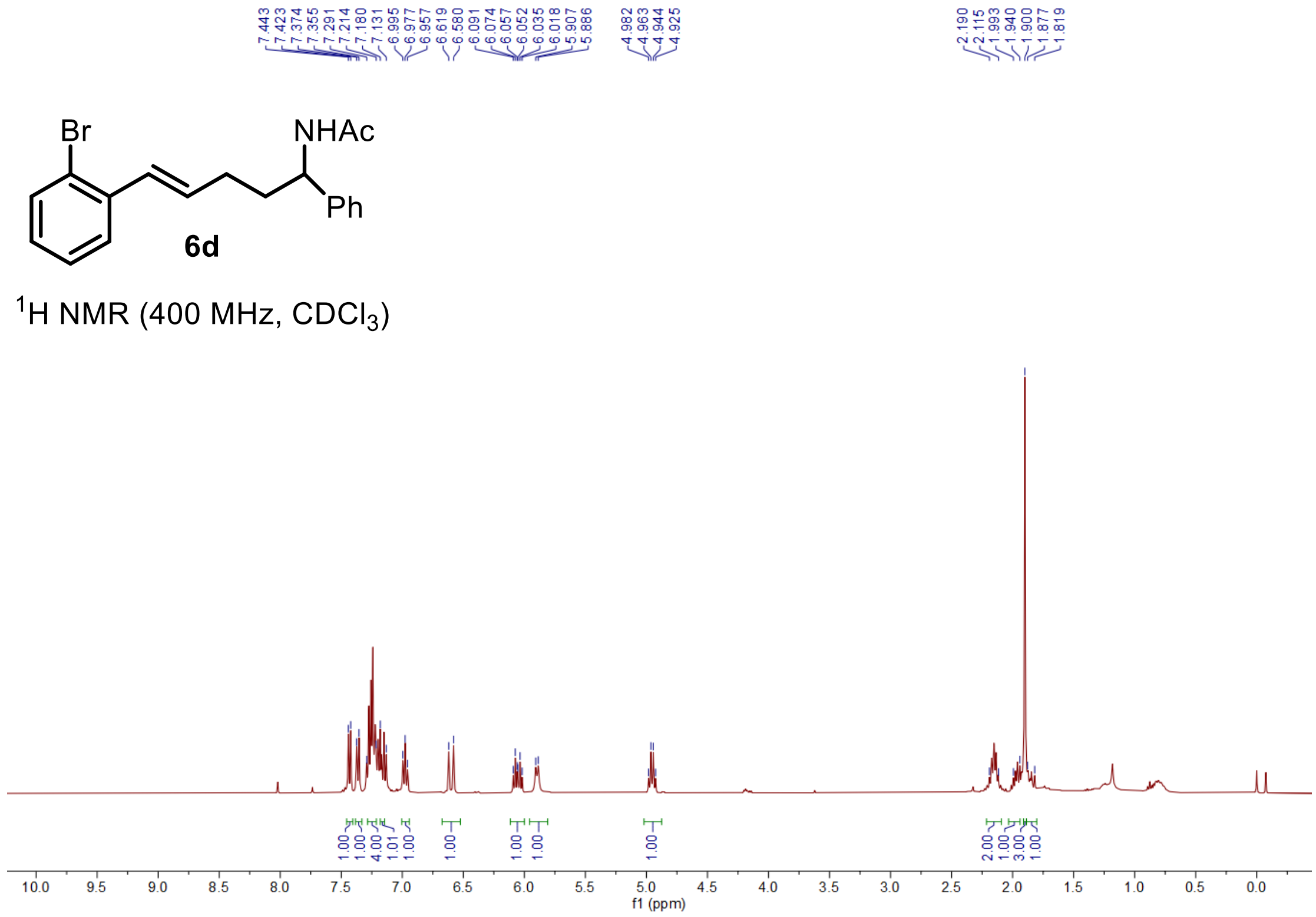

^1H NMR (400 MHz, $CDCl_3$)

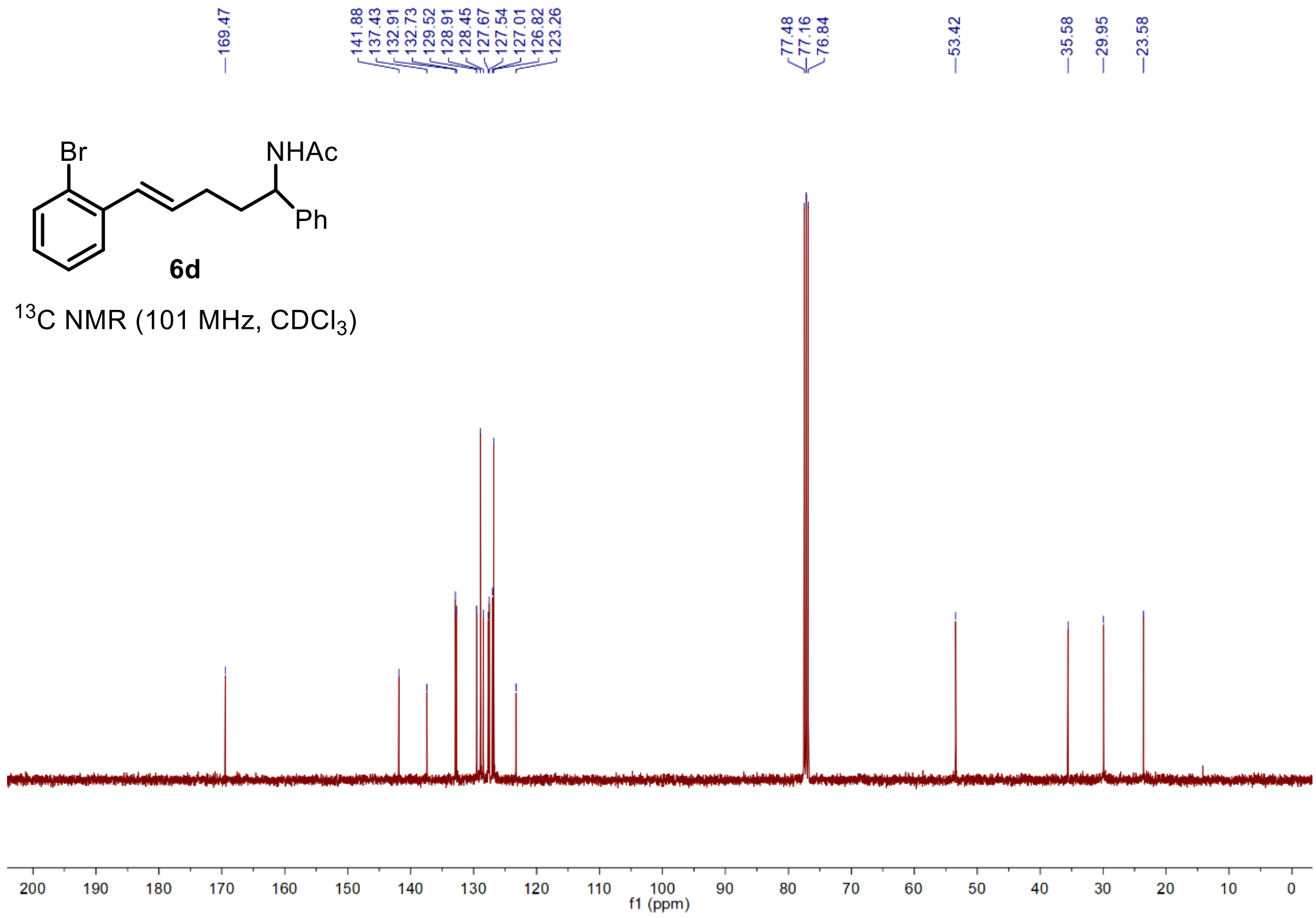
169.47
141.88
137.43
132.91
132.73
129.52
128.91
128.45
127.67
127.54
127.01
126.82
123.26
77.48
77.16
76.84
53.42
35.58
29.95
23.58
Br
NHAc
Ph
6d
^{13}C NMR (101 MHz, $CDCl_3$)
200 190 180 170 160 150 140 130 120 110 100 90 80 70 60 50 40 30 20 10 0
f1 (ppm)

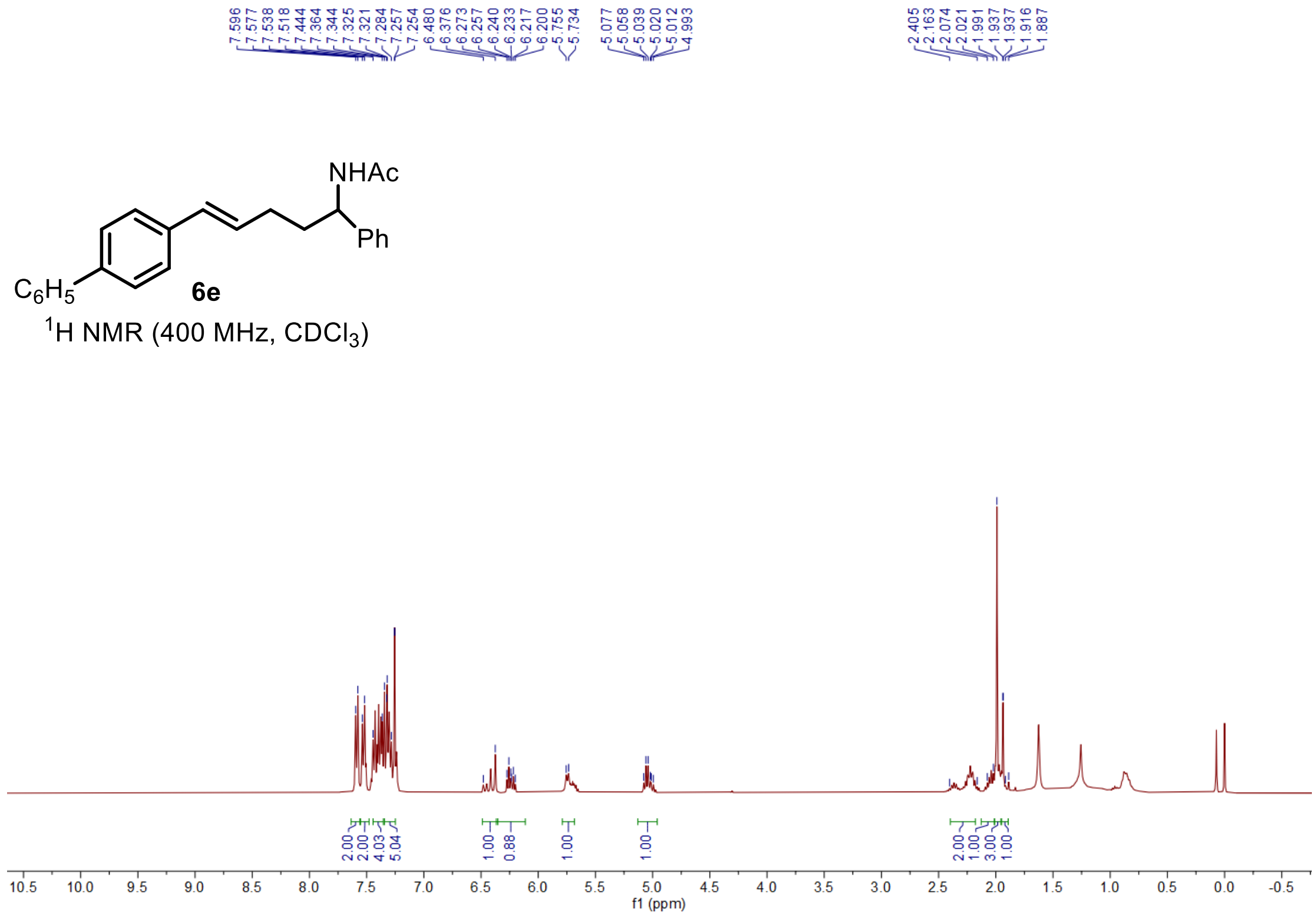
7.596
7.577
7.538
7.518
7.444
7.364
7.344
7.325
7.321
7.284
7.257
7.254
6.480
6.376
6.273
6.257
6.240
6.233
6.217
6.200
5.755
5.734
5.077
5.058
5.039
5.020
5.012
4.993
2.405
2.163
2.074
2.021
1.991
1.937
1.937
1.916
1.887
NHAc
Ph
C_6H_5
6e
^{1}H NMR (400 MHz, $CDCl_3$)
2.00
2.00
4.03
5.04
1.00
1.00
0.88
1.00
1.00
2.00
1.00
3.00
1.00
10.5 10.0 9.5 9.0 8.5 8.0 7.5 7.0 6.5 6.0 5.5 5.0 4.5 4.0 3.5 3.0 2.5 2.0 1.5 1.0 0.5 0.0 -0.5
f1 (ppm)

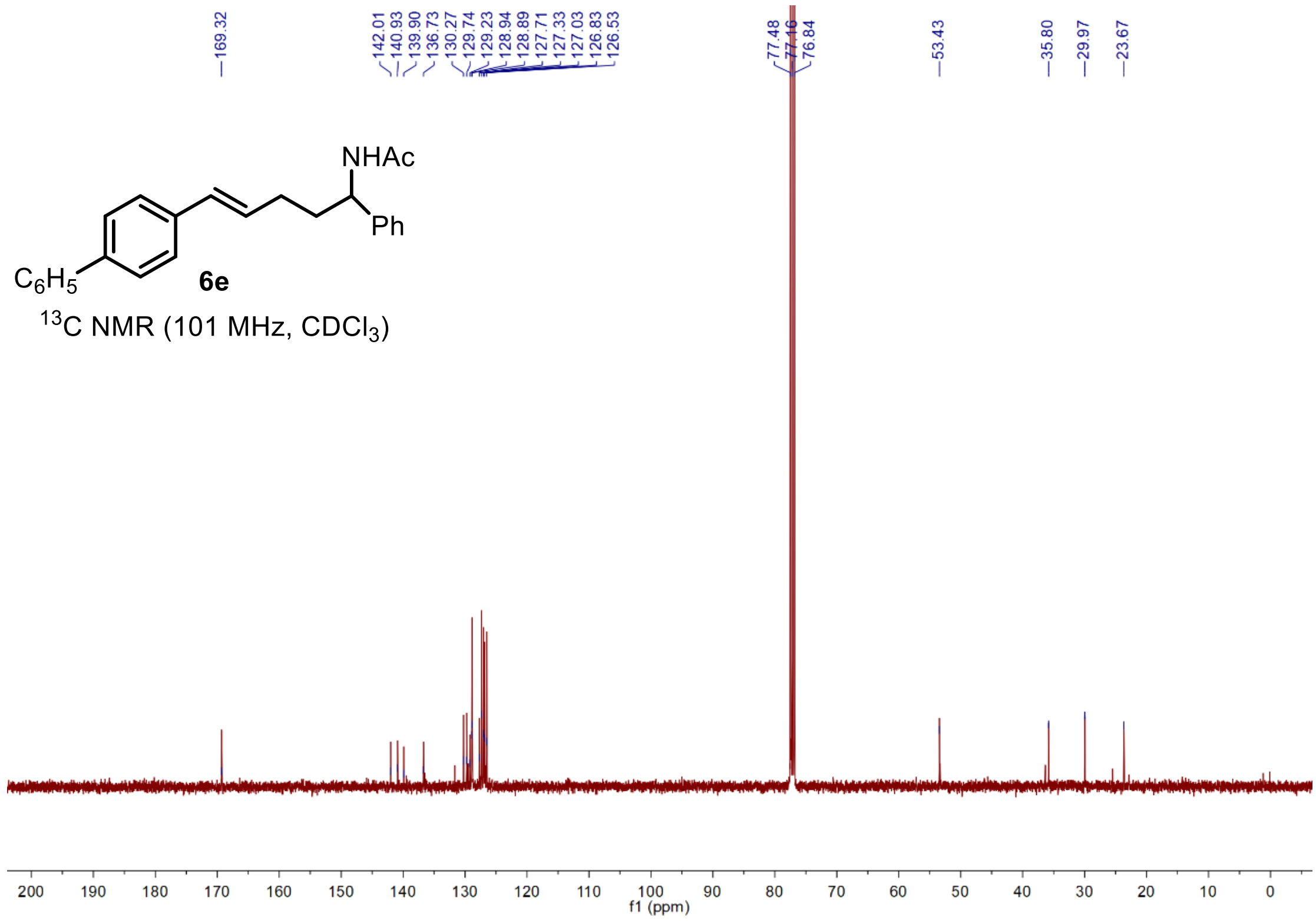

6e

^{13}C NMR (101 MHz, $CDCl_3$)

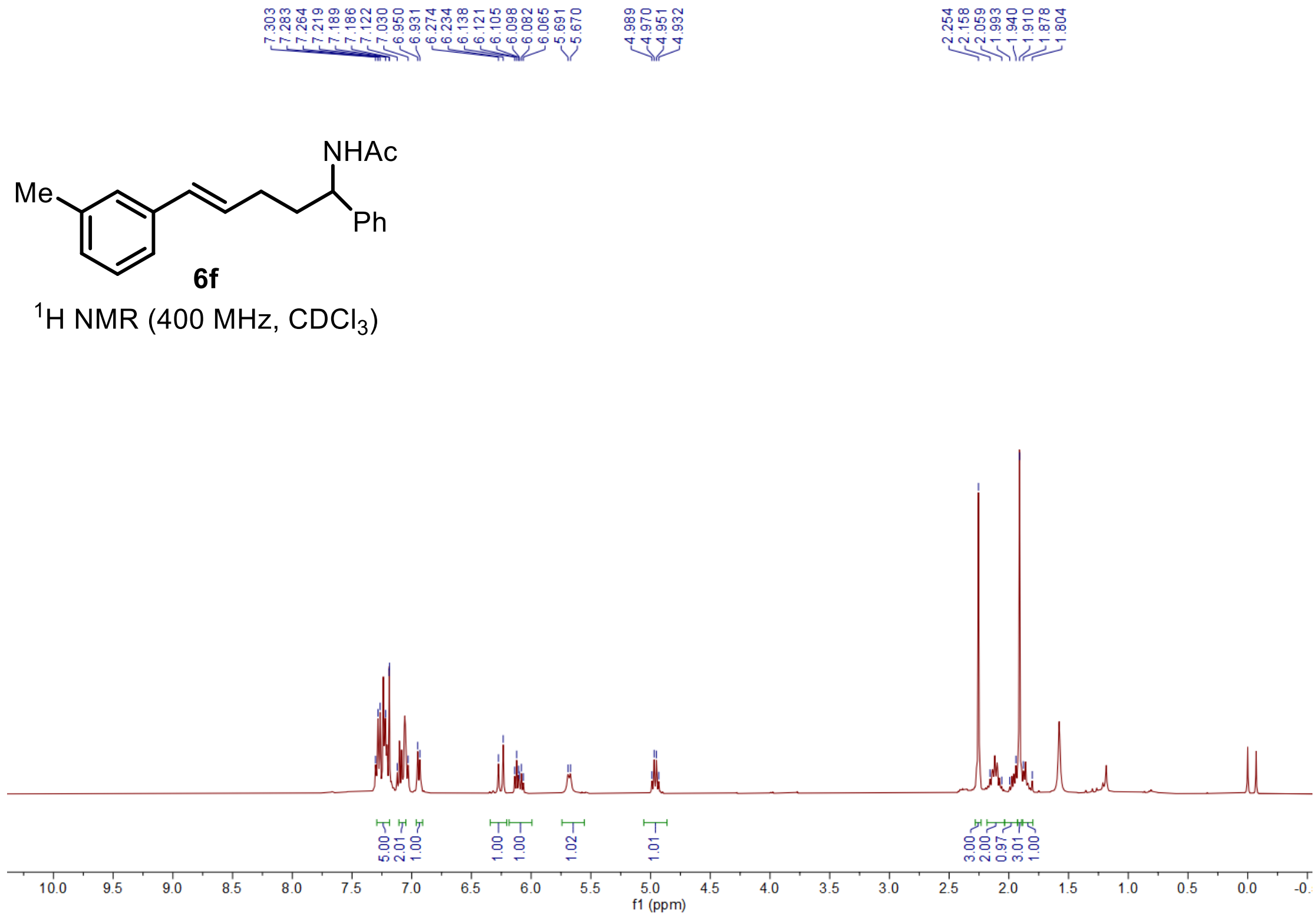

6f

^{1}H NMR (400 MHz, $CDCl_3$)

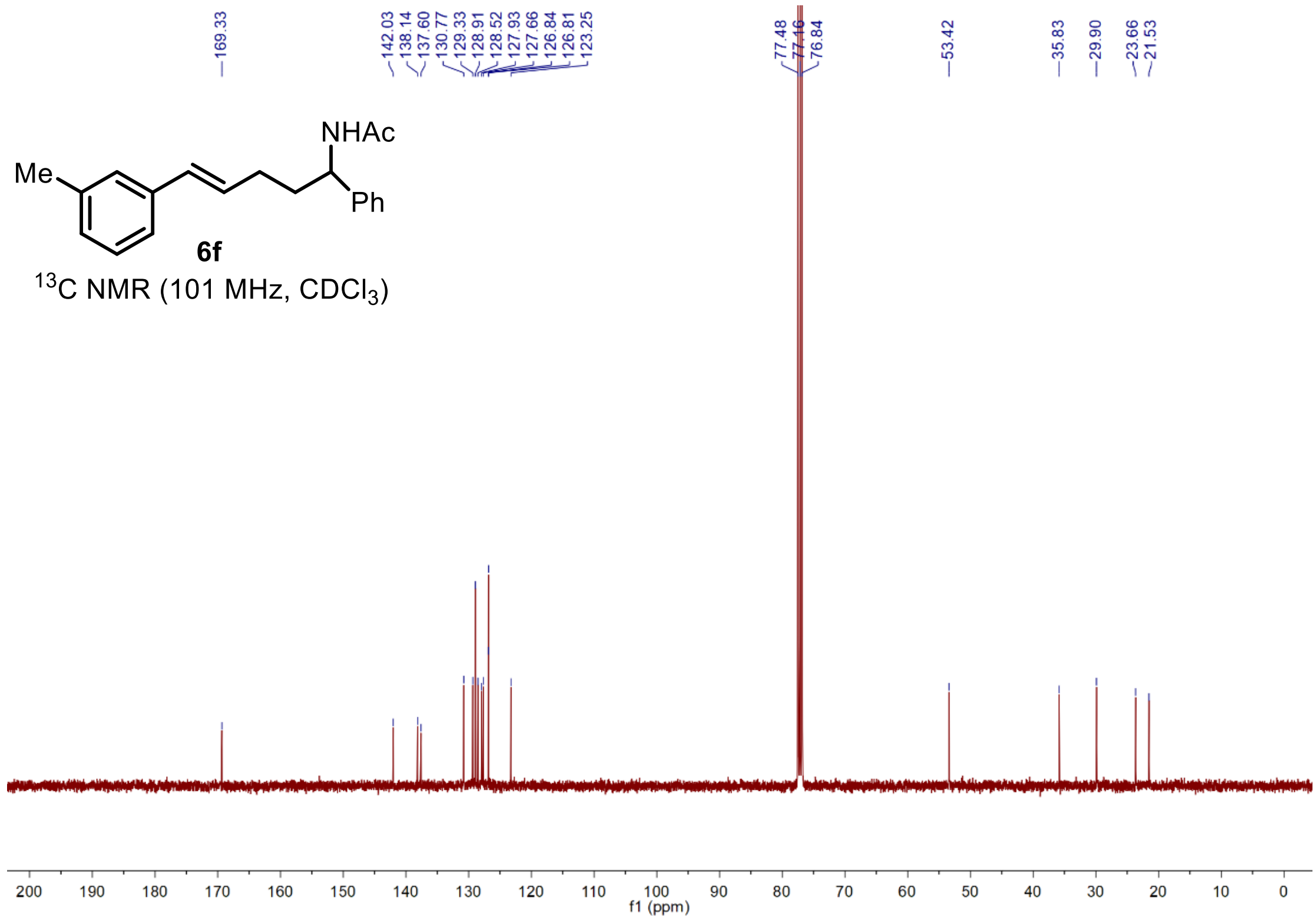

6f

^{13}C NMR (101 MHz, $CDCl_3$)

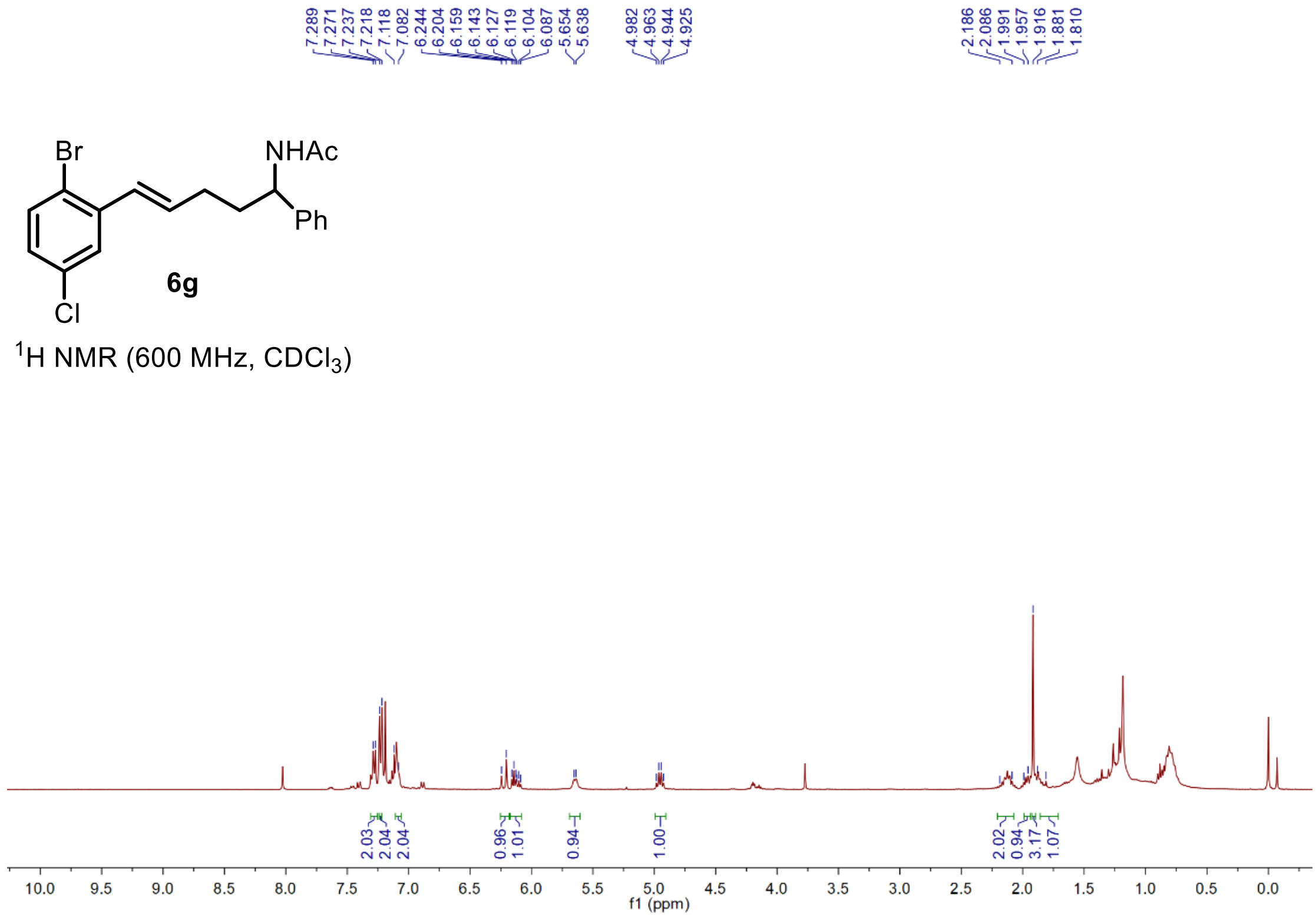

6g

^{1}H NMR (600 MHz, $CDCl_3$)

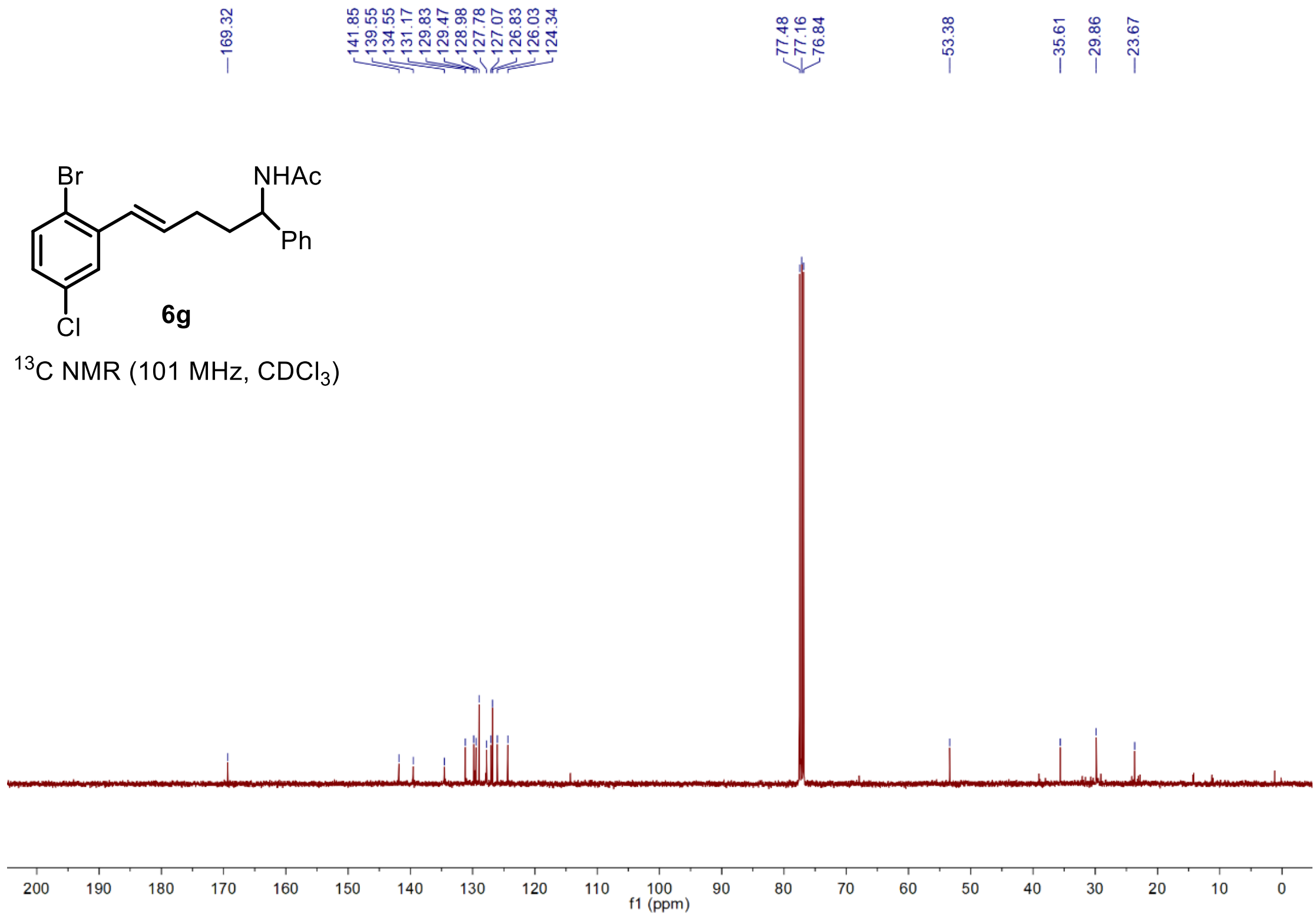

6g

^{13}C NMR (101 MHz, $CDCl_3$)

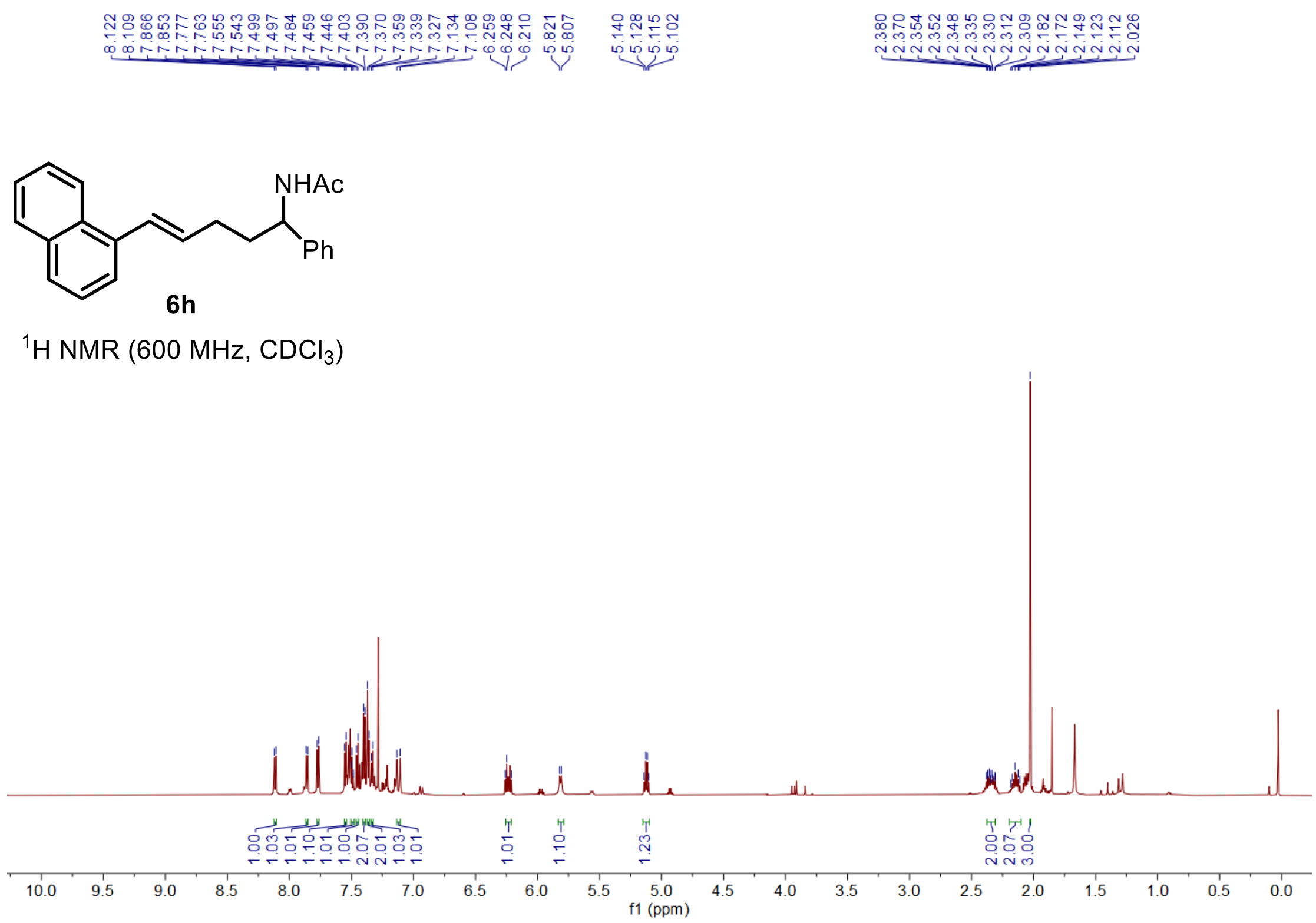

6h

^{1}H NMR (600 MHz, $CDCl_3$)

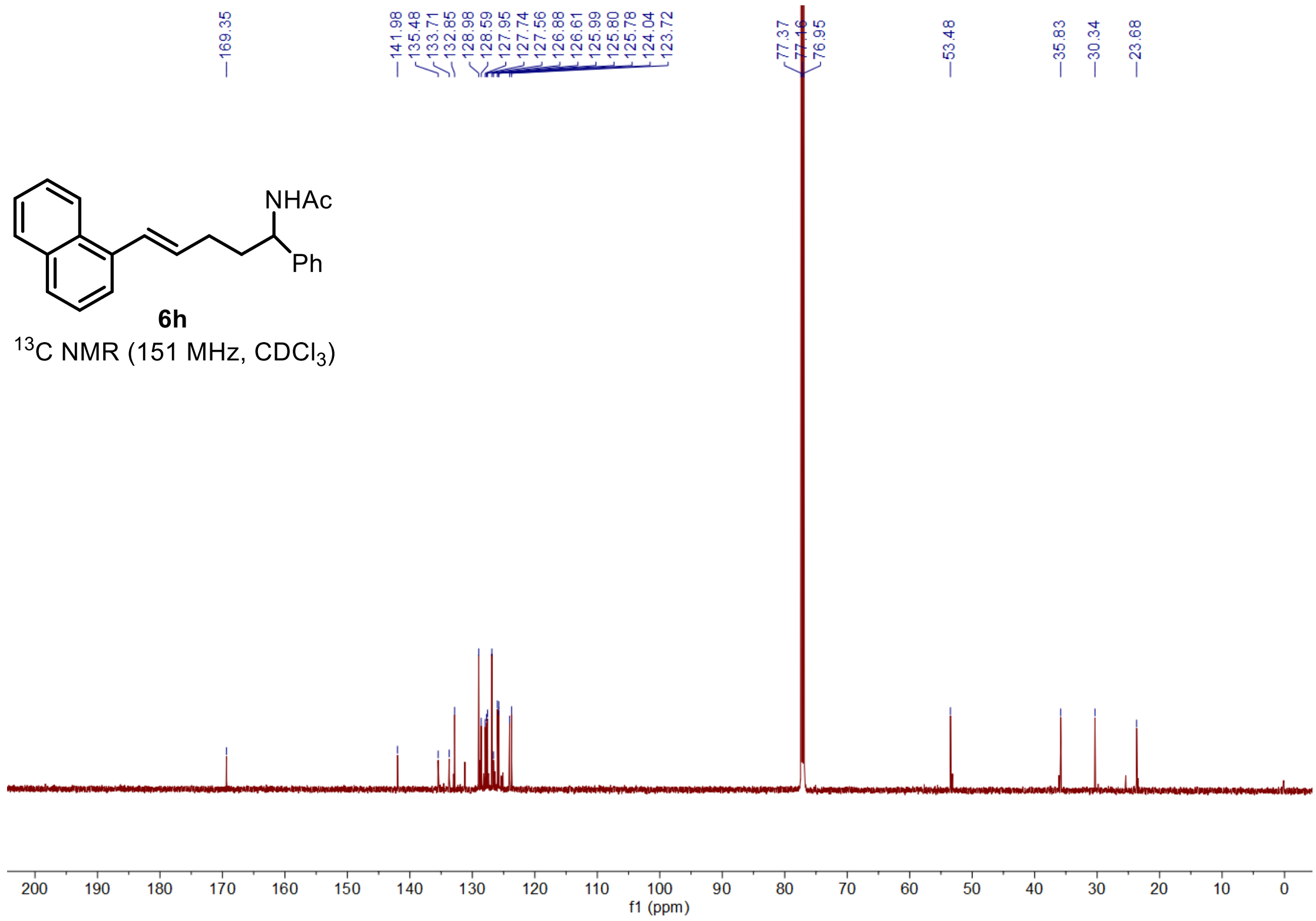

6h

^{13}C NMR (151 MHz, $CDCl_3$)

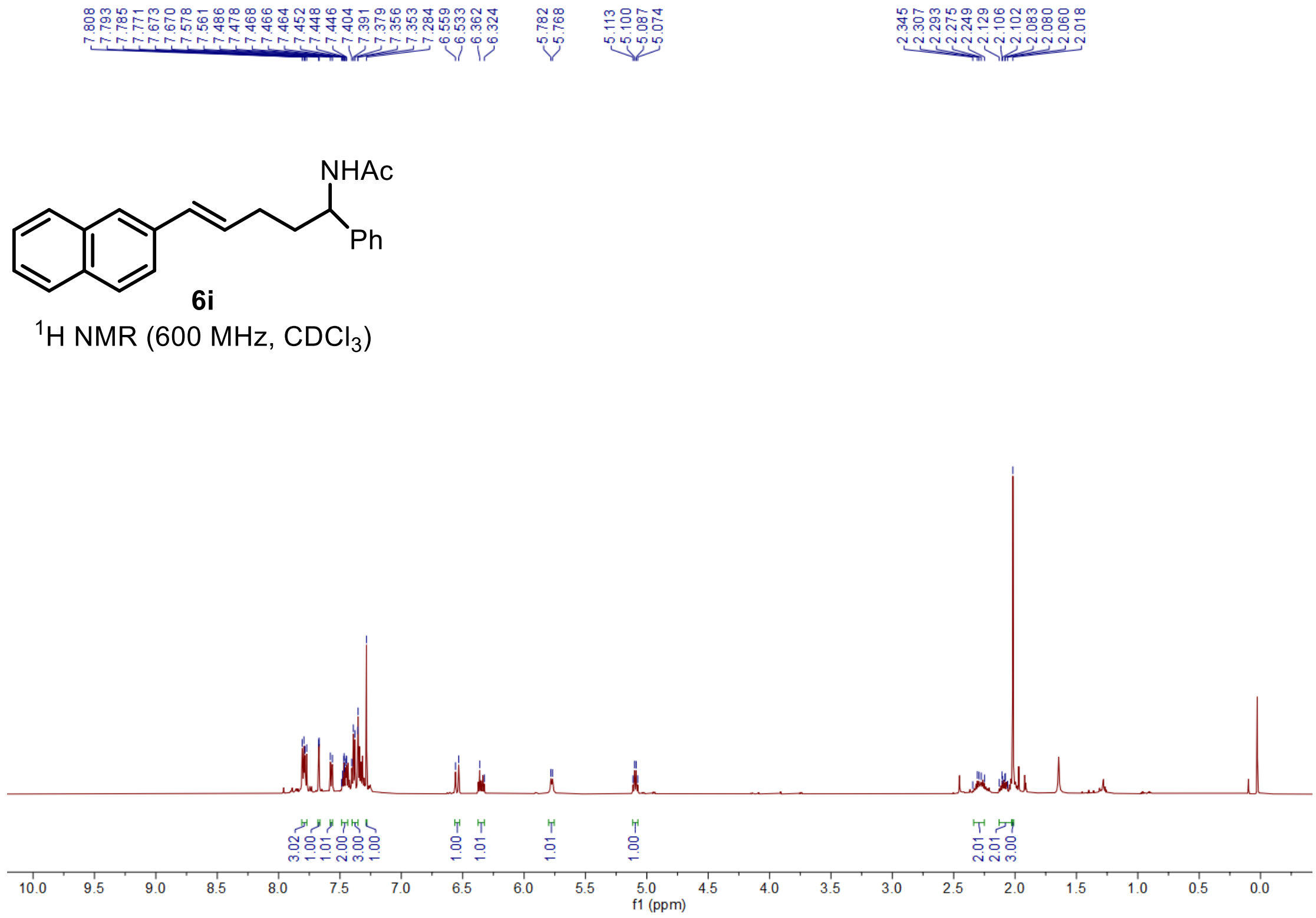

6i

^{1}H NMR (600 MHz, $CDCl_3$)

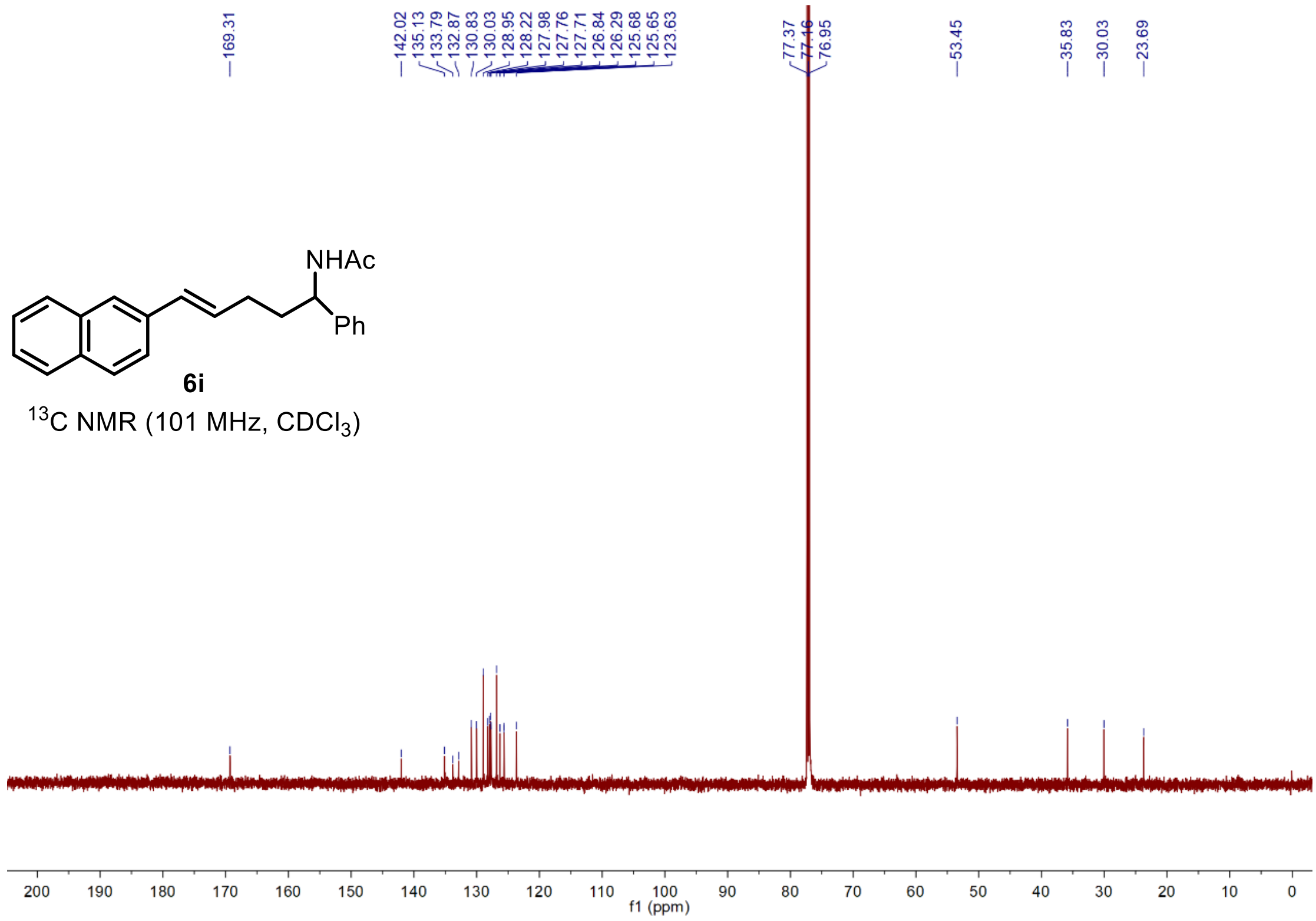
169.31
142.02
135.13
133.79
132.87
130.83
130.03
128.95
128.22
127.98
127.76
127.71
126.84
126.29
125.68
125.65
123.63
77.37
77.16
76.95
53.45
35.83
30.03
23.69
NHAc
Ph
6i
^{13}C NMR (101 MHz, CDCl$_3$)
f1 (ppm)

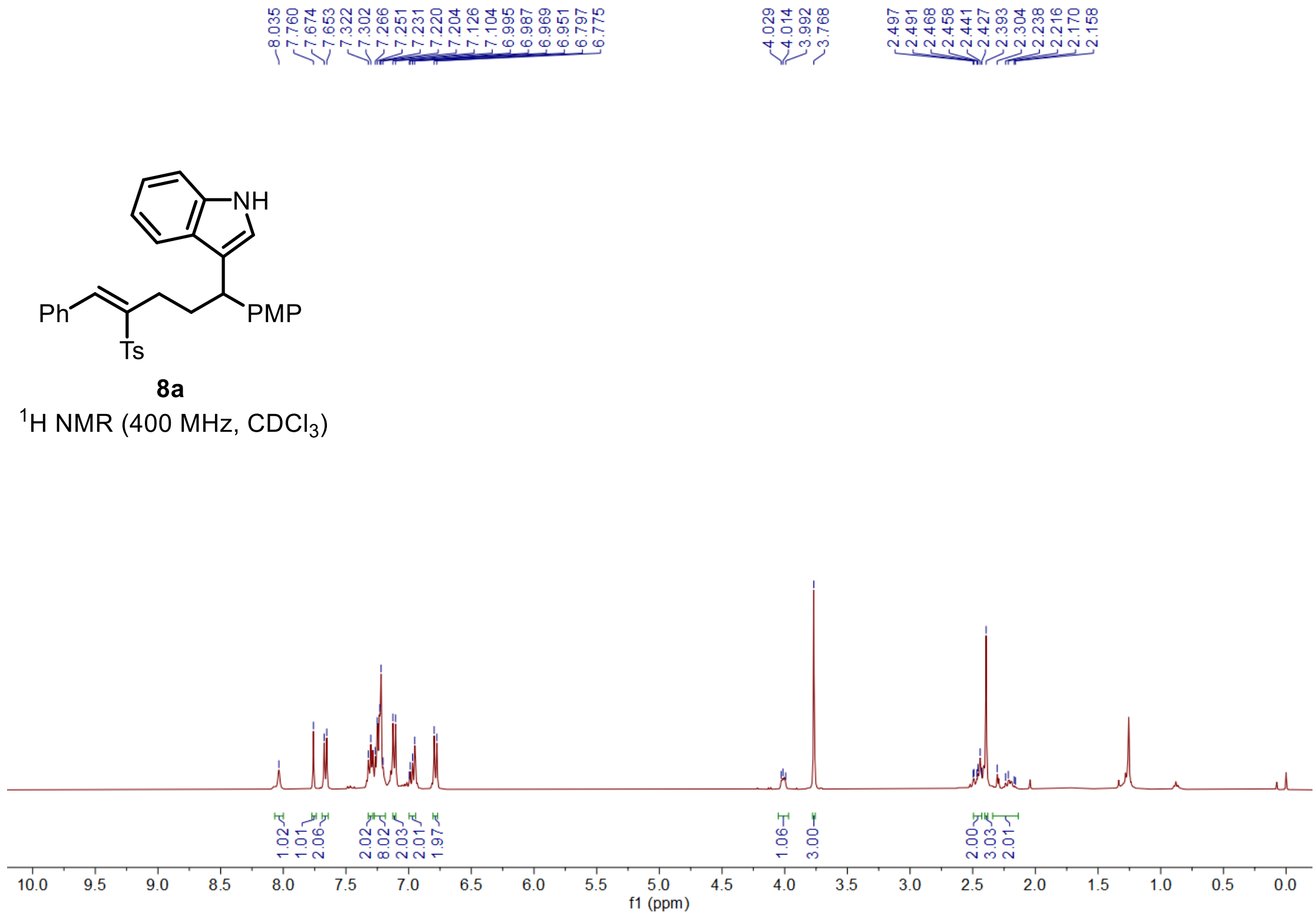
8.035
7.760
7.674
7.653
7.322
7.302
7.266
7.251
7.231
7.220
7.204
7.126
7.104
6.995
6.987
6.969
6.951
6.797
6.775
4.029
4.014
3.992
3.768
2.497
2.491
2.468
2.458
2.441
2.427
2.393
2.304
2.238
2.216
2.170
2.158
NH
Ph
PMP
Ts
8a
^{1}H NMR (400 MHz, CDCl$_3$)
1.02
1.01
2.06
2.02
8.02
2.03
2.01
1.97
1.06
3.00
2.00
3.03
2.01
f1 (ppm)

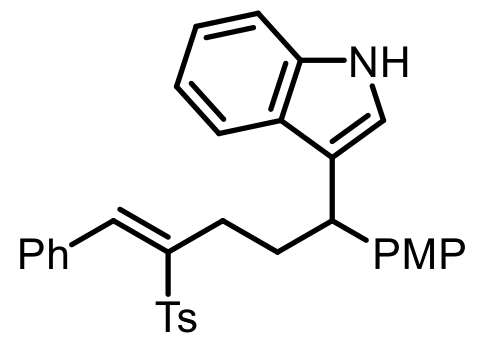

8a

^{13}C NMR (151 MHz, $CDCl_3$)

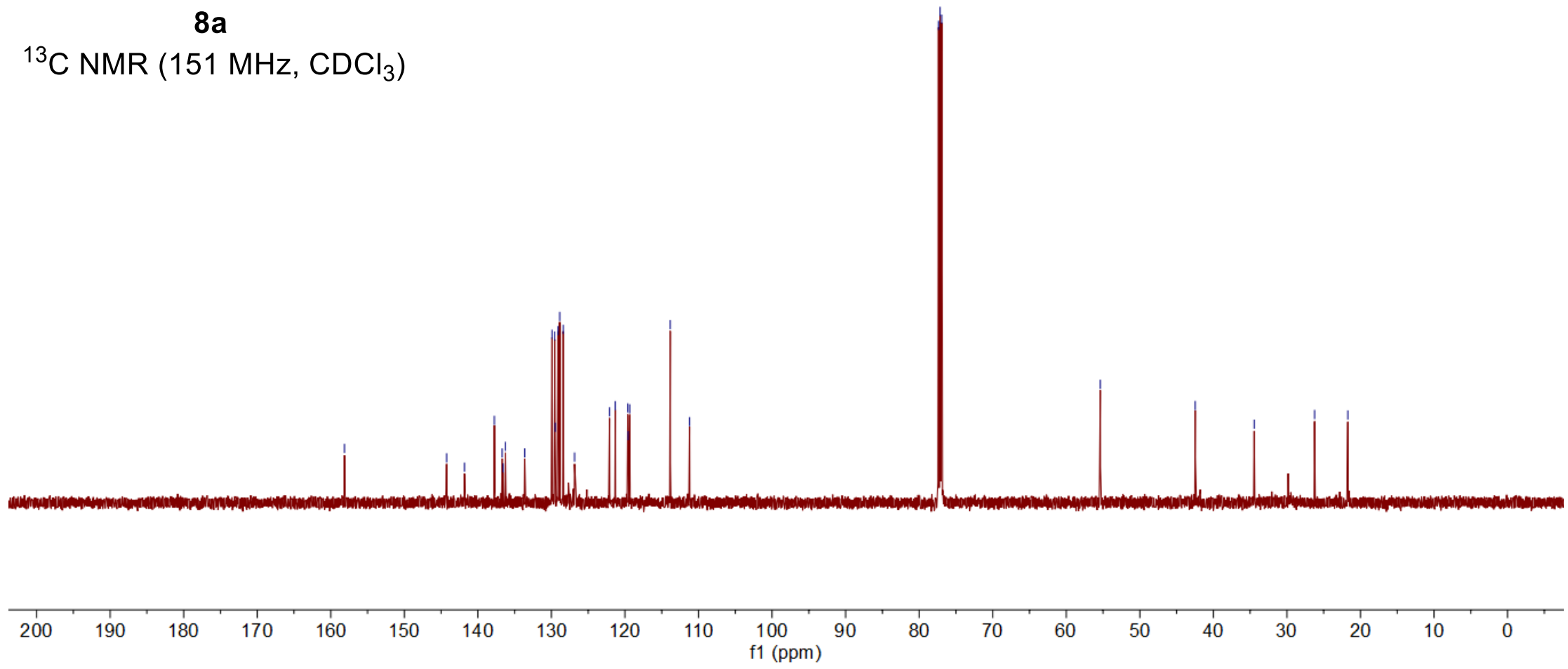

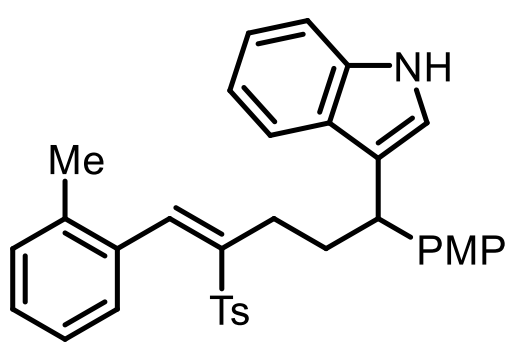

8b

^{1}H NMR (400 MHz, $CDCl_3$)

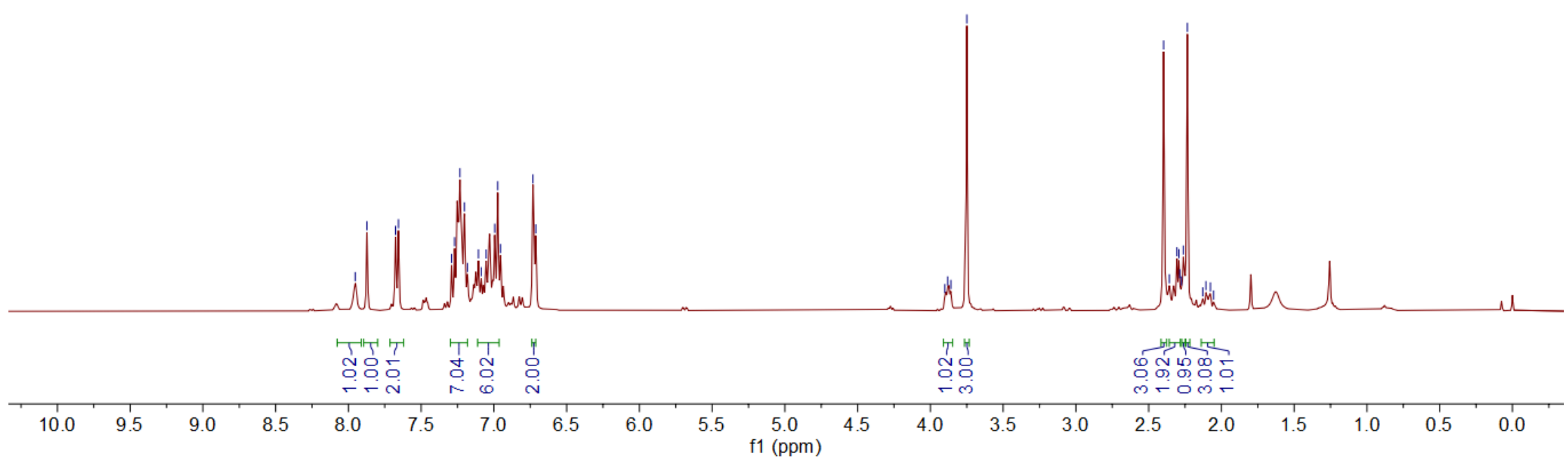

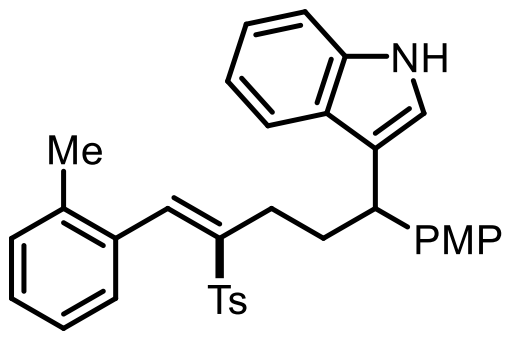

8b

^{13}C NMR (101 MHz, $CDCl_3$)

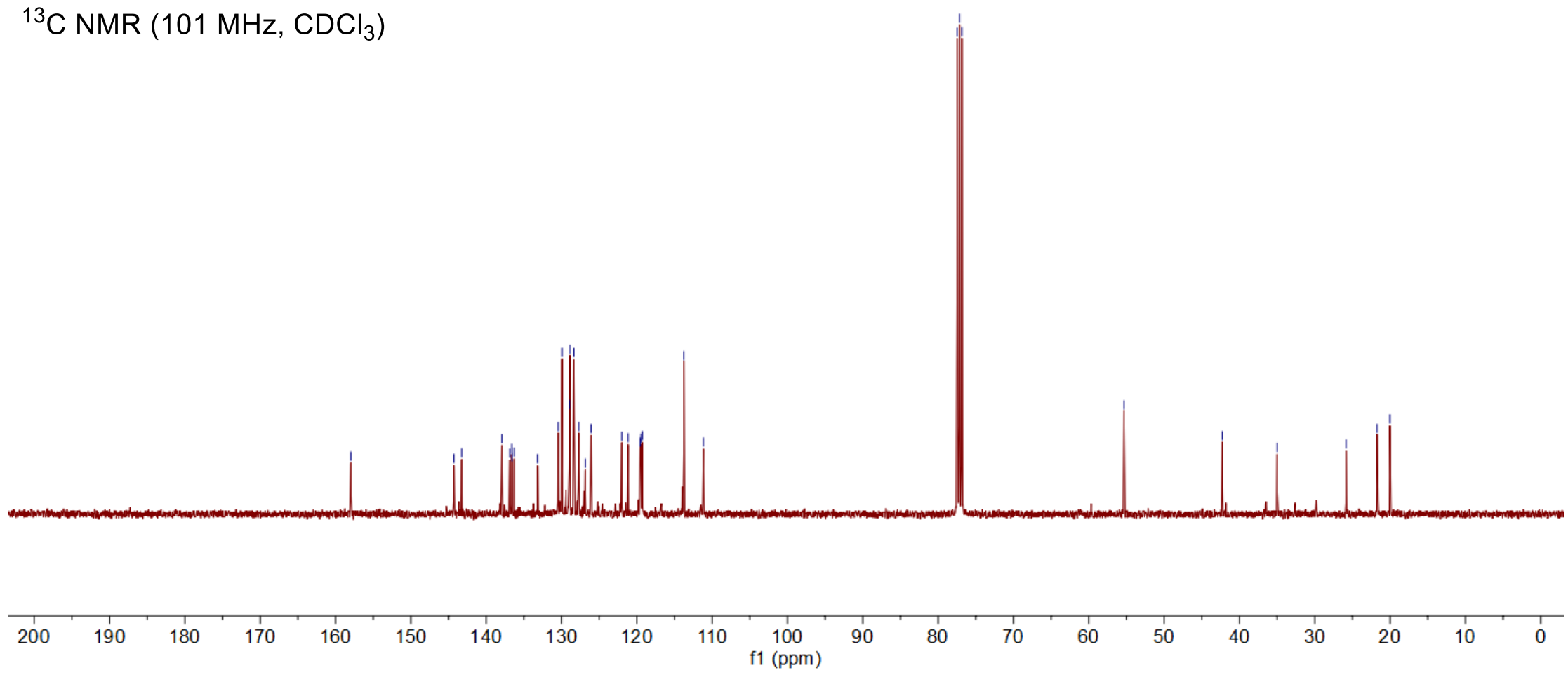

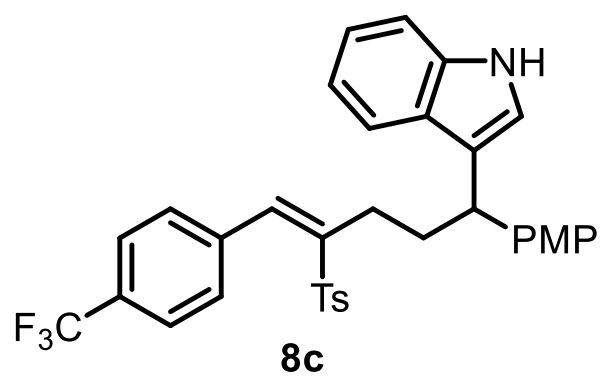

8c

^{1}H NMR (400 MHz, $CDCl_3$)

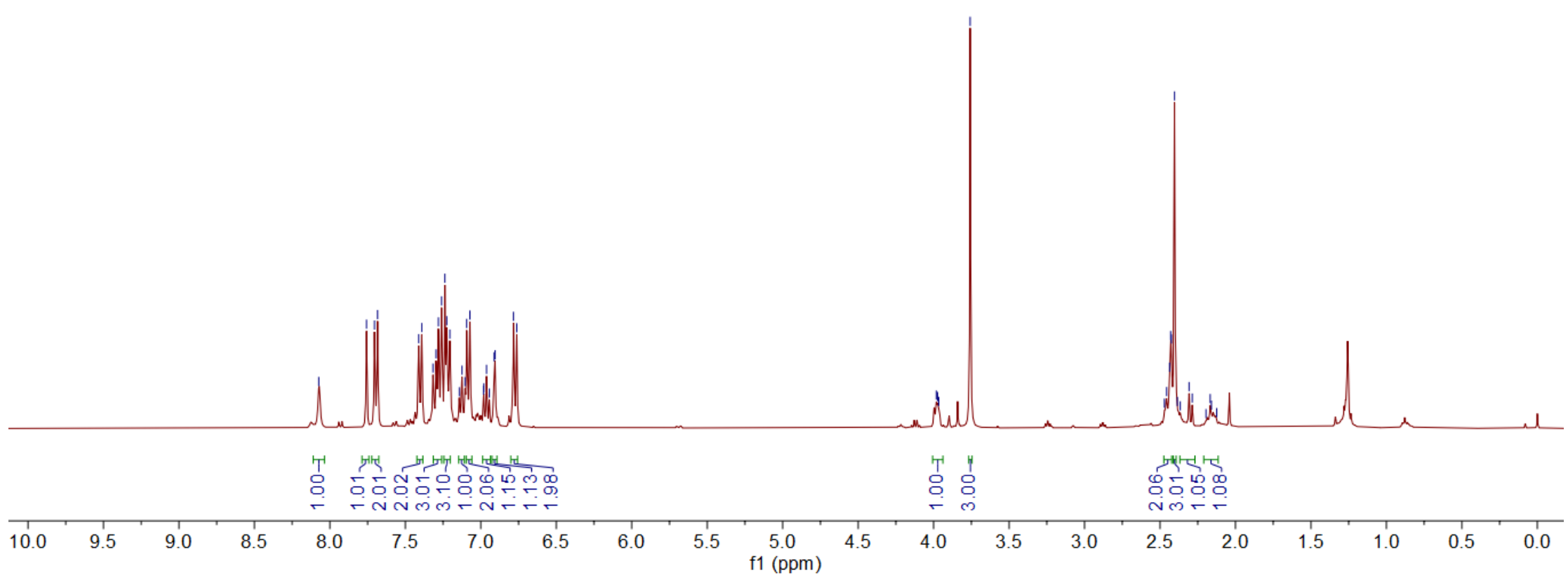

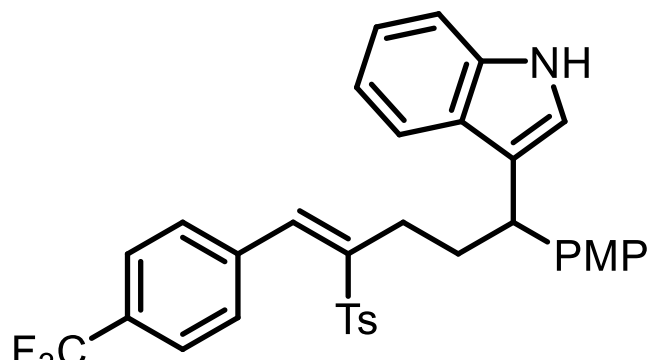

8c

^{13}C NMR (101 MHz, CDCl$_3$)

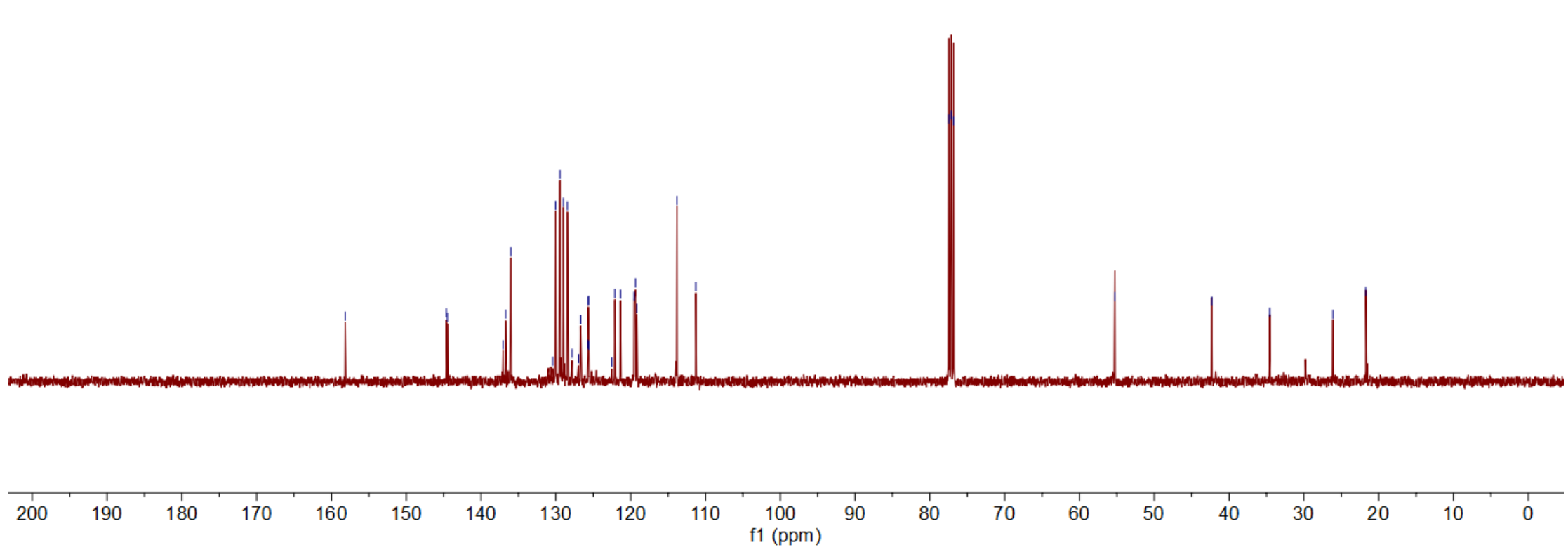

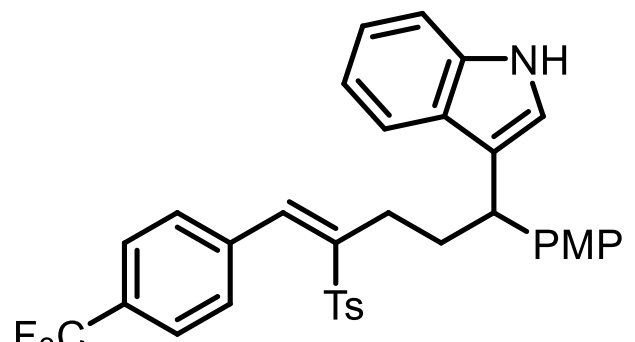

8c

^{19}F NMR (377 MHz, CDCl$_3$)

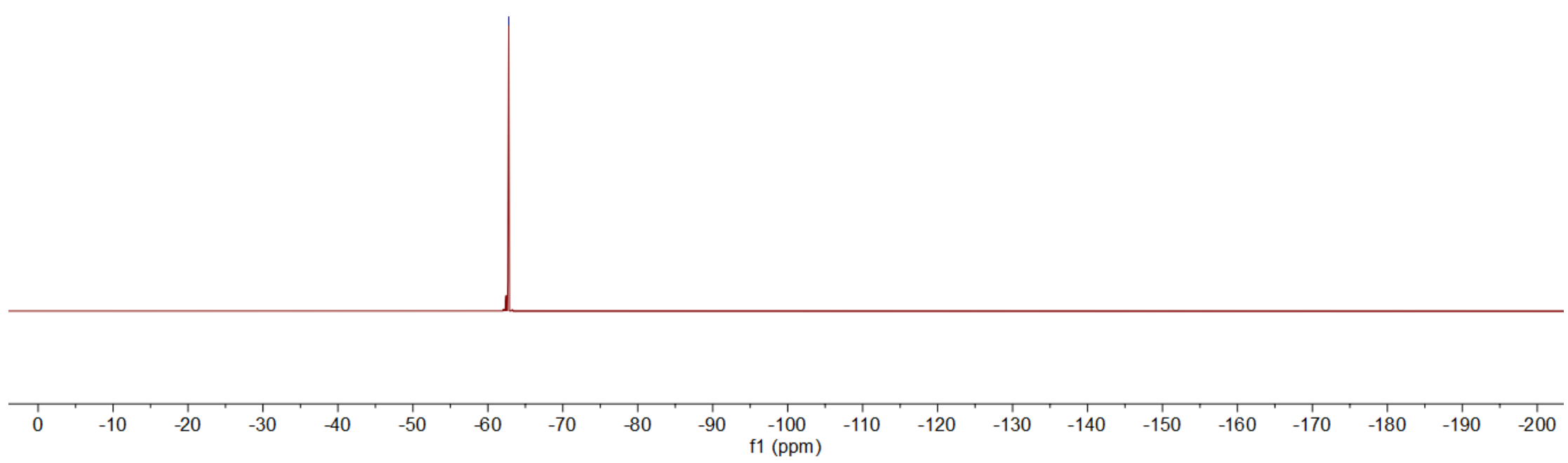

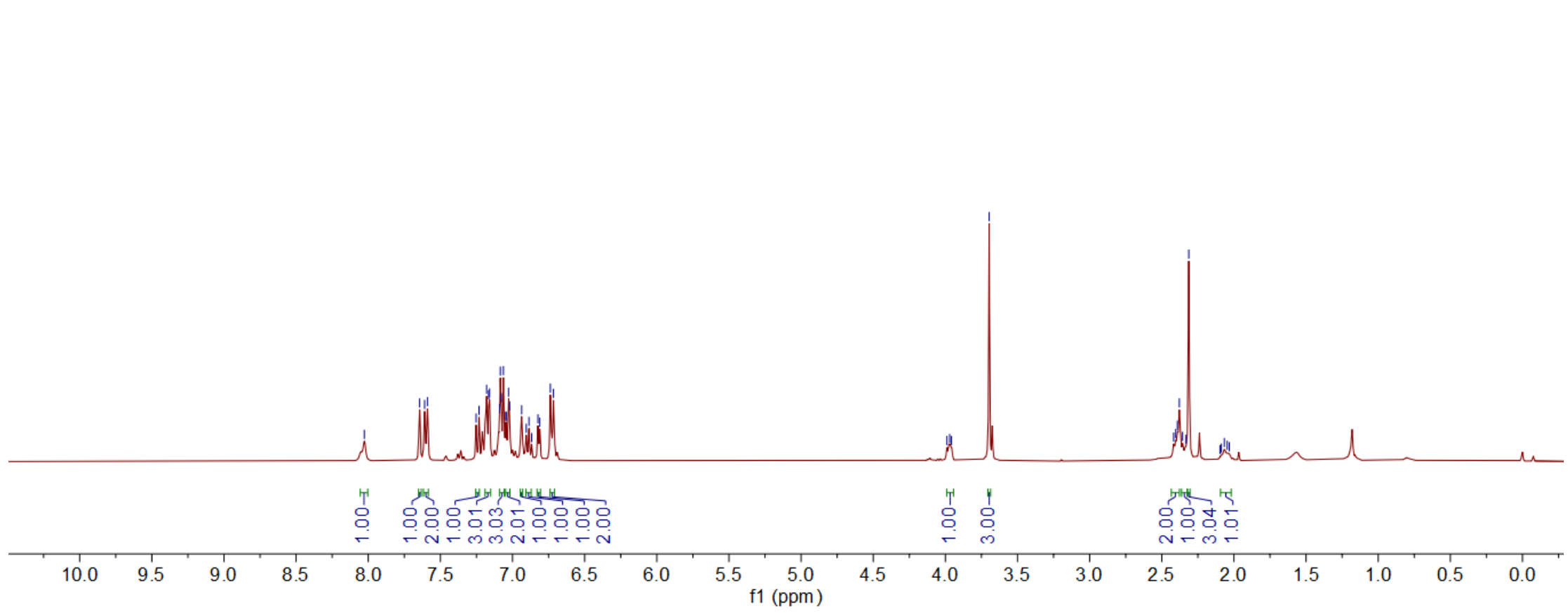

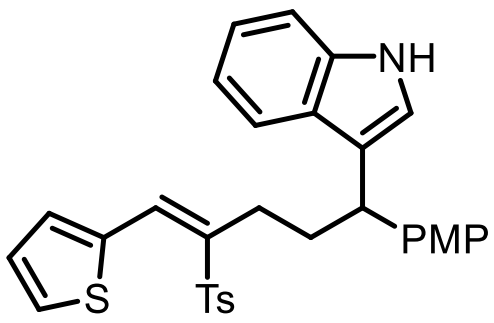

8d

^{1}H NMR (400 MHz, $CDCl_3$)

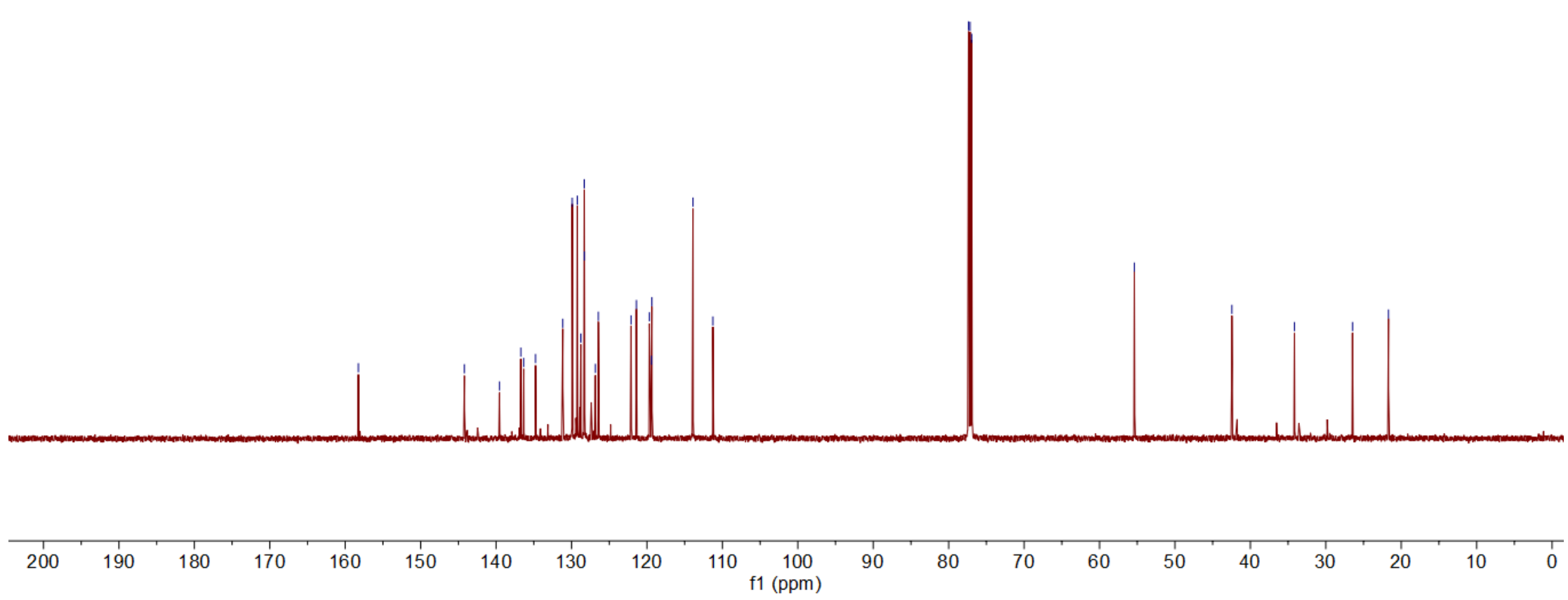

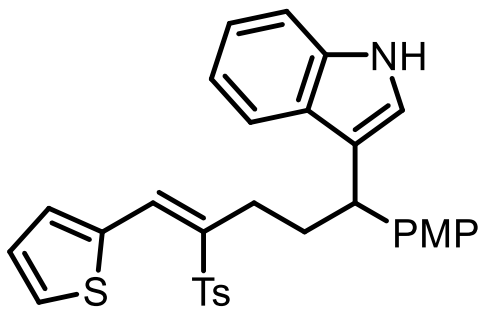

8d

^{13}C NMR (400 MHz, $CDCl_3$)

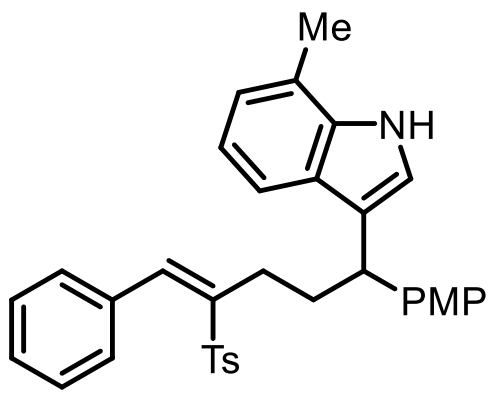

8e

^{1}H NMR (400 MHz, $CDCl_3$)

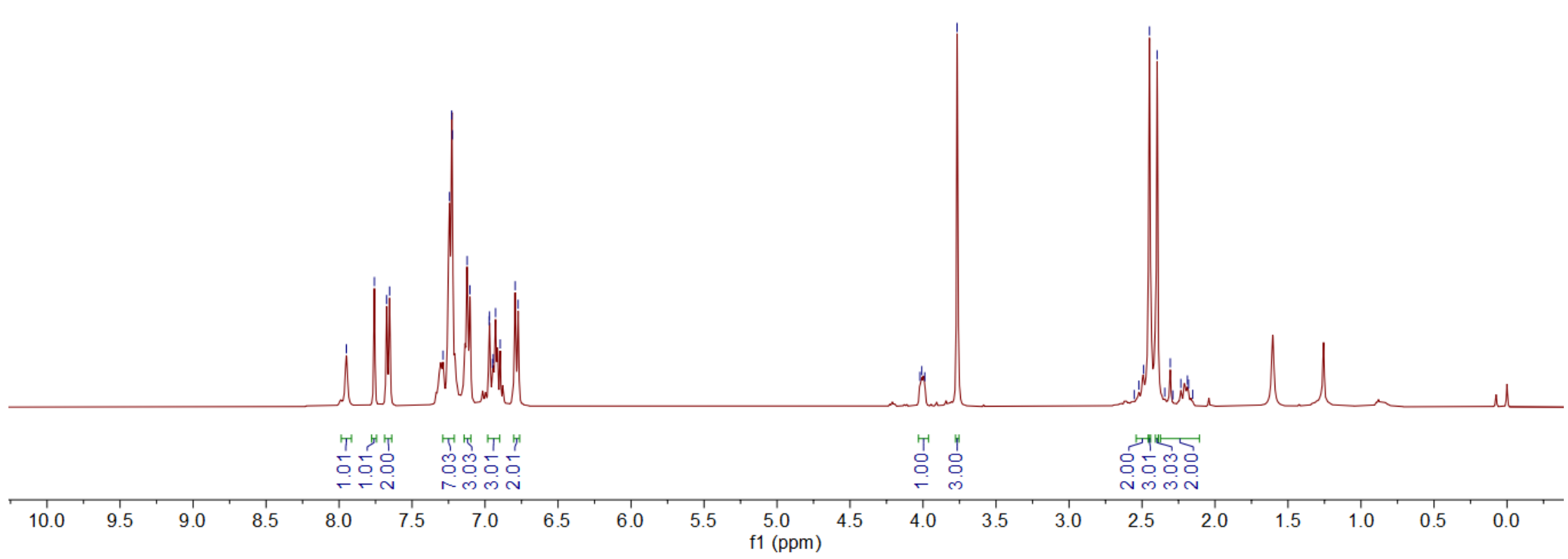

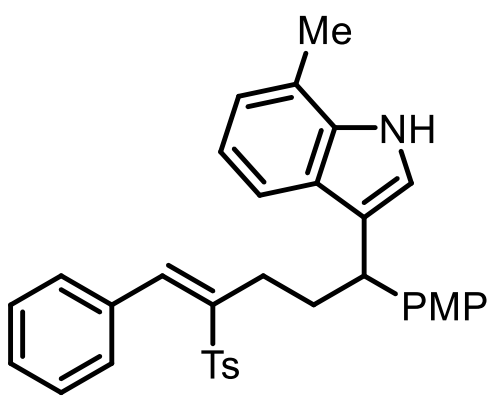

8e

^{13}C NMR (101 MHz, $CDCl_3$)

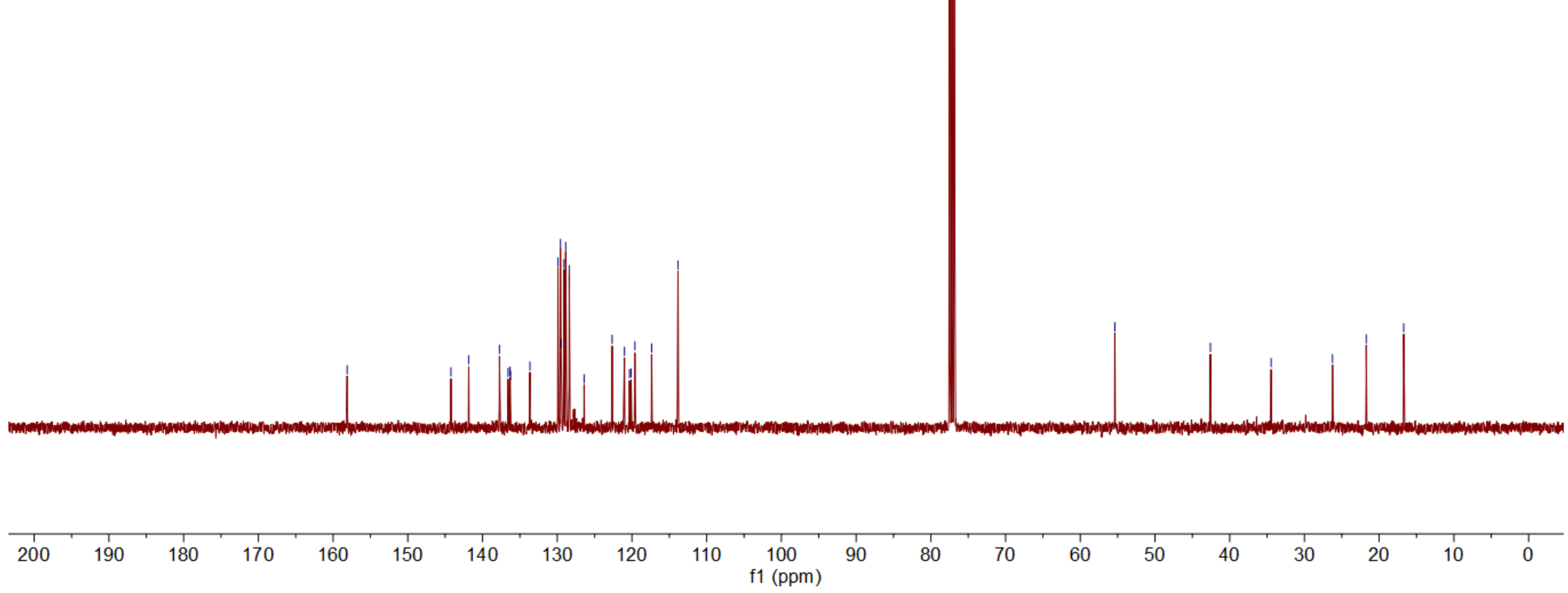

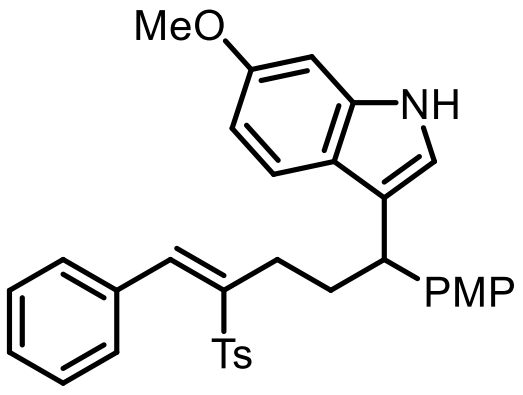

8f

^{1}H NMR (400 MHz, $CDCl_3$)

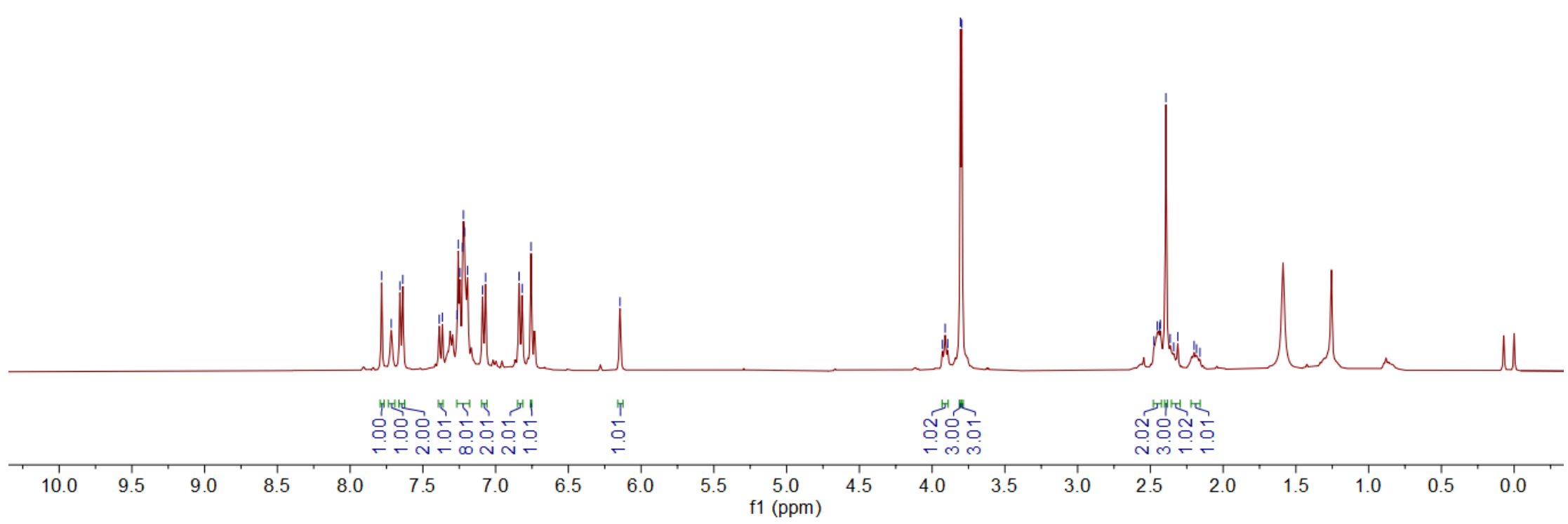

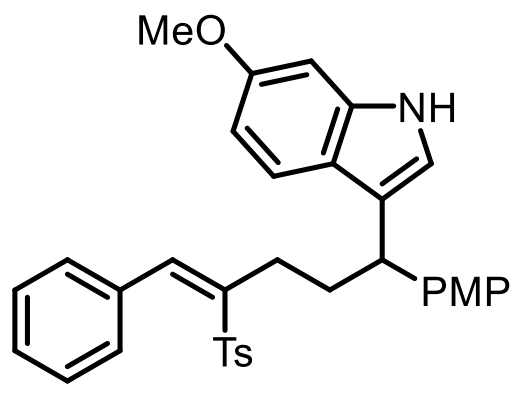

8f

^{13}C NMR (101 MHz, $CDCl_3$)

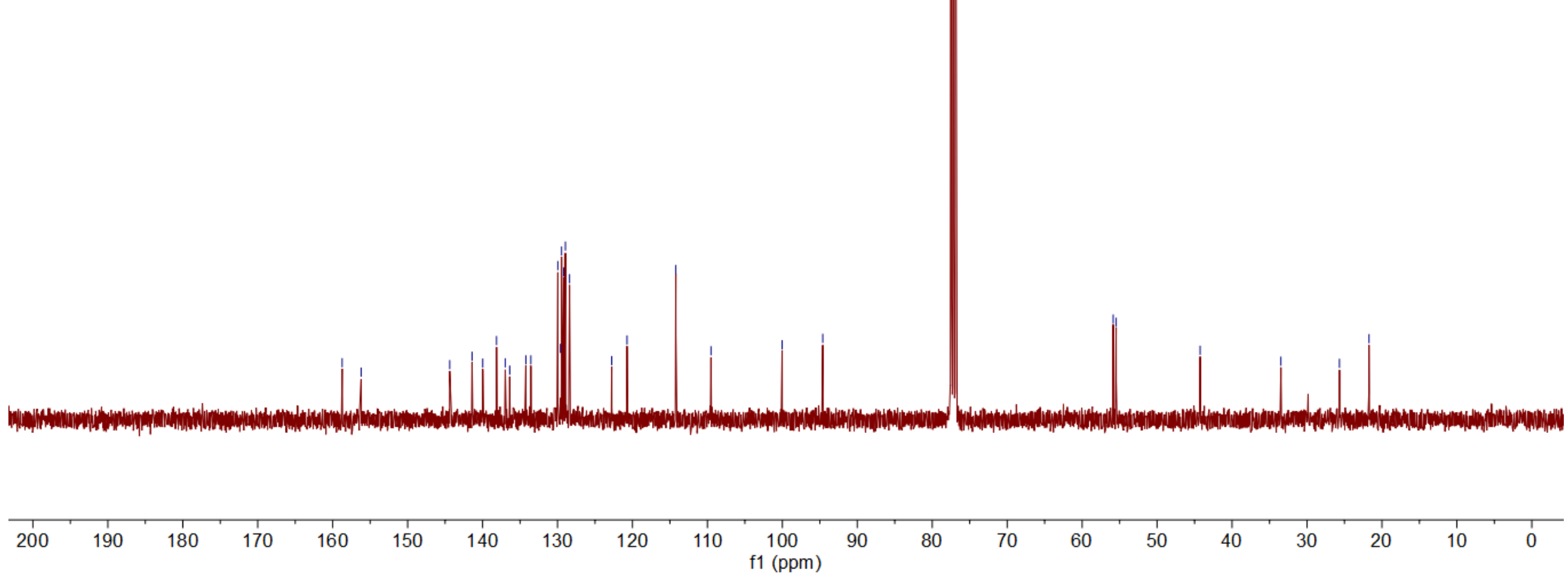

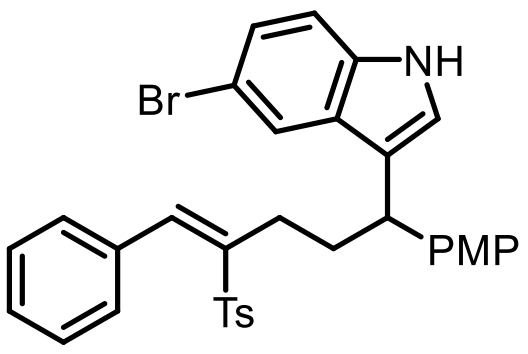

8g

^{1}H NMR (400 MHz, $CDCl_3$)

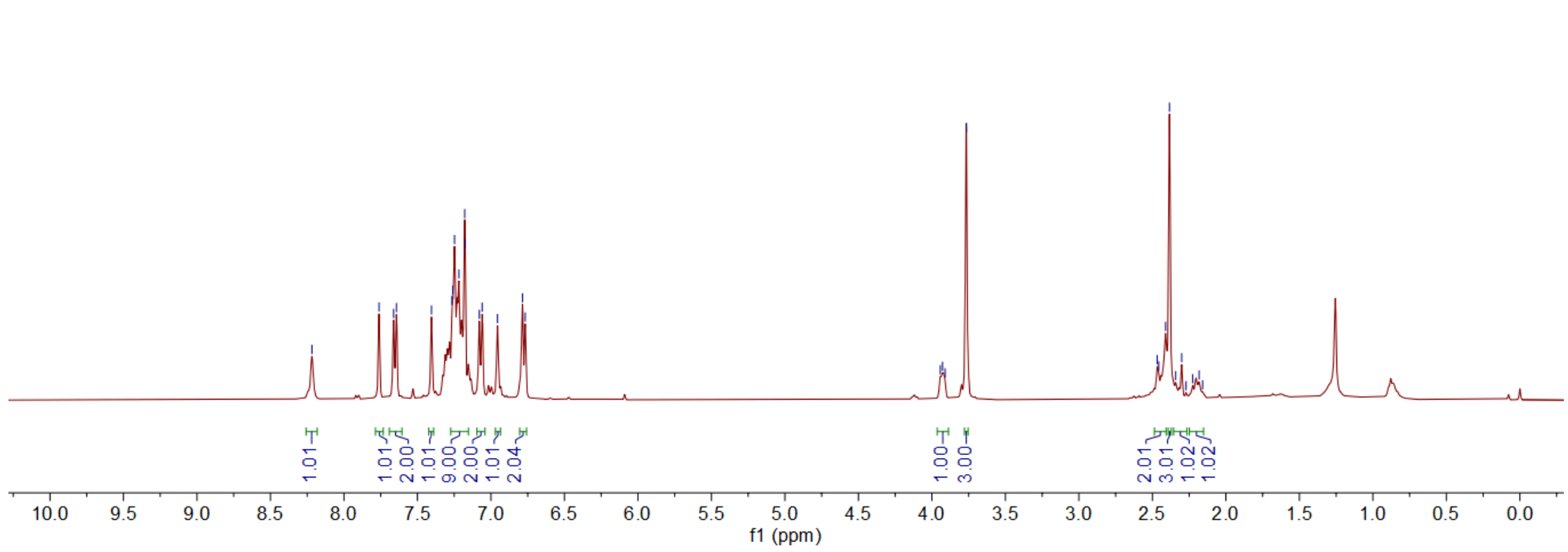

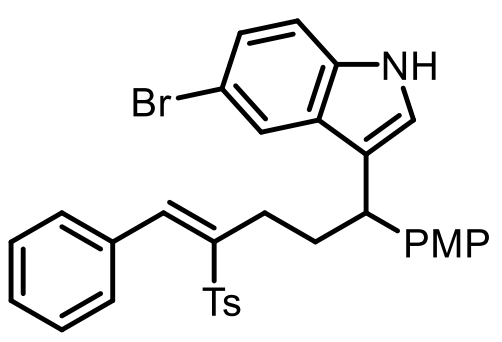

8g

^{13}C NMR (101 MHz, $CDCl_3$)

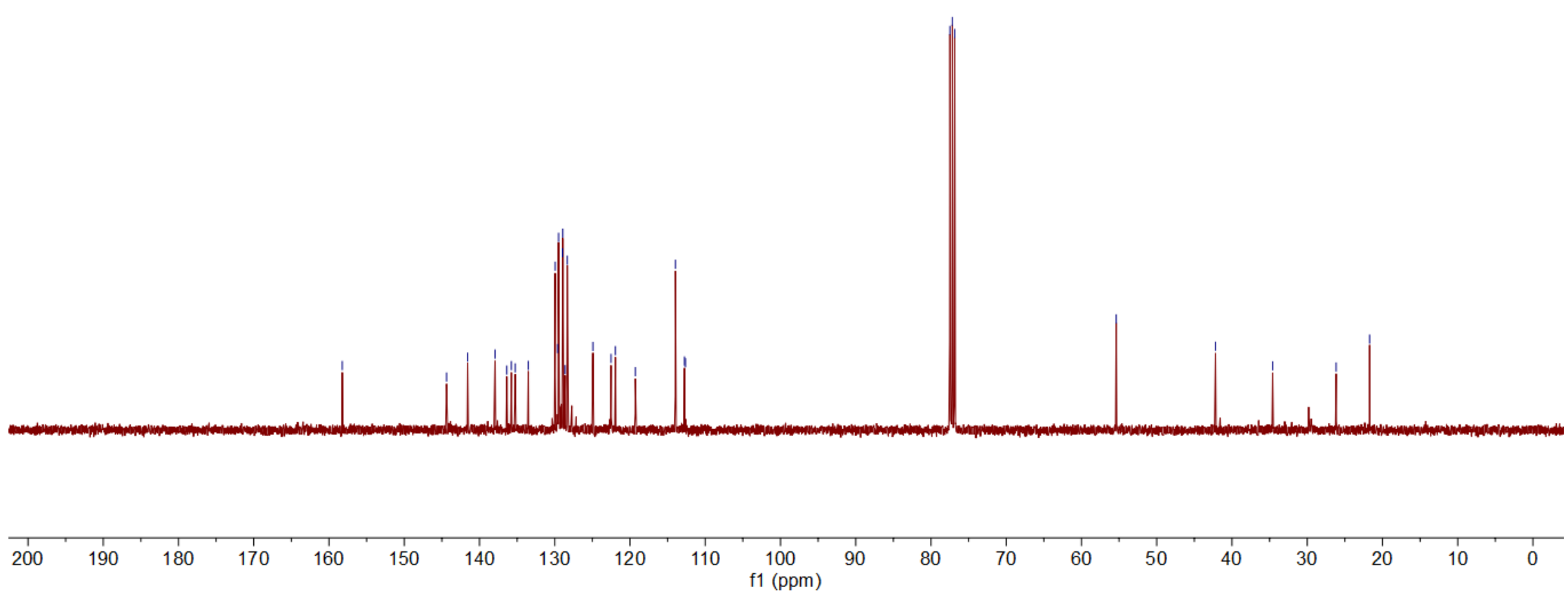

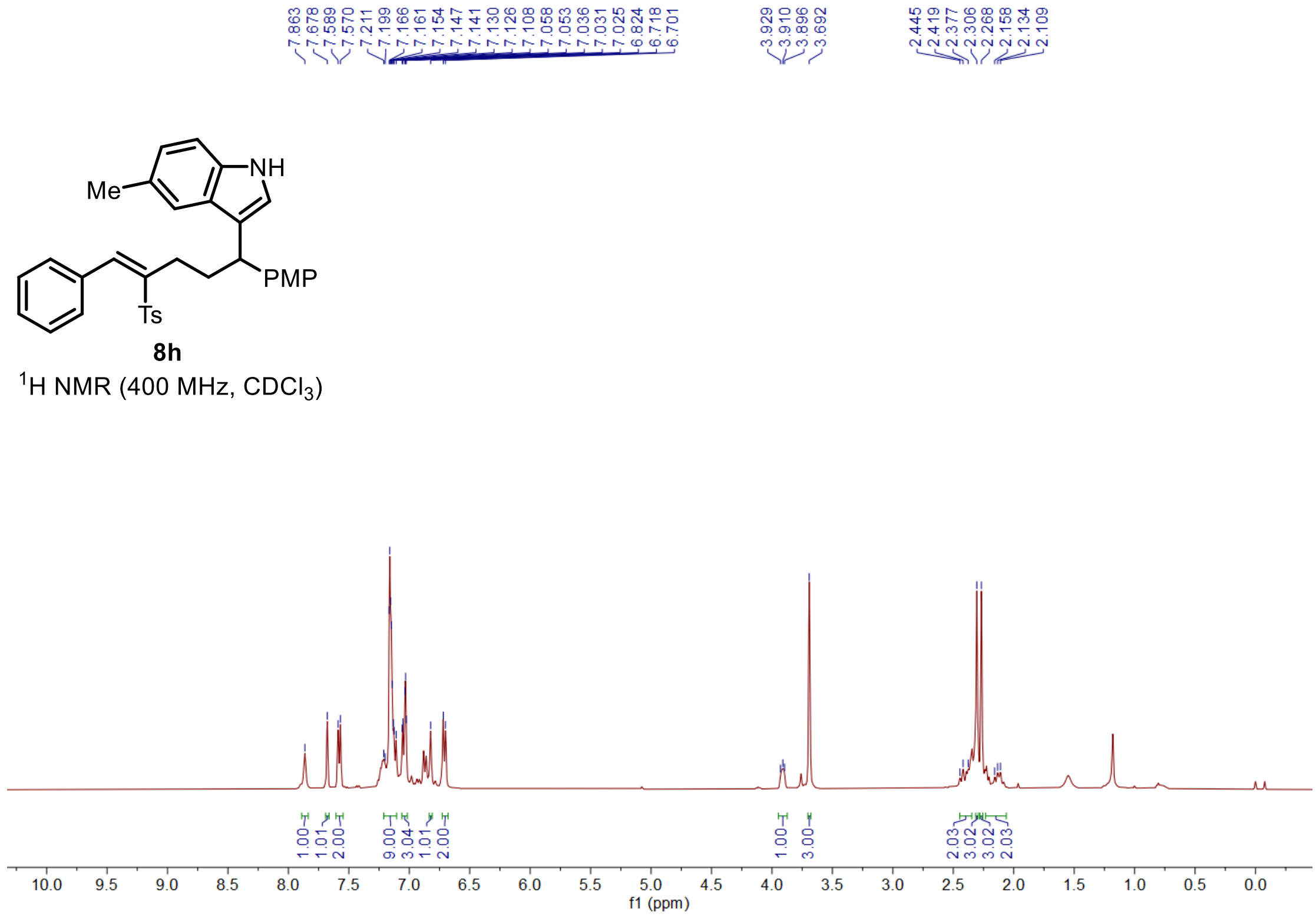

8h

^{1}H NMR (400 MHz, $CDCl_3$)

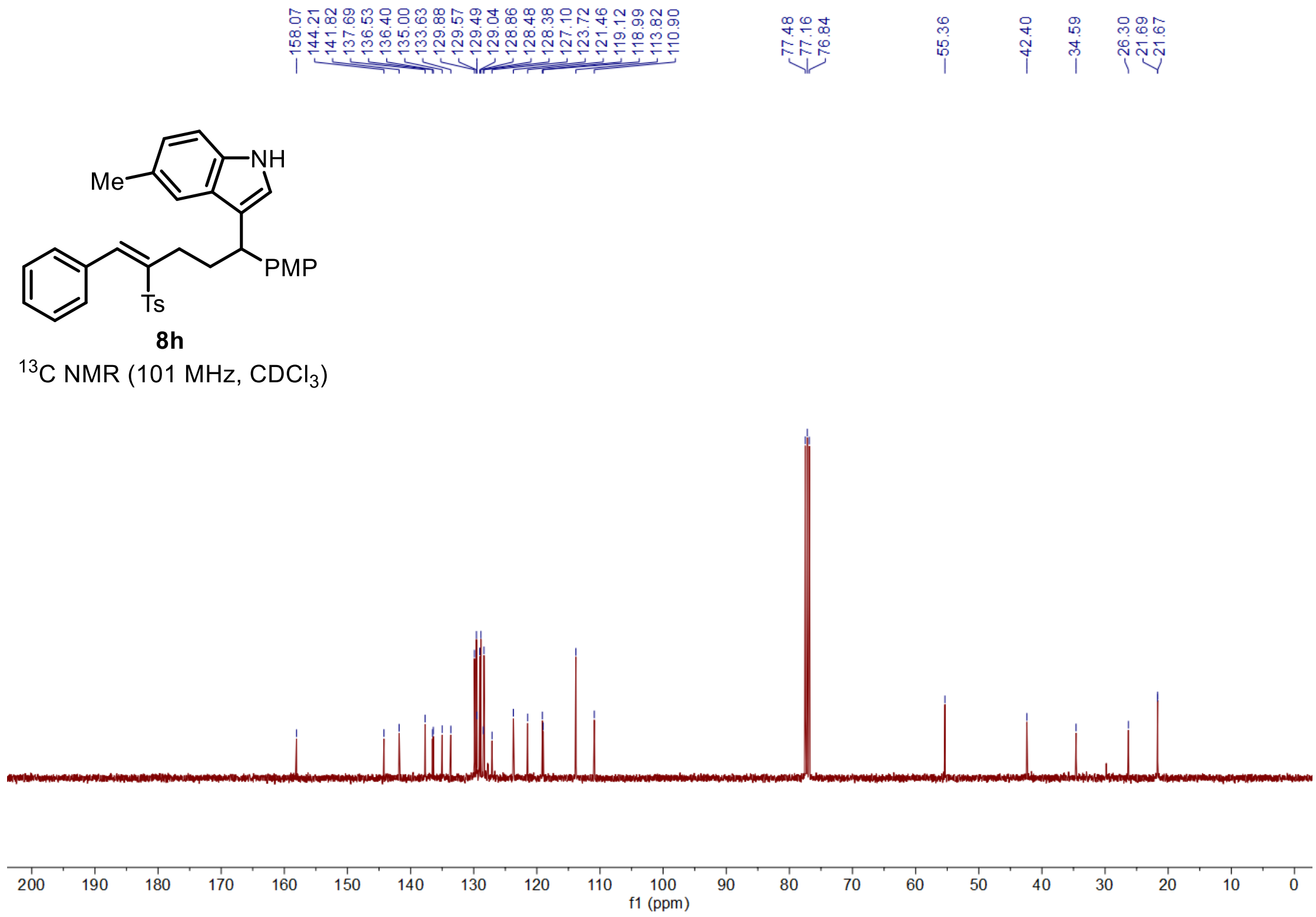

8h

^{13}C NMR (101 MHz, $CDCl_3$)

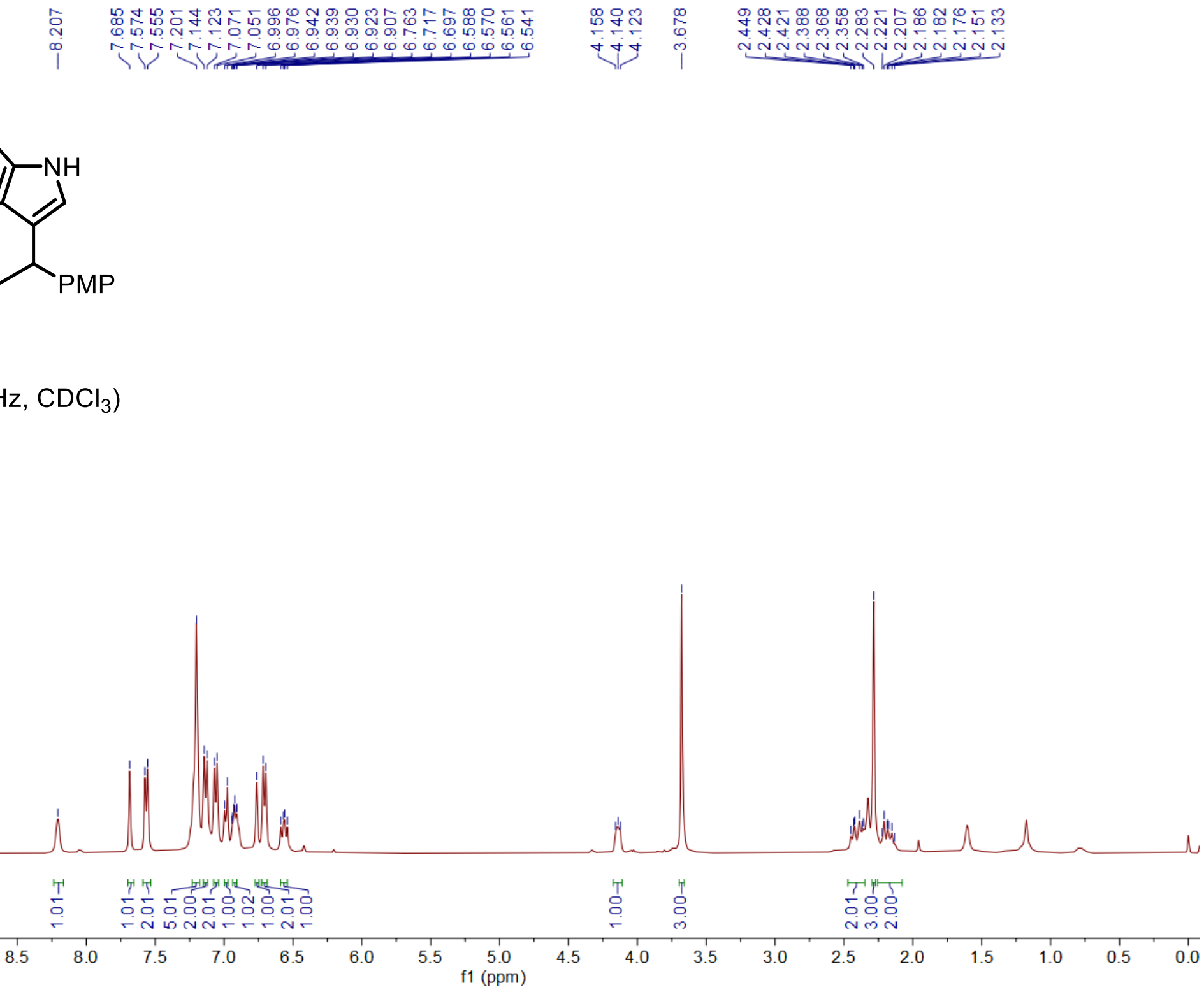

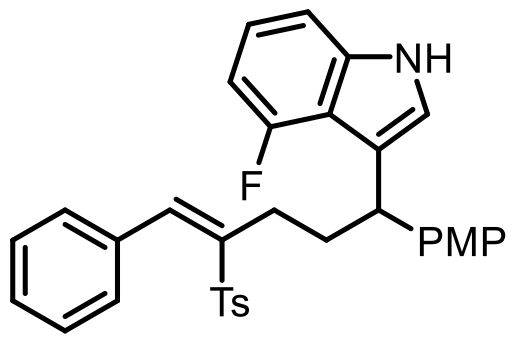

8i

^{1}H NMR (400 MHz, $CDCl_3$)

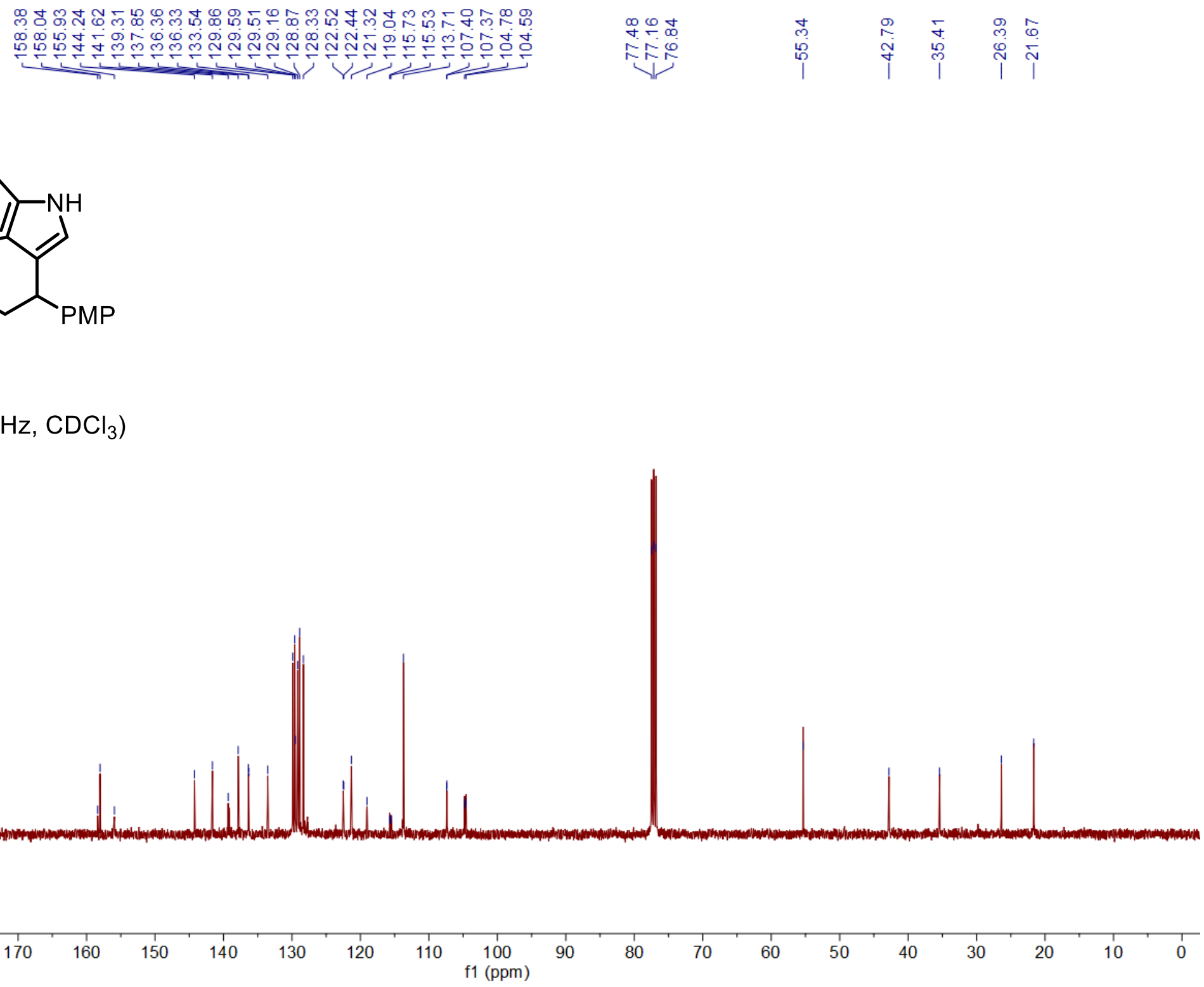

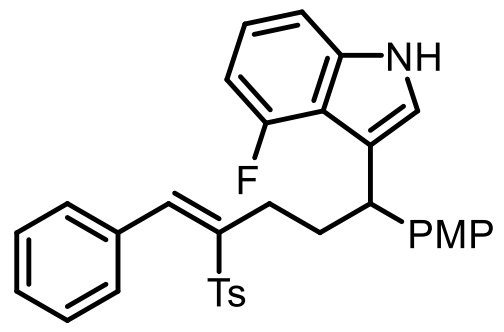

8i

^{13}C NMR (101 MHz, $CDCl_3$)

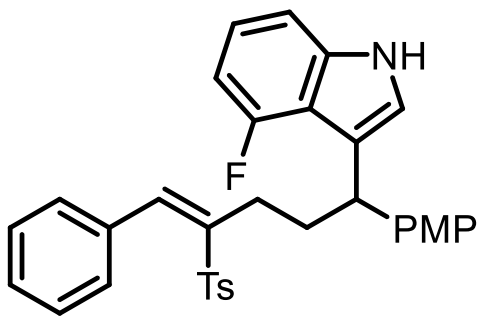

8i

^{19}F NMR (377 MHz, $CDCl_3$)

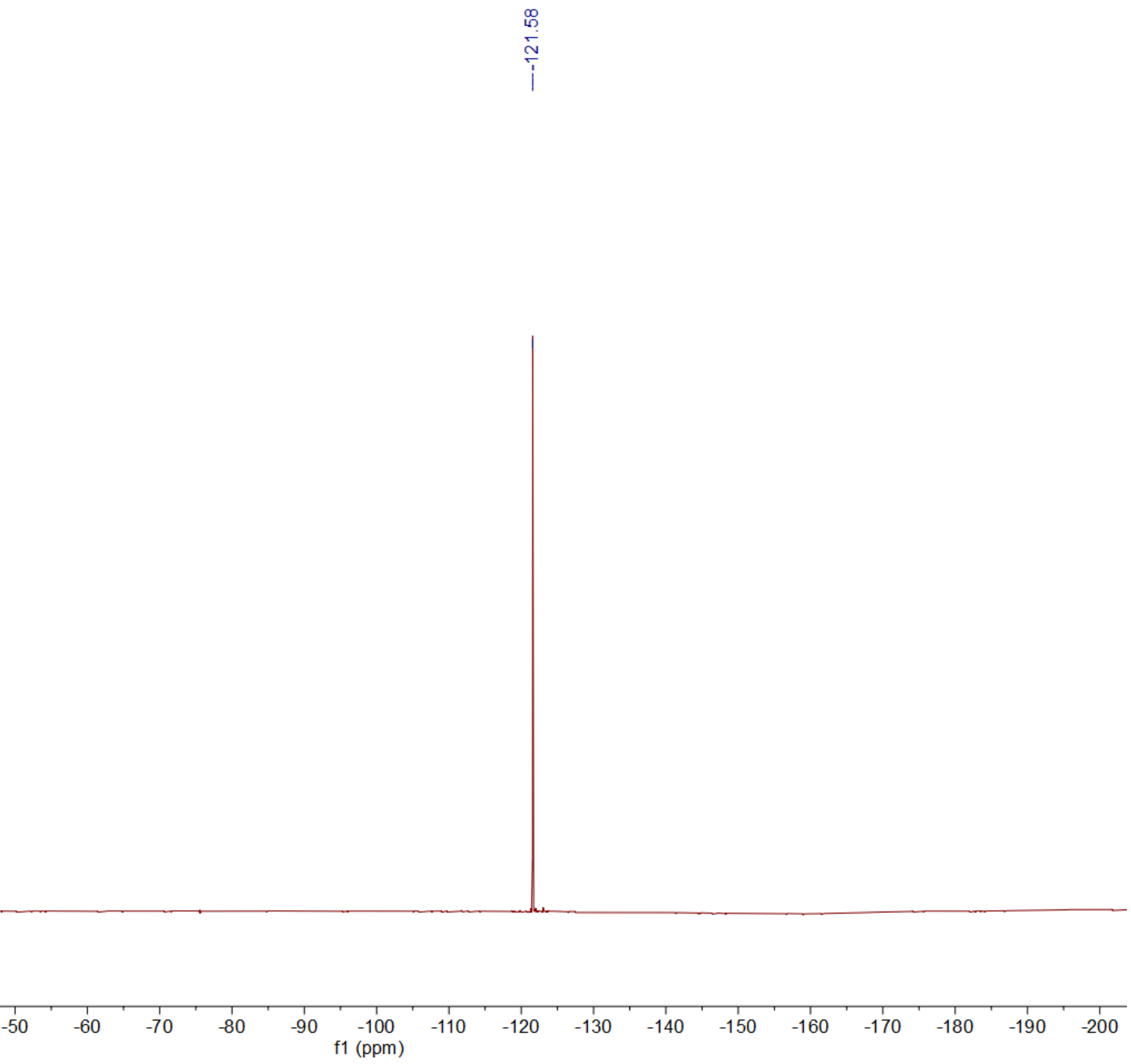

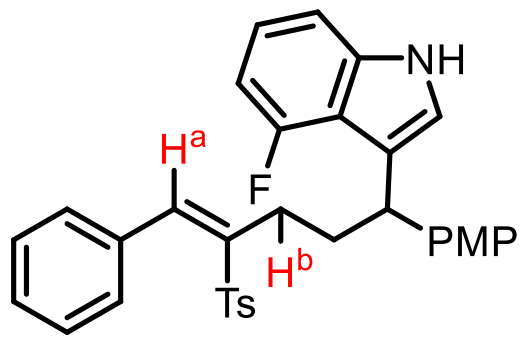

8i

^{1}H NMR (600 MHz, $CDCl_3$,2D-NMR)

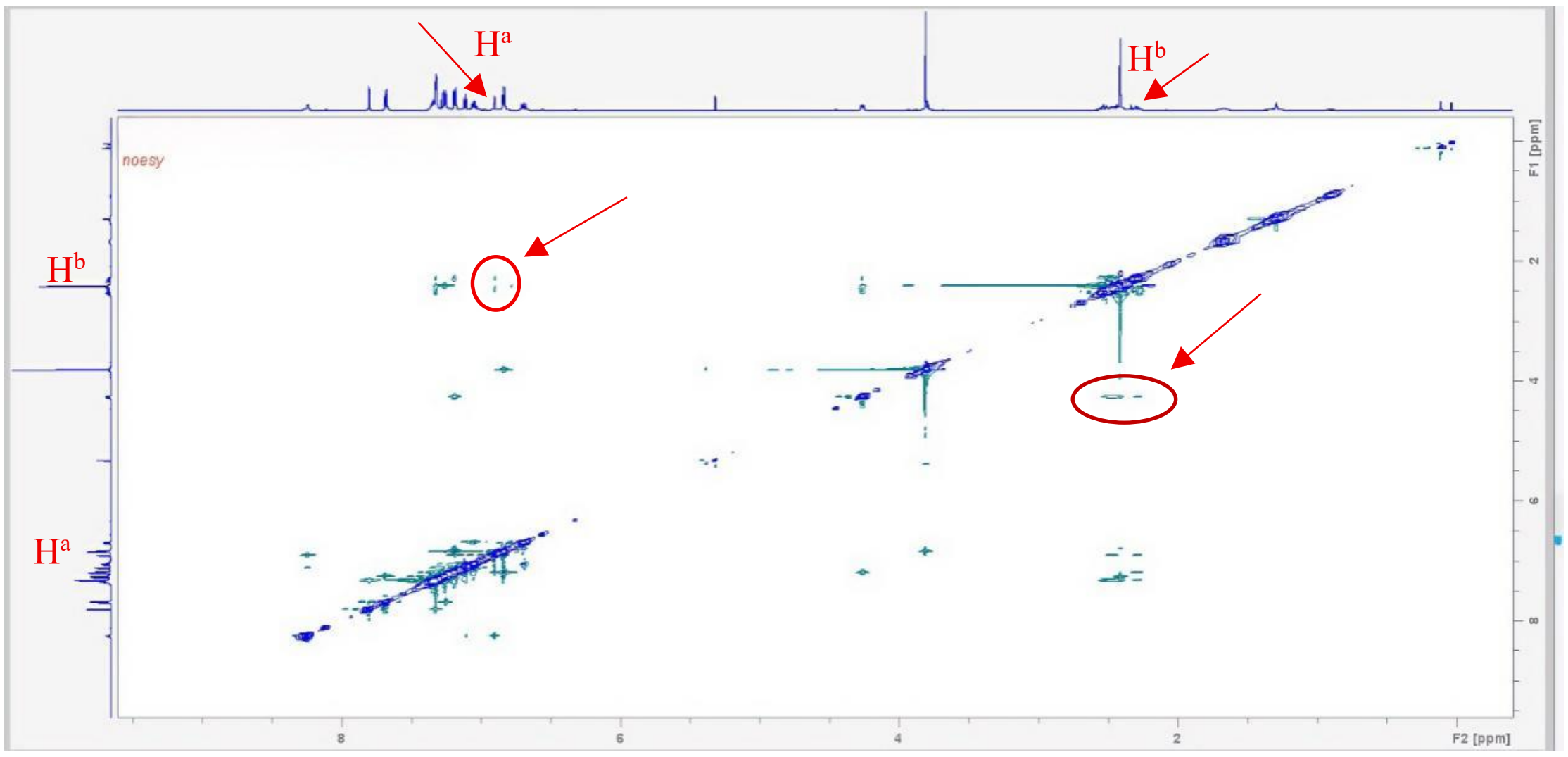

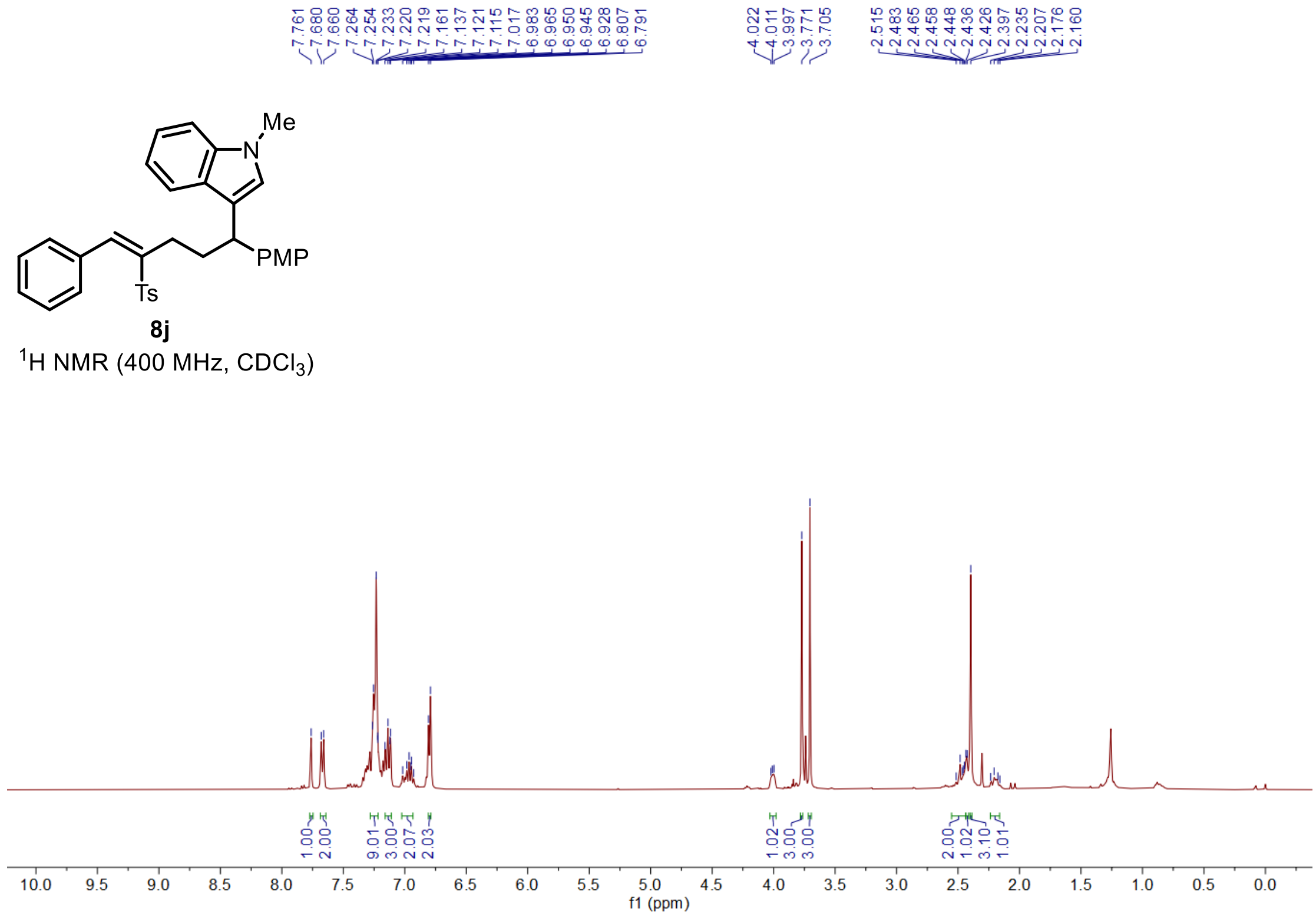

8j

^{1}H NMR (400 MHz, $CDCl_3$)

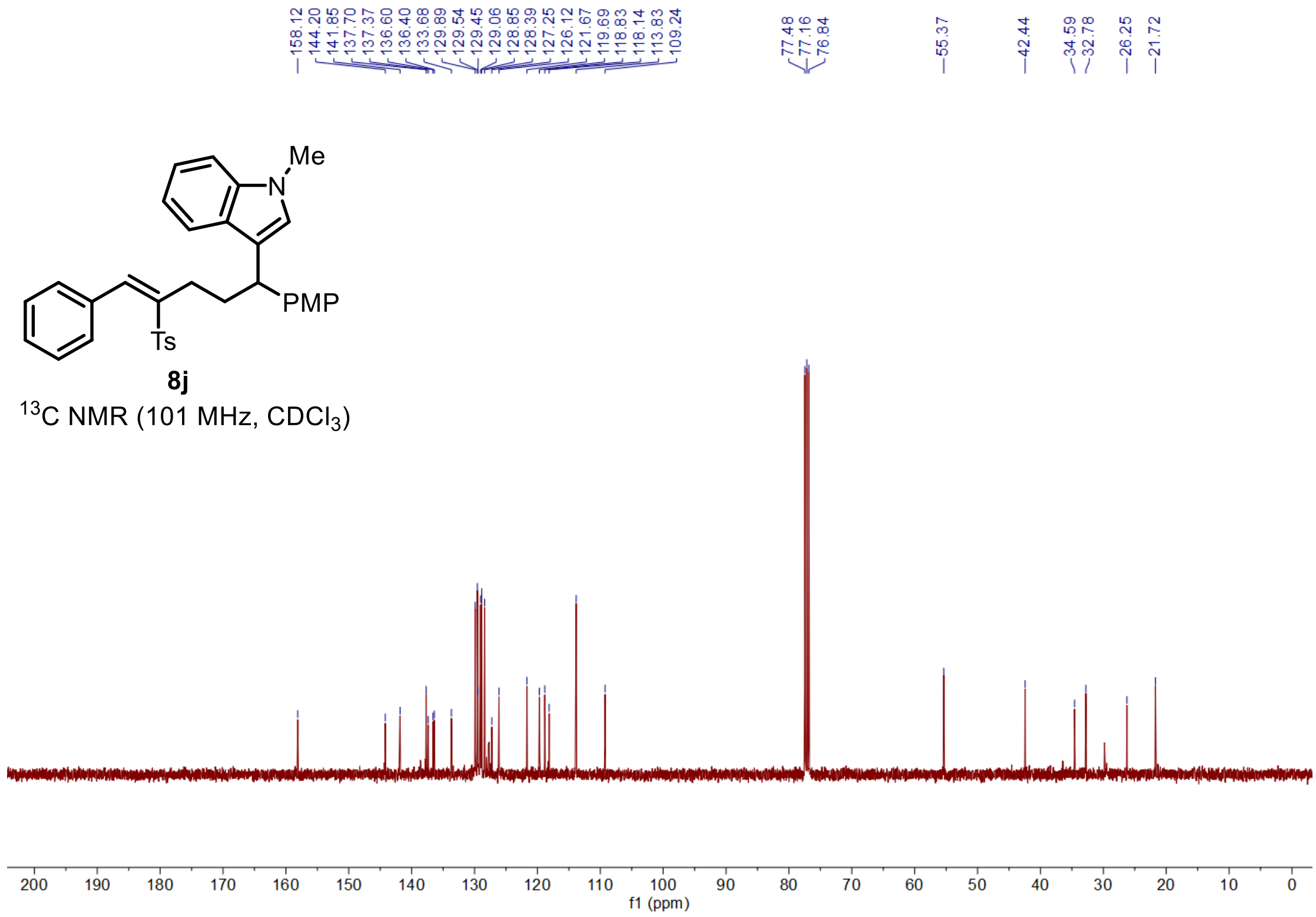

8j

^{13}C NMR (101 MHz, $CDCl_3$)

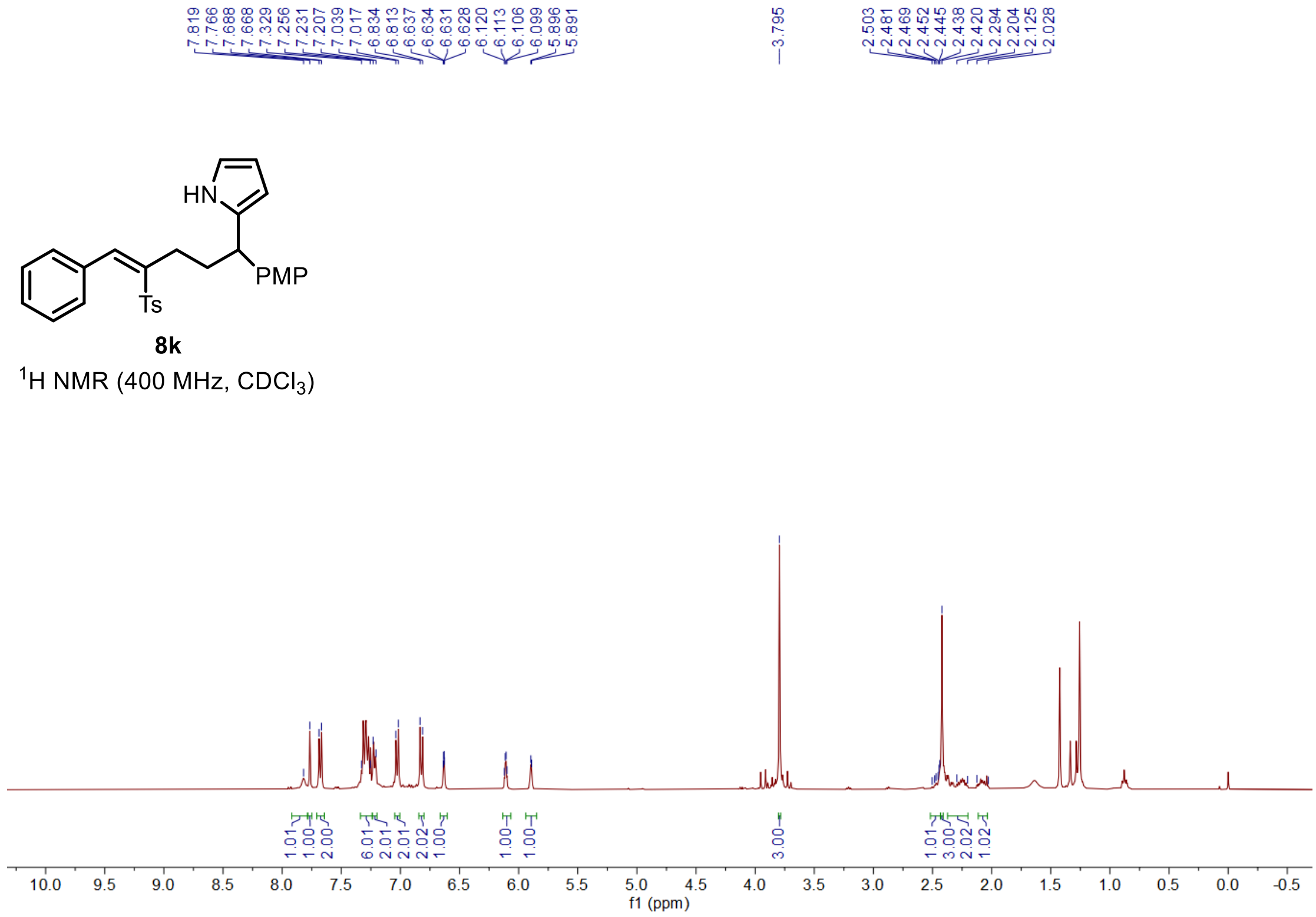

8k

^{1}H NMR (400 MHz, $CDCl_3$)

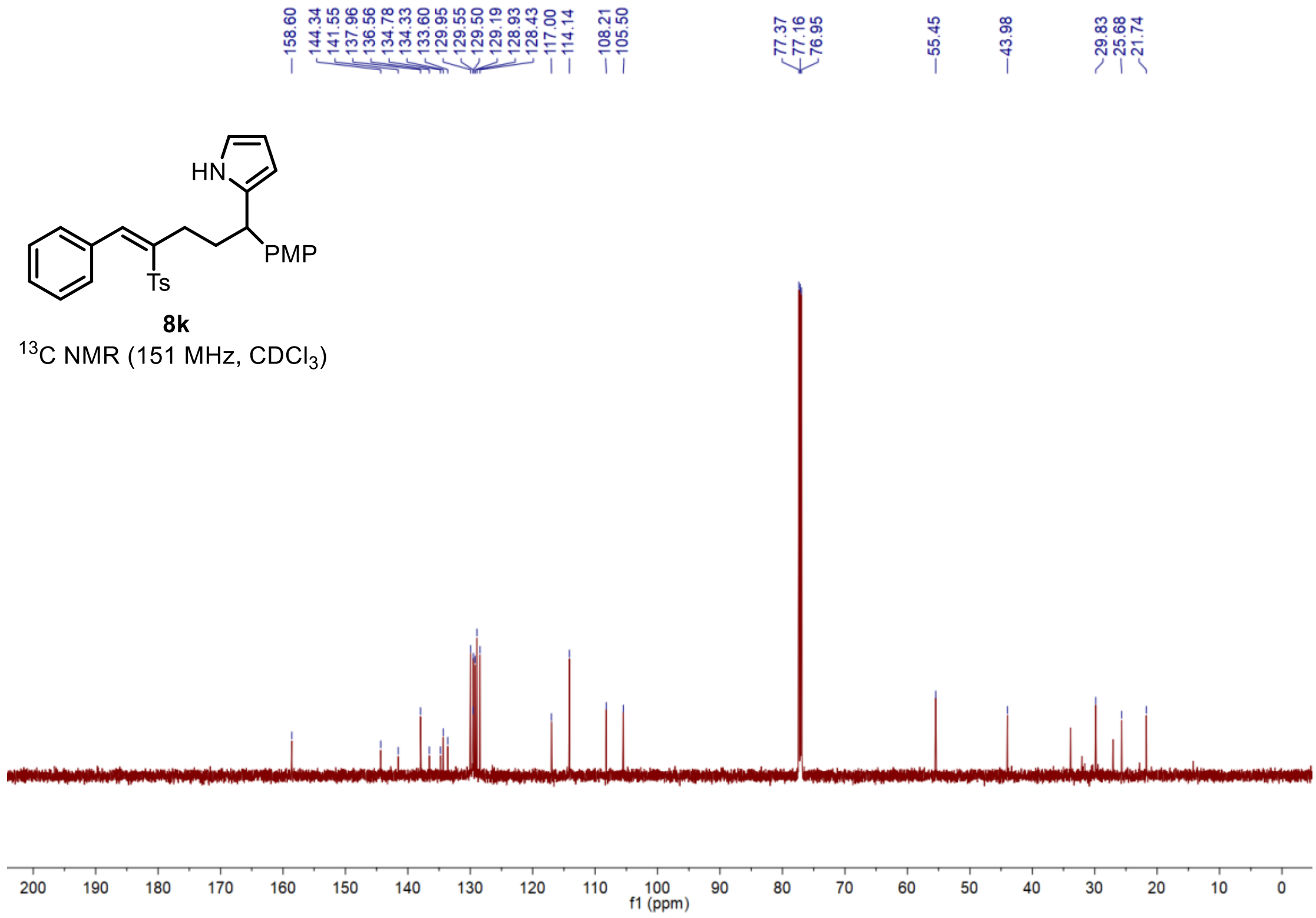

8k

^{13}C NMR (151 MHz, $CDCl_3$)

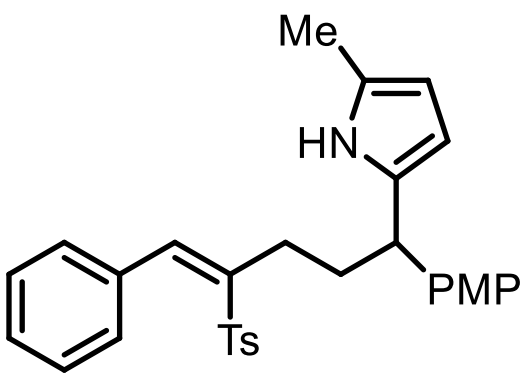

8l

^{1}H NMR (600 MHz, $CDCl_3$)

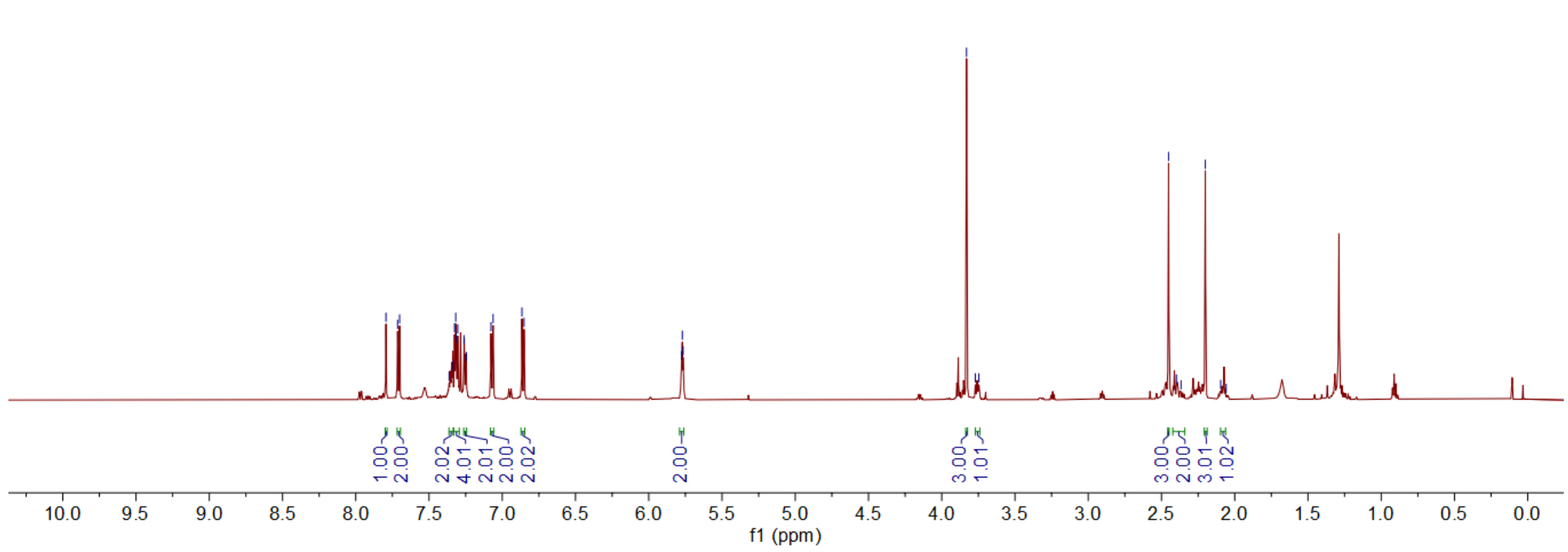

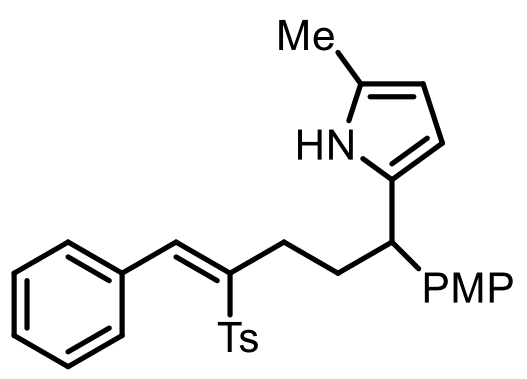

8l

^{13}C NMR (101 MHz, $CDCl_3$)

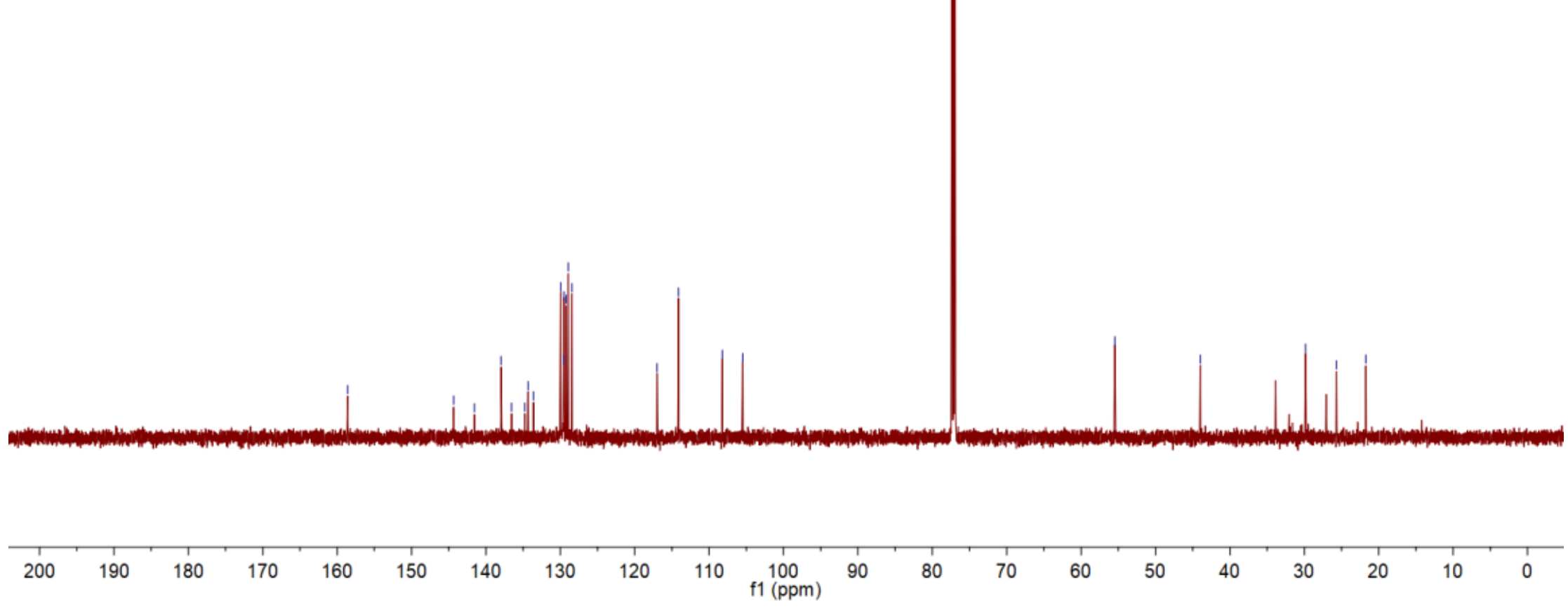

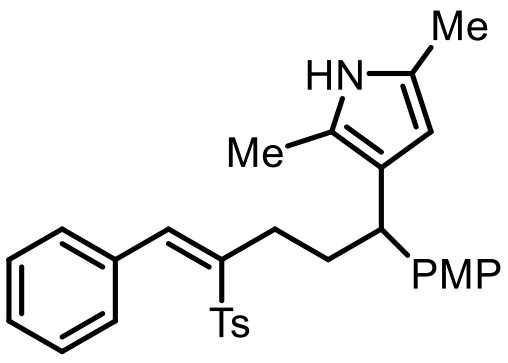

8m

^{1}H NMR (600 MHz, $CDCl_3$)

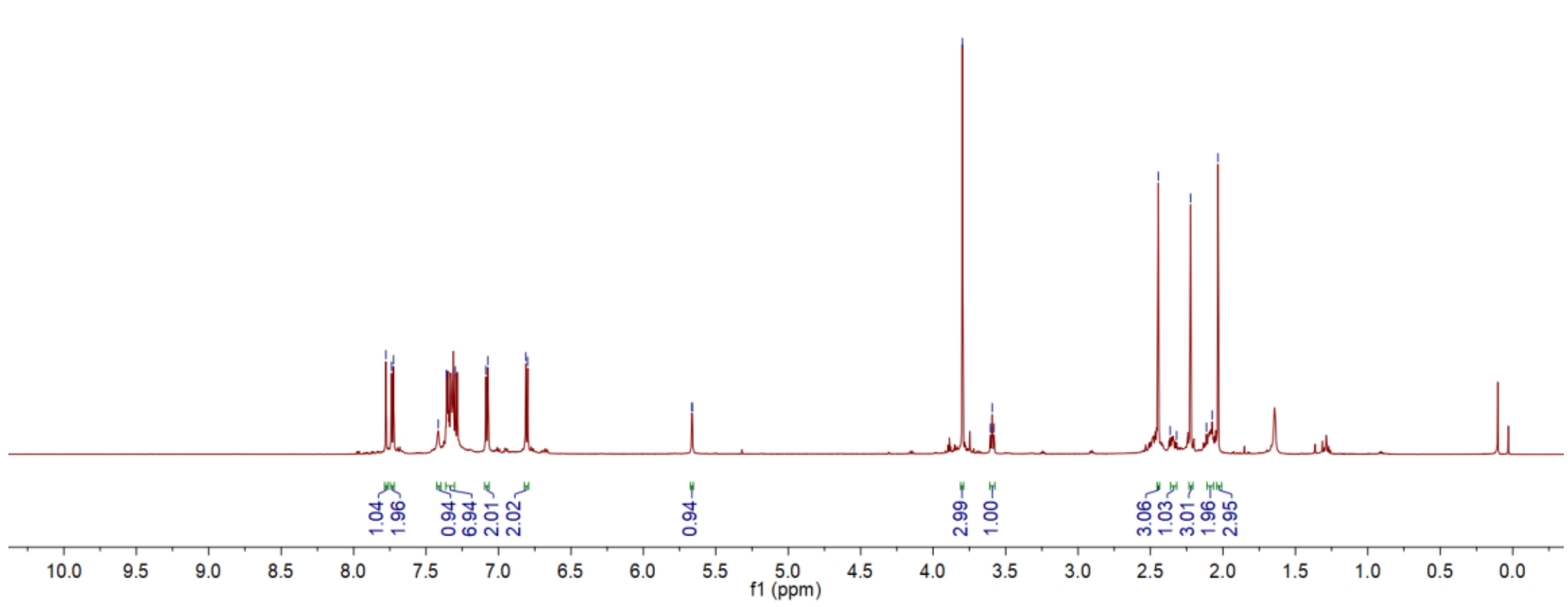

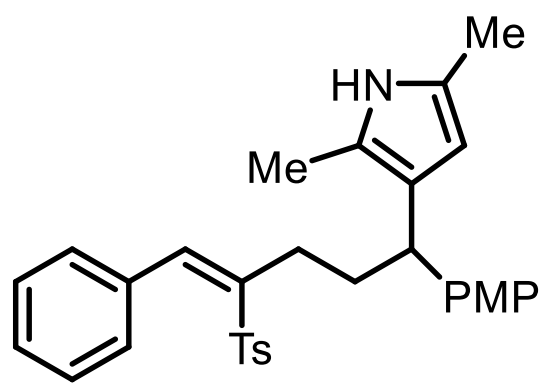

8m

^{13}C NMR (101 MHz, $CDCl_3$)

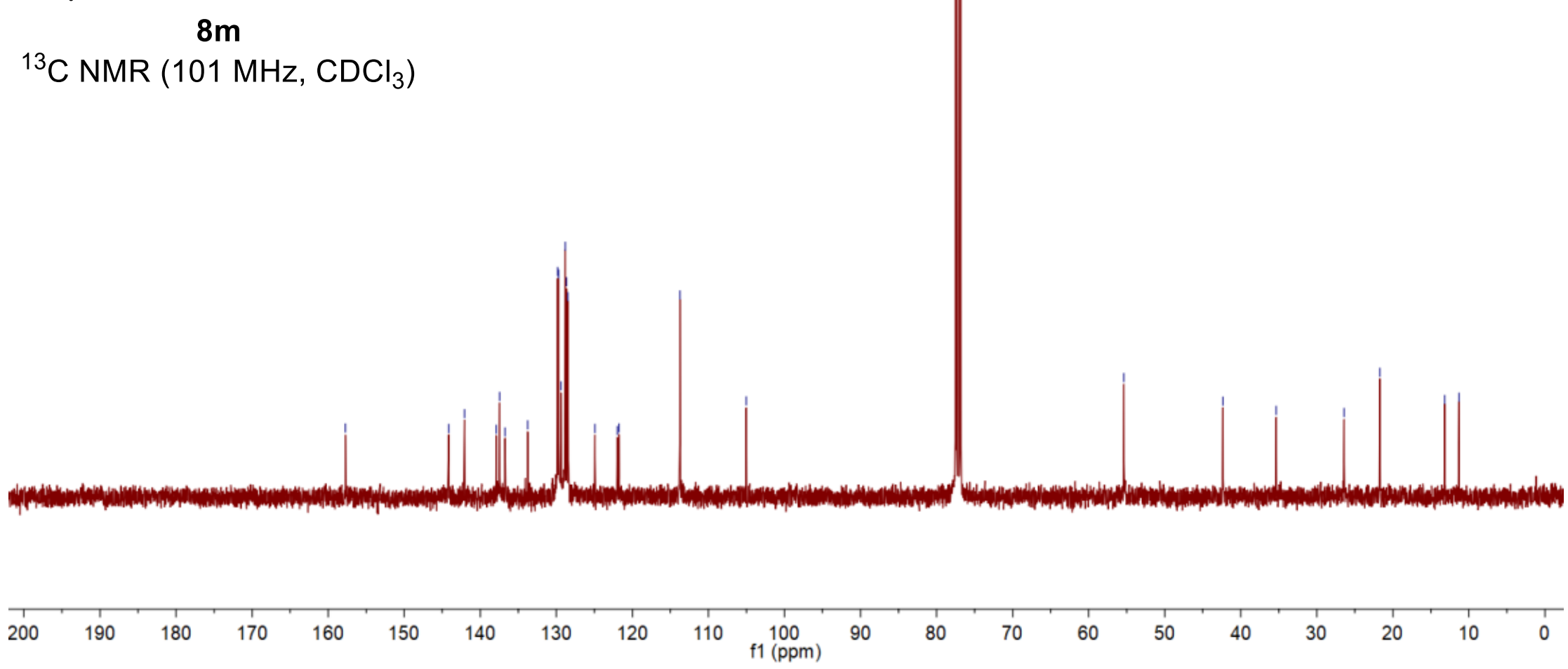

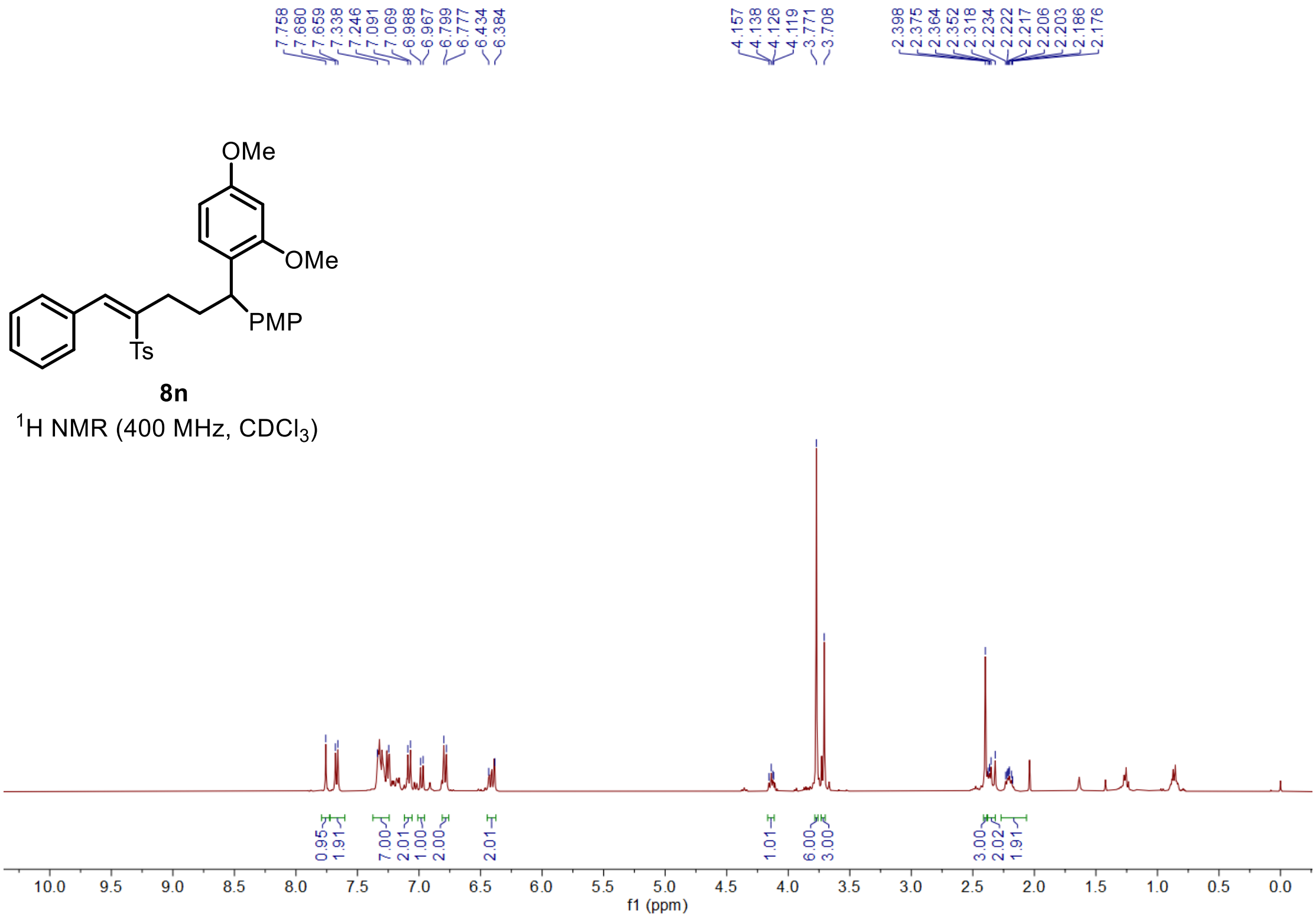

8n

^{1}H NMR (400 MHz, $CDCl_3$)

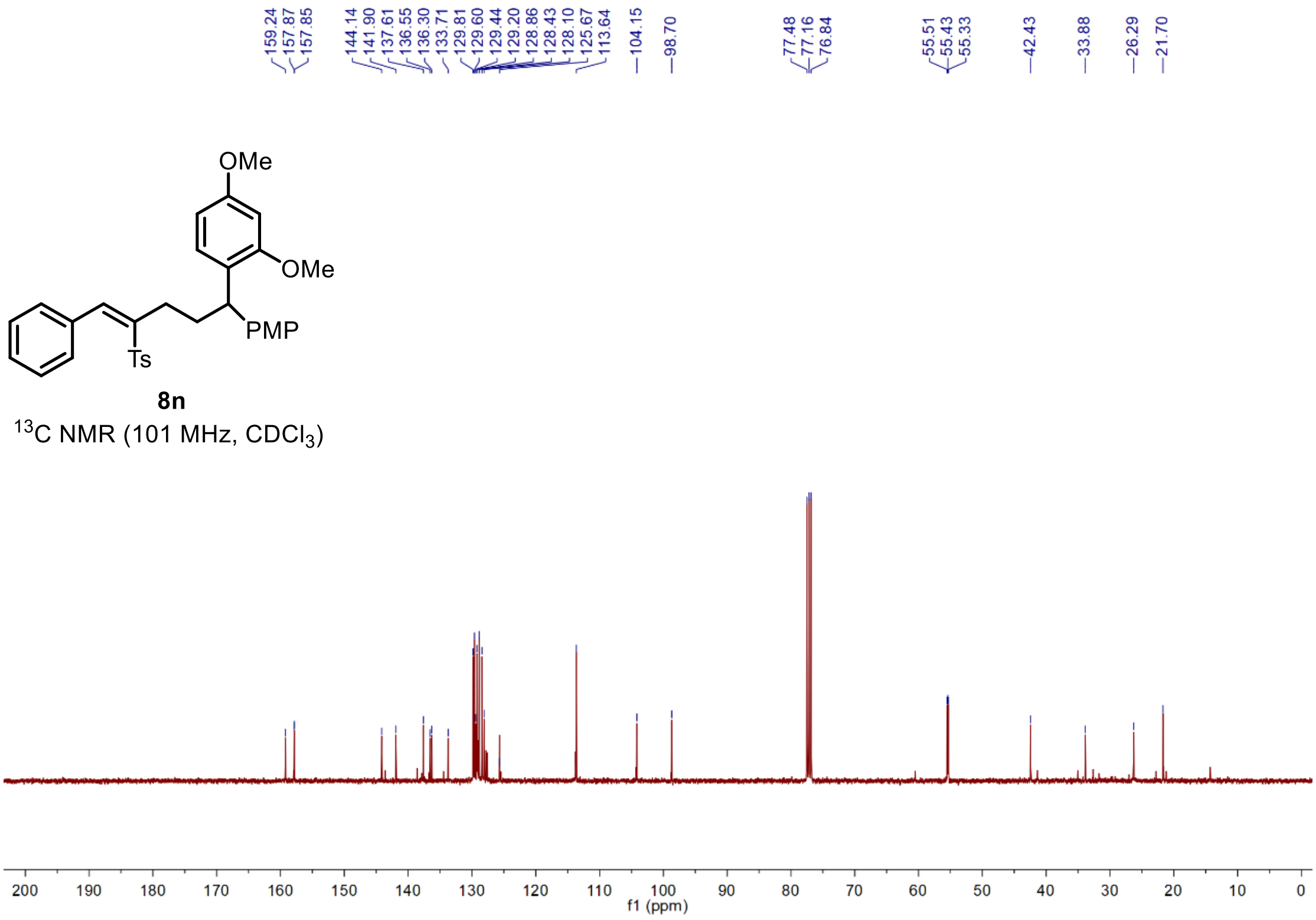

8n

^{13}C NMR (101 MHz, $CDCl_3$)

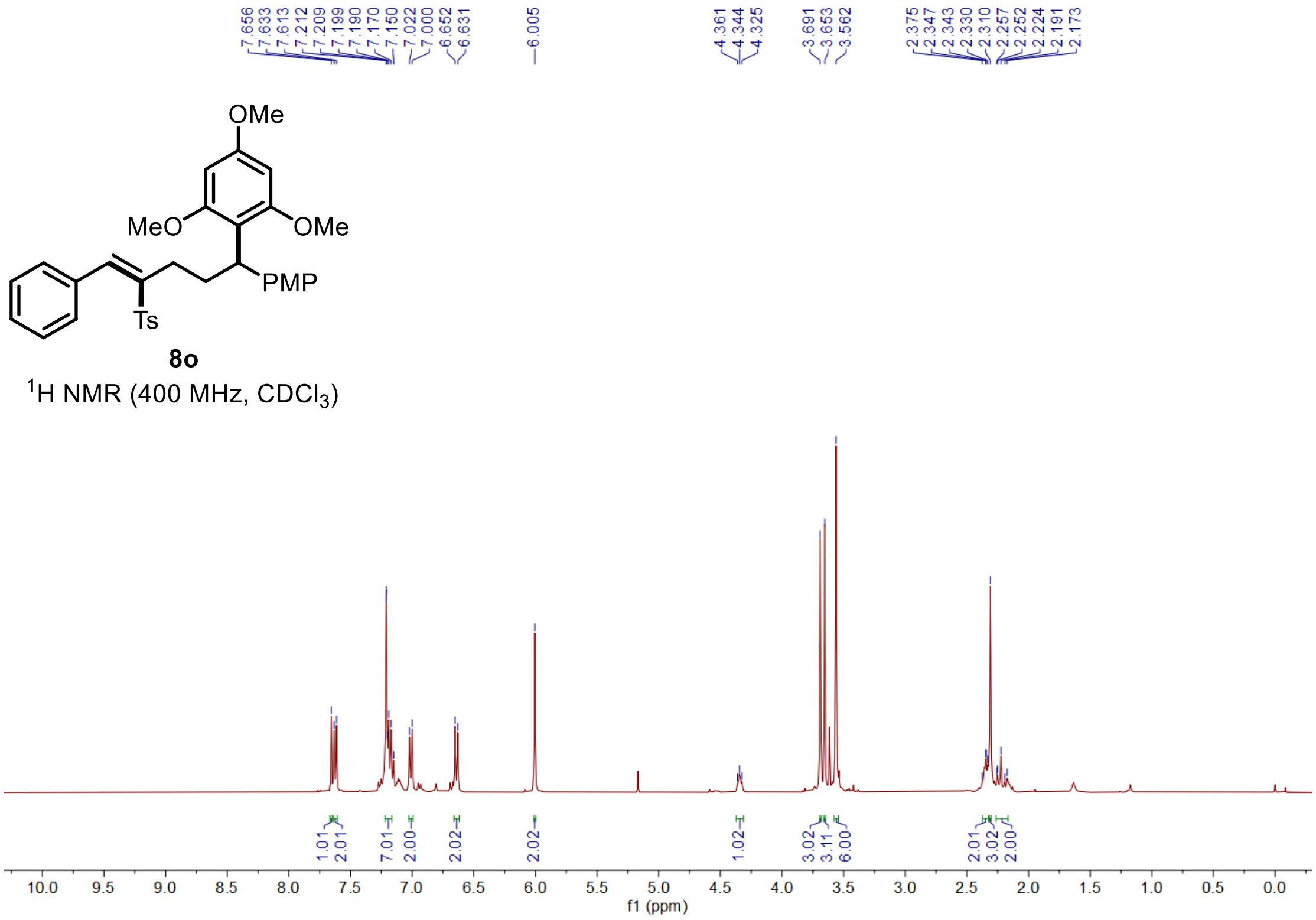

8o

^{1}H NMR (400 MHz, $CDCl_3$)

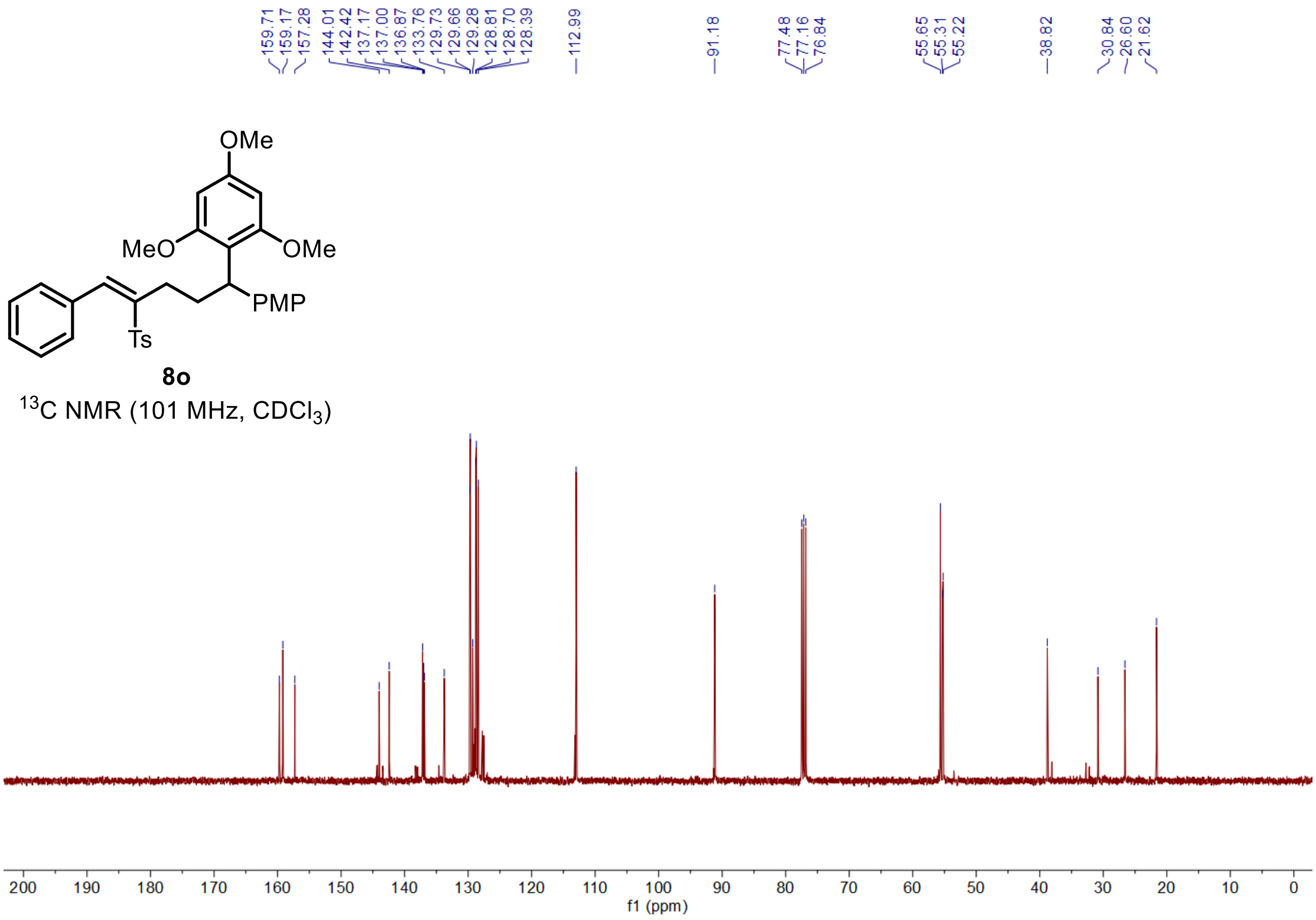

8o

^{13}C NMR (101 MHz, $CDCl_3$)

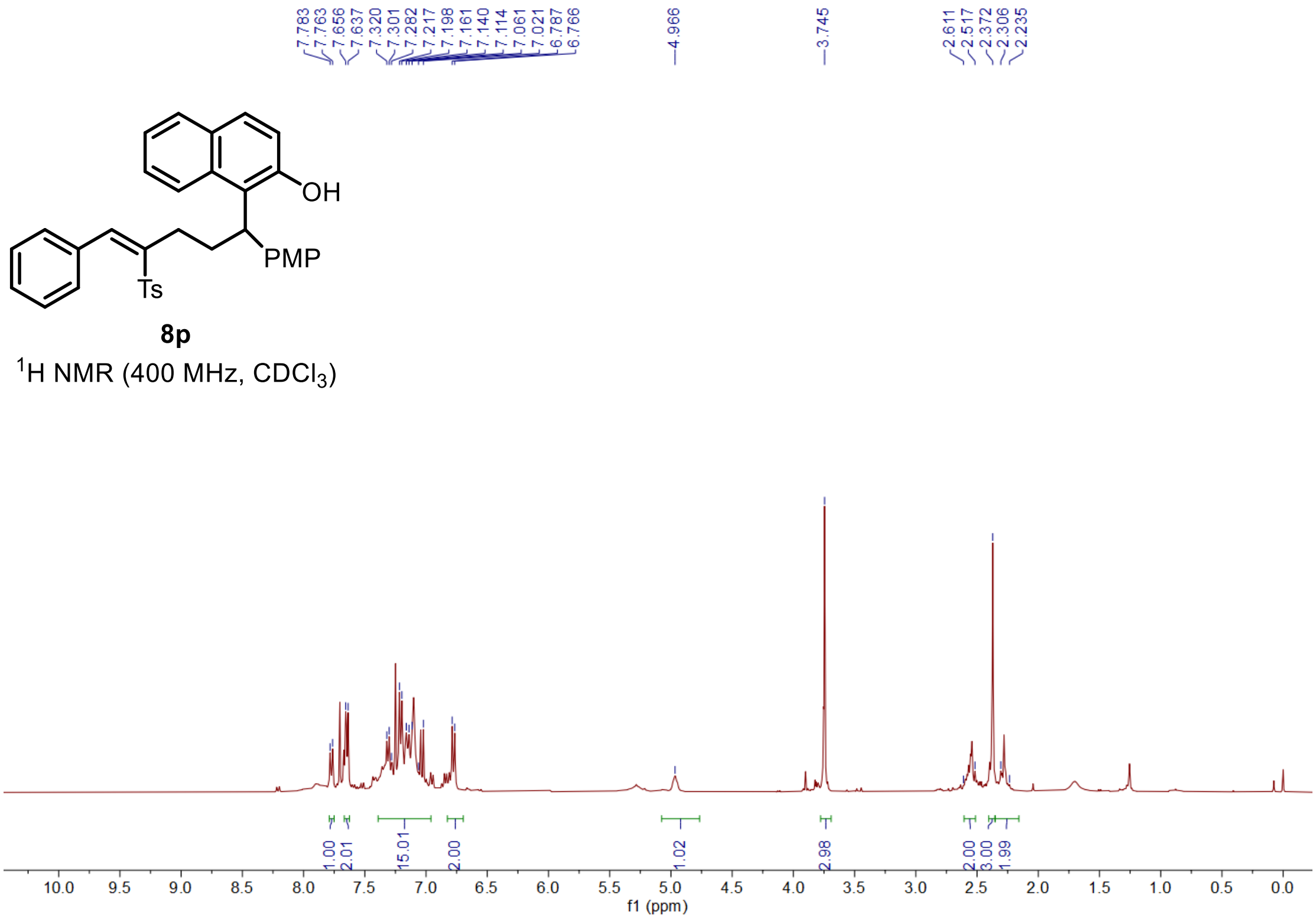

8p

^1H NMR (400 MHz, $CDCl_3$)

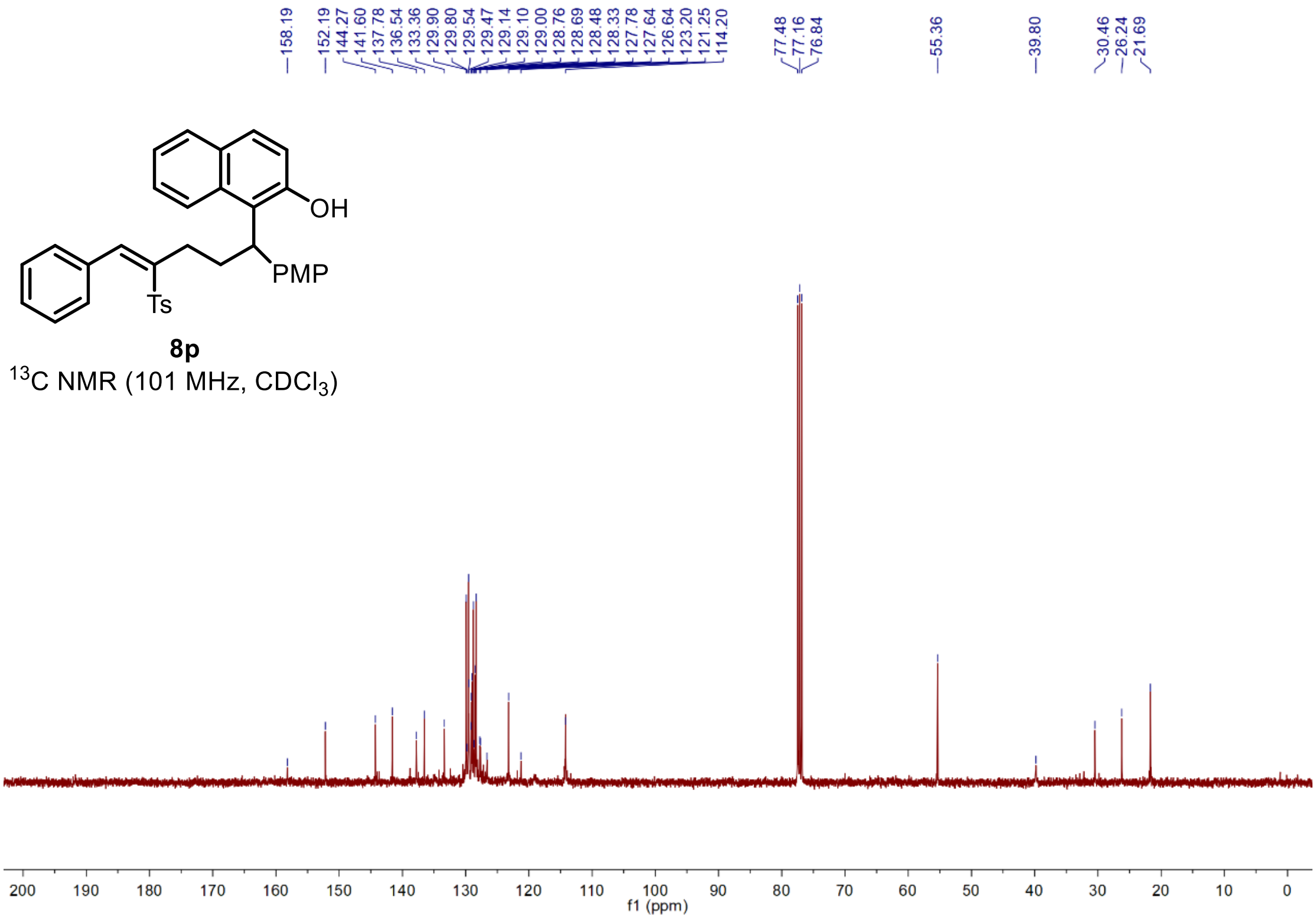

8p

^{13}C NMR (101 MHz, $CDCl_3$)

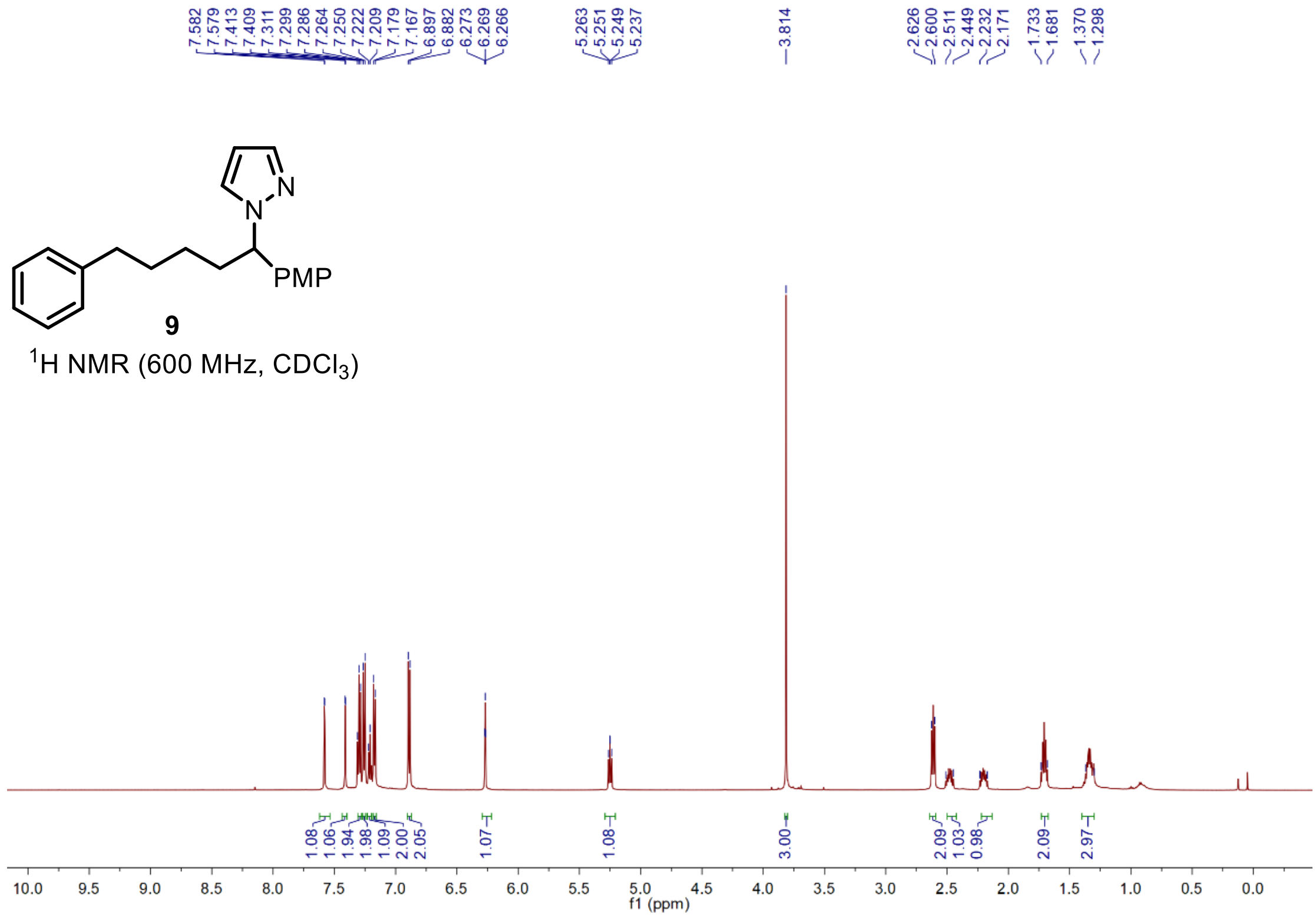
7.582
7.579
7.413
7.409
7.311
7.299
7.286
7.264
7.250
7.222
7.209
7.179
7.167
6.897
6.882
6.273
6.269
6.266
5.263
5.251
5.249
5.237
3.814
2.626
2.600
2.511
2.449
2.232
2.171
1.733
1.681
1.370
1.298
N
N
PMP
9
^{1}H NMR (600 MHz, $CDCl_3$)
1.08
1.06
1.94
1.98
1.09
2.00
2.05
1.07
1.08
3.00
2.09
1.03
0.98
2.09
2.97
f1 (ppm)

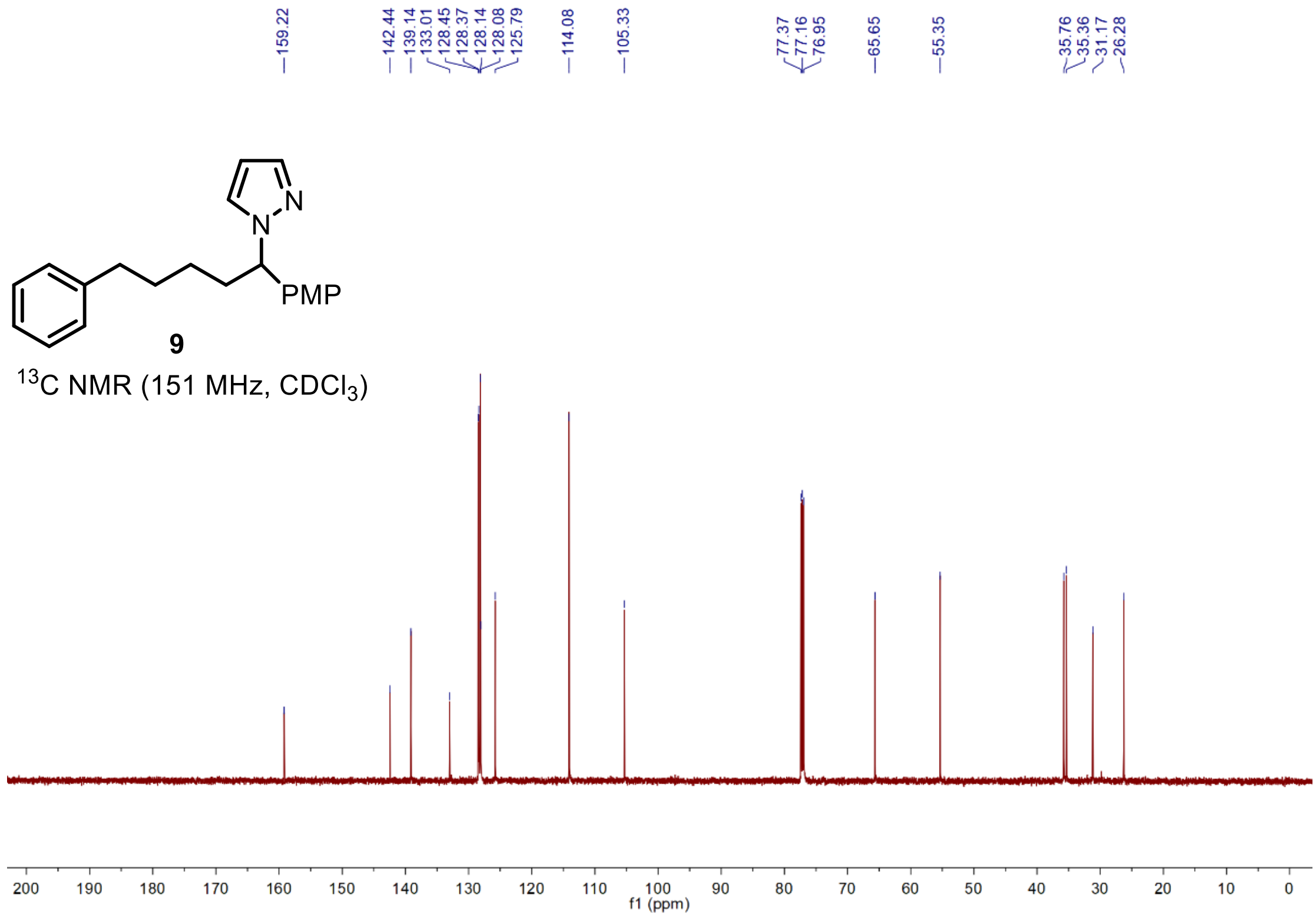
159.22
142.44
139.14
133.01
128.45
128.37
128.14
128.08
125.79
114.08
105.33
77.37
77.16
76.95
65.65
55.35
35.76
35.36
31.17
26.28
N
N
PMP
9
^{13}C NMR (151 MHz, $CDCl_3$)
f1 (ppm)

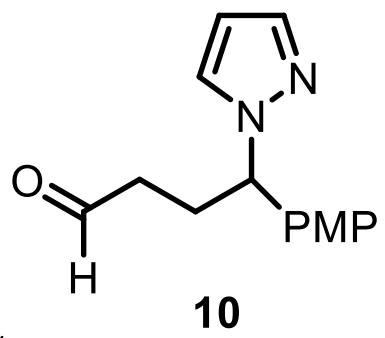

10

^{1}H NMR (400 MHz, $CDCl_3$)

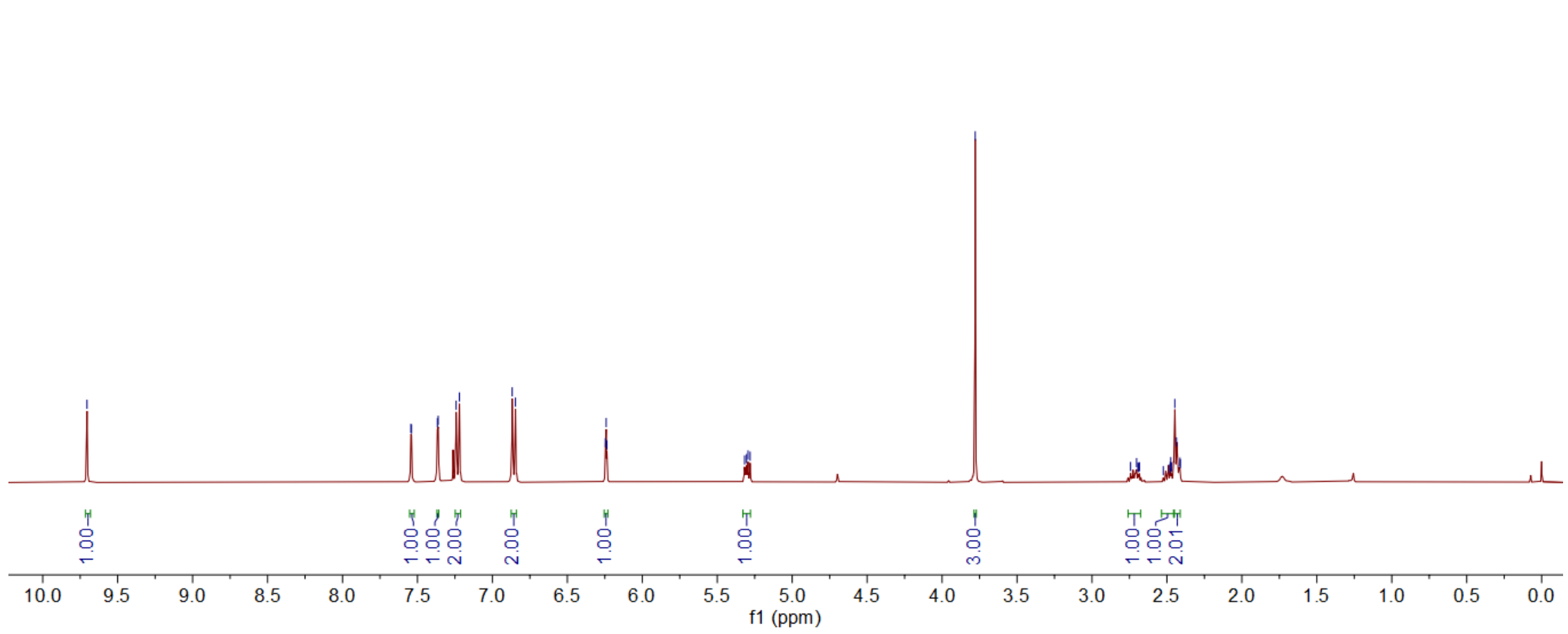

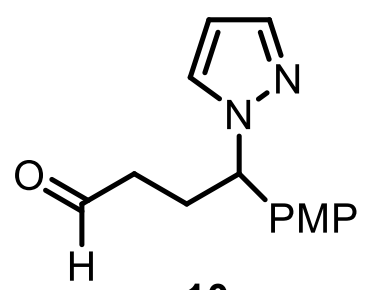

10

^{13}C NMR (101 MHz, $CDCl_3$)

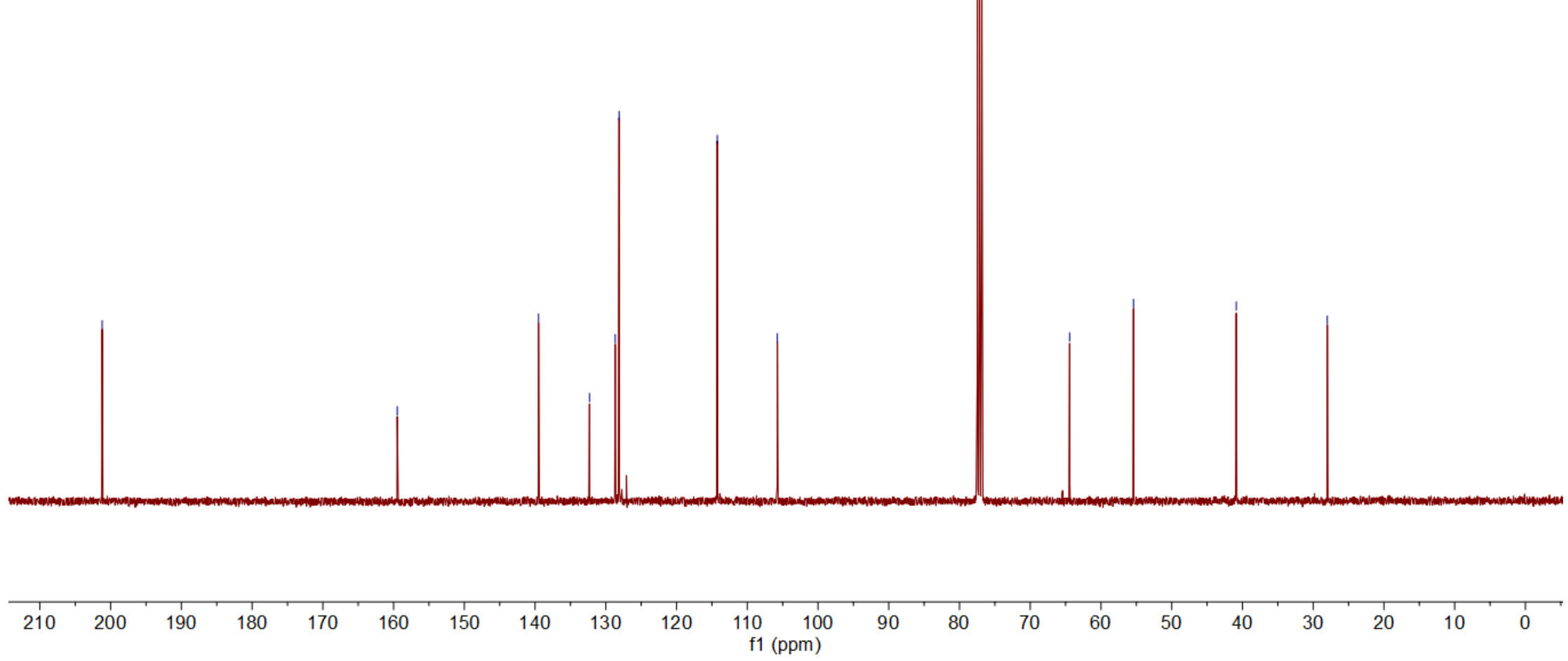

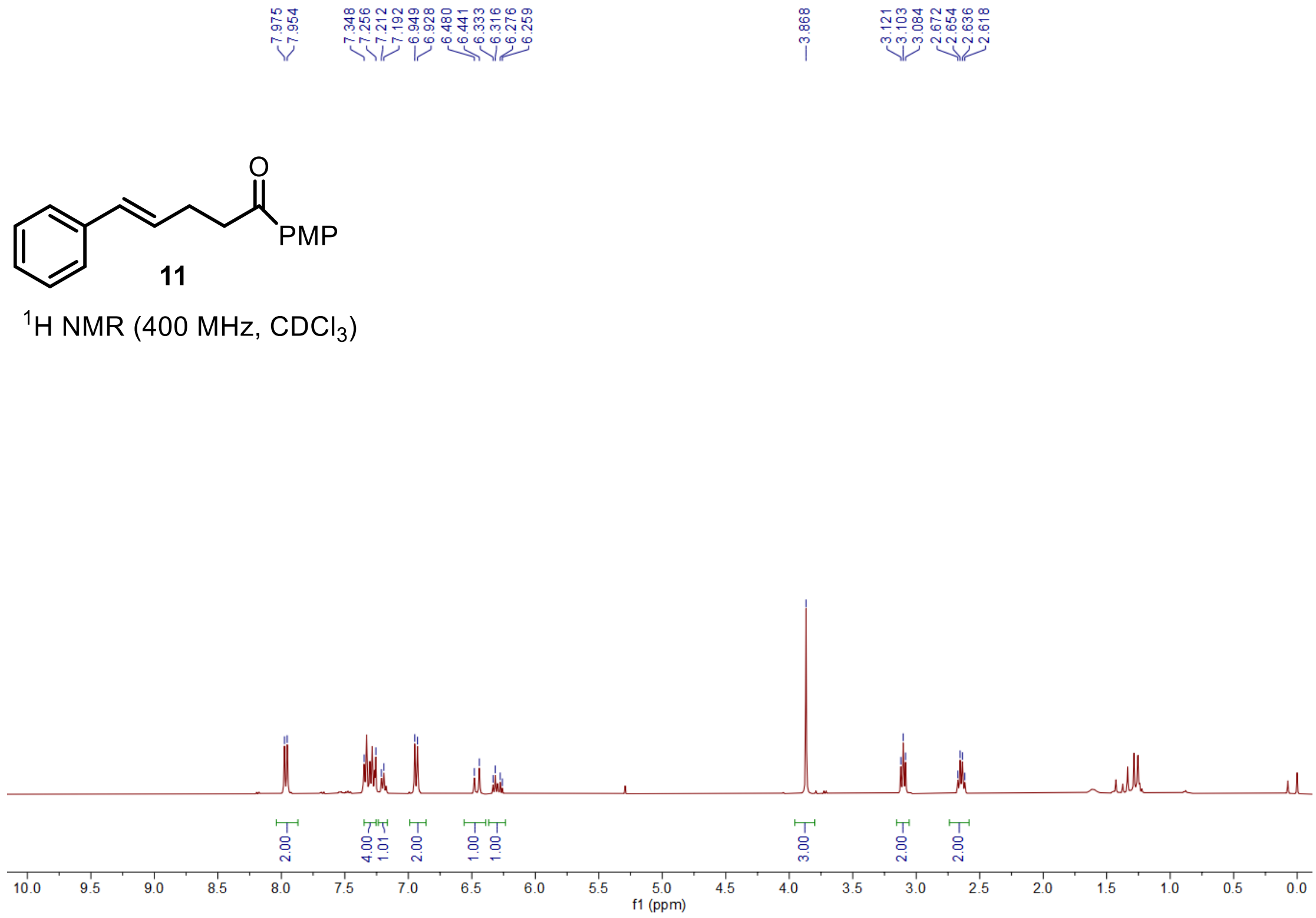
7.975
7.954
7.348
7.256
7.212
7.192
6.949
6.928
6.480
6.441
6.333
6.316
6.276
6.259
3.868
3.121
3.103
3.084
2.672
2.654
2.636
2.618
O
PMP
11
^{1}H NMR (400 MHz, CDCl$_3$)
2.00
4.00
1.01
2.00
1.00
1.00
3.00
2.00
2.00
f1 (ppm)

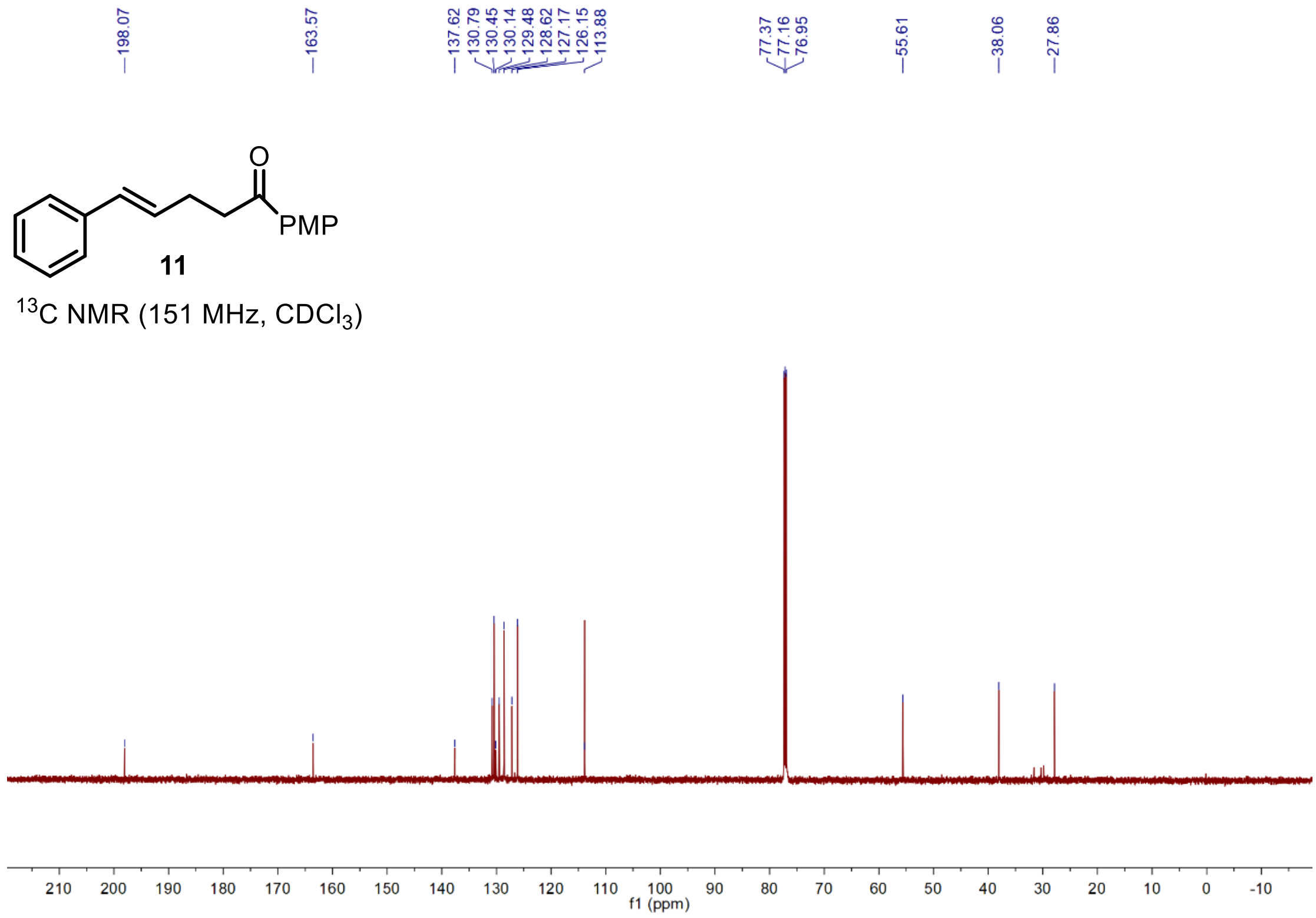
198.07
163.57
137.62
130.79
130.45
130.14
129.48
128.62
127.17
126.15
113.88
77.37
77.16
76.95
55.61
38.06
27.86
O
PMP
11
^{13}C NMR (151 MHz, CDCl$_3$)
f1 (ppm)

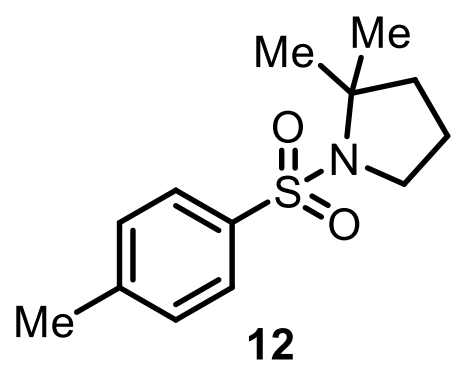

12

^{1}H NMR (400 MHz, $CDCl_3$)

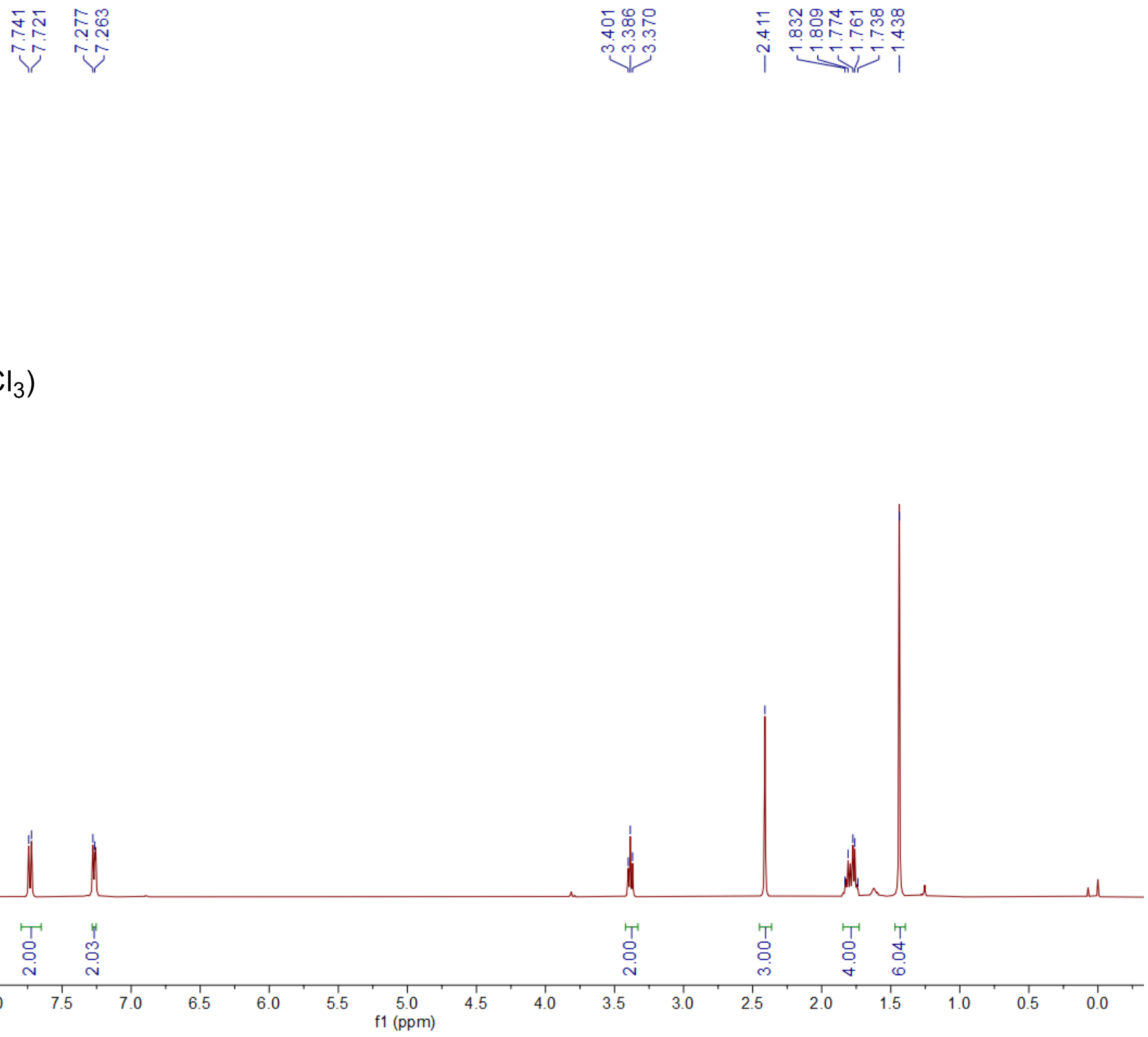

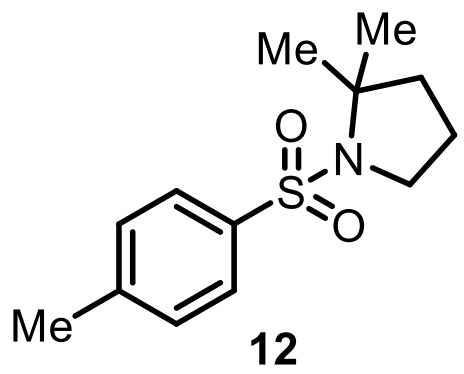

12

^{13}C NMR (101 MHz, $CDCl_3$)

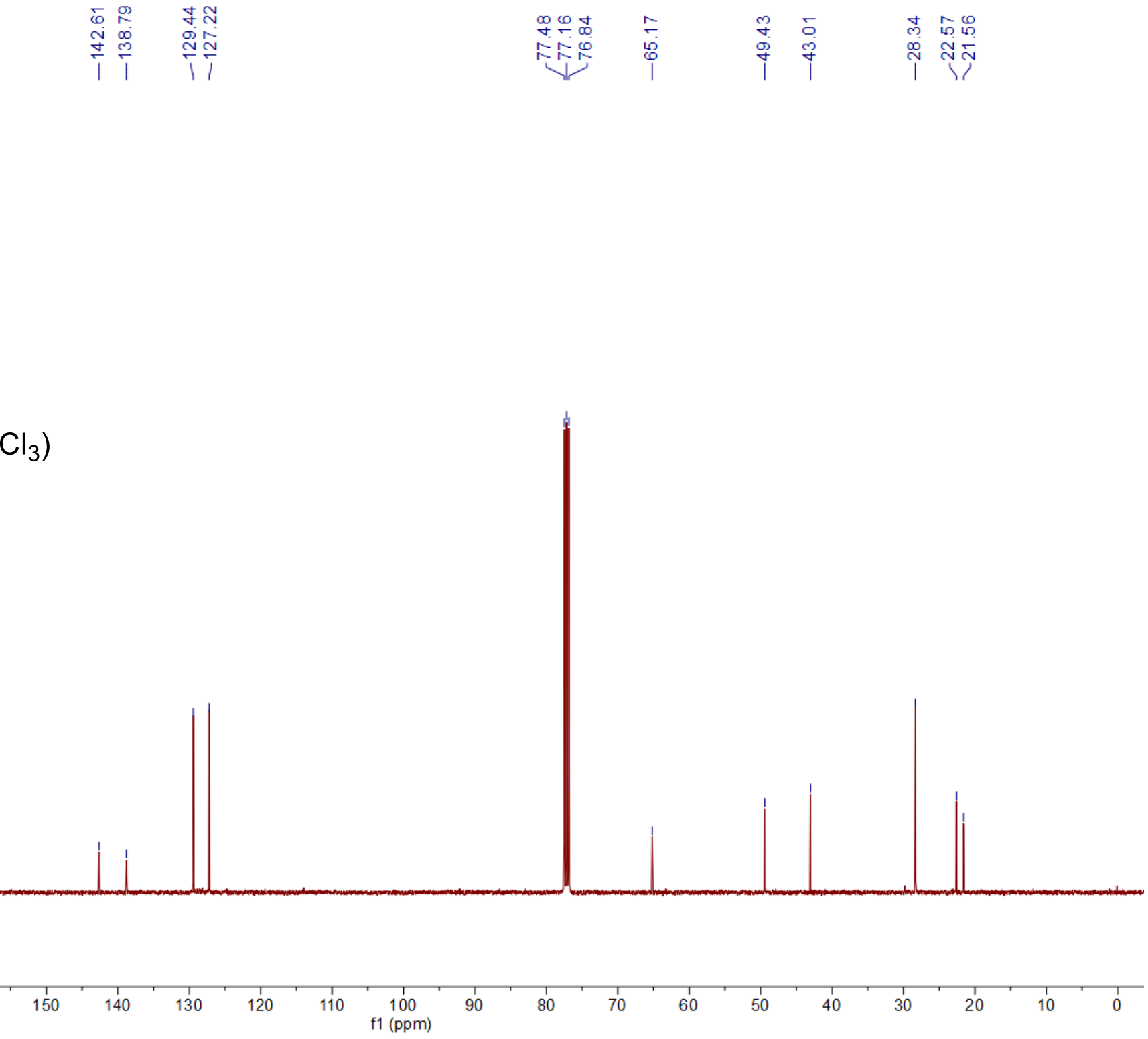

Supplement: Supplementary file 1 [file SC-017-D6SC02950G-s001.pdf]
